# Supplementary material for: Cellular location shapes quaternary structure of enzymes
Source: Nat Commun. 2024 Oct 1;15:8505. doi: 10.1038/s41467-024-52662-2 (PMC11445431; doi:10.1038/s41467-024-52662-2)
Supplement: Supplementary file 1 — Supplementary Information [file 41467_2024_52662_MOESM1_ESM.pdf]

Supplementary Notes and Figures to "Cellular location shapes quaternary structure of enzymes."

by György Abrusán and Aleksej Zelezniak

SUPPLEMENTARY FIGURES

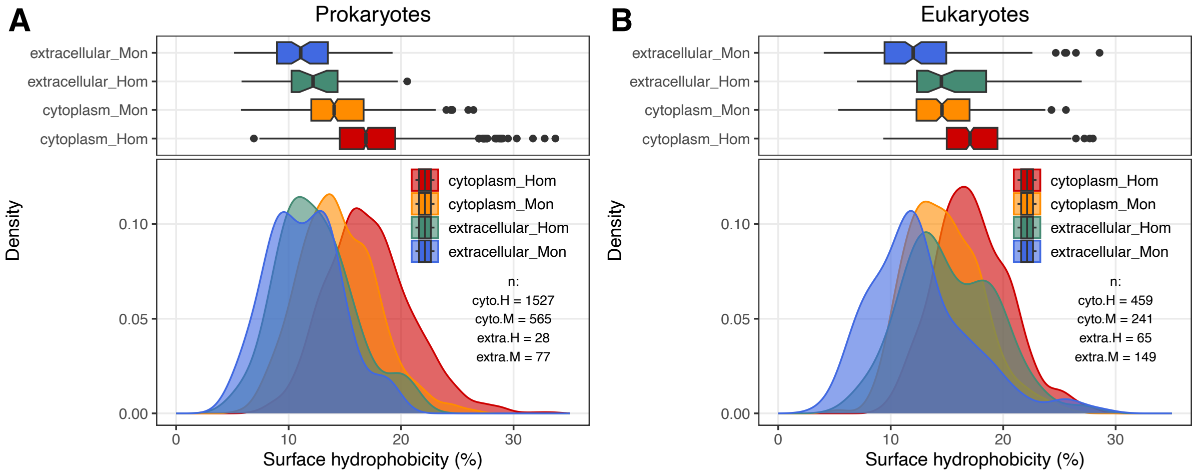

Supplementary Figure 1. Hydrophobicities of the full solvent-accessible surfaces of the homomer subunits and monomers. Hydrophobicity was calculated as the fraction of C,F,I,L,M,V,W residues in all residues with relative solvent accessibility above 0.2. In agreement with having a hydrophobic interface, subunits of cytoplasmic homomers have the highest hydrophobicity, while extracellular monomers have the lowest hydrophobicity, both in Prokaryotes and Eukaryotes, indicating that they do not have large hydrophobic patches on their surface. Thus, extracellular monomers are unlikely to be able to form stable homomers that are absent in the PDB. On both panels boxplots display the median, 25-75% interquartile range (IQR), and 1.5 * interquartile range from the hinge (whiskers). Notches are defined as 1.58 * IQR / sqrt(n). Datapoints beyond the whiskers are shown as outliers.

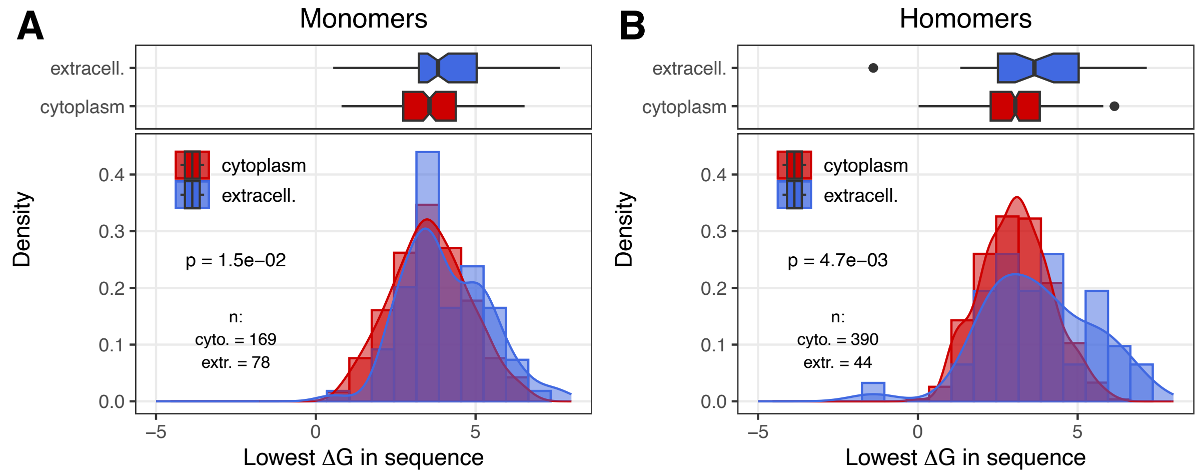

Supplementary Figure 2. In mammals, neither homomers nor monomers have transmembrane helices. The presence of transmembrane helices in mammalian proteins was predicted with the method of Hessa et al. [2]. Cytoplasmic and extracellular proteins are expected to have the free energy of insertion of a TM helix into the endoplasmic reticulum membrane (ΔG) above 0 (thus inserting the protein into a membrane needs energy), while membrane proteins have negative ΔG. The ΔG values in our dataset are positive in the vast majority of cases and extracellular proteins have somewhat higher values than cytoplasmic proteins (Wilcoxon rank sum tests), indicating that these proteins generally cannot be inserted into membranes. On both panels boxplots display the median, 25-75% interquartile range (IQR), and 1.5 * interquartile range from the hinge (whiskers). Notches are defined as 1.58 * IQR / sqrt(n). Datapoints beyond the whiskers are shown as outliers.

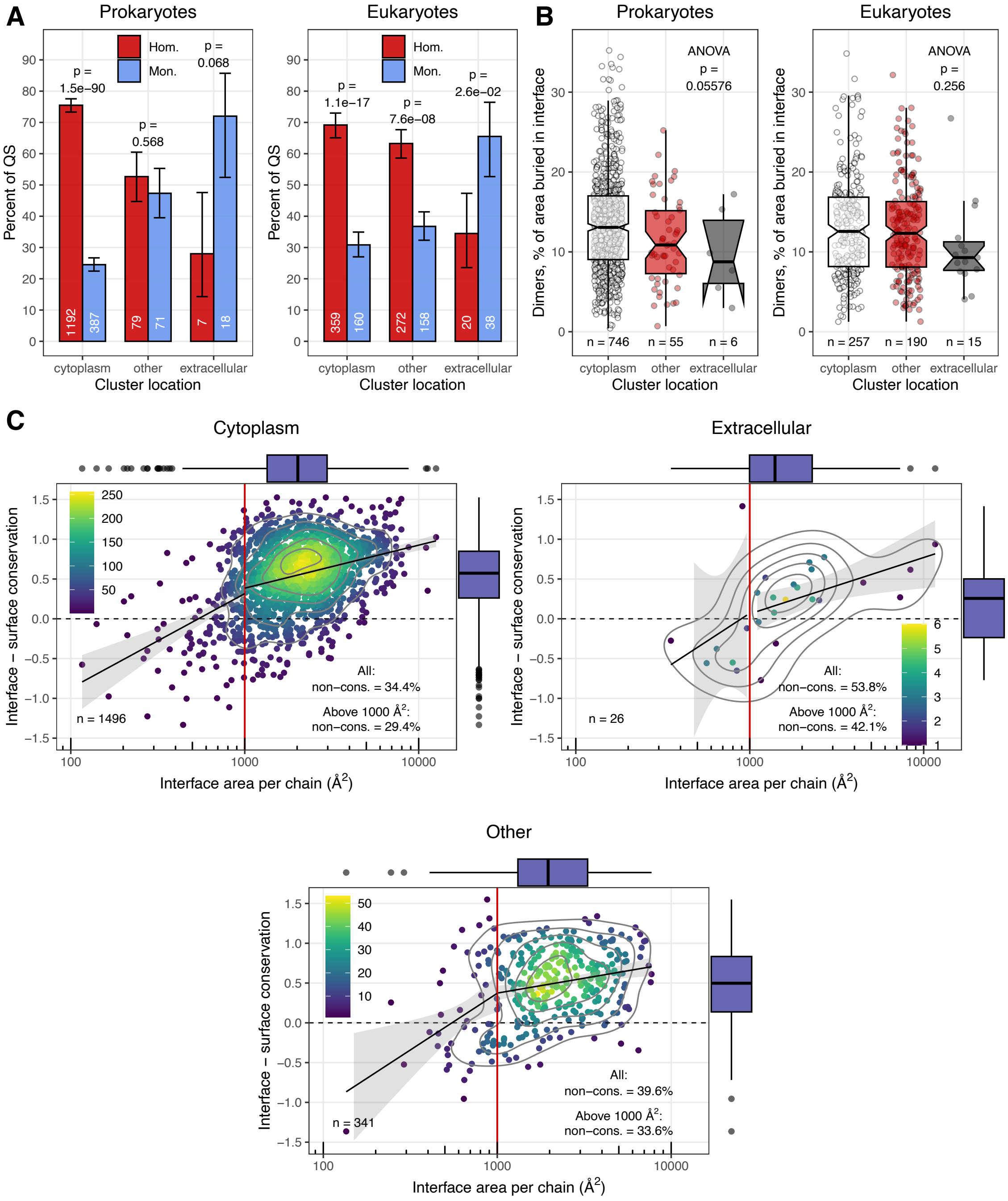

Supplementary Figure 3. The enrichment of monomers in extracellular enzymes, and their interface characteristics remain qualitatively similar when hydrolases are excluded. **A)** The frequency of homomers and monomers in the cytoplasm, extracellular space, and other cellular components. Bars indicate the fraction of quaternary structure in the set, whiskers represent 95% CI; p-values were calculated with tests of proportions, and were corrected with the Benjamini-Hochberg method. **B)** Relative surface areas of homodimers. **C)** Conservation of interfaces. The overall patterns without hydrolases are the same as with them, but due to the much lower number of extracellular proteins, significances are affected. On panels B and C boxplots display the median, 25-75% interquartile range (IQR), and 1.5 * interquartile range from the hinge (whiskers). Notches are defined as 1.58 * IQR / sqrt(n). Datapoints beyond the whiskers are shown as outliers.

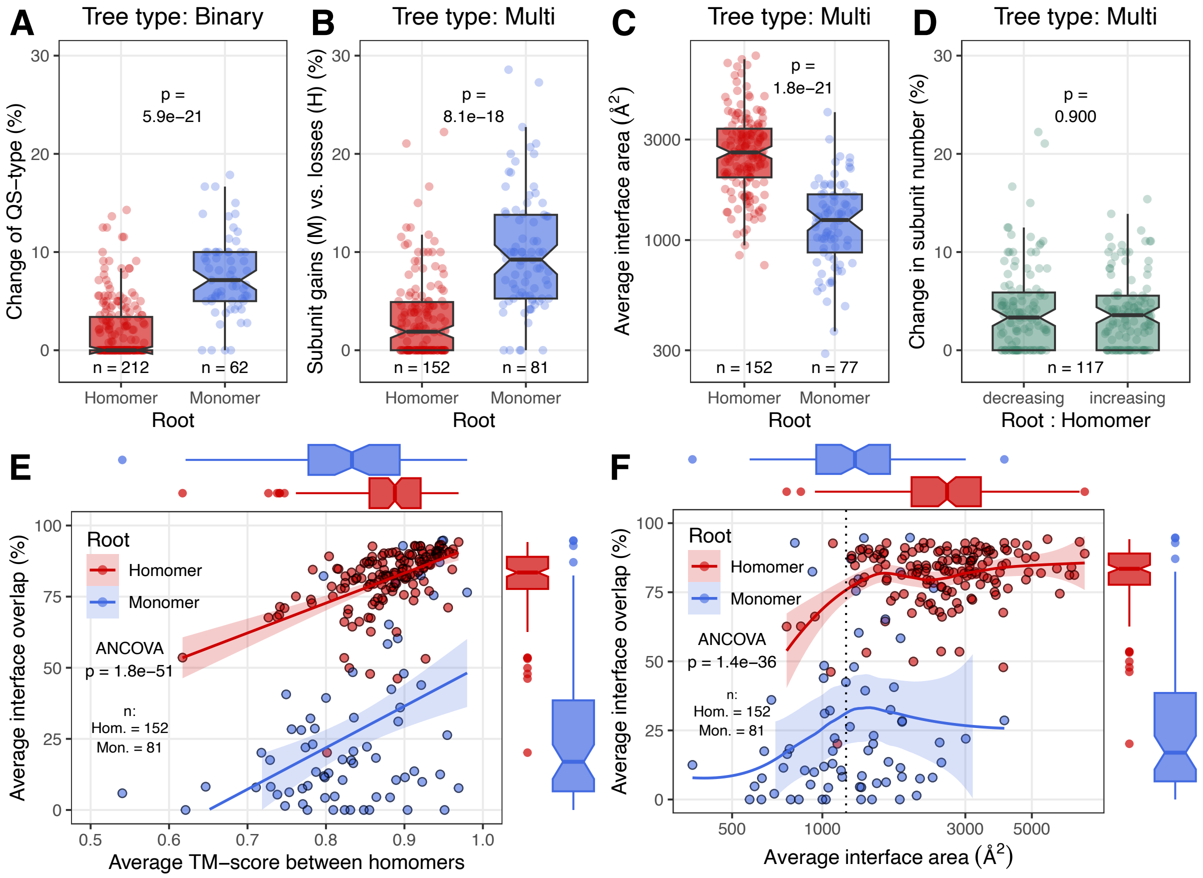

Supplementary Figure 4. The same as Figure 4, but using maximum parsimony for ancestral character reconstruction. **A-B)** The patterns are essentially similar to the ones obtained with maximum likelihood reconstructions: monomers gain interfaces/subunits easier than they are being lost in homomers; **C)** homomers in trees with monomer roots have small interfaces; **D)** in trees with homomer root subunit gains and losses are comparable; **E-F)** new interfaces in orthogroups with monomer root are frequently not homologous and show little overlap. On all panels boxplots display the median, 25-75% interquartile range (IQR), and 1.5 * interquartile range from the hinge (whiskers). Notches are defined as 1.58 * IQR / sqrt(n). Datapoints beyond the whiskers are shown as outliers. On panels A-D p-values were calculated with Wilcoxon rank sum tests.

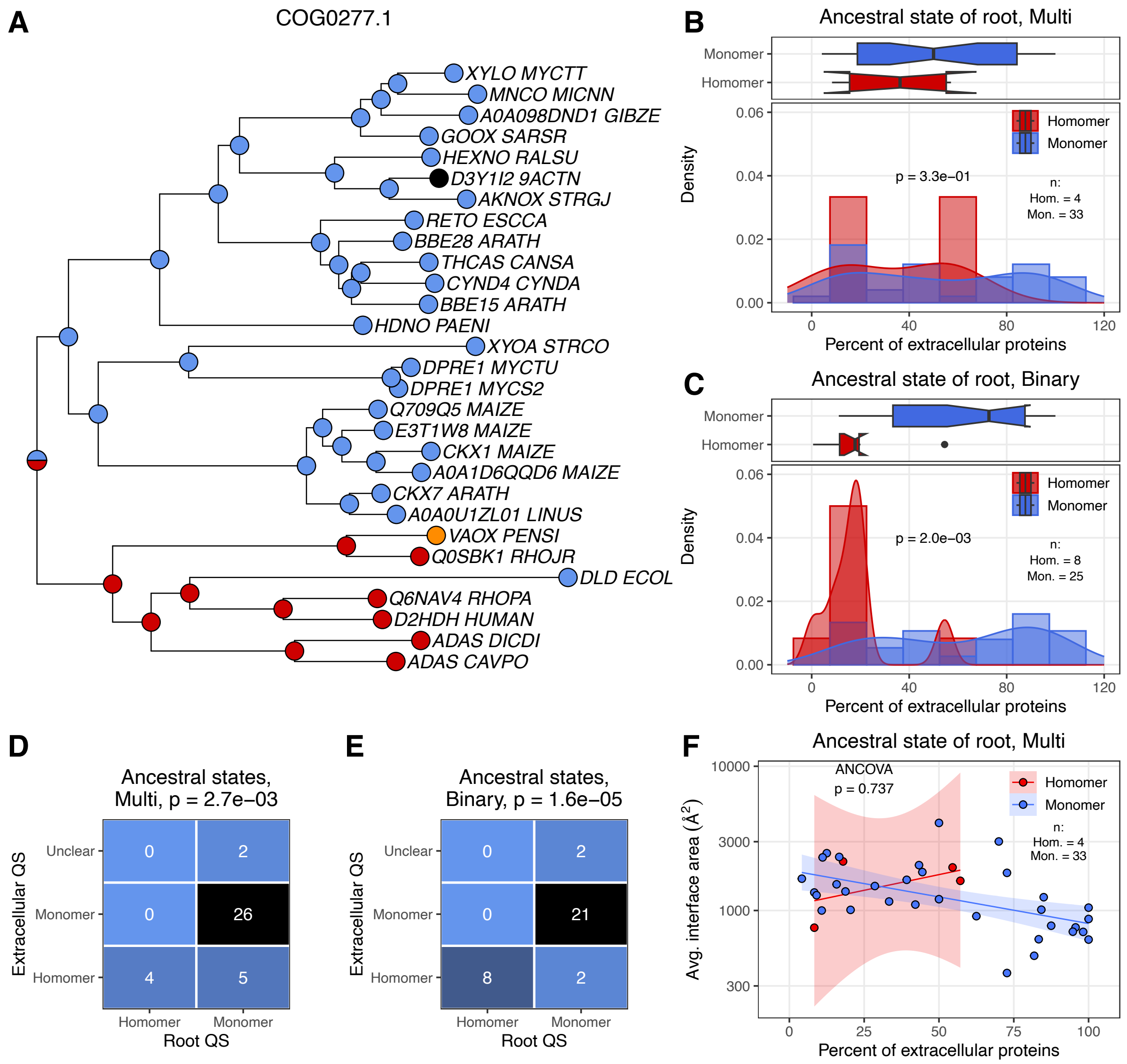

Supplementary Figure 5. The same as Figure 5, but using maximum parsimony for ancestral character reconstruction. **A)** Ancestral state reconstruction for COG0277. For this tree, MP results in similar probabilities of being a homomer or monomer at the root. **B-C)** Orthogroups with monomer root have higher frequency of extracellular proteins than the ones with homomer root, but the difference is significant only in the binary analysis (largely due to the very small number of orthogroups with homomer root in the Multi-state analysis, resulting in low power). On both panels boxplots display the median, 25-75% interquartile range (IQR), and 1.5 * interquartile range from the hinge (whiskers). Notches are defined as 1.58 * IQR / sqrt(n). Datapoints beyond the whiskers are shown as outliers. P-values were obtained with Wilcoxon rank sum tests. **D-E)** The quaternary structure of extracellular proteins is largely similar to the root of the trees, and extracellular monomers are present primarily in orthogroups with monomer root. P-values were calculated with the tests of proportions. **F)** The relationship between interface size and the frequency of extracellular proteins does not differ significantly in the two groups, (however the power of the test is very low, due to the very small number of orthogroups with homomer root).

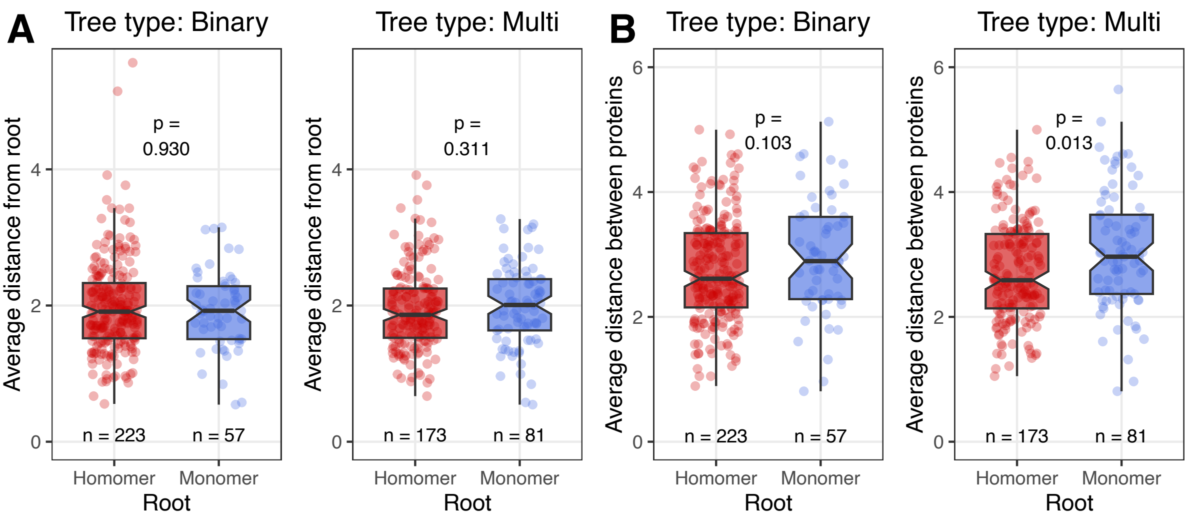

Supplementary Figure 6. General topological characteristics of the phylogenetic trees. **A)** Average distance of proteins from the root. **B)** average distance between proteins. On both panels boxplots display the median, 25-75% interquartile range from the hinge (IQR), and 1.5 * interquartile range (whiskers). Notches are defined as 1.58 * IQR / sqrt(n). P-values were calculated with Wilcoxon rank sum tests.

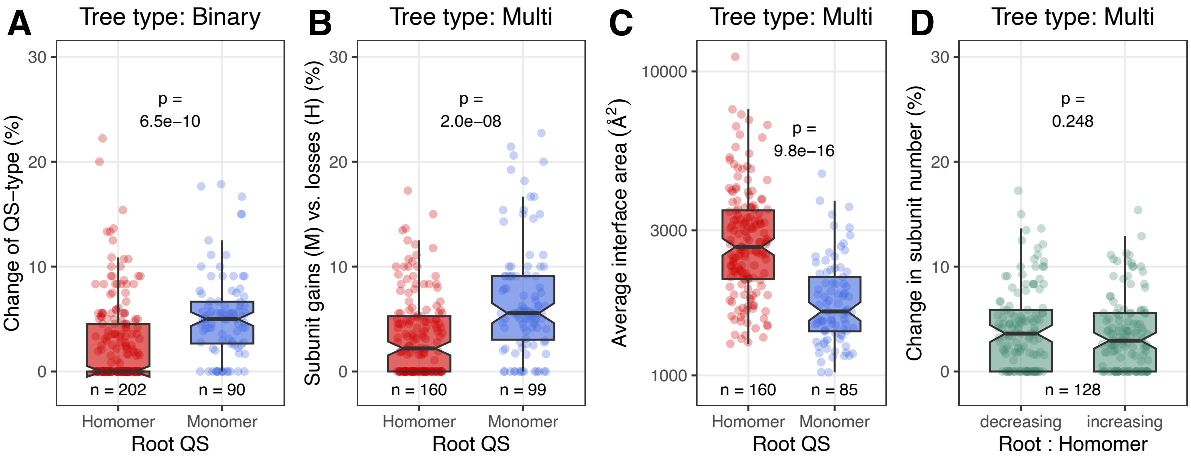

Supplementary Figure 7. When homomers with interface area below 1000 Å are assigned as monomers the patterns do not change qualitatively. **A)** The probability of changing quaternary structure (QS) type in trees with homomer and monomer root. **B)** The fraction of subunit losses and gains in the trees with different roots. **C)** Interface size in trees with homomer and monomer roots. **D)** The fraction of subunit number increases or decreases in the trees with homomer root. Taken together these results indicate that quaternary structure errors in the PDB are unlikely to substantially change the patterns of Figure 4. On all panels boxplots display the median, 25-75% interquartile range (IQR), and 1.5 * interquartile range from the hinge (whiskers). Notches are defined as 1.58 * IQR / sqrt(n). Datapoints beyond the whiskers are shown as outliers. On all panels, p-values were calculated with Wilcoxon rank sum tests.

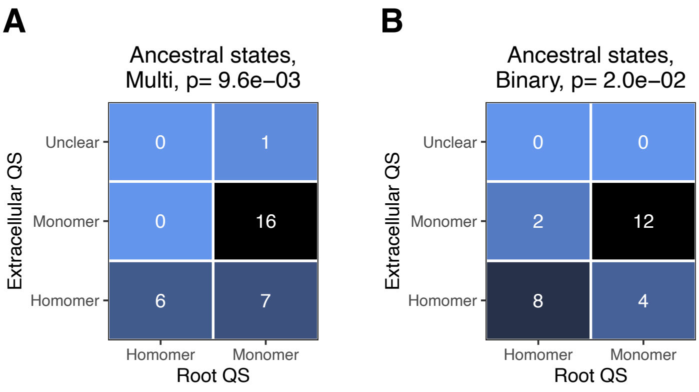

Supplementary Figure 8. Matrices with the quaternary structure (QS) of the root, and extracellular proteins in the orthogroups which do have extracellular proteins and their frequency is less than 50% of all proteins. Similarly to Figure 5, in the majority of cases, extracellular monomers are present in trees with monomer root, and the QS of extracellular proteins is largely the same as the root, indicating that most extracellular monomers did not lose their quaternary structure, but already evolved from monomers, while in trees with homomer root most of the time they remained homomers. P-values were obtained with the test of proportions.

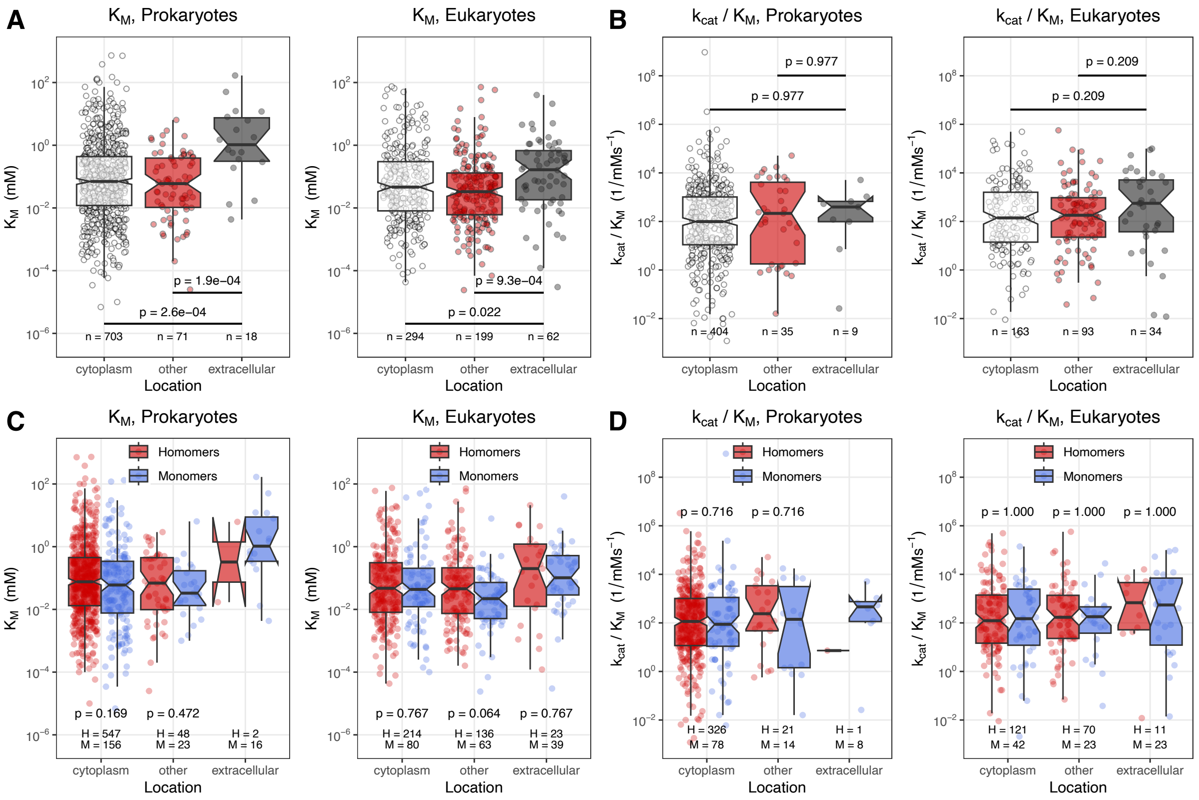

Supplementary Figure 9. Selection for higher catalytic efficiency is unlikely to cause the high frequency of monomers in extracellular enzymes. **A)** The K_M values of extracellular proteins are higher than in intracellular proteins, however in Eukaryotes significance drops when the catalytic constants of natural substrates are used (Supplementary Figure

8). **B)** There are no differences in k_{cat}/K_M between proteins in different cellular locations. **C and D)** There are no consistent differences in enzymatic efficiency between homomers and monomers, indicating that on evolutionary timescales, oligomerisation has no clear effect on catalytic efficiency. On all panels boxplots display the median, 25-75% interquartile range (IQR), and 1.5 * interquartile range from the hinge (whiskers). Notches are defined as 1.58 * IQR / sqrt(n). Datapoints beyond the whiskers are shown as outliers. P-values were calculated with Wilcoxon rank sum tests, and were corrected with the Benjamini-Hochberg method.

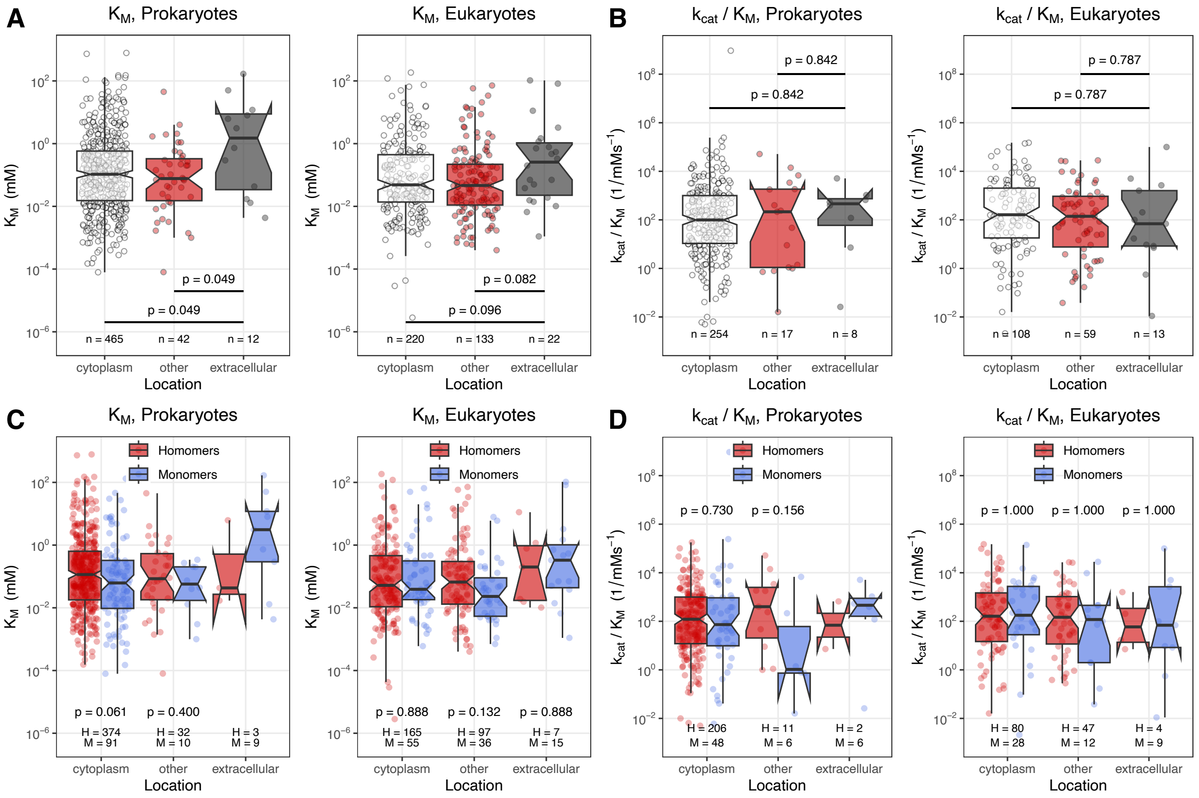

Supplementary Figure 10. Catalytic constants when only the K_M and k_{cat}/K_M values of natural substrates are included. The patterns are qualitatively similar to Supplementary Figure 7, suggesting that higher catalytic efficiency is not the reason for the high frequency of extracellular monomers. On all panels boxplots display the median, 25-75% interquartile range (IQR), and 1.5 * interquartile range from the hinge (whiskers). Notches are defined as 1.58 * IQR / sqrt(n). Datapoints beyond the whiskers are shown as outliers. P-values were calculated with Wilcoxon rank sum tests, and were corrected with the Benjamini-Hochberg method.

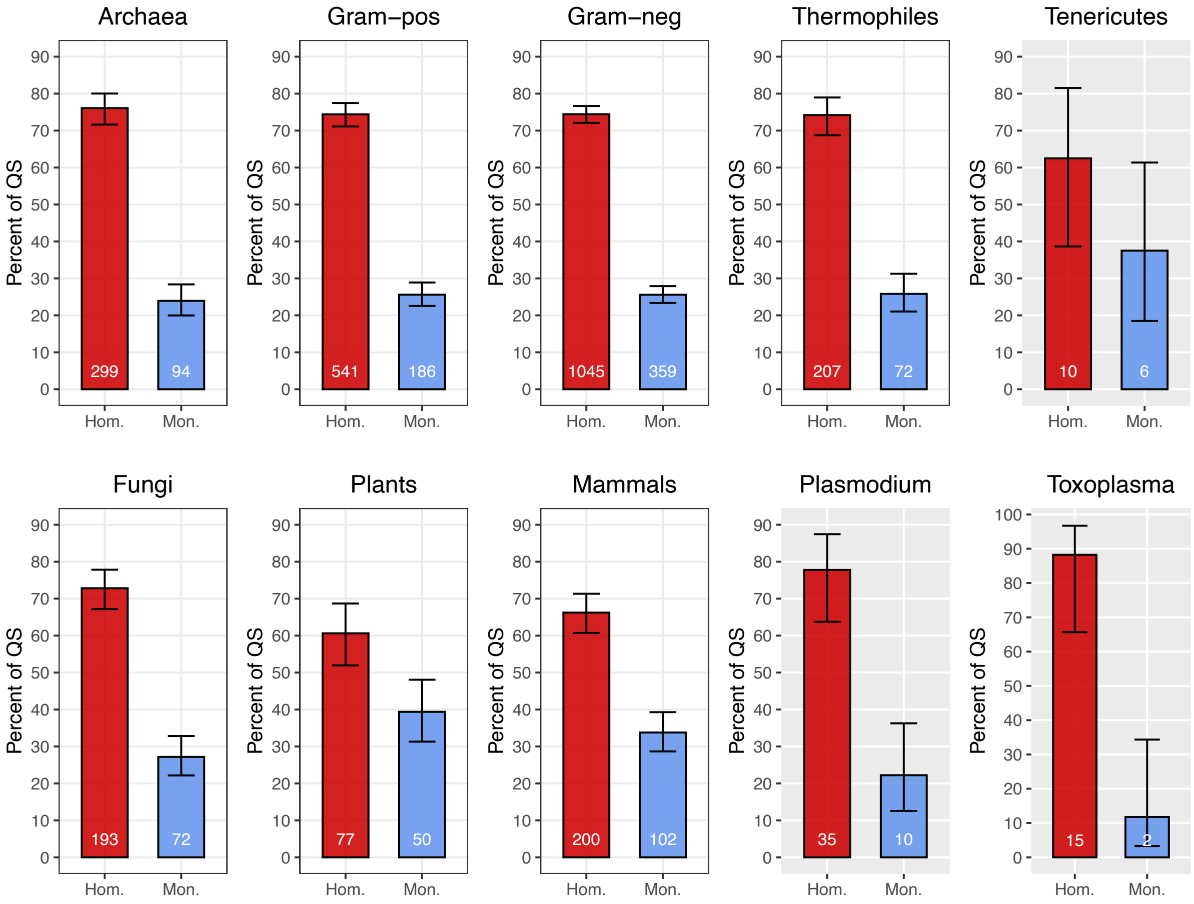

Supplementary Figure 11. Frequencies of homomers and monomers in cytoplasmic enzymes of different taxonomic groups in the PDB; panels with grey background indicate endoparasites. Bars indicate the fraction of quaternary structure in the set, whiskers represent 95% CI. Gram-positive and Gram-negative bacteria are based on PSORTDB; Thermophiles correspond to the combined set of proteins from the Gram-negative *Thermotoga maritima* and *Thermus thermophilus*. To remove redundancies, sequences in each group were clustered at 30% sequence similarity, and the cluster centroids were used. Error bars represent 95% confidence intervals. While the number and composition of proteins are variable for the different taxonomic groups, the frequency of homomers is comparable, ~75% in prokaryotes and 60-75% in the (non-Apicompexan) eukaryote groups. (The frequencies of homomers and monomers sum up to 100% in all cases)

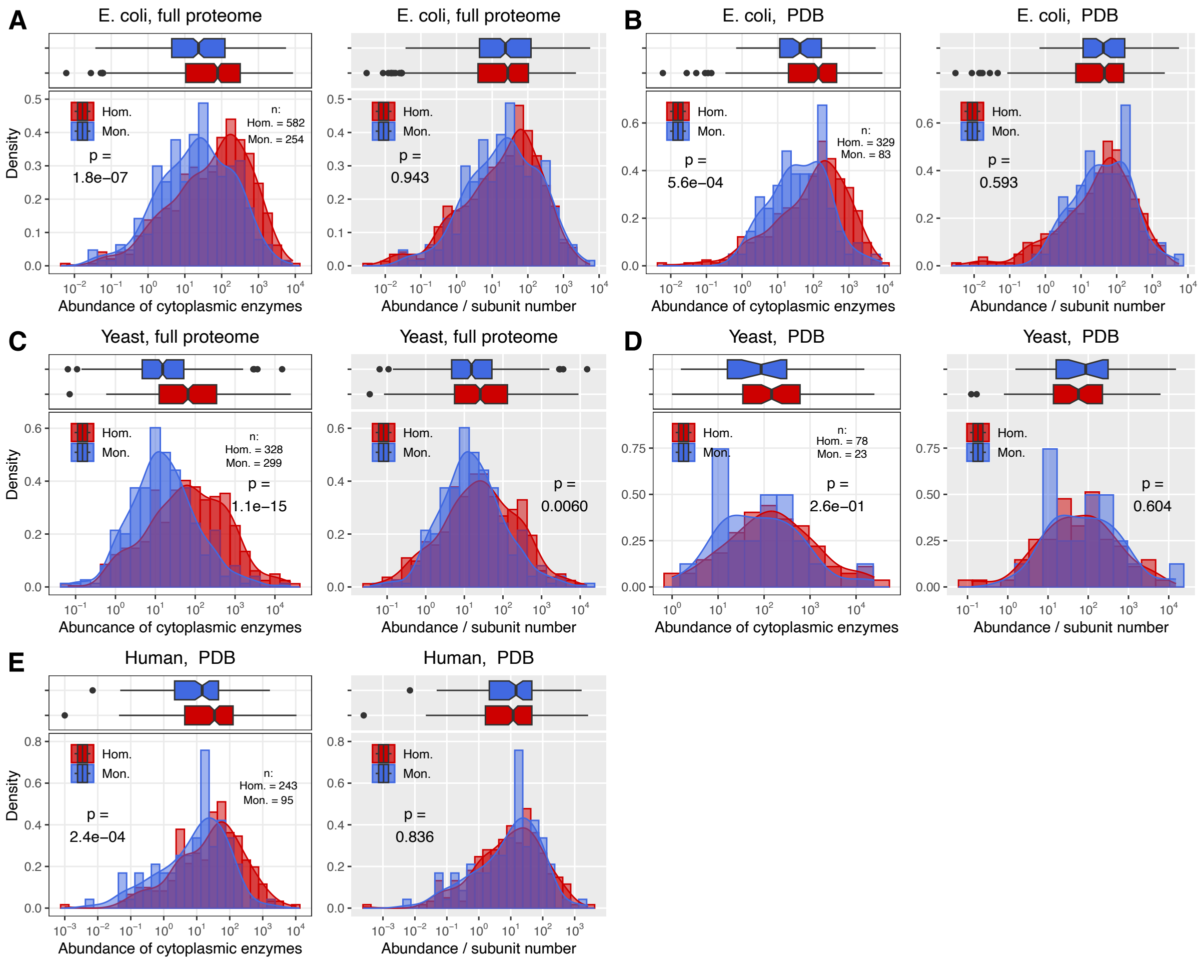

Supplementary Figure 12. The abundance of homomer and monomer enzymes in the cytoplasm. **A-B)** *E. coli*, **C-D)** Yeast, **E)** Human. The panels have a similar layout as on Figure 7. On all panels boxplots display the median, 25-75% interquartile range (IQR), and 1.5 * interquartile range from the hinge (whiskers). Notches are defined as 1.58 * IQR / sqrt(n). Datapoints beyond the whiskers are shown as outliers. On all panels P-values were calculated with Wilcoxon rank sum tests.

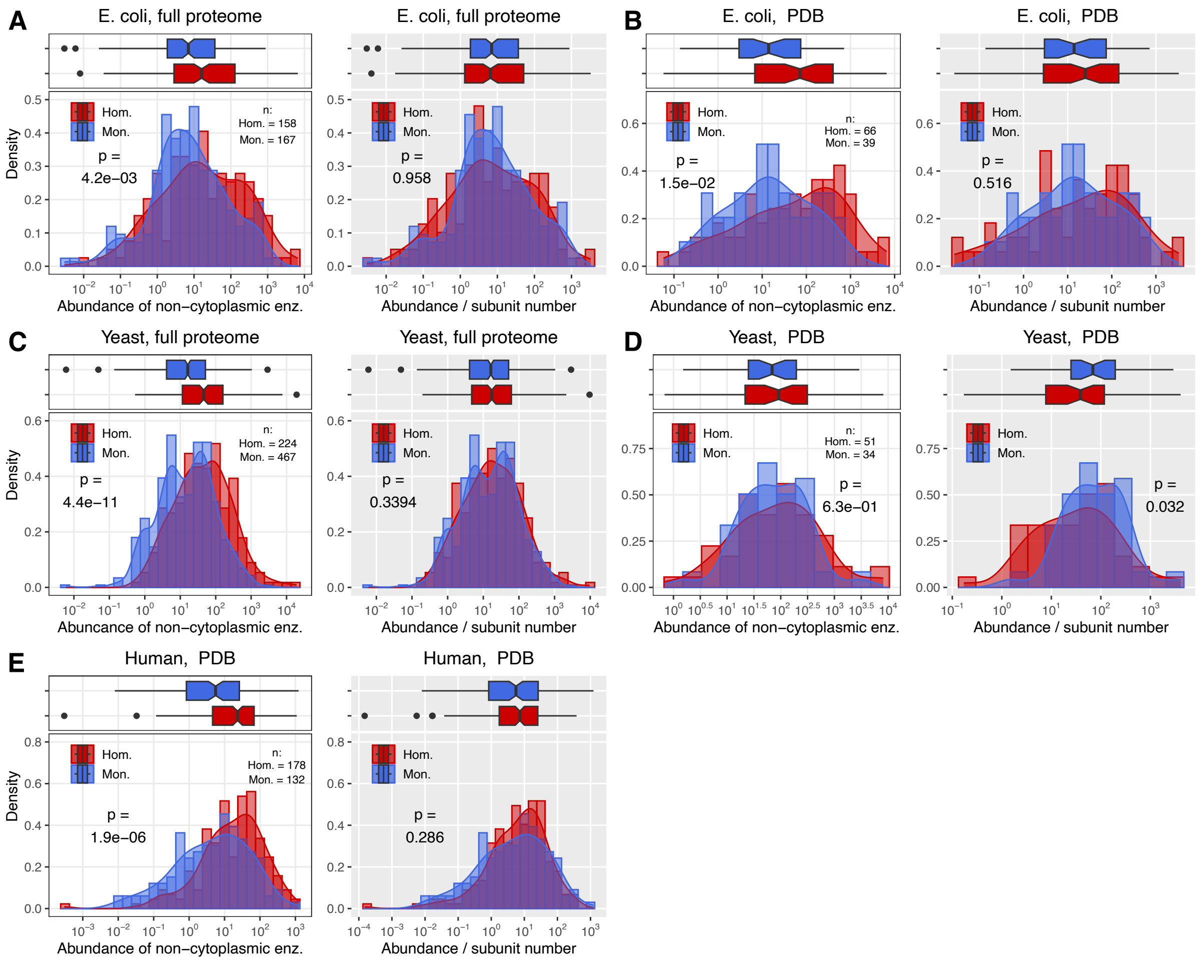

Supplementary Figure 13. The abundance of homomer and monomer enzymes in non-cytoplasmic proteins. **A-B)** *E. coli*, **C-D)** Yeast, **E)** Human. The panels have a similar layout as on Figure 7. On all panels boxplots display the median, 25-75% interquartile range (IQR), and 1.5 * interquartile range from the hinge (whiskers). Notches are defined as 1.58 * IQR / sqrt(n). Datapoints beyond the whiskers are shown as outliers. On all panels P-values were calculated with Wilcoxon rank sum tests.

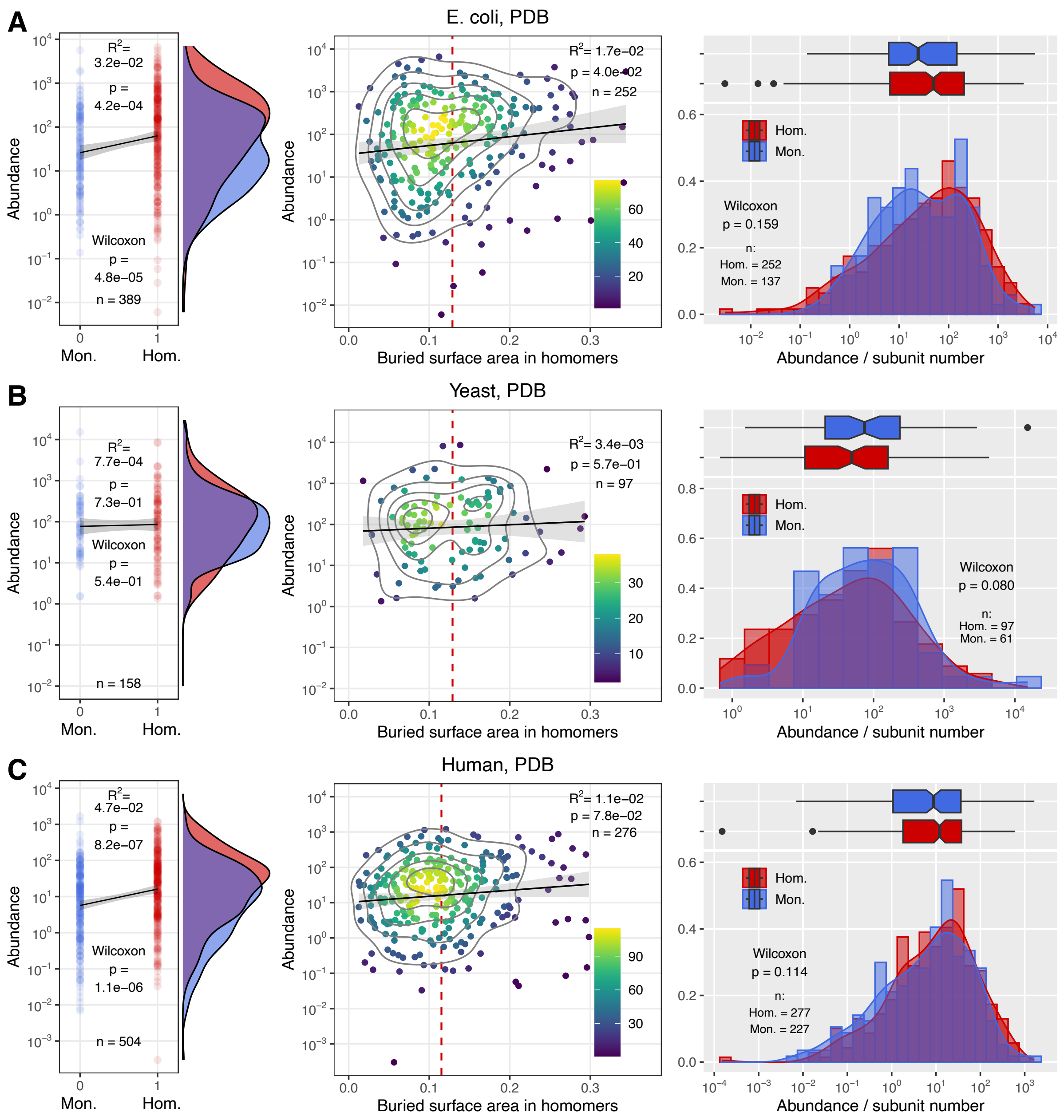

Supplementary Figure 14. Relationships between abundance, quaternary structure and interface size, using only homodimers. A) *E. coli*; **B)** Yeast; **C)** Human. Left panels indicate correlations between abundance and quaternary structure and the corresponding density plots of abundance, middle panels indicate correlations between the surface area buried in interfaces and abundance, right panels indicate abundances scaled with subunit number. The red vertical lines indicate the average buried surface area. Similarly to Figure 8, the correlation coefficients (R^2, their p-values were calculated with the Pearson correlation test) are generally low, nevertheless the correlations between quaternary structure and abundance explain a higher fraction of variance than the correlation between buried interface area and abundance, which is significant only in *E. coli*. Boxplots display the median, 25-75% interquartile range (IQR), and 1.5 * interquartile range from the hinge (whiskers). Notches are defined as 1.58 * IQR / sqrt(n). Datapoints beyond the whiskers are shown as outliers.

SUPPLEMENTARY NOTES

Data quality checks. To rule out the possibility that many of the monomers annotated as extracellular are in fact homomers, or are membrane proteins, we examined the overall surface hydrophobicity of homomers and monomers (Supplementary Figure 1), and in mammalian proteins the presence of transmembrane helices [1,2] (Supplementary Figure 2). We found that extracellular monomers have even lower surface hydrophobicity than cytoplasmic monomers (Supplementary Figure 1), which indicates the absence of a hydrophobic interface. Additionally, the distribution of the free energy (ΔG) of insertion of a TM helix into the endoplasmic reticulum membrane is somewhat higher in extracellular than in cytoplasmic enzymes (Supplementary Figure 2, p = 1.5e−02 for monomers, and p = 4.7e-03 for homomers), and is above 0, thus inserting them into membranes needs energy., This is in agreement with the distributions expected for cytoplasmic and secreted proteins [2], and largely rules out the possibility of being membrane-associated, at least for mammalian proteins.

Besides hydrophobicity and ΔG, we also examined what fraction of the homomers and monomers we use might be part of a larger heteromeric complex, as heteromers (i.e. complexes formed by different proteins) are highly incomplete and frequently missing in the PDB. For a few model organisms like yeast or *E. coli* essentially all of their stable macromolecular complexes are known [3], and allow for the quantification of this error in organisms where such information is absent. Using the data from Complex Portal, we found that in the case of yeast 5.3% of its homo- and monomer enzymes are present in a heteromer complex, while in the case of *E. coli* 3.8% (Supplementary Data 3). In the case of human enzymes the ratio is 2.2% (Supplementary Table 2), however this is certainly an underestimate. These numbers are well below the quaternary structure error rate of the PDB, which is estimated to be 10-15%.

Selection for high catalytic efficiency is unlikely to cause the high frequency of monomers in extracellular enzymes. Finally, we examined whether the high frequency of monomers in extracellular proteins is related to differences in catalytic rates between homomers and monomers. Previous studies indicate that the vast majority of enzymes are moderately efficient, and the catalytic rate of the typical enzyme is orders of magnitude below the rates of the most efficient enzymes [4], due to multiple factors, which include the location in the metabolic network (e.g. central vs. secondary metabolism), or the characteristics of ligands [4]. We hypothesised that the high frequency of monomers in the extracellular environment might also be, indirectly, the result of selection for higher efficiency, e.g. due to better hydration, or diffusion rates (Figure 2A) of monomers, especially in hydrolases. However, enzymatic efficiency can be measured directly by comparing catalytic constants, and thus we examined whether the Michaelis-Menten constants (K_M), and k_{cat}/K_M are different in the different cellular environments.

We used data from the BRENDA database [5], and for each protein present in BRENDA we used the lowest K_M, and highest k_{cat}/K_M values using all substrates (Supplementary Figure 9), and of the natural substrates, if they are known (Supplementary Figure 10). Similar to Figures 2 and 3, we used cluster centroids, to correct for redundancies. We found that K_M values are indeed higher in the extracellular environment (Supplementary Figure 7A), both in Prokaryotes and Eukaryotes, indicating that extracellular enzymes evolved to utilise higher concentrations of substrates than intracellular ones. However, k_{cat}/K_M is not different (Supplementary Figure 7B), indicating that the catalytic rate itself is not affected by cellular location. Moreover, the comparison

of monomers and homomers in each group does not indicate that oligomerisation significantly affects K_M, or k_{cat}/K_M, neither in the extracellular space nor in the cytoplasm (Supplementary Figure 7C and 7D). These patterns are qualitatively similar when only the K_M, and k_{cat}/K_M values of natural substrates are used (Supplementary Figure 8), or only hydrolases (EC=3) were included (not shown), the difference being that in these cases extracellular K_M values are not significantly higher in Eukaryotes. Taken together, even though extracellularity does seem to affect K_M, these results indicate that the high frequency of monomers among extracellular enzymes is unlikely to be the consequence of selection for high catalytic efficiency.

SUPPLEMENTARY REFERENCES

1. Hessa, T. *et al.* Recognition of transmembrane helices by the endoplasmic reticulum translocon. *Nature* **433**, 377–381 (2005).
2. Hessa, T. *et al.* Molecular code for transmembrane-helix recognition by the Sec61 translocon. *Nature* **450**, 1026–1030 (2007).
3. Meldal, B. H. M. *et al.* Complex Portal 2022: new curation frontiers. *Nucleic Acids Res.* **50**, D578–D586 (2022).
4. Bar-Even, A. *et al.* The Moderately Efficient Enzyme: Evolutionary and Physicochemical Trends Shaping Enzyme Parameters. *Biochemistry* **50**, 4402–4410 (2011).
5. Chang, A. *et al.* BRENDA, the ELIXIR core data resource in 2021: new developments and updates. *Nucleic Acids Res.* **49**, D498–D508 (2021).
