# Supplementary material for: Cellular location shapes quaternary structure of enzymes
Source: Nat Commun. 2024 Oct 1;15:8505. doi: 10.1038/s41467-024-52662-2 (PMC11445431; doi:10.1038/s41467-024-52662-2)
Supplement: Supplementary file 3 — Description of Additional Supplementary Files [file 41467_2024_52662_MOESM3_ESM.pdf]

Description of Additional Supplementary Files

File Name: Supplementary Data 1
Description: The list of the enzymes used in the study.

File Name: Supplementary Data 2
Description: Gene Ontology analysis results.

File Name: Supplementary Data 3
Description: The number of clusters in the PDB for each of the three species, with their quaternary structure (H or M) and cellular location, and the numbers that are also present in the Complex Portal heteromers.
