# Supplementary material for: Cellular location shapes quaternary structure of enzymes
Source: Nat Commun. 2024 Oct 1;15:8505. doi: 10.1038/s41467-024-52662-2 (PMC11445431; doi:10.1038/s41467-024-52662-2)
Supplement: Supplementary file 5 — Supplementary Data 2 [file 41467_2024_52662_MOESM5_ESM.zip › GO/Prok_P/output.P.txt.html]

 GeneMerge Output - output.P.txt

### GeneMerge v1.4

### Castillo-Davis, C.I. 2015. GeneMerge v1.4 - post-genomic data analysis

Output File Name: output.P.txt   
Gene Association File: terms.txt   
Description File: descriptions.txt   
Population File: prot\_all.txt   
Study File: prot\_test.txt  
Custom FDR: 0.5%  

|  |  |  |  |  |  |  |  |  |  |  |  |
| --- | --- | --- | --- | --- | --- | --- | --- | --- | --- | --- | --- |
| **GMRG Term** | **Pop Frequency** | **Pop Fraction** | **Study Fraction** | ***P*-value** | **Bon. Corr. *P*-value** | **10% FDR** | **5% FDR** | **1% FDR** | **0.5% FDR** | **Description** | **Contributing genes** |
| GO:0000272 | 0.0292501616031028 | 181/6188 | 48/182 | 4.36139615283791e-34 | 1.41745374967232e-31 | T | T | T | T | polysaccharide catabolic process | A0A160EBC2\_ACHDE | A0A4P8ESF9\_BACSP | A1TSQ3\_ACIAC | A3DJ82\_ACET2 | A8FDC4\_BACP2 | AMT6\_BACS7 | AMYB\_BACCE | B9MMA5\_CALBD | C7ED31\_ACET1 | D0VP31\_9BACI | E4Q2A4\_CALOW | ENG1\_ACET2 | G8LZE0\_ACECE | GUNA\_ACET2 | GUNC\_ACETH | GUNF\_RUMCH | GUNG\_RUMCH | GUNS\_ACETH | GUN\_ECOLI | IABF\_STRAW | K0IUV6\_9ENTR | L0EGW1\_THECK | LCHMO\_ENTFA | O30700\_9BACI | PLYL\_DICD3 | PLY\_BACSU | PMEA\_DICD3 | PTLY\_THEMA | Q09LY9\_GEOSE | Q17TM8\_ALKHA | Q59962\_9ACTN | Q65GB9\_BACLD | Q65JI7\_BACLD | Q65JI8\_BACLD | Q6RSN8\_ACETH | Q7SID8\_BACIU | Q7X4S4\_BACLI | Q88JL2\_PSEPK | Q8P6Z9\_XANCP | Q9AJF8\_ACETH | Q9AJM4\_9BACI | Q9AJS0\_ALIAC | Q9EYQ2\_9FIRM | Q9RC94\_9BACI | W5JXE0\_BACSP | XYN1\_GEOSE | XYNA\_HALH5 | XYNC\_BACSU |
 GO:0009057 | 0.0389463477698772 | 241/6188 | 53/182 | 2.95789008190865e-33 | 9.61314276620313e-31 | T | T | T | T | macromolecule catabolic process | A0A160EBC2\_ACHDE | A0A4P8ESF9\_BACSP | A0R5R2\_MYCS2 | A1TSQ3\_ACIAC | A3DJ82\_ACET2 | A8FDC4\_BACP2 | AMT6\_BACS7 | AMYB\_BACCE | B9MMA5\_CALBD | C7ED31\_ACET1 | D0VP31\_9BACI | DRNE\_VIBCH | E4Q2A4\_CALOW | ENG1\_ACET2 | G8LZE0\_ACECE | GUNA\_ACET2 | GUNC\_ACETH | GUNF\_RUMCH | GUNG\_RUMCH | GUNS\_ACETH | GUN\_ECOLI | IABF\_STRAW | K0IUV6\_9ENTR | L0EGW1\_THECK | LCHMO\_ENTFA | O06496\_CLOPF | O30700\_9BACI | O83009\_SERMA | PLYL\_DICD3 | PLY\_BACSU | PMEA\_DICD3 | PTLY\_THEMA | Q09LY9\_GEOSE | Q17TM8\_ALKHA | Q59962\_9ACTN | Q65GB9\_BACLD | Q65JI7\_BACLD | Q65JI8\_BACLD | Q6RSN8\_ACETH | Q7SID8\_BACIU | Q7X4S4\_BACLI | Q88JL2\_PSEPK | Q8P6Z9\_XANCP | Q9AJF8\_ACETH | Q9AJM4\_9BACI | Q9AJS0\_ALIAC | Q9EYQ2\_9FIRM | Q9RC94\_9BACI | W5JXE0\_BACSP | XYN1\_GEOSE | XYNA\_HALH5 | XYNC\_BACSU | YKFC\_BACC1 | GO:0005976 | 0.047349709114415 | 293/6188 | 50/182 | 8.36124011300599e-26 | 2.71740303672695e-23 | T | T | T | T | polysaccharide metabolic process | A0A160EBC2\_ACHDE | A0A4P8ESF9\_BACSP | A1TSQ3\_ACIAC | A3DJ82\_ACET2 | A8FDC4\_BACP2 | AMT6\_BACS7 | AMYB\_BACCE | B9MMA5\_CALBD | C7ED31\_ACET1 | D0VP31\_9BACI | E4Q2A4\_CALOW | ENG1\_ACET2 | G8LZE0\_ACECE | GUNA\_ACET2 | GUNC\_ACETH | GUNF\_RUMCH | GUNG\_RUMCH | GUNS\_ACETH | GUN\_ECOLI | IABF\_STRAW | K0IUV6\_9ENTR | L0EGW1\_THECK | LCHMO\_ENTFA | O30700\_9BACI | PLYL\_DICD3 | PLY\_BACSU | PMEA\_DICD3 | PTLY\_THEMA | Q09LY9\_GEOSE | Q17TM8\_ALKHA | Q59962\_9ACTN | Q65GB9\_BACLD | Q65JI7\_BACLD | Q65JI8\_BACLD | Q6RSN8\_ACETH | Q7SID8\_BACIU | Q7X4S4\_BACLI | Q88JL2\_PSEPK | Q8P6Z9\_XANCP | Q9AJF8\_ACETH | Q9AJM4\_9BACI | Q9AJS0\_ALIAC | Q9EYQ2\_9FIRM | Q9RC94\_9BACI | Q9RHW0\_BACSP | W5JXE0\_BACSP | XANLY\_BACGL | XYN1\_GEOSE | XYNA\_HALH5 | XYNC\_BACSU | GO:0005975 | 0.201357466063348 | 1246/6188 | 87/182 | 1.64940142011754e-17 | 5.36055461538202e-15 | T | T | T | T | carbohydrate metabolic process | A0A067XG64\_PARTM | A0A0B7LH44\_STREE | A0A0H2UQE4\_STRPN | A0A160EBC2\_ACHDE | A0A182DW00\_STREE | A0A243G6Q6\_BACTU | A0A4P8ESF9\_BACSP | A0A6L7H1P2\_BACAN | A1TSQ3\_ACIAC | A3DJ82\_ACET2 | A8FDC4\_BACP2 | AMT6\_BACS7 | AMYB\_BACCE | AMYM\_GEOSE | AMYS\_NEIPO | AMY\_BACAM | AMY\_GEOSE | B9MMA5\_CALBD | C7ED31\_ACET1 | CDGT\_BACS0 | CDGT\_GEOSE | CHIS\_BACSU | CHIS\_STRSN | D0VP31\_9BACI | D0VV09\_BACCE | E4Q2A4\_CALOW | EBA1\_ELIME | EBA3\_ELIME | ENG1\_ACET2 | G0L2Y1\_ZOBGA | G2NRC4\_STREK | G8LZE0\_ACECE | GUB\_BACLI | GUB\_BACSU | GUNA\_ACET2 | GUNC\_ACETH | GUNF\_RUMCH | GUNG\_RUMCH | GUNS\_ACETH | GUN\_ECOLI | HYSA\_STRA3 | I3P686\_BACLI | IABF\_STRAW | K0IUV6\_9ENTR | L0EGW1\_THECK | LCHMO\_ENTFA | NANB\_STRPN | NANH\_VIBCH | O30700\_9BACI | O52754\_RHOMR | O82839\_BACSP | P84141\_PAEAU | PGLR2\_PECPM | PLYL\_DICD3 | PLY\_BACSU | PMEA\_DICD3 | PTLY\_THEMA | Q09LY9\_GEOSE | Q17TM8\_ALKHA | Q54276\_SERMA | Q59962\_9ACTN | Q5DZ44\_ALIF1 | Q65GB9\_BACLD | Q65JI7\_BACLD | Q65JI8\_BACLD | Q6RSN8\_ACETH | Q7LYT7\_PYRWO | Q7SID8\_BACIU | Q7X4S4\_BACLI | Q82L26\_STRAW | Q88JL2\_PSEPK | Q8P6Z9\_XANCP | Q93I48\_9BACI | Q9AJF8\_ACETH | Q9AJM4\_9BACI | Q9AJS0\_ALIAC | Q9EYQ2\_9FIRM | Q9KWY6\_GEOSE | Q9RC94\_9BACI | Q9REI6\_ARTSP | Q9RHW0\_BACSP | RHA78\_STRAW | W5JXE0\_BACSP | XANLY\_BACGL | XYN1\_GEOSE | XYNA\_HALH5 | XYNC\_BACSU | GO:0043170 | 0.210407239819005 | 1302/6188 | 87/182 | 2.96133709901233e-16 | 9.62434557179009e-14 | T | T | T | T | macromolecule metabolic process | A0A0H2WX20\_STAAC | A0A0M3KKW7\_STRPY | A0A160EBC2\_ACHDE | A0A4P8ESF9\_BACSP | A0A4V8H037\_9RHOB | A0R5R2\_MYCS2 | A1TSQ3\_ACIAC | A3DJ82\_ACET2 | A8FDC4\_BACP2 | AMT6\_BACS7 | AMYB\_BACCE | B9MMA5\_CALBD | BSAP\_BACSU | BXX\_CLOBO | C7ED31\_ACET1 | CBPM\_STRAL | D0VP31\_9BACI | D0VXY8\_STAPS | D8H130\_BACAI | DACC\_BACSU | DAC\_STRSR | DRNE\_VIBCH | E4Q2A4\_CALOW | ENG1\_ACET2 | ETA\_STAAU | ETB\_STAAU | FLS\_FERPE | G8LZE0\_ACECE | GSEA\_STAES | GUNA\_ACET2 | GUNC\_ACETH | GUNF\_RUMCH | GUNG\_RUMCH | GUNS\_ACETH | GUN\_ECOLI | IABF\_STRAW | K0IUV6\_9ENTR | L0EGW1\_THECK | L0RUV7\_STAAU | LCHMO\_ENTFA | LYTM\_STAA8 | O06496\_CLOPF | O30700\_9BACI | O68424\_BACFG | O69771\_9PSED | O83009\_SERMA | O86049\_BACFG | PEPX\_LACHE | PLYL\_DICD3 | PLY\_BACSU | PMEA\_DICD3 | PRZN\_SERME | PTLY\_THEMA | Q09LY9\_GEOSE | Q17TM8\_ALKHA | Q59962\_9ACTN | Q5MJ80\_SERPR | Q65GB9\_BACLD | Q65JI7\_BACLD | Q65JI8\_BACLD | Q6RSN8\_ACETH | Q7SID8\_BACIU | Q7X4S4\_BACLI | Q88JL2\_PSEPK | Q8P6Z9\_XANCP | Q9AJF8\_ACETH | Q9AJM4\_9BACI | Q9AJS0\_ALIAC | Q9EYQ2\_9FIRM | Q9RC94\_9BACI | Q9RHW0\_BACSP | RIPB\_MYCTU | SFAS2\_STRFR | SPLA\_STAA8 | SPLB\_STAA8 | SPLC\_STAA8 | SPLD\_STAA8 | SPLE\_STAA8 | SSL1\_STAA8 | SUBD\_BACLI | V5IRV7\_THETH | W5JXE0\_BACSP | XANLY\_BACGL | XYN1\_GEOSE | XYNA\_HALH5 | XYNC\_BACSU | YKFC\_BACC1 | GO:0006508 | 0.0355526826115061 | 220/6188 | 33/182 | 2.90755162558979e-15 | 9.44954278316681e-13 | T | T | T | T | proteolysis | A0A0H2WX20\_STAAC | A0A0M3KKW7\_STRPY | A0A4V8H037\_9RHOB | BSAP\_BACSU | BXX\_CLOBO | CBPM\_STRAL | D0VXY8\_STAPS | D8H130\_BACAI | DACC\_BACSU | DAC\_STRSR | ETA\_STAAU | ETB\_STAAU | FLS\_FERPE | GSEA\_STAES | L0RUV7\_STAAU | LYTM\_STAA8 | O68424\_BACFG | O69771\_9PSED | O86049\_BACFG | PEPX\_LACHE | PRZN\_SERME | Q5MJ80\_SERPR | RIPB\_MYCTU | SFAS2\_STRFR | SPLA\_STAA8 | SPLB\_STAA8 | SPLC\_STAA8 | SPLD\_STAA8 | SPLE\_STAA8 | SSL1\_STAA8 | SUBD\_BACLI | V5IRV7\_THETH | YKFC\_BACC1 | GO:0016052 | 0.0832255979314803 | 515/6188 | 49/182 | 3.25427503159035e-14 | 1.05763938526686e-11 | T | T | T | T | carbohydrate catabolic process | A0A160EBC2\_ACHDE | A0A4P8ESF9\_BACSP | A1TSQ3\_ACIAC | A3DJ82\_ACET2 | A8FDC4\_BACP2 | AMT6\_BACS7 | AMYB\_BACCE | B9MMA5\_CALBD | C7ED31\_ACET1 | D0VP31\_9BACI | E4Q2A4\_CALOW | ENG1\_ACET2 | G8LZE0\_ACECE | GUNA\_ACET2 | GUNC\_ACETH | GUNF\_RUMCH | GUNG\_RUMCH | GUNS\_ACETH | GUN\_ECOLI | IABF\_STRAW | K0IUV6\_9ENTR | L0EGW1\_THECK | LCHMO\_ENTFA | NANH\_VIBCH | O30700\_9BACI | PLYL\_DICD3 | PLY\_BACSU | PMEA\_DICD3 | PTLY\_THEMA | Q09LY9\_GEOSE | Q17TM8\_ALKHA | Q59962\_9ACTN | Q65GB9\_BACLD | Q65JI7\_BACLD | Q65JI8\_BACLD | Q6RSN8\_ACETH | Q7SID8\_BACIU | Q7X4S4\_BACLI | Q88JL2\_PSEPK | Q8P6Z9\_XANCP | Q9AJF8\_ACETH | Q9AJM4\_9BACI | Q9AJS0\_ALIAC | Q9EYQ2\_9FIRM | Q9RC94\_9BACI | W5JXE0\_BACSP | XYN1\_GEOSE | XYNA\_HALH5 | XYNC\_BACSU | GO:0045493 | 0.0072721396250808 | 45/6188 | 15/182 | 9.55914985643167e-13 | 3.10672370334029e-10 | T | T | T | T | xylan catabolic process | A0A4P8ESF9\_BACSP | B9MMA5\_CALBD | E4Q2A4\_CALOW | G8LZE0\_ACECE | L0EGW1\_THECK | O30700\_9BACI | Q09LY9\_GEOSE | Q17TM8\_ALKHA | Q59962\_9ACTN | Q7SID8\_BACIU | Q9RC94\_9BACI | W5JXE0\_BACSP | XYN1\_GEOSE | XYNA\_HALH5 | XYNC\_BACSU | GO:0045491 | 0.00743374272786037 | 46/6188 | 15/182 | 1.38243003670697e-12 | 4.49289761929766e-10 | T | T | T | T | xylan metabolic process | A0A4P8ESF9\_BACSP | B9MMA5\_CALBD | E4Q2A4\_CALOW | G8LZE0\_ACECE | L0EGW1\_THECK | O30700\_9BACI | Q09LY9\_GEOSE | Q17TM8\_ALKHA | Q59962\_9ACTN | Q7SID8\_BACIU | Q9RC94\_9BACI | W5JXE0\_BACSP | XYN1\_GEOSE | XYNA\_HALH5 | XYNC\_BACSU | GO:0016998 | 0.0105042016806723 | 65/6188 | 17/182 | 2.38176523532301e-12 | 7.74073701479979e-10 | T | T | T | T | cell wall macromolecule catabolic process | A0A4P8ESF9\_BACSP | B9MMA5\_CALBD | E4Q2A4\_CALOW | G8LZE0\_ACECE | L0EGW1\_THECK | O06496\_CLOPF | O30700\_9BACI | Q09LY9\_GEOSE | Q17TM8\_ALKHA | Q59962\_9ACTN | Q7SID8\_BACIU | Q9RC94\_9BACI | W5JXE0\_BACSP | XYN1\_GEOSE | XYNA\_HALH5 | XYNC\_BACSU | YKFC\_BACC1 | GO:0044036 | 0.010989010989011 | 68/6188 | 17/182 | 5.30707420722972e-12 | 1.72479911734966e-09 | T | T | T | T | cell wall macromolecule metabolic process | A0A4P8ESF9\_BACSP | B9MMA5\_CALBD | E4Q2A4\_CALOW | G8LZE0\_ACECE | L0EGW1\_THECK | O06496\_CLOPF | O30700\_9BACI | Q09LY9\_GEOSE | Q17TM8\_ALKHA | Q59962\_9ACTN | Q7SID8\_BACIU | Q9RC94\_9BACI | W5JXE0\_BACSP | XYN1\_GEOSE | XYNA\_HALH5 | XYNC\_BACSU | YKFC\_BACC1 | GO:0044347 | 0.00840336134453781 | 52/6188 | 15/182 | 1.03744226689644e-11 | 3.37168736741343e-09 | T | T | T | T | cell wall polysaccharide catabolic process | A0A4P8ESF9\_BACSP | B9MMA5\_CALBD | E4Q2A4\_CALOW | G8LZE0\_ACECE | L0EGW1\_THECK | O30700\_9BACI | Q09LY9\_GEOSE | Q17TM8\_ALKHA | Q59962\_9ACTN | Q7SID8\_BACIU | Q9RC94\_9BACI | W5JXE0\_BACSP | XYN1\_GEOSE | XYNA\_HALH5 | XYNC\_BACSU | GO:2000895 | 0.00840336134453781 | 52/6188 | 15/182 | 1.03744226689644e-11 | 3.37168736741343e-09 | T | T | T | T | hemicellulose catabolic process | A0A4P8ESF9\_BACSP | B9MMA5\_CALBD | E4Q2A4\_CALOW | G8LZE0\_ACECE | L0EGW1\_THECK | O30700\_9BACI | Q09LY9\_GEOSE | Q17TM8\_ALKHA | Q59962\_9ACTN | Q7SID8\_BACIU | Q9RC94\_9BACI | W5JXE0\_BACSP | XYN1\_GEOSE | XYNA\_HALH5 | XYNC\_BACSU | GO:0010410 | 0.00856496444731739 | 53/6188 | 15/182 | 1.41020058367284e-11 | 4.58315189693672e-09 | T | T | T | T | hemicellulose metabolic process | A0A4P8ESF9\_BACSP | B9MMA5\_CALBD | E4Q2A4\_CALOW | G8LZE0\_ACECE | L0EGW1\_THECK | O30700\_9BACI | Q09LY9\_GEOSE | Q17TM8\_ALKHA | Q59962\_9ACTN | Q7SID8\_BACIU | Q9RC94\_9BACI | W5JXE0\_BACSP | XYN1\_GEOSE | XYNA\_HALH5 | XYNC\_BACSU | GO:0010383 | 0.00888817065287653 | 55/6188 | 15/182 | 2.55012128934096e-11 | 8.28789419035812e-09 | T | T | T | T | cell wall polysaccharide metabolic process | A0A4P8ESF9\_BACSP | B9MMA5\_CALBD | E4Q2A4\_CALOW | G8LZE0\_ACECE | L0EGW1\_THECK | O30700\_9BACI | Q09LY9\_GEOSE | Q17TM8\_ALKHA | Q59962\_9ACTN | Q7SID8\_BACIU | Q9RC94\_9BACI | W5JXE0\_BACSP | XYN1\_GEOSE | XYNA\_HALH5 | XYNC\_BACSU | GO:1901575 | 0.217356173238526 | 1345/6188 | 78/182 | 7.31861684896475e-11 | 2.37855047591354e-08 | T | T | T | T | organic substance catabolic process | A0A067XG64\_PARTM | A0A160EBC2\_ACHDE | A0A4P8ESF9\_BACSP | A0R5R2\_MYCS2 | A1TSQ3\_ACIAC | A3DJ82\_ACET2 | A5N1B8\_CLOK5 | A8FDC4\_BACP2 | AMT6\_BACS7 | AMYB\_BACCE | B9MMA5\_CALBD | BLAC\_BACSU | BLAC\_STRAL | C7ED31\_ACET1 | CHOD\_STRS0 | D0VP31\_9BACI | D2ARB3\_STRRD | D5DH82\_PRIM3 | D9TA79\_MICAI | DRNE\_VIBCH | E4Q2A4\_CALOW | ENG1\_ACET2 | ESTA\_BACSU | G8LZE0\_ACECE | GUNA\_ACET2 | GUNC\_ACETH | GUNF\_RUMCH | GUNG\_RUMCH | GUNS\_ACETH | GUN\_ECOLI | H0B8D4\_9ACTN | HYSA\_STRA3 | IABF\_STRAW | K0IUV6\_9ENTR | L0EGW1\_THECK | LCHMO\_ENTFA | LIP\_BACSP | LIP\_PSEAE | LYTM\_STAA8 | NANH\_VIBCH | O06496\_CLOPF | O30700\_9BACI | O83009\_SERMA | P94458\_BACLI | PLC\_BACCE | PLC\_BACTU | PLC\_LISMO | PLC\_STAAE | PLD\_STRAT | PLYL\_DICD3 | PLY\_BACSU | PMEA\_DICD3 | PTLY\_THEMA | Q09LY9\_GEOSE | Q17TM8\_ALKHA | Q59962\_9ACTN | Q65GB9\_BACLD | Q65JI7\_BACLD | Q65JI8\_BACLD | Q6RSN8\_ACETH | Q79DR3\_ECOLX | Q7SID8\_BACIU | Q7X4S4\_BACLI | Q81DA0\_BACCR | Q88JL2\_PSEPK | Q8P6Z9\_XANCP | Q9AJF8\_ACETH | Q9AJM4\_9BACI | Q9AJS0\_ALIAC | Q9EYQ2\_9FIRM | Q9JZ43\_NEIMB | Q9RC94\_9BACI | W5JXE0\_BACSP | W8FKE7\_BACPU | XYN1\_GEOSE | XYNA\_HALH5 | XYNC\_BACSU | YKFC\_BACC1 | GO:0030245 | 0.00904977375565611 | 56/6188 | 14/182 | 4.33732285564369e-10 | 1.4096299280842e-07 | T | T | T | T | cellulose catabolic process | A3DJ82\_ACET2 | A8FDC4\_BACP2 | C7ED31\_ACET1 | GUNA\_ACET2 | GUNC\_ACETH | GUNF\_RUMCH | GUNG\_RUMCH | GUNS\_ACETH | GUN\_ECOLI | Q65JI7\_BACLD | Q65JI8\_BACLD | Q6RSN8\_ACETH | Q9AJF8\_ACETH | Q9AJS0\_ALIAC | GO:0051273 | 0.00904977375565611 | 56/6188 | 14/182 | 4.33732285564369e-10 | 1.4096299280842e-07 | T | T | T | T | beta-glucan metabolic process | A3DJ82\_ACET2 | A8FDC4\_BACP2 | C7ED31\_ACET1 | GUNA\_ACET2 | GUNC\_ACETH | GUNF\_RUMCH | GUNG\_RUMCH | GUNS\_ACETH | GUN\_ECOLI | Q65JI7\_BACLD | Q65JI8\_BACLD | Q6RSN8\_ACETH | Q9AJF8\_ACETH | Q9AJS0\_ALIAC | GO:0030243 | 0.00904977375565611 | 56/6188 | 14/182 | 4.33732285564369e-10 | 1.4096299280842e-07 | T | T | T | T | cellulose metabolic process | A3DJ82\_ACET2 | A8FDC4\_BACP2 | C7ED31\_ACET1 | GUNA\_ACET2 | GUNC\_ACETH | GUNF\_RUMCH | GUNG\_RUMCH | GUNS\_ACETH | GUN\_ECOLI | Q65JI7\_BACLD | Q65JI8\_BACLD | Q6RSN8\_ACETH | Q9AJF8\_ACETH | Q9AJS0\_ALIAC | GO:0051275 | 0.00904977375565611 | 56/6188 | 14/182 | 4.33732285564369e-10 | 1.4096299280842e-07 | T | T | T | T | beta-glucan catabolic process | A3DJ82\_ACET2 | A8FDC4\_BACP2 | C7ED31\_ACET1 | GUNA\_ACET2 | GUNC\_ACETH | GUNF\_RUMCH | GUNG\_RUMCH | GUNS\_ACETH | GUN\_ECOLI | Q65JI7\_BACLD | Q65JI8\_BACLD | Q6RSN8\_ACETH | Q9AJF8\_ACETH | Q9AJS0\_ALIAC | GO:0009758 | 0.00145442792501616 | 9/6188 | 7/182 | 5.81996031246296e-10 | 1.89148710155046e-07 | T | T | T | T | carbohydrate utilization | A0A0M3KKU6\_ERWAE | B2IF78\_BEII9 | B2VCC3\_ERWT9 | D5DC07\_PRIM3 | D8J9C2\_HALJB | SACB\_BACSU | SACB\_GLUDI | GO:0009056 | 0.242889463477699 | 1503/6188 | 81/182 | 1.10765110653668e-09 | 3.59986609624421e-07 | T | T | T | T | catabolic process | A0A067XG64\_PARTM | A0A160EBC2\_ACHDE | A0A4P8ESF9\_BACSP | A0A4V8H037\_9RHOB | A0R5R2\_MYCS2 | A1TSQ3\_ACIAC | A3DJ82\_ACET2 | A5N1B8\_CLOK5 | A8FDC4\_BACP2 | AMT6\_BACS7 | AMYB\_BACCE | B4EPS2\_BURCJ | B9MMA5\_CALBD | BLAC\_BACSU | BLAC\_STRAL | C7ED31\_ACET1 | CHOD\_STRS0 | D0VP31\_9BACI | D2ARB3\_STRRD | D5DH82\_PRIM3 | D9TA79\_MICAI | DRNE\_VIBCH | E4Q2A4\_CALOW | ENG1\_ACET2 | ESTA\_BACSU | G8LZE0\_ACECE | GUNA\_ACET2 | GUNC\_ACETH | GUNF\_RUMCH | GUNG\_RUMCH | GUNS\_ACETH | GUN\_ECOLI | H0B8D4\_9ACTN | HYSA\_STRA3 | IABF\_STRAW | K0IUV6\_9ENTR | L0EGW1\_THECK | LCHMO\_ENTFA | LIP\_BACSP | LIP\_PSEAE | LYTM\_STAA8 | NANH\_VIBCH | O06496\_CLOPF | O30700\_9BACI | O83009\_SERMA | P94458\_BACLI | PLC\_BACCE | PLC\_BACTU | PLC\_LISMO | PLC\_STAAE | PLD\_STRAT | PLYL\_DICD3 | PLY\_BACSU | PMEA\_DICD3 | PTLY\_THEMA | Q09LY9\_GEOSE | Q17TM8\_ALKHA | Q59962\_9ACTN | Q65GB9\_BACLD | Q65JI7\_BACLD | Q65JI8\_BACLD | Q6RSN8\_ACETH | Q79DR3\_ECOLX | Q7SID8\_BACIU | Q7X4S4\_BACLI | Q81DA0\_BACCR | Q88JL2\_PSEPK | Q8P6Z9\_XANCP | Q9AJF8\_ACETH | Q9AJM4\_9BACI | Q9AJS0\_ALIAC | Q9EYQ2\_9FIRM | Q9JZ43\_NEIMB | Q9RC94\_9BACI | Q9XB24\_KLEPN | W5JXE0\_BACSP | W8FKE7\_BACPU | XYN1\_GEOSE | XYNA\_HALH5 | XYNC\_BACSU | YKFC\_BACC1 | GO:0009251 | 0.0114738202973497 | 71/6188 | 15/182 | 1.29851797423244e-09 | 4.22018341625542e-07 | T | T | T | T | glucan catabolic process | A3DJ82\_ACET2 | A8FDC4\_BACP2 | AMT6\_BACS7 | C7ED31\_ACET1 | GUNA\_ACET2 | GUNC\_ACETH | GUNF\_RUMCH | GUNG\_RUMCH | GUNS\_ACETH | GUN\_ECOLI | Q65JI7\_BACLD | Q65JI8\_BACLD | Q6RSN8\_ACETH | Q9AJF8\_ACETH | Q9AJS0\_ALIAC | GO:0006228 | 0.00323206205559147 | 20/6188 | 9/182 | 1.71910225996858e-09 | 5.58708234489789e-07 | T | T | T | T | UTP biosynthetic process | D0CAF1\_ACIB2 | NDK\_ACIBS | NDK\_AQUAE | NDK\_BURTA | NDK\_CAMJE | NDK\_HELPG | NDK\_MYXXA | NDK\_NEIG2 | V5VIC4\_ACIBA | GO:0046051 | 0.00339366515837104 | 21/6188 | 9/182 | 2.93272212853635e-09 | 9.53134691774313e-07 | T | T | T | T | UTP metabolic process | D0CAF1\_ACIB2 | NDK\_ACIBS | NDK\_AQUAE | NDK\_BURTA | NDK\_CAMJE | NDK\_HELPG | NDK\_MYXXA | NDK\_NEIG2 | V5VIC4\_ACIBA | GO:0006183 | 0.00339366515837104 | 21/6188 | 9/182 | 2.93272212853635e-09 | 9.53134691774313e-07 | T | T | T | T | GTP biosynthetic process | D0CAF1\_ACIB2 | NDK\_ACIBS | NDK\_AQUAE | NDK\_BURTA | NDK\_CAMJE | NDK\_HELPG | NDK\_MYXXA | NDK\_NEIG2 | V5VIC4\_ACIBA | GO:0046039 | 0.00339366515837104 | 21/6188 | 9/182 | 2.93272212853635e-09 | 9.53134691774313e-07 | T | T | T | T | GTP metabolic process | D0CAF1\_ACIB2 | NDK\_ACIBS | NDK\_AQUAE | NDK\_BURTA | NDK\_CAMJE | NDK\_HELPG | NDK\_MYXXA | NDK\_NEIG2 | V5VIC4\_ACIBA | GO:0009206 | 0.00404007756948933 | 25/6188 | 9/182 | 1.84089550726738e-08 | 5.982910398619e-06 | T | T | T | T | purine ribonucleoside triphosphate biosynthetic process | D0CAF1\_ACIB2 | NDK\_ACIBS | NDK\_AQUAE | NDK\_BURTA | NDK\_CAMJE | NDK\_HELPG | NDK\_MYXXA | NDK\_NEIG2 | V5VIC4\_ACIBA | GO:0009145 | 0.00404007756948933 | 25/6188 | 9/182 | 1.84089550726738e-08 | 5.982910398619e-06 | T | T | T | T | purine nucleoside triphosphate biosynthetic process | D0CAF1\_ACIB2 | NDK\_ACIBS | NDK\_AQUAE | NDK\_BURTA | NDK\_CAMJE | NDK\_HELPG | NDK\_MYXXA | NDK\_NEIG2 | V5VIC4\_ACIBA | GO:0141060 | 0.00210084033613445 | 13/6188 | 7/182 | 2.50955878851786e-08 | 8.15606606268305e-06 | T | T | T | T | disruption of anatomical structure in another organism | PHL2\_BACCE | PHLC1\_CLOPE | PHLC\_BACCE | PHLC\_CLOP1 | PHLC\_CLOPF | PHLC\_STAAU | PLYL\_DICD3 | GO:0009205 | 0.00420168067226891 | 26/6188 | 9/182 | 2.74468191201473e-08 | 8.92021621404786e-06 | T | T | T | T | purine ribonucleoside triphosphate metabolic process | D0CAF1\_ACIB2 | NDK\_ACIBS | NDK\_AQUAE | NDK\_BURTA | NDK\_CAMJE | NDK\_HELPG | NDK\_MYXXA | NDK\_NEIG2 | V5VIC4\_ACIBA | GO:0044042 | 0.0145442792501616 | 90/6188 | 15/182 | 3.98524396341457e-08 | 1.29520428810973e-05 | T | T | T | T | glucan metabolic process | A3DJ82\_ACET2 | A8FDC4\_BACP2 | AMT6\_BACS7 | C7ED31\_ACET1 | GUNA\_ACET2 | GUNC\_ACETH | GUNF\_RUMCH | GUNG\_RUMCH | GUNS\_ACETH | GUN\_ECOLI | Q65JI7\_BACLD | Q65JI8\_BACLD | Q6RSN8\_ACETH | Q9AJF8\_ACETH | Q9AJS0\_ALIAC | GO:0019538 | 0.0648028442146089 | 401/6188 | 33/182 | 4.33619162675685e-08 | 1.40926227869598e-05 | T | T | T | T | protein metabolic process | A0A0H2WX20\_STAAC | A0A0M3KKW7\_STRPY | A0A4V8H037\_9RHOB | BSAP\_BACSU | BXX\_CLOBO | CBPM\_STRAL | D0VXY8\_STAPS | D8H130\_BACAI | DACC\_BACSU | DAC\_STRSR | ETA\_STAAU | ETB\_STAAU | FLS\_FERPE | GSEA\_STAES | L0RUV7\_STAAU | LYTM\_STAA8 | O68424\_BACFG | O69771\_9PSED | O86049\_BACFG | PEPX\_LACHE | PRZN\_SERME | Q5MJ80\_SERPR | RIPB\_MYCTU | SFAS2\_STRFR | SPLA\_STAA8 | SPLB\_STAA8 | SPLC\_STAA8 | SPLD\_STAA8 | SPLE\_STAA8 | SSL1\_STAA8 | SUBD\_BACLI | V5IRV7\_THETH | YKFC\_BACC1 | GO:0031640 | 0.00193923723335488 | 12/6188 | 6/182 | 4.75749347188518e-07 | 0.000154618537836268 | T | T | T | T | killing of cells of another organism | PHL2\_BACCE | PHLC1\_CLOPE | PHLC\_BACCE | PHLC\_CLOP1 | PHLC\_CLOPF | PHLC\_STAAU | GO:0001906 | 0.00193923723335488 | 12/6188 | 6/182 | 4.75749347188518e-07 | 0.000154618537836268 | T | T | T | T | cell killing | PHL2\_BACCE | PHLC1\_CLOPE | PHLC\_BACCE | PHLC\_CLOP1 | PHLC\_CLOPF | PHLC\_STAAU | GO:0141061 | 0.00193923723335488 | 12/6188 | 6/182 | 4.75749347188518e-07 | 0.000154618537836268 | T | T | T | T | disruption of cell in another organism | PHL2\_BACCE | PHLC1\_CLOPE | PHLC\_BACCE | PHLC\_CLOP1 | PHLC\_CLOPF | PHLC\_STAAU | GO:0009144 | 0.00565610859728507 | 35/6188 | 9/182 | 4.93253927597923e-07 | 0.000160307526469325 | T | T | T | T | purine nucleoside triphosphate metabolic process | D0CAF1\_ACIB2 | NDK\_ACIBS | NDK\_AQUAE | NDK\_BURTA | NDK\_CAMJE | NDK\_HELPG | NDK\_MYXXA | NDK\_NEIG2 | V5VIC4\_ACIBA | GO:0046036 | 0.00581771170006464 | 36/6188 | 9/182 | 6.41155563683908e-07 | 0.00020837555819727 | T | T | T | T | CTP metabolic process | D0CAF1\_ACIB2 | NDK\_ACIBS | NDK\_AQUAE | NDK\_BURTA | NDK\_CAMJE | NDK\_HELPG | NDK\_MYXXA | NDK\_NEIG2 | V5VIC4\_ACIBA | GO:0006241 | 0.00581771170006464 | 36/6188 | 9/182 | 6.41155563683908e-07 | 0.00020837555819727 | T | T | T | T | CTP biosynthetic process | D0CAF1\_ACIB2 | NDK\_ACIBS | NDK\_AQUAE | NDK\_BURTA | NDK\_CAMJE | NDK\_HELPG | NDK\_MYXXA | NDK\_NEIG2 | V5VIC4\_ACIBA | GO:0009209 | 0.00581771170006464 | 36/6188 | 9/182 | 6.41155563683908e-07 | 0.00020837555819727 | T | T | T | T | pyrimidine ribonucleoside triphosphate biosynthetic process | D0CAF1\_ACIB2 | NDK\_ACIBS | NDK\_AQUAE | NDK\_BURTA | NDK\_CAMJE | NDK\_HELPG | NDK\_MYXXA | NDK\_NEIG2 | V5VIC4\_ACIBA | GO:0009208 | 0.00597931480284421 | 37/6188 | 9/182 | 8.25967354029049e-07 | 0.000268439390059441 | T | T | T | T | pyrimidine ribonucleoside triphosphate metabolic process | D0CAF1\_ACIB2 | NDK\_ACIBS | NDK\_AQUAE | NDK\_BURTA | NDK\_CAMJE | NDK\_HELPG | NDK\_MYXXA | NDK\_NEIG2 | V5VIC4\_ACIBA | GO:0016042 | 0.012120232708468 | 75/6188 | 12/182 | 1.51087246376423e-06 | 0.000491033550723373 | T | T | T | T | lipid catabolic process | CHOD\_STRS0 | ESTA\_BACSU | H0B8D4\_9ACTN | LIP\_BACSP | LIP\_PSEAE | NANH\_VIBCH | PLC\_BACCE | PLC\_BACTU | PLC\_LISMO | PLC\_STAAE | PLD\_STRAT | W8FKE7\_BACPU | GO:0009201 | 0.00662572721396251 | 41/6188 | 9/182 | 2.10118303664191e-06 | 0.000682884486908619 | T | T | T | T | ribonucleoside triphosphate biosynthetic process | D0CAF1\_ACIB2 | NDK\_ACIBS | NDK\_AQUAE | NDK\_BURTA | NDK\_CAMJE | NDK\_HELPG | NDK\_MYXXA | NDK\_NEIG2 | V5VIC4\_ACIBA | GO:0009199 | 0.00694893341952165 | 43/6188 | 9/182 | 3.21454117923904e-06 | 0.00104472588325269 | T | T | T | T | ribonucleoside triphosphate metabolic process | D0CAF1\_ACIB2 | NDK\_ACIBS | NDK\_AQUAE | NDK\_BURTA | NDK\_CAMJE | NDK\_HELPG | NDK\_MYXXA | NDK\_NEIG2 | V5VIC4\_ACIBA | GO:0010393 | 0.0024240465416936 | 15/6188 | 5/182 | 4.92281977353478e-05 | 0.015999164263988 | T | T | T | T | galacturonan metabolic process | A0A160EBC2\_ACHDE | PLYL\_DICD3 | PLY\_BACSU | PMEA\_DICD3 | Q9RHW0\_BACSP | GO:0045488 | 0.0024240465416936 | 15/6188 | 5/182 | 4.92281977353478e-05 | 0.015999164263988 | T | T | T | T | pectin metabolic process | A0A160EBC2\_ACHDE | PLYL\_DICD3 | PLY\_BACSU | PMEA\_DICD3 | Q9RHW0\_BACSP | GO:0031667 | 0.00646412411118293 | 40/6188 | 7/182 | 0.000139012687316935 | 0.0451791233780039 | T | T | T | T | response to nutrient levels | A0A0M3KKU6\_ERWAE | B2IF78\_BEII9 | B2VCC3\_ERWT9 | D5DC07\_PRIM3 | D8J9C2\_HALJB | SACB\_BACSU | SACB\_GLUDI | GO:0009148 | 0.0116354234001293 | 72/6188 | 9/182 | 0.000232668535948886 | 0.0756172741833881 | T | T | T | T | pyrimidine nucleoside triphosphate biosynthetic process | D0CAF1\_ACIB2 | NDK\_ACIBS | NDK\_AQUAE | NDK\_BURTA | NDK\_CAMJE | NDK\_HELPG | NDK\_MYXXA | NDK\_NEIG2 | V5VIC4\_ACIBA | GO:0009142 | 0.0124434389140271 | 77/6188 | 9/182 | 0.000388676283004261 | 0.126319791976385 | T | T | T | T | nucleoside triphosphate biosynthetic process | D0CAF1\_ACIB2 | NDK\_ACIBS | NDK\_AQUAE | NDK\_BURTA | NDK\_CAMJE | NDK\_HELPG | NDK\_MYXXA | NDK\_NEIG2 | V5VIC4\_ACIBA | GO:0006665 | 0.00210084033613445 | 13/6188 | 4/182 | 0.000420398550785285 | 0.136629529005218 | T | T | T | T | sphingolipid metabolic process | NANH\_VIBCH | PHL2\_BACCE | PHLC\_STAAU | XYNC\_BACSU | GO:0009991 | 0.00775694893341952 | 48/6188 | 7/182 | 0.000450173128412999 | 0.146306266734225 | T | T | T | T | response to extracellular stimulus | A0A0M3KKU6\_ERWAE | B2IF78\_BEII9 | B2VCC3\_ERWT9 | D5DC07\_PRIM3 | D8J9C2\_HALJB | SACB\_BACSU | SACB\_GLUDI | GO:0045490 | 0.00226244343891403 | 14/6188 | 4/182 | 0.000575111334996171 | 0.186911183873756 | T | T | T | T | pectin catabolic process | A0A160EBC2\_ACHDE | PLYL\_DICD3 | PLY\_BACSU | PMEA\_DICD3 | GO:0044238 | 0.687621202327085 | 4255/6188 | 145/182 | 0.000577586206522079 | 0.187715517119676 | T | T | T | T | primary metabolic process | A0A067XG64\_PARTM | A0A0B7LH44\_STREE | A0A0H2UQE4\_STRPN | A0A0H2WX20\_STAAC | A0A0M3KKW7\_STRPY | A0A160EBC2\_ACHDE | A0A182DW00\_STREE | A0A243G6Q6\_BACTU | A0A4P8ESF9\_BACSP | A0A4V8H037\_9RHOB | A0A6L7H1P2\_BACAN | A1TSQ3\_ACIAC | A3DJ82\_ACET2 | A85C\_MYCTU | A8FDC4\_BACP2 | AMT6\_BACS7 | AMYB\_BACCE | AMYM\_GEOSE | AMYS\_NEIPO | AMY\_BACAM | AMY\_GEOSE | B9MMA5\_CALBD | BSAP\_BACSU | BXX\_CLOBO | C7ED31\_ACET1 | CBPM\_STRAL | CDGT\_BACS0 | CDGT\_GEOSE | CHIS\_BACSU | CHIS\_STRSN | CHOD\_STRS0 | D0CAF1\_ACIB2 | D0VP31\_9BACI | D0VV09\_BACCE | D0VXY8\_STAPS | D8H130\_BACAI | DACC\_BACSU | DAC\_STRSR | DRNE\_VIBCH | E4Q2A4\_CALOW | EBA1\_ELIME | EBA3\_ELIME | ENG1\_ACET2 | ESTA\_BACSU | ESTA\_PSEAE | ETA\_STAAU | ETB\_STAAU | FLS\_FERPE | G0L2Y1\_ZOBGA | G2NRC4\_STREK | G8LZE0\_ACECE | GSEA\_STAES | GUB\_BACLI | GUB\_BACSU | GUNA\_ACET2 | GUNC\_ACETH | GUNF\_RUMCH | GUNG\_RUMCH | GUNS\_ACETH | GUN\_ECOLI | H0B8D4\_9ACTN | HYSA\_STRA3 | I3P686\_BACLI | IABF\_STRAW | K0IUV6\_9ENTR | L0EGW1\_THECK | L0RUV7\_STAAU | LCHMO\_ENTFA | LIP\_BACSP | LIP\_PSEAE | LYTM\_STAA8 | NANB\_STRPN | NANH\_VIBCH | NDK\_ACIBS | NDK\_AQUAE | NDK\_BURTA | NDK\_CAMJE | NDK\_HELPG | NDK\_MYXXA | NDK\_NEIG2 | O30700\_9BACI | O52754\_RHOMR | O68424\_BACFG | O69771\_9PSED | O82839\_BACSP | O86049\_BACFG | P84141\_PAEAU | PEPX\_LACHE | PGLR2\_PECPM | PHL2\_BACCE | PHLC\_STAAU | PLC\_BACCE | PLC\_BACTU | PLC\_LISMO | PLC\_STAAE | PLD\_STRAT | PLYL\_DICD3 | PLY\_BACSU | PMEA\_DICD3 | PRZN\_SERME | PTLY\_THEMA | Q09LY9\_GEOSE | Q17TM8\_ALKHA | Q54276\_SERMA | Q59962\_9ACTN | Q5DZ44\_ALIF1 | Q5MJ80\_SERPR | Q65GB9\_BACLD | Q65JI7\_BACLD | Q65JI8\_BACLD | Q6RSN8\_ACETH | Q7LYT7\_PYRWO | Q7SID8\_BACIU | Q7X4S4\_BACLI | Q82L26\_STRAW | Q88JL2\_PSEPK | Q8P6Z9\_XANCP | Q93I48\_9BACI | Q9AJF8\_ACETH | Q9AJM4\_9BACI | Q9AJS0\_ALIAC | Q9EYQ2\_9FIRM | Q9KWY6\_GEOSE | Q9RC94\_9BACI | Q9REI6\_ARTSP | Q9RHW0\_BACSP | RHA78\_STRAW | RIPB\_MYCTU | SFAS2\_STRFR | SPLA\_STAA8 | SPLB\_STAA8 | SPLC\_STAA8 | SPLD\_STAA8 | SPLE\_STAA8 | SSL1\_STAA8 | SUBD\_BACLI | V5IRV7\_THETH | V5VIC4\_ACIBA | W5JXE0\_BACSP | W8FKE7\_BACPU | XANLY\_BACGL | XYN1\_GEOSE | XYNA\_HALH5 | XYNC\_BACSU | YKFC\_BACC1 | GO:0030655 | 0.013251454427925 | 82/6188 | 9/182 | 0.000622367892719464 | 0.202269565133826 | T | T | T | T | beta-lactam antibiotic catabolic process | A5N1B8\_CLOK5 | BLAC\_BACSU | BLAC\_STRAL | D2ARB3\_STRRD | D5DH82\_PRIM3 | D9TA79\_MICAI | P94458\_BACLI | Q79DR3\_ECOLX | Q81DA0\_BACCR | GO:0030653 | 0.0134130575307046 | 83/6188 | 9/182 | 0.000680679429974636 | 0.221220814741757 | T | T | T | T | beta-lactam antibiotic metabolic process | A5N1B8\_CLOK5 | BLAC\_BACSU | BLAC\_STRAL | D2ARB3\_STRRD | D5DH82\_PRIM3 | D9TA79\_MICAI | P94458\_BACLI | Q79DR3\_ECOLX | Q81DA0\_BACCR | GO:0072340 | 0.0135746606334842 | 84/6188 | 9/182 | 0.000743382209634718 | 0.241599218131283 | T | T | T | T | lactam catabolic process | A5N1B8\_CLOK5 | BLAC\_BACSU | BLAC\_STRAL | D2ARB3\_STRRD | D5DH82\_PRIM3 | D9TA79\_MICAI | P94458\_BACLI | Q79DR3\_ECOLX | Q81DA0\_BACCR | GO:0009147 | 0.0135746606334842 | 84/6188 | 9/182 | 0.000743382209634718 | 0.241599218131283 | T | T | T | T | pyrimidine nucleoside triphosphate metabolic process | D0CAF1\_ACIB2 | NDK\_ACIBS | NDK\_AQUAE | NDK\_BURTA | NDK\_CAMJE | NDK\_HELPG | NDK\_MYXXA | NDK\_NEIG2 | V5VIC4\_ACIBA | GO:0098661 | 0.000323206205559147 | 2/6188 | 2/182 | 0.000860437920117035 | 0.279642324038036 | T | T | T | T | inorganic anion transmembrane transport | C3K8K1\_PSEFS | D0VWY2\_PSEFS | GO:0035435 | 0.000323206205559147 | 2/6188 | 2/182 | 0.000860437920117035 | 0.279642324038036 | T | T | T | T | phosphate ion transmembrane transport | C3K8K1\_PSEFS | D0VWY2\_PSEFS | GO:0006817 | 0.000323206205559147 | 2/6188 | 2/182 | 0.000860437920117035 | 0.279642324038036 | T | T | T | T | phosphate ion transport | C3K8K1\_PSEFS | D0VWY2\_PSEFS | GO:0015698 | 0.000323206205559147 | 2/6188 | 2/182 | 0.000860437920117035 | 0.279642324038036 | T | T | T | T | inorganic anion transport | C3K8K1\_PSEFS | D0VWY2\_PSEFS | GO:0072338 | 0.0142210730446025 | 88/6188 | 9/182 | 0.00104312485583572 | 0.339015578146608 | T | T | T | T | lactam metabolic process | A5N1B8\_CLOK5 | BLAC\_BACSU | BLAC\_STRAL | D2ARB3\_STRRD | D5DH82\_PRIM3 | D9TA79\_MICAI | P94458\_BACLI | Q79DR3\_ECOLX | Q81DA0\_BACCR | GO:0033212 | 0.00145442792501616 | 9/6188 | 3/182 | 0.0018444854802855 | 0.599457781092788 | T | T | T | F | iron import into cell | EFEN\_BACSU | Q9RKQ2\_STRCO | Q9ZBW9\_STRCO | GO:0009141 | 0.0159987071751778 | 99/6188 | 9/182 | 0.00239654962232586 | 0.778878627255904 | T | T | T | F | nucleoside triphosphate metabolic process | D0CAF1\_ACIB2 | NDK\_ACIBS | NDK\_AQUAE | NDK\_BURTA | NDK\_CAMJE | NDK\_HELPG | NDK\_MYXXA | NDK\_NEIG2 | V5VIC4\_ACIBA | GO:0009220 | 0.0159987071751778 | 99/6188 | 9/182 | 0.00239654962232586 | 0.778878627255904 | T | T | T | F | pyrimidine ribonucleotide biosynthetic process | D0CAF1\_ACIB2 | NDK\_ACIBS | NDK\_AQUAE | NDK\_BURTA | NDK\_CAMJE | NDK\_HELPG | NDK\_MYXXA | NDK\_NEIG2 | V5VIC4\_ACIBA | GO:0009218 | 0.0171299288946348 | 106/6188 | 9/182 | 0.00381398061120466 | 1 | T | T | F | F | pyrimidine ribonucleotide metabolic process | D0CAF1\_ACIB2 | NDK\_ACIBS | NDK\_AQUAE | NDK\_BURTA | NDK\_CAMJE | NDK\_HELPG | NDK\_MYXXA | NDK\_NEIG2 | V5VIC4\_ACIBA | GO:0017001 | 0.0277957336780866 | 172/6188 | 12/182 | 0.00456286156605778 | 1 | T | T | F | F | antibiotic catabolic process | A0A4V8H037\_9RHOB | A5N1B8\_CLOK5 | B4EPS2\_BURCJ | BLAC\_BACSU | BLAC\_STRAL | D2ARB3\_STRRD | D5DH82\_PRIM3 | D9TA79\_MICAI | P94458\_BACLI | Q79DR3\_ECOLX | Q81DA0\_BACCR | Q9XB24\_KLEPN | GO:0046677 | 0.043956043956044 | 272/6188 | 16/182 | 0.00609138208541941 | 1 | T | T | F | F | response to antibiotic | A0A4V8H037\_9RHOB | A5N1B8\_CLOK5 | A85B\_MYCTU | A85C\_MYCTU | B4EPS2\_BURCJ | BLAC\_BACSU | BLAC\_STRAL | D2ARB3\_STRRD | D5DH82\_PRIM3 | D9TA79\_MICAI | HCPB\_HELPY | HCPC\_HELPY | P94458\_BACLI | Q79DR3\_ECOLX | Q81DA0\_BACCR | Q9XB24\_KLEPN | GO:0051649 | 0.00226244343891403 | 14/6188 | 3/182 | 0.00717095184174768 | 1 | T | T | F | F | establishment of localization in cell | EFEN\_BACSU | Q9RKQ2\_STRCO | Q9ZBW9\_STRCO | GO:0006643 | 0.0096961861667744 | 60/6188 | 6/182 | 0.00799573891493561 | 1 | T | T | F | F | membrane lipid metabolic process | A85C\_MYCTU | ESTA\_PSEAE | NANH\_VIBCH | PHL2\_BACCE | PHLC\_STAAU | XYNC\_BACSU | GO:0006026 | 0.00468648998060763 | 29/6188 | 4/182 | 0.00967335534314601 | 1 | T | T | F | F | aminoglycan catabolic process | A0R5R2\_MYCS2 | LCHMO\_ENTFA | O06496\_CLOPF | O83009\_SERMA | GO:0044419 | 0.0166451195862961 | 103/6188 | 8/182 | 0.0105735331045231 | 1 | T | T | F | F | biological process involved in interspecies interaction between organisms | A85B\_MYCTU | PHL2\_BACCE | PHLC1\_CLOPE | PHLC\_BACCE | PHLC\_CLOP1 | PHLC\_CLOPF | PHLC\_STAAU | PLYL\_DICD3 | GO:0051234 | 0.0134130575307046 | 83/6188 | 7/182 | 0.0107292331162775 | 1 | T | T | F | F | establishment of localization | A85C\_MYCTU | C3K8K1\_PSEFS | D0VWY2\_PSEFS | EFEN\_BACSU | LIP\_PSEAE | Q9RKQ2\_STRCO | Q9ZBW9\_STRCO | GO:0006810 | 0.0134130575307046 | 83/6188 | 7/182 | 0.0107292331162775 | 1 | T | T | F | F | transport | A85C\_MYCTU | C3K8K1\_PSEFS | D0VWY2\_PSEFS | EFEN\_BACSU | LIP\_PSEAE | Q9RKQ2\_STRCO | Q9ZBW9\_STRCO | GO:0051179 | 0.0135746606334842 | 84/6188 | 7/182 | 0.0114222851205698 | 1 | T | T | F | F | localization | A85C\_MYCTU | C3K8K1\_PSEFS | D0VWY2\_PSEFS | EFEN\_BACSU | LIP\_PSEAE | Q9RKQ2\_STRCO | Q9ZBW9\_STRCO | GO:0098660 | 0.00129282482223659 | 8/6188 | 2/182 | 0.0214356946492661 | 1 | T | F | F | F | inorganic ion transmembrane transport | C3K8K1\_PSEFS | D0VWY2\_PSEFS | GO:0006221 | 0.0235940530058177 | 146/6188 | 9/182 | 0.02768201343767 | 1 | T | F | F | F | pyrimidine nucleotide biosynthetic process | D0CAF1\_ACIB2 | NDK\_ACIBS | NDK\_AQUAE | NDK\_BURTA | NDK\_CAMJE | NDK\_HELPG | NDK\_MYXXA | NDK\_NEIG2 | V5VIC4\_ACIBA | GO:0009605 | 0.0198771816418875 | 123/6188 | 8/182 | 0.0279907300237027 | 1 | T | F | F | F | response to external stimulus | A0A0M3KKU6\_ERWAE | A85B\_MYCTU | B2IF78\_BEII9 | B2VCC3\_ERWT9 | D5DC07\_PRIM3 | D8J9C2\_HALJB | SACB\_BACSU | SACB\_GLUDI | GO:0006032 | 0.00161603102779573 | 10/6188 | 2/182 | 0.0331469148298428 | 1 | T | F | F | F | chitin catabolic process | LCHMO\_ENTFA | O83009\_SERMA | GO:0006030 | 0.00161603102779573 | 10/6188 | 2/182 | 0.0331469148298428 | 1 | T | F | F | F | chitin metabolic process | LCHMO\_ENTFA | O83009\_SERMA | GO:0031222 | 0.00161603102779573 | 10/6188 | 2/182 | 0.0331469148298428 | 1 | T | F | F | F | arabinan catabolic process | IABF\_STRAW | Q65GB9\_BACLD | GO:0031221 | 0.00177763413057531 | 11/6188 | 2/182 | 0.0397420307637683 | 1 | T | F | F | F | arabinan metabolic process | IABF\_STRAW | Q65GB9\_BACLD | GO:0006220 | 0.0255332902391726 | 158/6188 | 9/182 | 0.0427183348905895 | 1 | T | F | F | F | pyrimidine nucleotide metabolic process | D0CAF1\_ACIB2 | NDK\_ACIBS | NDK\_AQUAE | NDK\_BURTA | NDK\_CAMJE | NDK\_HELPG | NDK\_MYXXA | NDK\_NEIG2 | V5VIC4\_ACIBA | GO:0042221 | 0.0586619263089851 | 363/6188 | 16/182 | 0.0672834654010124 | 1 | F | F | F | F | response to chemical | A0A4V8H037\_9RHOB | A5N1B8\_CLOK5 | A85B\_MYCTU | A85C\_MYCTU | B4EPS2\_BURCJ | BLAC\_BACSU | BLAC\_STRAL | D2ARB3\_STRRD | D5DH82\_PRIM3 | D9TA79\_MICAI | HCPB\_HELPY | HCPC\_HELPY | P94458\_BACLI | Q79DR3\_ECOLX | Q81DA0\_BACCR | Q9XB24\_KLEPN | GO:0071555 | 0.0321590174531351 | 199/6188 | 10/182 | 0.0679412391067792 | 1 | F | F | F | F | cell wall organization | CBPM\_STRAL | DACC\_BACSU | DAC\_STRSR | ENG1\_ACET2 | LYTM\_STAA8 | PGLR2\_PECPM | PMEA\_DICD3 | PTLY\_THEMA | RIPB\_MYCTU | YKFC\_BACC1 | GO:0071554 | 0.0324822236586942 | 201/6188 | 10/182 | 0.0716326213236541 | 1 | F | F | F | F | cell wall organization or biogenesis | CBPM\_STRAL | DACC\_BACSU | DAC\_STRSR | ENG1\_ACET2 | LYTM\_STAA8 | PGLR2\_PECPM | PMEA\_DICD3 | PTLY\_THEMA | RIPB\_MYCTU | YKFC\_BACC1 | GO:0055085 | 0.00549450549450549 | 34/6188 | 3/182 | 0.077007392149318 | 1 | F | F | F | F | transmembrane transport | C3K8K1\_PSEFS | D0VWY2\_PSEFS | LIP\_PSEAE | GO:0009253 | 0.00258564964447317 | 16/6188 | 2/182 | 0.0788257836571294 | 1 | F | F | F | F | peptidoglycan catabolic process | A0R5R2\_MYCS2 | O06496\_CLOPF | GO:0045229 | 0.0332902391725921 | 206/6188 | 10/182 | 0.0814093273668962 | 1 | F | F | F | F | external encapsulating structure organization | CBPM\_STRAL | DACC\_BACSU | DAC\_STRSR | ENG1\_ACET2 | LYTM\_STAA8 | PGLR2\_PECPM | PMEA\_DICD3 | PTLY\_THEMA | RIPB\_MYCTU | YKFC\_BACC1 | GO:0006826 | 0.00565610859728507 | 35/6188 | 3/182 | 0.0824821305803831 | 1 | F | F | F | F | iron ion transport | EFEN\_BACSU | Q9RKQ2\_STRCO | Q9ZBW9\_STRCO | GO:1901072 | 0.00274725274725275 | 17/6188 | 2/182 | 0.0876616559099276 | 1 | F | F | F | F | glucosamine-containing compound catabolic process | LCHMO\_ENTFA | O83009\_SERMA | GO:0000041 | 0.00614091790562379 | 38/6188 | 3/182 | 0.0998637553450399 | 1 | F | F | F | F | transition metal ion transport | EFEN\_BACSU | Q9RKQ2\_STRCO | Q9ZBW9\_STRCO | GO:0006879 | 0.00630252100840336 | 39/6188 | 3/182 | 0.105959915343356 | 1 | F | F | F | F | intracellular iron ion homeostasis | EFEN\_BACSU | Q9RKQ2\_STRCO | Q9ZBW9\_STRCO | GO:0006027 | 0.00307045895281189 | 19/6188 | 2/182 | 0.106144804818017 | 1 | F | F | F | F | glycosaminoglycan catabolic process | A0R5R2\_MYCS2 | O06496\_CLOPF | GO:0098771 | 0.00646412411118293 | 40/6188 | 3/182 | 0.112198543168553 | 1 | F | F | F | F | inorganic ion homeostasis | EFEN\_BACSU | Q9RKQ2\_STRCO | Q9ZBW9\_STRCO | GO:0030001 | 0.00678733031674208 | 42/6188 | 3/182 | 0.125081879197179 | 1 | F | F | F | F | metal ion transport | EFEN\_BACSU | Q9RKQ2\_STRCO | Q9ZBW9\_STRCO | GO:0072528 | 0.0319974143503555 | 198/6188 | 9/182 | 0.128431106716304 | 1 | F | F | F | F | pyrimidine-containing compound biosynthetic process | D0CAF1\_ACIB2 | NDK\_ACIBS | NDK\_AQUAE | NDK\_BURTA | NDK\_CAMJE | NDK\_HELPG | NDK\_MYXXA | NDK\_NEIG2 | V5VIC4\_ACIBA | GO:0055080 | 0.00694893341952165 | 43/6188 | 3/182 | 0.131715808282262 | 1 | F | F | F | F | monoatomic cation homeostasis | EFEN\_BACSU | Q9RKQ2\_STRCO | Q9ZBW9\_STRCO | GO:0006873 | 0.00694893341952165 | 43/6188 | 3/182 | 0.131715808282262 | 1 | F | F | F | F | intracellular monoatomic ion homeostasis | EFEN\_BACSU | Q9RKQ2\_STRCO | Q9ZBW9\_STRCO | GO:0050801 | 0.00694893341952165 | 43/6188 | 3/182 | 0.131715808282262 | 1 | F | F | F | F | monoatomic ion homeostasis | EFEN\_BACSU | Q9RKQ2\_STRCO | Q9ZBW9\_STRCO | GO:0030003 | 0.00694893341952165 | 43/6188 | 3/182 | 0.131715808282262 | 1 | F | F | F | F | intracellular monoatomic cation homeostasis | EFEN\_BACSU | Q9RKQ2\_STRCO | Q9ZBW9\_STRCO | GO:0006812 | 0.00711053652230123 | 44/6188 | 3/182 | 0.138470675017605 | 1 | F | F | F | F | monoatomic cation transport | EFEN\_BACSU | Q9RKQ2\_STRCO | Q9ZBW9\_STRCO | GO:0009308 | 0.0331286360698125 | 205/6188 | 9/182 | 0.149189168048985 | 1 | F | F | F | F | amine metabolic process | A5N1B8\_CLOK5 | BLAC\_BACSU | BLAC\_STRAL | D2ARB3\_STRRD | D5DH82\_PRIM3 | D9TA79\_MICAI | P94458\_BACLI | Q79DR3\_ECOLX | Q81DA0\_BACCR | GO:1901071 | 0.00387847446670976 | 24/6188 | 2/182 | 0.156052651844737 | 1 | F | F | F | F | glucosamine-containing compound metabolic process | LCHMO\_ENTFA | O83009\_SERMA | GO:0006811 | 0.00759534583063995 | 47/6188 | 3/182 | 0.159406452713976 | 1 | F | F | F | F | monoatomic ion transport | EFEN\_BACSU | Q9RKQ2\_STRCO | Q9ZBW9\_STRCO | GO:0016999 | 0.0479961215255333 | 297/6188 | 12/182 | 0.163796032486451 | 1 | F | F | F | F | antibiotic metabolic process | A0A4V8H037\_9RHOB | A5N1B8\_CLOK5 | B4EPS2\_BURCJ | BLAC\_BACSU | BLAC\_STRAL | D2ARB3\_STRRD | D5DH82\_PRIM3 | D9TA79\_MICAI | P94458\_BACLI | Q79DR3\_ECOLX | Q81DA0\_BACCR | Q9XB24\_KLEPN | GO:0006664 | 0.00791855203619909 | 49/6188 | 3/182 | 0.173868679627937 | 1 | F | F | F | F | glycolipid metabolic process | A85C\_MYCTU | ESTA\_PSEAE | NANH\_VIBCH | GO:0055082 | 0.00840336134453781 | 52/6188 | 3/182 | 0.196212348903429 | 1 | F | F | F | F | intracellular chemical homeostasis | EFEN\_BACSU | Q9RKQ2\_STRCO | Q9ZBW9\_STRCO | GO:0048878 | 0.00840336134453781 | 52/6188 | 3/182 | 0.196212348903429 | 1 | F | F | F | F | chemical homeostasis | EFEN\_BACSU | Q9RKQ2\_STRCO | Q9ZBW9\_STRCO | GO:0050896 | 0.105365223012282 | 652/6188 | 23/182 | 0.204644329282571 | 1 | F | F | F | F | response to stimulus | A0A0M3KKU6\_ERWAE | A0A4V8H037\_9RHOB | A5N1B8\_CLOK5 | A85B\_MYCTU | A85C\_MYCTU | B2IF78\_BEII9 | B2VCC3\_ERWT9 | B4EPS2\_BURCJ | BLAC\_BACSU | BLAC\_STRAL | D2ARB3\_STRRD | D5DC07\_PRIM3 | D5DH82\_PRIM3 | D8J9C2\_HALJB | D9TA79\_MICAI | HCPB\_HELPY | HCPC\_HELPY | P94458\_BACLI | Q79DR3\_ECOLX | Q81DA0\_BACCR | Q9XB24\_KLEPN | SACB\_BACSU | SACB\_GLUDI | GO:0009152 | 0.0378151260504202 | 234/6188 | 9/182 | 0.250642992366663 | 1 | F | F | F | F | purine ribonucleotide biosynthetic process | D0CAF1\_ACIB2 | NDK\_ACIBS | NDK\_AQUAE | NDK\_BURTA | NDK\_CAMJE | NDK\_HELPG | NDK\_MYXXA | NDK\_NEIG2 | V5VIC4\_ACIBA | GO:0071702 | 0.00533290239172592 | 33/6188 | 2/182 | 0.253172308774662 | 1 | F | F | F | F | organic substance transport | A85C\_MYCTU | LIP\_PSEAE | GO:0016043 | 0.0479961215255333 | 297/6188 | 11/182 | 0.257188518807028 | 1 | F | F | F | F | cellular component organization | A85C\_MYCTU | CBPM\_STRAL | DACC\_BACSU | DAC\_STRSR | ENG1\_ACET2 | LYTM\_STAA8 | PGLR2\_PECPM | PMEA\_DICD3 | PTLY\_THEMA | RIPB\_MYCTU | YKFC\_BACC1 | GO:0072527 | 0.0389463477698772 | 241/6188 | 9/182 | 0.278128101044524 | 1 | F | F | F | F | pyrimidine-containing compound metabolic process | D0CAF1\_ACIB2 | NDK\_ACIBS | NDK\_AQUAE | NDK\_BURTA | NDK\_CAMJE | NDK\_HELPG | NDK\_MYXXA | NDK\_NEIG2 | V5VIC4\_ACIBA | GO:0071840 | 0.0494505494505494 | 306/6188 | 11/182 | 0.289025083707853 | 1 | F | F | F | F | cellular component organization or biogenesis | A85C\_MYCTU | CBPM\_STRAL | DACC\_BACSU | DAC\_STRSR | ENG1\_ACET2 | LYTM\_STAA8 | PGLR2\_PECPM | PMEA\_DICD3 | PTLY\_THEMA | RIPB\_MYCTU | YKFC\_BACC1 | GO:0016311 | 0.00614091790562379 | 38/6188 | 2/182 | 0.30814922195251 | 1 | F | F | F | F | dephosphorylation | A0A378K9X8\_LEGPN | Q6MNP0\_BDEBA | GO:0046348 | 0.00646412411118293 | 40/6188 | 2/182 | 0.329966785792412 | 1 | F | F | F | F | amino sugar catabolic process | LCHMO\_ENTFA | O83009\_SERMA | GO:0071704 | 0.861829347123465 | 5333/6188 | 159/182 | 0.368019776144067 | 1 | F | F | F | F | organic substance metabolic process | A0A067XG64\_PARTM | A0A0B7LH44\_STREE | A0A0H2UQE4\_STRPN | A0A0H2WX20\_STAAC | A0A0M3KKW7\_STRPY | A0A160EBC2\_ACHDE | A0A182DW00\_STREE | A0A243G6Q6\_BACTU | A0A4P8ESF9\_BACSP | A0A4V8H037\_9RHOB | A0A6L7H1P2\_BACAN | A0R5R2\_MYCS2 | A1TSQ3\_ACIAC | A3DJ82\_ACET2 | A5N1B8\_CLOK5 | A85C\_MYCTU | A8FDC4\_BACP2 | AMT6\_BACS7 | AMYB\_BACCE | AMYM\_GEOSE | AMYS\_NEIPO | AMY\_BACAM | AMY\_GEOSE | B9MMA5\_CALBD | BLAC\_BACSU | BLAC\_STRAL | BSAP\_BACSU | BXX\_CLOBO | C7ED31\_ACET1 | CBPM\_STRAL | CDGT\_BACS0 | CDGT\_GEOSE | CHIS\_BACSU | CHIS\_STRSN | CHOD\_STRS0 | D0CAF1\_ACIB2 | D0VP31\_9BACI | D0VV09\_BACCE | D0VXY8\_STAPS | D2ARB3\_STRRD | D5DH82\_PRIM3 | D8H130\_BACAI | D9TA79\_MICAI | DACC\_BACSU | DAC\_STRSR | DRNE\_VIBCH | E4Q2A4\_CALOW | EBA1\_ELIME | EBA3\_ELIME | ENG1\_ACET2 | ESTA\_BACSU | ESTA\_PSEAE | ETA\_STAAU | ETB\_STAAU | FLS\_FERPE | G0L2Y1\_ZOBGA | G2NRC4\_STREK | G8LZE0\_ACECE | GSEA\_STAES | GUB\_BACLI | GUB\_BACSU | GUNA\_ACET2 | GUNC\_ACETH | GUNF\_RUMCH | GUNG\_RUMCH | GUNS\_ACETH | GUN\_ECOLI | H0B8D4\_9ACTN | HYSA\_STRA3 | I3P686\_BACLI | IABF\_STRAW | K0IUV6\_9ENTR | L0EGW1\_THECK | L0RUV7\_STAAU | LCHMO\_ENTFA | LIP\_BACSP | LIP\_PSEAE | LYTM\_STAA8 | NANB\_STRPN | NANH\_VIBCH | NDK\_ACIBS | NDK\_AQUAE | NDK\_BURTA | NDK\_CAMJE | NDK\_HELPG | NDK\_MYXXA | NDK\_NEIG2 | O06496\_CLOPF | O30700\_9BACI | O52754\_RHOMR | O68424\_BACFG | O69771\_9PSED | O82839\_BACSP | O83009\_SERMA | O86049\_BACFG | P84141\_PAEAU | P94458\_BACLI | PEPX\_LACHE | PGLR2\_PECPM | PHL2\_BACCE | PHLC\_STAAU | PLC\_BACCE | PLC\_BACTU | PLC\_LISMO | PLC\_STAAE | PLD\_STRAT | PLYL\_DICD3 | PLY\_BACSU | PMEA\_DICD3 | PRZN\_SERME | PTLY\_THEMA | Q09LY9\_GEOSE | Q17TM8\_ALKHA | Q21KE5\_SACD2 | Q54276\_SERMA | Q59962\_9ACTN | Q5DZ44\_ALIF1 | Q5MJ80\_SERPR | Q65GB9\_BACLD | Q65JI7\_BACLD | Q65JI8\_BACLD | Q6RSN8\_ACETH | Q79DR3\_ECOLX | Q7LYT7\_PYRWO | Q7SID8\_BACIU | Q7X4S4\_BACLI | Q81DA0\_BACCR | Q82L26\_STRAW | Q88JL2\_PSEPK | Q8P6Z9\_XANCP | Q93I48\_9BACI | Q9AJF8\_ACETH | Q9AJM4\_9BACI | Q9AJS0\_ALIAC | Q9EYQ2\_9FIRM | Q9JZ43\_NEIMB | Q9KWY6\_GEOSE | Q9RC94\_9BACI | Q9REI6\_ARTSP | Q9RHW0\_BACSP | RHA78\_STRAW | RIPB\_MYCTU | SFAS2\_STRFR | SPLA\_STAA8 | SPLB\_STAA8 | SPLC\_STAA8 | SPLD\_STAA8 | SPLE\_STAA8 | SSL1\_STAA8 | SUBD\_BACLI | V5IRV7\_THETH | V5VIC4\_ACIBA | W5JXE0\_BACSP | W8FKE7\_BACPU | XANLY\_BACGL | XYN1\_GEOSE | XYNA\_HALH5 | XYNC\_BACSU | YKFC\_BACC1 | GO:0009247 | 0.0072721396250808 | 45/6188 | 2/182 | 0.383566727455598 | 1 | F | F | F | F | glycolipid biosynthetic process | A85C\_MYCTU | ESTA\_PSEAE | GO:0046467 | 0.00775694893341952 | 48/6188 | 2/182 | 0.414851937882131 | 1 | F | F | F | F | membrane lipid biosynthetic process | A85C\_MYCTU | ESTA\_PSEAE | GO:1903509 | 0.0134130575307046 | 83/6188 | 3/182 | 0.443447144789102 | 1 | F | F | F | F | liposaccharide metabolic process | A85C\_MYCTU | ESTA\_PSEAE | NANH\_VIBCH | GO:0042592 | 0.0138978668390433 | 86/6188 | 3/182 | 0.466627604035251 | 1 | F | F | F | F | homeostatic process | EFEN\_BACSU | Q9RKQ2\_STRCO | Q9ZBW9\_STRCO | GO:0019725 | 0.0138978668390433 | 86/6188 | 3/182 | 0.466627604035251 | 1 | F | F | F | F | cellular homeostasis | EFEN\_BACSU | Q9RKQ2\_STRCO | Q9ZBW9\_STRCO | GO:0046700 | 0.0517129928894635 | 320/6188 | 10/182 | 0.46881210590775 | 1 | F | F | F | F | heterocycle catabolic process | A5N1B8\_CLOK5 | BLAC\_BACSU | BLAC\_STRAL | D2ARB3\_STRRD | D5DH82\_PRIM3 | D9TA79\_MICAI | DRNE\_VIBCH | P94458\_BACLI | Q79DR3\_ECOLX | Q81DA0\_BACCR | GO:0009150 | 0.0463800904977376 | 287/6188 | 9/182 | 0.471060062210109 | 1 | F | F | F | F | purine ribonucleotide metabolic process | D0CAF1\_ACIB2 | NDK\_ACIBS | NDK\_AQUAE | NDK\_BURTA | NDK\_CAMJE | NDK\_HELPG | NDK\_MYXXA | NDK\_NEIG2 | V5VIC4\_ACIBA | GO:0006022 | 0.0311893988364577 | 193/6188 | 6/182 | 0.504513145559986 | 1 | F | F | F | F | aminoglycan metabolic process | A0R5R2\_MYCS2 | DACC\_BACSU | DAC\_STRSR | LCHMO\_ENTFA | O06496\_CLOPF | O83009\_SERMA | GO:0006629 | 0.0953458306399483 | 590/6188 | 17/182 | 0.57415360948507 | 1 | F | F | F | F | lipid metabolic process | A85C\_MYCTU | CHOD\_STRS0 | ESTA\_BACSU | ESTA\_PSEAE | H0B8D4\_9ACTN | LIP\_BACSP | LIP\_PSEAE | NANH\_VIBCH | PHL2\_BACCE | PHLC\_STAAU | PLC\_BACCE | PLC\_BACTU | PLC\_LISMO | PLC\_STAAE | PLD\_STRAT | W8FKE7\_BACPU | XYNC\_BACSU | GO:0006164 | 0.0505817711700065 | 313/6188 | 9/182 | 0.577464659784387 | 1 | F | F | F | F | purine nucleotide biosynthetic process | D0CAF1\_ACIB2 | NDK\_ACIBS | NDK\_AQUAE | NDK\_BURTA | NDK\_CAMJE | NDK\_HELPG | NDK\_MYXXA | NDK\_NEIG2 | V5VIC4\_ACIBA | GO:0009260 | 0.0509049773755656 | 315/6188 | 9/182 | 0.585283176148501 | 1 | F | F | F | F | ribonucleotide biosynthetic process | D0CAF1\_ACIB2 | NDK\_ACIBS | NDK\_AQUAE | NDK\_BURTA | NDK\_CAMJE | NDK\_HELPG | NDK\_MYXXA | NDK\_NEIG2 | V5VIC4\_ACIBA | GO:0072522 | 0.0520361990950226 | 322/6188 | 9/182 | 0.612122249081852 | 1 | F | F | F | F | purine-containing compound biosynthetic process | D0CAF1\_ACIB2 | NDK\_ACIBS | NDK\_AQUAE | NDK\_BURTA | NDK\_CAMJE | NDK\_HELPG | NDK\_MYXXA | NDK\_NEIG2 | V5VIC4\_ACIBA | GO:1901565 | 0.0866192630898513 | 536/6188 | 15/182 | 0.620499825810924 | 1 | F | F | F | F | organonitrogen compound catabolic process | A0R5R2\_MYCS2 | A5N1B8\_CLOK5 | BLAC\_BACSU | BLAC\_STRAL | D2ARB3\_STRRD | D5DH82\_PRIM3 | D9TA79\_MICAI | LCHMO\_ENTFA | LYTM\_STAA8 | NANH\_VIBCH | O06496\_CLOPF | O83009\_SERMA | P94458\_BACLI | Q79DR3\_ECOLX | Q81DA0\_BACCR | GO:0046390 | 0.0526826115061409 | 326/6188 | 9/182 | 0.62706783870466 | 1 | F | F | F | F | ribose phosphate biosynthetic process | D0CAF1\_ACIB2 | NDK\_ACIBS | NDK\_AQUAE | NDK\_BURTA | NDK\_CAMJE | NDK\_HELPG | NDK\_MYXXA | NDK\_NEIG2 | V5VIC4\_ACIBA | GO:0006040 | 0.012120232708468 | 75/6188 | 2/182 | 0.652997739638816 | 1 | F | F | F | F | amino sugar metabolic process | LCHMO\_ENTFA | O83009\_SERMA | GO:1901361 | 0.0657724628312864 | 407/6188 | 11/182 | 0.659872167255217 | 1 | F | F | F | F | organic cyclic compound catabolic process | A5N1B8\_CLOK5 | BLAC\_BACSU | BLAC\_STRAL | CHOD\_STRS0 | D2ARB3\_STRRD | D5DH82\_PRIM3 | D9TA79\_MICAI | DRNE\_VIBCH | P94458\_BACLI | Q79DR3\_ECOLX | Q81DA0\_BACCR | GO:1901136 | 0.031835811247576 | 197/6188 | 5/182 | 0.694405199193214 | 1 | F | F | F | F | carbohydrate derivative catabolic process | A0R5R2\_MYCS2 | LCHMO\_ENTFA | NANH\_VIBCH | O06496\_CLOPF | O83009\_SERMA | GO:0050794 | 0.013251454427925 | 82/6188 | 2/182 | 0.700675574580981 | 1 | F | F | F | F | regulation of cellular process | A85B\_MYCTU | BXX\_CLOBO | GO:0016310 | 0.057207498383969 | 354/6188 | 9/182 | 0.722620499557859 | 1 | F | F | F | F | phosphorylation | D0CAF1\_ACIB2 | NDK\_ACIBS | NDK\_AQUAE | NDK\_BURTA | NDK\_CAMJE | NDK\_HELPG | NDK\_MYXXA | NDK\_NEIG2 | V5VIC4\_ACIBA | GO:0000270 | 0.0290885585003232 | 180/6188 | 4/182 | 0.782271218351717 | 1 | F | F | F | F | peptidoglycan metabolic process | A0R5R2\_MYCS2 | DACC\_BACSU | DAC\_STRSR | O06496\_CLOPF | GO:0009259 | 0.0607627666451196 | 376/6188 | 9/182 | 0.785516402020737 | 1 | F | F | F | F | ribonucleotide metabolic process | D0CAF1\_ACIB2 | NDK\_ACIBS | NDK\_AQUAE | NDK\_BURTA | NDK\_CAMJE | NDK\_HELPG | NDK\_MYXXA | NDK\_NEIG2 | V5VIC4\_ACIBA | GO:0030203 | 0.0295733678086619 | 183/6188 | 4/182 | 0.793077180859677 | 1 | F | F | F | F | glycosaminoglycan metabolic process | A0R5R2\_MYCS2 | DACC\_BACSU | DAC\_STRSR | O06496\_CLOPF | GO:0043603 | 0.0740142210730446 | 458/6188 | 11/182 | 0.800644773655454 | 1 | F | F | F | F | amide metabolic process | A5N1B8\_CLOK5 | BLAC\_BACSU | BLAC\_STRAL | D2ARB3\_STRRD | D5DH82\_PRIM3 | D9TA79\_MICAI | LYTM\_STAA8 | NANH\_VIBCH | P94458\_BACLI | Q79DR3\_ECOLX | Q81DA0\_BACCR | GO:0019693 | 0.0633484162895928 | 392/6188 | 9/182 | 0.824366419594751 | 1 | F | F | F | F | ribose phosphate metabolic process | D0CAF1\_ACIB2 | NDK\_ACIBS | NDK\_AQUAE | NDK\_BURTA | NDK\_CAMJE | NDK\_HELPG | NDK\_MYXXA | NDK\_NEIG2 | V5VIC4\_ACIBA | GO:0006163 | 0.0662572721396251 | 410/6188 | 9/182 | 0.861436907653621 | 1 | F | F | F | F | purine nucleotide metabolic process | D0CAF1\_ACIB2 | NDK\_ACIBS | NDK\_AQUAE | NDK\_BURTA | NDK\_CAMJE | NDK\_HELPG | NDK\_MYXXA | NDK\_NEIG2 | V5VIC4\_ACIBA | GO:0022604 | 0.0234324499030381 | 145/6188 | 2/182 | 0.93113026090472 | 1 | F | F | F | F | regulation of cell morphogenesis | DACC\_BACSU | DAC\_STRSR | GO:0008360 | 0.0234324499030381 | 145/6188 | 2/182 | 0.93113026090472 | 1 | F | F | F | F | regulation of cell shape | DACC\_BACSU | DAC\_STRSR | GO:0022603 | 0.0234324499030381 | 145/6188 | 2/182 | 0.93113026090472 | 1 | F | F | F | F | regulation of anatomical structure morphogenesis | DACC\_BACSU | DAC\_STRSR | GO:0006024 | 0.0239172592113769 | 148/6188 | 2/182 | 0.936077695672031 | 1 | F | F | F | F | glycosaminoglycan biosynthetic process | DACC\_BACSU | DAC\_STRSR | GO:0006023 | 0.0239172592113769 | 148/6188 | 2/182 | 0.936077695672031 | 1 | F | F | F | F | aminoglycan biosynthetic process | DACC\_BACSU | DAC\_STRSR | GO:0009252 | 0.0239172592113769 | 148/6188 | 2/182 | 0.936077695672031 | 1 | F | F | F | F | peptidoglycan biosynthetic process | DACC\_BACSU | DAC\_STRSR | GO:0050793 | 0.024240465416936 | 150/6188 | 2/182 | 0.939187772213501 | 1 | F | F | F | F | regulation of developmental process | DACC\_BACSU | DAC\_STRSR | GO:0072521 | 0.0759534583063995 | 470/6188 | 9/182 | 0.942323119649703 | 1 | F | F | F | F | purine-containing compound metabolic process | D0CAF1\_ACIB2 | NDK\_ACIBS | NDK\_AQUAE | NDK\_BURTA | NDK\_CAMJE | NDK\_HELPG | NDK\_MYXXA | NDK\_NEIG2 | V5VIC4\_ACIBA | GO:0050789 | 0.0405623787976729 | 251/6188 | 4/182 | 0.942435351257278 | 1 | F | F | F | F | regulation of biological process | A85B\_MYCTU | BXX\_CLOBO | DACC\_BACSU | DAC\_STRSR | GO:0009165 | 0.0764382676147382 | 473/6188 | 9/182 | 0.944974687276769 | 1 | F | F | F | F | nucleotide biosynthetic process | D0CAF1\_ACIB2 | NDK\_ACIBS | NDK\_AQUAE | NDK\_BURTA | NDK\_CAMJE | NDK\_HELPG | NDK\_MYXXA | NDK\_NEIG2 | V5VIC4\_ACIBA | GO:1901293 | 0.0769230769230769 | 476/6188 | 9/182 | 0.947519634389937 | 1 | F | F | F | F | nucleoside phosphate biosynthetic process | D0CAF1\_ACIB2 | NDK\_ACIBS | NDK\_AQUAE | NDK\_BURTA | NDK\_CAMJE | NDK\_HELPG | NDK\_MYXXA | NDK\_NEIG2 | V5VIC4\_ACIBA | GO:0065008 | 0.0266645119586296 | 165/6188 | 2/182 | 0.958344799007913 | 1 | F | F | F | F | regulation of biological quality | DACC\_BACSU | DAC\_STRSR | GO:0065007 | 0.0437944408532644 | 271/6188 | 4/182 | 0.961965301870847 | 1 | F | F | F | F | biological regulation | A85B\_MYCTU | BXX\_CLOBO | DACC\_BACSU | DAC\_STRSR | GO:0044038 | 0.0273109243697479 | 169/6188 | 2/182 | 0.962387810911378 | 1 | F | F | F | F | cell wall macromolecule biosynthetic process | DACC\_BACSU | DAC\_STRSR | GO:1901137 | 0.122171945701357 | 756/6188 | 13/182 | 0.991278958266361 | 1 | F | F | F | F | carbohydrate derivative biosynthetic process | A85C\_MYCTU | D0CAF1\_ACIB2 | DACC\_BACSU | DAC\_STRSR | ESTA\_PSEAE | NDK\_ACIBS | NDK\_AQUAE | NDK\_BURTA | NDK\_CAMJE | NDK\_HELPG | NDK\_MYXXA | NDK\_NEIG2 | V5VIC4\_ACIBA | GO:0034654 | 0.0977698771816419 | 605/6188 | 9/182 | 0.994651637529841 | 1 | F | F | F | F | nucleobase-containing compound biosynthetic process | D0CAF1\_ACIB2 | NDK\_ACIBS | NDK\_AQUAE | NDK\_BURTA | NDK\_CAMJE | NDK\_HELPG | NDK\_MYXXA | NDK\_NEIG2 | V5VIC4\_ACIBA | GO:0009117 | 0.101809954751131 | 630/6188 | 9/182 | 0.996730769379855 | 1 | F | F | F | F | nucleotide metabolic process | D0CAF1\_ACIB2 | NDK\_ACIBS | NDK\_AQUAE | NDK\_BURTA | NDK\_CAMJE | NDK\_HELPG | NDK\_MYXXA | NDK\_NEIG2 | V5VIC4\_ACIBA | GO:0044255 | 0.0788623141564318 | 488/6188 | 6/182 | 0.996991200458938 | 1 | F | F | F | F | cellular lipid metabolic process | A85C\_MYCTU | ESTA\_PSEAE | NANH\_VIBCH | PHL2\_BACCE | PHLC\_STAAU | XYNC\_BACSU | GO:0006753 | 0.102617970265029 | 635/6188 | 9/182 | 0.997042388725546 | 1 | F | F | F | F | nucleoside phosphate metabolic process | D0CAF1\_ACIB2 | NDK\_ACIBS | NDK\_AQUAE | NDK\_BURTA | NDK\_CAMJE | NDK\_HELPG | NDK\_MYXXA | NDK\_NEIG2 | V5VIC4\_ACIBA | GO:0044248 | 0.135585003232062 | 839/6188 | 13/182 | 0.998027259857876 | 1 | F | F | F | F | cellular catabolic process | A5N1B8\_CLOK5 | BLAC\_BACSU | BLAC\_STRAL | D2ARB3\_STRRD | D5DH82\_PRIM3 | D9TA79\_MICAI | DRNE\_VIBCH | LYTM\_STAA8 | NANH\_VIBCH | P94458\_BACLI | Q79DR3\_ECOLX | Q81DA0\_BACCR | Q9JZ43\_NEIMB | GO:0009059 | 0.057207498383969 | 354/6188 | 3/182 | 0.998562957320008 | 1 | F | F | F | F | macromolecule biosynthetic process | DACC\_BACSU | DAC\_STRSR | Q9RHW0\_BACSP | GO:0008152 | 0.952003878474467 | 5891/6188 | 163/182 | 0.999586239531642 | 1 | F | F | F | F | metabolic process | A0A067XG64\_PARTM | A0A0B7LH44\_STREE | A0A0H2UQE4\_STRPN | A0A0H2WX20\_STAAC | A0A0M3KKW7\_STRPY | A0A160EBC2\_ACHDE | A0A182DW00\_STREE | A0A243G6Q6\_BACTU | A0A378K9X8\_LEGPN | A0A4P8ESF9\_BACSP | A0A4V8H037\_9RHOB | A0A6L7H1P2\_BACAN | A0R5R2\_MYCS2 | A1TSQ3\_ACIAC | A3DJ82\_ACET2 | A5N1B8\_CLOK5 | A85C\_MYCTU | A8FDC4\_BACP2 | AMT6\_BACS7 | AMYB\_BACCE | AMYM\_GEOSE | AMYS\_NEIPO | AMY\_BACAM | AMY\_GEOSE | B4EPS2\_BURCJ | B9MMA5\_CALBD | BLAC\_BACSU | BLAC\_STRAL | BSAP\_BACSU | BXX\_CLOBO | C7ED31\_ACET1 | CBPM\_STRAL | CDGT\_BACS0 | CDGT\_GEOSE | CHIS\_BACSU | CHIS\_STRSN | CHOD\_STRS0 | D0CAF1\_ACIB2 | D0VP31\_9BACI | D0VV09\_BACCE | D0VXY8\_STAPS | D2ARB3\_STRRD | D5DH82\_PRIM3 | D8H130\_BACAI | D9TA79\_MICAI | DACC\_BACSU | DAC\_STRSR | DRNE\_VIBCH | E4Q2A4\_CALOW | EBA1\_ELIME | EBA3\_ELIME | ENG1\_ACET2 | ESTA\_BACSU | ESTA\_PSEAE | ETA\_STAAU | ETB\_STAAU | FLS\_FERPE | G0L2Y1\_ZOBGA | G2NRC4\_STREK | G8LZE0\_ACECE | GSEA\_STAES | GUB\_BACLI | GUB\_BACSU | GUNA\_ACET2 | GUNC\_ACETH | GUNF\_RUMCH | GUNG\_RUMCH | GUNS\_ACETH | GUN\_ECOLI | H0B8D4\_9ACTN | HYSA\_STRA3 | I3P686\_BACLI | IABF\_STRAW | K0IUV6\_9ENTR | L0EGW1\_THECK | L0RUV7\_STAAU | LCHMO\_ENTFA | LIP\_BACSP | LIP\_PSEAE | LYTM\_STAA8 | NANB\_STRPN | NANH\_VIBCH | NDK\_ACIBS | NDK\_AQUAE | NDK\_BURTA | NDK\_CAMJE | NDK\_HELPG | NDK\_MYXXA | NDK\_NEIG2 | O06496\_CLOPF | O30700\_9BACI | O52754\_RHOMR | O68424\_BACFG | O69771\_9PSED | O82839\_BACSP | O83009\_SERMA | O86049\_BACFG | P84141\_PAEAU | P94458\_BACLI | PEPX\_LACHE | PGLR2\_PECPM | PHL2\_BACCE | PHLC\_STAAU | PLC\_BACCE | PLC\_BACTU | PLC\_LISMO | PLC\_STAAE | PLD\_STRAT | PLYL\_DICD3 | PLY\_BACSU | PMEA\_DICD3 | PRZN\_SERME | PTLY\_THEMA | Q09LY9\_GEOSE | Q17TM8\_ALKHA | Q21KE5\_SACD2 | Q54276\_SERMA | Q59962\_9ACTN | Q5DZ44\_ALIF1 | Q5MJ80\_SERPR | Q65GB9\_BACLD | Q65JI7\_BACLD | Q65JI8\_BACLD | Q6MNP0\_BDEBA | Q6RSN8\_ACETH | Q79DR3\_ECOLX | Q7LYT7\_PYRWO | Q7SID8\_BACIU | Q7X4S4\_BACLI | Q81DA0\_BACCR | Q82L26\_STRAW | Q88JL2\_PSEPK | Q8P6Z9\_XANCP | Q93I48\_9BACI | Q9AJF8\_ACETH | Q9AJM4\_9BACI | Q9AJS0\_ALIAC | Q9EYQ2\_9FIRM | Q9JZ43\_NEIMB | Q9KWY6\_GEOSE | Q9RC94\_9BACI | Q9REI6\_ARTSP | Q9RHW0\_BACSP | Q9XB24\_KLEPN | RHA78\_STRAW | RIPB\_MYCTU | SFAS2\_STRFR | SPLA\_STAA8 | SPLB\_STAA8 | SPLC\_STAA8 | SPLD\_STAA8 | SPLE\_STAA8 | SSL1\_STAA8 | SUBD\_BACLI | V5IRV7\_THETH | V5VIC4\_ACIBA | W5JXE0\_BACSP | W8FKE7\_BACPU | XANLY\_BACGL | XYN1\_GEOSE | XYNA\_HALH5 | XYNC\_BACSU | YKFC\_BACC1 | GO:1901135 | 0.187782805429864 | 1162/6188 | 18/182 | 0.999717500291978 | 1 | F | F | F | F | carbohydrate derivative metabolic process | A0R5R2\_MYCS2 | A85C\_MYCTU | D0CAF1\_ACIB2 | DACC\_BACSU | DAC\_STRSR | ESTA\_PSEAE | LCHMO\_ENTFA | NANH\_VIBCH | NDK\_ACIBS | NDK\_AQUAE | NDK\_BURTA | NDK\_CAMJE | NDK\_HELPG | NDK\_MYXXA | NDK\_NEIG2 | O06496\_CLOPF | O83009\_SERMA | V5VIC4\_ACIBA | GO:0090407 | 0.122495151906917 | 758/6188 | 9/182 | 0.999786703148935 | 1 | F | F | F | F | organophosphate biosynthetic process | D0CAF1\_ACIB2 | NDK\_ACIBS | NDK\_AQUAE | NDK\_BURTA | NDK\_CAMJE | NDK\_HELPG | NDK\_MYXXA | NDK\_NEIG2 | V5VIC4\_ACIBA | GO:0008610 | 0.0709437621202327 | 439/6188 | 2/182 | 0.999980961523547 | 1 | F | F | F | F | lipid biosynthetic process | A85C\_MYCTU | ESTA\_PSEAE | GO:1901564 | 0.471234647705236 | 2916/6188 | 59/182 | 0.999984212256213 | 1 | F | F | F | F | organonitrogen compound metabolic process | A0A0H2WX20\_STAAC | A0A0M3KKW7\_STRPY | A0A4V8H037\_9RHOB | A0R5R2\_MYCS2 | A5N1B8\_CLOK5 | BLAC\_BACSU | BLAC\_STRAL | BSAP\_BACSU | BXX\_CLOBO | CBPM\_STRAL | D0CAF1\_ACIB2 | D0VXY8\_STAPS | D2ARB3\_STRRD | D5DH82\_PRIM3 | D8H130\_BACAI | D9TA79\_MICAI | DACC\_BACSU | DAC\_STRSR | ETA\_STAAU | ETB\_STAAU | FLS\_FERPE | GSEA\_STAES | L0RUV7\_STAAU | LCHMO\_ENTFA | LYTM\_STAA8 | NANH\_VIBCH | NDK\_ACIBS | NDK\_AQUAE | NDK\_BURTA | NDK\_CAMJE | NDK\_HELPG | NDK\_MYXXA | NDK\_NEIG2 | O06496\_CLOPF | O68424\_BACFG | O69771\_9PSED | O83009\_SERMA | O86049\_BACFG | P94458\_BACLI | PEPX\_LACHE | PHL2\_BACCE | PHLC\_STAAU | PRZN\_SERME | Q5MJ80\_SERPR | Q79DR3\_ECOLX | Q81DA0\_BACCR | RIPB\_MYCTU | SFAS2\_STRFR | SPLA\_STAA8 | SPLB\_STAA8 | SPLC\_STAA8 | SPLD\_STAA8 | SPLE\_STAA8 | SSL1\_STAA8 | SUBD\_BACLI | V5IRV7\_THETH | V5VIC4\_ACIBA | XYNC\_BACSU | YKFC\_BACC1 | GO:0055086 | 0.140433096315449 | 869/6188 | 9/182 | 0.999984369223997 | 1 | F | F | F | F | nucleobase-containing small molecule metabolic process | D0CAF1\_ACIB2 | NDK\_ACIBS | NDK\_AQUAE | NDK\_BURTA | NDK\_CAMJE | NDK\_HELPG | NDK\_MYXXA | NDK\_NEIG2 | V5VIC4\_ACIBA | GO:0019637 | 0.156916612798966 | 971/6188 | 9/182 | 0.999998809845163 | 1 | F | F | F | F | organophosphate metabolic process | D0CAF1\_ACIB2 | NDK\_ACIBS | NDK\_AQUAE | NDK\_BURTA | NDK\_CAMJE | NDK\_HELPG | NDK\_MYXXA | NDK\_NEIG2 | V5VIC4\_ACIBA | GO:0019438 | 0.167582417582418 | 1037/6188 | 9/182 | 0.999999792525869 | 1 | F | F | F | F | aromatic compound biosynthetic process | D0CAF1\_ACIB2 | NDK\_ACIBS | NDK\_AQUAE | NDK\_BURTA | NDK\_CAMJE | NDK\_HELPG | NDK\_MYXXA | NDK\_NEIG2 | V5VIC4\_ACIBA | GO:0018130 | 0.169036845507434 | 1046/6188 | 9/182 | 0.999999837263378 | 1 | F | F | F | F | heterocycle biosynthetic process | D0CAF1\_ACIB2 | NDK\_ACIBS | NDK\_AQUAE | NDK\_BURTA | NDK\_CAMJE | NDK\_HELPG | NDK\_MYXXA | NDK\_NEIG2 | V5VIC4\_ACIBA | GO:0044282 | 0.096961861667744 | 600/6188 | 2/182 | 0.999999862589704 | 1 | F | F | F | F | small molecule catabolic process | CHOD\_STRS0 | Q9JZ43\_NEIMB | GO:0006796 | 0.192469295410472 | 1191/6188 | 11/182 | 0.999999940092779 | 1 | F | F | F | F | phosphate-containing compound metabolic process | A0A378K9X8\_LEGPN | D0CAF1\_ACIB2 | NDK\_ACIBS | NDK\_AQUAE | NDK\_BURTA | NDK\_CAMJE | NDK\_HELPG | NDK\_MYXXA | NDK\_NEIG2 | Q6MNP0\_BDEBA | V5VIC4\_ACIBA | GO:0006793 | 0.204751131221719 | 1267/6188 | 11/182 | 0.999999992537889 | 1 | F | F | F | F | phosphorus metabolic process | A0A378K9X8\_LEGPN | D0CAF1\_ACIB2 | NDK\_ACIBS | NDK\_AQUAE | NDK\_BURTA | NDK\_CAMJE | NDK\_HELPG | NDK\_MYXXA | NDK\_NEIG2 | Q6MNP0\_BDEBA | V5VIC4\_ACIBA | GO:1901362 | 0.188267614738203 | 1165/6188 | 9/182 | 0.999999994068989 | 1 | F | F | F | F | organic cyclic compound biosynthetic process | D0CAF1\_ACIB2 | NDK\_ACIBS | NDK\_AQUAE | NDK\_BURTA | NDK\_CAMJE | NDK\_HELPG | NDK\_MYXXA | NDK\_NEIG2 | V5VIC4\_ACIBA | GO:0044271 | 0.188590820943762 | 1167/6188 | 9/182 | 0.999999994399044 | 1 | F | F | F | F | cellular nitrogen compound biosynthetic process | D0CAF1\_ACIB2 | NDK\_ACIBS | NDK\_AQUAE | NDK\_BURTA | NDK\_CAMJE | NDK\_HELPG | NDK\_MYXXA | NDK\_NEIG2 | V5VIC4\_ACIBA | GO:0006139 | 0.206851971557854 | 1280/6188 | 10/182 | 0.999999998915467 | 1 | F | F | F | F | nucleobase-containing compound metabolic process | D0CAF1\_ACIB2 | DRNE\_VIBCH | NDK\_ACIBS | NDK\_AQUAE | NDK\_BURTA | NDK\_CAMJE | NDK\_HELPG | NDK\_MYXXA | NDK\_NEIG2 | V5VIC4\_ACIBA | GO:0006807 | 0.560116354234001 | 3466/6188 | 60/182 | 0.999999999936779 | 1 | F | F | F | F | nitrogen compound metabolic process | A0A0H2WX20\_STAAC | A0A0M3KKW7\_STRPY | A0A4V8H037\_9RHOB | A0R5R2\_MYCS2 | A5N1B8\_CLOK5 | BLAC\_BACSU | BLAC\_STRAL | BSAP\_BACSU | BXX\_CLOBO | CBPM\_STRAL | D0CAF1\_ACIB2 | D0VXY8\_STAPS | D2ARB3\_STRRD | D5DH82\_PRIM3 | D8H130\_BACAI | D9TA79\_MICAI | DACC\_BACSU | DAC\_STRSR | DRNE\_VIBCH | ETA\_STAAU | ETB\_STAAU | FLS\_FERPE | GSEA\_STAES | L0RUV7\_STAAU | LCHMO\_ENTFA | LYTM\_STAA8 | NANH\_VIBCH | NDK\_ACIBS | NDK\_AQUAE | NDK\_BURTA | NDK\_CAMJE | NDK\_HELPG | NDK\_MYXXA | NDK\_NEIG2 | O06496\_CLOPF | O68424\_BACFG | O69771\_9PSED | O83009\_SERMA | O86049\_BACFG | P94458\_BACLI | PEPX\_LACHE | PHL2\_BACCE | PHLC\_STAAU | PRZN\_SERME | Q5MJ80\_SERPR | Q79DR3\_ECOLX | Q81DA0\_BACCR | RIPB\_MYCTU | SFAS2\_STRFR | SPLA\_STAA8 | SPLB\_STAA8 | SPLC\_STAA8 | SPLD\_STAA8 | SPLE\_STAA8 | SSL1\_STAA8 | SUBD\_BACLI | V5IRV7\_THETH | V5VIC4\_ACIBA | XYNC\_BACSU | YKFC\_BACC1 | GO:0046483 | 0.307369101486749 | 1902/6188 | 19/182 | 0.999999999991292 | 1 | F | F | F | F | heterocycle metabolic process | A5N1B8\_CLOK5 | BLAC\_BACSU | BLAC\_STRAL | D0CAF1\_ACIB2 | D2ARB3\_STRRD | D5DH82\_PRIM3 | D9TA79\_MICAI | DRNE\_VIBCH | NDK\_ACIBS | NDK\_AQUAE | NDK\_BURTA | NDK\_CAMJE | NDK\_HELPG | NDK\_MYXXA | NDK\_NEIG2 | P94458\_BACLI | Q79DR3\_ECOLX | Q81DA0\_BACCR | V5VIC4\_ACIBA | GO:0009987 | 0.748545572074984 | 4632/6188 | 46/182 | 0.999999999999459 | 1 | F | F | F | F | cellular process | A0A378K9X8\_LEGPN | A5N1B8\_CLOK5 | A85C\_MYCTU | BLAC\_BACSU | BLAC\_STRAL | C3K8K1\_PSEFS | CBPM\_STRAL | D0CAF1\_ACIB2 | D0VWY2\_PSEFS | D2ARB3\_STRRD | D5DH82\_PRIM3 | D9TA79\_MICAI | DACC\_BACSU | DAC\_STRSR | DRNE\_VIBCH | ENG1\_ACET2 | ESTA\_PSEAE | LIP\_PSEAE | LYTM\_STAA8 | NANH\_VIBCH | NDK\_ACIBS | NDK\_AQUAE | NDK\_BURTA | NDK\_CAMJE | NDK\_HELPG | NDK\_MYXXA | NDK\_NEIG2 | P94458\_BACLI | PGLR2\_PECPM | PHL2\_BACCE | PHLC1\_CLOPE | PHLC\_BACCE | PHLC\_CLOP1 | PHLC\_CLOPF | PHLC\_STAAU | PMEA\_DICD3 | PTLY\_THEMA | Q6MNP0\_BDEBA | Q79DR3\_ECOLX | Q81DA0\_BACCR | Q9JZ43\_NEIMB | Q9RHW0\_BACSP | RIPB\_MYCTU | V5VIC4\_ACIBA | XYNC\_BACSU | YKFC\_BACC1 | GO:0044249 | 0.40530058177117 | 2508/6188 | 14/182 | 0.999999999999813 | 1 | F | F | F | F | cellular biosynthetic process | A85C\_MYCTU | D0CAF1\_ACIB2 | DACC\_BACSU | DAC\_STRSR | ESTA\_PSEAE | NDK\_ACIBS | NDK\_AQUAE | NDK\_BURTA | NDK\_CAMJE | NDK\_HELPG | NDK\_MYXXA | NDK\_NEIG2 | Q9RHW0\_BACSP | V5VIC4\_ACIBA | GO:1901576 | 0.427440206851972 | 2645/6188 | 14/182 | 1 | 1 | F | F | F | F | organic substance biosynthetic process | A85C\_MYCTU | D0CAF1\_ACIB2 | DACC\_BACSU | DAC\_STRSR | ESTA\_PSEAE | NDK\_ACIBS | NDK\_AQUAE | NDK\_BURTA | NDK\_CAMJE | NDK\_HELPG | NDK\_MYXXA | NDK\_NEIG2 | Q9RHW0\_BACSP | V5VIC4\_ACIBA | GO:0044281 | 0.542501616031028 | 3357/6188 | 21/182 | 1 | 1 | F | F | F | F | small molecule metabolic process | A5N1B8\_CLOK5 | BLAC\_BACSU | BLAC\_STRAL | CHOD\_STRS0 | D0CAF1\_ACIB2 | D2ARB3\_STRRD | D5DH82\_PRIM3 | D9TA79\_MICAI | IABF\_STRAW | NDK\_ACIBS | NDK\_AQUAE | NDK\_BURTA | NDK\_CAMJE | NDK\_HELPG | NDK\_MYXXA | NDK\_NEIG2 | P94458\_BACLI | Q79DR3\_ECOLX | Q81DA0\_BACCR | Q9JZ43\_NEIMB | V5VIC4\_ACIBA | GO:0034641 | 0.305753070458953 | 1892/6188 | 10/182 | 1 | 1 | F | F | F | F | cellular nitrogen compound metabolic process | D0CAF1\_ACIB2 | DRNE\_VIBCH | NDK\_ACIBS | NDK\_AQUAE | NDK\_BURTA | NDK\_CAMJE | NDK\_HELPG | NDK\_MYXXA | NDK\_NEIG2 | V5VIC4\_ACIBA | GO:1901360 | 0.342598577892696 | 2120/6188 | 20/182 | 1 | 1 | F | F | F | F | organic cyclic compound metabolic process | A5N1B8\_CLOK5 | BLAC\_BACSU | BLAC\_STRAL | CHOD\_STRS0 | D0CAF1\_ACIB2 | D2ARB3\_STRRD | D5DH82\_PRIM3 | D9TA79\_MICAI | DRNE\_VIBCH | NDK\_ACIBS | NDK\_AQUAE | NDK\_BURTA | NDK\_CAMJE | NDK\_HELPG | NDK\_MYXXA | NDK\_NEIG2 | P94458\_BACLI | Q79DR3\_ECOLX | Q81DA0\_BACCR | V5VIC4\_ACIBA | GO:0008150 | 1 | 6188/6188 | 182/182 | 1 | 1 | F | F | F | F | biological\_process | A0A067XG64\_PARTM | A0A0B7LH44\_STREE | A0A0H2UQE4\_STRPN | A0A0H2WX20\_STAAC | A0A0M3KKU6\_ERWAE | A0A0M3KKW7\_STRPY | A0A160EBC2\_ACHDE | A0A182DW00\_STREE | A0A243G6Q6\_BACTU | A0A378K9X8\_LEGPN | A0A4P8ESF9\_BACSP | A0A4V8H037\_9RHOB | A0A6L7H1P2\_BACAN | A0R5R2\_MYCS2 | A1TSQ3\_ACIAC | A3DJ82\_ACET2 | A5N1B8\_CLOK5 | A85B\_MYCTU | A85C\_MYCTU | A8FDC4\_BACP2 | AMT6\_BACS7 | AMYB\_BACCE | AMYM\_GEOSE | AMYS\_NEIPO | AMY\_BACAM | AMY\_GEOSE | B2IF78\_BEII9 | B2VCC3\_ERWT9 | B4EPS2\_BURCJ | B9MMA5\_CALBD | BLAC\_BACSU | BLAC\_STRAL | BSAP\_BACSU | BXX\_CLOBO | C3K8K1\_PSEFS | C7ED31\_ACET1 | CBPM\_STRAL | CDGT\_BACS0 | CDGT\_GEOSE | CHIS\_BACSU | CHIS\_STRSN | CHOD\_STRS0 | D0CAF1\_ACIB2 | D0VP31\_9BACI | D0VV09\_BACCE | D0VWY2\_PSEFS | D0VXY8\_STAPS | D2ARB3\_STRRD | D5DC07\_PRIM3 | D5DH82\_PRIM3 | D8H130\_BACAI | D8J9C2\_HALJB | D9TA79\_MICAI | DACC\_BACSU | DAC\_STRSR | DRNE\_VIBCH | E4Q2A4\_CALOW | EBA1\_ELIME | EBA3\_ELIME | EFEN\_BACSU | ENG1\_ACET2 | ESTA\_BACSU | ESTA\_PSEAE | ETA\_STAAU | ETB\_STAAU | FLS\_FERPE | G0L2Y1\_ZOBGA | G2NRC4\_STREK | G8LZE0\_ACECE | GSEA\_STAES | GUB\_BACLI | GUB\_BACSU | GUNA\_ACET2 | GUNC\_ACETH | GUNF\_RUMCH | GUNG\_RUMCH | GUNS\_ACETH | GUN\_ECOLI | H0B8D4\_9ACTN | HCPB\_HELPY | HCPC\_HELPY | HYSA\_STRA3 | I3P686\_BACLI | IABF\_STRAW | K0IUV6\_9ENTR | L0EGW1\_THECK | L0RUV7\_STAAU | LCHMO\_ENTFA | LIP\_BACSP | LIP\_PSEAE | LYTM\_STAA8 | NANB\_STRPN | NANH\_VIBCH | NDK\_ACIBS | NDK\_AQUAE | NDK\_BURTA | NDK\_CAMJE | NDK\_HELPG | NDK\_MYXXA | NDK\_NEIG2 | O06496\_CLOPF | O30700\_9BACI | O52754\_RHOMR | O68424\_BACFG | O69771\_9PSED | O82839\_BACSP | O83009\_SERMA | O86049\_BACFG | P84141\_PAEAU | P94458\_BACLI | PEPX\_LACHE | PGLR2\_PECPM | PHL2\_BACCE | PHLC1\_CLOPE | PHLC\_BACCE | PHLC\_CLOP1 | PHLC\_CLOPF | PHLC\_STAAU | PLC\_BACCE | PLC\_BACTU | PLC\_LISMO | PLC\_STAAE | PLD\_STRAT | PLYL\_DICD3 | PLY\_BACSU | PMEA\_DICD3 | PRZN\_SERME | PTLY\_THEMA | Q09LY9\_GEOSE | Q17TM8\_ALKHA | Q21KE5\_SACD2 | Q54276\_SERMA | Q59962\_9ACTN | Q5DZ44\_ALIF1 | Q5MJ80\_SERPR | Q65GB9\_BACLD | Q65JI7\_BACLD | Q65JI8\_BACLD | Q6MNP0\_BDEBA | Q6RSN8\_ACETH | Q79DR3\_ECOLX | Q7LYT7\_PYRWO | Q7SID8\_BACIU | Q7X4S4\_BACLI | Q81DA0\_BACCR | Q82L26\_STRAW | Q88JL2\_PSEPK | Q8P6Z9\_XANCP | Q93I48\_9BACI | Q9AJF8\_ACETH | Q9AJM4\_9BACI | Q9AJS0\_ALIAC | Q9EYQ2\_9FIRM | Q9JZ43\_NEIMB | Q9KWY6\_GEOSE | Q9RC94\_9BACI | Q9REI6\_ARTSP | Q9RHW0\_BACSP | Q9RKQ2\_STRCO | Q9XB24\_KLEPN | Q9ZBW9\_STRCO | RHA78\_STRAW | RIPB\_MYCTU | SACB\_BACSU | SACB\_GLUDI | SFAS2\_STRFR | SPLA\_STAA8 | SPLB\_STAA8 | SPLC\_STAA8 | SPLD\_STAA8 | SPLE\_STAA8 | SSL1\_STAA8 | SUBD\_BACLI | V5IRV7\_THETH | V5VIC4\_ACIBA | W5JXE0\_BACSP | W8FKE7\_BACPU | XANLY\_BACGL | XYN1\_GEOSE | XYNA\_HALH5 | XYNC\_BACSU | YKFC\_BACC1 | GO:0006725 | 0.312540400775695 | 1934/6188 | 10/182 | 1 | 1 | F | F | F | F | cellular aromatic compound metabolic process | D0CAF1\_ACIB2 | DRNE\_VIBCH | NDK\_ACIBS | NDK\_AQUAE | NDK\_BURTA | NDK\_CAMJE | NDK\_HELPG | NDK\_MYXXA | NDK\_NEIG2 | V5VIC4\_ACIBA | GO:0009058 | 0.454751131221719 | 2814/6188 | 14/182 | 1 | 1 | F | F | F | F | biosynthetic process | A85C\_MYCTU | D0CAF1\_ACIB2 | DACC\_BACSU | DAC\_STRSR | ESTA\_PSEAE | NDK\_ACIBS | NDK\_AQUAE | NDK\_BURTA | NDK\_CAMJE | NDK\_HELPG | NDK\_MYXXA | NDK\_NEIG2 | Q9RHW0\_BACSP | V5VIC4\_ACIBA | GO:0044237 | 0.719457013574661 | 4452/6188 | 32/182 | 1 | 1 | F | F | F | F | cellular metabolic process | A0A378K9X8\_LEGPN | A5N1B8\_CLOK5 | A85C\_MYCTU | BLAC\_BACSU | BLAC\_STRAL | D0CAF1\_ACIB2 | D2ARB3\_STRRD | D5DH82\_PRIM3 | D9TA79\_MICAI | DACC\_BACSU | DAC\_STRSR | DRNE\_VIBCH | ESTA\_PSEAE | LYTM\_STAA8 | NANH\_VIBCH | NDK\_ACIBS | NDK\_AQUAE | NDK\_BURTA | NDK\_CAMJE | NDK\_HELPG | NDK\_MYXXA | NDK\_NEIG2 | P94458\_BACLI | PHL2\_BACCE | PHLC\_STAAU | Q6MNP0\_BDEBA | Q79DR3\_ECOLX | Q81DA0\_BACCR | Q9JZ43\_NEIMB | Q9RHW0\_BACSP | V5VIC4\_ACIBA | XYNC\_BACSU | GO:1901566 | 0.290562378797673 | 1798/6188 | 11/182 | 1 | 1 | F | F | F | F | organonitrogen compound biosynthetic process | D0CAF1\_ACIB2 | DACC\_BACSU | DAC\_STRSR | NDK\_ACIBS | NDK\_AQUAE | NDK\_BURTA | NDK\_CAMJE | NDK\_HELPG | NDK\_MYXXA | NDK\_NEIG2 | V5VIC4\_ACIBA | GO:0010468 | 0.00614091790562379 | 38/6188 | 1/182 | NA | NA | NA | NA | NA | NA | regulation of gene expression | A85B\_MYCTU | GO:0006706 | 0.00226244343891403 | 14/6188 | 1/182 | NA | NA | NA | NA | NA | NA | steroid catabolic process | CHOD\_STRS0 | GO:0009306 | 0.00048480930833872 | 3/6188 | 1/182 | NA | NA | NA | NA | NA | NA | protein secretion | LIP\_PSEAE | GO:0046903 | 0.00048480930833872 | 3/6188 | 1/182 | NA | NA | NA | NA | NA | NA | secretion | LIP\_PSEAE | GO:0007049 | 0.0103425985778927 | 64/6188 | 1/182 | NA | NA | NA | NA | NA | NA | cell cycle | DACC\_BACSU | GO:0006869 | 0.000161603102779573 | 1/6188 | 1/182 | NA | NA | NA | NA | NA | NA | lipid transport | A85C\_MYCTU | GO:0010557 | 0.00113122171945701 | 7/6188 | 1/182 | NA | NA | NA | NA | NA | NA | positive regulation of macromolecule biosynthetic process | A85B\_MYCTU | GO:0005982 | 0.00048480930833872 | 3/6188 | 1/182 | NA | NA | NA | NA | NA | NA | starch metabolic process | AMT6\_BACS7 | GO:0046466 | 0.000646412411118293 | 4/6188 | 1/182 | NA | NA | NA | NA | NA | NA | membrane lipid catabolic process | NANH\_VIBCH | GO:0043207 | 0.012120232708468 | 75/6188 | 1/182 | NA | NA | NA | NA | NA | NA | response to external biotic stimulus | A85B\_MYCTU | GO:0031323 | 0.00775694893341952 | 48/6188 | 1/182 | NA | NA | NA | NA | NA | NA | regulation of cellular metabolic process | A85B\_MYCTU | GO:0046929 | 0.000161603102779573 | 1/6188 | 1/182 | NA | NA | NA | NA | NA | NA | negative regulation of neurotransmitter secretion | BXX\_CLOBO | GO:1902652 | 0.00614091790562379 | 38/6188 | 1/182 | NA | NA | NA | NA | NA | NA | secondary alcohol metabolic process | CHOD\_STRS0 | GO:0051604 | 0.00323206205559147 | 20/6188 | 1/182 | NA | NA | NA | NA | NA | NA | protein maturation | FLS\_FERPE | GO:0051048 | 0.000323206205559147 | 2/6188 | 1/182 | NA | NA | NA | NA | NA | NA | negative regulation of secretion | BXX\_CLOBO | GO:0016051 | 0.0381383322559793 | 236/6188 | 1/182 | NA | NA | NA | NA | NA | NA | carbohydrate biosynthetic process | Q9RHW0\_BACSP | GO:0098754 | 0.00711053652230123 | 44/6188 | 1/182 | NA | NA | NA | NA | NA | NA | detoxification | Q9JZ43\_NEIMB | GO:0044403 | 0.00662572721396251 | 41/6188 | 1/182 | NA | NA | NA | NA | NA | NA | biological process involved in symbiotic interaction | A85B\_MYCTU | GO:0071978 | 0.00048480930833872 | 3/6188 | 1/182 | NA | NA | NA | NA | NA | NA | bacterial-type flagellum-dependent swarming motility | ESTA\_PSEAE | GO:0009607 | 0.0138978668390433 | 86/6188 | 1/182 | NA | NA | NA | NA | NA | NA | response to biotic stimulus | A85B\_MYCTU | GO:0009313 | 0.00404007756948933 | 25/6188 | 1/182 | NA | NA | NA | NA | NA | NA | oligosaccharide catabolic process | NANH\_VIBCH | GO:0008203 | 0.00210084033613445 | 13/6188 | 1/182 | NA | NA | NA | NA | NA | NA | cholesterol metabolic process | CHOD\_STRS0 | GO:0006687 | 0.00048480930833872 | 3/6188 | 1/182 | NA | NA | NA | NA | NA | NA | glycosphingolipid metabolic process | NANH\_VIBCH | GO:0140975 | 0.000323206205559147 | 2/6188 | 1/182 | NA | NA | NA | NA | NA | NA | disruption of cellular anatomical structure in another organism | PLYL\_DICD3 | GO:0009891 | 0.00129282482223659 | 8/6188 | 1/182 | NA | NA | NA | NA | NA | NA | positive regulation of biosynthetic process | A85B\_MYCTU | GO:0071692 | 0.00048480930833872 | 3/6188 | 1/182 | NA | NA | NA | NA | NA | NA | protein localization to extracellular region | LIP\_PSEAE | GO:0006308 | 0.000323206205559147 | 2/6188 | 1/182 | NA | NA | NA | NA | NA | NA | DNA catabolic process | DRNE\_VIBCH | GO:0019321 | 0.0106658047834518 | 66/6188 | 1/182 | NA | NA | NA | NA | NA | NA | pentose metabolic process | IABF\_STRAW | GO:0045862 | 0.00048480930833872 | 3/6188 | 1/182 | NA | NA | NA | NA | NA | NA | positive regulation of proteolysis | A85B\_MYCTU | GO:0045184 | 0.00177763413057531 | 11/6188 | 1/182 | NA | NA | NA | NA | NA | NA | establishment of protein localization | LIP\_PSEAE | GO:0051171 | 0.00614091790562379 | 38/6188 | 1/182 | NA | NA | NA | NA | NA | NA | regulation of nitrogen compound metabolic process | A85B\_MYCTU | GO:0140352 | 0.00048480930833872 | 3/6188 | 1/182 | NA | NA | NA | NA | NA | NA | export from cell | LIP\_PSEAE | GO:0048519 | 0.00468648998060763 | 29/6188 | 1/182 | NA | NA | NA | NA | NA | NA | negative regulation of biological process | BXX\_CLOBO | GO:0032940 | 0.00048480930833872 | 3/6188 | 1/182 | NA | NA | NA | NA | NA | NA | secretion by cell | LIP\_PSEAE | GO:0044179 | 0.000161603102779573 | 1/6188 | 1/182 | NA | NA | NA | NA | NA | NA | hemolysis in another organism | PHLC\_CLOP1 | GO:0042710 | 0.00096961861667744 | 6/6188 | 1/182 | NA | NA | NA | NA | NA | NA | biofilm formation | ESTA\_PSEAE | GO:0046479 | 0.00048480930833872 | 3/6188 | 1/182 | NA | NA | NA | NA | NA | NA | glycosphingolipid catabolic process | NANH\_VIBCH | GO:0010954 | 0.00048480930833872 | 3/6188 | 1/182 | NA | NA | NA | NA | NA | NA | positive regulation of protein processing | A85B\_MYCTU | GO:0030162 | 0.00048480930833872 | 3/6188 | 1/182 | NA | NA | NA | NA | NA | NA | regulation of proteolysis | A85B\_MYCTU | GO:0075136 | 0.00371687136393019 | 23/6188 | 1/182 | NA | NA | NA | NA | NA | NA | response to host | A85B\_MYCTU | GO:0051589 | 0.000161603102779573 | 1/6188 | 1/182 | NA | NA | NA | NA | NA | NA | negative regulation of neurotransmitter transport | BXX\_CLOBO | GO:0019439 | 0.0536522301228184 | 332/6188 | 1/182 | NA | NA | NA | NA | NA | NA | aromatic compound catabolic process | DRNE\_VIBCH | GO:0032502 | 0.00517129928894635 | 32/6188 | 1/182 | NA | NA | NA | NA | NA | NA | developmental process | SUBD\_BACLI | GO:0052173 | 0.00371687136393019 | 23/6188 | 1/182 | NA | NA | NA | NA | NA | NA | response to defenses of other organism | A85B\_MYCTU | GO:1903531 | 0.000323206205559147 | 2/6188 | 1/182 | NA | NA | NA | NA | NA | NA | negative regulation of secretion by cell | BXX\_CLOBO | GO:0042545 | 0.000646412411118293 | 4/6188 | 1/182 | NA | NA | NA | NA | NA | NA | cell wall modification | PMEA\_DICD3 | GO:0051715 | 0.000161603102779573 | 1/6188 | 1/182 | NA | NA | NA | NA | NA | NA | cytolysis in another organism | PHLC\_CLOP1 | GO:0051641 | 0.00177763413057531 | 11/6188 | 1/182 | NA | NA | NA | NA | NA | NA | cellular localization | LIP\_PSEAE | GO:0050804 | 0.000161603102779573 | 1/6188 | 1/182 | NA | NA | NA | NA | NA | NA | modulation of chemical synaptic transmission | BXX\_CLOBO | GO:0051703 | 0.00096961861667744 | 6/6188 | 1/182 | NA | NA | NA | NA | NA | NA | biological process involved in intraspecies interaction between organisms | ESTA\_PSEAE | GO:0022607 | 0.0119586296056884 | 74/6188 | 1/182 | NA | NA | NA | NA | NA | NA | cellular component assembly | A85C\_MYCTU | GO:0034655 | 0.023109243697479 | 143/6188 | 1/182 | NA | NA | NA | NA | NA | NA | nucleobase-containing compound catabolic process | DRNE\_VIBCH | GO:0023051 | 0.00048480930833872 | 3/6188 | 1/182 | NA | NA | NA | NA | NA | NA | regulation of signaling | BXX\_CLOBO | GO:0001539 | 0.00048480930833872 | 3/6188 | 1/182 | NA | NA | NA | NA | NA | NA | cilium or flagellum-dependent cell motility | ESTA\_PSEAE | GO:1903317 | 0.00048480930833872 | 3/6188 | 1/182 | NA | NA | NA | NA | NA | NA | regulation of protein maturation | A85B\_MYCTU | GO:0051707 | 0.012120232708468 | 75/6188 | 1/182 | NA | NA | NA | NA | NA | NA | response to other organism | A85B\_MYCTU | GO:0051246 | 0.00129282482223659 | 8/6188 | 1/182 | NA | NA | NA | NA | NA | NA | regulation of protein metabolic process | A85B\_MYCTU | GO:0044270 | 0.0366839043309632 | 227/6188 | 1/182 | NA | NA | NA | NA | NA | NA | cellular nitrogen compound catabolic process | DRNE\_VIBCH | GO:0048522 | 0.00210084033613445 | 13/6188 | 1/182 | NA | NA | NA | NA | NA | NA | positive regulation of cellular process | A85B\_MYCTU | GO:0052200 | 0.00371687136393019 | 23/6188 | 1/182 | NA | NA | NA | NA | NA | NA | response to host defenses | A85B\_MYCTU | GO:0006066 | 0.0281189398836458 | 174/6188 | 1/182 | NA | NA | NA | NA | NA | NA | alcohol metabolic process | CHOD\_STRS0 | GO:0016127 | 0.00210084033613445 | 13/6188 | 1/182 | NA | NA | NA | NA | NA | NA | sterol catabolic process | CHOD\_STRS0 | GO:0030149 | 0.000646412411118293 | 4/6188 | 1/182 | NA | NA | NA | NA | NA | NA | sphingolipid catabolic process | NANH\_VIBCH | GO:0005996 | 0.0421784098254687 | 261/6188 | 1/182 | NA | NA | NA | NA | NA | NA | monosaccharide metabolic process | IABF\_STRAW | GO:0015628 | 0.000161603102779573 | 1/6188 | 1/182 | NA | NA | NA | NA | NA | NA | protein secretion by the type II secretion system | LIP\_PSEAE | GO:0015031 | 0.00177763413057531 | 11/6188 | 1/182 | NA | NA | NA | NA | NA | NA | protein transport | LIP\_PSEAE | GO:0110095 | 0.00161603102779573 | 10/6188 | 1/182 | NA | NA | NA | NA | NA | NA | cellular detoxification of aldehyde | Q9JZ43\_NEIMB | GO:0006689 | 0.000323206205559147 | 2/6188 | 1/182 | NA | NA | NA | NA | NA | NA | ganglioside catabolic process | NANH\_VIBCH | GO:0098743 | 0.00096961861667744 | 6/6188 | 1/182 | NA | NA | NA | NA | NA | NA | cell aggregation | ESTA\_PSEAE | GO:0051046 | 0.000323206205559147 | 2/6188 | 1/182 | NA | NA | NA | NA | NA | NA | regulation of secretion | BXX\_CLOBO | GO:0040009 | 0.000161603102779573 | 1/6188 | 1/182 | NA | NA | NA | NA | NA | NA | regulation of growth rate | A85B\_MYCTU | GO:0019835 | 0.000161603102779573 | 1/6188 | 1/182 | NA | NA | NA | NA | NA | NA | cytolysis | PHLC\_CLOP1 | GO:0001573 | 0.000323206205559147 | 2/6188 | 1/182 | NA | NA | NA | NA | NA | NA | ganglioside metabolic process | NANH\_VIBCH | GO:0043952 | 0.000646412411118293 | 4/6188 | 1/182 | NA | NA | NA | NA | NA | NA | protein transport by the Sec complex | LIP\_PSEAE | GO:1903319 | 0.00048480930833872 | 3/6188 | 1/182 | NA | NA | NA | NA | NA | NA | positive regulation of protein maturation | A85B\_MYCTU | GO:0052572 | 0.00371687136393019 | 23/6188 | 1/182 | NA | NA | NA | NA | NA | NA | response to host immune response | A85B\_MYCTU | GO:0019566 | 0.00339366515837104 | 21/6188 | 1/182 | NA | NA | NA | NA | NA | NA | arabinose metabolic process | IABF\_STRAW | GO:0046164 | 0.00937297996121525 | 58/6188 | 1/182 | NA | NA | NA | NA | NA | NA | alcohol catabolic process | CHOD\_STRS0 | GO:0046292 | 0.00210084033613445 | 13/6188 | 1/182 | NA | NA | NA | NA | NA | NA | formaldehyde metabolic process | Q9JZ43\_NEIMB | GO:0031326 | 0.00630252100840336 | 39/6188 | 1/182 | NA | NA | NA | NA | NA | NA | regulation of cellular biosynthetic process | A85B\_MYCTU | GO:0006672 | 0.000646412411118293 | 4/6188 | 1/182 | NA | NA | NA | NA | NA | NA | ceramide metabolic process | NANH\_VIBCH | GO:0048523 | 0.00355526826115061 | 22/6188 | 1/182 | NA | NA | NA | NA | NA | NA | negative regulation of cellular process | BXX\_CLOBO | GO:0046514 | 0.000646412411118293 | 4/6188 | 1/182 | NA | NA | NA | NA | NA | NA | ceramide catabolic process | NANH\_VIBCH | GO:0071705 | 0.00193923723335488 | 12/6188 | 1/182 | NA | NA | NA | NA | NA | NA | nitrogen compound transport | LIP\_PSEAE | GO:0044010 | 0.00096961861667744 | 6/6188 | 1/182 | NA | NA | NA | NA | NA | NA | single-species biofilm formation | ESTA\_PSEAE | GO:0008104 | 0.00177763413057531 | 11/6188 | 1/182 | NA | NA | NA | NA | NA | NA | protein localization | LIP\_PSEAE | GO:0099177 | 0.000161603102779573 | 1/6188 | 1/182 | NA | NA | NA | NA | NA | NA | regulation of trans-synaptic signaling | BXX\_CLOBO | GO:1901615 | 0.0499353587588882 | 309/6188 | 1/182 | NA | NA | NA | NA | NA | NA | organic hydroxy compound metabolic process | CHOD\_STRS0 | GO:0010604 | 0.00145442792501616 | 9/6188 | 1/182 | NA | NA | NA | NA | NA | NA | positive regulation of macromolecule metabolic process | A85B\_MYCTU | GO:0045489 | 0.000161603102779573 | 1/6188 | 1/182 | NA | NA | NA | NA | NA | NA | pectin biosynthetic process | Q9RHW0\_BACSP | GO:0051588 | 0.000161603102779573 | 1/6188 | 1/182 | NA | NA | NA | NA | NA | NA | regulation of neurotransmitter transport | BXX\_CLOBO | GO:0071973 | 0.00048480930833872 | 3/6188 | 1/182 | NA | NA | NA | NA | NA | NA | bacterial-type flagellum-dependent cell motility | ESTA\_PSEAE | GO:0070727 | 0.00177763413057531 | 11/6188 | 1/182 | NA | NA | NA | NA | NA | NA | cellular macromolecule localization | LIP\_PSEAE | GO:1990748 | 0.00597931480284421 | 37/6188 | 1/182 | NA | NA | NA | NA | NA | NA | cellular detoxification | Q9JZ43\_NEIMB | GO:0060255 | 0.0072721396250808 | 45/6188 | 1/182 | NA | NA | NA | NA | NA | NA | regulation of macromolecule metabolic process | A85B\_MYCTU | GO:0046928 | 0.000161603102779573 | 1/6188 | 1/182 | NA | NA | NA | NA | NA | NA | regulation of neurotransmitter secretion | BXX\_CLOBO | GO:0030435 | 0.00323206205559147 | 20/6188 | 1/182 | NA | NA | NA | NA | NA | NA | sporulation resulting in formation of a cellular spore | SUBD\_BACLI | GO:0046373 | 0.00258564964447317 | 16/6188 | 1/182 | NA | NA | NA | NA | NA | NA | L-arabinose metabolic process | IABF\_STRAW | GO:0051051 | 0.000323206205559147 | 2/6188 | 1/182 | NA | NA | NA | NA | NA | NA | negative regulation of transport | BXX\_CLOBO | GO:1901616 | 0.0127666451195863 | 79/6188 | 1/182 | NA | NA | NA | NA | NA | NA | organic hydroxy compound catabolic process | CHOD\_STRS0 | GO:0040008 | 0.000646412411118293 | 4/6188 | 1/182 | NA | NA | NA | NA | NA | NA | regulation of growth | A85B\_MYCTU | GO:0016540 | 0.000161603102779573 | 1/6188 | 1/182 | NA | NA | NA | NA | NA | NA | protein autoprocessing | FLS\_FERPE | GO:0080090 | 0.00662572721396251 | 41/6188 | 1/182 | NA | NA | NA | NA | NA | NA | regulation of primary metabolic process | A85B\_MYCTU | GO:0048646 | 0.00323206205559147 | 20/6188 | 1/182 | NA | NA | NA | NA | NA | NA | anatomical structure formation involved in morphogenesis | SUBD\_BACLI | GO:0051247 | 0.000808015513897867 | 5/6188 | 1/182 | NA | NA | NA | NA | NA | NA | positive regulation of protein metabolic process | A85B\_MYCTU | GO:0006518 | 0.0172915319974144 | 107/6188 | 1/182 | NA | NA | NA | NA | NA | NA | peptide metabolic process | LYTM\_STAA8 | GO:0031325 | 0.00177763413057531 | 11/6188 | 1/182 | NA | NA | NA | NA | NA | NA | positive regulation of cellular metabolic process | A85B\_MYCTU | GO:0032879 | 0.000646412411118293 | 4/6188 | 1/182 | NA | NA | NA | NA | NA | NA | regulation of localization | BXX\_CLOBO | GO:0009311 | 0.0127666451195863 | 79/6188 | 1/182 | NA | NA | NA | NA | NA | NA | oligosaccharide metabolic process | NANH\_VIBCH | GO:0051173 | 0.00129282482223659 | 8/6188 | 1/182 | NA | NA | NA | NA | NA | NA | positive regulation of nitrogen compound metabolic process | A85B\_MYCTU | GO:0009889 | 0.00630252100840336 | 39/6188 | 1/182 | NA | NA | NA | NA | NA | NA | regulation of biosynthetic process | A85B\_MYCTU | GO:0033036 | 0.00177763413057531 | 11/6188 | 1/182 | NA | NA | NA | NA | NA | NA | macromolecule localization | LIP\_PSEAE | GO:0071769 | 0.000646412411118293 | 4/6188 | 1/182 | NA | NA | NA | NA | NA | NA | mycolate cell wall layer assembly | A85C\_MYCTU | GO:0071806 | 0.00129282482223659 | 8/6188 | 1/182 | NA | NA | NA | NA | NA | NA | protein transmembrane transport | LIP\_PSEAE | GO:0070613 | 0.00048480930833872 | 3/6188 | 1/182 | NA | NA | NA | NA | NA | NA | regulation of protein processing | A85B\_MYCTU | GO:0006081 | 0.0244020685197156 | 151/6188 | 1/182 | NA | NA | NA | NA | NA | NA | cellular aldehyde metabolic process | Q9JZ43\_NEIMB | GO:0035592 | 0.00048480930833872 | 3/6188 | 1/182 | NA | NA | NA | NA | NA | NA | establishment of protein localization to extracellular region | LIP\_PSEAE | GO:0051301 | 0.0105042016806723 | 65/6188 | 1/182 | NA | NA | NA | NA | NA | NA | cell division | DACC\_BACSU | GO:0044242 | 0.00694893341952165 | 43/6188 | 1/182 | NA | NA | NA | NA | NA | NA | cellular lipid catabolic process | NANH\_VIBCH | GO:0031328 | 0.00129282482223659 | 8/6188 | 1/182 | NA | NA | NA | NA | NA | NA | positive regulation of cellular biosynthetic process | A85B\_MYCTU | GO:0046185 | 0.00420168067226891 | 26/6188 | 1/182 | NA | NA | NA | NA | NA | NA | aldehyde catabolic process | Q9JZ43\_NEIMB | GO:0010756 | 0.00048480930833872 | 3/6188 | 1/182 | NA | NA | NA | NA | NA | NA | positive regulation of plasminogen activation | A85B\_MYCTU | GO:0009893 | 0.00177763413057531 | 11/6188 | 1/182 | NA | NA | NA | NA | NA | NA | positive regulation of metabolic process | A85B\_MYCTU | GO:0016125 | 0.0024240465416936 | 15/6188 | 1/182 | NA | NA | NA | NA | NA | NA | sterol metabolic process | CHOD\_STRS0 | GO:0016485 | 0.000646412411118293 | 4/6188 | 1/182 | NA | NA | NA | NA | NA | NA | protein processing | FLS\_FERPE | GO:0043934 | 0.00323206205559147 | 20/6188 | 1/182 | NA | NA | NA | NA | NA | NA | sporulation | SUBD\_BACLI | GO:0010628 | 0.00113122171945701 | 7/6188 | 1/182 | NA | NA | NA | NA | NA | NA | positive regulation of gene expression | A85B\_MYCTU | GO:0006259 | 0.0234324499030381 | 145/6188 | 1/182 | NA | NA | NA | NA | NA | NA | DNA metabolic process | DRNE\_VIBCH | GO:0051701 | 0.00565610859728507 | 35/6188 | 1/182 | NA | NA | NA | NA | NA | NA | biological process involved in interaction with host | A85B\_MYCTU | GO:0010646 | 0.00048480930833872 | 3/6188 | 1/182 | NA | NA | NA | NA | NA | NA | regulation of cell communication | BXX\_CLOBO | GO:1903530 | 0.000323206205559147 | 2/6188 | 1/182 | NA | NA | NA | NA | NA | NA | regulation of secretion by cell | BXX\_CLOBO | GO:0043171 | 0.00161603102779573 | 10/6188 | 1/182 | NA | NA | NA | NA | NA | NA | peptide catabolic process | LYTM\_STAA8 | GO:0019377 | 0.00048480930833872 | 3/6188 | 1/182 | NA | NA | NA | NA | NA | NA | glycolipid catabolic process | NANH\_VIBCH | GO:0005983 | 0.00048480930833872 | 3/6188 | 1/182 | NA | NA | NA | NA | NA | NA | starch catabolic process | AMT6\_BACS7 | GO:0010755 | 0.00048480930833872 | 3/6188 | 1/182 | NA | NA | NA | NA | NA | NA | regulation of plasminogen activation | A85B\_MYCTU | GO:0048518 | 0.0024240465416936 | 15/6188 | 1/182 | NA | NA | NA | NA | NA | NA | positive regulation of biological process | A85B\_MYCTU | GO:0097588 | 0.00048480930833872 | 3/6188 | 1/182 | NA | NA | NA | NA | NA | NA | archaeal or bacterial-type flagellum-dependent cell motility | ESTA\_PSEAE | GO:0000271 | 0.0174531351001939 | 108/6188 | 1/182 | NA | NA | NA | NA | NA | NA | polysaccharide biosynthetic process | Q9RHW0\_BACSP | GO:0044278 | 0.000161603102779573 | 1/6188 | 1/182 | NA | NA | NA | NA | NA | NA | disruption of cell wall in another organism | PLYL\_DICD3 | GO:0046294 | 0.00161603102779573 | 10/6188 | 1/182 | NA | NA | NA | NA | NA | NA | formaldehyde catabolic process | Q9JZ43\_NEIMB | GO:0090304 | 0.0702973497091144 | 435/6188 | 1/182 | NA | NA | NA | NA | NA | NA | nucleic acid metabolic process | DRNE\_VIBCH | GO:0051049 | 0.000646412411118293 | 4/6188 | 1/182 | NA | NA | NA | NA | NA | NA | regulation of transport | BXX\_CLOBO | GO:0008202 | 0.00533290239172592 | 33/6188 | 1/182 | NA | NA | NA | NA | NA | NA | steroid metabolic process | CHOD\_STRS0 | GO:0098630 | 0.00096961861667744 | 6/6188 | 1/182 | NA | NA | NA | NA | NA | NA | aggregation of unicellular organisms | ESTA\_PSEAE | GO:0098776 | 0.000161603102779573 | 1/6188 | 1/182 | NA | NA | NA | NA | NA | NA | protein transport across the cell outer membrane | LIP\_PSEAE | GO:0048870 | 0.00048480930833872 | 3/6188 | 1/182 | NA | NA | NA | NA | NA | NA | cell motility | ESTA\_PSEAE | GO:0010556 | 0.00614091790562379 | 38/6188 | 1/182 | NA | NA | NA | NA | NA | NA | regulation of macromolecule biosynthetic process | A85B\_MYCTU | GO:0006707 | 0.00210084033613445 | 13/6188 | 1/182 | NA | NA | NA | NA | NA | NA | cholesterol catabolic process | CHOD\_STRS0 | GO:0019222 | 0.00824175824175824 | 51/6188 | 1/182 | NA | NA | NA | NA | NA | NA | regulation of metabolic process | A85B\_MYCTU |

Total number of genes: 6188   
Total number of Study genes: 182   
Total number of Study gene GMRG terms (pop non-singletons): 341 (325)   
FDR Threshold *P*-values: [10% = 0.0432], [5% = 0.0195], [1% = 0.00339], [0.5% = 0.00161]   
Genes with GMRG information: 182   
Genes with no GMRG information:   

These are:
