# Supplementary material for: Cellular location shapes quaternary structure of enzymes
Source: Nat Commun. 2024 Oct 1;15:8505. doi: 10.1038/s41467-024-52662-2 (PMC11445431; doi:10.1038/s41467-024-52662-2)
Supplement: Supplementary file 5 — Supplementary Data 2 [file 41467_2024_52662_MOESM5_ESM.zip › GO/Euk_F/output.F.txt.html]

 GeneMerge Output - output.F.txt

### GeneMerge v1.4

### Castillo-Davis, C.I. 2015. GeneMerge v1.4 - post-genomic data analysis

Output File Name: output.F.txt   
Gene Association File: terms.txt   
Description File: descriptions.txt   
Population File: prot\_all.txt   
Study File: prot\_test.txt  
Custom FDR: 0.5%  

|  |  |  |  |  |  |  |  |  |  |  |  |
| --- | --- | --- | --- | --- | --- | --- | --- | --- | --- | --- | --- |
| **GMRG Term** | **Pop Frequency** | **Pop Fraction** | **Study Fraction** | ***P*-value** | **Bon. Corr. *P*-value** | **10% FDR** | **5% FDR** | **1% FDR** | **0.5% FDR** | **Description** | **Contributing genes** |
| GO:0016787 | 0.329991872121376 | 1218/3691 | 498/641 | 6.23961727112999e-147 | 2.50208652572313e-144 | T | T | T | T | hydrolase activity | A0A059U759\_9PEZI | A0A060N399\_9PLEO | A0A068FT77\_9PEZI | A0A075B5G4\_HUMIN | A0A075B5H6\_TRIHA | A0A075C6T6\_RHIMI | A0A086SY89\_ACRC1 | A0A086T6R4\_ACRC1 | A0A088T0J9\_GEOCN | A0A0J5Q413\_ASPFM | A0A0M3KKZ6\_RHIMI | A0A0M3KKZ8\_RHIMI | A0A0R4I979\_BRABE | A0A0S2GKZ1\_9APHY | A0A173N065\_EISFE | A0A1D3S5H0\_FUSOX | A0A1L6CE30\_9EURO | A0A1L8D5Z7\_BOTAT | A0A1L9WG58\_ASPA1 | A0A1S6YJF3\_MALCI | A0A1S9DRB1\_ASPOZ | A0A2H5BN17\_TALPI | A0A2N1LTK3\_TRIHA | A0A2U8ZTY7\_RHIZD | A0A2Z4HIN9\_9EURO | A0A384E148\_NICBE | A0A3B6UEQ2\_RHIMI | A0A3B6UEQ6\_EISFE | A0A3G2C3I4\_9EURO | A0A3G4RHU4\_9PEZI | A0A482LWB1\_OSTFU | A0A5J6BJN2\_MALCI | A0A6F8Z6Y2\_BOMMO | A0A6M9BP13\_9EURO | A0A6P6YAT6\_DERPT | A0A7S6G7I6\_9PEZI | A0A856TAI5\_9BASI | A0NFU8\_ANOGA | A1E266\_9PEZI | A1HA\_LOXIN | A1HB2\_LOXIN | A2QIR3\_ASPNC | A2QZC8\_ASPNC | A2TBB4\_9ASCO | A2TM14\_HEVBR | A311\_LOXLA | A4GX63\_TOXGO | A5AB48\_ASPNC | A6PZ97\_SALSA | A6YRT4\_9PEZI | A7KMF0\_9CAEN | A8NI40\_COPC7 | A9LI60\_BIOOC | A9ZSX9\_9BRYO | ABFB\_ASPKW | ADA2\_HUMAN | ADPG2\_ARATH | AGAL\_HUMAN | AGAL\_ORYSJ | AMPS2\_LITPI | AMY1A\_HUMAN | AMY1\_HORVU | AMY1\_ORYSJ | AMYA1\_ASPOR | AMYG\_SACFI | AMY\_ORYLA | ANAG\_HUMAN | ANG1\_BOVIN | ANG2\_MOUSE | ANG3\_MOUSE | ANG4\_MOUSE | ANGI\_MOUSE | AOAH\_MOUSE | ASAH1\_BALAS | ASM3A\_HUMAN | ASM3A\_MOUSE | ATLE\_CYCAE | AXE1\_ASPAW | AXE2\_TALPU | AXHA2\_EMENI | B1Q4V2\_HERER | B4F320\_LIMPO | B7X9Z0\_COPCI | B7X9Z2\_COPCI | B9TU22\_GADMO | BGALA\_ASPNC | BGALA\_ASPOR | BGALA\_PENSQ | BGL1\_ASPAC | BGLA\_ASPFU | BGLA\_ASPOR | BGLR\_HUMAN | C3VEV9\_PENCN | C7YSL3\_FUSV7 | CARP1\_CANAL | CARP2\_CANAX | CARP\_RHIPU | CATD\_RAT | CATH\_HUMAN | CATLL\_FASHE | CBHB\_ASPFU | CBHRE\_GEOS1 | CBPA1\_PIG | CBPD\_LOPSP | CBPN\_HUMAN | CDA\_COLLN | CDA\_EMENI | CEL2A\_PIG | CFAD\_MOUSE | CHI1\_COCPS | CHI2\_HORVU | CHI2\_ORYSJ | CHI33\_TRIHA | CHI42\_TRIHA | CHI4\_CRYJA | CHIA\_HUMAN | CHIC\_ARATH | CHIC\_SECCE | CHIL3\_MOUSE | CHIT\_PUNGR | CHLY\_HEVBR | CHYM\_CAMDR | COGS\_HYPLI | CONB\_CANEN | CUCM1\_CUCME | CUTI1\_ASPOR | CUTI1\_COLGL | CUTI1\_FUSVN | CUTI1\_HYPJR | CYSP\_BLOTA | D0QF43\_9HELO | D1M8S7\_HEVBR | D6XHE1\_TRYB2 | D9MWI4\_9ASPA | DDN1\_BOVIN | DEXT\_TALMI | DNAS1\_HUMAN | DNSL3\_HUMAN | DPP2\_HUMAN | E0A7J0\_YARLL | E0CX04\_MOMBA | E0XN39\_9EURO | E13B\_HORVU | E13C\_MUSAC | E3VTL0\_9ASPA | E5D0X5\_SCHOC | E7FH77\_DANRE | E9G5J5\_DAPPU | ECP\_HUMAN | EGFB2\_MOUSE | EGLB\_ASPNG | ENDO2\_ARATH | ENG1\_RHIMI | ENPP2\_HUMAN | ENPP2\_RAT | ERVB\_TABDI | EST6\_DROME | EXG1\_CANAL | EXG1\_YEAST | F0ZJZ1\_DICPU | F1CYZ0\_TALFU | F2Z7L1\_9ANNE | F6MIW5\_WHEAT | FAEA\_ASPNG | FAEB1\_ASPOR | FAEB2\_ASPOR | FUCO\_HUMAN | G0RVK1\_HYPJQ | G2Q665\_MYCTT | G2QVH2\_THETT | G3I1H5\_CRIGR | G3JPF7\_CORMM | G3YAL0\_ASPNA | G3YFQ1\_ASPNA | G8GLP2\_LENED | G9NTY1\_HYPAI | GALNS\_HUMAN | GANA\_ASPAC | GANA\_EMENI | GANA\_HUMIN | GBA1\_HUMAN | GCE2\_MYCTT | GCE\_CERUI | GCE\_HYPJQ | GGH\_HUMAN | GH7B\_LIMQU | GRAA\_HUMAN | GRAC\_MOUSE | GRAK\_HUMAN | GRASS\_DROME | GUB2\_HORVU | GUN2\_HYPJE | GUN6\_HUMIN | GUN7\_HYPJQ | GUNC\_FUSOX | GUN\_ASPAC | GUN\_CRYAT | GUN\_MYTED | GUX1\_HUMGT | GUX1\_HYPJE | GUX1\_TRIHA | GUX2\_HYPJE | H1AE14\_PHACH | HE12\_DANRE | HEXC\_OSTFU | HYAL1\_HUMAN | I1SB18\_VIPAE | I2FI81\_EISFE | I3RY46\_TRIHA | IDUA\_HUMAN | INU2\_ASPFI | INUE\_ASPAW | INV\_SCHOC | IPUA\_ASPNG | J7LCB0\_DEIAC | J9UN47\_GIBZA | K7CID1\_PANTR | K9L8F3\_MALCI | KLK10\_HUMAN | KLK1\_HUMAN | KLK2\_HORSE | KLK2\_HUMAN | KLK7\_HUMAN | KLK7\_MOUSE | KLK8\_MOUSE | L7SVX1\_RHIMI | LALBA\_BOVIN | LAPA\_ASPOR | LGMN\_MOUSE | LICH\_HUMAN | LIP1\_DIURU | LIP2\_DIURU | LIP2\_GEOCN | LIP3\_DIURU | LIPA\_MOEAP | LIPB\_PSEA2 | LIPG\_CANLF | LIPG\_HUMAN | LIPP\_HORSE | LIPR1\_CANLF | LIPR1\_HUMAN | LIPR2\_HUMAN | LIPR2\_RAT | LIP\_THELA | LYG\_STRCA | LYS1\_MUSDO | LYSC1\_ANAPL | LYSC1\_CANLF | LYSC1\_HORSE | LYSC2\_BOVIN | LYSC2\_ONCMY | LYSC\_COTJA | LYSC\_EQUAS | LYSC\_NUMME | LYSC\_OPIHO | LYSC\_PELSI | LYS\_BOMMO | LYS\_RUDPH | M2RAI8\_CERS8 | M9TI89\_RHIPU | MAN12\_PENCI | MAN4\_SOLLC | MANA\_ASPNC | MANA\_CANEN | MANA\_CRYAT | MANA\_HYPJR | MANA\_MYTED | MANA\_PODAN | MANBA\_MOUSE | MCPT2\_RAT | MDLA\_PENCA | MDLA\_PENCY | MEL1\_YEASX | MMP1\_PIG | NADA\_APLCA | NADA\_ASPFU | NAGAB\_CHICK | NAGAB\_HUMAN | NANL\_MACDE | NTP1\_TOXGO | NTP2\_TOXGO | NUP1\_PENCI | NUS1\_ASPOR | O00095\_HYPJE | O04358\_IRIHO | O44049\_TRYRA | O74705\_ASPNG | O77044\_9NEOP | O81100\_SOLLC | O81934\_CANEN | O97389\_HELAM | P79074\_9AGAR | PA1\_VESBA | PA21B\_BOVIN | PA21B\_PIG | PA2A1\_BUNCE | PA2A1\_ECHCA | PA2A1\_NAJAT | PA2A1\_OPHHA | PA2A2\_NAJNA | PA2A2\_OPHHA | PA2A2\_TROCA | PA2A4\_NAJSG | PA2A5\_TRIST | PA2A7\_GLOHA | PA2A\_BOTJR | PA2A\_CROAT | PA2A\_DEIAC | PA2A\_GLOHA | PA2A\_NAJAT | PA2B1\_AGKPI | PA2B2\_BOTJR | PA2B2\_PROFL | PA2B3\_BOTAS | PA2B3\_BUNCE | PA2B5\_BUNCE | PA2B5\_NOTSC | PA2BA\_VIPAA | PA2BB\_GLOHA | PA2BB\_PSEAU | PA2BC\_VIPAA | PA2BD\_CRODU | PA2B\_BUNCE | PA2B\_NOTSC | PA2GA\_HUMAN | PA2GE\_HUMAN | PA2GX\_HUMAN | PA2H1\_AGKCL | PA2H1\_BOTBZ | PA2H1\_BOTJR | PA2H1\_BOTMO | PA2H1\_BOTPI | PA2H2\_BOTAS | PA2H2\_BOTMO | PA2H2\_BOTPI | PA2H2\_CERGO | PA2H3\_BOTPI | PA2HB\_AGKPI | PA2HB\_OXYSC | PA2HH\_TRIST | PA2HS\_ECHCA | PA2H\_BOTPA | PA2H\_DEIAC | PA2H\_PROMB | PA2N\_GLOHA | PA2\_APIME | PAG15\_HUMAN | PCP\_HUMAN | PDE\_NAJAT | PEPA\_ASPPH | PGLR1\_ASPAC | PGLR1\_ASPNG | PGLR\_GIBFU | PGPSA\_DROME | PGRP1\_CAMDR | PHAZ\_TALFU | PHYA\_ASPFU | PHYA\_ASPNG | PHYB\_ASPAW | PLA22\_ORYSJ | PME\_DAUCA | PME\_SITOR | PPA5\_HUMAN | PPA5\_PIG | PPA5\_RAT | PPAF1\_HOLDI | PPAF1\_IPOBA | PPAF\_PHAVU | PPAP\_RAT | PPT1\_BOVIN | PPT1\_HUMAN | PPT2\_HUMAN | PRS57\_HUMAN | PRTN3\_HUMAN | Q02321\_PHACH | Q07524\_TROMA | Q0KFV0\_SOLLC | Q12715\_HYPJE | Q2QEH4\_SAPOF | Q2U8V9\_ASPOR | Q2Z1W1\_PHACH | Q43576\_TOBAC | Q45U61\_ASPNG | Q4AE59\_OSTFU | Q4WP32\_ASPFU | Q50KB2\_PHACH | Q55FE6\_DICDI | Q588B8\_CRYJA | Q5WRG2\_RAT | Q69G21\_TENMO | Q6ED33\_ASPNG | Q6NY42\_DANRE | Q6R7Z5\_9TRYP | Q6S5M9\_9ASCO | Q6VAY1\_9PEZI | Q6WER3\_GIBZA | Q6WSR8\_PICAB | Q70C53\_SOLTU | Q70SY0\_HYPJE | Q7LHI2\_PHACH | Q7LIJ0\_PHACH | Q7LST4\_PENEN | Q7RWP2\_NEUCR | Q7X9A9\_CAMSI | Q7XZV5\_NICGU | Q7YXL2\_TENMO | Q86RS6\_MANSE | Q874E9\_9TREE | Q8H0C9\_VIGUN | Q8J0K6\_MELAO | Q8J0K8\_MELAO | Q8NJY6\_9HYPO | Q8T0W7\_9NEOP | Q8TFL9\_TALEM | Q8TG26\_THEAU | Q8TGI8\_TALEM | Q8VX11\_LUPLU | Q92456\_HYPJE | Q92458\_HYPJE | Q93X60\_CICIN | Q94BW3\_CINCA | Q95KP4\_HORSE | Q95V66\_PENVA | Q9FUH3\_VIGUS | Q9GPG0\_MANSE | Q9LYJ5\_ARATH | Q9M7C7\_CALSE | Q9P8F7\_YARLL | Q9SSV1\_NICGU | Q9STC1\_GRALE | Q9XEI3\_HORVV | RENI\_RAT | RHA1\_ASPAC | RHGA\_ASPAC | RIP0\_DIACA | RIP1\_BRYDI | RIP1\_HORVU | RIP1\_MOMCH | RIP1\_PHYAM | RIP2\_PHYAM | RIP3\_MOMCH | RIPA\_PHYAM | RIPG\_SURMU | RIPL1\_PHYDI | RIPL2\_PHYDI | RIPT\_TRIKI | RNAS1\_BISBI | RNAS4\_HUMAN | RNAS4\_PIG | RNAS6\_HUMAN | RNF1\_GIBFU | RNLE\_SOLLC | RNRH\_RHINI | RNS11\_NICAL | RNS1B\_RAT | RNS3\_PYRPY | RNSL3\_DANRE | RNS\_BOVIN | RNT2\_HUMAN | S6BC01\_PSEA2 | S7Q6I2\_GLOTA | S7ZIW0\_PENO1 | SIA\_ASPFU | TLP\_PRUAV | TPP1\_HUMAN | TRFL\_BUBBU | TRFL\_HORSE | TRY1\_GADMO | TRY3\_SALSA | TRYB2\_HUMAN | V5NTD\_NAJAT | VM11\_BOTMO | VM12\_CROAD | VM1A3\_DEIAC | VM1BI\_BOTMO | VM1T1\_PROMU | VM1T2\_PROFL | VSPP\_DEIAC | VSPSX\_GLOSA | W0T408\_KLUMD | W4KMP1\_HETIT | W8P1L2\_TALEM | W8VR85\_TALPI | X0BTD8\_FUSOX | X0M5X0\_FUSOX | XGHA\_ASPTU | XTH34\_POPPZ | XYLA\_ASPNC | XYN1\_HYPJR | XYN2\_HYPJR | XYN3\_ASPKW | XYN3\_HYPJQ | XYNA\_FUSO4 | XYNA\_PENSI | XYNA\_THEAU | XYNA\_THELA | XYNC\_ASPNC | XYND\_EMENI |
 GO:0004553 | 0.0853427255486318 | 315/3691 | 228/641 | 1.47071102417046e-116 | 5.89755120692355e-114 | T | T | T | T | hydrolase activity, hydrolyzing O-glycosyl compounds | A0A059U759\_9PEZI | A0A068FT77\_9PEZI | A0A075B5H6\_TRIHA | A0A075C6T6\_RHIMI | A0A086SY89\_ACRC1 | A0A086T6R4\_ACRC1 | A0A088T0J9\_GEOCN | A0A0J5Q413\_ASPFM | A0A0M3KKZ6\_RHIMI | A0A0M3KKZ8\_RHIMI | A0A0S2GKZ1\_9APHY | A0A173N065\_EISFE | A0A1L6CE30\_9EURO | A0A1L9WG58\_ASPA1 | A0A1S9DRB1\_ASPOZ | A0A2H5BN17\_TALPI | A0A2N1LTK3\_TRIHA | A0A2U8ZTY7\_RHIZD | A0A2Z4HIN9\_9EURO | A0A384E148\_NICBE | A0A3B6UEQ2\_RHIMI | A0A3B6UEQ6\_EISFE | A0A3G2C3I4\_9EURO | A0A3G4RHU4\_9PEZI | A0A482LWB1\_OSTFU | A0A5J6BJN2\_MALCI | A0A6F8Z6Y2\_BOMMO | A0A6M9BP13\_9EURO | A0A7S6G7I6\_9PEZI | A0A856TAI5\_9BASI | A1E266\_9PEZI | A2TM14\_HEVBR | A5AB48\_ASPNC | A6PZ97\_SALSA | A6YRT4\_9PEZI | A7KMF0\_9CAEN | A8NI40\_COPC7 | A9LI60\_BIOOC | A9ZSX9\_9BRYO | ABFB\_ASPKW | ADPG2\_ARATH | AGAL\_HUMAN | AGAL\_ORYSJ | AMY1A\_HUMAN | AMY1\_HORVU | AMY1\_ORYSJ | AMYA1\_ASPOR | AMYG\_SACFI | AMY\_ORYLA | ANAG\_HUMAN | AXHA2\_EMENI | B7X9Z0\_COPCI | B7X9Z2\_COPCI | B9TU22\_GADMO | BGALA\_ASPNC | BGALA\_ASPOR | BGALA\_PENSQ | BGL1\_ASPAC | BGLA\_ASPFU | BGLA\_ASPOR | BGLR\_HUMAN | C3VEV9\_PENCN | C7YSL3\_FUSV7 | CBHB\_ASPFU | CBHRE\_GEOS1 | CHI1\_COCPS | CHI2\_HORVU | CHI2\_ORYSJ | CHI33\_TRIHA | CHI42\_TRIHA | CHI4\_CRYJA | CHIA\_HUMAN | CHIC\_ARATH | CHIC\_SECCE | CHIL3\_MOUSE | CHIT\_PUNGR | CHLY\_HEVBR | D0QF43\_9HELO | D1M8S7\_HEVBR | DEXT\_TALMI | E0XN39\_9EURO | E13B\_HORVU | E13C\_MUSAC | E5D0X5\_SCHOC | E9G5J5\_DAPPU | EGLB\_ASPNG | ENG1\_RHIMI | EXG1\_CANAL | EXG1\_YEAST | F0ZJZ1\_DICPU | F1CYZ0\_TALFU | F2Z7L1\_9ANNE | FUCO\_HUMAN | G0RVK1\_HYPJQ | G2Q665\_MYCTT | G2QVH2\_THETT | G3JPF7\_CORMM | G3YFQ1\_ASPNA | G8GLP2\_LENED | G9NTY1\_HYPAI | GANA\_ASPAC | GANA\_EMENI | GANA\_HUMIN | GBA1\_HUMAN | GH7B\_LIMQU | GUB2\_HORVU | GUN2\_HYPJE | GUN6\_HUMIN | GUN7\_HYPJQ | GUNC\_FUSOX | GUN\_ASPAC | GUN\_CRYAT | GUN\_MYTED | GUX1\_HUMGT | GUX1\_HYPJE | GUX1\_TRIHA | GUX2\_HYPJE | H1AE14\_PHACH | HEXC\_OSTFU | HYAL1\_HUMAN | I2FI81\_EISFE | I3RY46\_TRIHA | IDUA\_HUMAN | INU2\_ASPFI | INUE\_ASPAW | INV\_SCHOC | IPUA\_ASPNG | J9UN47\_GIBZA | K9L8F3\_MALCI | L7SVX1\_RHIMI | LALBA\_BOVIN | LYG\_STRCA | LYS1\_MUSDO | LYSC1\_ANAPL | LYSC1\_CANLF | LYSC1\_HORSE | LYSC2\_BOVIN | LYSC2\_ONCMY | LYSC\_COTJA | LYSC\_EQUAS | LYSC\_NUMME | LYSC\_OPIHO | LYSC\_PELSI | LYS\_BOMMO | LYS\_RUDPH | M2RAI8\_CERS8 | M9TI89\_RHIPU | MAN12\_PENCI | MAN4\_SOLLC | MANA\_ASPNC | MANA\_CANEN | MANA\_CRYAT | MANA\_HYPJR | MANA\_MYTED | MANA\_PODAN | MANBA\_MOUSE | MEL1\_YEASX | NAGAB\_CHICK | NAGAB\_HUMAN | NANL\_MACDE | O00095\_HYPJE | O44049\_TRYRA | O74705\_ASPNG | O77044\_9NEOP | O81100\_SOLLC | O81934\_CANEN | P79074\_9AGAR | PGLR1\_ASPAC | PGLR1\_ASPNG | PGLR\_GIBFU | Q02321\_PHACH | Q07524\_TROMA | Q12715\_HYPJE | Q2U8V9\_ASPOR | Q2Z1W1\_PHACH | Q43576\_TOBAC | Q4AE59\_OSTFU | Q4WP32\_ASPFU | Q50KB2\_PHACH | Q55FE6\_DICDI | Q588B8\_CRYJA | Q6VAY1\_9PEZI | Q6WSR8\_PICAB | Q70C53\_SOLTU | Q70SY0\_HYPJE | Q7LHI2\_PHACH | Q7LIJ0\_PHACH | Q7RWP2\_NEUCR | Q7X9A9\_CAMSI | Q8H0C9\_VIGUN | Q8J0K6\_MELAO | Q8J0K8\_MELAO | Q8NJY6\_9HYPO | Q8T0W7\_9NEOP | Q8TFL9\_TALEM | Q8TG26\_THEAU | Q8TGI8\_TALEM | Q92456\_HYPJE | Q92458\_HYPJE | Q93X60\_CICIN | Q95V66\_PENVA | Q9FUH3\_VIGUS | Q9LYJ5\_ARATH | Q9STC1\_GRALE | Q9XEI3\_HORVV | RHGA\_ASPAC | S7Q6I2\_GLOTA | S7ZIW0\_PENO1 | SIA\_ASPFU | TLP\_PRUAV | W0T408\_KLUMD | W4KMP1\_HETIT | W8P1L2\_TALEM | W8VR85\_TALPI | X0M5X0\_FUSOX | XGHA\_ASPTU | XTH34\_POPPZ | XYLA\_ASPNC | XYN1\_HYPJR | XYN2\_HYPJR | XYN3\_ASPKW | XYN3\_HYPJQ | XYNA\_FUSO4 | XYNA\_PENSI | XYNA\_THEAU | XYNA\_THELA | XYNC\_ASPNC | XYND\_EMENI | GO:0016798 | 0.100514765646166 | 371/3691 | 248/641 | 2.91768455413683e-115 | 1.16999150620887e-112 | T | T | T | T | hydrolase activity, acting on glycosyl bonds | A0A059U759\_9PEZI | A0A068FT77\_9PEZI | A0A075B5H6\_TRIHA | A0A075C6T6\_RHIMI | A0A086SY89\_ACRC1 | A0A086T6R4\_ACRC1 | A0A088T0J9\_GEOCN | A0A0J5Q413\_ASPFM | A0A0M3KKZ6\_RHIMI | A0A0M3KKZ8\_RHIMI | A0A0S2GKZ1\_9APHY | A0A173N065\_EISFE | A0A1L6CE30\_9EURO | A0A1L9WG58\_ASPA1 | A0A1S9DRB1\_ASPOZ | A0A2H5BN17\_TALPI | A0A2N1LTK3\_TRIHA | A0A2U8ZTY7\_RHIZD | A0A2Z4HIN9\_9EURO | A0A384E148\_NICBE | A0A3B6UEQ2\_RHIMI | A0A3B6UEQ6\_EISFE | A0A3G2C3I4\_9EURO | A0A3G4RHU4\_9PEZI | A0A482LWB1\_OSTFU | A0A5J6BJN2\_MALCI | A0A6F8Z6Y2\_BOMMO | A0A6M9BP13\_9EURO | A0A7S6G7I6\_9PEZI | A0A856TAI5\_9BASI | A1E266\_9PEZI | A2TM14\_HEVBR | A5AB48\_ASPNC | A6PZ97\_SALSA | A6YRT4\_9PEZI | A7KMF0\_9CAEN | A8NI40\_COPC7 | A9LI60\_BIOOC | A9ZSX9\_9BRYO | ABFB\_ASPKW | ADPG2\_ARATH | AGAL\_HUMAN | AGAL\_ORYSJ | AMY1A\_HUMAN | AMY1\_HORVU | AMY1\_ORYSJ | AMYA1\_ASPOR | AMYG\_SACFI | AMY\_ORYLA | ANAG\_HUMAN | AXHA2\_EMENI | B7X9Z0\_COPCI | B7X9Z2\_COPCI | B9TU22\_GADMO | BGALA\_ASPNC | BGALA\_ASPOR | BGALA\_PENSQ | BGL1\_ASPAC | BGLA\_ASPFU | BGLA\_ASPOR | BGLR\_HUMAN | C3VEV9\_PENCN | C7YSL3\_FUSV7 | CBHB\_ASPFU | CBHRE\_GEOS1 | CHI1\_COCPS | CHI2\_HORVU | CHI2\_ORYSJ | CHI33\_TRIHA | CHI42\_TRIHA | CHI4\_CRYJA | CHIA\_HUMAN | CHIC\_ARATH | CHIC\_SECCE | CHIL3\_MOUSE | CHIT\_PUNGR | CHLY\_HEVBR | D0QF43\_9HELO | D1M8S7\_HEVBR | D9MWI4\_9ASPA | DEXT\_TALMI | E0CX04\_MOMBA | E0XN39\_9EURO | E13B\_HORVU | E13C\_MUSAC | E3VTL0\_9ASPA | E5D0X5\_SCHOC | E9G5J5\_DAPPU | EGLB\_ASPNG | ENG1\_RHIMI | EXG1\_CANAL | EXG1\_YEAST | F0ZJZ1\_DICPU | F1CYZ0\_TALFU | F2Z7L1\_9ANNE | FUCO\_HUMAN | G0RVK1\_HYPJQ | G2Q665\_MYCTT | G2QVH2\_THETT | G3JPF7\_CORMM | G3YFQ1\_ASPNA | G8GLP2\_LENED | G9NTY1\_HYPAI | GANA\_ASPAC | GANA\_EMENI | GANA\_HUMIN | GBA1\_HUMAN | GH7B\_LIMQU | GUB2\_HORVU | GUN2\_HYPJE | GUN6\_HUMIN | GUN7\_HYPJQ | GUNC\_FUSOX | GUN\_ASPAC | GUN\_CRYAT | GUN\_MYTED | GUX1\_HUMGT | GUX1\_HYPJE | GUX1\_TRIHA | GUX2\_HYPJE | H1AE14\_PHACH | HEXC\_OSTFU | HYAL1\_HUMAN | I2FI81\_EISFE | I3RY46\_TRIHA | IDUA\_HUMAN | INU2\_ASPFI | INUE\_ASPAW | INV\_SCHOC | IPUA\_ASPNG | J9UN47\_GIBZA | K9L8F3\_MALCI | L7SVX1\_RHIMI | LALBA\_BOVIN | LYG\_STRCA | LYS1\_MUSDO | LYSC1\_ANAPL | LYSC1\_CANLF | LYSC1\_HORSE | LYSC2\_BOVIN | LYSC2\_ONCMY | LYSC\_COTJA | LYSC\_EQUAS | LYSC\_NUMME | LYSC\_OPIHO | LYSC\_PELSI | LYS\_BOMMO | LYS\_RUDPH | M2RAI8\_CERS8 | M9TI89\_RHIPU | MAN12\_PENCI | MAN4\_SOLLC | MANA\_ASPNC | MANA\_CANEN | MANA\_CRYAT | MANA\_HYPJR | MANA\_MYTED | MANA\_PODAN | MANBA\_MOUSE | MEL1\_YEASX | NADA\_APLCA | NADA\_ASPFU | NAGAB\_CHICK | NAGAB\_HUMAN | NANL\_MACDE | O00095\_HYPJE | O04358\_IRIHO | O44049\_TRYRA | O74705\_ASPNG | O77044\_9NEOP | O81100\_SOLLC | O81934\_CANEN | P79074\_9AGAR | PGLR1\_ASPAC | PGLR1\_ASPNG | PGLR\_GIBFU | Q02321\_PHACH | Q07524\_TROMA | Q12715\_HYPJE | Q2QEH4\_SAPOF | Q2U8V9\_ASPOR | Q2Z1W1\_PHACH | Q43576\_TOBAC | Q4AE59\_OSTFU | Q4WP32\_ASPFU | Q50KB2\_PHACH | Q55FE6\_DICDI | Q588B8\_CRYJA | Q6VAY1\_9PEZI | Q6WSR8\_PICAB | Q70C53\_SOLTU | Q70SY0\_HYPJE | Q7LHI2\_PHACH | Q7LIJ0\_PHACH | Q7RWP2\_NEUCR | Q7X9A9\_CAMSI | Q8H0C9\_VIGUN | Q8J0K6\_MELAO | Q8J0K8\_MELAO | Q8NJY6\_9HYPO | Q8T0W7\_9NEOP | Q8TFL9\_TALEM | Q8TG26\_THEAU | Q8TGI8\_TALEM | Q92456\_HYPJE | Q92458\_HYPJE | Q93X60\_CICIN | Q94BW3\_CINCA | Q95V66\_PENVA | Q9FUH3\_VIGUS | Q9LYJ5\_ARATH | Q9STC1\_GRALE | Q9XEI3\_HORVV | RHGA\_ASPAC | RIP0\_DIACA | RIP1\_BRYDI | RIP1\_HORVU | RIP1\_MOMCH | RIP1\_PHYAM | RIP2\_PHYAM | RIP3\_MOMCH | RIPA\_PHYAM | RIPG\_SURMU | RIPL1\_PHYDI | RIPL2\_PHYDI | RIPT\_TRIKI | S7Q6I2\_GLOTA | S7ZIW0\_PENO1 | SIA\_ASPFU | TLP\_PRUAV | W0T408\_KLUMD | W4KMP1\_HETIT | W8P1L2\_TALEM | W8VR85\_TALPI | X0M5X0\_FUSOX | XGHA\_ASPTU | XTH34\_POPPZ | XYLA\_ASPNC | XYN1\_HYPJR | XYN2\_HYPJR | XYN3\_ASPKW | XYN3\_HYPJQ | XYNA\_FUSO4 | XYNA\_PENSI | XYNA\_THEAU | XYNA\_THELA | XYNC\_ASPNC | XYND\_EMENI | GO:0052689 | 0.0436196152804118 | 161/3691 | 108/641 | 7.1016768967591e-47 | 2.8477724356004e-44 | T | T | T | T | carboxylic ester hydrolase activity | A0A060N399\_9PLEO | A0A075B5G4\_HUMIN | A0A1D3S5H0\_FUSOX | A0A1L8D5Z7\_BOTAT | A0A1S6YJF3\_MALCI | A2QIR3\_ASPNC | AOAH\_MOUSE | AXE1\_ASPAW | AXE2\_TALPU | CUTI1\_ASPOR | CUTI1\_COLGL | CUTI1\_FUSVN | CUTI1\_HYPJR | E0A7J0\_YARLL | ENPP2\_HUMAN | ENPP2\_RAT | EST6\_DROME | FAEA\_ASPNG | FAEB1\_ASPOR | FAEB2\_ASPOR | G3YAL0\_ASPNA | GCE2\_MYCTT | GCE\_CERUI | GCE\_HYPJQ | I1SB18\_VIPAE | LICH\_HUMAN | LIP1\_DIURU | LIP2\_DIURU | LIP2\_GEOCN | LIP3\_DIURU | LIPA\_MOEAP | LIPB\_PSEA2 | LIPG\_CANLF | LIPG\_HUMAN | LIPP\_HORSE | LIPR1\_CANLF | LIPR1\_HUMAN | LIPR2\_HUMAN | LIPR2\_RAT | LIP\_THELA | PA1\_VESBA | PA21B\_BOVIN | PA21B\_PIG | PA2A1\_BUNCE | PA2A1\_ECHCA | PA2A1\_NAJAT | PA2A1\_OPHHA | PA2A2\_NAJNA | PA2A2\_OPHHA | PA2A2\_TROCA | PA2A4\_NAJSG | PA2A5\_TRIST | PA2A7\_GLOHA | PA2A\_BOTJR | PA2A\_CROAT | PA2A\_DEIAC | PA2A\_GLOHA | PA2A\_NAJAT | PA2B1\_AGKPI | PA2B2\_BOTJR | PA2B2\_PROFL | PA2B3\_BOTAS | PA2B3\_BUNCE | PA2B5\_BUNCE | PA2B5\_NOTSC | PA2BA\_VIPAA | PA2BB\_GLOHA | PA2BB\_PSEAU | PA2BC\_VIPAA | PA2BD\_CRODU | PA2B\_BUNCE | PA2B\_NOTSC | PA2GA\_HUMAN | PA2GE\_HUMAN | PA2GX\_HUMAN | PA2H1\_AGKCL | PA2H1\_BOTBZ | PA2H1\_BOTJR | PA2H1\_BOTMO | PA2H1\_BOTPI | PA2H2\_BOTAS | PA2H2\_BOTMO | PA2H2\_BOTPI | PA2H2\_CERGO | PA2H3\_BOTPI | PA2HB\_AGKPI | PA2HB\_OXYSC | PA2HH\_TRIST | PA2HS\_ECHCA | PA2H\_BOTPA | PA2H\_DEIAC | PA2H\_PROMB | PA2N\_GLOHA | PA2\_APIME | PAG15\_HUMAN | PHAZ\_TALFU | PLA22\_ORYSJ | PME\_DAUCA | PME\_SITOR | Q6S5M9\_9ASCO | Q6WER3\_GIBZA | Q7LST4\_PENEN | Q874E9\_9TREE | Q95KP4\_HORSE | Q9GPG0\_MANSE | Q9P8F7\_YARLL | S6BC01\_PSEA2 | X0BTD8\_FUSOX | GO:0016298 | 0.0292603630452452 | 108/3691 | 81/641 | 7.45210702921526e-41 | 2.98829491871532e-38 | T | T | T | T | lipase activity | A0A1L8D5Z7\_BOTAT | E0A7J0\_YARLL | ENPP2\_HUMAN | ENPP2\_RAT | I1SB18\_VIPAE | LICH\_HUMAN | LIP1\_DIURU | LIP2\_DIURU | LIP2\_GEOCN | LIP3\_DIURU | LIPA\_MOEAP | LIPB\_PSEA2 | LIPG\_CANLF | LIPG\_HUMAN | LIPP\_HORSE | LIPR1\_CANLF | LIPR1\_HUMAN | LIPR2\_HUMAN | LIPR2\_RAT | LIP\_THELA | PA1\_VESBA | PA21B\_BOVIN | PA21B\_PIG | PA2A1\_BUNCE | PA2A1\_ECHCA | PA2A1\_NAJAT | PA2A1\_OPHHA | PA2A2\_NAJNA | PA2A2\_OPHHA | PA2A2\_TROCA | PA2A4\_NAJSG | PA2A5\_TRIST | PA2A7\_GLOHA | PA2A\_BOTJR | PA2A\_CROAT | PA2A\_DEIAC | PA2A\_GLOHA | PA2A\_NAJAT | PA2B1\_AGKPI | PA2B2\_BOTJR | PA2B2\_PROFL | PA2B3\_BOTAS | PA2B3\_BUNCE | PA2B5\_BUNCE | PA2B5\_NOTSC | PA2BA\_VIPAA | PA2BB\_GLOHA | PA2BB\_PSEAU | PA2BC\_VIPAA | PA2BD\_CRODU | PA2B\_BUNCE | PA2B\_NOTSC | PA2GA\_HUMAN | PA2GE\_HUMAN | PA2GX\_HUMAN | PA2H1\_AGKCL | PA2H1\_BOTBZ | PA2H1\_BOTJR | PA2H1\_BOTMO | PA2H1\_BOTPI | PA2H2\_BOTAS | PA2H2\_BOTMO | PA2H2\_BOTPI | PA2H2\_CERGO | PA2H3\_BOTPI | PA2HB\_AGKPI | PA2HB\_OXYSC | PA2HH\_TRIST | PA2HS\_ECHCA | PA2H\_BOTPA | PA2H\_DEIAC | PA2H\_PROMB | PA2N\_GLOHA | PA2\_APIME | PAG15\_HUMAN | PLA22\_ORYSJ | Q6S5M9\_9ASCO | Q6WER3\_GIBZA | Q7LST4\_PENEN | Q95KP4\_HORSE | Q9P8F7\_YARLL | GO:0090729 | 0.0224871308588458 | 83/3691 | 68/641 | 8.89175742702576e-39 | 3.56559472823733e-36 | T | T | T | T | toxin activity | A1HA\_LOXIN | A1HB2\_LOXIN | A311\_LOXLA | E0CX04\_MOMBA | O04358\_IRIHO | OXLA\_BOTAT | OXLA\_CALRH | OXLA\_GLOHA | PA2A1\_BUNCE | PA2A1\_ECHCA | PA2A1\_NAJAT | PA2A1\_OPHHA | PA2A2\_OPHHA | PA2A2\_TROCA | PA2A4\_NAJSG | PA2A5\_TRIST | PA2A\_BOTJR | PA2A\_CROAT | PA2A\_DEIAC | PA2A\_GLOHA | PA2A\_NAJAT | PA2B2\_BOTJR | PA2B2\_PROFL | PA2B3\_BOTAS | PA2B3\_BUNCE | PA2B5\_BUNCE | PA2B5\_NOTSC | PA2BA\_VIPAA | PA2BB\_GLOHA | PA2BC\_VIPAA | PA2BD\_CRODU | PA2B\_BUNCE | PA2B\_NOTSC | PA2H1\_AGKCL | PA2H1\_BOTBZ | PA2H1\_BOTJR | PA2H1\_BOTMO | PA2H1\_BOTPI | PA2H2\_BOTAS | PA2H2\_BOTMO | PA2H2\_BOTPI | PA2H2\_CERGO | PA2H3\_BOTPI | PA2HB\_AGKPI | PA2HH\_TRIST | PA2H\_BOTPA | PA2H\_DEIAC | PA2H\_PROMB | PA2N\_GLOHA | PDE\_NAJAT | Q2QEH4\_SAPOF | Q94BW3\_CINCA | RIP0\_DIACA | RIP1\_BRYDI | RIP1\_HORVU | RIP1\_MOMCH | RIP1\_PHYAM | RIP2\_PHYAM | RIP3\_MOMCH | RIPA\_PHYAM | RIPG\_SURMU | RIPL1\_PHYDI | RIPL2\_PHYDI | RIPT\_TRIKI | VM1A3\_DEIAC | VM1BI\_BOTMO | VSPP\_DEIAC | VSPSX\_GLOSA | GO:0004623 | 0.0170685451097264 | 63/3691 | 57/641 | 1.32388058862122e-37 | 5.30876116037108e-35 | T | T | T | T | phospholipase A2 activity | A0A1L8D5Z7\_BOTAT | I1SB18\_VIPAE | PA21B\_BOVIN | PA21B\_PIG | PA2A1\_BUNCE | PA2A1\_ECHCA | PA2A1\_NAJAT | PA2A1\_OPHHA | PA2A2\_NAJNA | PA2A2\_OPHHA | PA2A2\_TROCA | PA2A4\_NAJSG | PA2A5\_TRIST | PA2A7\_GLOHA | PA2A\_BOTJR | PA2A\_CROAT | PA2A\_DEIAC | PA2A\_GLOHA | PA2A\_NAJAT | PA2B1\_AGKPI | PA2B2\_BOTJR | PA2B2\_PROFL | PA2B3\_BOTAS | PA2B3\_BUNCE | PA2B5\_BUNCE | PA2B5\_NOTSC | PA2BA\_VIPAA | PA2BB\_GLOHA | PA2BB\_PSEAU | PA2BC\_VIPAA | PA2BD\_CRODU | PA2B\_BUNCE | PA2B\_NOTSC | PA2GA\_HUMAN | PA2GE\_HUMAN | PA2GX\_HUMAN | PA2H1\_AGKCL | PA2H1\_BOTBZ | PA2H1\_BOTJR | PA2H1\_BOTMO | PA2H1\_BOTPI | PA2H2\_BOTAS | PA2H2\_BOTMO | PA2H2\_BOTPI | PA2H2\_CERGO | PA2H3\_BOTPI | PA2HB\_AGKPI | PA2HB\_OXYSC | PA2HH\_TRIST | PA2HS\_ECHCA | PA2H\_BOTPA | PA2H\_DEIAC | PA2H\_PROMB | PA2N\_GLOHA | PA2\_APIME | PAG15\_HUMAN | PLA22\_ORYSJ | GO:0005509 | 0.0354917366567326 | 131/3691 | 84/641 | 4.69393970250281e-34 | 1.88226982070363e-31 | T | T | T | T | calcium ion binding | A0A1L8D5Z7\_BOTAT | AMY1A\_HUMAN | AMY1\_HORVU | AMY1\_ORYSJ | AMYA1\_ASPOR | AOAH\_MOUSE | AOC1\_HUMAN | CHIT\_PUNGR | DNSL3\_HUMAN | ENPP2\_HUMAN | ENPP2\_RAT | GRASS\_DROME | I1SB18\_VIPAE | K9L8F3\_MALCI | LALBA\_BOVIN | LALBA\_CAPHI | LALBA\_CAVPO | LALBA\_PAPCY | LIPR1\_CANLF | LIPR1\_HUMAN | LIPR2\_HUMAN | LIPR2\_RAT | M9TI89\_RHIPU | MAN12\_PENCI | PA21B\_BOVIN | PA21B\_PIG | PA2A1\_BUNCE | PA2A1\_ECHCA | PA2A1\_NAJAT | PA2A1\_OPHHA | PA2A2\_NAJNA | PA2A2\_OPHHA | PA2A2\_TROCA | PA2A4\_NAJSG | PA2A5\_TRIST | PA2A7\_GLOHA | PA2A\_BOTJR | PA2A\_CROAT | PA2A\_DEIAC | PA2A\_GLOHA | PA2A\_NAJAT | PA2B1\_AGKPI | PA2B2\_BOTJR | PA2B2\_PROFL | PA2B3\_BOTAS | PA2B3\_BUNCE | PA2B5\_BUNCE | PA2B5\_NOTSC | PA2BA\_VIPAA | PA2BB\_GLOHA | PA2BB\_PSEAU | PA2BC\_VIPAA | PA2BD\_CRODU | PA2B\_BUNCE | PA2B\_NOTSC | PA2GA\_HUMAN | PA2GE\_HUMAN | PA2GX\_HUMAN | PA2H1\_AGKCL | PA2H1\_BOTBZ | PA2H1\_BOTJR | PA2H1\_BOTMO | PA2H1\_BOTPI | PA2H2\_BOTAS | PA2H2\_BOTMO | PA2H2\_BOTPI | PA2H2\_CERGO | PA2H3\_BOTPI | PA2HB\_AGKPI | PA2HB\_OXYSC | PA2HH\_TRIST | PA2HS\_ECHCA | PA2H\_BOTPA | PA2H\_DEIAC | PA2H\_PROMB | PA2N\_GLOHA | PA2\_APIME | PER1\_SORBI | PERL\_BOVIN | PERL\_BUBBU | PERL\_CAPHI | PLA22\_ORYSJ | PPAF1\_HOLDI | VM1A3\_DEIAC | GO:0016788 | 0.110268219994581 | 407/3691 | 167/641 | 1.28152449102492e-33 | 5.13891320900991e-31 | T | T | T | T | hydrolase activity, acting on ester bonds | A0A060N399\_9PLEO | A0A075B5G4\_HUMIN | A0A1D3S5H0\_FUSOX | A0A1L8D5Z7\_BOTAT | A0A1S6YJF3\_MALCI | A1HA\_LOXIN | A1HB2\_LOXIN | A2QIR3\_ASPNC | A2TBB4\_9ASCO | A311\_LOXLA | A4GX63\_TOXGO | AMPS2\_LITPI | ANG1\_BOVIN | ANG2\_MOUSE | ANG3\_MOUSE | ANG4\_MOUSE | ANGI\_MOUSE | AOAH\_MOUSE | ASM3A\_HUMAN | ASM3A\_MOUSE | ATLE\_CYCAE | AXE1\_ASPAW | AXE2\_TALPU | B1Q4V2\_HERER | CUTI1\_ASPOR | CUTI1\_COLGL | CUTI1\_FUSVN | CUTI1\_HYPJR | DNAS1\_HUMAN | DNSL3\_HUMAN | E0A7J0\_YARLL | E7FH77\_DANRE | ECP\_HUMAN | ENDO2\_ARATH | ENPP2\_HUMAN | ENPP2\_RAT | EST6\_DROME | F6MIW5\_WHEAT | FAEA\_ASPNG | FAEB1\_ASPOR | FAEB2\_ASPOR | G3YAL0\_ASPNA | GALNS\_HUMAN | GCE2\_MYCTT | GCE\_CERUI | GCE\_HYPJQ | I1SB18\_VIPAE | K7CID1\_PANTR | LICH\_HUMAN | LIP1\_DIURU | LIP2\_DIURU | LIP2\_GEOCN | LIP3\_DIURU | LIPA\_MOEAP | LIPB\_PSEA2 | LIPG\_CANLF | LIPG\_HUMAN | LIPP\_HORSE | LIPR1\_CANLF | LIPR1\_HUMAN | LIPR2\_HUMAN | LIPR2\_RAT | LIP\_THELA | NUP1\_PENCI | NUS1\_ASPOR | PA1\_VESBA | PA21B\_BOVIN | PA21B\_PIG | PA2A1\_BUNCE | PA2A1\_ECHCA | PA2A1\_NAJAT | PA2A1\_OPHHA | PA2A2\_NAJNA | PA2A2\_OPHHA | PA2A2\_TROCA | PA2A4\_NAJSG | PA2A5\_TRIST | PA2A7\_GLOHA | PA2A\_BOTJR | PA2A\_CROAT | PA2A\_DEIAC | PA2A\_GLOHA | PA2A\_NAJAT | PA2B1\_AGKPI | PA2B2\_BOTJR | PA2B2\_PROFL | PA2B3\_BOTAS | PA2B3\_BUNCE | PA2B5\_BUNCE | PA2B5\_NOTSC | PA2BA\_VIPAA | PA2BB\_GLOHA | PA2BB\_PSEAU | PA2BC\_VIPAA | PA2BD\_CRODU | PA2B\_BUNCE | PA2B\_NOTSC | PA2GA\_HUMAN | PA2GE\_HUMAN | PA2GX\_HUMAN | PA2H1\_AGKCL | PA2H1\_BOTBZ | PA2H1\_BOTJR | PA2H1\_BOTMO | PA2H1\_BOTPI | PA2H2\_BOTAS | PA2H2\_BOTMO | PA2H2\_BOTPI | PA2H2\_CERGO | PA2H3\_BOTPI | PA2HB\_AGKPI | PA2HB\_OXYSC | PA2HH\_TRIST | PA2HS\_ECHCA | PA2H\_BOTPA | PA2H\_DEIAC | PA2H\_PROMB | PA2N\_GLOHA | PA2\_APIME | PAG15\_HUMAN | PHAZ\_TALFU | PHYA\_ASPFU | PHYA\_ASPNG | PHYB\_ASPAW | PLA22\_ORYSJ | PME\_DAUCA | PME\_SITOR | PPA5\_HUMAN | PPA5\_PIG | PPA5\_RAT | PPAF1\_IPOBA | PPAF\_PHAVU | PPAP\_RAT | PPT1\_BOVIN | PPT1\_HUMAN | PPT2\_HUMAN | Q0KFV0\_SOLLC | Q45U61\_ASPNG | Q5WRG2\_RAT | Q6S5M9\_9ASCO | Q6WER3\_GIBZA | Q7LST4\_PENEN | Q7XZV5\_NICGU | Q874E9\_9TREE | Q8VX11\_LUPLU | Q95KP4\_HORSE | Q9GPG0\_MANSE | Q9M7C7\_CALSE | Q9P8F7\_YARLL | Q9SSV1\_NICGU | RHA1\_ASPAC | RNAS1\_BISBI | RNAS4\_HUMAN | RNAS4\_PIG | RNAS6\_HUMAN | RNF1\_GIBFU | RNLE\_SOLLC | RNRH\_RHINI | RNS11\_NICAL | RNS1B\_RAT | RNS3\_PYRPY | RNSL3\_DANRE | RNS\_BOVIN | RNT2\_HUMAN | S6BC01\_PSEA2 | V5NTD\_NAJAT | X0BTD8\_FUSOX | GO:0004620 | 0.0219452722839339 | 81/3691 | 62/641 | 3.38428835431093e-32 | 1.35709963007868e-29 | T | T | T | T | phospholipase activity | A0A1L8D5Z7\_BOTAT | ENPP2\_HUMAN | ENPP2\_RAT | I1SB18\_VIPAE | LIPR2\_HUMAN | LIPR2\_RAT | PA1\_VESBA | PA21B\_BOVIN | PA21B\_PIG | PA2A1\_BUNCE | PA2A1\_ECHCA | PA2A1\_NAJAT | PA2A1\_OPHHA | PA2A2\_NAJNA | PA2A2\_OPHHA | PA2A2\_TROCA | PA2A4\_NAJSG | PA2A5\_TRIST | PA2A7\_GLOHA | PA2A\_BOTJR | PA2A\_CROAT | PA2A\_DEIAC | PA2A\_GLOHA | PA2A\_NAJAT | PA2B1\_AGKPI | PA2B2\_BOTJR | PA2B2\_PROFL | PA2B3\_BOTAS | PA2B3\_BUNCE | PA2B5\_BUNCE | PA2B5\_NOTSC | PA2BA\_VIPAA | PA2BB\_GLOHA | PA2BB\_PSEAU | PA2BC\_VIPAA | PA2BD\_CRODU | PA2B\_BUNCE | PA2B\_NOTSC | PA2GA\_HUMAN | PA2GE\_HUMAN | PA2GX\_HUMAN | PA2H1\_AGKCL | PA2H1\_BOTBZ | PA2H1\_BOTJR | PA2H1\_BOTMO | PA2H1\_BOTPI | PA2H2\_BOTAS | PA2H2\_BOTMO | PA2H2\_BOTPI | PA2H2\_CERGO | PA2H3\_BOTPI | PA2HB\_AGKPI | PA2HB\_OXYSC | PA2HH\_TRIST | PA2HS\_ECHCA | PA2H\_BOTPA | PA2H\_DEIAC | PA2H\_PROMB | PA2N\_GLOHA | PA2\_APIME | PAG15\_HUMAN | PLA22\_ORYSJ | GO:0030248 | 0.00839880791113519 | 31/3691 | 31/641 | 1.46497131982913e-24 | 5.8745349925148e-22 | T | T | T | T | cellulose binding | A0A086SY89\_ACRC1 | A0A088T0J9\_GEOCN | A0A0J5Q413\_ASPFM | A0A0S2GKZ1\_9APHY | A0A1L9WG58\_ASPA1 | A0A2N1LTK3\_TRIHA | A0A5J6BJN2\_MALCI | A8NI40\_COPC7 | B7X9Z0\_COPCI | CBHB\_ASPFU | F1CYZ0\_TALFU | FAEA\_ASPNG | G0RVK1\_HYPJQ | G2Q665\_MYCTT | G9NTY1\_HYPAI | GCE\_CERUI | GCE\_HYPJQ | GUN2\_HYPJE | GUX1\_HUMGT | GUX1\_HYPJE | GUX1\_TRIHA | GUX2\_HYPJE | H1AE14\_PHACH | M2RAI8\_CERS8 | MANA\_HYPJR | Q02321\_PHACH | Q4WP32\_ASPFU | Q55FE6\_DICDI | Q7LHI2\_PHACH | Q7LIJ0\_PHACH | W4KMP1\_HETIT | GO:0030247 | 0.0121918179355188 | 45/3691 | 39/641 | 2.3882592138199e-24 | 9.57691944741781e-22 | T | T | T | T | polysaccharide binding | A0A086SY89\_ACRC1 | A0A088T0J9\_GEOCN | A0A0J5Q413\_ASPFM | A0A0S2GKZ1\_9APHY | A0A1L9WG58\_ASPA1 | A0A2N1LTK3\_TRIHA | A0A5J6BJN2\_MALCI | A8NI40\_COPC7 | ATLE\_CYCAE | B7X9Z0\_COPCI | CBHB\_ASPFU | ENPP2\_HUMAN | ENPP2\_RAT | F1CYZ0\_TALFU | FAEA\_ASPNG | G0RVK1\_HYPJQ | G2Q665\_MYCTT | G9NTY1\_HYPAI | GANA\_HUMIN | GCE\_CERUI | GCE\_HYPJQ | GUN2\_HYPJE | GUX1\_HUMGT | GUX1\_HYPJE | GUX1\_TRIHA | GUX2\_HYPJE | H1AE14\_PHACH | K7CID1\_PANTR | M2RAI8\_CERS8 | MANA\_CRYAT | MANA\_HYPJR | Q02321\_PHACH | Q4WP32\_ASPFU | Q55FE6\_DICDI | Q7LHI2\_PHACH | Q7LIJ0\_PHACH | S7ZIW0\_PENO1 | W4KMP1\_HETIT | XTH34\_POPPZ | GO:0008810 | 0.00758602004876727 | 28/3691 | 27/641 | 4.39675004312522e-20 | 1.76309676729321e-17 | T | T | T | T | cellulase activity | A0A086T6R4\_ACRC1 | A0A0S2GKZ1\_9APHY | A0A1L6CE30\_9EURO | A0A3G2C3I4\_9EURO | A0A5J6BJN2\_MALCI | A7KMF0\_9CAEN | EGLB\_ASPNG | F2Z7L1\_9ANNE | GUN2\_HYPJE | GUN6\_HUMIN | GUN7\_HYPJQ | GUNC\_FUSOX | GUN\_ASPAC | GUN\_CRYAT | GUN\_MYTED | H1AE14\_PHACH | I2FI81\_EISFE | I3RY46\_TRIHA | M2RAI8\_CERS8 | O00095\_HYPJE | O74705\_ASPNG | O77044\_9NEOP | Q4WP32\_ASPFU | Q8J0K8\_MELAO | Q8NJY6\_9HYPO | Q8TG26\_THEAU | W4KMP1\_HETIT | GO:0004175 | 0.0249254944459496 | 92/3691 | 52/641 | 9.08380235443639e-18 | 3.64260474412899e-15 | T | T | T | T | endopeptidase activity | A0A6P6YAT6\_DERPT | B4F320\_LIMPO | CARP1\_CANAL | CARP2\_CANAX | CARP\_RHIPU | CATD\_RAT | CATH\_HUMAN | CATLL\_FASHE | CEL2A\_PIG | CFAD\_MOUSE | CHYM\_CAMDR | COGS\_HYPLI | CUCM1\_CUCME | D6XHE1\_TRYB2 | DDN1\_BOVIN | EGFB2\_MOUSE | G3I1H5\_CRIGR | GRAA\_HUMAN | GRAC\_MOUSE | GRAK\_HUMAN | GRASS\_DROME | HE12\_DANRE | J7LCB0\_DEIAC | KLK10\_HUMAN | KLK1\_HUMAN | KLK2\_HORSE | KLK2\_HUMAN | KLK7\_HUMAN | KLK7\_MOUSE | KLK8\_MOUSE | LGMN\_MOUSE | MCPT2\_RAT | MMP1\_PIG | PEPA\_ASPPH | PPAF1\_HOLDI | PRS57\_HUMAN | PRTN3\_HUMAN | Q69G21\_TENMO | RENI\_RAT | TPP1\_HUMAN | TRFL\_BUBBU | TRY1\_GADMO | TRY3\_SALSA | TRYB2\_HUMAN | VM11\_BOTMO | VM12\_CROAD | VM1A3\_DEIAC | VM1BI\_BOTMO | VM1T1\_PROMU | VM1T2\_PROFL | VSPP\_DEIAC | VSPSX\_GLOSA | GO:0052716 | 0.0062313736114874 | 23/3691 | 22/641 | 2.67884580517042e-16 | 1.07421716787334e-13 | T | T | T | T | hydroquinone:oxygen oxidoreductase activity | A0A0M3U1T9\_9APHY | A0A2H4A2Q2\_9APHY | A0A3F2YLU5\_9APHY | A2QS62\_ASPNC | B2L9C1\_TRAHI | D7F485\_9APHY | F6N9E7\_9PEZI | I1SB14\_9APHY | I1VE66\_9APHY | LAC1\_MELAO | LAC1\_TRAMX | LAC2\_TRAVE | M1GME7\_9APHY | Q12571\_9BASI | Q1W6B1\_9APHY | Q5EBY5\_9APHY | Q6H9H7\_9APHY | Q8TG94\_TRAPU | Q96UT7\_TRAVE | Q9HDQ0\_9APHY | Q9UVQ2\_PYCCI | Q9Y780\_COPCI | GO:0004568 | 0.00677323218639935 | 25/3691 | 23/641 | 4.94272294248894e-16 | 1.98203189993806e-13 | T | T | T | T | chitinase activity | A0A3B6UEQ2\_RHIMI | A5AB48\_ASPNC | A9LI60\_BIOOC | A9ZSX9\_9BRYO | CHI1\_COCPS | CHI2\_HORVU | CHI2\_ORYSJ | CHI33\_TRIHA | CHI42\_TRIHA | CHI4\_CRYJA | CHIA\_HUMAN | CHIC\_ARATH | CHIC\_SECCE | CHIT\_PUNGR | CHLY\_HEVBR | G3JPF7\_CORMM | LYS\_RUDPH | O81934\_CANEN | Q43576\_TOBAC | Q4AE59\_OSTFU | Q6WSR8\_PICAB | Q8H0C9\_VIGUN | Q9FUH3\_VIGUS | GO:0008236 | 0.0140883229477106 | 52/3691 | 35/641 | 1.2371000176383e-15 | 4.96077107072958e-13 | T | T | T | T | serine-type peptidase activity | A0A6P6YAT6\_DERPT | CATH\_HUMAN | CEL2A\_PIG | CFAD\_MOUSE | COGS\_HYPLI | CUCM1\_CUCME | DDN1\_BOVIN | DPP2\_HUMAN | EGFB2\_MOUSE | GRAA\_HUMAN | GRAC\_MOUSE | GRAK\_HUMAN | GRASS\_DROME | J7LCB0\_DEIAC | KLK10\_HUMAN | KLK1\_HUMAN | KLK2\_HORSE | KLK2\_HUMAN | KLK7\_HUMAN | KLK7\_MOUSE | KLK8\_MOUSE | MCPT2\_RAT | MMP1\_PIG | PCP\_HUMAN | PPAF1\_HOLDI | PRS57\_HUMAN | PRTN3\_HUMAN | TPP1\_HUMAN | TRFL\_BUBBU | TRFL\_HORSE | TRY1\_GADMO | TRY3\_SALSA | TRYB2\_HUMAN | VSPP\_DEIAC | VSPSX\_GLOSA | GO:0017171 | 0.0149011108100786 | 55/3691 | 36/641 | 1.84703822217271e-15 | 7.40662327091255e-13 | T | T | T | T | serine hydrolase activity | A0A6P6YAT6\_DERPT | CATH\_HUMAN | CEL2A\_PIG | CFAD\_MOUSE | COGS\_HYPLI | CUCM1\_CUCME | DDN1\_BOVIN | DPP2\_HUMAN | EGFB2\_MOUSE | EST6\_DROME | GRAA\_HUMAN | GRAC\_MOUSE | GRAK\_HUMAN | GRASS\_DROME | J7LCB0\_DEIAC | KLK10\_HUMAN | KLK1\_HUMAN | KLK2\_HORSE | KLK2\_HUMAN | KLK7\_HUMAN | KLK7\_MOUSE | KLK8\_MOUSE | MCPT2\_RAT | MMP1\_PIG | PCP\_HUMAN | PPAF1\_HOLDI | PRS57\_HUMAN | PRTN3\_HUMAN | TPP1\_HUMAN | TRFL\_BUBBU | TRFL\_HORSE | TRY1\_GADMO | TRY3\_SALSA | TRYB2\_HUMAN | VSPP\_DEIAC | VSPSX\_GLOSA | GO:0008061 | 0.00704416147385532 | 26/3691 | 23/641 | 3.59213860145614e-15 | 1.44044757918391e-12 | T | T | T | T | chitin binding | A0A0R4I979\_BRABE | A0A3B6UEQ2\_RHIMI | A0A482LWB1\_OSTFU | A5AB48\_ASPNC | A9LI60\_BIOOC | CDA\_COLLN | CDA\_EMENI | CHI1\_COCPS | CHI2\_ORYSJ | CHI33\_TRIHA | CHI42\_TRIHA | CHI4\_CRYJA | CHIA\_HUMAN | CHIC\_ARATH | CHIC\_SECCE | CHIL3\_MOUSE | O81934\_CANEN | Q43576\_TOBAC | Q4AE59\_OSTFU | Q4W6L6\_CYCRE | Q6WSR8\_PICAB | Q8H0C9\_VIGUN | Q9FUH3\_VIGUS | GO:0004252 | 0.0127336765104308 | 47/3691 | 32/641 | 1.30723263922177e-14 | 5.24200288327928e-12 | T | T | T | T | serine-type endopeptidase activity | A0A6P6YAT6\_DERPT | CATH\_HUMAN | CEL2A\_PIG | CFAD\_MOUSE | COGS\_HYPLI | CUCM1\_CUCME | DDN1\_BOVIN | EGFB2\_MOUSE | GRAA\_HUMAN | GRAC\_MOUSE | GRAK\_HUMAN | GRASS\_DROME | J7LCB0\_DEIAC | KLK10\_HUMAN | KLK1\_HUMAN | KLK2\_HORSE | KLK2\_HUMAN | KLK7\_HUMAN | KLK7\_MOUSE | KLK8\_MOUSE | MCPT2\_RAT | MMP1\_PIG | PPAF1\_HOLDI | PRS57\_HUMAN | PRTN3\_HUMAN | TPP1\_HUMAN | TRFL\_BUBBU | TRY1\_GADMO | TRY3\_SALSA | TRYB2\_HUMAN | VSPP\_DEIAC | VSPSX\_GLOSA | GO:0140825 | 0.0062313736114874 | 23/3691 | 21/641 | 1.46323924901514e-14 | 5.86758938855071e-12 | T | T | T | T | lactoperoxidase activity | A0A087WNH2\_FICBE | A0A0A0Y4H8\_TRAFO | A0A1S4NYF8\_PANVG | A0A3L6SKP5\_PANMI | D1MPT2\_ROYRE | DYP\_AURAJ | K7N5L9\_RAPSA | L8ICE9\_9CETA | O22443\_SOYBN | PER1A\_ARMRU | PER1\_ARAHY | PER1\_SORBI | PER53\_ARATH | PER59\_ARATH | PERL\_BOVIN | PERL\_BUBBU | PERL\_CAPHI | PER\_ARTRA | PER\_COPCI | POXA\_DICDI | Q40069\_HORVU | GO:0008233 | 0.0452451910051477 | 167/3691 | 69/641 | 7.07020099837636e-14 | 2.83515060034892e-11 | T | T | T | T | peptidase activity | A0A6P6YAT6\_DERPT | A0NFU8\_ANOGA | B4F320\_LIMPO | CARP1\_CANAL | CARP2\_CANAX | CARP\_RHIPU | CATD\_RAT | CATH\_HUMAN | CATLL\_FASHE | CBPA1\_PIG | CBPD\_LOPSP | CBPN\_HUMAN | CEL2A\_PIG | CFAD\_MOUSE | CHYM\_CAMDR | COGS\_HYPLI | CUCM1\_CUCME | CYSP\_BLOTA | D6XHE1\_TRYB2 | DDN1\_BOVIN | DPP2\_HUMAN | EGFB2\_MOUSE | ERVB\_TABDI | G3I1H5\_CRIGR | GGH\_HUMAN | GRAA\_HUMAN | GRAC\_MOUSE | GRAK\_HUMAN | GRASS\_DROME | HE12\_DANRE | J7LCB0\_DEIAC | KLK10\_HUMAN | KLK1\_HUMAN | KLK2\_HORSE | KLK2\_HUMAN | KLK7\_HUMAN | KLK7\_MOUSE | KLK8\_MOUSE | LAPA\_ASPOR | LGMN\_MOUSE | LYS\_RUDPH | MCPT2\_RAT | MMP1\_PIG | O97389\_HELAM | PCP\_HUMAN | PEPA\_ASPPH | PGPSA\_DROME | PPAF1\_HOLDI | PRS57\_HUMAN | PRTN3\_HUMAN | Q69G21\_TENMO | Q6NY42\_DANRE | Q6R7Z5\_9TRYP | Q7YXL2\_TENMO | RENI\_RAT | TPP1\_HUMAN | TRFL\_BUBBU | TRFL\_HORSE | TRY1\_GADMO | TRY3\_SALSA | TRYB2\_HUMAN | VM11\_BOTMO | VM12\_CROAD | VM1A3\_DEIAC | VM1BI\_BOTMO | VM1T1\_PROMU | VM1T2\_PROFL | VSPP\_DEIAC | VSPSX\_GLOSA | GO:0004806 | 0.00596044432403143 | 22/3691 | 20/641 | 7.90056629711627e-14 | 3.16812708514362e-11 | T | T | T | T | triglyceride lipase activity | E0A7J0\_YARLL | LIP1\_DIURU | LIP2\_DIURU | LIP2\_GEOCN | LIP3\_DIURU | LIPA\_MOEAP | LIPB\_PSEA2 | LIPG\_CANLF | LIPG\_HUMAN | LIPP\_HORSE | LIPR1\_CANLF | LIPR1\_HUMAN | LIPR2\_HUMAN | LIPR2\_RAT | LIP\_THELA | Q6S5M9\_9ASCO | Q6WER3\_GIBZA | Q7LST4\_PENEN | Q95KP4\_HORSE | Q9P8F7\_YARLL | GO:0016682 | 0.00785694933622325 | 29/3691 | 23/641 | 3.87026391389371e-13 | 1.55197582947138e-10 | T | T | T | T | oxidoreductase activity, acting on diphenols and related substances as donors, oxygen as acceptor | A0A0M3U1T9\_9APHY | A0A2H4A2Q2\_9APHY | A0A3F2YLU5\_9APHY | A2QS62\_ASPNC | B2L9C1\_TRAHI | D7F485\_9APHY | F6N9E7\_9PEZI | I1SB14\_9APHY | I1VE66\_9APHY | LAC1\_MELAO | LAC1\_TRAMX | LAC2\_TRAVE | M1GME7\_9APHY | PPO8\_ANOGA | Q12571\_9BASI | Q1W6B1\_9APHY | Q5EBY5\_9APHY | Q6H9H7\_9APHY | Q8TG94\_TRAPU | Q96UT7\_TRAVE | Q9HDQ0\_9APHY | Q9UVQ2\_PYCCI | Q9Y780\_COPCI | GO:0004519 | 0.0146301815226226 | 54/3691 | 33/641 | 5.23876304831635e-13 | 2.10074398237486e-10 | T | T | T | T | endonuclease activity | AMPS2\_LITPI | ANG1\_BOVIN | ANG2\_MOUSE | ANG3\_MOUSE | ANG4\_MOUSE | ANGI\_MOUSE | B1Q4V2\_HERER | DNAS1\_HUMAN | DNSL3\_HUMAN | E7FH77\_DANRE | ECP\_HUMAN | ENDO2\_ARATH | NUP1\_PENCI | NUS1\_ASPOR | Q0KFV0\_SOLLC | Q45U61\_ASPNG | Q5WRG2\_RAT | Q7XZV5\_NICGU | Q9M7C7\_CALSE | Q9SSV1\_NICGU | RNAS1\_BISBI | RNAS4\_HUMAN | RNAS4\_PIG | RNAS6\_HUMAN | RNF1\_GIBFU | RNLE\_SOLLC | RNRH\_RHINI | RNS11\_NICAL | RNS1B\_RAT | RNS3\_PYRPY | RNSL3\_DANRE | RNS\_BOVIN | RNT2\_HUMAN | GO:0005507 | 0.021132484421566 | 78/3691 | 41/641 | 8.5039146313893e-13 | 3.41006976718711e-10 | T | T | T | T | copper ion binding | A0A0A7M685\_TRAHI | A0A0M3U1T9\_9APHY | A0A2H4A2Q2\_9APHY | A0A3F2YLU5\_9APHY | A2QS62\_ASPNC | ANG1\_BOVIN | ANG2\_MOUSE | ANG3\_MOUSE | ANG4\_MOUSE | ANGI\_MOUSE | AOC1\_HUMAN | AOCX\_BOVIN | B2L9C1\_TRAHI | BLRO\_ALBVE | CERU\_RAT | D7F485\_9APHY | DOPO\_HUMAN | F6N9E7\_9PEZI | G2QG31\_MYCTT | H8ZRU2\_9HELO | I1SB14\_9APHY | I1VE66\_9APHY | LAC1\_MELAO | LAC1\_TRAMX | LAC2\_TRAVE | M1GME7\_9APHY | Q08J22\_BOMMO | Q12571\_9BASI | Q1W6B1\_9APHY | Q5B038\_EMENI | Q5EBY5\_9APHY | Q5WRG2\_RAT | Q6H9H7\_9APHY | Q8TG94\_TRAPU | Q96TR6\_PYCCO | Q96UT7\_TRAVE | Q96X16\_PICPA | Q9HDQ0\_9APHY | Q9UVQ2\_PYCCI | Q9Y780\_COPCI | SODE\_ONCVO | GO:0061783 | 0.00812787862367922 | 30/3691 | 23/641 | 1.39100884368123e-12 | 5.57794546316171e-10 | T | T | T | T | peptidoglycan muralytic activity | A0A0R4I979\_BRABE | A0A7S6G7I6\_9PEZI | A6PZ97\_SALSA | B9TU22\_GADMO | CHLY\_HEVBR | LALBA\_BOVIN | LYG\_STRCA | LYS1\_MUSDO | LYSC1\_ANAPL | LYSC1\_CANLF | LYSC1\_HORSE | LYSC2\_BOVIN | LYSC2\_ONCMY | LYSC\_COTJA | LYSC\_EQUAS | LYSC\_NUMME | LYSC\_OPIHO | LYSC\_PELSI | LYS\_BOMMO | LYS\_RUDPH | PGRP1\_CAMDR | Q86RS6\_MANSE | Q95V66\_PENVA | GO:0097599 | 0.00704416147385532 | 26/3691 | 21/641 | 2.24534893516513e-12 | 9.00384923001217e-10 | T | T | T | T | xylanase activity | A0A086SY89\_ACRC1 | A0A0J5Q413\_ASPFM | A0A1L9WG58\_ASPA1 | C3VEV9\_PENCN | C7YSL3\_FUSV7 | D0QF43\_9HELO | Q6VAY1\_9PEZI | Q92458\_HYPJE | S7Q6I2\_GLOTA | W8VR85\_TALPI | X0M5X0\_FUSOX | XYN1\_HYPJR | XYN2\_HYPJR | XYN3\_ASPKW | XYN3\_HYPJQ | XYNA\_FUSO4 | XYNA\_PENSI | XYNA\_THEAU | XYNA\_THELA | XYNC\_ASPNC | XYND\_EMENI | GO:0016679 | 0.00839880791113519 | 31/3691 | 23/641 | 4.5203982888387e-12 | 1.81267971382432e-09 | T | T | T | T | oxidoreductase activity, acting on diphenols and related substances as donors | A0A0M3U1T9\_9APHY | A0A2H4A2Q2\_9APHY | A0A3F2YLU5\_9APHY | A2QS62\_ASPNC | B2L9C1\_TRAHI | D7F485\_9APHY | F6N9E7\_9PEZI | I1SB14\_9APHY | I1VE66\_9APHY | LAC1\_MELAO | LAC1\_TRAMX | LAC2\_TRAVE | M1GME7\_9APHY | PPO8\_ANOGA | Q12571\_9BASI | Q1W6B1\_9APHY | Q5EBY5\_9APHY | Q6H9H7\_9APHY | Q8TG94\_TRAPU | Q96UT7\_TRAVE | Q9HDQ0\_9APHY | Q9UVQ2\_PYCCI | Q9Y780\_COPCI | GO:0003796 | 0.00677323218639935 | 25/3691 | 20/641 | 1.07311014717952e-11 | 4.30317169018988e-09 | T | T | T | T | lysozyme activity | A0A7S6G7I6\_9PEZI | A6PZ97\_SALSA | B9TU22\_GADMO | CHLY\_HEVBR | LALBA\_BOVIN | LYG\_STRCA | LYS1\_MUSDO | LYSC1\_ANAPL | LYSC1\_CANLF | LYSC1\_HORSE | LYSC2\_BOVIN | LYSC2\_ONCMY | LYSC\_COTJA | LYSC\_EQUAS | LYSC\_NUMME | LYSC\_OPIHO | LYSC\_PELSI | LYS\_BOMMO | LYS\_RUDPH | Q95V66\_PENVA | GO:0031176 | 0.0062313736114874 | 23/3691 | 19/641 | 1.25964329553986e-11 | 5.05116961511485e-09 | T | T | T | T | endo-1,4-beta-xylanase activity | A0A086SY89\_ACRC1 | A0A0J5Q413\_ASPFM | A0A1L9WG58\_ASPA1 | C3VEV9\_PENCN | C7YSL3\_FUSV7 | D0QF43\_9HELO | Q6VAY1\_9PEZI | S7Q6I2\_GLOTA | W8VR85\_TALPI | X0M5X0\_FUSOX | XYN1\_HYPJR | XYN2\_HYPJR | XYN3\_ASPKW | XYN3\_HYPJQ | XYNA\_FUSO4 | XYNA\_PENSI | XYNA\_THEAU | XYNA\_THELA | XYNC\_ASPNC | GO:0030246 | 0.0327824437821729 | 121/3691 | 52/641 | 1.77051265680031e-11 | 7.09975575376925e-09 | T | T | T | T | carbohydrate binding | A0A086SY89\_ACRC1 | A0A088T0J9\_GEOCN | A0A0J5Q413\_ASPFM | A0A0S2GKZ1\_9APHY | A0A1L9WG58\_ASPA1 | A0A2N1LTK3\_TRIHA | A0A5J6BJN2\_MALCI | A8NI40\_COPC7 | ATLE\_CYCAE | B7X9Z0\_COPCI | BGALA\_ASPNC | BGALA\_ASPOR | BGALA\_PENSQ | BGLR\_HUMAN | CBHB\_ASPFU | CHIL3\_MOUSE | DOPO\_HUMAN | ENPP2\_HUMAN | ENPP2\_RAT | F1CYZ0\_TALFU | FAEA\_ASPNG | G0RVK1\_HYPJQ | G2Q665\_MYCTT | G9NTY1\_HYPAI | GANA\_HUMIN | GCE\_CERUI | GCE\_HYPJQ | GUN2\_HYPJE | GUX1\_HUMGT | GUX1\_HYPJE | GUX1\_TRIHA | GUX2\_HYPJE | H1AE14\_PHACH | K7CID1\_PANTR | M2RAI8\_CERS8 | MANA\_CANEN | MANA\_CRYAT | MANA\_HYPJR | MANBA\_MOUSE | Q02321\_PHACH | Q4WP32\_ASPFU | Q50KB2\_PHACH | Q55FE6\_DICDI | Q7LHI2\_PHACH | Q7LIJ0\_PHACH | Q9STC1\_GRALE | RGLA\_ASPAC | RIPG\_SURMU | S7ZIW0\_PENO1 | W4KMP1\_HETIT | XTH34\_POPPZ | XYLA\_ASPNC | GO:0008201 | 0.00325115144947169 | 12/3691 | 12/641 | 6.90804357255504e-10 | 2.77012547259457e-07 | T | T | T | T | heparin binding | ADA2\_HUMAN | ANG1\_BOVIN | ANG2\_MOUSE | ANG3\_MOUSE | ANG4\_MOUSE | ANGI\_MOUSE | AOC1\_HUMAN | PA2H1\_AGKCL | PA2H1\_BOTJR | PA2H2\_BOTAS | PRS57\_HUMAN | Q5WRG2\_RAT | GO:0004650 | 0.00298022216201571 | 11/3691 | 11/641 | 4.03517465825728e-09 | 1.61810503796117e-06 | T | T | T | T | polygalacturonase activity | A0A6M9BP13\_9EURO | A1E266\_9PEZI | ADPG2\_ARATH | P79074\_9AGAR | PGLR1\_ASPAC | PGLR1\_ASPNG | PGLR\_GIBFU | Q2Z1W1\_PHACH | Q9LYJ5\_ARATH | RHGA\_ASPAC | XGHA\_ASPTU | GO:0004518 | 0.0216743429964779 | 80/3691 | 36/641 | 5.82224759000793e-09 | 2.33472128359318e-06 | T | T | T | T | nuclease activity | AMPS2\_LITPI | ANG1\_BOVIN | ANG2\_MOUSE | ANG3\_MOUSE | ANG4\_MOUSE | ANGI\_MOUSE | ATLE\_CYCAE | B1Q4V2\_HERER | DNAS1\_HUMAN | DNSL3\_HUMAN | E7FH77\_DANRE | ECP\_HUMAN | ENDO2\_ARATH | ENPP2\_HUMAN | ENPP2\_RAT | NUP1\_PENCI | NUS1\_ASPOR | Q0KFV0\_SOLLC | Q45U61\_ASPNG | Q5WRG2\_RAT | Q7XZV5\_NICGU | Q9M7C7\_CALSE | Q9SSV1\_NICGU | RNAS1\_BISBI | RNAS4\_HUMAN | RNAS4\_PIG | RNAS6\_HUMAN | RNF1\_GIBFU | RNLE\_SOLLC | RNRH\_RHINI | RNS11\_NICAL | RNS1B\_RAT | RNS3\_PYRPY | RNSL3\_DANRE | RNS\_BOVIN | RNT2\_HUMAN | GO:0005539 | 0.00541858574911948 | 20/3691 | 15/641 | 2.23645633514233e-08 | 8.96818990392075e-06 | T | T | T | T | glycosaminoglycan binding | ADA2\_HUMAN | ANG1\_BOVIN | ANG2\_MOUSE | ANG3\_MOUSE | ANG4\_MOUSE | ANGI\_MOUSE | AOC1\_HUMAN | PA2H1\_AGKCL | PA2H1\_BOTJR | PA2H2\_BOTAS | PGPSA\_DROME | PGRP1\_CAMDR | PRS57\_HUMAN | Q5WRG2\_RAT | Q86RS6\_MANSE | GO:0015925 | 0.00704416147385532 | 26/3691 | 17/641 | 6.46718707175934e-08 | 2.59334201577549e-05 | T | T | T | T | galactosidase activity | A0A2Z4HIN9\_9EURO | A0A384E148\_NICBE | AGAL\_HUMAN | AGAL\_ORYSJ | BGALA\_ASPNC | BGALA\_ASPOR | BGALA\_PENSQ | GANA\_ASPAC | GANA\_EMENI | GANA\_HUMIN | MEL1\_YEASX | NAGAB\_CHICK | NAGAB\_HUMAN | O81100\_SOLLC | Q50KB2\_PHACH | Q70SY0\_HYPJE | Q92456\_HYPJE | GO:0004540 | 0.0151720400975345 | 56/3691 | 27/641 | 8.3502718401457e-08 | 3.34845900789843e-05 | T | T | T | T | RNA nuclease activity | ANG1\_BOVIN | ANG2\_MOUSE | ANG3\_MOUSE | ANG4\_MOUSE | ANGI\_MOUSE | B1Q4V2\_HERER | ECP\_HUMAN | ENDO2\_ARATH | Q0KFV0\_SOLLC | Q45U61\_ASPNG | Q5WRG2\_RAT | Q7XZV5\_NICGU | Q9M7C7\_CALSE | Q9SSV1\_NICGU | RNAS1\_BISBI | RNAS4\_HUMAN | RNAS4\_PIG | RNAS6\_HUMAN | RNF1\_GIBFU | RNLE\_SOLLC | RNRH\_RHINI | RNS11\_NICAL | RNS1B\_RAT | RNS3\_PYRPY | RNSL3\_DANRE | RNS\_BOVIN | RNT2\_HUMAN | GO:0140102 | 0.00677323218639935 | 25/3691 | 16/641 | 2.49680843073009e-07 | 0.000100122018072276 | T | T | T | T | catalytic activity, acting on a rRNA | E0CX04\_MOMBA | O04358\_IRIHO | Q2QEH4\_SAPOF | Q94BW3\_CINCA | RIP0\_DIACA | RIP1\_BRYDI | RIP1\_HORVU | RIP1\_MOMCH | RIP1\_PHYAM | RIP2\_PHYAM | RIP3\_MOMCH | RIPA\_PHYAM | RIPG\_SURMU | RIPL1\_PHYDI | RIPL2\_PHYDI | RIPT\_TRIKI | GO:0030598 | 0.00677323218639935 | 25/3691 | 16/641 | 2.49680843073009e-07 | 0.000100122018072276 | T | T | T | T | rRNA N-glycosylase activity | E0CX04\_MOMBA | O04358\_IRIHO | Q2QEH4\_SAPOF | Q94BW3\_CINCA | RIP0\_DIACA | RIP1\_BRYDI | RIP1\_HORVU | RIP1\_MOMCH | RIP1\_PHYAM | RIP2\_PHYAM | RIP3\_MOMCH | RIPA\_PHYAM | RIPG\_SURMU | RIPL1\_PHYDI | RIPL2\_PHYDI | RIPT\_TRIKI | GO:0030597 | 0.00677323218639935 | 25/3691 | 16/641 | 2.49680843073009e-07 | 0.000100122018072276 | T | T | T | T | RNA glycosylase activity | E0CX04\_MOMBA | O04358\_IRIHO | Q2QEH4\_SAPOF | Q94BW3\_CINCA | RIP0\_DIACA | RIP1\_BRYDI | RIP1\_HORVU | RIP1\_MOMCH | RIP1\_PHYAM | RIP2\_PHYAM | RIP3\_MOMCH | RIPA\_PHYAM | RIPG\_SURMU | RIPL1\_PHYDI | RIPL2\_PHYDI | RIPT\_TRIKI | GO:0015926 | 0.0132755350853427 | 49/3691 | 24/641 | 3.06577205516353e-07 | 0.000122937459412058 | T | T | T | T | glucosidase activity | A0A068FT77\_9PEZI | A2TM14\_HEVBR | AMYG\_SACFI | BGL1\_ASPAC | BGLA\_ASPFU | BGLA\_ASPOR | D1M8S7\_HEVBR | E13B\_HORVU | E13C\_MUSAC | ENG1\_RHIMI | EXG1\_CANAL | EXG1\_YEAST | GANA\_ASPAC | GANA\_EMENI | GANA\_HUMIN | GBA1\_HUMAN | Q12715\_HYPJE | Q2Z1W1\_PHACH | Q70C53\_SOLTU | Q7RWP2\_NEUCR | Q8T0W7\_9NEOP | Q8TGI8\_TALEM | S7ZIW0\_PENO1 | TLP\_PRUAV | GO:0050525 | 0.00216743429964779 | 8/3691 | 8/641 | 7.97927642183382e-07 | 0.000319968984515536 | T | T | T | T | cutinase activity | A0A060N399\_9PLEO | A0A075B5G4\_HUMIN | A0A1S6YJF3\_MALCI | CUTI1\_ASPOR | CUTI1\_COLGL | CUTI1\_FUSVN | CUTI1\_HYPJR | X0BTD8\_FUSOX | GO:0004557 | 0.00216743429964779 | 8/3691 | 8/641 | 7.97927642183382e-07 | 0.000319968984515536 | T | T | T | T | alpha-galactosidase activity | A0A2Z4HIN9\_9EURO | A0A384E148\_NICBE | AGAL\_HUMAN | AGAL\_ORYSJ | MEL1\_YEASX | NAGAB\_CHICK | NAGAB\_HUMAN | Q92456\_HYPJE | GO:0016162 | 0.00270929287455974 | 10/3691 | 9/641 | 1.15954802130682e-06 | 0.000464978756544036 | T | T | T | T | cellulose 1,4-beta-cellobiosidase activity | CBHB\_ASPFU | E9G5J5\_DAPPU | F1CYZ0\_TALFU | GH7B\_LIMQU | GUX1\_HUMGT | GUX1\_HYPJE | GUX1\_TRIHA | GUX2\_HYPJE | Q55FE6\_DICDI | GO:0004567 | 0.00460579788675156 | 17/3691 | 12/641 | 1.81409683351035e-06 | 0.000727452830237652 | T | T | T | T | beta-mannosidase activity | A0A075B5H6\_TRIHA | A0A075C6T6\_RHIMI | A0A2U8ZTY7\_RHIZD | A0A3G4RHU4\_9PEZI | L7SVX1\_RHIMI | MAN4\_SOLLC | MANA\_ASPNC | MANA\_CRYAT | MANA\_HYPJR | MANA\_MYTED | MANA\_PODAN | MANBA\_MOUSE | GO:0015923 | 0.0062313736114874 | 23/3691 | 14/641 | 3.47772040187881e-06 | 0.0013945658811534 | T | T | T | T | mannosidase activity | A0A075B5H6\_TRIHA | A0A075C6T6\_RHIMI | A0A2U8ZTY7\_RHIZD | A0A3G4RHU4\_9PEZI | L7SVX1\_RHIMI | MAN12\_PENCI | MAN4\_SOLLC | MANA\_ASPNC | MANA\_CANEN | MANA\_CRYAT | MANA\_HYPJR | MANA\_MYTED | MANA\_PODAN | MANBA\_MOUSE | GO:0016837 | 0.00189650501219182 | 7/3691 | 7/641 | 4.6365385391244e-06 | 0.00185925195418889 | T | T | T | T | carbon-oxygen lyase activity, acting on polysaccharides | G2X3Y1\_VERDV | NANL\_MACDE | PELA\_ASPNG | PELB\_ASPNG | PLY1\_JUNAS | Q9STC1\_GRALE | RGLA\_ASPAC | GO:0033897 | 0.00298022216201571 | 11/3691 | 9/641 | 5.39388753641341e-06 | 0.00216294890210178 | T | T | T | T | ribonuclease T2 activity | Q45U61\_ASPNG | Q7XZV5\_NICGU | Q9M7C7\_CALSE | Q9SSV1\_NICGU | RNLE\_SOLLC | RNRH\_RHINI | RNS11\_NICAL | RNS3\_PYRPY | RNT2\_HUMAN | GO:0008422 | 0.010566242210783 | 39/3691 | 19/641 | 5.97855427806898e-06 | 0.00239740026550566 | T | T | T | T | beta-glucosidase activity | A0A068FT77\_9PEZI | A2TM14\_HEVBR | BGL1\_ASPAC | BGLA\_ASPFU | BGLA\_ASPOR | D1M8S7\_HEVBR | E13B\_HORVU | E13C\_MUSAC | ENG1\_RHIMI | EXG1\_CANAL | EXG1\_YEAST | GBA1\_HUMAN | Q12715\_HYPJE | Q2Z1W1\_PHACH | Q70C53\_SOLTU | Q7RWP2\_NEUCR | Q8T0W7\_9NEOP | Q8TGI8\_TALEM | TLP\_PRUAV | GO:0004521 | 0.00894066648604714 | 33/3691 | 17/641 | 7.1489600681312e-06 | 0.00286673298732061 | T | T | T | T | RNA endonuclease activity | B1Q4V2\_HERER | ENDO2\_ARATH | Q0KFV0\_SOLLC | Q45U61\_ASPNG | Q7XZV5\_NICGU | Q9M7C7\_CALSE | Q9SSV1\_NICGU | RNAS1\_BISBI | RNAS4\_HUMAN | RNF1\_GIBFU | RNLE\_SOLLC | RNRH\_RHINI | RNS11\_NICAL | RNS1B\_RAT | RNS3\_PYRPY | RNS\_BOVIN | RNT2\_HUMAN | GO:0140098 | 0.0365754538065565 | 135/3691 | 43/641 | 2.09288247933187e-05 | 0.00839245874212081 | T | T | T | T | catalytic activity, acting on RNA | ANG1\_BOVIN | ANG2\_MOUSE | ANG3\_MOUSE | ANG4\_MOUSE | ANGI\_MOUSE | B1Q4V2\_HERER | E0CX04\_MOMBA | ECP\_HUMAN | ENDO2\_ARATH | O04358\_IRIHO | Q0KFV0\_SOLLC | Q2QEH4\_SAPOF | Q45U61\_ASPNG | Q5WRG2\_RAT | Q7XZV5\_NICGU | Q94BW3\_CINCA | Q9M7C7\_CALSE | Q9SSV1\_NICGU | RIP0\_DIACA | RIP1\_BRYDI | RIP1\_HORVU | RIP1\_MOMCH | RIP1\_PHYAM | RIP2\_PHYAM | RIP3\_MOMCH | RIPA\_PHYAM | RIPG\_SURMU | RIPL1\_PHYDI | RIPL2\_PHYDI | RIPT\_TRIKI | RNAS1\_BISBI | RNAS4\_HUMAN | RNAS4\_PIG | RNAS6\_HUMAN | RNF1\_GIBFU | RNLE\_SOLLC | RNRH\_RHINI | RNS11\_NICAL | RNS1B\_RAT | RNS3\_PYRPY | RNSL3\_DANRE | RNS\_BOVIN | RNT2\_HUMAN | GO:0004556 | 0.00270929287455974 | 10/3691 | 8/641 | 2.57829346802097e-05 | 0.0103389568067641 | T | T | T | T | alpha-amylase activity | A0A173N065\_EISFE | AMY1A\_HUMAN | AMY1\_HORVU | AMY1\_ORYSJ | AMYA1\_ASPOR | AMY\_ORYLA | K9L8F3\_MALCI | M9TI89\_RHIPU | GO:0052692 | 0.00162557572473584 | 6/3691 | 6/641 | 2.69065267979074e-05 | 0.0107895172459609 | T | T | T | T | raffinose alpha-galactosidase activity | A0A2Z4HIN9\_9EURO | A0A384E148\_NICBE | AGAL\_HUMAN | AGAL\_ORYSJ | MEL1\_YEASX | Q92456\_HYPJE | GO:0016849 | 0.00352208073692766 | 13/3691 | 9/641 | 5.02046660556929e-05 | 0.0201320710883329 | T | T | T | T | phosphorus-oxygen lyase activity | Q45U61\_ASPNG | Q7XZV5\_NICGU | Q9M7C7\_CALSE | Q9SSV1\_NICGU | RNLE\_SOLLC | RNRH\_RHINI | RNS11\_NICAL | RNS3\_PYRPY | RNT2\_HUMAN | GO:0016985 | 0.00352208073692766 | 13/3691 | 9/641 | 5.02046660556929e-05 | 0.0201320710883329 | T | T | T | T | mannan endo-1,4-beta-mannosidase activity | A0A2U8ZTY7\_RHIZD | A0A3G4RHU4\_9PEZI | L7SVX1\_RHIMI | MAN4\_SOLLC | MANA\_ASPNC | MANA\_CRYAT | MANA\_HYPJR | MANA\_MYTED | MANA\_PODAN | GO:0140640 | 0.041452180980764 | 153/3691 | 46/641 | 5.57693890433794e-05 | 0.0223635250063951 | T | T | T | T | catalytic activity, acting on a nucleic acid | ANG1\_BOVIN | ANG2\_MOUSE | ANG3\_MOUSE | ANG4\_MOUSE | ANGI\_MOUSE | ATLE\_CYCAE | B1Q4V2\_HERER | DNAS1\_HUMAN | DNSL3\_HUMAN | E0CX04\_MOMBA | ECP\_HUMAN | ENDO2\_ARATH | O04358\_IRIHO | Q0KFV0\_SOLLC | Q2QEH4\_SAPOF | Q45U61\_ASPNG | Q5WRG2\_RAT | Q7XZV5\_NICGU | Q94BW3\_CINCA | Q9M7C7\_CALSE | Q9SSV1\_NICGU | RIP0\_DIACA | RIP1\_BRYDI | RIP1\_HORVU | RIP1\_MOMCH | RIP1\_PHYAM | RIP2\_PHYAM | RIP3\_MOMCH | RIPA\_PHYAM | RIPG\_SURMU | RIPL1\_PHYDI | RIPL2\_PHYDI | RIPT\_TRIKI | RNAS1\_BISBI | RNAS4\_HUMAN | RNAS4\_PIG | RNAS6\_HUMAN | RNF1\_GIBFU | RNLE\_SOLLC | RNRH\_RHINI | RNS11\_NICAL | RNS1B\_RAT | RNS3\_PYRPY | RNSL3\_DANRE | RNS\_BOVIN | RNT2\_HUMAN | GO:0070001 | 0.00243836358710377 | 9/3691 | 7/641 | 0.000120485877943708 | 0.0483148370554267 | T | T | T | T | aspartic-type peptidase activity | CARP1\_CANAL | CARP2\_CANAX | CARP\_RHIPU | CATD\_RAT | CHYM\_CAMDR | PEPA\_ASPPH | RENI\_RAT | GO:0046556 | 0.00243836358710377 | 9/3691 | 7/641 | 0.000120485877943708 | 0.0483148370554267 | T | T | T | T | alpha-L-arabinofuranosidase activity | A0A059U759\_9PEZI | A0A2H5BN17\_TALPI | A8NI40\_COPC7 | ABFB\_ASPKW | AXHA2\_EMENI | G2QVH2\_THETT | XYND\_EMENI | GO:0004190 | 0.00243836358710377 | 9/3691 | 7/641 | 0.000120485877943708 | 0.0483148370554267 | T | T | T | T | aspartic-type endopeptidase activity | CARP1\_CANAL | CARP2\_CANAX | CARP\_RHIPU | CATD\_RAT | CHYM\_CAMDR | PEPA\_ASPPH | RENI\_RAT | GO:0042973 | 0.00243836358710377 | 9/3691 | 7/641 | 0.000120485877943708 | 0.0483148370554267 | T | T | T | T | glucan endo-1,3-beta-D-glucosidase activity | A2TM14\_HEVBR | D1M8S7\_HEVBR | E13B\_HORVU | E13C\_MUSAC | ENG1\_RHIMI | Q70C53\_SOLTU | TLP\_PRUAV | GO:0036393 | 0.00135464643727987 | 5/3691 | 5/641 | 0.000155939399020533 | 0.0625316990072339 | T | T | T | T | thiocyanate peroxidase activity | L8ICE9\_9CETA | PERL\_BOVIN | PERL\_BUBBU | PERL\_CAPHI | POXA\_DICDI | GO:0003993 | 0.00487672717420753 | 18/3691 | 10/641 | 0.00026752143047962 | 0.107276093622328 | T | T | T | T | acid phosphatase activity | A2TBB4\_9ASCO | F6MIW5\_WHEAT | PHYA\_ASPFU | PPA5\_HUMAN | PPA5\_PIG | PPA5\_RAT | PPAF1\_IPOBA | PPAF\_PHAVU | PPAP\_RAT | Q8VX11\_LUPLU | GO:0004222 | 0.00487672717420753 | 18/3691 | 10/641 | 0.00026752143047962 | 0.107276093622328 | T | T | T | T | metalloendopeptidase activity | B4F320\_LIMPO | HE12\_DANRE | KLK7\_HUMAN | MMP1\_PIG | VM11\_BOTMO | VM12\_CROAD | VM1A3\_DEIAC | VM1BI\_BOTMO | VM1T1\_PROMU | VM1T2\_PROFL | GO:0005102 | 0.0132755350853427 | 49/3691 | 19/641 | 0.000287525233564103 | 0.115297618659205 | T | T | T | T | signaling receptor binding | ADA2\_HUMAN | AGAL\_HUMAN | ANG1\_BOVIN | ANG2\_MOUSE | ANG3\_MOUSE | ANG4\_MOUSE | ANGI\_MOUSE | BGLR\_HUMAN | GBA1\_HUMAN | IDUA\_HUMAN | PA21B\_BOVIN | PA21B\_PIG | PRTN3\_HUMAN | Q5WRG2\_RAT | Q9PTT3\_SPAAU | RENI\_RAT | TTHY\_CHICK | TTHY\_MOUSE | TTHY\_RAT | GO:0003779 | 0.00270929287455974 | 10/3691 | 7/641 | 0.000341459412225271 | 0.136925224302334 | T | T | T | T | actin binding | ANG1\_BOVIN | ANG2\_MOUSE | ANG3\_MOUSE | ANG4\_MOUSE | ANGI\_MOUSE | DNAS1\_HUMAN | Q5WRG2\_RAT | GO:0004601 | 0.0373882416689244 | 138/3691 | 40/641 | 0.000401673408620525 | 0.161071036856831 | T | T | T | T | peroxidase activity | A0A087WNH2\_FICBE | A0A0A0Y4H8\_TRAFO | A0A1S4NYF8\_PANVG | A0A1Y2TH07\_9PEZI | A0A2P1C6N4\_IRPLA | A0A3L6SKP5\_PANMI | APO1\_CYCAE | CAT3\_NEUCR | D1MPT2\_ROYRE | DYP\_AURAJ | GPX3\_HUMAN | GPX5\_HUMAN | GPX6\_MOUSE | K7N5L9\_RAPSA | K7ZUA3\_ASCNO | KATG2\_MAGO7 | L8ICE9\_9CETA | LIG2\_PHACH | LIG4\_PHACH | LIG8\_PHACH | O22443\_SOYBN | PEM1\_PHACH | PER1A\_ARMRU | PER1\_ARAHY | PER1\_SORBI | PER53\_ARATH | PER59\_ARATH | PERL\_BOVIN | PERL\_BUBBU | PERL\_CAPHI | PER\_ARTRA | PER\_COPCI | PGH2\_HUMAN | POXA\_DICDI | PRXC\_LEPFU | Q40069\_HORVU | Q60FD2\_9APHY | Q8WZK8\_9APHY | VPL1\_PLEER | VPL2\_PLEER | GO:0016684 | 0.0373882416689244 | 138/3691 | 40/641 | 0.000401673408620525 | 0.161071036856831 | T | T | T | T | oxidoreductase activity, acting on peroxide as acceptor | A0A087WNH2\_FICBE | A0A0A0Y4H8\_TRAFO | A0A1S4NYF8\_PANVG | A0A1Y2TH07\_9PEZI | A0A2P1C6N4\_IRPLA | A0A3L6SKP5\_PANMI | APO1\_CYCAE | CAT3\_NEUCR | D1MPT2\_ROYRE | DYP\_AURAJ | GPX3\_HUMAN | GPX5\_HUMAN | GPX6\_MOUSE | K7N5L9\_RAPSA | K7ZUA3\_ASCNO | KATG2\_MAGO7 | L8ICE9\_9CETA | LIG2\_PHACH | LIG4\_PHACH | LIG8\_PHACH | O22443\_SOYBN | PEM1\_PHACH | PER1A\_ARMRU | PER1\_ARAHY | PER1\_SORBI | PER53\_ARATH | PER59\_ARATH | PERL\_BOVIN | PERL\_BUBBU | PERL\_CAPHI | PER\_ARTRA | PER\_COPCI | PGH2\_HUMAN | POXA\_DICDI | PRXC\_LEPFU | Q40069\_HORVU | Q60FD2\_9APHY | Q8WZK8\_9APHY | VPL1\_PLEER | VPL2\_PLEER | GO:0015929 | 0.00216743429964779 | 8/3691 | 6/641 | 0.00054758538094909 | 0.219581737760585 | T | T | T | T | hexosaminidase activity | ANAG\_HUMAN | CHIL3\_MOUSE | HEXC\_OSTFU | HYAL1\_HUMAN | NAGAB\_CHICK | NAGAB\_HUMAN | GO:0030600 | 0.00162557572473584 | 6/3691 | 5/641 | 0.000801103760133867 | 0.321242607813681 | T | T | T | T | feruloyl esterase activity | A0A1D3S5H0\_FUSOX | A2QIR3\_ASPNC | FAEA\_ASPNG | FAEB1\_ASPOR | FAEB2\_ASPOR | GO:0005179 | 0.0010837171498239 | 4/3691 | 4/641 | 0.000902588012855664 | 0.361937793155121 | T | T | T | T | hormone activity | Q9PTT3\_SPAAU | TTHY\_CHICK | TTHY\_MOUSE | TTHY\_RAT | GO:0030570 | 0.0010837171498239 | 4/3691 | 4/641 | 0.000902588012855664 | 0.361937793155121 | T | T | T | T | pectate lyase activity | G2X3Y1\_VERDV | PELA\_ASPNG | PELB\_ASPNG | PLY1\_JUNAS | GO:0004461 | 0.0010837171498239 | 4/3691 | 4/641 | 0.000902588012855664 | 0.361937793155121 | T | T | T | T | lactose synthase activity | LALBA\_BOVIN | LALBA\_CAPHI | LALBA\_CAVPO | LALBA\_PAPCY | GO:0051381 | 0.0010837171498239 | 4/3691 | 4/641 | 0.000902588012855664 | 0.361937793155121 | T | T | T | T | histamine binding | NP1\_RHOPR | NP2\_RHOPR | NP4\_RHOPR | Q86PT9\_RHOPR | GO:0004522 | 0.0010837171498239 | 4/3691 | 4/641 | 0.000902588012855664 | 0.361937793155121 | T | T | T | T | ribonuclease A activity | RNAS1\_BISBI | RNAS4\_HUMAN | RNS1B\_RAT | RNS\_BOVIN | GO:0016799 | 0.0140883229477106 | 52/3691 | 18/641 | 0.0019524247466888 | 0.782922323422209 | T | T | T | F | hydrolase activity, hydrolyzing N-glycosyl compounds | E0CX04\_MOMBA | NADA\_APLCA | NADA\_ASPFU | O04358\_IRIHO | Q2QEH4\_SAPOF | Q94BW3\_CINCA | RIP0\_DIACA | RIP1\_BRYDI | RIP1\_HORVU | RIP1\_MOMCH | RIP1\_PHYAM | RIP2\_PHYAM | RIP3\_MOMCH | RIPA\_PHYAM | RIPG\_SURMU | RIPL1\_PHYDI | RIPL2\_PHYDI | RIPT\_TRIKI | GO:0042277 | 0.00514765646166351 | 19/3691 | 9/641 | 0.00239994712871343 | 0.962378798614085 | T | T | T | F | peptide binding | ANG2\_MOUSE | ANG3\_MOUSE | ANG4\_MOUSE | ANGI\_MOUSE | CATD\_RAT | CYP5\_CAEEL | PGPSA\_DROME | Q5WRG2\_RAT | TPP1\_HUMAN | GO:0016603 | 0.00189650501219182 | 7/3691 | 5/641 | 0.00240254701959248 | 0.963421354856583 | T | T | T | F | glutaminyl-peptide cyclotransferase activity | O81226\_CARPA | QPCT1\_DROME | QPCT2\_DROME | QPCT\_IXOSC | QPCT\_MOUSE | GO:0047498 | 0.00189650501219182 | 7/3691 | 5/641 | 0.00240254701959248 | 0.963421354856583 | T | T | T | F | calcium-dependent phospholipase A2 activity | PA21B\_BOVIN | PA21B\_PIG | PA2GA\_HUMAN | PA2GE\_HUMAN | PA2GX\_HUMAN | GO:0016160 | 0.00433486859929558 | 16/3691 | 8/641 | 0.00275582119538173 | 1 | T | T | T | F | amylase activity | A0A173N065\_EISFE | AMY1A\_HUMAN | AMY1\_HORVU | AMY1\_ORYSJ | AMYA1\_ASPOR | AMY\_ORYLA | K9L8F3\_MALCI | M9TI89\_RHIPU | GO:0020037 | 0.0341370902194527 | 126/3691 | 34/641 | 0.00402213764010527 | 1 | T | T | F | F | heme binding | A0A087WNH2\_FICBE | A0A0A0Y4H8\_TRAFO | A0A1S4NYF8\_PANVG | A0A2P1C6N4\_IRPLA | A0A3L6SKP5\_PANMI | APO1\_CYCAE | CAT3\_NEUCR | D1MPT2\_ROYRE | DYP\_AURAJ | K7N5L9\_RAPSA | KATG2\_MAGO7 | L8ICE9\_9CETA | LIG2\_PHACH | LIG4\_PHACH | LIG8\_PHACH | O22443\_SOYBN | PEM1\_PHACH | PER1A\_ARMRU | PER1\_ARAHY | PER1\_SORBI | PER53\_ARATH | PER59\_ARATH | PERL\_BOVIN | PERL\_BUBBU | PERL\_CAPHI | PER\_ARTRA | PER\_COPCI | PGH2\_HUMAN | POXA\_DICDI | Q40069\_HORVU | Q60FD2\_9APHY | Q8WZK8\_9APHY | VPL1\_PLEER | VPL2\_PLEER | GO:0015066 | 0.000812787862367922 | 3/3691 | 3/641 | 0.00521746801161791 | 1 | T | T | F | F | alpha-amylase inhibitor activity | B2ZGS7\_9ASPA | D9MWI4\_9ASPA | E3VTL0\_9ASPA | GO:0030599 | 0.000812787862367922 | 3/3691 | 3/641 | 0.00521746801161791 | 1 | T | T | F | F | pectinesterase activity | G3YAL0\_ASPNA | PME\_DAUCA | PME\_SITOR | GO:0120146 | 0.000812787862367922 | 3/3691 | 3/641 | 0.00521746801161791 | 1 | T | T | F | F | sulfatide binding | PPT1\_BOVIN | PPT1\_HUMAN | TPP1\_HUMAN | GO:0004099 | 0.000812787862367922 | 3/3691 | 3/641 | 0.00521746801161791 | 1 | T | T | F | F | chitin deacetylase activity | A2QZC8\_ASPNC | CDA\_COLLN | CDA\_EMENI | GO:0016690 | 0.000812787862367922 | 3/3691 | 3/641 | 0.00521746801161791 | 1 | T | T | F | F | diarylpropane peroxidase activity | LIG2\_PHACH | LIG4\_PHACH | LIG8\_PHACH | GO:0070492 | 0.000812787862367922 | 3/3691 | 3/641 | 0.00521746801161791 | 1 | T | T | F | F | oligosaccharide binding | BGALA\_ASPNC | MANA\_CRYAT | MANA\_HYPJR | GO:0004338 | 0.000812787862367922 | 3/3691 | 3/641 | 0.00521746801161791 | 1 | T | T | F | F | glucan exo-1,3-beta-glucosidase activity | EXG1\_CANAL | EXG1\_YEAST | Q2Z1W1\_PHACH | GO:0016158 | 0.000812787862367922 | 3/3691 | 3/641 | 0.00521746801161791 | 1 | T | T | F | F | 3-phytase activity | PHYA\_ASPFU | PHYA\_ASPNG | PHYB\_ASPAW | GO:0016689 | 0.000812787862367922 | 3/3691 | 3/641 | 0.00521746801161791 | 1 | T | T | F | F | manganese peroxidase activity | PEM1\_PHACH | VPL1\_PLEER | VPL2\_PLEER | GO:0045330 | 0.000812787862367922 | 3/3691 | 3/641 | 0.00521746801161791 | 1 | T | T | F | F | aspartyl esterase activity | G3YAL0\_ASPNA | PME\_DAUCA | PME\_SITOR | GO:0070324 | 0.00216743429964779 | 8/3691 | 5/641 | 0.00549414975066085 | 1 | T | T | F | F | thyroid hormone binding | CATH\_HUMAN | Q9PTT3\_SPAAU | TTHY\_CHICK | TTHY\_MOUSE | TTHY\_RAT | GO:0046872 | 0.338661609319967 | 1250/3691 | 245/641 | 0.00617942273781692 | 1 | T | T | F | F | metal ion binding | A0A075B5H6\_TRIHA | A0A087WNH2\_FICBE | A0A0A0Y4H8\_TRAFO | A0A0A7M685\_TRAHI | A0A0M3U1T9\_9APHY | A0A0R4I979\_BRABE | A0A0S2GKZ1\_9APHY | A0A173N065\_EISFE | A0A183C5H8\_GLOPA | A0A1D3S5H0\_FUSOX | A0A1L8D5Z7\_BOTAT | A0A1S4NYF8\_PANVG | A0A1Y2TH07\_9PEZI | A0A2H4A2Q2\_9APHY | A0A3F2YLU5\_9APHY | A0A3L6SKP5\_PANMI | A0NFU8\_ANOGA | A1HA\_LOXIN | A1HB2\_LOXIN | A2QIR3\_ASPNC | A2QS62\_ASPNC | A311\_LOXLA | ADA2\_HUMAN | AMY1A\_HUMAN | AMY1\_HORVU | AMY1\_ORYSJ | AMYA1\_ASPOR | AMY\_ORYLA | ANG1\_BOVIN | ANG2\_MOUSE | ANG3\_MOUSE | ANG4\_MOUSE | ANGI\_MOUSE | AOAH\_MOUSE | AOC1\_HUMAN | AOCX\_BOVIN | APO1\_CYCAE | ASM3A\_HUMAN | ASM3A\_MOUSE | B2L9C1\_TRAHI | B4F320\_LIMPO | B7X9Z0\_COPCI | BLRO\_ALBVE | CAH1\_CHLRE | CAH6\_HUMAN | CAT3\_NEUCR | CBPA1\_PIG | CBPD\_LOPSP | CBPN\_HUMAN | CDA\_COLLN | CDA\_EMENI | CERU\_RAT | CHIT\_PUNGR | D1MPT2\_ROYRE | D7F485\_9APHY | DABA\_PSEMU | DNSL3\_HUMAN | DOPO\_HUMAN | DYP\_AURAJ | ENDO2\_ARATH | ENPP2\_HUMAN | ENPP2\_RAT | F2Z7L1\_9ANNE | F6MIW5\_WHEAT | F6N9E7\_9PEZI | FAEB1\_ASPOR | FAEB2\_ASPOR | G2QG31\_MYCTT | GALNS\_HUMAN | GAOA\_GIBZA | GOOX\_SARSR | GRASS\_DROME | GUN7\_HYPJQ | H1AE14\_PHACH | H8ZRU2\_9HELO | HE12\_DANRE | I1SB14\_9APHY | I1SB18\_VIPAE | I1VE66\_9APHY | I2FI81\_EISFE | IDH\_OSTTA | K7CID1\_PANTR | K7N5L9\_RAPSA | K9L8F3\_MALCI | KATG2\_MAGO7 | L8ICE9\_9CETA | LAC1\_MELAO | LAC1\_TRAMX | LAC2\_TRAVE | LALBA\_BOVIN | LALBA\_CAPHI | LALBA\_CAVPO | LALBA\_PAPCY | LAPA\_ASPOR | LIG2\_PHACH | LIG4\_PHACH | LIG8\_PHACH | LIPP\_HORSE | LIPR1\_CANLF | LIPR1\_HUMAN | LIPR2\_HUMAN | LIPR2\_RAT | LYSC1\_CANLF | LYSC1\_HORSE | LYSC\_EQUAS | M1GME7\_9APHY | M9TI89\_RHIPU | MAN12\_PENCI | MANA\_CANEN | MMP1\_PIG | MNCO\_MICNN | MNLOX\_MAGO7 | NADA\_ASPFU | NP1\_RHOPR | NP2\_RHOPR | NP4\_RHOPR | NUP1\_PENCI | NUS1\_ASPOR | O22443\_SOYBN | O77044\_9NEOP | O81226\_CARPA | O97389\_HELAM | OXLA\_BOTAT | PA21B\_BOVIN | PA21B\_PIG | PA2A1\_BUNCE | PA2A1\_ECHCA | PA2A1\_NAJAT | PA2A1\_OPHHA | PA2A2\_NAJNA | PA2A2\_OPHHA | PA2A2\_TROCA | PA2A4\_NAJSG | PA2A5\_TRIST | PA2A7\_GLOHA | PA2A\_BOTJR | PA2A\_CROAT | PA2A\_DEIAC | PA2A\_GLOHA | PA2A\_NAJAT | PA2B1\_AGKPI | PA2B2\_BOTJR | PA2B2\_PROFL | PA2B3\_BOTAS | PA2B3\_BUNCE | PA2B5\_BUNCE | PA2B5\_NOTSC | PA2BA\_VIPAA | PA2BB\_GLOHA | PA2BB\_PSEAU | PA2BC\_VIPAA | PA2BD\_CRODU | PA2B\_BUNCE | PA2B\_NOTSC | PA2GA\_HUMAN | PA2GE\_HUMAN | PA2GX\_HUMAN | PA2H1\_AGKCL | PA2H1\_BOTBZ | PA2H1\_BOTJR | PA2H1\_BOTMO | PA2H1\_BOTPI | PA2H2\_BOTAS | PA2H2\_BOTMO | PA2H2\_BOTPI | PA2H2\_CERGO | PA2H3\_BOTPI | PA2HB\_AGKPI | PA2HB\_OXYSC | PA2HH\_TRIST | PA2HS\_ECHCA | PA2H\_BOTPA | PA2H\_DEIAC | PA2H\_PROMB | PA2N\_GLOHA | PA2\_APIME | PAG15\_HUMAN | PDE\_NAJAT | PEM1\_PHACH | PER1A\_ARMRU | PER1\_ARAHY | PER1\_SORBI | PER53\_ARATH | PER59\_ARATH | PERL\_BOVIN | PERL\_BUBBU | PERL\_CAPHI | PER\_ARTRA | PER\_COPCI | PGH2\_HUMAN | PGPSA\_DROME | PGRP1\_CAMDR | PLA22\_ORYSJ | PLY1\_JUNAS | PPA5\_HUMAN | PPA5\_PIG | PPA5\_RAT | PPAF1\_HOLDI | PPAF1\_IPOBA | PPAF\_PHAVU | PPO8\_ANOGA | PRXC\_LEPFU | Q07524\_TROMA | Q08J22\_BOMMO | Q0KFV0\_SOLLC | Q12571\_9BASI | Q1K4Q1\_NEUCR | Q1W6B1\_9APHY | Q2TWF5\_ASPOR | Q40069\_HORVU | Q5B038\_EMENI | Q5EBY5\_9APHY | Q5WRG2\_RAT | Q60FD2\_9APHY | Q6H9H7\_9APHY | Q86RS6\_MANSE | Q8TG94\_TRAPU | Q8VX11\_LUPLU | Q8WZK8\_9APHY | Q92458\_HYPJE | Q95KP4\_HORSE | Q96TR6\_PYCCO | Q96UT7\_TRAVE | Q96X16\_PICPA | Q9HDQ0\_9APHY | Q9UVQ2\_PYCCI | Q9Y780\_COPCI | QPCT1\_DROME | QPCT2\_DROME | QPCT\_IXOSC | QPCT\_MOUSE | SODE\_ONCVO | TPP1\_HUMAN | TRFL\_BUBBU | TRFL\_HORSE | TRY3\_SALSA | V5NTD\_NAJAT | VM11\_BOTMO | VM12\_CROAD | VM1A3\_DEIAC | VM1BI\_BOTMO | VM1T1\_PROMU | VM1T2\_PROFL | VPL1\_PLEER | VPL2\_PLEER | GO:0046906 | 0.0354917366567326 | 131/3691 | 34/641 | 0.00773768337898399 | 1 | T | T | F | F | tetrapyrrole binding | A0A087WNH2\_FICBE | A0A0A0Y4H8\_TRAFO | A0A1S4NYF8\_PANVG | A0A2P1C6N4\_IRPLA | A0A3L6SKP5\_PANMI | APO1\_CYCAE | CAT3\_NEUCR | D1MPT2\_ROYRE | DYP\_AURAJ | K7N5L9\_RAPSA | KATG2\_MAGO7 | L8ICE9\_9CETA | LIG2\_PHACH | LIG4\_PHACH | LIG8\_PHACH | O22443\_SOYBN | PEM1\_PHACH | PER1A\_ARMRU | PER1\_ARAHY | PER1\_SORBI | PER53\_ARATH | PER59\_ARATH | PERL\_BOVIN | PERL\_BUBBU | PERL\_CAPHI | PER\_ARTRA | PER\_COPCI | PGH2\_HUMAN | POXA\_DICDI | Q40069\_HORVU | Q60FD2\_9APHY | Q8WZK8\_9APHY | VPL1\_PLEER | VPL2\_PLEER | GO:0035250 | 0.00162557572473584 | 6/3691 | 4/641 | 0.0100653398843156 | 1 | T | T | F | F | UDP-galactosyltransferase activity | LALBA\_BOVIN | LALBA\_CAPHI | LALBA\_CAVPO | LALBA\_PAPCY | GO:0008378 | 0.00162557572473584 | 6/3691 | 4/641 | 0.0100653398843156 | 1 | T | T | F | F | galactosyltransferase activity | LALBA\_BOVIN | LALBA\_CAPHI | LALBA\_CAVPO | LALBA\_PAPCY | GO:0070026 | 0.00162557572473584 | 6/3691 | 4/641 | 0.0100653398843156 | 1 | T | T | F | F | nitric oxide binding | NP1\_RHOPR | NP2\_RHOPR | NP4\_RHOPR | Q86PT9\_RHOPR | GO:0102483 | 0.00541858574911948 | 20/3691 | 8/641 | 0.014120069461014 | 1 | T | T | F | F | scopolin beta-glucosidase activity | A0A068FT77\_9PEZI | BGL1\_ASPAC | BGLA\_ASPFU | BGLA\_ASPOR | Q12715\_HYPJE | Q7RWP2\_NEUCR | Q8T0W7\_9NEOP | Q8TGI8\_TALEM | GO:0043169 | 0.347331346518559 | 1282/3691 | 247/641 | 0.0151125785130647 | 1 | T | T | F | F | cation binding | A0A075B5H6\_TRIHA | A0A087WNH2\_FICBE | A0A0A0Y4H8\_TRAFO | A0A0A7M685\_TRAHI | A0A0M3U1T9\_9APHY | A0A0R4I979\_BRABE | A0A0S2GKZ1\_9APHY | A0A173N065\_EISFE | A0A183C5H8\_GLOPA | A0A1D3S5H0\_FUSOX | A0A1L8D5Z7\_BOTAT | A0A1S4NYF8\_PANVG | A0A1Y2TH07\_9PEZI | A0A2H4A2Q2\_9APHY | A0A3F2YLU5\_9APHY | A0A3L6SKP5\_PANMI | A0NFU8\_ANOGA | A1HA\_LOXIN | A1HB2\_LOXIN | A2QIR3\_ASPNC | A2QS62\_ASPNC | A311\_LOXLA | ADA2\_HUMAN | AMY1A\_HUMAN | AMY1\_HORVU | AMY1\_ORYSJ | AMYA1\_ASPOR | AMY\_ORYLA | ANG1\_BOVIN | ANG2\_MOUSE | ANG3\_MOUSE | ANG4\_MOUSE | ANGI\_MOUSE | AOAH\_MOUSE | AOC1\_HUMAN | AOCX\_BOVIN | APO1\_CYCAE | ASM3A\_HUMAN | ASM3A\_MOUSE | B2L9C1\_TRAHI | B4F320\_LIMPO | B7X9Z0\_COPCI | BLRO\_ALBVE | CAH1\_CHLRE | CAH6\_HUMAN | CAT3\_NEUCR | CBPA1\_PIG | CBPD\_LOPSP | CBPN\_HUMAN | CDA\_COLLN | CDA\_EMENI | CERU\_RAT | CHIT\_PUNGR | D1MPT2\_ROYRE | D7F485\_9APHY | DABA\_PSEMU | DNSL3\_HUMAN | DOPO\_HUMAN | DYP\_AURAJ | ENDO2\_ARATH | ENPP2\_HUMAN | ENPP2\_RAT | F2Z7L1\_9ANNE | F6MIW5\_WHEAT | F6N9E7\_9PEZI | FAEB1\_ASPOR | FAEB2\_ASPOR | G2QG31\_MYCTT | GALNS\_HUMAN | GAOA\_GIBZA | GOOX\_SARSR | GRASS\_DROME | GUN7\_HYPJQ | H1AE14\_PHACH | H8ZRU2\_9HELO | HE12\_DANRE | I1SB14\_9APHY | I1SB18\_VIPAE | I1VE66\_9APHY | I2FI81\_EISFE | IDH\_OSTTA | K7CID1\_PANTR | K7N5L9\_RAPSA | K9L8F3\_MALCI | KATG2\_MAGO7 | L8ICE9\_9CETA | LAC1\_MELAO | LAC1\_TRAMX | LAC2\_TRAVE | LALBA\_BOVIN | LALBA\_CAPHI | LALBA\_CAVPO | LALBA\_PAPCY | LAPA\_ASPOR | LIG2\_PHACH | LIG4\_PHACH | LIG8\_PHACH | LIPP\_HORSE | LIPR1\_CANLF | LIPR1\_HUMAN | LIPR2\_HUMAN | LIPR2\_RAT | LYSC1\_CANLF | LYSC1\_HORSE | LYSC\_EQUAS | M1GME7\_9APHY | M9TI89\_RHIPU | MAN12\_PENCI | MANA\_CANEN | MMP1\_PIG | MNCO\_MICNN | MNLOX\_MAGO7 | NADA\_ASPFU | NP1\_RHOPR | NP2\_RHOPR | NP4\_RHOPR | NUP1\_PENCI | NUS1\_ASPOR | O22443\_SOYBN | O77044\_9NEOP | O81226\_CARPA | O97389\_HELAM | OXLA\_BOTAT | PA21B\_BOVIN | PA21B\_PIG | PA2A1\_BUNCE | PA2A1\_ECHCA | PA2A1\_NAJAT | PA2A1\_OPHHA | PA2A2\_NAJNA | PA2A2\_OPHHA | PA2A2\_TROCA | PA2A4\_NAJSG | PA2A5\_TRIST | PA2A7\_GLOHA | PA2A\_BOTJR | PA2A\_CROAT | PA2A\_DEIAC | PA2A\_GLOHA | PA2A\_NAJAT | PA2B1\_AGKPI | PA2B2\_BOTJR | PA2B2\_PROFL | PA2B3\_BOTAS | PA2B3\_BUNCE | PA2B5\_BUNCE | PA2B5\_NOTSC | PA2BA\_VIPAA | PA2BB\_GLOHA | PA2BB\_PSEAU | PA2BC\_VIPAA | PA2BD\_CRODU | PA2B\_BUNCE | PA2B\_NOTSC | PA2GA\_HUMAN | PA2GE\_HUMAN | PA2GX\_HUMAN | PA2H1\_AGKCL | PA2H1\_BOTBZ | PA2H1\_BOTJR | PA2H1\_BOTMO | PA2H1\_BOTPI | PA2H2\_BOTAS | PA2H2\_BOTMO | PA2H2\_BOTPI | PA2H2\_CERGO | PA2H3\_BOTPI | PA2HB\_AGKPI | PA2HB\_OXYSC | PA2HH\_TRIST | PA2HS\_ECHCA | PA2H\_BOTPA | PA2H\_DEIAC | PA2H\_PROMB | PA2N\_GLOHA | PA2\_APIME | PAG15\_HUMAN | PDE\_NAJAT | PEM1\_PHACH | PER1A\_ARMRU | PER1\_ARAHY | PER1\_SORBI | PER53\_ARATH | PER59\_ARATH | PERL\_BOVIN | PERL\_BUBBU | PERL\_CAPHI | PER\_ARTRA | PER\_COPCI | PGH2\_HUMAN | PGPSA\_DROME | PGRP1\_CAMDR | PLA22\_ORYSJ | PLY1\_JUNAS | PPA5\_HUMAN | PPA5\_PIG | PPA5\_RAT | PPAF1\_HOLDI | PPAF1\_IPOBA | PPAF\_PHAVU | PPAP\_RAT | PPO8\_ANOGA | PRXC\_LEPFU | Q07524\_TROMA | Q08J22\_BOMMO | Q0KFV0\_SOLLC | Q12571\_9BASI | Q1K4Q1\_NEUCR | Q1W6B1\_9APHY | Q2TWF5\_ASPOR | Q40069\_HORVU | Q5B038\_EMENI | Q5EBY5\_9APHY | Q5WRG2\_RAT | Q60FD2\_9APHY | Q6H9H7\_9APHY | Q86PT9\_RHOPR | Q86RS6\_MANSE | Q8TG94\_TRAPU | Q8VX11\_LUPLU | Q8WZK8\_9APHY | Q92458\_HYPJE | Q95KP4\_HORSE | Q96TR6\_PYCCO | Q96UT7\_TRAVE | Q96X16\_PICPA | Q9HDQ0\_9APHY | Q9UVQ2\_PYCCI | Q9Y780\_COPCI | QPCT1\_DROME | QPCT2\_DROME | QPCT\_IXOSC | QPCT\_MOUSE | SODE\_ONCVO | TPP1\_HUMAN | TRFL\_BUBBU | TRFL\_HORSE | TRY3\_SALSA | V5NTD\_NAJAT | VM11\_BOTMO | VM12\_CROAD | VM1A3\_DEIAC | VM1BI\_BOTMO | VM1T1\_PROMU | VM1T2\_PROFL | VPL1\_PLEER | VPL2\_PLEER | GO:0004565 | 0.00352208073692766 | 13/3691 | 6/641 | 0.0152812993314463 | 1 | T | T | F | F | beta-galactosidase activity | BGALA\_ASPNC | BGALA\_ASPOR | BGALA\_PENSQ | O81100\_SOLLC | Q50KB2\_PHACH | Q70SY0\_HYPJE | GO:0004180 | 0.00460579788675156 | 17/3691 | 7/641 | 0.018031685467733 | 1 | T | T | F | F | carboxypeptidase activity | A0NFU8\_ANOGA | CBPA1\_PIG | CBPD\_LOPSP | CBPN\_HUMAN | O97389\_HELAM | PCP\_HUMAN | PGPSA\_DROME | GO:0051861 | 0.0010837171498239 | 4/3691 | 3/641 | 0.0181621080078996 | 1 | T | T | F | F | glycolipid binding | PPT1\_BOVIN | PPT1\_HUMAN | TPP1\_HUMAN | GO:0004564 | 0.0010837171498239 | 4/3691 | 3/641 | 0.0181621080078996 | 1 | T | T | F | F | beta-fructofuranosidase activity | A0A6F8Z6Y2\_BOMMO | E5D0X5\_SCHOC | INV\_SCHOC | GO:0004308 | 0.0010837171498239 | 4/3691 | 3/641 | 0.0181621080078996 | 1 | T | T | F | F | exo-alpha-sialidase activity | NANL\_MACDE | O44049\_TRYRA | SIA\_ASPFU | GO:0038024 | 0.0010837171498239 | 4/3691 | 3/641 | 0.0181621080078996 | 1 | T | T | F | F | cargo receptor activity | ENPP2\_HUMAN | ENPP2\_RAT | K7CID1\_PANTR | GO:0016997 | 0.0010837171498239 | 4/3691 | 3/641 | 0.0181621080078996 | 1 | T | T | F | F | alpha-sialidase activity | NANL\_MACDE | O44049\_TRYRA | SIA\_ASPFU | GO:0031218 | 0.0010837171498239 | 4/3691 | 3/641 | 0.0181621080078996 | 1 | T | T | F | F | arabinogalactan endo-1,4-beta-galactosidase activity | GANA\_ASPAC | GANA\_EMENI | GANA\_HUMIN | GO:0005044 | 0.0010837171498239 | 4/3691 | 3/641 | 0.0181621080078996 | 1 | T | T | F | F | scavenger receptor activity | ENPP2\_HUMAN | ENPP2\_RAT | K7CID1\_PANTR | GO:0140096 | 0.105391492820374 | 389/3691 | 83/641 | 0.0189056839839763 | 1 | T | T | F | F | catalytic activity, acting on a protein | A0A0R3QSA7\_9BILA | A0A3S5H5N2\_LEIDO | A0A6P6YAT6\_DERPT | A0NFU8\_ANOGA | A4GX63\_TOXGO | B4F320\_LIMPO | CARP1\_CANAL | CARP2\_CANAX | CARP\_RHIPU | CATD\_RAT | CATH\_HUMAN | CATLL\_FASHE | CBPA1\_PIG | CBPD\_LOPSP | CBPN\_HUMAN | CEL2A\_PIG | CFAD\_MOUSE | CHYM\_CAMDR | COGS\_HYPLI | CUCM1\_CUCME | CYP5\_CAEEL | CYSP\_BLOTA | D6XHE1\_TRYB2 | DDN1\_BOVIN | DPP2\_HUMAN | EGFB2\_MOUSE | ERVB\_TABDI | G3I1H5\_CRIGR | GGH\_HUMAN | GRAA\_HUMAN | GRAC\_MOUSE | GRAK\_HUMAN | GRASS\_DROME | HE12\_DANRE | J7LCB0\_DEIAC | KLK10\_HUMAN | KLK1\_HUMAN | KLK2\_HORSE | KLK2\_HUMAN | KLK7\_HUMAN | KLK7\_MOUSE | KLK8\_MOUSE | LAPA\_ASPOR | LGMN\_MOUSE | LYS\_RUDPH | MCPT2\_RAT | MMP1\_PIG | O81226\_CARPA | O97389\_HELAM | OFUT1\_CAEEL | PCP\_HUMAN | PEPA\_ASPPH | PGPSA\_DROME | PPAF1\_HOLDI | PPAP\_RAT | PPT1\_BOVIN | PPT1\_HUMAN | PRS57\_HUMAN | PRTN3\_HUMAN | Q06AK3\_TOXGO | Q69G21\_TENMO | Q6NY42\_DANRE | Q6R7Z5\_9TRYP | Q7YXL2\_TENMO | QPCT1\_DROME | QPCT2\_DROME | QPCT\_IXOSC | QPCT\_MOUSE | RENI\_RAT | TPP1\_HUMAN | TRFL\_BUBBU | TRFL\_HORSE | TRY1\_GADMO | TRY3\_SALSA | TRYB2\_HUMAN | VM11\_BOTMO | VM12\_CROAD | VM1A3\_DEIAC | VM1BI\_BOTMO | VM1T1\_PROMU | VM1T2\_PROFL | VSPP\_DEIAC | VSPSX\_GLOSA | GO:0005543 | 0.00568951503657545 | 21/3691 | 8/641 | 0.0194257264487057 | 1 | T | F | F | F | phospholipid binding | PA21B\_BOVIN | PA21B\_PIG | PA2GA\_HUMAN | PA2GE\_HUMAN | PA2GX\_HUMAN | PAG15\_HUMAN | PPT1\_HUMAN | TPP1\_HUMAN | GO:0008237 | 0.0151720400975345 | 56/3691 | 16/641 | 0.024958627684899 | 1 | T | F | F | F | metallopeptidase activity | A0NFU8\_ANOGA | B4F320\_LIMPO | CBPA1\_PIG | CBPD\_LOPSP | CBPN\_HUMAN | HE12\_DANRE | KLK7\_HUMAN | LAPA\_ASPOR | MMP1\_PIG | O97389\_HELAM | VM11\_BOTMO | VM12\_CROAD | VM1A3\_DEIAC | VM1BI\_BOTMO | VM1T1\_PROMU | VM1T2\_PROFL | GO:0008131 | 0.00487672717420753 | 18/3691 | 7/641 | 0.0251965166799598 | 1 | T | F | F | F | primary amine oxidase activity | AOC1\_HUMAN | AOCX\_BOVIN | OXLA\_BOTAT | OXLA\_CALRH | OXLA\_GLOHA | Q5B038\_EMENI | Q96X16\_PICPA | GO:0047490 | 0.000541858574911948 | 2/3691 | 2/641 | 0.0301208755788104 | 1 | T | F | F | F | pectin lyase activity | PELA\_ASPNG | PELB\_ASPNG | GO:0004549 | 0.000541858574911948 | 2/3691 | 2/641 | 0.0301208755788104 | 1 | T | F | F | F | tRNA-specific ribonuclease activity | ANGI\_MOUSE | Q5WRG2\_RAT | GO:0004339 | 0.000541858574911948 | 2/3691 | 2/641 | 0.0301208755788104 | 1 | T | F | F | F | glucan 1,4-alpha-glucosidase activity | AMYG\_SACFI | S7ZIW0\_PENO1 | GO:0047714 | 0.000541858574911948 | 2/3691 | 2/641 | 0.0301208755788104 | 1 | T | F | F | F | galactolipase activity | LIPR2\_HUMAN | LIPR2\_RAT | GO:0051669 | 0.000541858574911948 | 2/3691 | 2/641 | 0.0301208755788104 | 1 | T | F | F | F | fructan beta-fructosidase activity | INUE\_ASPAW | Q93X60\_CICIN | GO:0052739 | 0.000541858574911948 | 2/3691 | 2/641 | 0.0301208755788104 | 1 | T | F | F | F | phosphatidylserine 1-acylhydrolase activity | PA1\_VESBA | PAG15\_HUMAN | GO:0106329 | 0.000541858574911948 | 2/3691 | 2/641 | 0.0301208755788104 | 1 | T | F | F | F | L-phenylalaine oxidase activity | OXLA\_BOTAT | OXLA\_CALRH | GO:0004560 | 0.000541858574911948 | 2/3691 | 2/641 | 0.0301208755788104 | 1 | T | F | F | F | alpha-L-fucosidase activity | FUCO\_HUMAN | J9UN47\_GIBZA | GO:0070008 | 0.000541858574911948 | 2/3691 | 2/641 | 0.0301208755788104 | 1 | T | F | F | F | serine-type exopeptidase activity | PCP\_HUMAN | TPP1\_HUMAN | GO:0102549 | 0.000541858574911948 | 2/3691 | 2/641 | 0.0301208755788104 | 1 | T | F | F | F | 1-18:1-2-16:0-monogalactosyldiacylglycerol lipase activity | LIPR2\_HUMAN | LIPR2\_RAT | GO:0046555 | 0.000541858574911948 | 2/3691 | 2/641 | 0.0301208755788104 | 1 | T | F | F | F | acetylxylan esterase activity | AXE1\_ASPAW | AXE2\_TALPU | GO:0052740 | 0.000541858574911948 | 2/3691 | 2/641 | 0.0301208755788104 | 1 | T | F | F | F | 1-acyl-2-lysophosphatidylserine acylhydrolase activity | PA1\_VESBA | PAG15\_HUMAN | GO:0016762 | 0.000541858574911948 | 2/3691 | 2/641 | 0.0301208755788104 | 1 | T | F | F | F | xyloglucan:xyloglucosyl transferase activity | Q07524\_TROMA | XTH34\_POPPZ | GO:0052750 | 0.000541858574911948 | 2/3691 | 2/641 | 0.0301208755788104 | 1 | T | F | F | F | reactive-black-5:hydrogen-peroxide oxidoreductase activity | VPL1\_PLEER | VPL2\_PLEER | GO:0008456 | 0.000541858574911948 | 2/3691 | 2/641 | 0.0301208755788104 | 1 | T | F | F | F | alpha-N-acetylgalactosaminidase activity | NAGAB\_CHICK | NAGAB\_HUMAN | GO:0008843 | 0.000541858574911948 | 2/3691 | 2/641 | 0.0301208755788104 | 1 | T | F | F | F | endochitinase activity | CHI4\_CRYJA | CHIC\_ARATH | GO:0009044 | 0.000541858574911948 | 2/3691 | 2/641 | 0.0301208755788104 | 1 | T | F | F | F | xylan 1,4-beta-xylosidase activity | Q92458\_HYPJE | XYND\_EMENI | GO:0035727 | 0.000541858574911948 | 2/3691 | 2/641 | 0.0301208755788104 | 1 | T | F | F | F | lysophosphatidic acid binding | PPT1\_HUMAN | TPP1\_HUMAN | GO:0047391 | 0.000541858574911948 | 2/3691 | 2/641 | 0.0301208755788104 | 1 | T | F | F | F | alkylglycerophosphoethanolamine phosphodiesterase activity | ENPP2\_HUMAN | ENPP2\_RAT | GO:0034722 | 0.000541858574911948 | 2/3691 | 2/641 | 0.0301208755788104 | 1 | T | F | F | F | gamma-glutamyl-peptidase activity | GGH\_HUMAN | Q6NY42\_DANRE | GO:0042972 | 0.000541858574911948 | 2/3691 | 2/641 | 0.0301208755788104 | 1 | T | F | F | F | licheninase activity | E0XN39\_9EURO | GUB2\_HORVU | GO:0016892 | 0.00216743429964779 | 8/3691 | 4/641 | 0.0350706443238774 | 1 | T | F | F | F | RNA endonuclease activity, producing 3'-phosphomonoesters | RNAS1\_BISBI | RNAS4\_HUMAN | RNS1B\_RAT | RNS\_BOVIN | GO:0004181 | 0.00216743429964779 | 8/3691 | 4/641 | 0.0350706443238774 | 1 | T | F | F | F | metallocarboxypeptidase activity | CBPA1\_PIG | CBPD\_LOPSP | CBPN\_HUMAN | O97389\_HELAM | GO:0001716 | 0.00135464643727987 | 5/3691 | 3/641 | 0.0395714963174343 | 1 | T | F | F | F | L-amino-acid oxidase activity | OXLA\_BOTAT | OXLA\_CALRH | OXLA\_GLOHA | GO:0008745 | 0.00135464643727987 | 5/3691 | 3/641 | 0.0395714963174343 | 1 | T | F | F | F | N-acetylmuramoyl-L-alanine amidase activity | A0A0R4I979\_BRABE | PGRP1\_CAMDR | Q86RS6\_MANSE | GO:0004520 | 0.00243836358710377 | 9/3691 | 4/641 | 0.0546394746187242 | 1 | F | F | F | F | DNA endonuclease activity | DNAS1\_HUMAN | DNSL3\_HUMAN | ENDO2\_ARATH | Q0KFV0\_SOLLC | GO:0016894 | 0.00243836358710377 | 9/3691 | 4/641 | 0.0546394746187242 | 1 | F | F | F | F | endonuclease activity, active with either ribo- or deoxyribonucleic acids and producing 3'-phosphomonoesters | RNAS1\_BISBI | RNAS4\_HUMAN | RNS1B\_RAT | RNS\_BOVIN | GO:0008234 | 0.0116499593606069 | 43/3691 | 12/641 | 0.0572068952082312 | 1 | F | F | F | F | cysteine-type peptidase activity | CATH\_HUMAN | CATLL\_FASHE | CYSP\_BLOTA | D6XHE1\_TRYB2 | ERVB\_TABDI | G3I1H5\_CRIGR | GGH\_HUMAN | LGMN\_MOUSE | Q69G21\_TENMO | Q6NY42\_DANRE | Q6R7Z5\_9TRYP | Q7YXL2\_TENMO | GO:0042834 | 0.00162557572473584 | 6/3691 | 3/641 | 0.0690776527505828 | 1 | F | F | F | F | peptidoglycan binding | PGPSA\_DROME | PGRP1\_CAMDR | Q86RS6\_MANSE | GO:0016641 | 0.00596044432403143 | 22/3691 | 7/641 | 0.0722659395881394 | 1 | F | F | F | F | oxidoreductase activity, acting on the CH-NH2 group of donors, oxygen as acceptor | AOC1\_HUMAN | AOCX\_BOVIN | OXLA\_BOTAT | OXLA\_CALRH | OXLA\_GLOHA | Q5B038\_EMENI | Q96X16\_PICPA | GO:0046562 | 0.000812787862367922 | 3/3691 | 2/641 | 0.0799276907132 | 1 | F | F | F | F | glucose oxidase activity | GOX\_ASPNG | MNCO\_MICNN | GO:0005534 | 0.000812787862367922 | 3/3691 | 2/641 | 0.0799276907132 | 1 | F | F | F | F | galactose binding | BGALA\_ASPOR | BGALA\_PENSQ | GO:0052794 | 0.000812787862367922 | 3/3691 | 2/641 | 0.0799276907132 | 1 | F | F | F | F | exo-alpha-(2->3)-sialidase activity | O44049\_TRYRA | SIA\_ASPFU | GO:0102148 | 0.000812787862367922 | 3/3691 | 2/641 | 0.0799276907132 | 1 | F | F | F | F | N-acetyl-beta-D-galactosaminidase activity | CHIL3\_MOUSE | HEXC\_OSTFU | GO:0000014 | 0.000812787862367922 | 3/3691 | 2/641 | 0.0799276907132 | 1 | F | F | F | F | single-stranded DNA endodeoxyribonuclease activity | ENDO2\_ARATH | Q0KFV0\_SOLLC | GO:0015928 | 0.000812787862367922 | 3/3691 | 2/641 | 0.0799276907132 | 1 | F | F | F | F | fucosidase activity | FUCO\_HUMAN | J9UN47\_GIBZA | GO:0052796 | 0.000812787862367922 | 3/3691 | 2/641 | 0.0799276907132 | 1 | F | F | F | F | exo-alpha-(2->8)-sialidase activity | O44049\_TRYRA | SIA\_ASPFU | GO:0047979 | 0.000812787862367922 | 3/3691 | 2/641 | 0.0799276907132 | 1 | F | F | F | F | hexose oxidase activity | GOX\_ASPNG | MNCO\_MICNN | GO:0004563 | 0.000812787862367922 | 3/3691 | 2/641 | 0.0799276907132 | 1 | F | F | F | F | beta-N-acetylhexosaminidase activity | CHIL3\_MOUSE | HEXC\_OSTFU | GO:0052795 | 0.000812787862367922 | 3/3691 | 2/641 | 0.0799276907132 | 1 | F | F | F | F | exo-alpha-(2->6)-sialidase activity | O44049\_TRYRA | SIA\_ASPFU | GO:0052597 | 0.000812787862367922 | 3/3691 | 2/641 | 0.0799276907132 | 1 | F | F | F | F | diamine oxidase activity | AOC1\_HUMAN | Q96X16\_PICPA | GO:0004197 | 0.00514765646166351 | 19/3691 | 6/641 | 0.0962658892185284 | 1 | F | F | F | F | cysteine-type endopeptidase activity | CATH\_HUMAN | CATLL\_FASHE | D6XHE1\_TRYB2 | G3I1H5\_CRIGR | LGMN\_MOUSE | Q69G21\_TENMO | GO:0042562 | 0.00406393931183961 | 15/3691 | 5/641 | 0.102977983835401 | 1 | F | F | F | F | hormone binding | CATH\_HUMAN | Q9PTT3\_SPAAU | TTHY\_CHICK | TTHY\_MOUSE | TTHY\_RAT | GO:0004857 | 0.00406393931183961 | 15/3691 | 5/641 | 0.102977983835401 | 1 | F | F | F | F | enzyme inhibitor activity | A0A097P6E1\_9FABA | A0A158RFS0\_MUCPR | B2ZGS7\_9ASPA | D9MWI4\_9ASPA | E3VTL0\_9ASPA | GO:0061134 | 0.00189650501219182 | 7/3691 | 3/641 | 0.105674094535567 | 1 | F | F | F | F | peptidase regulator activity | A0A097P6E1\_9FABA | A0A158RFS0\_MUCPR | CATH\_HUMAN | GO:0048038 | 0.00298022216201571 | 11/3691 | 4/641 | 0.107591940270007 | 1 | F | F | F | F | quinone binding | AOC1\_HUMAN | AOCX\_BOVIN | Q5B038\_EMENI | Q96X16\_PICPA | GO:0008081 | 0.00650230289894338 | 24/3691 | 7/641 | 0.10773842419561 | 1 | F | F | F | F | phosphoric diester hydrolase activity | A1HA\_LOXIN | A1HB2\_LOXIN | A311\_LOXLA | ASM3A\_HUMAN | ASM3A\_MOUSE | ENPP2\_HUMAN | ENPP2\_RAT | GO:1901681 | 0.0186941208344622 | 69/3691 | 16/641 | 0.130929800828764 | 1 | F | F | F | F | sulfur compound binding | ADA2\_HUMAN | ANG1\_BOVIN | ANG2\_MOUSE | ANG3\_MOUSE | ANG4\_MOUSE | ANGI\_MOUSE | AOC1\_HUMAN | PA2H1\_AGKCL | PA2H1\_BOTJR | PA2H2\_BOTAS | PPA5\_HUMAN | PPA5\_PIG | PPA5\_RAT | PPAF\_PHAVU | PRS57\_HUMAN | Q5WRG2\_RAT | GO:0008242 | 0.0010837171498239 | 4/3691 | 2/641 | 0.14169327341846 | 1 | F | F | F | F | omega peptidase activity | GGH\_HUMAN | Q6NY42\_DANRE | GO:0004622 | 0.00216743429964779 | 8/3691 | 3/641 | 0.14803616466245 | 1 | F | F | F | F | lysophospholipase activity | ENPP2\_HUMAN | ENPP2\_RAT | PAG15\_HUMAN | GO:0098599 | 0.00216743429964779 | 8/3691 | 3/641 | 0.14803616466245 | 1 | F | F | F | F | palmitoyl hydrolase activity | PPT1\_BOVIN | PPT1\_HUMAN | PPT2\_HUMAN | GO:0016209 | 0.0563532917908426 | 208/3691 | 42/641 | 0.155459669687337 | 1 | F | F | F | F | antioxidant activity | A0A087WNH2\_FICBE | A0A0A0Y4H8\_TRAFO | A0A1S4NYF8\_PANVG | A0A1Y2TH07\_9PEZI | A0A2P1C6N4\_IRPLA | A0A3L6SKP5\_PANMI | APO1\_CYCAE | CAT3\_NEUCR | D1MPT2\_ROYRE | DYP\_AURAJ | GPX3\_HUMAN | GPX5\_HUMAN | GPX6\_MOUSE | K7N5L9\_RAPSA | K7ZUA3\_ASCNO | KATG2\_MAGO7 | L8ICE9\_9CETA | LIG2\_PHACH | LIG4\_PHACH | LIG8\_PHACH | O22443\_SOYBN | PEM1\_PHACH | PER1A\_ARMRU | PER1\_ARAHY | PER1\_SORBI | PER53\_ARATH | PER59\_ARATH | PERL\_BOVIN | PERL\_BUBBU | PERL\_CAPHI | PER\_ARTRA | PER\_COPCI | PGH2\_HUMAN | POXA\_DICDI | PRXC\_LEPFU | Q08J22\_BOMMO | Q40069\_HORVU | Q60FD2\_9APHY | Q8WZK8\_9APHY | SODE\_ONCVO | VPL1\_PLEER | VPL2\_PLEER | GO:0003676 | 0.0495800596044432 | 183/3691 | 37/641 | 0.171784210134598 | 1 | F | F | F | F | nucleic acid binding | AMPS2\_LITPI | ANG1\_BOVIN | ANG2\_MOUSE | ANG3\_MOUSE | ANG4\_MOUSE | ANGI\_MOUSE | B1Q4V2\_HERER | DNAS1\_HUMAN | DNSL3\_HUMAN | E7FH77\_DANRE | ECP\_HUMAN | ENDO2\_ARATH | ENPP2\_HUMAN | ENPP2\_RAT | K7CID1\_PANTR | NUP1\_PENCI | NUS1\_ASPOR | PDE\_NAJAT | Q0KFV0\_SOLLC | Q45U61\_ASPNG | Q5WRG2\_RAT | Q7XZV5\_NICGU | Q9M7C7\_CALSE | Q9SSV1\_NICGU | RNAS1\_BISBI | RNAS4\_HUMAN | RNAS4\_PIG | RNAS6\_HUMAN | RNF1\_GIBFU | RNLE\_SOLLC | RNRH\_RHINI | RNS11\_NICAL | RNS1B\_RAT | RNS3\_PYRPY | RNSL3\_DANRE | RNS\_BOVIN | RNT2\_HUMAN | GO:0008092 | 0.0073150907613113 | 27/3691 | 7/641 | 0.175049638504582 | 1 | F | F | F | F | cytoskeletal protein binding | ANG1\_BOVIN | ANG2\_MOUSE | ANG3\_MOUSE | ANG4\_MOUSE | ANGI\_MOUSE | DNAS1\_HUMAN | Q5WRG2\_RAT | GO:0004536 | 0.00487672717420753 | 18/3691 | 5/641 | 0.190025676463613 | 1 | F | F | F | F | DNA nuclease activity | ATLE\_CYCAE | DNAS1\_HUMAN | DNSL3\_HUMAN | ENDO2\_ARATH | Q0KFV0\_SOLLC | GO:0008238 | 0.0143592522351666 | 53/3691 | 12/641 | 0.197457294919039 | 1 | F | F | F | F | exopeptidase activity | A0NFU8\_ANOGA | CATH\_HUMAN | CBPA1\_PIG | CBPD\_LOPSP | CBPN\_HUMAN | DPP2\_HUMAN | GGH\_HUMAN | LAPA\_ASPOR | O97389\_HELAM | PCP\_HUMAN | PGPSA\_DROME | TPP1\_HUMAN | GO:0004559 | 0.00135464643727987 | 5/3691 | 2/641 | 0.209774458152293 | 1 | F | F | F | F | alpha-mannosidase activity | MAN12\_PENCI | MANA\_CANEN | GO:0016247 | 0.00135464643727987 | 5/3691 | 2/641 | 0.209774458152293 | 1 | F | F | F | F | channel regulator activity | PA2A\_NAJAT | PA2BD\_CRODU | GO:0099106 | 0.00135464643727987 | 5/3691 | 2/641 | 0.209774458152293 | 1 | F | F | F | F | ion channel regulator activity | PA2A\_NAJAT | PA2BD\_CRODU | GO:0004866 | 0.00135464643727987 | 5/3691 | 2/641 | 0.209774458152293 | 1 | F | F | F | F | endopeptidase inhibitor activity | A0A097P6E1\_9FABA | A0A158RFS0\_MUCPR | GO:0008970 | 0.00135464643727987 | 5/3691 | 2/641 | 0.209774458152293 | 1 | F | F | F | F | phospholipase A1 activity | PA1\_VESBA | PAG15\_HUMAN | GO:0004528 | 0.00135464643727987 | 5/3691 | 2/641 | 0.209774458152293 | 1 | F | F | F | F | phosphodiesterase I activity | ENPP2\_HUMAN | ENPP2\_RAT | GO:0061135 | 0.00135464643727987 | 5/3691 | 2/641 | 0.209774458152293 | 1 | F | F | F | F | endopeptidase regulator activity | A0A097P6E1\_9FABA | A0A158RFS0\_MUCPR | GO:0140678 | 0.00514765646166351 | 19/3691 | 5/641 | 0.223511314765564 | 1 | F | F | F | F | molecular function inhibitor activity | A0A097P6E1\_9FABA | A0A158RFS0\_MUCPR | B2ZGS7\_9ASPA | D9MWI4\_9ASPA | E3VTL0\_9ASPA | GO:0016899 | 0.00406393931183961 | 15/3691 | 4/641 | 0.255439431547781 | 1 | F | F | F | F | oxidoreductase activity, acting on the CH-OH group of donors, oxygen as acceptor | GAOA\_GIBZA | GOX\_ASPNG | MNCO\_MICNN | O94219\_PLEER | GO:0048018 | 0.00541858574911948 | 20/3691 | 5/641 | 0.258580043156787 | 1 | F | F | F | F | receptor ligand activity | ADA2\_HUMAN | Q9PTT3\_SPAAU | TTHY\_CHICK | TTHY\_MOUSE | TTHY\_RAT | GO:0030546 | 0.00541858574911948 | 20/3691 | 5/641 | 0.258580043156787 | 1 | F | F | F | F | signaling receptor activator activity | ADA2\_HUMAN | Q9PTT3\_SPAAU | TTHY\_CHICK | TTHY\_MOUSE | TTHY\_RAT | GO:0004667 | 0.00162557572473584 | 6/3691 | 2/641 | 0.28012286085327 | 1 | F | F | F | F | prostaglandin-D synthase activity | PTGDS\_HUMAN | PTGDS\_MOUSE | GO:0030414 | 0.00162557572473584 | 6/3691 | 2/641 | 0.28012286085327 | 1 | F | F | F | F | peptidase inhibitor activity | A0A097P6E1\_9FABA | A0A158RFS0\_MUCPR | GO:0140905 | 0.00162557572473584 | 6/3691 | 2/641 | 0.28012286085327 | 1 | F | F | F | F | haloperoxidase activity | POXA\_DICDI | PRXC\_LEPFU | GO:0106411 | 0.00162557572473584 | 6/3691 | 2/641 | 0.28012286085327 | 1 | F | F | F | F | XMP 5'-nucleosidase activity | PPAP\_RAT | V5NTD\_NAJAT | GO:0140906 | 0.00162557572473584 | 6/3691 | 2/641 | 0.28012286085327 | 1 | F | F | F | F | halogenase activity | POXA\_DICDI | PRXC\_LEPFU | GO:0030545 | 0.00568951503657545 | 21/3691 | 5/641 | 0.294821391109647 | 1 | F | F | F | F | signaling receptor regulator activity | ADA2\_HUMAN | Q9PTT3\_SPAAU | TTHY\_CHICK | TTHY\_MOUSE | TTHY\_RAT | GO:0019213 | 0.00298022216201571 | 11/3691 | 3/641 | 0.295664052123882 | 1 | F | F | F | F | deacetylase activity | A2QZC8\_ASPNC | CDA\_COLLN | CDA\_EMENI | GO:0016638 | 0.00894066648604714 | 33/3691 | 7/641 | 0.345417304281489 | 1 | F | F | F | F | oxidoreductase activity, acting on the CH-NH2 group of donors | AOC1\_HUMAN | AOCX\_BOVIN | OXLA\_BOTAT | OXLA\_CALRH | OXLA\_GLOHA | Q5B038\_EMENI | Q96X16\_PICPA | GO:0047372 | 0.00189650501219182 | 7/3691 | 2/641 | 0.349902367380414 | 1 | F | F | F | F | acylglycerol lipase activity | LIPR2\_HUMAN | LIPR2\_RAT | GO:0008239 | 0.00189650501219182 | 7/3691 | 2/641 | 0.349902367380414 | 1 | F | F | F | F | dipeptidyl-peptidase activity | DPP2\_HUMAN | PCP\_HUMAN | GO:0003953 | 0.00189650501219182 | 7/3691 | 2/641 | 0.349902367380414 | 1 | F | F | F | F | NAD+ nucleosidase activity | NADA\_APLCA | NADA\_ASPFU | GO:0008474 | 0.00189650501219182 | 7/3691 | 2/641 | 0.349902367380414 | 1 | F | F | F | F | palmitoyl-(protein) hydrolase activity | PPT1\_BOVIN | PPT1\_HUMAN | GO:0008235 | 0.00758602004876727 | 28/3691 | 6/641 | 0.356768088874459 | 1 | F | F | F | F | metalloexopeptidase activity | A0NFU8\_ANOGA | CBPA1\_PIG | CBPD\_LOPSP | CBPN\_HUMAN | LAPA\_ASPOR | O97389\_HELAM | GO:0033218 | 0.0124627472229748 | 46/3691 | 9/641 | 0.405101167574308 | 1 | F | F | F | F | amide binding | ANG2\_MOUSE | ANG3\_MOUSE | ANG4\_MOUSE | ANGI\_MOUSE | CATD\_RAT | CYP5\_CAEEL | PGPSA\_DROME | Q5WRG2\_RAT | TPP1\_HUMAN | GO:0016755 | 0.00677323218639935 | 25/3691 | 5/641 | 0.443756244116687 | 1 | F | F | F | F | aminoacyltransferase activity | O81226\_CARPA | QPCT1\_DROME | QPCT2\_DROME | QPCT\_IXOSC | QPCT\_MOUSE | GO:0005501 | 0.00243836358710377 | 9/3691 | 2/641 | 0.480750127886325 | 1 | F | F | F | F | retinoid binding | PTGDS\_HUMAN | PTGDS\_MOUSE | GO:0038023 | 0.00243836358710377 | 9/3691 | 2/641 | 0.480750127886325 | 1 | F | F | F | F | signaling receptor activity | CATH\_HUMAN | PGPSA\_DROME | GO:0060089 | 0.00243836358710377 | 9/3691 | 2/641 | 0.480750127886325 | 1 | F | F | F | F | molecular transducer activity | CATH\_HUMAN | PGPSA\_DROME | GO:0032052 | 0.00243836358710377 | 9/3691 | 2/641 | 0.480750127886325 | 1 | F | F | F | F | bile acid binding | PA21B\_BOVIN | PA21B\_PIG | GO:0016290 | 0.00243836358710377 | 9/3691 | 2/641 | 0.480750127886325 | 1 | F | F | F | F | palmitoyl-CoA hydrolase activity | PPT1\_BOVIN | PPT1\_HUMAN | GO:0008289 | 0.0216743429964779 | 80/3691 | 14/641 | 0.53432969391536 | 1 | F | F | F | F | lipid binding | ECP\_HUMAN | LIPG\_HUMAN | PA21B\_BOVIN | PA21B\_PIG | PA2GA\_HUMAN | PA2GE\_HUMAN | PA2GX\_HUMAN | PAG15\_HUMAN | PLA22\_ORYSJ | PPT1\_BOVIN | PPT1\_HUMAN | PTGDS\_HUMAN | PTGDS\_MOUSE | TPP1\_HUMAN | GO:0047617 | 0.00270929287455974 | 10/3691 | 2/641 | 0.539844745362411 | 1 | F | F | F | F | acyl-CoA hydrolase activity | PPT1\_BOVIN | PPT1\_HUMAN | GO:0140097 | 0.00785694933622325 | 29/3691 | 5/641 | 0.583905084133439 | 1 | F | F | F | F | catalytic activity, acting on DNA | ATLE\_CYCAE | DNAS1\_HUMAN | DNSL3\_HUMAN | ENDO2\_ARATH | Q0KFV0\_SOLLC | GO:0008253 | 0.00298022216201571 | 11/3691 | 2/641 | 0.594107121638481 | 1 | F | F | F | F | 5'-nucleotidase activity | PPAP\_RAT | V5NTD\_NAJAT | GO:0046914 | 0.116770522893525 | 431/3691 | 73/641 | 0.620836448295018 | 1 | F | F | F | F | transition metal ion binding | A0A0A7M685\_TRAHI | A0A0M3U1T9\_9APHY | A0A0R4I979\_BRABE | A0A2H4A2Q2\_9APHY | A0A3F2YLU5\_9APHY | A0NFU8\_ANOGA | A2QS62\_ASPNC | ADA2\_HUMAN | ANG1\_BOVIN | ANG2\_MOUSE | ANG3\_MOUSE | ANG4\_MOUSE | ANGI\_MOUSE | AOC1\_HUMAN | AOCX\_BOVIN | ASM3A\_HUMAN | ASM3A\_MOUSE | B2L9C1\_TRAHI | B4F320\_LIMPO | BLRO\_ALBVE | CAH1\_CHLRE | CAH6\_HUMAN | CBPA1\_PIG | CBPD\_LOPSP | CBPN\_HUMAN | CDA\_COLLN | CERU\_RAT | D7F485\_9APHY | DOPO\_HUMAN | ENPP2\_HUMAN | ENPP2\_RAT | F6N9E7\_9PEZI | G2QG31\_MYCTT | H8ZRU2\_9HELO | HE12\_DANRE | I1SB14\_9APHY | I1VE66\_9APHY | LAC1\_MELAO | LAC1\_TRAMX | LAC2\_TRAVE | M1GME7\_9APHY | MMP1\_PIG | O97389\_HELAM | PAG15\_HUMAN | PGPSA\_DROME | PGRP1\_CAMDR | PPA5\_HUMAN | PPA5\_PIG | PPA5\_RAT | PPAF\_PHAVU | Q08J22\_BOMMO | Q12571\_9BASI | Q1W6B1\_9APHY | Q2TWF5\_ASPOR | Q5B038\_EMENI | Q5EBY5\_9APHY | Q5WRG2\_RAT | Q6H9H7\_9APHY | Q86RS6\_MANSE | Q8TG94\_TRAPU | Q96TR6\_PYCCO | Q96UT7\_TRAVE | Q96X16\_PICPA | Q9HDQ0\_9APHY | Q9UVQ2\_PYCCI | Q9Y780\_COPCI | QPCT1\_DROME | QPCT2\_DROME | QPCT\_IXOSC | QPCT\_MOUSE | SODE\_ONCVO | TRFL\_BUBBU | VM1A3\_DEIAC | GO:0019840 | 0.00352208073692766 | 13/3691 | 2/641 | 0.687893680746689 | 1 | F | F | F | F | isoprenoid binding | PTGDS\_HUMAN | PTGDS\_MOUSE | GO:0016289 | 0.00352208073692766 | 13/3691 | 2/641 | 0.687893680746689 | 1 | F | F | F | F | CoA hydrolase activity | PPT1\_BOVIN | PPT1\_HUMAN | GO:0016758 | 0.0157138986724465 | 58/3691 | 9/641 | 0.699276534897854 | 1 | F | F | F | F | hexosyltransferase activity | GBA1\_HUMAN | HYAL1\_HUMAN | LALBA\_BOVIN | LALBA\_CAPHI | LALBA\_CAVPO | LALBA\_PAPCY | OFUT1\_CAEEL | Q07524\_TROMA | XTH34\_POPPZ | GO:0008199 | 0.0073150907613113 | 27/3691 | 4/641 | 0.714976137339146 | 1 | F | F | F | F | ferric iron binding | PPA5\_HUMAN | PPA5\_PIG | PPA5\_RAT | PPAF\_PHAVU | GO:0098772 | 0.0230289894337578 | 85/3691 | 13/641 | 0.738029844654235 | 1 | F | F | F | F | molecular function regulator activity | A0A097P6E1\_9FABA | A0A158RFS0\_MUCPR | ADA2\_HUMAN | B2ZGS7\_9ASPA | CATH\_HUMAN | D9MWI4\_9ASPA | E3VTL0\_9ASPA | PA2A\_NAJAT | PA2BD\_CRODU | Q9PTT3\_SPAAU | TTHY\_CHICK | TTHY\_MOUSE | TTHY\_RAT | GO:0003677 | 0.0111081007856949 | 41/3691 | 6/641 | 0.740783561173049 | 1 | F | F | F | F | DNA binding | ANG1\_BOVIN | ANG2\_MOUSE | ANG3\_MOUSE | ANGI\_MOUSE | DNAS1\_HUMAN | DNSL3\_HUMAN | GO:0016811 | 0.0130046057978868 | 48/3691 | 7/641 | 0.752506247699232 | 1 | F | F | F | F | hydrolase activity, acting on carbon-nitrogen (but not peptide) bonds, in linear amides | A0A0R4I979\_BRABE | A2QZC8\_ASPNC | ASAH1\_BALAS | CDA\_COLLN | CDA\_EMENI | PGRP1\_CAMDR | Q86RS6\_MANSE | GO:0140677 | 0.0113790300731509 | 42/3691 | 6/641 | 0.762264135354217 | 1 | F | F | F | F | molecular function activator activity | ADA2\_HUMAN | CATH\_HUMAN | Q9PTT3\_SPAAU | TTHY\_CHICK | TTHY\_MOUSE | TTHY\_RAT | GO:0008252 | 0.00406393931183961 | 15/3691 | 2/641 | 0.763104961590509 | 1 | F | F | F | F | nucleotidase activity | PPAP\_RAT | V5NTD\_NAJAT | GO:0008198 | 0.00650230289894338 | 24/3691 | 3/641 | 0.81356162257179 | 1 | F | F | F | F | ferrous iron binding | PPA5\_HUMAN | PPA5\_PIG | PPA5\_RAT | GO:0030234 | 0.0121918179355188 | 45/3691 | 6/641 | 0.81876620404083 | 1 | F | F | F | F | enzyme regulator activity | A0A097P6E1\_9FABA | A0A158RFS0\_MUCPR | B2ZGS7\_9ASPA | CATH\_HUMAN | D9MWI4\_9ASPA | E3VTL0\_9ASPA | GO:0042578 | 0.0387428881062043 | 143/3691 | 21/641 | 0.835312271583439 | 1 | F | F | F | F | phosphoric ester hydrolase activity | A1HA\_LOXIN | A1HB2\_LOXIN | A2TBB4\_9ASCO | A311\_LOXLA | A4GX63\_TOXGO | ASM3A\_HUMAN | ASM3A\_MOUSE | ENPP2\_HUMAN | ENPP2\_RAT | F6MIW5\_WHEAT | PHYA\_ASPFU | PHYA\_ASPNG | PHYB\_ASPAW | PPA5\_HUMAN | PPA5\_PIG | PPA5\_RAT | PPAF1\_IPOBA | PPAF\_PHAVU | PPAP\_RAT | Q8VX11\_LUPLU | V5NTD\_NAJAT | GO:0016790 | 0.00677323218639935 | 25/3691 | 3/641 | 0.835331227167959 | 1 | F | F | F | F | thiolester hydrolase activity | PPT1\_BOVIN | PPT1\_HUMAN | PPT2\_HUMAN | GO:0008270 | 0.052560281766459 | 194/3691 | 29/641 | 0.844165257581068 | 1 | F | F | F | F | zinc ion binding | A0A0R4I979\_BRABE | A0NFU8\_ANOGA | ADA2\_HUMAN | AOC1\_HUMAN | ASM3A\_HUMAN | ASM3A\_MOUSE | B4F320\_LIMPO | CAH1\_CHLRE | CAH6\_HUMAN | CBPA1\_PIG | CBPD\_LOPSP | CBPN\_HUMAN | CDA\_COLLN | ENPP2\_HUMAN | ENPP2\_RAT | HE12\_DANRE | MMP1\_PIG | O97389\_HELAM | PAG15\_HUMAN | PGPSA\_DROME | PGRP1\_CAMDR | PPAF\_PHAVU | Q2TWF5\_ASPOR | Q86RS6\_MANSE | QPCT1\_DROME | QPCT2\_DROME | QPCT\_IXOSC | QPCT\_MOUSE | VM1A3\_DEIAC | GO:0004527 | 0.00487672717420753 | 18/3691 | 2/641 | 0.846317467594966 | 1 | F | F | F | F | exonuclease activity | ENPP2\_HUMAN | ENPP2\_RAT | GO:0004089 | 0.00704416147385532 | 26/3691 | 3/641 | 0.854875797341026 | 1 | F | F | F | F | carbonate dehydratase activity | CAH1\_CHLRE | CAH6\_HUMAN | Q2TWF5\_ASPOR | GO:0071949 | 0.0149011108100786 | 55/3691 | 7/641 | 0.86550563800295 | 1 | F | F | F | F | FAD binding | A0A098DND1\_GIBZE | CKX1\_MAIZE | CYND4\_CYNDA | GOOX\_SARSR | MNCO\_MICNN | THCAS\_CANSA | XYLO\_MYCTT | GO:0004096 | 0.00541858574911948 | 20/3691 | 2/641 | 0.886009194522145 | 1 | F | F | F | F | catalase activity | CAT3\_NEUCR | KATG2\_MAGO7 | GO:0050660 | 0.031698726632349 | 117/3691 | 16/641 | 0.886565450696542 | 1 | F | F | F | F | flavin adenine dinucleotide binding | A0A060SC37\_PYCCI | A0A098DND1\_GIBZE | A0A1S9DW10\_ASPOZ | B8MX95\_ASPFN | CKX1\_MAIZE | CYND4\_CYNDA | G0SAW6\_CHATD | G2PZJ2\_MYCTT | GOOX\_SARSR | GOX\_ASPNG | MDL1\_PRUDU | MNCO\_MICNN | O94219\_PLEER | PDH1\_LEUMG | THCAS\_CANSA | XYLO\_MYCTT | GO:0046527 | 0.00758602004876727 | 28/3691 | 3/641 | 0.887961074192607 | 1 | F | F | F | F | glucosyltransferase activity | GBA1\_HUMAN | Q07524\_TROMA | XTH34\_POPPZ | GO:0003755 | 0.00758602004876727 | 28/3691 | 3/641 | 0.887961074192607 | 1 | F | F | F | F | peptidyl-prolyl cis-trans isomerase activity | A0A0R3QSA7\_9BILA | A0A3S5H5N2\_LEIDO | CYP5\_CAEEL | GO:0005504 | 0.00568951503657545 | 21/3691 | 2/641 | 0.902082188098677 | 1 | F | F | F | F | fatty acid binding | PTGDS\_HUMAN | PTGDS\_MOUSE | GO:0008194 | 0.0121918179355188 | 45/3691 | 5/641 | 0.912178798385261 | 1 | F | F | F | F | UDP-glycosyltransferase activity | HYAL1\_HUMAN | LALBA\_BOVIN | LALBA\_CAPHI | LALBA\_CAVPO | LALBA\_PAPCY | GO:0004177 | 0.00812787862367922 | 30/3691 | 3/641 | 0.914136679732304 | 1 | F | F | F | F | aminopeptidase activity | CATH\_HUMAN | DPP2\_HUMAN | LAPA\_ASPOR | GO:0003824 | 0.986453535627201 | 3641/3691 | 629/641 | 0.919593971061657 | 1 | F | F | F | F | catalytic activity | A0A059U759\_9PEZI | A0A060N399\_9PLEO | A0A060SC37\_PYCCI | A0A068FT77\_9PEZI | A0A075B5G4\_HUMIN | A0A075B5H6\_TRIHA | A0A075C6T6\_RHIMI | A0A086SY89\_ACRC1 | A0A086T6R4\_ACRC1 | A0A087WNH2\_FICBE | A0A088T0J9\_GEOCN | A0A097P6E1\_9FABA | A0A098DND1\_GIBZE | A0A0A0Y4H8\_TRAFO | A0A0A7M685\_TRAHI | A0A0J5Q413\_ASPFM | A0A0M3KKZ6\_RHIMI | A0A0M3KKZ8\_RHIMI | A0A0M3U1T9\_9APHY | A0A0R3QSA7\_9BILA | A0A0R4I979\_BRABE | A0A0S2GKZ1\_9APHY | A0A173N065\_EISFE | A0A183C5H8\_GLOPA | A0A1D3S5H0\_FUSOX | A0A1L6CE30\_9EURO | A0A1L8D5Z7\_BOTAT | A0A1L9WG58\_ASPA1 | A0A1S4NYF8\_PANVG | A0A1S6YJF3\_MALCI | A0A1S9DRB1\_ASPOZ | A0A1S9DW10\_ASPOZ | A0A1Y2TH07\_9PEZI | A0A2H4A2Q2\_9APHY | A0A2H5BN17\_TALPI | A0A2N1LTK3\_TRIHA | A0A2P1C6N4\_IRPLA | A0A2U8ZTY7\_RHIZD | A0A2Z4HIN9\_9EURO | A0A384E148\_NICBE | A0A3B6UEQ2\_RHIMI | A0A3B6UEQ6\_EISFE | A0A3F2YLU5\_9APHY | A0A3G2C3I4\_9EURO | A0A3G4RHU4\_9PEZI | A0A3L6SKP5\_PANMI | A0A3S5H5N2\_LEIDO | A0A482LWB1\_OSTFU | A0A5J6BJN2\_MALCI | A0A6F8Z6Y2\_BOMMO | A0A6M9BP13\_9EURO | A0A6P6YAT6\_DERPT | A0A7S6G7I6\_9PEZI | A0A856TAI5\_9BASI | A0NFU8\_ANOGA | A1E266\_9PEZI | A1HA\_LOXIN | A1HB2\_LOXIN | A2QIR3\_ASPNC | A2QS62\_ASPNC | A2QZC8\_ASPNC | A2TBB4\_9ASCO | A2TM14\_HEVBR | A311\_LOXLA | A4GX63\_TOXGO | A5AB48\_ASPNC | A6PZ97\_SALSA | A6YRT4\_9PEZI | A7KMF0\_9CAEN | A8NI40\_COPC7 | A9LI60\_BIOOC | A9ZSX9\_9BRYO | ABFB\_ASPKW | ADA2\_HUMAN | ADPG2\_ARATH | AGAL\_HUMAN | AGAL\_ORYSJ | AMPS2\_LITPI | AMY1A\_HUMAN | AMY1\_HORVU | AMY1\_ORYSJ | AMYA1\_ASPOR | AMYG\_SACFI | AMY\_ORYLA | ANAG\_HUMAN | ANG1\_BOVIN | ANG2\_MOUSE | ANG3\_MOUSE | ANG4\_MOUSE | ANGI\_MOUSE | AOAH\_MOUSE | AOC1\_HUMAN | AOCX\_BOVIN | APO1\_CYCAE | ASAH1\_BALAS | ASM3A\_HUMAN | ASM3A\_MOUSE | ATLE\_CYCAE | AXE1\_ASPAW | AXE2\_TALPU | AXHA2\_EMENI | B1Q4V2\_HERER | B2L9C1\_TRAHI | B4F320\_LIMPO | B7X9Z0\_COPCI | B7X9Z2\_COPCI | B8MX95\_ASPFN | B9TU22\_GADMO | BGALA\_ASPNC | BGALA\_ASPOR | BGALA\_PENSQ | BGL1\_ASPAC | BGLA\_ASPFU | BGLA\_ASPOR | BGLR\_HUMAN | BLRO\_ALBVE | C3VEV9\_PENCN | C7YSL3\_FUSV7 | CAH1\_CHLRE | CAH6\_HUMAN | CARP1\_CANAL | CARP2\_CANAX | CARP\_RHIPU | CAT3\_NEUCR | CATD\_RAT | CATH\_HUMAN | CATLL\_FASHE | CBHB\_ASPFU | CBHRE\_GEOS1 | CBPA1\_PIG | CBPD\_LOPSP | CBPN\_HUMAN | CDA\_COLLN | CDA\_EMENI | CEL2A\_PIG | CERU\_RAT | CFAD\_MOUSE | CHI1\_COCPS | CHI2\_HORVU | CHI2\_ORYSJ | CHI33\_TRIHA | CHI42\_TRIHA | CHI4\_CRYJA | CHIA\_HUMAN | CHIC\_ARATH | CHIC\_SECCE | CHIL3\_MOUSE | CHIT\_PUNGR | CHLY\_HEVBR | CHYM\_CAMDR | CKX1\_MAIZE | COGS\_HYPLI | CONB\_CANEN | CUCM1\_CUCME | CUTI1\_ASPOR | CUTI1\_COLGL | CUTI1\_FUSVN | CUTI1\_HYPJR | CYND4\_CYNDA | CYP5\_CAEEL | CYSP\_BLOTA | D0QF43\_9HELO | D1M8S7\_HEVBR | D1MPT2\_ROYRE | D6XHE1\_TRYB2 | D7F485\_9APHY | D9MWI4\_9ASPA | DABA\_PSEMU | DDN1\_BOVIN | DEXT\_TALMI | DIR\_GLYEC | DNAS1\_HUMAN | DNSL3\_HUMAN | DOPO\_HUMAN | DPP2\_HUMAN | DYP\_AURAJ | E0A7J0\_YARLL | E0CX04\_MOMBA | E0XN39\_9EURO | E13B\_HORVU | E13C\_MUSAC | E3VTL0\_9ASPA | E5D0X5\_SCHOC | E7FH77\_DANRE | E9G5J5\_DAPPU | ECP\_HUMAN | EGFB2\_MOUSE | EGLB\_ASPNG | ENDO2\_ARATH | ENG1\_RHIMI | ENPP2\_HUMAN | ENPP2\_RAT | ERVB\_TABDI | EST6\_DROME | EXG1\_CANAL | EXG1\_YEAST | F0ZJZ1\_DICPU | F1CYZ0\_TALFU | F2Z7L1\_9ANNE | F6MIW5\_WHEAT | F6N9E7\_9PEZI | FAEA\_ASPNG | FAEB1\_ASPOR | FAEB2\_ASPOR | FUCO\_HUMAN | G0RVK1\_HYPJQ | G0SAW6\_CHATD | G2PZJ2\_MYCTT | G2Q665\_MYCTT | G2QG31\_MYCTT | G2QVH2\_THETT | G2X3Y1\_VERDV | G3I1H5\_CRIGR | G3JPF7\_CORMM | G3YAL0\_ASPNA | G3YFQ1\_ASPNA | G8GLP2\_LENED | G9NTY1\_HYPAI | GALNS\_HUMAN | GANA\_ASPAC | GANA\_EMENI | GANA\_HUMIN | GAOA\_GIBZA | GBA1\_HUMAN | GCE2\_MYCTT | GCE\_CERUI | GCE\_HYPJQ | GGH\_HUMAN | GH7B\_LIMQU | GILT\_MOUSE | GOOX\_SARSR | GOX\_ASPNG | GPX3\_HUMAN | GPX5\_HUMAN | GPX6\_MOUSE | GRAA\_HUMAN | GRAC\_MOUSE | GRAK\_HUMAN | GRASS\_DROME | GUB2\_HORVU | GUN2\_HYPJE | GUN6\_HUMIN | GUN7\_HYPJQ | GUNC\_FUSOX | GUN\_ASPAC | GUN\_CRYAT | GUN\_MYTED | GUX1\_HUMGT | GUX1\_HYPJE | GUX1\_TRIHA | GUX2\_HYPJE | H1AE14\_PHACH | H8ZRU2\_9HELO | HE12\_DANRE | HEXC\_OSTFU | HS3S1\_MOUSE | HYAL1\_HUMAN | I1SB14\_9APHY | I1SB18\_VIPAE | I1VE66\_9APHY | I2FI81\_EISFE | I3RY46\_TRIHA | IDH\_OSTTA | IDUA\_HUMAN | INU2\_ASPFI | INUE\_ASPAW | INV\_SCHOC | IPUA\_ASPNG | J7LCB0\_DEIAC | J9UN47\_GIBZA | K7CID1\_PANTR | K7N5L9\_RAPSA | K7ZUA3\_ASCNO | K9L8F3\_MALCI | KATG2\_MAGO7 | KLK10\_HUMAN | KLK1\_HUMAN | KLK2\_HORSE | KLK2\_HUMAN | KLK7\_HUMAN | KLK7\_MOUSE | KLK8\_MOUSE | L7SVX1\_RHIMI | L8ICE9\_9CETA | LAC1\_MELAO | LAC1\_TRAMX | LAC2\_TRAVE | LALBA\_BOVIN | LALBA\_CAPHI | LALBA\_CAVPO | LALBA\_PAPCY | LAPA\_ASPOR | LGMN\_MOUSE | LICH\_HUMAN | LIG2\_PHACH | LIG4\_PHACH | LIG8\_PHACH | LIP1\_DIURU | LIP2\_DIURU | LIP2\_GEOCN | LIP3\_DIURU | LIPA\_MOEAP | LIPB\_PSEA2 | LIPG\_CANLF | LIPG\_HUMAN | LIPP\_HORSE | LIPR1\_CANLF | LIPR1\_HUMAN | LIPR2\_HUMAN | LIPR2\_RAT | LIP\_THELA | LUCI\_OPLGR | LYG\_STRCA | LYS1\_MUSDO | LYSC1\_ANAPL | LYSC1\_CANLF | LYSC1\_HORSE | LYSC2\_BOVIN | LYSC2\_ONCMY | LYSC\_COTJA | LYSC\_EQUAS | LYSC\_NUMME | LYSC\_OPIHO | LYSC\_PELSI | LYS\_BOMMO | LYS\_RUDPH | M1GME7\_9APHY | M2RAI8\_CERS8 | M9TI89\_RHIPU | MAN12\_PENCI | MAN4\_SOLLC | MANA\_ASPNC | MANA\_CANEN | MANA\_CRYAT | MANA\_HYPJR | MANA\_MYTED | MANA\_PODAN | MANBA\_MOUSE | MCPT2\_RAT | MDL1\_PRUDU | MDLA\_PENCA | MDLA\_PENCY | MEL1\_YEASX | MMP1\_PIG | MNCO\_MICNN | MNLOX\_MAGO7 | NADA\_APLCA | NADA\_ASPFU | NAGAB\_CHICK | NAGAB\_HUMAN | NANL\_MACDE | NCS\_THLFG | NP4\_RHOPR | NTP1\_TOXGO | NTP2\_TOXGO | NUP1\_PENCI | NUS1\_ASPOR | O00095\_HYPJE | O04358\_IRIHO | O22443\_SOYBN | O44049\_TRYRA | O74705\_ASPNG | O77044\_9NEOP | O81100\_SOLLC | O81226\_CARPA | O81934\_CANEN | O94219\_PLEER | O97389\_HELAM | OFUT1\_CAEEL | OXLA\_BOTAT | OXLA\_CALRH | OXLA\_GLOHA | P79074\_9AGAR | PA1\_VESBA | PA21B\_BOVIN | PA21B\_PIG | PA2A1\_BUNCE | PA2A1\_ECHCA | PA2A1\_NAJAT | PA2A1\_OPHHA | PA2A2\_NAJNA | PA2A2\_OPHHA | PA2A2\_TROCA | PA2A4\_NAJSG | PA2A5\_TRIST | PA2A7\_GLOHA | PA2A\_BOTJR | PA2A\_CROAT | PA2A\_DEIAC | PA2A\_GLOHA | PA2A\_NAJAT | PA2B1\_AGKPI | PA2B2\_BOTJR | PA2B2\_PROFL | PA2B3\_BOTAS | PA2B3\_BUNCE | PA2B5\_BUNCE | PA2B5\_NOTSC | PA2BA\_VIPAA | PA2BB\_GLOHA | PA2BB\_PSEAU | PA2BC\_VIPAA | PA2BD\_CRODU | PA2B\_BUNCE | PA2B\_NOTSC | PA2GA\_HUMAN | PA2GE\_HUMAN | PA2GX\_HUMAN | PA2H1\_AGKCL | PA2H1\_BOTBZ | PA2H1\_BOTJR | PA2H1\_BOTMO | PA2H1\_BOTPI | PA2H2\_BOTAS | PA2H2\_BOTMO | PA2H2\_BOTPI | PA2H2\_CERGO | PA2H3\_BOTPI | PA2HB\_AGKPI | PA2HB\_OXYSC | PA2HH\_TRIST | PA2HS\_ECHCA | PA2H\_BOTPA | PA2H\_DEIAC | PA2H\_PROMB | PA2N\_GLOHA | PA2\_APIME | PAG15\_HUMAN | PCP\_HUMAN | PDE\_NAJAT | PDH1\_LEUMG | PELA\_ASPNG | PELB\_ASPNG | PEM1\_PHACH | PEPA\_ASPPH | PER1A\_ARMRU | PER1\_ARAHY | PER1\_SORBI | PER53\_ARATH | PER59\_ARATH | PERL\_BOVIN | PERL\_BUBBU | PERL\_CAPHI | PER\_ARTRA | PER\_COPCI | PGH2\_HUMAN | PGLR1\_ASPAC | PGLR1\_ASPNG | PGLR\_GIBFU | PGPSA\_DROME | PGRP1\_CAMDR | PHAZ\_TALFU | PHNH\_PENHR | PHYA\_ASPFU | PHYA\_ASPNG | PHYB\_ASPAW | PLA22\_ORYSJ | PLY1\_JUNAS | PME\_DAUCA | PME\_SITOR | POXA\_DICDI | PPA5\_HUMAN | PPA5\_PIG | PPA5\_RAT | PPAF1\_HOLDI | PPAF1\_IPOBA | PPAF\_PHAVU | PPAP\_RAT | PPO8\_ANOGA | PPT1\_BOVIN | PPT1\_HUMAN | PPT2\_HUMAN | PRS57\_HUMAN | PRTN3\_HUMAN | PRXC\_LEPFU | PTGDS\_HUMAN | PTGDS\_MOUSE | Q02321\_PHACH | Q06AK3\_TOXGO | Q07524\_TROMA | Q08J22\_BOMMO | Q0KFV0\_SOLLC | Q12571\_9BASI | Q12715\_HYPJE | Q1W6B1\_9APHY | Q2QEH4\_SAPOF | Q2TWF5\_ASPOR | Q2U8V9\_ASPOR | Q2Z1W1\_PHACH | Q40069\_HORVU | Q43576\_TOBAC | Q45U61\_ASPNG | Q4AE59\_OSTFU | Q4WP32\_ASPFU | Q50KB2\_PHACH | Q55FE6\_DICDI | Q588B8\_CRYJA | Q5B038\_EMENI | Q5EBY5\_9APHY | Q5WRG2\_RAT | Q60FD2\_9APHY | Q69G21\_TENMO | Q6ED33\_ASPNG | Q6H9H7\_9APHY | Q6NY42\_DANRE | Q6R7Z5\_9TRYP | Q6S5M9\_9ASCO | Q6VAY1\_9PEZI | Q6WER3\_GIBZA | Q6WSR8\_PICAB | Q70C53\_SOLTU | Q70SY0\_HYPJE | Q7LHI2\_PHACH | Q7LIJ0\_PHACH | Q7LST4\_PENEN | Q7RWP2\_NEUCR | Q7X9A9\_CAMSI | Q7XZV5\_NICGU | Q7YXL2\_TENMO | Q86RS6\_MANSE | Q874E9\_9TREE | Q8H0C9\_VIGUN | Q8J0K6\_MELAO | Q8J0K8\_MELAO | Q8NJY6\_9HYPO | Q8T0W7\_9NEOP | Q8TFL9\_TALEM | Q8TG26\_THEAU | Q8TG94\_TRAPU | Q8TGI8\_TALEM | Q8VX11\_LUPLU | Q8WZK8\_9APHY | Q92456\_HYPJE | Q92458\_HYPJE | Q93X60\_CICIN | Q94BW3\_CINCA | Q95KP4\_HORSE | Q95V66\_PENVA | Q96TR6\_PYCCO | Q96UT7\_TRAVE | Q96X16\_PICPA | Q9FUH3\_VIGUS | Q9GPG0\_MANSE | Q9HDQ0\_9APHY | Q9LYJ5\_ARATH | Q9M7C7\_CALSE | Q9P8F7\_YARLL | Q9SSV1\_NICGU | Q9STC1\_GRALE | Q9UVQ2\_PYCCI | Q9XEI3\_HORVV | Q9Y780\_COPCI | QPCT1\_DROME | QPCT2\_DROME | QPCT\_IXOSC | QPCT\_MOUSE | RENI\_RAT | RGLA\_ASPAC | RHA1\_ASPAC | RHGA\_ASPAC | RIP0\_DIACA | RIP1\_BRYDI | RIP1\_HORVU | RIP1\_MOMCH | RIP1\_PHYAM | RIP2\_PHYAM | RIP3\_MOMCH | RIPA\_PHYAM | RIPG\_SURMU | RIPL1\_PHYDI | RIPL2\_PHYDI | RIPT\_TRIKI | RNAS1\_BISBI | RNAS4\_HUMAN | RNAS4\_PIG | RNAS6\_HUMAN | RNF1\_GIBFU | RNLE\_SOLLC | RNRH\_RHINI | RNS11\_NICAL | RNS1B\_RAT | RNS3\_PYRPY | RNSL3\_DANRE | RNS\_BOVIN | RNT2\_HUMAN | S6BC01\_PSEA2 | S7Q6I2\_GLOTA | S7ZIW0\_PENO1 | SIA\_ASPFU | SODE\_ONCVO | THCAS\_CANSA | TLP\_PRUAV | TPP1\_HUMAN | TRFL\_BUBBU | TRFL\_HORSE | TRY1\_GADMO | TRY3\_SALSA | TRYB2\_HUMAN | V5NTD\_NAJAT | VM11\_BOTMO | VM12\_CROAD | VM1A3\_DEIAC | VM1BI\_BOTMO | VM1T1\_PROMU | VM1T2\_PROFL | VPL1\_PLEER | VPL2\_PLEER | VSPP\_DEIAC | VSPSX\_GLOSA | W0T408\_KLUMD | W4KMP1\_HETIT | W8P1L2\_TALEM | W8VR85\_TALPI | X0BTD8\_FUSOX | X0M5X0\_FUSOX | XGHA\_ASPTU | XTH34\_POPPZ | XYLA\_ASPNC | XYLO\_MYCTT | XYN1\_HYPJR | XYN2\_HYPJR | XYN3\_ASPKW | XYN3\_HYPJQ | XYNA\_FUSO4 | XYNA\_PENSI | XYNA\_THEAU | XYNA\_THELA | XYNC\_ASPNC | XYND\_EMENI | GO:0019900 | 0.0062313736114874 | 23/3691 | 2/641 | 0.928079168286155 | 1 | F | F | F | F | kinase binding | CHIA\_HUMAN | CHIL3\_MOUSE | GO:0016859 | 0.00866973719859117 | 32/3691 | 3/641 | 0.934629445229816 | 1 | F | F | F | F | cis-trans isomerase activity | A0A0R3QSA7\_9BILA | A0A3S5H5N2\_LEIDO | CYP5\_CAEEL | GO:0033293 | 0.010837171498239 | 40/3691 | 4/641 | 0.935193518739489 | 1 | F | F | F | F | monocarboxylic acid binding | PA21B\_BOVIN | PA21B\_PIG | PTGDS\_HUMAN | PTGDS\_MOUSE | GO:0019904 | 0.00650230289894338 | 24/3691 | 2/641 | 0.938489854259169 | 1 | F | F | F | F | protein domain specific binding | BGLR\_HUMAN | TTHY\_CHICK | GO:0016791 | 0.031698726632349 | 117/3691 | 14/641 | 0.959859517067836 | 1 | F | F | F | F | phosphatase activity | A2TBB4\_9ASCO | A4GX63\_TOXGO | F6MIW5\_WHEAT | PHYA\_ASPFU | PHYA\_ASPNG | PHYB\_ASPAW | PPA5\_HUMAN | PPA5\_PIG | PPA5\_RAT | PPAF1\_IPOBA | PPAF\_PHAVU | PPAP\_RAT | Q8VX11\_LUPLU | V5NTD\_NAJAT | GO:0044877 | 0.0124627472229748 | 46/3691 | 4/641 | 0.970239008082891 | 1 | F | F | F | F | protein-containing complex binding | AOC1\_HUMAN | TLP\_PRUAV | TTHY\_MOUSE | TTHY\_RAT | GO:0048029 | 0.0132755350853427 | 49/3691 | 4/641 | 0.980164234782791 | 1 | F | F | F | F | monosaccharide binding | BGALA\_ASPOR | BGALA\_PENSQ | DOPO\_HUMAN | MANBA\_MOUSE | GO:0003723 | 0.0330533730696288 | 122/3691 | 13/641 | 0.98680715891657 | 1 | F | F | F | F | RNA binding | ANGI\_MOUSE | B1Q4V2\_HERER | Q45U61\_ASPNG | Q7XZV5\_NICGU | Q9M7C7\_CALSE | Q9SSV1\_NICGU | RNF1\_GIBFU | RNLE\_SOLLC | RNRH\_RHINI | RNS11\_NICAL | RNS3\_PYRPY | RNSL3\_DANRE | RNT2\_HUMAN | GO:0004602 | 0.0119208886480629 | 44/3691 | 3/641 | 0.988574765503654 | 1 | F | F | F | F | glutathione peroxidase activity | GPX3\_HUMAN | GPX5\_HUMAN | GPX6\_MOUSE | GO:0016701 | 0.0135464643727987 | 50/3691 | 3/641 | 0.995468363343307 | 1 | F | F | F | F | oxidoreductase activity, acting on single donors with incorporation of molecular oxygen | LUCI\_OPLGR | MNLOX\_MAGO7 | PGH2\_HUMAN | GO:0016721 | 0.010837171498239 | 40/3691 | 2/641 | 0.995582067284525 | 1 | F | F | F | F | oxidoreductase activity, acting on superoxide radicals as acceptor | Q08J22\_BOMMO | SODE\_ONCVO | GO:0004784 | 0.010837171498239 | 40/3691 | 2/641 | 0.995582067284525 | 1 | F | F | F | F | superoxide dismutase activity | Q08J22\_BOMMO | SODE\_ONCVO | GO:0016702 | 0.0111081007856949 | 41/3691 | 2/641 | 0.996275118731983 | 1 | F | F | F | F | oxidoreductase activity, acting on single donors with incorporation of molecular oxygen, incorporation of two atoms of oxygen | MNLOX\_MAGO7 | PGH2\_HUMAN | GO:0004721 | 0.0113790300731509 | 42/3691 | 2/641 | 0.996861131705774 | 1 | F | F | F | F | phosphoprotein phosphatase activity | A4GX63\_TOXGO | PPAP\_RAT | GO:0140101 | 0.0124627472229748 | 46/3691 | 2/641 | 0.998425088159911 | 1 | F | F | F | F | catalytic activity, acting on a tRNA | ANGI\_MOUSE | Q5WRG2\_RAT | GO:0016757 | 0.0327824437821729 | 121/3691 | 9/641 | 0.999599922673683 | 1 | F | F | F | F | glycosyltransferase activity | GBA1\_HUMAN | HYAL1\_HUMAN | LALBA\_BOVIN | LALBA\_CAPHI | LALBA\_CAVPO | LALBA\_PAPCY | OFUT1\_CAEEL | Q07524\_TROMA | XTH34\_POPPZ | GO:0016835 | 0.041181251693308 | 152/3691 | 12/641 | 0.999809966539234 | 1 | F | F | F | F | carbon-oxygen lyase activity | CAH1\_CHLRE | CAH6\_HUMAN | DIR\_GLYEC | G2X3Y1\_VERDV | NANL\_MACDE | NCS\_THLFG | PELA\_ASPNG | PELB\_ASPNG | PLY1\_JUNAS | Q2TWF5\_ASPOR | Q9STC1\_GRALE | RGLA\_ASPAC | GO:0016810 | 0.0322405852072609 | 119/3691 | 8/641 | 0.999837787669143 | 1 | F | F | F | F | hydrolase activity, acting on carbon-nitrogen (but not peptide) bonds | A0A0R4I979\_BRABE | A2QZC8\_ASPNC | ADA2\_HUMAN | ASAH1\_BALAS | CDA\_COLLN | CDA\_EMENI | PGRP1\_CAMDR | Q86RS6\_MANSE | GO:0019899 | 0.0222162015713899 | 82/3691 | 4/641 | 0.999859287292638 | 1 | F | F | F | F | enzyme binding | CHIA\_HUMAN | CHIL3\_MOUSE | PGH2\_HUMAN | PRTN3\_HUMAN | GO:0016836 | 0.0270929287455974 | 100/3691 | 5/641 | 0.999958953822304 | 1 | F | F | F | F | hydro-lyase activity | CAH1\_CHLRE | CAH6\_HUMAN | DIR\_GLYEC | NCS\_THLFG | Q2TWF5\_ASPOR | GO:0043177 | 0.0273638580330534 | 101/3691 | 5/641 | 0.999964919119617 | 1 | F | F | F | F | organic acid binding | DOPO\_HUMAN | PA21B\_BOVIN | PA21B\_PIG | PTGDS\_HUMAN | PTGDS\_MOUSE | GO:0031406 | 0.0273638580330534 | 101/3691 | 5/641 | 0.999964919119617 | 1 | F | F | F | F | carboxylic acid binding | DOPO\_HUMAN | PA21B\_BOVIN | PA21B\_PIG | PTGDS\_HUMAN | PTGDS\_MOUSE | GO:0017111 | 0.0192359794093742 | 71/3691 | 2/641 | 0.999981499766243 | 1 | F | F | F | F | ribonucleoside triphosphate phosphatase activity | NTP1\_TOXGO | NTP2\_TOXGO | GO:0051213 | 0.0200487672717421 | 74/3691 | 2/641 | 0.999989272779103 | 1 | F | F | F | F | dioxygenase activity | MNLOX\_MAGO7 | PGH2\_HUMAN | GO:0004497 | 0.0249254944459496 | 92/3691 | 3/641 | 0.999995932390723 | 1 | F | F | F | F | monooxygenase activity | DOPO\_HUMAN | LUCI\_OPLGR | PPO8\_ANOGA | GO:0016705 | 0.0284475751828773 | 105/3691 | 4/641 | 0.999996791890505 | 1 | F | F | F | F | oxidoreductase activity, acting on paired donors, with incorporation or reduction of molecular oxygen | DOPO\_HUMAN | PGH2\_HUMAN | POXA\_DICDI | PRXC\_LEPFU | GO:0042803 | 0.0585207260904904 | 216/3691 | 15/641 | 0.999998737075004 | 1 | F | F | F | F | protein homodimerization activity | ADA2\_HUMAN | AGAL\_HUMAN | ANG2\_MOUSE | ANG3\_MOUSE | ANG4\_MOUSE | ANGI\_MOUSE | AOC1\_HUMAN | GRAA\_HUMAN | IDH\_OSTTA | LYS\_RUDPH | NAGAB\_HUMAN | PGH2\_HUMAN | PPAP\_RAT | Q5WRG2\_RAT | RIPG\_SURMU | GO:0005506 | 0.0338661609319967 | 125/3691 | 5/641 | 0.99999927661792 | 1 | F | F | F | F | iron ion binding | PPA5\_HUMAN | PPA5\_PIG | PPA5\_RAT | PPAF\_PHAVU | TRFL\_BUBBU | GO:0005488 | 0.665673259279328 | 2457/3691 | 374/641 | 0.999999338876902 | 1 | F | F | F | F | binding | A0A060SC37\_PYCCI | A0A075B5H6\_TRIHA | A0A086SY89\_ACRC1 | A0A087WNH2\_FICBE | A0A088T0J9\_GEOCN | A0A098DND1\_GIBZE | A0A0A0Y4H8\_TRAFO | A0A0A7M685\_TRAHI | A0A0J5Q413\_ASPFM | A0A0M3U1T9\_9APHY | A0A0R4I979\_BRABE | A0A0S2GKZ1\_9APHY | A0A173N065\_EISFE | A0A183C5H8\_GLOPA | A0A1D3S5H0\_FUSOX | A0A1L8D5Z7\_BOTAT | A0A1L9WG58\_ASPA1 | A0A1S4NYF8\_PANVG | A0A1S9DW10\_ASPOZ | A0A1Y2TH07\_9PEZI | A0A2H4A2Q2\_9APHY | A0A2N1LTK3\_TRIHA | A0A2P1C6N4\_IRPLA | A0A3B6UEQ2\_RHIMI | A0A3F2YLU5\_9APHY | A0A3L6SKP5\_PANMI | A0A482LWB1\_OSTFU | A0A5J6BJN2\_MALCI | A0NFU8\_ANOGA | A1HA\_LOXIN | A1HB2\_LOXIN | A2QIR3\_ASPNC | A2QS62\_ASPNC | A311\_LOXLA | A5AB48\_ASPNC | A8NI40\_COPC7 | A9LI60\_BIOOC | ADA2\_HUMAN | AGAL\_HUMAN | AMPS2\_LITPI | AMY1A\_HUMAN | AMY1\_HORVU | AMY1\_ORYSJ | AMYA1\_ASPOR | AMY\_ORYLA | ANG1\_BOVIN | ANG2\_MOUSE | ANG3\_MOUSE | ANG4\_MOUSE | ANGI\_MOUSE | AOAH\_MOUSE | AOC1\_HUMAN | AOCX\_BOVIN | APO1\_CYCAE | ASM3A\_HUMAN | ASM3A\_MOUSE | ATLE\_CYCAE | B1Q4V2\_HERER | B2L9C1\_TRAHI | B4F320\_LIMPO | B7X9Z0\_COPCI | B8MX95\_ASPFN | BGALA\_ASPNC | BGALA\_ASPOR | BGALA\_PENSQ | BGLR\_HUMAN | BLRO\_ALBVE | CAH1\_CHLRE | CAH6\_HUMAN | CAT3\_NEUCR | CATD\_RAT | CATH\_HUMAN | CBHB\_ASPFU | CBPA1\_PIG | CBPD\_LOPSP | CBPN\_HUMAN | CDA\_COLLN | CDA\_EMENI | CERU\_RAT | CHI1\_COCPS | CHI2\_ORYSJ | CHI33\_TRIHA | CHI42\_TRIHA | CHI4\_CRYJA | CHIA\_HUMAN | CHIC\_ARATH | CHIC\_SECCE | CHIL3\_MOUSE | CHIT\_PUNGR | CKX1\_MAIZE | CYND4\_CYNDA | CYP5\_CAEEL | D1MPT2\_ROYRE | D7F485\_9APHY | DABA\_PSEMU | DNAS1\_HUMAN | DNSL3\_HUMAN | DOPO\_HUMAN | DYP\_AURAJ | E7FH77\_DANRE | ECP\_HUMAN | ENDO2\_ARATH | ENPP2\_HUMAN | ENPP2\_RAT | EXG1\_CANAL | F1CYZ0\_TALFU | F2Z7L1\_9ANNE | F6MIW5\_WHEAT | F6N9E7\_9PEZI | FAEA\_ASPNG | FAEB1\_ASPOR | FAEB2\_ASPOR | G0RVK1\_HYPJQ | G0SAW6\_CHATD | G2PZJ2\_MYCTT | G2Q665\_MYCTT | G2QG31\_MYCTT | G9NTY1\_HYPAI | GALNS\_HUMAN | GANA\_HUMIN | GAOA\_GIBZA | GBA1\_HUMAN | GCE\_CERUI | GCE\_HYPJQ | GOOX\_SARSR | GOX\_ASPNG | GPX3\_HUMAN | GRAA\_HUMAN | GRASS\_DROME | GUN2\_HYPJE | GUN7\_HYPJQ | GUX1\_HUMGT | GUX1\_HYPJE | GUX1\_TRIHA | GUX2\_HYPJE | H1AE14\_PHACH | H8ZRU2\_9HELO | HE12\_DANRE | I1SB14\_9APHY | I1SB18\_VIPAE | I1VE66\_9APHY | I2FI81\_EISFE | IDH\_OSTTA | IDUA\_HUMAN | K7CID1\_PANTR | K7N5L9\_RAPSA | K9L8F3\_MALCI | KATG2\_MAGO7 | L8ICE9\_9CETA | LAC1\_MELAO | LAC1\_TRAMX | LAC2\_TRAVE | LALBA\_BOVIN | LALBA\_CAPHI | LALBA\_CAVPO | LALBA\_PAPCY | LAPA\_ASPOR | LCN15\_HUMAN | LIG2\_PHACH | LIG4\_PHACH | LIG8\_PHACH | LIPG\_HUMAN | LIPP\_HORSE | LIPR1\_CANLF | LIPR1\_HUMAN | LIPR2\_HUMAN | LIPR2\_RAT | LYSC1\_CANLF | LYSC1\_HORSE | LYSC\_EQUAS | LYS\_RUDPH | M1GME7\_9APHY | M2RAI8\_CERS8 | M9TI89\_RHIPU | MAN12\_PENCI | MANA\_CANEN | MANA\_CRYAT | MANA\_HYPJR | MANBA\_MOUSE | MDL1\_PRUDU | MMP1\_PIG | MNCO\_MICNN | MNLOX\_MAGO7 | NADA\_ASPFU | NAGAB\_HUMAN | NP1\_RHOPR | NP2\_RHOPR | NP4\_RHOPR | NUP1\_PENCI | NUS1\_ASPOR | O22443\_SOYBN | O77044\_9NEOP | O81226\_CARPA | O81934\_CANEN | O94219\_PLEER | O97389\_HELAM | OXLA\_BOTAT | PA21B\_BOVIN | PA21B\_PIG | PA2A1\_BUNCE | PA2A1\_ECHCA | PA2A1\_NAJAT | PA2A1\_OPHHA | PA2A2\_NAJNA | PA2A2\_OPHHA | PA2A2\_TROCA | PA2A4\_NAJSG | PA2A5\_TRIST | PA2A7\_GLOHA | PA2A\_BOTJR | PA2A\_CROAT | PA2A\_DEIAC | PA2A\_GLOHA | PA2A\_NAJAT | PA2B1\_AGKPI | PA2B2\_BOTJR | PA2B2\_PROFL | PA2B3\_BOTAS | PA2B3\_BUNCE | PA2B5\_BUNCE | PA2B5\_NOTSC | PA2BA\_VIPAA | PA2BB\_GLOHA | PA2BB\_PSEAU | PA2BC\_VIPAA | PA2BD\_CRODU | PA2B\_BUNCE | PA2B\_NOTSC | PA2GA\_HUMAN | PA2GE\_HUMAN | PA2GX\_HUMAN | PA2H1\_AGKCL | PA2H1\_BOTBZ | PA2H1\_BOTJR | PA2H1\_BOTMO | PA2H1\_BOTPI | PA2H2\_BOTAS | PA2H2\_BOTMO | PA2H2\_BOTPI | PA2H2\_CERGO | PA2H3\_BOTPI | PA2HB\_AGKPI | PA2HB\_OXYSC | PA2HH\_TRIST | PA2HS\_ECHCA | PA2H\_BOTPA | PA2H\_DEIAC | PA2H\_PROMB | PA2N\_GLOHA | PA2\_APIME | PAG15\_HUMAN | PDE\_NAJAT | PDH1\_LEUMG | PEM1\_PHACH | PER1A\_ARMRU | PER1\_ARAHY | PER1\_SORBI | PER53\_ARATH | PER59\_ARATH | PERL\_BOVIN | PERL\_BUBBU | PERL\_CAPHI | PER\_ARTRA | PER\_COPCI | PGH2\_HUMAN | PGPSA\_DROME | PGRP1\_CAMDR | PLA22\_ORYSJ | PLY1\_JUNAS | POXA\_DICDI | PPA5\_HUMAN | PPA5\_PIG | PPA5\_RAT | PPAF1\_HOLDI | PPAF1\_IPOBA | PPAF\_PHAVU | PPAP\_RAT | PPO8\_ANOGA | PPT1\_BOVIN | PPT1\_HUMAN | PRS57\_HUMAN | PRTN3\_HUMAN | PRXC\_LEPFU | PTGDS\_HUMAN | PTGDS\_MOUSE | Q02321\_PHACH | Q06AK3\_TOXGO | Q07524\_TROMA | Q08J22\_BOMMO | Q0KFV0\_SOLLC | Q12571\_9BASI | Q1K4Q1\_NEUCR | Q1W6B1\_9APHY | Q2TWF5\_ASPOR | Q40069\_HORVU | Q43576\_TOBAC | Q45U61\_ASPNG | Q4AE59\_OSTFU | Q4W6L6\_CYCRE | Q4WP32\_ASPFU | Q50KB2\_PHACH | Q55FE6\_DICDI | Q5B038\_EMENI | Q5EBY5\_9APHY | Q5WRG2\_RAT | Q60FD2\_9APHY | Q6H9H7\_9APHY | Q6WSR8\_PICAB | Q7LHI2\_PHACH | Q7LIJ0\_PHACH | Q7XZV5\_NICGU | Q86PT9\_RHOPR | Q86RS6\_MANSE | Q8H0C9\_VIGUN | Q8TG94\_TRAPU | Q8VX11\_LUPLU | Q8WZK8\_9APHY | Q92458\_HYPJE | Q95KP4\_HORSE | Q96TR6\_PYCCO | Q96UT7\_TRAVE | Q96X16\_PICPA | Q9FUH3\_VIGUS | Q9HDQ0\_9APHY | Q9M7C7\_CALSE | Q9PTT3\_SPAAU | Q9SSV1\_NICGU | Q9STC1\_GRALE | Q9UVQ2\_PYCCI | Q9Y780\_COPCI | QPCT1\_DROME | QPCT2\_DROME | QPCT\_IXOSC | QPCT\_MOUSE | RENI\_RAT | RGLA\_ASPAC | RIPG\_SURMU | RNAS1\_BISBI | RNAS4\_HUMAN | RNAS4\_PIG | RNAS6\_HUMAN | RNF1\_GIBFU | RNLE\_SOLLC | RNRH\_RHINI | RNS11\_NICAL | RNS1B\_RAT | RNS3\_PYRPY | RNSL3\_DANRE | RNS\_BOVIN | RNT2\_HUMAN | S7ZIW0\_PENO1 | SODE\_ONCVO | THCAS\_CANSA | TLP\_PRUAV | TPP1\_HUMAN | TRFL\_BUBBU | TRFL\_HORSE | TRY3\_SALSA | TTHY\_CHICK | TTHY\_MOUSE | TTHY\_RAT | V5NTD\_NAJAT | VM11\_BOTMO | VM12\_CROAD | VM1A3\_DEIAC | VM1BI\_BOTMO | VM1T1\_PROMU | VM1T2\_PROFL | VPL1\_PLEER | VPL2\_PLEER | W4KMP1\_HETIT | XTH34\_POPPZ | XYLA\_ASPNC | XYLO\_MYCTT | GO:0016860 | 0.0243836358710377 | 90/3691 | 2/641 | 0.999999431693945 | 1 | F | F | F | F | intramolecular oxidoreductase activity | PTGDS\_HUMAN | PTGDS\_MOUSE | GO:0046983 | 0.0658358168518017 | 243/3691 | 16/641 | 0.99999989617175 | 1 | F | F | F | F | protein dimerization activity | ADA2\_HUMAN | AGAL\_HUMAN | ANG2\_MOUSE | ANG3\_MOUSE | ANG4\_MOUSE | ANGI\_MOUSE | AOC1\_HUMAN | GRAA\_HUMAN | IDH\_OSTTA | LYS\_RUDPH | NAGAB\_HUMAN | PGH2\_HUMAN | PPAP\_RAT | Q5WRG2\_RAT | RIPG\_SURMU | TTHY\_CHICK | GO:0043167 | 0.507992413979951 | 1875/3691 | 265/641 | 0.99999994846902 | 1 | F | F | F | F | ion binding | A0A060SC37\_PYCCI | A0A075B5H6\_TRIHA | A0A087WNH2\_FICBE | A0A098DND1\_GIBZE | A0A0A0Y4H8\_TRAFO | A0A0A7M685\_TRAHI | A0A0M3U1T9\_9APHY | A0A0R4I979\_BRABE | A0A0S2GKZ1\_9APHY | A0A173N065\_EISFE | A0A183C5H8\_GLOPA | A0A1D3S5H0\_FUSOX | A0A1L8D5Z7\_BOTAT | A0A1S4NYF8\_PANVG | A0A1S9DW10\_ASPOZ | A0A1Y2TH07\_9PEZI | A0A2H4A2Q2\_9APHY | A0A3F2YLU5\_9APHY | A0A3L6SKP5\_PANMI | A0NFU8\_ANOGA | A1HA\_LOXIN | A1HB2\_LOXIN | A2QIR3\_ASPNC | A2QS62\_ASPNC | A311\_LOXLA | ADA2\_HUMAN | AMY1A\_HUMAN | AMY1\_HORVU | AMY1\_ORYSJ | AMYA1\_ASPOR | AMY\_ORYLA | ANG1\_BOVIN | ANG2\_MOUSE | ANG3\_MOUSE | ANG4\_MOUSE | ANGI\_MOUSE | AOAH\_MOUSE | AOC1\_HUMAN | AOCX\_BOVIN | APO1\_CYCAE | ASM3A\_HUMAN | ASM3A\_MOUSE | B2L9C1\_TRAHI | B4F320\_LIMPO | B7X9Z0\_COPCI | B8MX95\_ASPFN | BLRO\_ALBVE | CAH1\_CHLRE | CAH6\_HUMAN | CAT3\_NEUCR | CBPA1\_PIG | CBPD\_LOPSP | CBPN\_HUMAN | CDA\_COLLN | CDA\_EMENI | CERU\_RAT | CHIT\_PUNGR | CKX1\_MAIZE | CYND4\_CYNDA | D1MPT2\_ROYRE | D7F485\_9APHY | DABA\_PSEMU | DNSL3\_HUMAN | DOPO\_HUMAN | DYP\_AURAJ | ENDO2\_ARATH | ENPP2\_HUMAN | ENPP2\_RAT | F2Z7L1\_9ANNE | F6MIW5\_WHEAT | F6N9E7\_9PEZI | FAEB1\_ASPOR | FAEB2\_ASPOR | G0SAW6\_CHATD | G2PZJ2\_MYCTT | G2QG31\_MYCTT | GALNS\_HUMAN | GAOA\_GIBZA | GOOX\_SARSR | GOX\_ASPNG | GRASS\_DROME | GUN7\_HYPJQ | H1AE14\_PHACH | H8ZRU2\_9HELO | HE12\_DANRE | I1SB14\_9APHY | I1SB18\_VIPAE | I1VE66\_9APHY | I2FI81\_EISFE | IDH\_OSTTA | K7CID1\_PANTR | K7N5L9\_RAPSA | K9L8F3\_MALCI | KATG2\_MAGO7 | L8ICE9\_9CETA | LAC1\_MELAO | LAC1\_TRAMX | LAC2\_TRAVE | LALBA\_BOVIN | LALBA\_CAPHI | LALBA\_CAVPO | LALBA\_PAPCY | LAPA\_ASPOR | LIG2\_PHACH | LIG4\_PHACH | LIG8\_PHACH | LIPP\_HORSE | LIPR1\_CANLF | LIPR1\_HUMAN | LIPR2\_HUMAN | LIPR2\_RAT | LYSC1\_CANLF | LYSC1\_HORSE | LYSC\_EQUAS | M1GME7\_9APHY | M9TI89\_RHIPU | MAN12\_PENCI | MANA\_CANEN | MDL1\_PRUDU | MMP1\_PIG | MNCO\_MICNN | MNLOX\_MAGO7 | NADA\_ASPFU | NP1\_RHOPR | NP2\_RHOPR | NP4\_RHOPR | NUP1\_PENCI | NUS1\_ASPOR | O22443\_SOYBN | O77044\_9NEOP | O81226\_CARPA | O94219\_PLEER | O97389\_HELAM | OXLA\_BOTAT | PA21B\_BOVIN | PA21B\_PIG | PA2A1\_BUNCE | PA2A1\_ECHCA | PA2A1\_NAJAT | PA2A1\_OPHHA | PA2A2\_NAJNA | PA2A2\_OPHHA | PA2A2\_TROCA | PA2A4\_NAJSG | PA2A5\_TRIST | PA2A7\_GLOHA | PA2A\_BOTJR | PA2A\_CROAT | PA2A\_DEIAC | PA2A\_GLOHA | PA2A\_NAJAT | PA2B1\_AGKPI | PA2B2\_BOTJR | PA2B2\_PROFL | PA2B3\_BOTAS | PA2B3\_BUNCE | PA2B5\_BUNCE | PA2B5\_NOTSC | PA2BA\_VIPAA | PA2BB\_GLOHA | PA2BB\_PSEAU | PA2BC\_VIPAA | PA2BD\_CRODU | PA2B\_BUNCE | PA2B\_NOTSC | PA2GA\_HUMAN | PA2GE\_HUMAN | PA2GX\_HUMAN | PA2H1\_AGKCL | PA2H1\_BOTBZ | PA2H1\_BOTJR | PA2H1\_BOTMO | PA2H1\_BOTPI | PA2H2\_BOTAS | PA2H2\_BOTMO | PA2H2\_BOTPI | PA2H2\_CERGO | PA2H3\_BOTPI | PA2HB\_AGKPI | PA2HB\_OXYSC | PA2HH\_TRIST | PA2HS\_ECHCA | PA2H\_BOTPA | PA2H\_DEIAC | PA2H\_PROMB | PA2N\_GLOHA | PA2\_APIME | PAG15\_HUMAN | PDE\_NAJAT | PDH1\_LEUMG | PEM1\_PHACH | PER1A\_ARMRU | PER1\_ARAHY | PER1\_SORBI | PER53\_ARATH | PER59\_ARATH | PERL\_BOVIN | PERL\_BUBBU | PERL\_CAPHI | PER\_ARTRA | PER\_COPCI | PGH2\_HUMAN | PGPSA\_DROME | PGRP1\_CAMDR | PLA22\_ORYSJ | PLY1\_JUNAS | PPA5\_HUMAN | PPA5\_PIG | PPA5\_RAT | PPAF1\_HOLDI | PPAF1\_IPOBA | PPAF\_PHAVU | PPAP\_RAT | PPO8\_ANOGA | PPT1\_HUMAN | PRXC\_LEPFU | PTGDS\_HUMAN | PTGDS\_MOUSE | Q06AK3\_TOXGO | Q07524\_TROMA | Q08J22\_BOMMO | Q0KFV0\_SOLLC | Q12571\_9BASI | Q1K4Q1\_NEUCR | Q1W6B1\_9APHY | Q2TWF5\_ASPOR | Q40069\_HORVU | Q5B038\_EMENI | Q5EBY5\_9APHY | Q5WRG2\_RAT | Q60FD2\_9APHY | Q6H9H7\_9APHY | Q86PT9\_RHOPR | Q86RS6\_MANSE | Q8TG94\_TRAPU | Q8VX11\_LUPLU | Q8WZK8\_9APHY | Q92458\_HYPJE | Q95KP4\_HORSE | Q96TR6\_PYCCO | Q96UT7\_TRAVE | Q96X16\_PICPA | Q9HDQ0\_9APHY | Q9UVQ2\_PYCCI | Q9Y780\_COPCI | QPCT1\_DROME | QPCT2\_DROME | QPCT\_IXOSC | QPCT\_MOUSE | SODE\_ONCVO | THCAS\_CANSA | TPP1\_HUMAN | TRFL\_BUBBU | TRFL\_HORSE | TRY3\_SALSA | V5NTD\_NAJAT | VM11\_BOTMO | VM12\_CROAD | VM1A3\_DEIAC | VM1BI\_BOTMO | VM1T1\_PROMU | VM1T2\_PROFL | VPL1\_PLEER | VPL2\_PLEER | XYLO\_MYCTT | GO:0016829 | 0.103494987808182 | 382/3691 | 32/641 | 0.999999960382072 | 1 | F | F | F | F | lyase activity | A1HA\_LOXIN | A1HB2\_LOXIN | A311\_LOXLA | CAH1\_CHLRE | CAH6\_HUMAN | DIR\_GLYEC | G2X3Y1\_VERDV | LYS\_RUDPH | MDL1\_PRUDU | NANL\_MACDE | NCS\_THLFG | PELA\_ASPNG | PELB\_ASPNG | PHNH\_PENHR | PLY1\_JUNAS | Q2TWF5\_ASPOR | Q45U61\_ASPNG | Q7XZV5\_NICGU | Q9LYJ5\_ARATH | Q9M7C7\_CALSE | Q9SSV1\_NICGU | Q9STC1\_GRALE | RGLA\_ASPAC | RNAS1\_BISBI | RNF1\_GIBFU | RNLE\_SOLLC | RNRH\_RHINI | RNS11\_NICAL | RNS1B\_RAT | RNS3\_PYRPY | RNS\_BOVIN | RNT2\_HUMAN | GO:0016746 | 0.0430777567054999 | 159/3691 | 6/641 | 0.999999984375126 | 1 | F | F | F | F | acyltransferase activity | O81226\_CARPA | PAG15\_HUMAN | QPCT1\_DROME | QPCT2\_DROME | QPCT\_IXOSC | QPCT\_MOUSE | GO:0016462 | 0.0344080195069087 | 127/3691 | 3/641 | 0.999999992205 | 1 | F | F | F | F | pyrophosphatase activity | NTP1\_TOXGO | NTP2\_TOXGO | PDE\_NAJAT | GO:0016818 | 0.0346789487943647 | 128/3691 | 3/641 | 0.999999993503469 | 1 | F | F | F | F | hydrolase activity, acting on acid anhydrides, in phosphorus-containing anhydrides | NTP1\_TOXGO | NTP2\_TOXGO | PDE\_NAJAT | GO:0016817 | 0.0346789487943647 | 128/3691 | 3/641 | 0.999999993503469 | 1 | F | F | F | F | hydrolase activity, acting on acid anhydrides | NTP1\_TOXGO | NTP2\_TOXGO | PDE\_NAJAT | GO:0016614 | 0.0755892712002167 | 279/3691 | 13/641 | 0.999999999988333 | 1 | F | F | F | F | oxidoreductase activity, acting on CH-OH group of donors | A0A060SC37\_PYCCI | A0A1S9DW10\_ASPOZ | B8MX95\_ASPFN | G0SAW6\_CHATD | G2PZJ2\_MYCTT | GAOA\_GIBZA | GOX\_ASPNG | IDH\_OSTTA | LIPG\_HUMAN | MDL1\_PRUDU | MNCO\_MICNN | O94219\_PLEER | PDH1\_LEUMG | GO:0097367 | 0.149823895963154 | 553/3691 | 45/641 | 0.999999999989283 | 1 | F | F | F | F | carbohydrate derivative binding | A0A0R4I979\_BRABE | A0A183C5H8\_GLOPA | A0A3B6UEQ2\_RHIMI | A0A482LWB1\_OSTFU | A5AB48\_ASPNC | A9LI60\_BIOOC | ADA2\_HUMAN | AGAL\_HUMAN | ANG1\_BOVIN | ANG2\_MOUSE | ANG3\_MOUSE | ANG4\_MOUSE | ANGI\_MOUSE | AOC1\_HUMAN | CDA\_COLLN | CDA\_EMENI | CHI1\_COCPS | CHI2\_ORYSJ | CHI33\_TRIHA | CHI42\_TRIHA | CHI4\_CRYJA | CHIA\_HUMAN | CHIC\_ARATH | CHIC\_SECCE | CHIL3\_MOUSE | ECP\_HUMAN | O81934\_CANEN | PA2H1\_AGKCL | PA2H1\_BOTJR | PA2H2\_BOTAS | PGPSA\_DROME | PGRP1\_CAMDR | PPT1\_BOVIN | PPT1\_HUMAN | PRS57\_HUMAN | Q06AK3\_TOXGO | Q43576\_TOBAC | Q4AE59\_OSTFU | Q4W6L6\_CYCRE | Q5WRG2\_RAT | Q6WSR8\_PICAB | Q86RS6\_MANSE | Q8H0C9\_VIGUN | Q9FUH3\_VIGUS | TPP1\_HUMAN | GO:0097159 | 0.352749932267678 | 1302/3691 | 95/641 | 0.999999999991876 | 1 | F | F | F | F | organic cyclic compound binding | A0A060SC37\_PYCCI | A0A087WNH2\_FICBE | A0A098DND1\_GIBZE | A0A0A0Y4H8\_TRAFO | A0A183C5H8\_GLOPA | A0A1S4NYF8\_PANVG | A0A1S9DW10\_ASPOZ | A0A2P1C6N4\_IRPLA | A0A3L6SKP5\_PANMI | AMPS2\_LITPI | ANG1\_BOVIN | ANG2\_MOUSE | ANG3\_MOUSE | ANG4\_MOUSE | ANGI\_MOUSE | APO1\_CYCAE | B1Q4V2\_HERER | B8MX95\_ASPFN | CAT3\_NEUCR | CKX1\_MAIZE | CYND4\_CYNDA | D1MPT2\_ROYRE | DNAS1\_HUMAN | DNSL3\_HUMAN | DOPO\_HUMAN | DYP\_AURAJ | E7FH77\_DANRE | ECP\_HUMAN | ENDO2\_ARATH | ENPP2\_HUMAN | ENPP2\_RAT | G0SAW6\_CHATD | G2PZJ2\_MYCTT | GOOX\_SARSR | GOX\_ASPNG | K7CID1\_PANTR | K7N5L9\_RAPSA | KATG2\_MAGO7 | L8ICE9\_9CETA | LIG2\_PHACH | LIG4\_PHACH | LIG8\_PHACH | MDL1\_PRUDU | MNCO\_MICNN | NP1\_RHOPR | NP2\_RHOPR | NP4\_RHOPR | NUP1\_PENCI | NUS1\_ASPOR | O22443\_SOYBN | O94219\_PLEER | PDE\_NAJAT | PDH1\_LEUMG | PEM1\_PHACH | PER1A\_ARMRU | PER1\_ARAHY | PER1\_SORBI | PER53\_ARATH | PER59\_ARATH | PERL\_BOVIN | PERL\_BUBBU | PERL\_CAPHI | PER\_ARTRA | PER\_COPCI | PGH2\_HUMAN | POXA\_DICDI | Q06AK3\_TOXGO | Q0KFV0\_SOLLC | Q40069\_HORVU | Q45U61\_ASPNG | Q5WRG2\_RAT | Q60FD2\_9APHY | Q7XZV5\_NICGU | Q86PT9\_RHOPR | Q8WZK8\_9APHY | Q9M7C7\_CALSE | Q9SSV1\_NICGU | RNAS1\_BISBI | RNAS4\_HUMAN | RNAS4\_PIG | RNAS6\_HUMAN | RNF1\_GIBFU | RNLE\_SOLLC | RNRH\_RHINI | RNS11\_NICAL | RNS1B\_RAT | RNS3\_PYRPY | RNSL3\_DANRE | RNS\_BOVIN | RNT2\_HUMAN | THCAS\_CANSA | V5NTD\_NAJAT | VPL1\_PLEER | VPL2\_PLEER | XYLO\_MYCTT | GO:0016853 | 0.0579788675155784 | 214/3691 | 5/641 | 0.999999999993223 | 1 | F | F | F | F | isomerase activity | A0A0R3QSA7\_9BILA | A0A3S5H5N2\_LEIDO | CYP5\_CAEEL | PTGDS\_HUMAN | PTGDS\_MOUSE | GO:0036094 | 0.302357084800867 | 1116/3691 | 30/641 | 0.999999999993292 | 1 | F | F | F | F | small molecule binding | A0A060SC37\_PYCCI | A0A098DND1\_GIBZE | A0A183C5H8\_GLOPA | A0A1S9DW10\_ASPOZ | B8MX95\_ASPFN | BGALA\_ASPOR | BGALA\_PENSQ | CKX1\_MAIZE | CYND4\_CYNDA | DOPO\_HUMAN | G0SAW6\_CHATD | G2PZJ2\_MYCTT | GOOX\_SARSR | GOX\_ASPNG | LCN15\_HUMAN | MANBA\_MOUSE | MDL1\_PRUDU | MNCO\_MICNN | O94219\_PLEER | PA21B\_BOVIN | PA21B\_PIG | PDH1\_LEUMG | PTGDS\_HUMAN | PTGDS\_MOUSE | Q06AK3\_TOXGO | Q9SSV1\_NICGU | THCAS\_CANSA | TTHY\_CHICK | V5NTD\_NAJAT | XYLO\_MYCTT | GO:0016491 | 0.278244378217285 | 1027/3691 | 103/641 | 0.999999999994259 | 1 | F | F | F | F | oxidoreductase activity | A0A060SC37\_PYCCI | A0A087WNH2\_FICBE | A0A097P6E1\_9FABA | A0A098DND1\_GIBZE | A0A0A0Y4H8\_TRAFO | A0A0A7M685\_TRAHI | A0A0M3U1T9\_9APHY | A0A1S4NYF8\_PANVG | A0A1S9DW10\_ASPOZ | A0A1Y2TH07\_9PEZI | A0A2H4A2Q2\_9APHY | A0A2P1C6N4\_IRPLA | A0A3F2YLU5\_9APHY | A0A3L6SKP5\_PANMI | A2QS62\_ASPNC | AOC1\_HUMAN | AOCX\_BOVIN | APO1\_CYCAE | B2L9C1\_TRAHI | B8MX95\_ASPFN | BLRO\_ALBVE | CAT3\_NEUCR | CERU\_RAT | CKX1\_MAIZE | CYND4\_CYNDA | D1MPT2\_ROYRE | D7F485\_9APHY | DOPO\_HUMAN | DYP\_AURAJ | F6N9E7\_9PEZI | G0SAW6\_CHATD | G2PZJ2\_MYCTT | G2QG31\_MYCTT | GAOA\_GIBZA | GILT\_MOUSE | GOOX\_SARSR | GOX\_ASPNG | GPX3\_HUMAN | GPX5\_HUMAN | GPX6\_MOUSE | H8ZRU2\_9HELO | I1SB14\_9APHY | I1VE66\_9APHY | IDH\_OSTTA | K7N5L9\_RAPSA | K7ZUA3\_ASCNO | KATG2\_MAGO7 | L8ICE9\_9CETA | LAC1\_MELAO | LAC1\_TRAMX | LAC2\_TRAVE | LIG2\_PHACH | LIG4\_PHACH | LIG8\_PHACH | LIPG\_HUMAN | LUCI\_OPLGR | M1GME7\_9APHY | MDL1\_PRUDU | MNCO\_MICNN | MNLOX\_MAGO7 | NP4\_RHOPR | O22443\_SOYBN | O94219\_PLEER | OXLA\_BOTAT | OXLA\_CALRH | OXLA\_GLOHA | PDH1\_LEUMG | PEM1\_PHACH | PER1A\_ARMRU | PER1\_ARAHY | PER1\_SORBI | PER53\_ARATH | PER59\_ARATH | PERL\_BOVIN | PERL\_BUBBU | PERL\_CAPHI | PER\_ARTRA | PER\_COPCI | PGH2\_HUMAN | POXA\_DICDI | PPO8\_ANOGA | PRXC\_LEPFU | Q08J22\_BOMMO | Q12571\_9BASI | Q1W6B1\_9APHY | Q40069\_HORVU | Q5B038\_EMENI | Q5EBY5\_9APHY | Q60FD2\_9APHY | Q6H9H7\_9APHY | Q8TG94\_TRAPU | Q8WZK8\_9APHY | Q96TR6\_PYCCO | Q96UT7\_TRAVE | Q96X16\_PICPA | Q9HDQ0\_9APHY | Q9UVQ2\_PYCCI | Q9Y780\_COPCI | SODE\_ONCVO | THCAS\_CANSA | VPL1\_PLEER | VPL2\_PLEER | XYLO\_MYCTT | GO:0000287 | 0.0484963424546193 | 179/3691 | 2/641 | 0.999999999995481 | 1 | F | F | F | F | magnesium ion binding | A0A183C5H8\_GLOPA | IDH\_OSTTA | GO:0035639 | 0.118937957193173 | 439/3691 | 2/641 | 0.99999999999626 | 1 | F | F | F | F | purine ribonucleoside triphosphate binding | A0A183C5H8\_GLOPA | Q06AK3\_TOXGO | GO:0043168 | 0.22541316716337 | 832/3691 | 26/641 | 0.99999999999695 | 1 | F | F | F | F | anion binding | A0A060SC37\_PYCCI | A0A098DND1\_GIBZE | A0A183C5H8\_GLOPA | A0A1S9DW10\_ASPOZ | AMY1A\_HUMAN | B8MX95\_ASPFN | CKX1\_MAIZE | CYND4\_CYNDA | DOPO\_HUMAN | G0SAW6\_CHATD | G2PZJ2\_MYCTT | GOOX\_SARSR | GOX\_ASPNG | MDL1\_PRUDU | MNCO\_MICNN | O94219\_PLEER | PA21B\_BOVIN | PA21B\_PIG | PDH1\_LEUMG | PPT1\_HUMAN | PTGDS\_HUMAN | PTGDS\_MOUSE | Q06AK3\_TOXGO | THCAS\_CANSA | TPP1\_HUMAN | XYLO\_MYCTT | GO:0042802 | 0.1292332701165 | 477/3691 | 23/641 | 0.999999999997112 | 1 | F | F | F | F | identical protein binding | ADA2\_HUMAN | AGAL\_HUMAN | ANG2\_MOUSE | ANG3\_MOUSE | ANG4\_MOUSE | ANGI\_MOUSE | AOC1\_HUMAN | GPX3\_HUMAN | GRAA\_HUMAN | GUX2\_HYPJE | IDH\_OSTTA | LALBA\_BOVIN | LYS\_RUDPH | NAGAB\_HUMAN | PA2A5\_TRIST | PGH2\_HUMAN | PPAP\_RAT | Q2TWF5\_ASPOR | Q5WRG2\_RAT | RIPG\_SURMU | RNS\_BOVIN | TTHY\_MOUSE | TTHY\_RAT | GO:0016740 | 0.280411812516933 | 1035/3691 | 21/641 | 0.999999999997161 | 1 | F | F | F | F | transferase activity | DABA\_PSEMU | EXG1\_CANAL | GBA1\_HUMAN | HS3S1\_MOUSE | HYAL1\_HUMAN | LALBA\_BOVIN | LALBA\_CAPHI | LALBA\_CAVPO | LALBA\_PAPCY | NADA\_APLCA | O74705\_ASPNG | O81226\_CARPA | OFUT1\_CAEEL | PAG15\_HUMAN | Q06AK3\_TOXGO | Q07524\_TROMA | QPCT1\_DROME | QPCT2\_DROME | QPCT\_IXOSC | QPCT\_MOUSE | XTH34\_POPPZ | GO:0032553 | 0.131129775128691 | 484/3691 | 2/641 | 0.99999999999746 | 1 | F | F | F | F | ribonucleotide binding | A0A183C5H8\_GLOPA | Q06AK3\_TOXGO | GO:0032559 | 0.106204280682742 | 392/3691 | 2/641 | 0.999999999997575 | 1 | F | F | F | F | adenyl ribonucleotide binding | A0A183C5H8\_GLOPA | Q06AK3\_TOXGO | GO:0030554 | 0.154158764562449 | 569/3691 | 2/641 | 0.999999999997637 | 1 | F | F | F | F | adenyl nucleotide binding | A0A183C5H8\_GLOPA | Q06AK3\_TOXGO | GO:0005515 | 0.167434299647792 | 618/3691 | 38/641 | 0.999999999997964 | 1 | F | F | F | F | protein binding | ADA2\_HUMAN | AGAL\_HUMAN | ANG1\_BOVIN | ANG2\_MOUSE | ANG3\_MOUSE | ANG4\_MOUSE | ANGI\_MOUSE | AOC1\_HUMAN | BGLR\_HUMAN | CERU\_RAT | CHIA\_HUMAN | CHIL3\_MOUSE | DNAS1\_HUMAN | EXG1\_CANAL | GBA1\_HUMAN | GPX3\_HUMAN | GRAA\_HUMAN | GUX2\_HYPJE | IDH\_OSTTA | IDUA\_HUMAN | LALBA\_BOVIN | LYS\_RUDPH | NAGAB\_HUMAN | PA21B\_BOVIN | PA21B\_PIG | PA2A5\_TRIST | PGH2\_HUMAN | PPAP\_RAT | PRTN3\_HUMAN | Q2TWF5\_ASPOR | Q5WRG2\_RAT | Q9PTT3\_SPAAU | RENI\_RAT | RIPG\_SURMU | RNS\_BOVIN | TTHY\_CHICK | TTHY\_MOUSE | TTHY\_RAT | GO:1901265 | 0.244649146572745 | 903/3691 | 20/641 | 0.999999999998431 | 1 | F | F | F | F | nucleoside phosphate binding | A0A060SC37\_PYCCI | A0A098DND1\_GIBZE | A0A183C5H8\_GLOPA | A0A1S9DW10\_ASPOZ | B8MX95\_ASPFN | CKX1\_MAIZE | CYND4\_CYNDA | G0SAW6\_CHATD | G2PZJ2\_MYCTT | GOOX\_SARSR | GOX\_ASPNG | MDL1\_PRUDU | MNCO\_MICNN | O94219\_PLEER | PDH1\_LEUMG | Q06AK3\_TOXGO | Q9SSV1\_NICGU | THCAS\_CANSA | V5NTD\_NAJAT | XYLO\_MYCTT | GO:0000166 | 0.244649146572745 | 903/3691 | 20/641 | 0.999999999998431 | 1 | F | F | F | F | nucleotide binding | A0A060SC37\_PYCCI | A0A098DND1\_GIBZE | A0A183C5H8\_GLOPA | A0A1S9DW10\_ASPOZ | B8MX95\_ASPFN | CKX1\_MAIZE | CYND4\_CYNDA | G0SAW6\_CHATD | G2PZJ2\_MYCTT | GOOX\_SARSR | GOX\_ASPNG | MDL1\_PRUDU | MNCO\_MICNN | O94219\_PLEER | PDH1\_LEUMG | Q06AK3\_TOXGO | Q9SSV1\_NICGU | THCAS\_CANSA | V5NTD\_NAJAT | XYLO\_MYCTT | GO:0005524 | 0.101327553508534 | 374/3691 | 2/641 | 0.999999999998692 | 1 | F | F | F | F | ATP binding | A0A183C5H8\_GLOPA | Q06AK3\_TOXGO | GO:0017076 | 0.171498238959632 | 633/3691 | 2/641 | 0.999999999998991 | 1 | F | F | F | F | purine nucleotide binding | A0A183C5H8\_GLOPA | Q06AK3\_TOXGO | GO:0032555 | 0.123543755079924 | 456/3691 | 2/641 | 0.999999999999074 | 1 | F | F | F | F | purine ribonucleotide binding | A0A183C5H8\_GLOPA | Q06AK3\_TOXGO | GO:1901363 | 0.280140883229477 | 1034/3691 | 21/641 | 1 | 1 | F | F | F | F | heterocyclic compound binding | A0A060SC37\_PYCCI | A0A098DND1\_GIBZE | A0A183C5H8\_GLOPA | A0A1S9DW10\_ASPOZ | B8MX95\_ASPFN | CKX1\_MAIZE | CYND4\_CYNDA | DOPO\_HUMAN | G0SAW6\_CHATD | G2PZJ2\_MYCTT | GOOX\_SARSR | GOX\_ASPNG | MDL1\_PRUDU | MNCO\_MICNN | O94219\_PLEER | PDH1\_LEUMG | Q06AK3\_TOXGO | Q9SSV1\_NICGU | THCAS\_CANSA | V5NTD\_NAJAT | XYLO\_MYCTT | GO:0003674 | 1 | 3691/3691 | 641/641 | 1 | 1 | F | F | F | F | molecular\_function | A0A059U759\_9PEZI | A0A060N399\_9PLEO | A0A060SC37\_PYCCI | A0A068FT77\_9PEZI | A0A075B5G4\_HUMIN | A0A075B5H6\_TRIHA | A0A075C6T6\_RHIMI | A0A086SY89\_ACRC1 | A0A086T6R4\_ACRC1 | A0A087WNH2\_FICBE | A0A088T0J9\_GEOCN | A0A097P6E1\_9FABA | A0A098DND1\_GIBZE | A0A0A0Y4H8\_TRAFO | A0A0A7M685\_TRAHI | A0A0J5Q413\_ASPFM | A0A0M3KKZ6\_RHIMI | A0A0M3KKZ8\_RHIMI | A0A0M3U1T9\_9APHY | A0A0R3QSA7\_9BILA | A0A0R4I979\_BRABE | A0A0S2GKZ1\_9APHY | A0A158RFS0\_MUCPR | A0A173N065\_EISFE | A0A183C5H8\_GLOPA | A0A1D3S5H0\_FUSOX | A0A1L6CE30\_9EURO | A0A1L8D5Z7\_BOTAT | A0A1L9WG58\_ASPA1 | A0A1S4NYF8\_PANVG | A0A1S6YJF3\_MALCI | A0A1S9DRB1\_ASPOZ | A0A1S9DW10\_ASPOZ | A0A1Y2TH07\_9PEZI | A0A2H4A2Q2\_9APHY | A0A2H5BN17\_TALPI | A0A2N1LTK3\_TRIHA | A0A2P1C6N4\_IRPLA | A0A2U8ZTY7\_RHIZD | A0A2Z4HIN9\_9EURO | A0A384E148\_NICBE | A0A3B6UEQ2\_RHIMI | A0A3B6UEQ6\_EISFE | A0A3F2YLU5\_9APHY | A0A3G2C3I4\_9EURO | A0A3G4RHU4\_9PEZI | A0A3L6SKP5\_PANMI | A0A3S5H5N2\_LEIDO | A0A482LWB1\_OSTFU | A0A5J6BJN2\_MALCI | A0A6F8Z6Y2\_BOMMO | A0A6M9BP13\_9EURO | A0A6P6YAT6\_DERPT | A0A7S6G7I6\_9PEZI | A0A856TAI5\_9BASI | A0NFU8\_ANOGA | A1E266\_9PEZI | A1HA\_LOXIN | A1HB2\_LOXIN | A2QIR3\_ASPNC | A2QS62\_ASPNC | A2QZC8\_ASPNC | A2TBB4\_9ASCO | A2TM14\_HEVBR | A311\_LOXLA | A4GX63\_TOXGO | A5AB48\_ASPNC | A6PZ97\_SALSA | A6YRT4\_9PEZI | A7KMF0\_9CAEN | A8NI40\_COPC7 | A9LI60\_BIOOC | A9ZSX9\_9BRYO | ABFB\_ASPKW | ADA2\_HUMAN | ADPG2\_ARATH | AGAL\_HUMAN | AGAL\_ORYSJ | AMPS2\_LITPI | AMY1A\_HUMAN | AMY1\_HORVU | AMY1\_ORYSJ | AMYA1\_ASPOR | AMYG\_SACFI | AMY\_ORYLA | ANAG\_HUMAN | ANG1\_BOVIN | ANG2\_MOUSE | ANG3\_MOUSE | ANG4\_MOUSE | ANGI\_MOUSE | AOAH\_MOUSE | AOC1\_HUMAN | AOCX\_BOVIN | APO1\_CYCAE | ASAH1\_BALAS | ASM3A\_HUMAN | ASM3A\_MOUSE | ATLE\_CYCAE | AXE1\_ASPAW | AXE2\_TALPU | AXHA2\_EMENI | B1Q4V2\_HERER | B2L9C1\_TRAHI | B2ZGS7\_9ASPA | B4F320\_LIMPO | B7X9Z0\_COPCI | B7X9Z2\_COPCI | B8MX95\_ASPFN | B9TU22\_GADMO | BGALA\_ASPNC | BGALA\_ASPOR | BGALA\_PENSQ | BGL1\_ASPAC | BGLA\_ASPFU | BGLA\_ASPOR | BGLR\_HUMAN | BLRO\_ALBVE | C3VEV9\_PENCN | C7YSL3\_FUSV7 | CAH1\_CHLRE | CAH6\_HUMAN | CARP1\_CANAL | CARP2\_CANAX | CARP\_RHIPU | CAT3\_NEUCR | CATD\_RAT | CATH\_HUMAN | CATLL\_FASHE | CBHB\_ASPFU | CBHRE\_GEOS1 | CBPA1\_PIG | CBPD\_LOPSP | CBPN\_HUMAN | CDA\_COLLN | CDA\_EMENI | CEL2A\_PIG | CERU\_RAT | CFAD\_MOUSE | CHI1\_COCPS | CHI2\_HORVU | CHI2\_ORYSJ | CHI33\_TRIHA | CHI42\_TRIHA | CHI4\_CRYJA | CHIA\_HUMAN | CHIC\_ARATH | CHIC\_SECCE | CHIL3\_MOUSE | CHIT\_PUNGR | CHLY\_HEVBR | CHYM\_CAMDR | CKX1\_MAIZE | COGS\_HYPLI | CONB\_CANEN | CUCM1\_CUCME | CUTI1\_ASPOR | CUTI1\_COLGL | CUTI1\_FUSVN | CUTI1\_HYPJR | CYND4\_CYNDA | CYP5\_CAEEL | CYSP\_BLOTA | D0QF43\_9HELO | D1M8S7\_HEVBR | D1MPT2\_ROYRE | D6XHE1\_TRYB2 | D7F485\_9APHY | D9MWI4\_9ASPA | DABA\_PSEMU | DDN1\_BOVIN | DEXT\_TALMI | DIR\_GLYEC | DNAS1\_HUMAN | DNSL3\_HUMAN | DOPO\_HUMAN | DPP2\_HUMAN | DYP\_AURAJ | E0A7J0\_YARLL | E0CX04\_MOMBA | E0XN39\_9EURO | E13B\_HORVU | E13C\_MUSAC | E3VTL0\_9ASPA | E5D0X5\_SCHOC | E7FH77\_DANRE | E9G5J5\_DAPPU | ECP\_HUMAN | EGFB2\_MOUSE | EGLB\_ASPNG | ENDO2\_ARATH | ENG1\_RHIMI | ENPP2\_HUMAN | ENPP2\_RAT | ERVB\_TABDI | EST6\_DROME | EXG1\_CANAL | EXG1\_YEAST | F0ZJZ1\_DICPU | F1CYZ0\_TALFU | F2Z7L1\_9ANNE | F6MIW5\_WHEAT | F6N9E7\_9PEZI | FAEA\_ASPNG | FAEB1\_ASPOR | FAEB2\_ASPOR | FUCO\_HUMAN | G0RVK1\_HYPJQ | G0SAW6\_CHATD | G2PZJ2\_MYCTT | G2Q665\_MYCTT | G2QG31\_MYCTT | G2QVH2\_THETT | G2X3Y1\_VERDV | G3I1H5\_CRIGR | G3JPF7\_CORMM | G3YAL0\_ASPNA | G3YFQ1\_ASPNA | G8GLP2\_LENED | G9NTY1\_HYPAI | GALNS\_HUMAN | GANA\_ASPAC | GANA\_EMENI | GANA\_HUMIN | GAOA\_GIBZA | GBA1\_HUMAN | GCE2\_MYCTT | GCE\_CERUI | GCE\_HYPJQ | GGH\_HUMAN | GH7B\_LIMQU | GILT\_MOUSE | GOOX\_SARSR | GOX\_ASPNG | GPX3\_HUMAN | GPX5\_HUMAN | GPX6\_MOUSE | GRAA\_HUMAN | GRAC\_MOUSE | GRAK\_HUMAN | GRASS\_DROME | GUB2\_HORVU | GUN2\_HYPJE | GUN6\_HUMIN | GUN7\_HYPJQ | GUNC\_FUSOX | GUN\_ASPAC | GUN\_CRYAT | GUN\_MYTED | GUX1\_HUMGT | GUX1\_HYPJE | GUX1\_TRIHA | GUX2\_HYPJE | H1AE14\_PHACH | H8ZRU2\_9HELO | HE12\_DANRE | HEXC\_OSTFU | HS3S1\_MOUSE | HYAL1\_HUMAN | I1SB14\_9APHY | I1SB18\_VIPAE | I1VE66\_9APHY | I2FI81\_EISFE | I3RY46\_TRIHA | IDH\_OSTTA | IDUA\_HUMAN | INU2\_ASPFI | INUE\_ASPAW | INV\_SCHOC | IPUA\_ASPNG | J7LCB0\_DEIAC | J9UN47\_GIBZA | K7CID1\_PANTR | K7N5L9\_RAPSA | K7ZUA3\_ASCNO | K9L8F3\_MALCI | KATG2\_MAGO7 | KLK10\_HUMAN | KLK1\_HUMAN | KLK2\_HORSE | KLK2\_HUMAN | KLK7\_HUMAN | KLK7\_MOUSE | KLK8\_MOUSE | L7SVX1\_RHIMI | L8ICE9\_9CETA | LAC1\_MELAO | LAC1\_TRAMX | LAC2\_TRAVE | LALBA\_BOVIN | LALBA\_CAPHI | LALBA\_CAVPO | LALBA\_PAPCY | LAPA\_ASPOR | LCN15\_HUMAN | LGMN\_MOUSE | LICH\_HUMAN | LIG2\_PHACH | LIG4\_PHACH | LIG8\_PHACH | LIP1\_DIURU | LIP2\_DIURU | LIP2\_GEOCN | LIP3\_DIURU | LIPA\_MOEAP | LIPB\_PSEA2 | LIPG\_CANLF | LIPG\_HUMAN | LIPP\_HORSE | LIPR1\_CANLF | LIPR1\_HUMAN | LIPR2\_HUMAN | LIPR2\_RAT | LIP\_THELA | LUCI\_OPLGR | LYG\_STRCA | LYS1\_MUSDO | LYSC1\_ANAPL | LYSC1\_CANLF | LYSC1\_HORSE | LYSC2\_BOVIN | LYSC2\_ONCMY | LYSC\_COTJA | LYSC\_EQUAS | LYSC\_NUMME | LYSC\_OPIHO | LYSC\_PELSI | LYS\_BOMMO | LYS\_RUDPH | M1GME7\_9APHY | M2RAI8\_CERS8 | M9TI89\_RHIPU | MAN12\_PENCI | MAN4\_SOLLC | MANA\_ASPNC | MANA\_CANEN | MANA\_CRYAT | MANA\_HYPJR | MANA\_MYTED | MANA\_PODAN | MANBA\_MOUSE | MCPT2\_RAT | MDL1\_PRUDU | MDLA\_PENCA | MDLA\_PENCY | MEL1\_YEASX | MMP1\_PIG | MNCO\_MICNN | MNLOX\_MAGO7 | NADA\_APLCA | NADA\_ASPFU | NAGAB\_CHICK | NAGAB\_HUMAN | NANL\_MACDE | NCS\_THLFG | NP1\_RHOPR | NP2\_RHOPR | NP4\_RHOPR | NTP1\_TOXGO | NTP2\_TOXGO | NUP1\_PENCI | NUS1\_ASPOR | O00095\_HYPJE | O04358\_IRIHO | O22443\_SOYBN | O44049\_TRYRA | O74705\_ASPNG | O77044\_9NEOP | O81100\_SOLLC | O81226\_CARPA | O81934\_CANEN | O94219\_PLEER | O97389\_HELAM | OFUT1\_CAEEL | OXLA\_BOTAT | OXLA\_CALRH | OXLA\_GLOHA | P79074\_9AGAR | PA1\_VESBA | PA21B\_BOVIN | PA21B\_PIG | PA2A1\_BUNCE | PA2A1\_ECHCA | PA2A1\_NAJAT | PA2A1\_OPHHA | PA2A2\_NAJNA | PA2A2\_OPHHA | PA2A2\_TROCA | PA2A4\_NAJSG | PA2A5\_TRIST | PA2A7\_GLOHA | PA2A\_BOTJR | PA2A\_CROAT | PA2A\_DEIAC | PA2A\_GLOHA | PA2A\_NAJAT | PA2B1\_AGKPI | PA2B2\_BOTJR | PA2B2\_PROFL | PA2B3\_BOTAS | PA2B3\_BUNCE | PA2B5\_BUNCE | PA2B5\_NOTSC | PA2BA\_VIPAA | PA2BB\_GLOHA | PA2BB\_PSEAU | PA2BC\_VIPAA | PA2BD\_CRODU | PA2B\_BUNCE | PA2B\_NOTSC | PA2GA\_HUMAN | PA2GE\_HUMAN | PA2GX\_HUMAN | PA2H1\_AGKCL | PA2H1\_BOTBZ | PA2H1\_BOTJR | PA2H1\_BOTMO | PA2H1\_BOTPI | PA2H2\_BOTAS | PA2H2\_BOTMO | PA2H2\_BOTPI | PA2H2\_CERGO | PA2H3\_BOTPI | PA2HB\_AGKPI | PA2HB\_OXYSC | PA2HH\_TRIST | PA2HS\_ECHCA | PA2H\_BOTPA | PA2H\_DEIAC | PA2H\_PROMB | PA2N\_GLOHA | PA2\_APIME | PAG15\_HUMAN | PCP\_HUMAN | PDE\_NAJAT | PDH1\_LEUMG | PELA\_ASPNG | PELB\_ASPNG | PEM1\_PHACH | PEPA\_ASPPH | PER1A\_ARMRU | PER1\_ARAHY | PER1\_SORBI | PER53\_ARATH | PER59\_ARATH | PERL\_BOVIN | PERL\_BUBBU | PERL\_CAPHI | PER\_ARTRA | PER\_COPCI | PGH2\_HUMAN | PGLR1\_ASPAC | PGLR1\_ASPNG | PGLR\_GIBFU | PGPSA\_DROME | PGRP1\_CAMDR | PHAZ\_TALFU | PHNH\_PENHR | PHYA\_ASPFU | PHYA\_ASPNG | PHYB\_ASPAW | PLA22\_ORYSJ | PLY1\_JUNAS | PME\_DAUCA | PME\_SITOR | POXA\_DICDI | PPA5\_HUMAN | PPA5\_PIG | PPA5\_RAT | PPAF1\_HOLDI | PPAF1\_IPOBA | PPAF\_PHAVU | PPAP\_RAT | PPO8\_ANOGA | PPT1\_BOVIN | PPT1\_HUMAN | PPT2\_HUMAN | PRS57\_HUMAN | PRTN3\_HUMAN | PRXC\_LEPFU | PTGDS\_HUMAN | PTGDS\_MOUSE | Q02321\_PHACH | Q06AK3\_TOXGO | Q07524\_TROMA | Q08J22\_BOMMO | Q0KFV0\_SOLLC | Q12571\_9BASI | Q12715\_HYPJE | Q1K4Q1\_NEUCR | Q1W6B1\_9APHY | Q2QEH4\_SAPOF | Q2TWF5\_ASPOR | Q2U8V9\_ASPOR | Q2Z1W1\_PHACH | Q40069\_HORVU | Q43576\_TOBAC | Q45U61\_ASPNG | Q4AE59\_OSTFU | Q4W6L6\_CYCRE | Q4WP32\_ASPFU | Q50KB2\_PHACH | Q55FE6\_DICDI | Q588B8\_CRYJA | Q5B038\_EMENI | Q5EBY5\_9APHY | Q5WRG2\_RAT | Q60FD2\_9APHY | Q69G21\_TENMO | Q6ED33\_ASPNG | Q6H9H7\_9APHY | Q6NY42\_DANRE | Q6R7Z5\_9TRYP | Q6S5M9\_9ASCO | Q6VAY1\_9PEZI | Q6WER3\_GIBZA | Q6WSR8\_PICAB | Q70C53\_SOLTU | Q70SY0\_HYPJE | Q7LHI2\_PHACH | Q7LIJ0\_PHACH | Q7LST4\_PENEN | Q7RWP2\_NEUCR | Q7X9A9\_CAMSI | Q7XZV5\_NICGU | Q7YXL2\_TENMO | Q86PT9\_RHOPR | Q86RS6\_MANSE | Q874E9\_9TREE | Q8H0C9\_VIGUN | Q8J0K6\_MELAO | Q8J0K8\_MELAO | Q8NJY6\_9HYPO | Q8T0W7\_9NEOP | Q8TFL9\_TALEM | Q8TG26\_THEAU | Q8TG94\_TRAPU | Q8TGI8\_TALEM | Q8VX11\_LUPLU | Q8WZK8\_9APHY | Q92456\_HYPJE | Q92458\_HYPJE | Q93X60\_CICIN | Q94BW3\_CINCA | Q95KP4\_HORSE | Q95V66\_PENVA | Q96TR6\_PYCCO | Q96UT7\_TRAVE | Q96X16\_PICPA | Q9FUH3\_VIGUS | Q9GPG0\_MANSE | Q9HDQ0\_9APHY | Q9LYJ5\_ARATH | Q9M7C7\_CALSE | Q9P8F7\_YARLL | Q9PTT3\_SPAAU | Q9SSV1\_NICGU | Q9STC1\_GRALE | Q9UVQ2\_PYCCI | Q9XEI3\_HORVV | Q9Y780\_COPCI | QPCT1\_DROME | QPCT2\_DROME | QPCT\_IXOSC | QPCT\_MOUSE | RENI\_RAT | RGLA\_ASPAC | RHA1\_ASPAC | RHGA\_ASPAC | RIP0\_DIACA | RIP1\_BRYDI | RIP1\_HORVU | RIP1\_MOMCH | RIP1\_PHYAM | RIP2\_PHYAM | RIP3\_MOMCH | RIPA\_PHYAM | RIPG\_SURMU | RIPL1\_PHYDI | RIPL2\_PHYDI | RIPT\_TRIKI | RNAS1\_BISBI | RNAS4\_HUMAN | RNAS4\_PIG | RNAS6\_HUMAN | RNF1\_GIBFU | RNLE\_SOLLC | RNRH\_RHINI | RNS11\_NICAL | RNS1B\_RAT | RNS3\_PYRPY | RNSL3\_DANRE | RNS\_BOVIN | RNT2\_HUMAN | S6BC01\_PSEA2 | S7Q6I2\_GLOTA | S7ZIW0\_PENO1 | SIA\_ASPFU | SODE\_ONCVO | THCAS\_CANSA | TLP\_PRUAV | TPP1\_HUMAN | TRFL\_BUBBU | TRFL\_HORSE | TRY1\_GADMO | TRY3\_SALSA | TRYB2\_HUMAN | TTHY\_CHICK | TTHY\_MOUSE | TTHY\_RAT | V5NTD\_NAJAT | VM11\_BOTMO | VM12\_CROAD | VM1A3\_DEIAC | VM1BI\_BOTMO | VM1T1\_PROMU | VM1T2\_PROFL | VPL1\_PLEER | VPL2\_PLEER | VSPP\_DEIAC | VSPSX\_GLOSA | W0T408\_KLUMD | W4KMP1\_HETIT | W8P1L2\_TALEM | W8VR85\_TALPI | X0BTD8\_FUSOX | X0M5X0\_FUSOX | XGHA\_ASPTU | XTH34\_POPPZ | XYLA\_ASPNC | XYLO\_MYCTT | XYN1\_HYPJR | XYN2\_HYPJR | XYN3\_ASPKW | XYN3\_HYPJQ | XYNA\_FUSO4 | XYNA\_PENSI | XYNA\_THEAU | XYNA\_THELA | XYNC\_ASPNC | XYND\_EMENI | GO:0102210 | 0.000270929287455974 | 1/3691 | 1/641 | NA | NA | NA | NA | NA | NA | rhamnogalacturonan endolyase activity | RGLA\_ASPAC | GO:0045289 | 0.00162557572473584 | 6/3691 | 1/641 | NA | NA | NA | NA | NA | NA | luciferin monooxygenase activity | LUCI\_OPLGR | GO:0046936 | 0.00135464643727987 | 5/3691 | 1/641 | NA | NA | NA | NA | NA | NA | 2'-deoxyadenosine deaminase activity | ADA2\_HUMAN | GO:0052599 | 0.000270929287455974 | 1/3691 | 1/641 | NA | NA | NA | NA | NA | NA | methylputrescine oxidase activity | AOC1\_HUMAN | GO:0047705 | 0.000270929287455974 | 1/3691 | 1/641 | NA | NA | NA | NA | NA | NA | bilirubin oxidase activity | BLRO\_ALBVE | GO:0016634 | 0.00514765646166351 | 19/3691 | 1/641 | NA | NA | NA | NA | NA | NA | oxidoreductase activity, acting on the CH-CH group of donors, oxygen as acceptor | BLRO\_ALBVE | GO:0046993 | 0.000812787862367922 | 3/3691 | 1/641 | NA | NA | NA | NA | NA | NA | oxidoreductase activity, acting on X-H and Y-H to form an X-Y bond, with oxygen as acceptor | THCAS\_CANSA | GO:0008083 | 0.0010837171498239 | 4/3691 | 1/641 | NA | NA | NA | NA | NA | NA | growth factor activity | ADA2\_HUMAN | GO:0016722 | 0.00568951503657545 | 21/3691 | 1/641 | NA | NA | NA | NA | NA | NA | oxidoreductase activity, acting on metal ions | CERU\_RAT | GO:0102778 | 0.000270929287455974 | 1/3691 | 1/641 | NA | NA | NA | NA | NA | NA | delta9-tetrahydrocannabinolate synthase activity | THCAS\_CANSA | GO:0015036 | 0.00812787862367922 | 30/3691 | 1/641 | NA | NA | NA | NA | NA | NA | disulfide oxidoreductase activity | GILT\_MOUSE | GO:0016747 | 0.0322405852072609 | 119/3691 | 1/641 | NA | NA | NA | NA | NA | NA | acyltransferase activity, transferring groups other than amino-acyl groups | PAG15\_HUMAN | GO:0045480 | 0.000270929287455974 | 1/3691 | 1/641 | NA | NA | NA | NA | NA | NA | galactose oxidase activity | GAOA\_GIBZA | GO:0016879 | 0.0124627472229748 | 46/3691 | 1/641 | NA | NA | NA | NA | NA | NA | ligase activity, forming carbon-nitrogen bonds | A0A183C5H8\_GLOPA | GO:0008240 | 0.000270929287455974 | 1/3691 | 1/641 | NA | NA | NA | NA | NA | NA | tripeptidyl-peptidase activity | TPP1\_HUMAN | GO:0004771 | 0.000812787862367922 | 3/3691 | 1/641 | NA | NA | NA | NA | NA | NA | sterol esterase activity | LICH\_HUMAN | GO:0052600 | 0.000270929287455974 | 1/3691 | 1/641 | NA | NA | NA | NA | NA | NA | propane-1,3-diamine oxidase activity | AOC1\_HUMAN | GO:0035885 | 0.000270929287455974 | 1/3691 | 1/641 | NA | NA | NA | NA | NA | NA | exochitinase activity | CHIC\_ARATH | GO:0004450 | 0.00216743429964779 | 8/3691 | 1/641 | NA | NA | NA | NA | NA | NA | isocitrate dehydrogenase (NADP+) activity | IDH\_OSTTA | GO:0031418 | 0.00270929287455974 | 10/3691 | 1/641 | NA | NA | NA | NA | NA | NA | L-ascorbic acid binding | DOPO\_HUMAN | GO:0061809 | 0.00135464643727987 | 5/3691 | 1/641 | NA | NA | NA | NA | NA | NA | NAD+ nucleotidase, cyclic ADP-ribose generating | NADA\_APLCA | GO:0046589 | 0.000812787862367922 | 3/3691 | 1/641 | NA | NA | NA | NA | NA | NA | ribonuclease T1 activity | RNF1\_GIBFU | GO:0004530 | 0.000270929287455974 | 1/3691 | 1/641 | NA | NA | NA | NA | NA | NA | deoxyribonuclease I activity | DNAS1\_HUMAN | GO:0016874 | 0.0238417772961257 | 88/3691 | 1/641 | NA | NA | NA | NA | NA | NA | ligase activity | A0A183C5H8\_GLOPA | GO:0046593 | 0.000812787862367922 | 3/3691 | 1/641 | NA | NA | NA | NA | NA | NA | mandelonitrile lyase activity | MDL1\_PRUDU | GO:0043295 | 0.00704416147385532 | 26/3691 | 1/641 | NA | NA | NA | NA | NA | NA | glutathione binding | A0A183C5H8\_GLOPA | GO:0001664 | 0.000541858574911948 | 2/3691 | 1/641 | NA | NA | NA | NA | NA | NA | G protein-coupled receptor binding | ADA2\_HUMAN | GO:0004449 | 0.000812787862367922 | 3/3691 | 1/641 | NA | NA | NA | NA | NA | NA | isocitrate dehydrogenase (NAD+) activity | IDH\_OSTTA | GO:0043028 | 0.0010837171498239 | 4/3691 | 1/641 | NA | NA | NA | NA | NA | NA | cysteine-type endopeptidase regulator activity involved in apoptotic process | CATH\_HUMAN | GO:0047376 | 0.000541858574911948 | 2/3691 | 1/641 | NA | NA | NA | NA | NA | NA | all-trans-retinyl-palmitate hydrolase, all-trans-retinol forming activity | LIPP\_HORSE | GO:0061634 | 0.000270929287455974 | 1/3691 | 1/641 | NA | NA | NA | NA | NA | NA | alpha-D-xyloside xylohydrolase | XYLA\_ASPNC | GO:0017040 | 0.000812787862367922 | 3/3691 | 1/641 | NA | NA | NA | NA | NA | NA | N-acylsphingosine amidohydrolase activity | ASAH1\_BALAS | GO:0004571 | 0.000541858574911948 | 2/3691 | 1/641 | NA | NA | NA | NA | NA | NA | mannosyl-oligosaccharide 1,2-alpha-mannosidase activity | MAN12\_PENCI | GO:0050839 | 0.00433486859929558 | 16/3691 | 1/641 | NA | NA | NA | NA | NA | NA | cell adhesion molecule binding | EXG1\_CANAL | GO:0016671 | 0.00325115144947169 | 12/3691 | 1/641 | NA | NA | NA | NA | NA | NA | oxidoreductase activity, acting on a sulfur group of donors, disulfide as acceptor | GILT\_MOUSE | GO:0031685 | 0.000270929287455974 | 1/3691 | 1/641 | NA | NA | NA | NA | NA | NA | adenosine receptor binding | ADA2\_HUMAN | GO:0046992 | 0.000812787862367922 | 3/3691 | 1/641 | NA | NA | NA | NA | NA | NA | oxidoreductase activity, acting on X-H and Y-H to form an X-Y bond | THCAS\_CANSA | GO:0051675 | 0.000270929287455974 | 1/3691 | 1/641 | NA | NA | NA | NA | NA | NA | isopullulanase activity | IPUA\_ASPNG | GO:0102121 | 0.000812787862367922 | 3/3691 | 1/641 | NA | NA | NA | NA | NA | NA | ceramidase activity | ASAH1\_BALAS | GO:0052594 | 0.000812787862367922 | 3/3691 | 1/641 | NA | NA | NA | NA | NA | NA | aminoacetone:oxygen oxidoreductase(deaminating) activity | AOCX\_BOVIN | GO:0060090 | 0.00270929287455974 | 10/3691 | 1/641 | NA | NA | NA | NA | NA | NA | molecular adaptor activity | PPAP\_RAT | GO:0050135 | 0.00135464643727987 | 5/3691 | 1/641 | NA | NA | NA | NA | NA | NA | NAD(P)+ nucleosidase activity | NADA\_APLCA | GO:0019239 | 0.00839880791113519 | 31/3691 | 1/641 | NA | NA | NA | NA | NA | NA | deaminase activity | ADA2\_HUMAN | GO:0047499 | 0.0010837171498239 | 4/3691 | 1/641 | NA | NA | NA | NA | NA | NA | calcium-independent phospholipase A2 activity | PAG15\_HUMAN | GO:0016936 | 0.000541858574911948 | 2/3691 | 1/641 | NA | NA | NA | NA | NA | NA | galactoside binding | AGAL\_HUMAN | GO:0016893 | 0.00162557572473584 | 6/3691 | 1/641 | NA | NA | NA | NA | NA | NA | endonuclease activity, active with either ribo- or deoxyribonucleic acids and producing 5'-phosphomonoesters | DNAS1\_HUMAN | GO:0016842 | 0.00460579788675156 | 17/3691 | 1/641 | NA | NA | NA | NA | NA | NA | amidine-lyase activity | LYS\_RUDPH | GO:0046922 | 0.000541858574911948 | 2/3691 | 1/641 | NA | NA | NA | NA | NA | NA | peptide-O-fucosyltransferase activity | OFUT1\_CAEEL | GO:0016411 | 0.000541858574911948 | 2/3691 | 1/641 | NA | NA | NA | NA | NA | NA | acylglycerol O-acyltransferase activity | PAG15\_HUMAN | GO:0052642 | 0.0010837171498239 | 4/3691 | 1/641 | NA | NA | NA | NA | NA | NA | lysophosphatidic acid phosphatase activity | PPAP\_RAT | GO:0043262 | 0.000541858574911948 | 2/3691 | 1/641 | NA | NA | NA | NA | NA | NA | ADP phosphatase activity | PDE\_NAJAT | GO:0003940 | 0.000270929287455974 | 1/3691 | 1/641 | NA | NA | NA | NA | NA | NA | L-iduronidase activity | IDUA\_HUMAN | GO:0017064 | 0.000812787862367922 | 3/3691 | 1/641 | NA | NA | NA | NA | NA | NA | fatty acid amide hydrolase activity | ASAH1\_BALAS | GO:0050162 | 0.000541858574911948 | 2/3691 | 1/641 | NA | NA | NA | NA | NA | NA | oxalate oxidase activity | A0A097P6E1\_9FABA | GO:0008430 | 0.000812787862367922 | 3/3691 | 1/641 | NA | NA | NA | NA | NA | NA | selenium binding | GPX3\_HUMAN | GO:0016018 | 0.00162557572473584 | 6/3691 | 1/641 | NA | NA | NA | NA | NA | NA | cyclosporin A binding | CYP5\_CAEEL | GO:0043765 | 0.000270929287455974 | 1/3691 | 1/641 | NA | NA | NA | NA | NA | NA | T/G mismatch-specific endonuclease activity | ENDO2\_ARATH | GO:0016715 | 0.000812787862367922 | 3/3691 | 1/641 | NA | NA | NA | NA | NA | NA | oxidoreductase activity, acting on paired donors, with incorporation or reduction of molecular oxygen, reduced ascorbate as one donor, and incorporation of one atom of oxygen | DOPO\_HUMAN | GO:0070573 | 0.00162557572473584 | 6/3691 | 1/641 | NA | NA | NA | NA | NA | NA | metallodipeptidase activity | A0NFU8\_ANOGA | GO:0008146 | 0.00541858574911948 | 20/3691 | 1/641 | NA | NA | NA | NA | NA | NA | sulfotransferase activity | HS3S1\_MOUSE | GO:0102402 | 0.000270929287455974 | 1/3691 | 1/641 | NA | NA | NA | NA | NA | NA | 2-phenylethyl 6-O-beta-D-xylopyranosyl-beta-D-glucopyranoside glucosidase (Yabukita) activity | Q7X9A9\_CAMSI | GO:0016773 | 0.0384719588187483 | 142/3691 | 1/641 | NA | NA | NA | NA | NA | NA | phosphotransferase activity, alcohol group as acceptor | Q06AK3\_TOXGO | GO:0004415 | 0.000541858574911948 | 2/3691 | 1/641 | NA | NA | NA | NA | NA | NA | hyalurononglucosaminidase activity | HYAL1\_HUMAN | GO:0051670 | 0.000270929287455974 | 1/3691 | 1/641 | NA | NA | NA | NA | NA | NA | inulinase activity | INU2\_ASPFI | GO:0004363 | 0.00135464643727987 | 5/3691 | 1/641 | NA | NA | NA | NA | NA | NA | glutathione synthase activity | A0A183C5H8\_GLOPA | GO:0048030 | 0.000270929287455974 | 1/3691 | 1/641 | NA | NA | NA | NA | NA | NA | disaccharide binding | MANA\_HYPJR | GO:0016724 | 0.00487672717420753 | 18/3691 | 1/641 | NA | NA | NA | NA | NA | NA | oxidoreductase activity, acting on metal ions, oxygen as acceptor | CERU\_RAT | GO:0004000 | 0.00243836358710377 | 9/3691 | 1/641 | NA | NA | NA | NA | NA | NA | adenosine deaminase activity | ADA2\_HUMAN | GO:0052593 | 0.0010837171498239 | 4/3691 | 1/641 | NA | NA | NA | NA | NA | NA | tryptamine:oxygen oxidoreductase (deaminating) activity | AOCX\_BOVIN | GO:0016505 | 0.000270929287455974 | 1/3691 | 1/641 | NA | NA | NA | NA | NA | NA | peptidase activator activity involved in apoptotic process | CATH\_HUMAN | GO:0032393 | 0.000270929287455974 | 1/3691 | 1/641 | NA | NA | NA | NA | NA | NA | MHC class I receptor activity | CATH\_HUMAN | GO:0033904 | 0.000270929287455974 | 1/3691 | 1/641 | NA | NA | NA | NA | NA | NA | dextranase activity | DEXT\_TALMI | GO:0016840 | 0.010024383635871 | 37/3691 | 1/641 | NA | NA | NA | NA | NA | NA | carbon-nitrogen lyase activity | LYS\_RUDPH | GO:0016782 | 0.00975345434841506 | 36/3691 | 1/641 | NA | NA | NA | NA | NA | NA | transferase activity, transferring sulphur-containing groups | HS3S1\_MOUSE | GO:0019863 | 0.00162557572473584 | 6/3691 | 1/641 | NA | NA | NA | NA | NA | NA | IgE binding | TLP\_PRUAV | GO:0008656 | 0.000270929287455974 | 1/3691 | 1/641 | NA | NA | NA | NA | NA | NA | cysteine-type endopeptidase activator activity involved in apoptotic process | CATH\_HUMAN | GO:0004666 | 0.000541858574911948 | 2/3691 | 1/641 | NA | NA | NA | NA | NA | NA | prostaglandin-endoperoxide synthase activity | PGH2\_HUMAN | GO:0031404 | 0.000812787862367922 | 3/3691 | 1/641 | NA | NA | NA | NA | NA | NA | chloride ion binding | AMY1A\_HUMAN | GO:1990136 | 0.0010837171498239 | 4/3691 | 1/641 | NA | NA | NA | NA | NA | NA | linoleate 9S-lipoxygenase activity | MNLOX\_MAGO7 | GO:0004888 | 0.0010837171498239 | 4/3691 | 1/641 | NA | NA | NA | NA | NA | NA | transmembrane signaling receptor activity | CATH\_HUMAN | GO:0016504 | 0.000270929287455974 | 1/3691 | 1/641 | NA | NA | NA | NA | NA | NA | peptidase activator activity | CATH\_HUMAN | GO:0050501 | 0.000270929287455974 | 1/3691 | 1/641 | NA | NA | NA | NA | NA | NA | hyaluronan synthase activity | HYAL1\_HUMAN | GO:0034483 | 0.000541858574911948 | 2/3691 | 1/641 | NA | NA | NA | NA | NA | NA | heparan sulfate sulfotransferase activity | HS3S1\_MOUSE | GO:0016832 | 0.010024383635871 | 37/3691 | 1/641 | NA | NA | NA | NA | NA | NA | aldehyde-lyase activity | MDL1\_PRUDU | GO:0005159 | 0.000270929287455974 | 1/3691 | 1/641 | NA | NA | NA | NA | NA | NA | insulin-like growth factor receptor binding | RENI\_RAT | GO:0050253 | 0.0010837171498239 | 4/3691 | 1/641 | NA | NA | NA | NA | NA | NA | retinyl-palmitate esterase activity | LIPP\_HORSE | GO:0016691 | 0.000541858574911948 | 2/3691 | 1/641 | NA | NA | NA | NA | NA | NA | chloride peroxidase activity | PRXC\_LEPFU | GO:0038187 | 0.000270929287455974 | 1/3691 | 1/641 | NA | NA | NA | NA | NA | NA | pattern recognition receptor activity | PGPSA\_DROME | GO:0033945 | 0.000270929287455974 | 1/3691 | 1/641 | NA | NA | NA | NA | NA | NA | oligoxyloglucan reducing-end-specific cellobiohydrolase activity | CBHRE\_GEOS1 | GO:0004500 | 0.000270929287455974 | 1/3691 | 1/641 | NA | NA | NA | NA | NA | NA | dopamine beta-monooxygenase activity | DOPO\_HUMAN | GO:1904091 | 0.00135464643727987 | 5/3691 | 1/641 | NA | NA | NA | NA | NA | NA | non-ribosomal peptide synthetase activity | A0A183C5H8\_GLOPA | GO:0016903 | 0.0260092115957735 | 96/3691 | 1/641 | NA | NA | NA | NA | NA | NA | oxidoreductase activity, acting on the aldehyde or oxo group of donors | A0A097P6E1\_9FABA | GO:0015459 | 0.000270929287455974 | 1/3691 | 1/641 | NA | NA | NA | NA | NA | NA | potassium channel regulator activity | PA2A\_NAJAT | GO:0004867 | 0.000270929287455974 | 1/3691 | 1/641 | NA | NA | NA | NA | NA | NA | serine-type endopeptidase inhibitor activity | A0A097P6E1\_9FABA | GO:0032500 | 0.000270929287455974 | 1/3691 | 1/641 | NA | NA | NA | NA | NA | NA | muramyl dipeptide binding | PGPSA\_DROME | GO:0016881 | 0.00460579788675156 | 17/3691 | 1/641 | NA | NA | NA | NA | NA | NA | acid-amino acid ligase activity | A0A183C5H8\_GLOPA | GO:0008484 | 0.0010837171498239 | 4/3691 | 1/641 | NA | NA | NA | NA | NA | NA | sulfuric ester hydrolase activity | GALNS\_HUMAN | GO:0052757 | 0.000270929287455974 | 1/3691 | 1/641 | NA | NA | NA | NA | NA | NA | chondroitin hydrolase activity | HYAL1\_HUMAN | GO:0004561 | 0.000270929287455974 | 1/3691 | 1/641 | NA | NA | NA | NA | NA | NA | alpha-N-acetylglucosaminidase activity | ANAG\_HUMAN | GO:0032397 | 0.000270929287455974 | 1/3691 | 1/641 | NA | NA | NA | NA | NA | NA | activating MHC class I receptor activity | CATH\_HUMAN | GO:0140859 | 0.000270929287455974 | 1/3691 | 1/641 | NA | NA | NA | NA | NA | NA | pterocarpan synthase activity | DIR\_GLYEC | GO:0016616 | 0.0682741804389054 | 252/3691 | 1/641 | NA | NA | NA | NA | NA | NA | oxidoreductase activity, acting on the CH-OH group of donors, NAD or NADP as acceptor | IDH\_OSTTA | GO:0043890 | 0.000270929287455974 | 1/3691 | 1/641 | NA | NA | NA | NA | NA | NA | N-acetylgalactosamine-6-sulfatase activity | GALNS\_HUMAN | GO:0004185 | 0.000270929287455974 | 1/3691 | 1/641 | NA | NA | NA | NA | NA | NA | serine-type carboxypeptidase activity | PCP\_HUMAN | GO:0030108 | 0.000270929287455974 | 1/3691 | 1/641 | NA | NA | NA | NA | NA | NA | HLA-A specific activating MHC class I receptor activity | CATH\_HUMAN | GO:0016703 | 0.00189650501219182 | 7/3691 | 1/641 | NA | NA | NA | NA | NA | NA | oxidoreductase activity, acting on single donors with incorporation of molecular oxygen, incorporation of one atom of oxygen (internal monooxygenases or internal mixed function oxidases) | LUCI\_OPLGR | GO:1990238 | 0.000270929287455974 | 1/3691 | 1/641 | NA | NA | NA | NA | NA | NA | double-stranded DNA endonuclease activity | ENDO2\_ARATH | GO:0050584 | 0.000541858574911948 | 2/3691 | 1/641 | NA | NA | NA | NA | NA | NA | linoleate 11-lipoxygenase activity | MNLOX\_MAGO7 | GO:0061810 | 0.000541858574911948 | 2/3691 | 1/641 | NA | NA | NA | NA | NA | NA | NAD glycohydrolase activity | NADA\_ASPFU | GO:0106415 | 0.000270929287455974 | 1/3691 | 1/641 | NA | NA | NA | NA | NA | NA | muramoyltetrapeptide carboxypeptidase activity | PGPSA\_DROME | GO:0019865 | 0.00162557572473584 | 6/3691 | 1/641 | NA | NA | NA | NA | NA | NA | immunoglobulin binding | TLP\_PRUAV | GO:0008467 | 0.000541858574911948 | 2/3691 | 1/641 | NA | NA | NA | NA | NA | NA | [heparan sulfate]-glucosamine 3-sulfotransferase 1 activity | HS3S1\_MOUSE | GO:0052595 | 0.00162557572473584 | 6/3691 | 1/641 | NA | NA | NA | NA | NA | NA | aliphatic amine oxidase activity | AOCX\_BOVIN | GO:0050528 | 0.000270929287455974 | 1/3691 | 1/641 | NA | NA | NA | NA | NA | NA | acyloxyacyl hydrolase activity | AOAH\_MOUSE | GO:0052861 | 0.000270929287455974 | 1/3691 | 1/641 | NA | NA | NA | NA | NA | NA | glucan endo-1,3-beta-glucanase activity, C-3 substituted reducing group | ENG1\_RHIMI | GO:0008374 | 0.00514765646166351 | 19/3691 | 1/641 | NA | NA | NA | NA | NA | NA | O-acyltransferase activity | PAG15\_HUMAN | GO:0004322 | 0.00487672717420753 | 18/3691 | 1/641 | NA | NA | NA | NA | NA | NA | ferroxidase activity | CERU\_RAT | GO:0033931 | 0.000270929287455974 | 1/3691 | 1/641 | NA | NA | NA | NA | NA | NA | endogalactosaminidase activity | HYAL1\_HUMAN | GO:0102404 | 0.000270929287455974 | 1/3691 | 1/641 | NA | NA | NA | NA | NA | NA | linalyl 6-O-alpha-L-arabinopyranosyl-beta-D-glucopyranoside glucosidase (Yabukita) activity | Q7X9A9\_CAMSI | GO:0046982 | 0.00135464643727987 | 5/3691 | 1/641 | NA | NA | NA | NA | NA | NA | protein heterodimerization activity | TTHY\_CHICK | GO:0019139 | 0.00162557572473584 | 6/3691 | 1/641 | NA | NA | NA | NA | NA | NA | cytokinin dehydrogenase activity | CKX1\_MAIZE | GO:0004447 | 0.000270929287455974 | 1/3691 | 1/641 | NA | NA | NA | NA | NA | NA | iodide peroxidase activity | POXA\_DICDI | GO:0016623 | 0.00216743429964779 | 8/3691 | 1/641 | NA | NA | NA | NA | NA | NA | oxidoreductase activity, acting on the aldehyde or oxo group of donors, oxygen as acceptor | A0A097P6E1\_9FABA | GO:0017110 | 0.00135464643727987 | 5/3691 | 1/641 | NA | NA | NA | NA | NA | NA | nucleoside diphosphate phosphatase activity | PDE\_NAJAT | GO:0033995 | 0.000270929287455974 | 1/3691 | 1/641 | NA | NA | NA | NA | NA | NA | anhydrosialidase activity | NANL\_MACDE | GO:0016645 | 0.0146301815226226 | 54/3691 | 1/641 | NA | NA | NA | NA | NA | NA | oxidoreductase activity, acting on the CH-NH group of donors | CKX1\_MAIZE | GO:0004722 | 0.00568951503657545 | 21/3691 | 1/641 | NA | NA | NA | NA | NA | NA | protein serine/threonine phosphatase activity | A4GX63\_TOXGO | GO:0033718 | 0.000270929287455974 | 1/3691 | 1/641 | NA | NA | NA | NA | NA | NA | pyranose dehydrogenase (acceptor) activity | PDH1\_LEUMG | GO:0050535 | 0.000270929287455974 | 1/3691 | 1/641 | NA | NA | NA | NA | NA | NA | beta-primeverosidase activity | Q7X9A9\_CAMSI | GO:0005537 | 0.000270929287455974 | 1/3691 | 1/641 | NA | NA | NA | NA | NA | NA | mannose binding | MANBA\_MOUSE | GO:0050474 | 0.000270929287455974 | 1/3691 | 1/641 | NA | NA | NA | NA | NA | NA | (S)-norcoclaurine synthase activity | NCS\_THLFG | GO:0016667 | 0.0162557572473584 | 60/3691 | 1/641 | NA | NA | NA | NA | NA | NA | oxidoreductase activity, acting on a sulfur group of donors | GILT\_MOUSE | GO:0016627 | 0.0230289894337578 | 85/3691 | 1/641 | NA | NA | NA | NA | NA | NA | oxidoreductase activity, acting on the CH-CH group of donors | BLRO\_ALBVE | GO:0004348 | 0.000541858574911948 | 2/3691 | 1/641 | NA | NA | NA | NA | NA | NA | glucosylceramidase activity | GBA1\_HUMAN | GO:0003943 | 0.000541858574911948 | 2/3691 | 1/641 | NA | NA | NA | NA | NA | NA | N-acetylgalactosamine-4-sulfatase activity | GALNS\_HUMAN | GO:0004065 | 0.000541858574911948 | 2/3691 | 1/641 | NA | NA | NA | NA | NA | NA | arylsulfatase activity | GALNS\_HUMAN | GO:0017041 | 0.000270929287455974 | 1/3691 | 1/641 | NA | NA | NA | NA | NA | NA | obsolete galactosylgalactosylglucosylceramidase activity | AGAL\_HUMAN | GO:0008241 | 0.000270929287455974 | 1/3691 | 1/641 | NA | NA | NA | NA | NA | NA | peptidyl-dipeptidase activity | A0NFU8\_ANOGA | GO:0016814 | 0.0073150907613113 | 27/3691 | 1/641 | NA | NA | NA | NA | NA | NA | hydrolase activity, acting on carbon-nitrogen (but not peptide) bonds, in cyclic amidines | ADA2\_HUMAN | GO:0016888 | 0.000812787862367922 | 3/3691 | 1/641 | NA | NA | NA | NA | NA | NA | endodeoxyribonuclease activity, producing 5'-phosphomonoesters | DNAS1\_HUMAN | GO:0016805 | 0.00243836358710377 | 9/3691 | 1/641 | NA | NA | NA | NA | NA | NA | dipeptidase activity | A0NFU8\_ANOGA | GO:0016615 | 0.00596044432403143 | 22/3691 | 1/641 | NA | NA | NA | NA | NA | NA | malate dehydrogenase activity | LIPG\_HUMAN | GO:0043394 | 0.000270929287455974 | 1/3691 | 1/641 | NA | NA | NA | NA | NA | NA | proteoglycan binding | ADA2\_HUMAN | GO:0004566 | 0.000270929287455974 | 1/3691 | 1/641 | NA | NA | NA | NA | NA | NA | beta-glucuronidase activity | BGLR\_HUMAN | GO:0034338 | 0.000812787862367922 | 3/3691 | 1/641 | NA | NA | NA | NA | NA | NA | short-chain carboxylesterase activity | EST6\_DROME | GO:0106435 | 0.00270929287455974 | 10/3691 | 1/641 | NA | NA | NA | NA | NA | NA | carboxylesterase activity | EST6\_DROME | GO:0051087 | 0.00352208073692766 | 13/3691 | 1/641 | NA | NA | NA | NA | NA | NA | protein-folding chaperone binding | CERU\_RAT | GO:0050295 | 0.000541858574911948 | 2/3691 | 1/641 | NA | NA | NA | NA | NA | NA | steryl-beta-glucosidase activity | GBA1\_HUMAN | GO:0015924 | 0.000812787862367922 | 3/3691 | 1/641 | NA | NA | NA | NA | NA | NA | mannosyl-oligosaccharide mannosidase activity | MAN12\_PENCI | GO:0042131 | 0.000270929287455974 | 1/3691 | 1/641 | NA | NA | NA | NA | NA | NA | thiamine phosphate phosphatase activity | PPAP\_RAT | GO:0008417 | 0.0010837171498239 | 4/3691 | 1/641 | NA | NA | NA | NA | NA | NA | fucosyltransferase activity | OFUT1\_CAEEL | GO:0052598 | 0.000270929287455974 | 1/3691 | 1/641 | NA | NA | NA | NA | NA | NA | histamine oxidase activity | AOC1\_HUMAN | GO:0052596 | 0.00135464643727987 | 5/3691 | 1/641 | NA | NA | NA | NA | NA | NA | phenethylamine:oxygen oxidoreductase (deaminating) activity | AOCX\_BOVIN | GO:0019842 | 0.041994039555676 | 155/3691 | 1/641 | NA | NA | NA | NA | NA | NA | vitamin binding | DOPO\_HUMAN | GO:0102545 | 0.000541858574911948 | 2/3691 | 1/641 | NA | NA | NA | NA | NA | NA | phosphatidyl phospholipase B activity | PAG15\_HUMAN | GO:0004336 | 0.000812787862367922 | 3/3691 | 1/641 | NA | NA | NA | NA | NA | NA | galactosylceramidase activity | GBA1\_HUMAN | GO:2001070 | 0.0010837171498239 | 4/3691 | 1/641 | NA | NA | NA | NA | NA | NA | starch binding | S7ZIW0\_PENO1 | GO:0005124 | 0.000270929287455974 | 1/3691 | 1/641 | NA | NA | NA | NA | NA | NA | scavenger receptor binding | GBA1\_HUMAN | GO:0052862 | 0.000270929287455974 | 1/3691 | 1/641 | NA | NA | NA | NA | NA | NA | glucan endo-1,4-beta-glucanase activity, C-3 substituted reducing group | ENG1\_RHIMI | GO:0004672 | 0.0146301815226226 | 54/3691 | 1/641 | NA | NA | NA | NA | NA | NA | protein kinase activity | Q06AK3\_TOXGO | GO:0061929 | 0.000812787862367922 | 3/3691 | 1/641 | NA | NA | NA | NA | NA | NA | gamma-glutamylaminecyclotransferase activity | LYS\_RUDPH | GO:0016019 | 0.000270929287455974 | 1/3691 | 1/641 | NA | NA | NA | NA | NA | NA | peptidoglycan immune receptor activity | PGPSA\_DROME | GO:0140375 | 0.000270929287455974 | 1/3691 | 1/641 | NA | NA | NA | NA | NA | NA | immune receptor activity | CATH\_HUMAN | GO:0047682 | 0.000270929287455974 | 1/3691 | 1/641 | NA | NA | NA | NA | NA | NA | aryl-alcohol oxidase activity | O94219\_PLEER | GO:0008047 | 0.00541858574911948 | 20/3691 | 1/641 | NA | NA | NA | NA | NA | NA | enzyme activator activity | CATH\_HUMAN | GO:0003847 | 0.000541858574911948 | 2/3691 | 1/641 | NA | NA | NA | NA | NA | NA | 1-alkyl-2-acetylglycerophosphocholine esterase activity | PA2GX\_HUMAN | GO:0016301 | 0.0658358168518017 | 243/3691 | 1/641 | NA | NA | NA | NA | NA | NA | kinase activity | Q06AK3\_TOXGO | GO:0001530 | 0.000270929287455974 | 1/3691 | 1/641 | NA | NA | NA | NA | NA | NA | lipopolysaccharide binding | ECP\_HUMAN | GO:0052736 | 0.000270929287455974 | 1/3691 | 1/641 | NA | NA | NA | NA | NA | NA | beta-glucanase activity | ENG1\_RHIMI | GO:0033265 | 0.000812787862367922 | 3/3691 | 1/641 | NA | NA | NA | NA | NA | NA | choline binding | PPAP\_RAT | GO:0004448 | 0.00243836358710377 | 9/3691 | 1/641 | NA | NA | NA | NA | NA | NA | isocitrate dehydrogenase [NAD(P)+] activity | IDH\_OSTTA | GO:0033756 | 0.000270929287455974 | 1/3691 | 1/641 | NA | NA | NA | NA | NA | NA | Oplophorus-luciferin 2-monooxygenase activity | LUCI\_OPLGR | GO:0004097 | 0.0010837171498239 | 4/3691 | 1/641 | NA | NA | NA | NA | NA | NA | catechol oxidase activity | PPO8\_ANOGA | GO:0016830 | 0.0338661609319967 | 125/3691 | 1/641 | NA | NA | NA | NA | NA | NA | carbon-carbon lyase activity | MDL1\_PRUDU | GO:0033943 | 0.000270929287455974 | 1/3691 | 1/641 | NA | NA | NA | NA | NA | NA | galactan 1,3-beta-galactosidase activity | Q50KB2\_PHACH | GO:0016772 | 0.0780276347873205 | 288/3691 | 1/641 | NA | NA | NA | NA | NA | NA | transferase activity, transferring phosphorus-containing groups | Q06AK3\_TOXGO | GO:0047457 | 0.000270929287455974 | 1/3691 | 1/641 | NA | NA | NA | NA | NA | NA | exo-(1,4)-alpha-D-glucan lyase activity | Q9STC1\_GRALE | GO:0004725 | 0.00650230289894338 | 24/3691 | 1/641 | NA | NA | NA | NA | NA | NA | protein tyrosine phosphatase activity | PPAP\_RAT |

Total number of genes: 3691   
Total number of Study genes: 641   
Total number of Study gene GMRG terms (pop non-singletons): 466 (401)   
FDR Threshold *P*-values: [10% = 0.0476], [5% = 0.0189], [1% = 0.00278], [0.5% = 0.0013]   
Genes with GMRG information: 641   
Genes with no GMRG information:   

These are:
