# Supplementary material for: Cellular location shapes quaternary structure of enzymes
Source: Nat Commun. 2024 Oct 1;15:8505. doi: 10.1038/s41467-024-52662-2 (PMC11445431; doi:10.1038/s41467-024-52662-2)
Supplement: Supplementary file 5 — Supplementary Data 2 [file 41467_2024_52662_MOESM5_ESM.zip › GO/Prok_F/output.F.txt.html]

 GeneMerge Output - output.F.txt

### GeneMerge v1.4

### Castillo-Davis, C.I. 2015. GeneMerge v1.4 - post-genomic data analysis

Output File Name: output.F.txt   
Gene Association File: terms.txt   
Description File: descriptions.txt   
Population File: prot\_all.txt   
Study File: prot\_test.txt  
Custom FDR: 0.5%  

|  |  |  |  |  |  |  |  |  |  |  |  |
| --- | --- | --- | --- | --- | --- | --- | --- | --- | --- | --- | --- |
| **GMRG Term** | **Pop Frequency** | **Pop Fraction** | **Study Fraction** | ***P*-value** | **Bon. Corr. *P*-value** | **10% FDR** | **5% FDR** | **1% FDR** | **0.5% FDR** | **Description** | **Contributing genes** |
| GO:0016787 | 0.274441687344913 | 2212/8060 | 172/235 | 1.51629688165421e-49 | 2.92645298159262e-47 | T | T | T | T | hydrolase activity | A0A067XG64\_PARTM | A0A0B7LH44\_STREE | A0A0H2UQE4\_STRPN | A0A0H2WX20\_STAAC | A0A0M3KKW7\_STRPY | A0A182DW00\_STREE | A0A1L1QK12\_STRSQ | A0A1L1QK13\_9ACTN | A0A1L1QK16\_9ACTN | A0A243G6Q6\_BACTU | A0A378K9X8\_LEGPN | A0A4P8ESF9\_BACSP | A0A4V8H037\_9RHOB | A0A6L7H1P2\_BACAN | A0Q436\_FRATN | A0R5R2\_MYCS2 | A3DJ82\_ACET2 | A5N1B8\_CLOK5 | A8FDC4\_BACP2 | AMT6\_BACS7 | AMYB\_BACCE | AMYM\_GEOSE | AMY\_BACAM | AMY\_GEOSE | B4EPS2\_BURCJ | B4SL31\_STRM5 | B9MMA5\_CALBD | BLAC\_BACSU | BLAC\_STRAL | BSAP\_BACSU | BXX\_CLOBO | C3X8T5\_OXAFO | C3X9F9\_OXAFO | C3X9N1\_OXAFO | C7ED31\_ACET1 | CBPM\_STRAL | CDGT\_BACS0 | CDGT\_GEOSE | CDTB\_ECOLX | CHIS\_BACSU | CHIS\_STRSN | CRAX\_BACCE | D0VV09\_BACCE | D0VXY8\_STAPS | D2ARB3\_STRRD | D5DH82\_PRIM3 | D8H130\_BACAI | D9TA79\_MICAI | DACC\_BACSU | DAC\_STRSR | DRNE\_VIBCH | E4Q2A4\_CALOW | EBA1\_ELIME | EBA3\_ELIME | ENG1\_ACET2 | ESTA\_BACSU | ESTA\_PSEAE | ESTA\_STRSC | ETA\_STAAU | ETB\_STAAU | FLS\_FERPE | G0L2Y1\_ZOBGA | G2NRC4\_STREK | G7CF24\_MYCT3 | G8LZE0\_ACECE | GSEA\_STAES | GUB\_BACLI | GUB\_BACSU | GUNA\_ACET2 | GUNC\_ACETH | GUNF\_RUMCH | GUNG\_RUMCH | GUNS\_ACETH | GUN\_ECOLI | H0B8D4\_9ACTN | HCPB\_HELPY | HCPC\_HELPY | I3P686\_BACLI | IABF\_STRAW | K0IUV6\_9ENTR | L0EGW1\_THECK | L0RUV7\_STAAU | LIP\_BACSP | LIP\_PSEAE | LYTM\_STAA8 | NANB\_STRPN | NANH\_VIBCH | NUCA\_SERMA | O06496\_CLOPF | O30700\_9BACI | O31243\_RHIRD | O52754\_RHOMR | O66015\_GEOSE | O68424\_BACFG | O69771\_9PSED | O82839\_BACSP | O86049\_BACFG | P84147\_STRSM | P94458\_BACLI | PEPX\_LACHE | PGLR2\_PECPM | PHL2\_BACCE | PHLC1\_CLOPE | PHLC\_BACCE | PHLC\_CLOP1 | PHLC\_CLOPF | PHLC\_STAAU | PHYC\_BACIU | PHYT\_BACSD | PLC\_BACCE | PLC\_BACTU | PLC\_LISMO | PLC\_STAAE | PLD\_STRAT | PMEA\_DICD3 | PRZN\_SERME | Q09LY9\_GEOSE | Q17TM8\_ALKHA | Q21KE5\_SACD2 | Q2XSK9\_VIBCL | Q2XSL7\_ALISL | Q52NH3\_BACAN | Q54276\_SERMA | Q59962\_9ACTN | Q5DZ44\_ALIF1 | Q5I4I3\_9BACI | Q5MJ80\_SERPR | Q5ZWG6\_LEGPH | Q65GB9\_BACLD | Q65JI7\_BACLD | Q65JI8\_BACLD | Q6MNP0\_BDEBA | Q6RSN8\_ACETH | Q79DR3\_ECOLX | Q7LYT7\_PYRWO | Q7SID8\_BACIU | Q7X4S4\_BACLI | Q81DA0\_BACCR | Q82L26\_STRAW | Q88JL2\_PSEPK | Q8L1V2\_GEOTH | Q8ZRI7\_SALTY | Q93A71\_GEOSE | Q93I48\_9BACI | Q9AJF8\_ACETH | Q9AJS0\_ALIAC | Q9EYQ2\_9FIRM | Q9I229\_PSEAE | Q9JZ43\_NEIMB | Q9KWY6\_GEOSE | Q9RC94\_9BACI | Q9REI6\_ARTSP | Q9RHW0\_BACSP | Q9XB24\_KLEPN | RHA78\_STRAW | RIPB\_MYCTU | RNS3\_KITAU | SFAS2\_STRFR | SPLA\_STAA8 | SPLB\_STAA8 | SPLC\_STAA8 | SPLD\_STAA8 | SPLE\_STAA8 | SSL1\_STAA8 | SUBD\_BACLI | V5IRV7\_THETH | W5JXE0\_BACSP | W8FKE7\_BACPU | XYN1\_GEOSE | XYNA\_HALH5 | XYNC\_BACSU | YKFC\_BACC1 |
 GO:0004553 | 0.0560794044665012 | 452/8060 | 78/235 | 7.02125639316332e-41 | 1.35510248388052e-38 | T | T | T | T | hydrolase activity, hydrolyzing O-glycosyl compounds | A0A067XG64\_PARTM | A0A0B7LH44\_STREE | A0A0H2UQE4\_STRPN | A0A182DW00\_STREE | A0A1L1QK12\_STRSQ | A0A1L1QK13\_9ACTN | A0A1L1QK16\_9ACTN | A0A243G6Q6\_BACTU | A0A4P8ESF9\_BACSP | A0A6L7H1P2\_BACAN | A3DJ82\_ACET2 | A8FDC4\_BACP2 | AMT6\_BACS7 | AMYB\_BACCE | AMYM\_GEOSE | AMY\_BACAM | AMY\_GEOSE | B9MMA5\_CALBD | C7ED31\_ACET1 | CDGT\_BACS0 | CDGT\_GEOSE | CHIS\_BACSU | CHIS\_STRSN | D0VV09\_BACCE | E4Q2A4\_CALOW | EBA1\_ELIME | EBA3\_ELIME | ENG1\_ACET2 | G0L2Y1\_ZOBGA | G2NRC4\_STREK | G8LZE0\_ACECE | GUB\_BACLI | GUB\_BACSU | GUNA\_ACET2 | GUNC\_ACETH | GUNF\_RUMCH | GUNG\_RUMCH | GUNS\_ACETH | GUN\_ECOLI | I3P686\_BACLI | IABF\_STRAW | K0IUV6\_9ENTR | L0EGW1\_THECK | NANB\_STRPN | NANH\_VIBCH | O06496\_CLOPF | O30700\_9BACI | O52754\_RHOMR | O82839\_BACSP | PGLR2\_PECPM | Q09LY9\_GEOSE | Q17TM8\_ALKHA | Q21KE5\_SACD2 | Q54276\_SERMA | Q59962\_9ACTN | Q5DZ44\_ALIF1 | Q65GB9\_BACLD | Q65JI7\_BACLD | Q65JI8\_BACLD | Q6RSN8\_ACETH | Q7LYT7\_PYRWO | Q7SID8\_BACIU | Q7X4S4\_BACLI | Q82L26\_STRAW | Q88JL2\_PSEPK | Q93I48\_9BACI | Q9AJF8\_ACETH | Q9AJS0\_ALIAC | Q9EYQ2\_9FIRM | Q9KWY6\_GEOSE | Q9RC94\_9BACI | Q9REI6\_ARTSP | Q9RHW0\_BACSP | RHA78\_STRAW | W5JXE0\_BACSP | XYN1\_GEOSE | XYNA\_HALH5 | XYNC\_BACSU | GO:0016798 | 0.0653846153846154 | 527/8060 | 78/235 | 6.83608431482954e-36 | 1.3193642727621e-33 | T | T | T | T | hydrolase activity, acting on glycosyl bonds | A0A067XG64\_PARTM | A0A0B7LH44\_STREE | A0A0H2UQE4\_STRPN | A0A182DW00\_STREE | A0A1L1QK12\_STRSQ | A0A1L1QK13\_9ACTN | A0A1L1QK16\_9ACTN | A0A243G6Q6\_BACTU | A0A4P8ESF9\_BACSP | A0A6L7H1P2\_BACAN | A3DJ82\_ACET2 | A8FDC4\_BACP2 | AMT6\_BACS7 | AMYB\_BACCE | AMYM\_GEOSE | AMY\_BACAM | AMY\_GEOSE | B9MMA5\_CALBD | C7ED31\_ACET1 | CDGT\_BACS0 | CDGT\_GEOSE | CHIS\_BACSU | CHIS\_STRSN | D0VV09\_BACCE | E4Q2A4\_CALOW | EBA1\_ELIME | EBA3\_ELIME | ENG1\_ACET2 | G0L2Y1\_ZOBGA | G2NRC4\_STREK | G8LZE0\_ACECE | GUB\_BACLI | GUB\_BACSU | GUNA\_ACET2 | GUNC\_ACETH | GUNF\_RUMCH | GUNG\_RUMCH | GUNS\_ACETH | GUN\_ECOLI | I3P686\_BACLI | IABF\_STRAW | K0IUV6\_9ENTR | L0EGW1\_THECK | NANB\_STRPN | NANH\_VIBCH | O06496\_CLOPF | O30700\_9BACI | O52754\_RHOMR | O82839\_BACSP | PGLR2\_PECPM | Q09LY9\_GEOSE | Q17TM8\_ALKHA | Q21KE5\_SACD2 | Q54276\_SERMA | Q59962\_9ACTN | Q5DZ44\_ALIF1 | Q65GB9\_BACLD | Q65JI7\_BACLD | Q65JI8\_BACLD | Q6RSN8\_ACETH | Q7LYT7\_PYRWO | Q7SID8\_BACIU | Q7X4S4\_BACLI | Q82L26\_STRAW | Q88JL2\_PSEPK | Q93I48\_9BACI | Q9AJF8\_ACETH | Q9AJS0\_ALIAC | Q9EYQ2\_9FIRM | Q9KWY6\_GEOSE | Q9RC94\_9BACI | Q9REI6\_ARTSP | Q9RHW0\_BACSP | RHA78\_STRAW | W5JXE0\_BACSP | XYN1\_GEOSE | XYNA\_HALH5 | XYNC\_BACSU | GO:0008810 | 0.00471464019851117 | 38/8060 | 17/235 | 7.46239322333496e-17 | 1.44024189210365e-14 | T | T | T | T | cellulase activity | A8FDC4\_BACP2 | C7ED31\_ACET1 | GUNA\_ACET2 | GUNC\_ACETH | GUNF\_RUMCH | GUNG\_RUMCH | GUNS\_ACETH | GUN\_ECOLI | K0IUV6\_9ENTR | Q5DZ44\_ALIF1 | Q65JI7\_BACLD | Q65JI8\_BACLD | Q6RSN8\_ACETH | Q7X4S4\_BACLI | Q88JL2\_PSEPK | Q9AJS0\_ALIAC | Q9EYQ2\_9FIRM | GO:0016298 | 0.00446650124069479 | 36/8060 | 16/235 | 7.13931677484851e-16 | 1.37788813754576e-13 | T | T | T | T | lipase activity | ESTA\_BACSU | ESTA\_PSEAE | LIP\_BACSP | LIP\_PSEAE | O66015\_GEOSE | P84147\_STRSM | PHL2\_BACCE | PHLC1\_CLOPE | PHLC\_BACCE | PHLC\_CLOP1 | PHLC\_CLOPF | PHLC\_STAAU | PLD\_STRAT | Q5I4I3\_9BACI | Q8L1V2\_GEOTH | Q93A71\_GEOSE | GO:0004175 | 0.00843672456575682 | 68/8060 | 20/235 | 2.08711073369238e-15 | 4.02812371602629e-13 | T | T | T | T | endopeptidase activity | A0A0H2WX20\_STAAC | BXX\_CLOBO | D0VXY8\_STAPS | ETA\_STAAU | ETB\_STAAU | FLS\_FERPE | GSEA\_STAES | L0RUV7\_STAAU | LYTM\_STAA8 | O68424\_BACFG | O69771\_9PSED | PRZN\_SERME | Q5MJ80\_SERPR | SFAS2\_STRFR | SPLA\_STAA8 | SPLB\_STAA8 | SPLC\_STAA8 | SPLE\_STAA8 | SUBD\_BACLI | V5IRV7\_THETH | GO:0008233 | 0.0306451612903226 | 247/8060 | 35/235 | 3.08228109713875e-15 | 5.9488025174778e-13 | T | T | T | T | peptidase activity | A0A0H2WX20\_STAAC | A0A0M3KKW7\_STRPY | A0A4V8H037\_9RHOB | BSAP\_BACSU | BXX\_CLOBO | CBPM\_STRAL | CRAX\_BACCE | D0VXY8\_STAPS | D8H130\_BACAI | DACC\_BACSU | DAC\_STRSR | ETA\_STAAU | ETB\_STAAU | FLS\_FERPE | GSEA\_STAES | L0RUV7\_STAAU | LYTM\_STAA8 | O68424\_BACFG | O69771\_9PSED | O86049\_BACFG | PEPX\_LACHE | PRZN\_SERME | Q52NH3\_BACAN | Q5MJ80\_SERPR | RIPB\_MYCTU | SFAS2\_STRFR | SPLA\_STAA8 | SPLB\_STAA8 | SPLC\_STAA8 | SPLD\_STAA8 | SPLE\_STAA8 | SSL1\_STAA8 | SUBD\_BACLI | V5IRV7\_THETH | YKFC\_BACC1 | GO:0097599 | 0.0054590570719603 | 44/8060 | 16/235 | 3.30726684902284e-14 | 6.38302501861408e-12 | T | T | T | T | xylanase activity | A0A067XG64\_PARTM | A0A4P8ESF9\_BACSP | B9MMA5\_CALBD | E4Q2A4\_CALOW | G8LZE0\_ACECE | L0EGW1\_THECK | O30700\_9BACI | Q09LY9\_GEOSE | Q17TM8\_ALKHA | Q59962\_9ACTN | Q7SID8\_BACIU | Q9RC94\_9BACI | W5JXE0\_BACSP | XYN1\_GEOSE | XYNA\_HALH5 | XYNC\_BACSU | GO:0031176 | 0.00397022332506203 | 32/8060 | 14/235 | 6.45783133936456e-14 | 1.24636144849736e-11 | T | T | T | T | endo-1,4-beta-xylanase activity | A0A4P8ESF9\_BACSP | B9MMA5\_CALBD | E4Q2A4\_CALOW | G8LZE0\_ACECE | L0EGW1\_THECK | O30700\_9BACI | Q09LY9\_GEOSE | Q17TM8\_ALKHA | Q59962\_9ACTN | Q7SID8\_BACIU | Q9RC94\_9BACI | W5JXE0\_BACSP | XYN1\_GEOSE | XYNA\_HALH5 | GO:0090729 | 0.00334987593052109 | 27/8060 | 13/235 | 1.10531588959137e-13 | 2.13325966691135e-11 | T | T | T | T | toxin activity | A0A0M3KKW7\_STRPY | BXX\_CLOBO | CDTB\_ECOLX | CHXA\_VIBCL | CRAX\_BACCE | ENTC2\_STAAU | ETA\_STAAU | ETB\_STAAU | PHLC1\_CLOPE | PHLC\_CLOP1 | PHLC\_CLOPF | PHLC\_STAAU | Q9RQQ5\_STRPY | GO:0030570 | 0.00186104218362283 | 15/8060 | 10/235 | 9.70790789462867e-13 | 1.87362622366333e-10 | T | T | T | T | pectate lyase activity | A0A160EBC2\_ACHDE | A1TSQ3\_ACIAC | D0VP31\_9BACI | PLYL\_DICD3 | PLY\_BACSU | PTLY\_THEMA | Q47465\_PECCA | Q8P6Z9\_XANCP | Q9AJM4\_9BACI | Q9RHW0\_BACSP | GO:0140096 | 0.045409429280397 | 366/8060 | 38/235 | 4.39785891823247e-12 | 8.48786771218868e-10 | T | T | T | T | catalytic activity, acting on a protein | A0A0H2WX20\_STAAC | A0A0M3KKW7\_STRPY | A0A378K9X8\_LEGPN | A0A4V8H037\_9RHOB | BSAP\_BACSU | BXX\_CLOBO | CBPM\_STRAL | CRAX\_BACCE | D0VXY8\_STAPS | D8H130\_BACAI | DACC\_BACSU | DAC\_STRSR | ETA\_STAAU | ETB\_STAAU | FLS\_FERPE | GSEA\_STAES | L0RUV7\_STAAU | LYTM\_STAA8 | O68424\_BACFG | O69771\_9PSED | O86049\_BACFG | PEPX\_LACHE | PRZN\_SERME | Q52NH3\_BACAN | Q5MJ80\_SERPR | Q6MNP0\_BDEBA | Q9ADS9\_STAAU | RIPB\_MYCTU | SFAS2\_STRFR | SPLA\_STAA8 | SPLB\_STAA8 | SPLC\_STAA8 | SPLD\_STAA8 | SPLE\_STAA8 | SSL1\_STAA8 | SUBD\_BACLI | V5IRV7\_THETH | YKFC\_BACC1 | GO:0016837 | 0.00558312655086849 | 45/8060 | 14/235 | 1.63096052052448e-11 | 3.14775380461224e-09 | T | T | T | T | carbon-oxygen lyase activity, acting on polysaccharides | A0A160EBC2\_ACHDE | A1TSQ3\_ACIAC | ALYA\_KLEPN | D0VP31\_9BACI | G0L2Y1\_ZOBGA | HYSA\_STRA3 | PLYL\_DICD3 | PLY\_BACSU | PTLY\_THEMA | Q47465\_PECCA | Q8P6Z9\_XANCP | Q9AJM4\_9BACI | Q9RHW0\_BACSP | XANLY\_BACGL | GO:0005509 | 0.00657568238213399 | 53/8060 | 14/235 | 1.90892069637304e-10 | 3.68421694399996e-08 | T | T | T | T | calcium ion binding | AMT6\_BACS7 | AMY\_BACAM | AMY\_GEOSE | ENG1\_ACET2 | FLS\_FERPE | I3P686\_BACLI | O69771\_9PSED | O82839\_BACSP | PRZN\_SERME | Q7LYT7\_PYRWO | Q93I48\_9BACI | Q9KWY6\_GEOSE | Q9RHW0\_BACSP | XANLY\_BACGL | GO:0004252 | 0.00558312655086849 | 45/8060 | 13/235 | 2.52230449114538e-10 | 4.86804766791059e-08 | T | T | T | T | serine-type endopeptidase activity | A0A0H2WX20\_STAAC | D0VXY8\_STAPS | ETA\_STAAU | ETB\_STAAU | FLS\_FERPE | GSEA\_STAES | L0RUV7\_STAAU | SFAS2\_STRFR | SPLA\_STAA8 | SPLB\_STAA8 | SPLC\_STAA8 | SPLE\_STAA8 | SUBD\_BACLI | GO:0050053 | 0.0011166253101737 | 9/8060 | 7/235 | 5.61831785467373e-10 | 1.08433534595203e-07 | T | T | T | T | levansucrase activity | A0A0M3KKU6\_ERWAE | B2IF78\_BEII9 | B2VCC3\_ERWT9 | D5DC07\_PRIM3 | D8J9C2\_HALJB | SACB\_BACSU | SACB\_GLUDI | GO:0016160 | 0.00334987593052109 | 27/8060 | 10/235 | 2.00328685495797e-09 | 3.86634363006888e-07 | T | T | T | T | amylase activity | AMT6\_BACS7 | AMYB\_BACCE | AMYM\_GEOSE | AMY\_BACAM | AMY\_GEOSE | CDGT\_BACS0 | CDGT\_GEOSE | I3P686\_BACLI | Q7LYT7\_PYRWO | Q9KWY6\_GEOSE | GO:0008236 | 0.0121588089330025 | 98/8060 | 17/235 | 2.49526787549748e-09 | 4.81586699971013e-07 | T | T | T | T | serine-type peptidase activity | A0A0H2WX20\_STAAC | D0VXY8\_STAPS | DACC\_BACSU | DAC\_STRSR | ETA\_STAAU | ETB\_STAAU | FLS\_FERPE | GSEA\_STAES | L0RUV7\_STAAU | PEPX\_LACHE | SFAS2\_STRFR | SPLA\_STAA8 | SPLB\_STAA8 | SPLC\_STAA8 | SPLD\_STAA8 | SPLE\_STAA8 | SUBD\_BACLI | GO:0017171 | 0.0121588089330025 | 98/8060 | 17/235 | 2.49526787549748e-09 | 4.81586699971013e-07 | T | T | T | T | serine hydrolase activity | A0A0H2WX20\_STAAC | D0VXY8\_STAPS | DACC\_BACSU | DAC\_STRSR | ETA\_STAAU | ETB\_STAAU | FLS\_FERPE | GSEA\_STAES | L0RUV7\_STAAU | PEPX\_LACHE | SFAS2\_STRFR | SPLA\_STAA8 | SPLB\_STAA8 | SPLC\_STAA8 | SPLD\_STAA8 | SPLE\_STAA8 | SUBD\_BACLI | GO:0004620 | 0.00186104218362283 | 15/8060 | 8/235 | 2.50329620826061e-09 | 4.83136168194298e-07 | T | T | T | T | phospholipase activity | P84147\_STRSM | PHL2\_BACCE | PHLC1\_CLOPE | PHLC\_BACCE | PHLC\_CLOP1 | PHLC\_CLOPF | PHLC\_STAAU | PLD\_STRAT | GO:0008081 | 0.00558312655086849 | 45/8060 | 12/235 | 3.50007727281443e-09 | 6.75514913653184e-07 | T | T | T | T | phosphoric diester hydrolase activity | P84147\_STRSM | PHL2\_BACCE | PHLC1\_CLOPE | PHLC\_BACCE | PHLC\_CLOP1 | PHLC\_CLOPF | PHLC\_STAAU | PLC\_BACCE | PLC\_BACTU | PLC\_LISMO | PLC\_STAAE | PLD\_STRAT | GO:0004550 | 0.0032258064516129 | 26/8060 | 9/235 | 2.65028771592968e-08 | 5.11505529174429e-06 | T | T | T | T | nucleoside diphosphate kinase activity | D0CAF1\_ACIB2 | NDK\_ACIBS | NDK\_AQUAE | NDK\_BURTA | NDK\_CAMJE | NDK\_HELPG | NDK\_MYXXA | NDK\_NEIG2 | V5VIC4\_ACIBA | GO:0004556 | 0.0032258064516129 | 26/8060 | 9/235 | 2.65028771592968e-08 | 5.11505529174429e-06 | T | T | T | T | alpha-amylase activity | AMT6\_BACS7 | AMYM\_GEOSE | AMY\_BACAM | AMY\_GEOSE | CDGT\_BACS0 | CDGT\_GEOSE | I3P686\_BACLI | Q7LYT7\_PYRWO | Q9KWY6\_GEOSE | GO:0030247 | 0.00173697270471464 | 14/8060 | 7/235 | 4.72546215965465e-08 | 9.12014196813347e-06 | T | T | T | T | polysaccharide binding | A0A243G6Q6\_BACTU | AMYB\_BACCE | AMYM\_GEOSE | CDGT\_BACS0 | CDGT\_GEOSE | GUNG\_RUMCH | Q9AJF8\_ACETH | GO:0034480 | 0.000744416873449132 | 6/8060 | 5/235 | 1.18384737489299e-07 | 2.28482543354347e-05 | T | T | T | T | phosphatidylcholine phospholipase C activity | PHLC1\_CLOPE | PHLC\_BACCE | PHLC\_CLOP1 | PHLC\_CLOPF | PHLC\_STAAU | GO:0004222 | 0.00223325062034739 | 18/8060 | 7/235 | 3.96448528477095e-07 | 7.65145659960794e-05 | T | T | T | T | metalloendopeptidase activity | BXX\_CLOBO | LYTM\_STAA8 | O68424\_BACFG | O69771\_9PSED | PRZN\_SERME | Q5MJ80\_SERPR | V5IRV7\_THETH | GO:0004629 | 0.00086848635235732 | 7/8060 | 5/235 | 4.04493132243197e-07 | 7.80671745229371e-05 | T | T | T | T | phospholipase C activity | PHLC1\_CLOPE | PHLC\_BACCE | PHLC\_CLOP1 | PHLC\_CLOPF | PHLC\_STAAU | GO:0016997 | 0.00086848635235732 | 7/8060 | 5/235 | 4.04493132243197e-07 | 7.80671745229371e-05 | T | T | T | T | alpha-sialidase activity | A0A0B7LH44\_STREE | A0A0H2UQE4\_STRPN | A0A182DW00\_STREE | NANB\_STRPN | NANH\_VIBCH | GO:0052795 | 0.00086848635235732 | 7/8060 | 5/235 | 4.04493132243197e-07 | 7.80671745229371e-05 | T | T | T | T | exo-alpha-(2->6)-sialidase activity | A0A0B7LH44\_STREE | A0A0H2UQE4\_STRPN | A0A182DW00\_STREE | NANB\_STRPN | NANH\_VIBCH | GO:0052796 | 0.00086848635235732 | 7/8060 | 5/235 | 4.04493132243197e-07 | 7.80671745229371e-05 | T | T | T | T | exo-alpha-(2->8)-sialidase activity | A0A0B7LH44\_STREE | A0A0H2UQE4\_STRPN | A0A182DW00\_STREE | NANB\_STRPN | NANH\_VIBCH | GO:0004308 | 0.00086848635235732 | 7/8060 | 5/235 | 4.04493132243197e-07 | 7.80671745229371e-05 | T | T | T | T | exo-alpha-sialidase activity | A0A0B7LH44\_STREE | A0A0H2UQE4\_STRPN | A0A182DW00\_STREE | NANB\_STRPN | NANH\_VIBCH | GO:0052794 | 0.00086848635235732 | 7/8060 | 5/235 | 4.04493132243197e-07 | 7.80671745229371e-05 | T | T | T | T | exo-alpha-(2->3)-sialidase activity | A0A0B7LH44\_STREE | A0A0H2UQE4\_STRPN | A0A182DW00\_STREE | NANB\_STRPN | NANH\_VIBCH | GO:0004806 | 0.00235732009925558 | 19/8060 | 7/235 | 6.12211535880472e-07 | 0.000118156826424931 | T | T | T | T | triglyceride lipase activity | ESTA\_BACSU | LIP\_BACSP | LIP\_PSEAE | O66015\_GEOSE | Q5I4I3\_9BACI | Q8L1V2\_GEOTH | Q93A71\_GEOSE | GO:0004436 | 0.000496277915632754 | 4/8060 | 4/235 | 7.0487287987927e-07 | 0.000136040465816699 | T | T | T | T | phosphatidylinositol diacylglycerol-lyase activity | PLC\_BACCE | PLC\_BACTU | PLC\_LISMO | PLC\_STAAE | GO:0004784 | 0.00620347394540943 | 50/8060 | 10/235 | 1.35214649660265e-06 | 0.000260964273844311 | T | T | T | T | superoxide dismutase activity | Q186I6\_CLOD6 | Q5M4Z1\_STRT2 | SODF\_METTH | SODM1\_STAA8 | SODM2\_BACAN | SODM2\_STAA8 | SODM\_BACSU | SODM\_GEOSE | SODM\_PROFR | SODM\_STRMU | GO:0008237 | 0.011166253101737 | 90/8060 | 13/235 | 1.76984819279311e-06 | 0.00034158070120907 | T | T | T | T | metallopeptidase activity | BSAP\_BACSU | BXX\_CLOBO | CBPM\_STRAL | CRAX\_BACCE | D8H130\_BACAI | LYTM\_STAA8 | O68424\_BACFG | O69771\_9PSED | O86049\_BACFG | PRZN\_SERME | Q52NH3\_BACAN | Q5MJ80\_SERPR | V5IRV7\_THETH | GO:0016788 | 0.0681141439205955 | 549/8060 | 36/235 | 3.14797914390884e-06 | 0.000607559974774407 | T | T | T | T | hydrolase activity, acting on ester bonds | A0A378K9X8\_LEGPN | A0Q436\_FRATN | B4SL31\_STRM5 | CDTB\_ECOLX | DRNE\_VIBCH | ESTA\_BACSU | ESTA\_PSEAE | ESTA\_STRSC | LIP\_BACSP | LIP\_PSEAE | NUCA\_SERMA | O66015\_GEOSE | P84147\_STRSM | PHL2\_BACCE | PHLC1\_CLOPE | PHLC\_BACCE | PHLC\_CLOP1 | PHLC\_CLOPF | PHLC\_STAAU | PHYC\_BACIU | PHYT\_BACSD | PLC\_BACCE | PLC\_BACTU | PLC\_LISMO | PLC\_STAAE | PLD\_STRAT | PMEA\_DICD3 | Q2XSK9\_VIBCL | Q2XSL7\_ALISL | Q5I4I3\_9BACI | Q5ZWG6\_LEGPH | Q6MNP0\_BDEBA | Q8L1V2\_GEOTH | Q93A71\_GEOSE | Q9JZ43\_NEIMB | RNS3\_KITAU | GO:0016721 | 0.00694789081885856 | 56/8060 | 10/235 | 4.01960284540676e-06 | 0.000775783349163504 | T | T | T | T | oxidoreductase activity, acting on superoxide radicals as acceptor | Q186I6\_CLOD6 | Q5M4Z1\_STRT2 | SODF\_METTH | SODM1\_STAA8 | SODM2\_BACAN | SODM2\_STAA8 | SODM\_BACSU | SODM\_GEOSE | SODM\_PROFR | SODM\_STRMU | GO:0030246 | 0.0127791563275434 | 103/8060 | 13/235 | 8.23629849839111e-06 | 0.00158960561018948 | T | T | T | T | carbohydrate binding | A0A243G6Q6\_BACTU | AMYB\_BACCE | AMYM\_GEOSE | CDGT\_BACS0 | CDGT\_GEOSE | GUNG\_RUMCH | HYSA\_STRA3 | P84141\_PAEAU | Q54276\_SERMA | Q9AJF8\_ACETH | Q9RC94\_9BACI | Q9REI6\_ARTSP | XANLY\_BACGL | GO:0008061 | 0.00235732009925558 | 19/8060 | 6/235 | 1.13711538133311e-05 | 0.0021946326859729 | T | T | T | T | chitin binding | A0A243G6Q6\_BACTU | A0A6L7H1P2\_BACAN | D0VV09\_BACCE | LCHMO\_ENTFA | Q54276\_SERMA | Q9REI6\_ARTSP | GO:0008800 | 0.0227047146401985 | 183/8060 | 17/235 | 2.26569725734198e-05 | 0.00437279570667002 | T | T | T | T | beta-lactamase activity | A0A4V8H037\_9RHOB | A5N1B8\_CLOK5 | B4EPS2\_BURCJ | BLAC\_BACSU | BLAC\_STRAL | C3X8T5\_OXAFO | C3X9F9\_OXAFO | C3X9N1\_OXAFO | D2ARB3\_STRRD | D5DH82\_PRIM3 | D9TA79\_MICAI | HCPB\_HELPY | HCPC\_HELPY | P94458\_BACLI | Q79DR3\_ECOLX | Q81DA0\_BACCR | Q9XB24\_KLEPN | GO:0042578 | 0.0255583126550868 | 206/8060 | 18/235 | 2.97966032386178e-05 | 0.00575074442505323 | T | T | T | T | phosphoric ester hydrolase activity | A0A378K9X8\_LEGPN | B4SL31\_STRM5 | P84147\_STRSM | PHL2\_BACCE | PHLC1\_CLOPE | PHLC\_BACCE | PHLC\_CLOP1 | PHLC\_CLOPF | PHLC\_STAAU | PHYC\_BACIU | PHYT\_BACSD | PLC\_BACCE | PLC\_BACTU | PLC\_LISMO | PLC\_STAAE | PLD\_STRAT | Q5ZWG6\_LEGPH | Q6MNP0\_BDEBA | GO:2001070 | 0.00124069478908189 | 10/8060 | 4/235 | 0.000128823181375421 | 0.0248628740054563 | T | T | T | T | starch binding | AMYB\_BACCE | AMYM\_GEOSE | CDGT\_BACS0 | CDGT\_GEOSE | GO:0016812 | 0.0272952853598015 | 220/8060 | 17/235 | 0.000226366244928713 | 0.0436886852712416 | T | T | T | T | hydrolase activity, acting on carbon-nitrogen (but not peptide) bonds, in cyclic amides | A0A4V8H037\_9RHOB | A5N1B8\_CLOK5 | B4EPS2\_BURCJ | BLAC\_BACSU | BLAC\_STRAL | C3X8T5\_OXAFO | C3X9F9\_OXAFO | C3X9N1\_OXAFO | D2ARB3\_STRRD | D5DH82\_PRIM3 | D9TA79\_MICAI | HCPB\_HELPY | HCPC\_HELPY | P94458\_BACLI | Q79DR3\_ECOLX | Q81DA0\_BACCR | Q9XB24\_KLEPN | GO:0016758 | 0.0122828784119107 | 99/8060 | 10/235 | 0.000587559394369577 | 0.113398963113328 | T | T | T | T | hexosyltransferase activity | A0A0M3KKU6\_ERWAE | AMYS\_NEIPO | B2IF78\_BEII9 | B2VCC3\_ERWT9 | CDGT\_BACS0 | CDGT\_GEOSE | D5DC07\_PRIM3 | D8J9C2\_HALJB | SACB\_BACSU | SACB\_GLUDI | GO:0016977 | 0.00086848635235732 | 7/8060 | 3/235 | 0.000785264819663794 | 0.151556110195112 | T | T | T | T | chitosanase activity | CHIS\_BACSU | CHIS\_STRSN | G2NRC4\_STREK | GO:0004630 | 0.000248138957816377 | 2/8060 | 2/235 | 0.000846579060077079 | 0.163389758594876 | T | T | T | T | phospholipase D activity | P84147\_STRSM | PLD\_STRAT | GO:0030248 | 0.000248138957816377 | 2/8060 | 2/235 | 0.000846579060077079 | 0.163389758594876 | T | T | T | T | cellulose binding | GUNG\_RUMCH | Q9AJF8\_ACETH | GO:0050348 | 0.000248138957816377 | 2/8060 | 2/235 | 0.000846579060077079 | 0.163389758594876 | T | T | T | T | trehalose O-mycolyltransferase activity | A85B\_MYCTU | A85C\_MYCTU | GO:0004650 | 0.000248138957816377 | 2/8060 | 2/235 | 0.000846579060077079 | 0.163389758594876 | T | T | T | T | polygalacturonase activity | PGLR2\_PECPM | Q9RHW0\_BACSP | GO:0070290 | 0.000248138957816377 | 2/8060 | 2/235 | 0.000846579060077079 | 0.163389758594876 | T | T | T | T | N-acylphosphatidylethanolamine-specific phospholipase D activity | P84147\_STRSM | PLD\_STRAT | GO:0016209 | 0.0228287841191067 | 184/8060 | 14/235 | 0.000941063028125171 | 0.181625164428158 | T | T | T | T | antioxidant activity | D1A807\_THECD | EFEN\_BACSU | Q186I6\_CLOD6 | Q5M4Z1\_STRT2 | Q9RKQ2\_STRCO | Q9ZBW9\_STRCO | SODF\_METTH | SODM1\_STAA8 | SODM2\_BACAN | SODM2\_STAA8 | SODM\_BACSU | SODM\_GEOSE | SODM\_PROFR | SODM\_STRMU | GO:0019205 | 0.0114143920595534 | 92/8060 | 9/235 | 0.00138271008551396 | 0.266863046504194 | T | T | T | T | nucleobase-containing compound kinase activity | D0CAF1\_ACIB2 | NDK\_ACIBS | NDK\_AQUAE | NDK\_BURTA | NDK\_CAMJE | NDK\_HELPG | NDK\_MYXXA | NDK\_NEIG2 | V5VIC4\_ACIBA | GO:0035375 | 0.0011166253101737 | 9/8060 | 3/235 | 0.00180460998023712 | 0.348289726185764 | T | T | T | T | zymogen binding | A85B\_MYCTU | A85C\_MYCTU | MPT51\_MYCTU | GO:0004767 | 0.000372208436724566 | 3/8060 | 2/235 | 0.00249077889753077 | 0.480720327223439 | T | T | T | F | sphingomyelin phosphodiesterase activity | PHL2\_BACCE | PHLC\_STAAU | GO:0042972 | 0.000372208436724566 | 3/8060 | 2/235 | 0.00249077889753077 | 0.480720327223439 | T | T | T | F | licheninase activity | GUB\_BACLI | GUB\_BACSU | GO:0016776 | 0.0127791563275434 | 103/8060 | 9/235 | 0.00303017164853757 | 0.584823128167751 | T | T | T | F | phosphotransferase activity, phosphate group as acceptor | D0CAF1\_ACIB2 | NDK\_ACIBS | NDK\_AQUAE | NDK\_BURTA | NDK\_CAMJE | NDK\_HELPG | NDK\_MYXXA | NDK\_NEIG2 | V5VIC4\_ACIBA | GO:0004567 | 0.00136476426799007 | 11/8060 | 3/235 | 0.00339467441090547 | 0.655172161304755 | T | T | T | F | beta-mannosidase activity | A0A1L1QK12\_STRSQ | A0A1L1QK13\_9ACTN | A0A1L1QK16\_9ACTN | GO:0016985 | 0.00136476426799007 | 11/8060 | 3/235 | 0.00339467441090547 | 0.655172161304755 | T | T | T | F | mannan endo-1,4-beta-mannosidase activity | A0A1L1QK12\_STRSQ | A0A1L1QK13\_9ACTN | A0A1L1QK16\_9ACTN | GO:0052689 | 0.0181141439205955 | 146/8060 | 11/235 | 0.00352125445126535 | 0.679602109094212 | T | T | T | F | carboxylic ester hydrolase activity | ESTA\_BACSU | ESTA\_PSEAE | ESTA\_STRSC | LIP\_BACSP | LIP\_PSEAE | O66015\_GEOSE | PMEA\_DICD3 | Q5I4I3\_9BACI | Q8L1V2\_GEOTH | Q93A71\_GEOSE | Q9JZ43\_NEIMB | GO:0001968 | 0.000496277915632754 | 4/8060 | 2/235 | 0.00488575584830055 | 0.942950878722005 | T | T | F | F | fibronectin binding | A85B\_MYCTU | MPT51\_MYCTU | GO:0004144 | 0.000496277915632754 | 4/8060 | 2/235 | 0.00488575584830055 | 0.942950878722005 | T | T | F | F | diacylglycerol O-acyltransferase activity | A85B\_MYCTU | A85C\_MYCTU | GO:0004325 | 0.00161290322580645 | 13/8060 | 3/235 | 0.00563566372805825 | 1 | T | T | F | F | ferrochelatase activity | EFEN\_BACSU | Q9RKQ2\_STRCO | Q9ZBW9\_STRCO | GO:0016158 | 0.000620347394540943 | 5/8060 | 2/235 | 0.00798670002007734 | 1 | T | T | F | F | 3-phytase activity | PHYC\_BACIU | PHYT\_BACSD | GO:0016411 | 0.000620347394540943 | 5/8060 | 2/235 | 0.00798670002007734 | 1 | T | T | F | F | acylglycerol O-acyltransferase activity | A85B\_MYCTU | A85C\_MYCTU | GO:0008234 | 0.00186104218362283 | 15/8060 | 3/235 | 0.00858836778236447 | 1 | T | T | F | F | cysteine-type peptidase activity | A0A0M3KKW7\_STRPY | RIPB\_MYCTU | YKFC\_BACC1 | GO:0015923 | 0.00186104218362283 | 15/8060 | 3/235 | 0.00858836778236447 | 1 | T | T | F | F | mannosidase activity | A0A1L1QK12\_STRSQ | A0A1L1QK13\_9ACTN | A0A1L1QK16\_9ACTN | GO:0043895 | 0.000744416873449132 | 6/8060 | 2/235 | 0.0117507573310081 | 1 | T | T | F | F | cyclomaltodextrin glucanotransferase activity | CDGT\_BACS0 | CDGT\_GEOSE | GO:0045135 | 0.000744416873449132 | 6/8060 | 2/235 | 0.0117507573310081 | 1 | T | T | F | F | poly(beta-D-mannuronate) lyase activity | ALYA\_KLEPN | G0L2Y1\_ZOBGA | GO:0033925 | 0.000744416873449132 | 6/8060 | 2/235 | 0.0117507573310081 | 1 | T | T | F | F | mannosyl-glycoprotein endo-beta-N-acetylglucosaminidase activity | EBA1\_ELIME | EBA3\_ELIME | GO:0019899 | 0.00210918114143921 | 17/8060 | 3/235 | 0.0122965786976489 | 1 | T | T | F | F | enzyme binding | A85B\_MYCTU | A85C\_MYCTU | MPT51\_MYCTU | GO:0042301 | 0.00086848635235732 | 7/8060 | 2/235 | 0.0161369543355479 | 1 | T | T | F | F | phosphate ion binding | C3K8K1\_PSEFS | D0VWY2\_PSEFS | GO:0016849 | 0.00459057071960298 | 37/8060 | 4/235 | 0.0218966917952681 | 1 | T | T | F | F | phosphorus-oxygen lyase activity | PLC\_BACCE | PLC\_BACTU | PLC\_LISMO | PLC\_STAAE | GO:0004519 | 0.012531017369727 | 101/8060 | 6/235 | 0.0742403493415461 | 1 | F | F | F | F | endonuclease activity | CDTB\_ECOLX | DRNE\_VIBCH | NUCA\_SERMA | Q2XSK9\_VIBCL | Q2XSL7\_ALISL | RNS3\_KITAU | GO:0016757 | 0.0358560794044665 | 289/8060 | 13/235 | 0.0796394053950256 | 1 | F | F | F | F | glycosyltransferase activity | A0A0M3KKU6\_ERWAE | AMYS\_NEIPO | B2IF78\_BEII9 | B2VCC3\_ERWT9 | CDGT\_BACS0 | CDGT\_GEOSE | CHXA\_VIBCL | CRAX\_BACCE | D5DC07\_PRIM3 | D8J9C2\_HALJB | Q9ADS9\_STAAU | SACB\_BACSU | SACB\_GLUDI | GO:0004725 | 0.00223325062034739 | 18/8060 | 2/235 | 0.0954052467126399 | 1 | F | F | F | F | protein tyrosine phosphatase activity | A0A378K9X8\_LEGPN | Q6MNP0\_BDEBA | GO:0016151 | 0.00223325062034739 | 18/8060 | 2/235 | 0.0954052467126399 | 1 | F | F | F | F | nickel cation binding | ENG1\_ACET2 | LYTM\_STAA8 | GO:0046872 | 0.350992555831265 | 2829/8060 | 92/235 | 0.106114903087648 | 1 | F | F | F | F | metal ion binding | A0A0H2WX20\_STAAC | A0A160EBC2\_ACHDE | A0A6L7H1P2\_BACAN | A1TSQ3\_ACIAC | A3DJ82\_ACET2 | A5I055\_CLOBH | A8FDC4\_BACP2 | AMT6\_BACS7 | AMYB\_BACCE | AMYM\_GEOSE | AMY\_BACAM | AMY\_GEOSE | B9ZZP0\_9BACI | BSAP\_BACSU | BXX\_CLOBO | CBPM\_STRAL | CDGT\_BACS0 | CDGT\_GEOSE | CRAX\_BACCE | D0CAF1\_ACIB2 | D0VV09\_BACCE | D1A807\_THECD | D5DC07\_PRIM3 | D8H130\_BACAI | EFEN\_BACSU | ENG1\_ACET2 | ENTC2\_STAAU | FLS\_FERPE | GUNS\_ACETH | I3P686\_BACLI | LCHMO\_ENTFA | LIP\_BACSP | LIP\_PSEAE | LYTM\_STAA8 | NDK\_ACIBS | NDK\_AQUAE | NDK\_BURTA | NDK\_CAMJE | NDK\_HELPG | NDK\_MYXXA | NDK\_NEIG2 | NUCA\_SERMA | O06496\_CLOPF | O30700\_9BACI | O52754\_RHOMR | O66015\_GEOSE | O68424\_BACFG | O69771\_9PSED | O82839\_BACSP | O86049\_BACFG | PHLC1\_CLOPE | PHLC\_BACCE | PHLC\_CLOP1 | PHLC\_CLOPF | PLYL\_DICD3 | PLY\_BACSU | PRZN\_SERME | PTLY\_THEMA | Q186I6\_CLOD6 | Q2XSL7\_ALISL | Q47465\_PECCA | Q52NH3\_BACAN | Q5I4I3\_9BACI | Q5M4Z1\_STRT2 | Q5MJ80\_SERPR | Q7LYT7\_PYRWO | Q8ZRI7\_SALTY | Q93A71\_GEOSE | Q93I48\_9BACI | Q9AJS0\_ALIAC | Q9EYQ2\_9FIRM | Q9KWY6\_GEOSE | Q9L6D3\_GEOSE | Q9RC94\_9BACI | Q9RHW0\_BACSP | Q9RKQ2\_STRCO | Q9RQQ5\_STRPY | Q9ZBW9\_STRCO | RHA78\_STRAW | SODF\_METTH | SODM1\_STAA8 | SODM2\_BACAN | SODM2\_STAA8 | SODM\_BACSU | SODM\_GEOSE | SODM\_PROFR | SODM\_STRMU | SUBD\_BACLI | UL25\_PSEXP | V5IRV7\_THETH | V5VIC4\_ACIBA | XANLY\_BACGL | GO:0061783 | 0.00248138957816377 | 20/8060 | 2/235 | 0.11413406237719 | 1 | F | F | F | F | peptidoglycan muralytic activity | A0R5R2\_MYCS2 | O06496\_CLOPF | GO:0043169 | 0.353846153846154 | 2852/8060 | 92/235 | 0.124313540852113 | 1 | F | F | F | F | cation binding | A0A0H2WX20\_STAAC | A0A160EBC2\_ACHDE | A0A6L7H1P2\_BACAN | A1TSQ3\_ACIAC | A3DJ82\_ACET2 | A5I055\_CLOBH | A8FDC4\_BACP2 | AMT6\_BACS7 | AMYB\_BACCE | AMYM\_GEOSE | AMY\_BACAM | AMY\_GEOSE | B9ZZP0\_9BACI | BSAP\_BACSU | BXX\_CLOBO | CBPM\_STRAL | CDGT\_BACS0 | CDGT\_GEOSE | CRAX\_BACCE | D0CAF1\_ACIB2 | D0VV09\_BACCE | D1A807\_THECD | D5DC07\_PRIM3 | D8H130\_BACAI | EFEN\_BACSU | ENG1\_ACET2 | ENTC2\_STAAU | FLS\_FERPE | GUNS\_ACETH | I3P686\_BACLI | LCHMO\_ENTFA | LIP\_BACSP | LIP\_PSEAE | LYTM\_STAA8 | NDK\_ACIBS | NDK\_AQUAE | NDK\_BURTA | NDK\_CAMJE | NDK\_HELPG | NDK\_MYXXA | NDK\_NEIG2 | NUCA\_SERMA | O06496\_CLOPF | O30700\_9BACI | O52754\_RHOMR | O66015\_GEOSE | O68424\_BACFG | O69771\_9PSED | O82839\_BACSP | O86049\_BACFG | PHLC1\_CLOPE | PHLC\_BACCE | PHLC\_CLOP1 | PHLC\_CLOPF | PLYL\_DICD3 | PLY\_BACSU | PRZN\_SERME | PTLY\_THEMA | Q186I6\_CLOD6 | Q2XSL7\_ALISL | Q47465\_PECCA | Q52NH3\_BACAN | Q5I4I3\_9BACI | Q5M4Z1\_STRT2 | Q5MJ80\_SERPR | Q7LYT7\_PYRWO | Q8ZRI7\_SALTY | Q93A71\_GEOSE | Q93I48\_9BACI | Q9AJS0\_ALIAC | Q9EYQ2\_9FIRM | Q9KWY6\_GEOSE | Q9L6D3\_GEOSE | Q9RC94\_9BACI | Q9RHW0\_BACSP | Q9RKQ2\_STRCO | Q9RQQ5\_STRPY | Q9ZBW9\_STRCO | RHA78\_STRAW | SODF\_METTH | SODM1\_STAA8 | SODM2\_BACAN | SODM2\_STAA8 | SODM\_BACSU | SODM\_GEOSE | SODM\_PROFR | SODM\_STRMU | SUBD\_BACLI | UL25\_PSEXP | V5IRV7\_THETH | V5VIC4\_ACIBA | XANLY\_BACGL | GO:0004721 | 0.00310173697270471 | 25/8060 | 2/235 | 0.16429122359833 | 1 | F | F | F | F | phosphoprotein phosphatase activity | A0A378K9X8\_LEGPN | Q6MNP0\_BDEBA | GO:0009002 | 0.00334987593052109 | 27/8060 | 2/235 | 0.185304550261387 | 1 | F | F | F | F | serine-type D-Ala-D-Ala carboxypeptidase activity | DACC\_BACSU | DAC\_STRSR | GO:0004180 | 0.00682382133995037 | 55/8060 | 3/235 | 0.215637815020869 | 1 | F | F | F | F | carboxypeptidase activity | CBPM\_STRAL | DACC\_BACSU | DAC\_STRSR | GO:0004518 | 0.0172456575682382 | 139/8060 | 6/235 | 0.21955270916072 | 1 | F | F | F | F | nuclease activity | CDTB\_ECOLX | DRNE\_VIBCH | NUCA\_SERMA | Q2XSK9\_VIBCL | Q2XSL7\_ALISL | RNS3\_KITAU | GO:0004185 | 0.00409429280397022 | 33/8060 | 2/235 | 0.25003645696508 | 1 | F | F | F | F | serine-type carboxypeptidase activity | DACC\_BACSU | DAC\_STRSR | GO:0070008 | 0.0043424317617866 | 35/8060 | 2/235 | 0.271849952725948 | 1 | F | F | F | F | serine-type exopeptidase activity | DACC\_BACSU | DAC\_STRSR | GO:0016791 | 0.019106699751861 | 154/8060 | 6/235 | 0.293156390910017 | 1 | F | F | F | F | phosphatase activity | A0A378K9X8\_LEGPN | B4SL31\_STRM5 | PHYC\_BACIU | PHYT\_BACSD | Q5ZWG6\_LEGPH | Q6MNP0\_BDEBA | GO:0016684 | 0.0119106699751861 | 96/8060 | 4/235 | 0.306843267273949 | 1 | F | F | F | F | oxidoreductase activity, acting on peroxide as acceptor | D1A807\_THECD | EFEN\_BACSU | Q9RKQ2\_STRCO | Q9ZBW9\_STRCO | GO:0004601 | 0.0119106699751861 | 96/8060 | 4/235 | 0.306843267273949 | 1 | F | F | F | F | peroxidase activity | D1A807\_THECD | EFEN\_BACSU | Q9RKQ2\_STRCO | Q9ZBW9\_STRCO | GO:0016810 | 0.0673697270471464 | 543/8060 | 18/235 | 0.319426334533674 | 1 | F | F | F | F | hydrolase activity, acting on carbon-nitrogen (but not peptide) bonds | A0A4V8H037\_9RHOB | A0R5R2\_MYCS2 | A5N1B8\_CLOK5 | B4EPS2\_BURCJ | BLAC\_BACSU | BLAC\_STRAL | C3X8T5\_OXAFO | C3X9F9\_OXAFO | C3X9N1\_OXAFO | D2ARB3\_STRRD | D5DH82\_PRIM3 | D9TA79\_MICAI | HCPB\_HELPY | HCPC\_HELPY | P94458\_BACLI | Q79DR3\_ECOLX | Q81DA0\_BACCR | Q9XB24\_KLEPN | GO:0008235 | 0.00521091811414392 | 42/8060 | 2/235 | 0.3476015285745 | 1 | F | F | F | F | metalloexopeptidase activity | BSAP\_BACSU | CBPM\_STRAL | GO:0016835 | 0.0523573200992556 | 422/8060 | 14/235 | 0.34760754433686 | 1 | F | F | F | F | carbon-oxygen lyase activity | A0A160EBC2\_ACHDE | A1TSQ3\_ACIAC | ALYA\_KLEPN | D0VP31\_9BACI | G0L2Y1\_ZOBGA | HYSA\_STRA3 | PLYL\_DICD3 | PLY\_BACSU | PTLY\_THEMA | Q47465\_PECCA | Q8P6Z9\_XANCP | Q9AJM4\_9BACI | Q9RHW0\_BACSP | XANLY\_BACGL | GO:0008238 | 0.0166253101736973 | 134/8060 | 5/235 | 0.352497015035945 | 1 | F | F | F | F | exopeptidase activity | BSAP\_BACSU | CBPM\_STRAL | DACC\_BACSU | DAC\_STRSR | PEPX\_LACHE | GO:0008374 | 0.00570719602977668 | 46/8060 | 2/235 | 0.389779868579173 | 1 | F | F | F | F | O-acyltransferase activity | A85B\_MYCTU | A85C\_MYCTU | GO:0008270 | 0.0409429280397022 | 330/8060 | 10/235 | 0.496734988521121 | 1 | F | F | F | F | zinc ion binding | BXX\_CLOBO | LYTM\_STAA8 | O68424\_BACFG | O69771\_9PSED | O86049\_BACFG | PHLC1\_CLOPE | PHLC\_BACCE | PHLC\_CLOP1 | PHLC\_CLOPF | PRZN\_SERME | GO:0004177 | 0.00794044665012407 | 64/8060 | 2/235 | 0.56119153892107 | 1 | F | F | F | F | aminopeptidase activity | BSAP\_BACSU | PEPX\_LACHE | GO:0020037 | 0.0204714640198511 | 165/8060 | 4/235 | 0.714262155563495 | 1 | F | F | F | F | heme binding | D1A807\_THECD | EFEN\_BACSU | Q9RKQ2\_STRCO | Q9ZBW9\_STRCO | GO:0046906 | 0.0210918114143921 | 170/8060 | 4/235 | 0.73591533478811 | 1 | F | F | F | F | tetrapyrrole binding | D1A807\_THECD | EFEN\_BACSU | Q9RKQ2\_STRCO | Q9ZBW9\_STRCO | GO:0016829 | 0.129776674937965 | 1046/8060 | 25/235 | 0.883456827597633 | 1 | F | F | F | F | lyase activity | A0A160EBC2\_ACHDE | A1TSQ3\_ACIAC | ALYA\_KLEPN | D0VP31\_9BACI | EFEN\_BACSU | G0L2Y1\_ZOBGA | HYSA\_STRA3 | P84141\_PAEAU | PLC\_BACCE | PLC\_BACTU | PLC\_LISMO | PLC\_STAAE | PLYL\_DICD3 | PLY\_BACSU | PTLY\_THEMA | Q47465\_PECCA | Q5E1U2\_ALIF1 | Q8P6Z9\_XANCP | Q9AJM4\_9BACI | Q9RHW0\_BACSP | Q9RKQ2\_STRCO | Q9ZBW9\_STRCO | RNS3\_KITAU | UL25\_PSEXP | XANLY\_BACGL | GO:0016301 | 0.053970223325062 | 435/8060 | 9/235 | 0.894909445827214 | 1 | F | F | F | F | kinase activity | D0CAF1\_ACIB2 | NDK\_ACIBS | NDK\_AQUAE | NDK\_BURTA | NDK\_CAMJE | NDK\_HELPG | NDK\_MYXXA | NDK\_NEIG2 | V5VIC4\_ACIBA | GO:0003674 | 0.999751861042184 | 8058/8060 | 235/235 | 0.942533923973208 | 1 | F | F | F | F | molecular\_function | A0A067XG64\_PARTM | A0A0B7LH44\_STREE | A0A0H2UQE4\_STRPN | A0A0H2WX20\_STAAC | A0A0H3KHT8\_BURM1 | A0A0M3KKU6\_ERWAE | A0A0M3KKW7\_STRPY | A0A160EBC2\_ACHDE | A0A182DW00\_STREE | A0A1L1QK12\_STRSQ | A0A1L1QK13\_9ACTN | A0A1L1QK16\_9ACTN | A0A243G6Q6\_BACTU | A0A378K9X8\_LEGPN | A0A4P8ESF9\_BACSP | A0A4V8H037\_9RHOB | A0A6L7H1P2\_BACAN | A0Q436\_FRATN | A0R5R2\_MYCS2 | A1TSQ3\_ACIAC | A3DJ82\_ACET2 | A5I055\_CLOBH | A5N1B8\_CLOK5 | A85B\_MYCTU | A85C\_MYCTU | A8FDC4\_BACP2 | ALYA\_KLEPN | AMT6\_BACS7 | AMYB\_BACCE | AMYM\_GEOSE | AMYS\_NEIPO | AMY\_BACAM | AMY\_GEOSE | B2IF78\_BEII9 | B2VCC3\_ERWT9 | B4EPS2\_BURCJ | B4SL31\_STRM5 | B9MMA5\_CALBD | B9ZZP0\_9BACI | BLAC\_BACSU | BLAC\_STRAL | BSAP\_BACSU | BXX\_CLOBO | C3K8K1\_PSEFS | C3X8T5\_OXAFO | C3X9F9\_OXAFO | C3X9N1\_OXAFO | C7ED31\_ACET1 | CBPM\_STRAL | CDGT\_BACS0 | CDGT\_GEOSE | CDTB\_ECOLX | CHIS\_BACSU | CHIS\_STRSN | CHOD\_STRS0 | CHXA\_VIBCL | CRAX\_BACCE | D0CAF1\_ACIB2 | D0VP31\_9BACI | D0VV09\_BACCE | D0VWY2\_PSEFS | D0VXY8\_STAPS | D1A807\_THECD | D2ARB3\_STRRD | D5DC07\_PRIM3 | D5DH82\_PRIM3 | D8H130\_BACAI | D8J9C2\_HALJB | D9TA79\_MICAI | DACC\_BACSU | DAC\_STRSR | DRNE\_VIBCH | E4Q2A4\_CALOW | EBA1\_ELIME | EBA3\_ELIME | EFEN\_BACSU | ENG1\_ACET2 | ENTC2\_STAAU | ESTA\_BACSU | ESTA\_PSEAE | ESTA\_STRSC | ETA\_STAAU | ETB\_STAAU | FLS\_FERPE | G0L2Y1\_ZOBGA | G2NRC4\_STREK | G7CF24\_MYCT3 | G8LZE0\_ACECE | GSEA\_STAES | GUB\_BACLI | GUB\_BACSU | GUNA\_ACET2 | GUNC\_ACETH | GUNF\_RUMCH | GUNG\_RUMCH | GUNS\_ACETH | GUN\_ECOLI | H0B8D4\_9ACTN | HCPB\_HELPY | HCPC\_HELPY | HYSA\_STRA3 | I3P686\_BACLI | IABF\_STRAW | K0IUV6\_9ENTR | L0EGW1\_THECK | L0RUV7\_STAAU | LCHMO\_ENTFA | LIP\_BACSP | LIP\_PSEAE | LYTM\_STAA8 | MPT51\_MYCTU | NANB\_STRPN | NANH\_VIBCH | NDK\_ACIBS | NDK\_AQUAE | NDK\_BURTA | NDK\_CAMJE | NDK\_HELPG | NDK\_MYXXA | NDK\_NEIG2 | NUCA\_SERMA | O06496\_CLOPF | O30700\_9BACI | O31243\_RHIRD | O52754\_RHOMR | O66015\_GEOSE | O68424\_BACFG | O69771\_9PSED | O82839\_BACSP | O86049\_BACFG | P84141\_PAEAU | P84147\_STRSM | P94458\_BACLI | PEPX\_LACHE | PGLR2\_PECPM | PHL2\_BACCE | PHLC1\_CLOPE | PHLC\_BACCE | PHLC\_CLOP1 | PHLC\_CLOPF | PHLC\_STAAU | PHYC\_BACIU | PHYT\_BACSD | PLC\_BACCE | PLC\_BACTU | PLC\_LISMO | PLC\_STAAE | PLD\_STRAT | PLYL\_DICD3 | PLY\_BACSU | PMEA\_DICD3 | PRZN\_SERME | PTLY\_THEMA | Q09LY9\_GEOSE | Q17TM8\_ALKHA | Q186I6\_CLOD6 | Q21KE5\_SACD2 | Q2XSK9\_VIBCL | Q2XSL7\_ALISL | Q47465\_PECCA | Q52NH3\_BACAN | Q54276\_SERMA | Q59962\_9ACTN | Q5DZ44\_ALIF1 | Q5E1U2\_ALIF1 | Q5I4I3\_9BACI | Q5M4Z1\_STRT2 | Q5MJ80\_SERPR | Q5ZWG6\_LEGPH | Q65GB9\_BACLD | Q65JI7\_BACLD | Q65JI8\_BACLD | Q6MNP0\_BDEBA | Q6RSN8\_ACETH | Q79DR3\_ECOLX | Q7LYT7\_PYRWO | Q7SID8\_BACIU | Q7X4S4\_BACLI | Q81DA0\_BACCR | Q82L26\_STRAW | Q844J9\_BACTU | Q88JL2\_PSEPK | Q8L1V2\_GEOTH | Q8P6Z9\_XANCP | Q8ZRI7\_SALTY | Q93A71\_GEOSE | Q93I48\_9BACI | Q9ADS9\_STAAU | Q9AJF8\_ACETH | Q9AJM4\_9BACI | Q9AJS0\_ALIAC | Q9EYQ2\_9FIRM | Q9I229\_PSEAE | Q9JZ43\_NEIMB | Q9KWY6\_GEOSE | Q9L6D3\_GEOSE | Q9RC94\_9BACI | Q9REI6\_ARTSP | Q9RHW0\_BACSP | Q9RKQ2\_STRCO | Q9RQQ5\_STRPY | Q9XB24\_KLEPN | Q9ZBW9\_STRCO | RHA78\_STRAW | RIPB\_MYCTU | RNS3\_KITAU | SACB\_BACSU | SACB\_GLUDI | SFAS2\_STRFR | SODF\_METTH | SODM1\_STAA8 | SODM2\_BACAN | SODM2\_STAA8 | SODM\_BACSU | SODM\_GEOSE | SODM\_PROFR | SODM\_STRMU | SPLA\_STAA8 | SPLB\_STAA8 | SPLC\_STAA8 | SPLD\_STAA8 | SPLE\_STAA8 | SSL1\_STAA8 | SUBD\_BACLI | UL25\_PSEXP | V5IRV7\_THETH | V5VIC4\_ACIBA | W5JXE0\_BACSP | W8FKE7\_BACPU | X5I2D7\_CLOPF | XANLY\_BACGL | XYN1\_GEOSE | XYNA\_HALH5 | XYNC\_BACSU | YKFC\_BACC1 | GO:0016763 | 0.0193548387096774 | 156/8060 | 2/235 | 0.945377626035791 | 1 | F | F | F | F | pentosyltransferase activity | CHXA\_VIBCL | Q9ADS9\_STAAU | GO:0016779 | 0.0227047146401985 | 183/8060 | 2/235 | 0.972314454423613 | 1 | F | F | F | F | nucleotidyltransferase activity | CHXA\_VIBCL | CRAX\_BACCE | GO:0016772 | 0.0785359801488834 | 633/8060 | 11/235 | 0.981101282623596 | 1 | F | F | F | F | transferase activity, transferring phosphorus-containing groups | CHXA\_VIBCL | CRAX\_BACCE | D0CAF1\_ACIB2 | NDK\_ACIBS | NDK\_AQUAE | NDK\_BURTA | NDK\_CAMJE | NDK\_HELPG | NDK\_MYXXA | NDK\_NEIG2 | V5VIC4\_ACIBA | GO:0016747 | 0.0357320099255583 | 288/8060 | 3/235 | 0.991504966339944 | 1 | F | F | F | F | acyltransferase activity, transferring groups other than amino-acyl groups | A85B\_MYCTU | A85C\_MYCTU | MPT51\_MYCTU | GO:0016746 | 0.0442928039702233 | 357/8060 | 4/235 | 0.993805433628062 | 1 | F | F | F | F | acyltransferase activity | A85B\_MYCTU | A85C\_MYCTU | MPT51\_MYCTU | Q8ZRI7\_SALTY | GO:0046914 | 0.0967741935483871 | 780/8060 | 12/235 | 0.996723039021117 | 1 | F | F | F | F | transition metal ion binding | BXX\_CLOBO | ENG1\_ACET2 | LCHMO\_ENTFA | LYTM\_STAA8 | O68424\_BACFG | O69771\_9PSED | O86049\_BACFG | PHLC1\_CLOPE | PHLC\_BACCE | PHLC\_CLOP1 | PHLC\_CLOPF | PRZN\_SERME | GO:0003824 | 0.984863523573201 | 7938/8060 | 226/235 | 0.997097308451515 | 1 | F | F | F | F | catalytic activity | A0A067XG64\_PARTM | A0A0B7LH44\_STREE | A0A0H2UQE4\_STRPN | A0A0H2WX20\_STAAC | A0A0H3KHT8\_BURM1 | A0A0M3KKU6\_ERWAE | A0A0M3KKW7\_STRPY | A0A160EBC2\_ACHDE | A0A182DW00\_STREE | A0A1L1QK12\_STRSQ | A0A1L1QK13\_9ACTN | A0A1L1QK16\_9ACTN | A0A243G6Q6\_BACTU | A0A378K9X8\_LEGPN | A0A4P8ESF9\_BACSP | A0A4V8H037\_9RHOB | A0A6L7H1P2\_BACAN | A0Q436\_FRATN | A0R5R2\_MYCS2 | A1TSQ3\_ACIAC | A3DJ82\_ACET2 | A5N1B8\_CLOK5 | A85B\_MYCTU | A85C\_MYCTU | A8FDC4\_BACP2 | ALYA\_KLEPN | AMT6\_BACS7 | AMYB\_BACCE | AMYM\_GEOSE | AMYS\_NEIPO | AMY\_BACAM | AMY\_GEOSE | B2IF78\_BEII9 | B2VCC3\_ERWT9 | B4EPS2\_BURCJ | B4SL31\_STRM5 | B9MMA5\_CALBD | BLAC\_BACSU | BLAC\_STRAL | BSAP\_BACSU | BXX\_CLOBO | C3X8T5\_OXAFO | C3X9F9\_OXAFO | C3X9N1\_OXAFO | C7ED31\_ACET1 | CBPM\_STRAL | CDGT\_BACS0 | CDGT\_GEOSE | CDTB\_ECOLX | CHIS\_BACSU | CHIS\_STRSN | CHOD\_STRS0 | CHXA\_VIBCL | CRAX\_BACCE | D0CAF1\_ACIB2 | D0VP31\_9BACI | D0VV09\_BACCE | D0VXY8\_STAPS | D1A807\_THECD | D2ARB3\_STRRD | D5DC07\_PRIM3 | D5DH82\_PRIM3 | D8H130\_BACAI | D8J9C2\_HALJB | D9TA79\_MICAI | DACC\_BACSU | DAC\_STRSR | DRNE\_VIBCH | E4Q2A4\_CALOW | EBA1\_ELIME | EBA3\_ELIME | EFEN\_BACSU | ENG1\_ACET2 | ESTA\_BACSU | ESTA\_PSEAE | ESTA\_STRSC | ETA\_STAAU | ETB\_STAAU | FLS\_FERPE | G0L2Y1\_ZOBGA | G2NRC4\_STREK | G7CF24\_MYCT3 | G8LZE0\_ACECE | GSEA\_STAES | GUB\_BACLI | GUB\_BACSU | GUNA\_ACET2 | GUNC\_ACETH | GUNF\_RUMCH | GUNG\_RUMCH | GUNS\_ACETH | GUN\_ECOLI | H0B8D4\_9ACTN | HCPB\_HELPY | HCPC\_HELPY | HYSA\_STRA3 | I3P686\_BACLI | IABF\_STRAW | K0IUV6\_9ENTR | L0EGW1\_THECK | L0RUV7\_STAAU | LCHMO\_ENTFA | LIP\_BACSP | LIP\_PSEAE | LYTM\_STAA8 | MPT51\_MYCTU | NANB\_STRPN | NANH\_VIBCH | NDK\_ACIBS | NDK\_AQUAE | NDK\_BURTA | NDK\_CAMJE | NDK\_HELPG | NDK\_MYXXA | NDK\_NEIG2 | NUCA\_SERMA | O06496\_CLOPF | O30700\_9BACI | O31243\_RHIRD | O52754\_RHOMR | O66015\_GEOSE | O68424\_BACFG | O69771\_9PSED | O82839\_BACSP | O86049\_BACFG | P84141\_PAEAU | P84147\_STRSM | P94458\_BACLI | PEPX\_LACHE | PGLR2\_PECPM | PHL2\_BACCE | PHLC1\_CLOPE | PHLC\_BACCE | PHLC\_CLOP1 | PHLC\_CLOPF | PHLC\_STAAU | PHYC\_BACIU | PHYT\_BACSD | PLC\_BACCE | PLC\_BACTU | PLC\_LISMO | PLC\_STAAE | PLD\_STRAT | PLYL\_DICD3 | PLY\_BACSU | PMEA\_DICD3 | PRZN\_SERME | PTLY\_THEMA | Q09LY9\_GEOSE | Q17TM8\_ALKHA | Q186I6\_CLOD6 | Q21KE5\_SACD2 | Q2XSK9\_VIBCL | Q2XSL7\_ALISL | Q47465\_PECCA | Q52NH3\_BACAN | Q54276\_SERMA | Q59962\_9ACTN | Q5DZ44\_ALIF1 | Q5E1U2\_ALIF1 | Q5I4I3\_9BACI | Q5M4Z1\_STRT2 | Q5MJ80\_SERPR | Q5ZWG6\_LEGPH | Q65GB9\_BACLD | Q65JI7\_BACLD | Q65JI8\_BACLD | Q6MNP0\_BDEBA | Q6RSN8\_ACETH | Q79DR3\_ECOLX | Q7LYT7\_PYRWO | Q7SID8\_BACIU | Q7X4S4\_BACLI | Q81DA0\_BACCR | Q82L26\_STRAW | Q88JL2\_PSEPK | Q8L1V2\_GEOTH | Q8P6Z9\_XANCP | Q8ZRI7\_SALTY | Q93A71\_GEOSE | Q93I48\_9BACI | Q9ADS9\_STAAU | Q9AJF8\_ACETH | Q9AJM4\_9BACI | Q9AJS0\_ALIAC | Q9EYQ2\_9FIRM | Q9I229\_PSEAE | Q9JZ43\_NEIMB | Q9KWY6\_GEOSE | Q9RC94\_9BACI | Q9REI6\_ARTSP | Q9RHW0\_BACSP | Q9RKQ2\_STRCO | Q9XB24\_KLEPN | Q9ZBW9\_STRCO | RHA78\_STRAW | RIPB\_MYCTU | RNS3\_KITAU | SACB\_BACSU | SACB\_GLUDI | SFAS2\_STRFR | SODF\_METTH | SODM1\_STAA8 | SODM2\_BACAN | SODM2\_STAA8 | SODM\_BACSU | SODM\_GEOSE | SODM\_PROFR | SODM\_STRMU | SPLA\_STAA8 | SPLB\_STAA8 | SPLC\_STAA8 | SPLD\_STAA8 | SPLE\_STAA8 | SSL1\_STAA8 | SUBD\_BACLI | UL25\_PSEXP | V5IRV7\_THETH | V5VIC4\_ACIBA | W5JXE0\_BACSP | W8FKE7\_BACPU | XANLY\_BACGL | XYN1\_GEOSE | XYNA\_HALH5 | XYNC\_BACSU | YKFC\_BACC1 | GO:0005515 | 0.0441687344913151 | 356/8060 | 3/235 | 0.998445181634312 | 1 | F | F | F | F | protein binding | A85B\_MYCTU | A85C\_MYCTU | MPT51\_MYCTU | GO:0043167 | 0.507444168734491 | 4090/8060 | 96/235 | 0.999188350025041 | 1 | F | F | F | F | ion binding | A0A0H2WX20\_STAAC | A0A160EBC2\_ACHDE | A0A6L7H1P2\_BACAN | A1TSQ3\_ACIAC | A3DJ82\_ACET2 | A5I055\_CLOBH | A8FDC4\_BACP2 | AMT6\_BACS7 | AMYB\_BACCE | AMYM\_GEOSE | AMY\_BACAM | AMY\_GEOSE | B9ZZP0\_9BACI | BSAP\_BACSU | BXX\_CLOBO | C3K8K1\_PSEFS | CBPM\_STRAL | CDGT\_BACS0 | CDGT\_GEOSE | CHOD\_STRS0 | CRAX\_BACCE | D0CAF1\_ACIB2 | D0VV09\_BACCE | D0VWY2\_PSEFS | D1A807\_THECD | D5DC07\_PRIM3 | D8H130\_BACAI | EFEN\_BACSU | ENG1\_ACET2 | ENTC2\_STAAU | FLS\_FERPE | GUNS\_ACETH | I3P686\_BACLI | LCHMO\_ENTFA | LIP\_BACSP | LIP\_PSEAE | LYTM\_STAA8 | NANH\_VIBCH | NDK\_ACIBS | NDK\_AQUAE | NDK\_BURTA | NDK\_CAMJE | NDK\_HELPG | NDK\_MYXXA | NDK\_NEIG2 | NUCA\_SERMA | O06496\_CLOPF | O30700\_9BACI | O52754\_RHOMR | O66015\_GEOSE | O68424\_BACFG | O69771\_9PSED | O82839\_BACSP | O86049\_BACFG | PHLC1\_CLOPE | PHLC\_BACCE | PHLC\_CLOP1 | PHLC\_CLOPF | PLYL\_DICD3 | PLY\_BACSU | PRZN\_SERME | PTLY\_THEMA | Q186I6\_CLOD6 | Q2XSL7\_ALISL | Q47465\_PECCA | Q52NH3\_BACAN | Q5I4I3\_9BACI | Q5M4Z1\_STRT2 | Q5MJ80\_SERPR | Q7LYT7\_PYRWO | Q8ZRI7\_SALTY | Q93A71\_GEOSE | Q93I48\_9BACI | Q9AJS0\_ALIAC | Q9EYQ2\_9FIRM | Q9KWY6\_GEOSE | Q9L6D3\_GEOSE | Q9RC94\_9BACI | Q9RHW0\_BACSP | Q9RKQ2\_STRCO | Q9RQQ5\_STRPY | Q9ZBW9\_STRCO | RHA78\_STRAW | SODF\_METTH | SODM1\_STAA8 | SODM2\_BACAN | SODM2\_STAA8 | SODM\_BACSU | SODM\_GEOSE | SODM\_PROFR | SODM\_STRMU | SUBD\_BACLI | UL25\_PSEXP | V5IRV7\_THETH | V5VIC4\_ACIBA | XANLY\_BACGL | GO:0003676 | 0.0398263027295285 | 321/8060 | 2/235 | 0.999319351468031 | 1 | F | F | F | F | nucleic acid binding | NUCA\_SERMA | RNS3\_KITAU | GO:0097367 | 0.142059553349876 | 1145/8060 | 16/235 | 0.999891130531435 | 1 | F | F | F | F | carbohydrate derivative binding | A0A243G6Q6\_BACTU | A0A6L7H1P2\_BACAN | D0CAF1\_ACIB2 | D0VV09\_BACCE | LCHMO\_ENTFA | NANH\_VIBCH | NDK\_ACIBS | NDK\_AQUAE | NDK\_BURTA | NDK\_CAMJE | NDK\_HELPG | NDK\_MYXXA | NDK\_NEIG2 | Q54276\_SERMA | Q9REI6\_ARTSP | V5VIC4\_ACIBA | GO:0140640 | 0.0562034739454094 | 453/8060 | 2/235 | 0.999984332638416 | 1 | F | F | F | F | catalytic activity, acting on a nucleic acid | DRNE\_VIBCH | RNS3\_KITAU | GO:0005524 | 0.110794044665012 | 893/8060 | 9/235 | 0.99998637751725 | 1 | F | F | F | F | ATP binding | D0CAF1\_ACIB2 | NDK\_ACIBS | NDK\_AQUAE | NDK\_BURTA | NDK\_CAMJE | NDK\_HELPG | NDK\_MYXXA | NDK\_NEIG2 | V5VIC4\_ACIBA | GO:0032559 | 0.112282878411911 | 905/8060 | 9/235 | 0.999989749460379 | 1 | F | F | F | F | adenyl ribonucleotide binding | D0CAF1\_ACIB2 | NDK\_ACIBS | NDK\_AQUAE | NDK\_BURTA | NDK\_CAMJE | NDK\_HELPG | NDK\_MYXXA | NDK\_NEIG2 | V5VIC4\_ACIBA | GO:0005488 | 0.610669975186104 | 4922/8060 | 111/235 | 0.999995114014826 | 1 | F | F | F | F | binding | A0A0H2WX20\_STAAC | A0A0H3KHT8\_BURM1 | A0A160EBC2\_ACHDE | A0A243G6Q6\_BACTU | A0A6L7H1P2\_BACAN | A1TSQ3\_ACIAC | A3DJ82\_ACET2 | A5I055\_CLOBH | A85B\_MYCTU | A85C\_MYCTU | A8FDC4\_BACP2 | AMT6\_BACS7 | AMYB\_BACCE | AMYM\_GEOSE | AMY\_BACAM | AMY\_GEOSE | B9ZZP0\_9BACI | BSAP\_BACSU | BXX\_CLOBO | C3K8K1\_PSEFS | CBPM\_STRAL | CDGT\_BACS0 | CDGT\_GEOSE | CHOD\_STRS0 | CRAX\_BACCE | D0CAF1\_ACIB2 | D0VV09\_BACCE | D0VWY2\_PSEFS | D1A807\_THECD | D5DC07\_PRIM3 | D8H130\_BACAI | EFEN\_BACSU | ENG1\_ACET2 | ENTC2\_STAAU | FLS\_FERPE | GUNG\_RUMCH | GUNS\_ACETH | HYSA\_STRA3 | I3P686\_BACLI | LCHMO\_ENTFA | LIP\_BACSP | LIP\_PSEAE | LYTM\_STAA8 | MPT51\_MYCTU | NANH\_VIBCH | NDK\_ACIBS | NDK\_AQUAE | NDK\_BURTA | NDK\_CAMJE | NDK\_HELPG | NDK\_MYXXA | NDK\_NEIG2 | NUCA\_SERMA | O06496\_CLOPF | O30700\_9BACI | O52754\_RHOMR | O66015\_GEOSE | O68424\_BACFG | O69771\_9PSED | O82839\_BACSP | O86049\_BACFG | P84141\_PAEAU | PHLC1\_CLOPE | PHLC\_BACCE | PHLC\_CLOP1 | PHLC\_CLOPF | PLYL\_DICD3 | PLY\_BACSU | PRZN\_SERME | PTLY\_THEMA | Q186I6\_CLOD6 | Q2XSL7\_ALISL | Q47465\_PECCA | Q52NH3\_BACAN | Q54276\_SERMA | Q5I4I3\_9BACI | Q5M4Z1\_STRT2 | Q5MJ80\_SERPR | Q7LYT7\_PYRWO | Q844J9\_BACTU | Q8ZRI7\_SALTY | Q93A71\_GEOSE | Q93I48\_9BACI | Q9ADS9\_STAAU | Q9AJF8\_ACETH | Q9AJS0\_ALIAC | Q9EYQ2\_9FIRM | Q9KWY6\_GEOSE | Q9L6D3\_GEOSE | Q9RC94\_9BACI | Q9REI6\_ARTSP | Q9RHW0\_BACSP | Q9RKQ2\_STRCO | Q9RQQ5\_STRPY | Q9ZBW9\_STRCO | RHA78\_STRAW | RNS3\_KITAU | SODF\_METTH | SODM1\_STAA8 | SODM2\_BACAN | SODM2\_STAA8 | SODM\_BACSU | SODM\_GEOSE | SODM\_PROFR | SODM\_STRMU | SUBD\_BACLI | UL25\_PSEXP | V5IRV7\_THETH | V5VIC4\_ACIBA | X5I2D7\_CLOPF | XANLY\_BACGL | GO:0035639 | 0.118982630272953 | 959/8060 | 9/235 | 0.999997215400861 | 1 | F | F | F | F | purine ribonucleoside triphosphate binding | D0CAF1\_ACIB2 | NDK\_ACIBS | NDK\_AQUAE | NDK\_BURTA | NDK\_CAMJE | NDK\_HELPG | NDK\_MYXXA | NDK\_NEIG2 | V5VIC4\_ACIBA | GO:0016614 | 0.0636476426799008 | 513/8060 | 2/235 | 0.999997320008523 | 1 | F | F | F | F | oxidoreductase activity, acting on CH-OH group of donors | A0A0H3KHT8\_BURM1 | CHOD\_STRS0 | GO:0032555 | 0.121588089330025 | 980/8060 | 9/235 | 0.999998339248754 | 1 | F | F | F | F | purine ribonucleotide binding | D0CAF1\_ACIB2 | NDK\_ACIBS | NDK\_AQUAE | NDK\_BURTA | NDK\_CAMJE | NDK\_HELPG | NDK\_MYXXA | NDK\_NEIG2 | V5VIC4\_ACIBA | GO:0032553 | 0.134491315136476 | 1084/8060 | 9/235 | 0.999999881148062 | 1 | F | F | F | F | ribonucleotide binding | D0CAF1\_ACIB2 | NDK\_ACIBS | NDK\_AQUAE | NDK\_BURTA | NDK\_CAMJE | NDK\_HELPG | NDK\_MYXXA | NDK\_NEIG2 | V5VIC4\_ACIBA | GO:0016740 | 0.275806451612903 | 2223/8060 | 26/235 | 0.999999999891566 | 1 | F | F | F | F | transferase activity | A0A0M3KKU6\_ERWAE | A85B\_MYCTU | A85C\_MYCTU | AMYS\_NEIPO | B2IF78\_BEII9 | B2VCC3\_ERWT9 | CDGT\_BACS0 | CDGT\_GEOSE | CHXA\_VIBCL | CRAX\_BACCE | D0CAF1\_ACIB2 | D5DC07\_PRIM3 | D8J9C2\_HALJB | MPT51\_MYCTU | NDK\_ACIBS | NDK\_AQUAE | NDK\_BURTA | NDK\_CAMJE | NDK\_HELPG | NDK\_MYXXA | NDK\_NEIG2 | Q8ZRI7\_SALTY | Q9ADS9\_STAAU | SACB\_BACSU | SACB\_GLUDI | V5VIC4\_ACIBA | GO:0030554 | 0.167369727047146 | 1349/8060 | 9/235 | 0.99999999991428 | 1 | F | F | F | F | adenyl nucleotide binding | D0CAF1\_ACIB2 | NDK\_ACIBS | NDK\_AQUAE | NDK\_BURTA | NDK\_CAMJE | NDK\_HELPG | NDK\_MYXXA | NDK\_NEIG2 | V5VIC4\_ACIBA | GO:0017076 | 0.176426799007444 | 1422/8060 | 9/235 | 0.999999999990423 | 1 | F | F | F | F | purine nucleotide binding | D0CAF1\_ACIB2 | NDK\_ACIBS | NDK\_AQUAE | NDK\_BURTA | NDK\_CAMJE | NDK\_HELPG | NDK\_MYXXA | NDK\_NEIG2 | V5VIC4\_ACIBA | GO:0016491 | 0.236104218362283 | 1903/8060 | 17/235 | 0.999999999997499 | 1 | F | F | F | F | oxidoreductase activity | A0A0H3KHT8\_BURM1 | CHOD\_STRS0 | D1A807\_THECD | EFEN\_BACSU | LCHMO\_ENTFA | Q186I6\_CLOD6 | Q5M4Z1\_STRT2 | Q9RKQ2\_STRCO | Q9ZBW9\_STRCO | SODF\_METTH | SODM1\_STAA8 | SODM2\_BACAN | SODM2\_STAA8 | SODM\_BACSU | SODM\_GEOSE | SODM\_PROFR | SODM\_STRMU | GO:1901363 | 0.303722084367246 | 2448/8060 | 15/235 | 0.999999999997549 | 1 | F | F | F | F | heterocyclic compound binding | A0A0H3KHT8\_BURM1 | CHOD\_STRS0 | CRAX\_BACCE | D0CAF1\_ACIB2 | NDK\_ACIBS | NDK\_AQUAE | NDK\_BURTA | NDK\_CAMJE | NDK\_HELPG | NDK\_MYXXA | NDK\_NEIG2 | Q844J9\_BACTU | Q9ADS9\_STAAU | V5VIC4\_ACIBA | X5I2D7\_CLOPF | GO:0043168 | 0.222208436724566 | 1791/8060 | 13/235 | 0.999999999998712 | 1 | F | F | F | F | anion binding | C3K8K1\_PSEFS | CHOD\_STRS0 | D0CAF1\_ACIB2 | D0VWY2\_PSEFS | NANH\_VIBCH | NDK\_ACIBS | NDK\_AQUAE | NDK\_BURTA | NDK\_CAMJE | NDK\_HELPG | NDK\_MYXXA | NDK\_NEIG2 | V5VIC4\_ACIBA | GO:0036094 | 0.309925558312655 | 2498/8060 | 17/235 | 0.999999999998728 | 1 | F | F | F | F | small molecule binding | A0A0H3KHT8\_BURM1 | CHOD\_STRS0 | CRAX\_BACCE | D0CAF1\_ACIB2 | NANH\_VIBCH | NDK\_ACIBS | NDK\_AQUAE | NDK\_BURTA | NDK\_CAMJE | NDK\_HELPG | NDK\_MYXXA | NDK\_NEIG2 | Q844J9\_BACTU | Q9ADS9\_STAAU | V5VIC4\_ACIBA | X5I2D7\_CLOPF | XANLY\_BACGL | GO:0097159 | 0.351240694789082 | 2831/8060 | 21/235 | 0.999999999999924 | 1 | F | F | F | F | organic cyclic compound binding | A0A0H3KHT8\_BURM1 | CHOD\_STRS0 | CRAX\_BACCE | D0CAF1\_ACIB2 | D1A807\_THECD | EFEN\_BACSU | NDK\_ACIBS | NDK\_AQUAE | NDK\_BURTA | NDK\_CAMJE | NDK\_HELPG | NDK\_MYXXA | NDK\_NEIG2 | NUCA\_SERMA | Q844J9\_BACTU | Q9ADS9\_STAAU | Q9RKQ2\_STRCO | Q9ZBW9\_STRCO | RNS3\_KITAU | V5VIC4\_ACIBA | X5I2D7\_CLOPF | GO:0000166 | 0.253846153846154 | 2046/8060 | 15/235 | 1 | 1 | F | F | F | F | nucleotide binding | A0A0H3KHT8\_BURM1 | CHOD\_STRS0 | CRAX\_BACCE | D0CAF1\_ACIB2 | NDK\_ACIBS | NDK\_AQUAE | NDK\_BURTA | NDK\_CAMJE | NDK\_HELPG | NDK\_MYXXA | NDK\_NEIG2 | Q844J9\_BACTU | Q9ADS9\_STAAU | V5VIC4\_ACIBA | X5I2D7\_CLOPF | GO:1901265 | 0.253846153846154 | 2046/8060 | 15/235 | 1 | 1 | F | F | F | F | nucleoside phosphate binding | A0A0H3KHT8\_BURM1 | CHOD\_STRS0 | CRAX\_BACCE | D0CAF1\_ACIB2 | NDK\_ACIBS | NDK\_AQUAE | NDK\_BURTA | NDK\_CAMJE | NDK\_HELPG | NDK\_MYXXA | NDK\_NEIG2 | Q844J9\_BACTU | Q9ADS9\_STAAU | V5VIC4\_ACIBA | X5I2D7\_CLOPF | GO:0030145 | 0.0147642679900744 | 119/8060 | 1/235 | NA | NA | NA | NA | NA | NA | manganese ion binding | LYTM\_STAA8 | GO:0106435 | 0.00310173697270471 | 25/8060 | 1/235 | NA | NA | NA | NA | NA | NA | carboxylesterase activity | ESTA\_PSEAE | GO:0140098 | 0.0437965260545906 | 353/8060 | 1/235 | NA | NA | NA | NA | NA | NA | catalytic activity, acting on RNA | RNS3\_KITAU | GO:0008745 | 0.00086848635235732 | 7/8060 | 1/235 | NA | NA | NA | NA | NA | NA | N-acetylmuramoyl-L-alanine amidase activity | A0R5R2\_MYCS2 | GO:0016833 | 0.00806451612903226 | 65/8060 | 1/235 | NA | NA | NA | NA | NA | NA | oxo-acid-lyase activity | Q5E1U2\_ALIF1 | GO:0043897 | 0.000124069478908189 | 1/8060 | 1/235 | NA | NA | NA | NA | NA | NA | glucan 1,4-alpha-maltohydrolase activity | AMYM\_GEOSE | GO:0030599 | 0.000372208436724566 | 3/8060 | 1/235 | NA | NA | NA | NA | NA | NA | pectinesterase activity | PMEA\_DICD3 | GO:0008700 | 0.000620347394540943 | 5/8060 | 1/235 | NA | NA | NA | NA | NA | NA | 4-hydroxy-2-oxoglutarate aldolase activity | Q5E1U2\_ALIF1 | GO:0008675 | 0.00148883374689826 | 12/8060 | 1/235 | NA | NA | NA | NA | NA | NA | 2-dehydro-3-deoxy-phosphogluconate aldolase activity | Q5E1U2\_ALIF1 | GO:0004090 | 0.000124069478908189 | 1/8060 | 1/235 | NA | NA | NA | NA | NA | NA | carbonyl reductase (NADPH) activity | A0A0H3KHT8\_BURM1 | GO:0016162 | 0.000248138957816377 | 2/8060 | 1/235 | NA | NA | NA | NA | NA | NA | cellulose 1,4-beta-cellobiosidase activity | Q6RSN8\_ACETH | GO:0030340 | 0.000248138957816377 | 2/8060 | 1/235 | NA | NA | NA | NA | NA | NA | hyaluronate lyase activity | HYSA\_STRA3 | GO:0016616 | 0.0600496277915633 | 484/8060 | 1/235 | NA | NA | NA | NA | NA | NA | oxidoreductase activity, acting on the CH-OH group of donors, NAD or NADP as acceptor | A0A0H3KHT8\_BURM1 | GO:0004557 | 0.00124069478908189 | 10/8060 | 1/235 | NA | NA | NA | NA | NA | NA | alpha-galactosidase activity | Q82L26\_STRAW | GO:0015925 | 0.00657568238213399 | 53/8060 | 1/235 | NA | NA | NA | NA | NA | NA | galactosidase activity | Q82L26\_STRAW | GO:0047669 | 0.000124069478908189 | 1/8060 | 1/235 | NA | NA | NA | NA | NA | NA | amylosucrase activity | AMYS\_NEIPO | GO:0016832 | 0.0137717121588089 | 111/8060 | 1/235 | NA | NA | NA | NA | NA | NA | aldehyde-lyase activity | Q5E1U2\_ALIF1 | GO:0003993 | 0.000992555831265509 | 8/8060 | 1/235 | NA | NA | NA | NA | NA | NA | acid phosphatase activity | Q5ZWG6\_LEGPH | GO:0004181 | 0.00136476426799007 | 11/8060 | 1/235 | NA | NA | NA | NA | NA | NA | metallocarboxypeptidase activity | CBPM\_STRAL | GO:0048029 | 0.00384615384615385 | 31/8060 | 1/235 | NA | NA | NA | NA | NA | NA | monosaccharide binding | XANLY\_BACGL | GO:0043177 | 0.0155086848635236 | 125/8060 | 1/235 | NA | NA | NA | NA | NA | NA | organic acid binding | NANH\_VIBCH | GO:0003723 | 0.019727047146402 | 159/8060 | 1/235 | NA | NA | NA | NA | NA | NA | RNA binding | RNS3\_KITAU | GO:0004540 | 0.00694789081885856 | 56/8060 | 1/235 | NA | NA | NA | NA | NA | NA | RNA nuclease activity | RNS3\_KITAU | GO:0016860 | 0.0210918114143921 | 170/8060 | 1/235 | NA | NA | NA | NA | NA | NA | intramolecular oxidoreductase activity | CHOD\_STRS0 | GO:0022884 | 0.000992555831265509 | 8/8060 | 1/235 | NA | NA | NA | NA | NA | NA | macromolecule transmembrane transporter activity | BXX\_CLOBO | GO:0047286 | 0.000496277915632754 | 4/8060 | 1/235 | NA | NA | NA | NA | NA | NA | NAD+-diphthamide ADP-ribosyltransferase activity | CHXA\_VIBCL | GO:0031406 | 0.0155086848635236 | 125/8060 | 1/235 | NA | NA | NA | NA | NA | NA | carboxylic acid binding | NANH\_VIBCH | GO:0005215 | 0.00632754342431762 | 51/8060 | 1/235 | NA | NA | NA | NA | NA | NA | transporter activity | BXX\_CLOBO | GO:0016790 | 0.00521091811414392 | 42/8060 | 1/235 | NA | NA | NA | NA | NA | NA | thiolester hydrolase activity | Q9JZ43\_NEIMB | GO:0033691 | 0.000124069478908189 | 1/8060 | 1/235 | NA | NA | NA | NA | NA | NA | sialic acid binding | NANH\_VIBCH | GO:0004521 | 0.00409429280397022 | 33/8060 | 1/235 | NA | NA | NA | NA | NA | NA | RNA endonuclease activity | RNS3\_KITAU | GO:0050897 | 0.00459057071960298 | 37/8060 | 1/235 | NA | NA | NA | NA | NA | NA | cobalt ion binding | LYTM\_STAA8 | GO:0005507 | 0.00719602977667494 | 58/8060 | 1/235 | NA | NA | NA | NA | NA | NA | copper ion binding | LCHMO\_ENTFA | GO:0004497 | 0.0253101736972705 | 204/8060 | 1/235 | NA | NA | NA | NA | NA | NA | monooxygenase activity | LCHMO\_ENTFA | GO:0016995 | 0.000248138957816377 | 2/8060 | 1/235 | NA | NA | NA | NA | NA | NA | cholesterol oxidase activity | CHOD\_STRS0 | GO:0052736 | 0.000620347394540943 | 5/8060 | 1/235 | NA | NA | NA | NA | NA | NA | beta-glucanase activity | ENG1\_ACET2 | GO:0004568 | 0.00086848635235732 | 7/8060 | 1/235 | NA | NA | NA | NA | NA | NA | chitinase activity | Q9REI6\_ARTSP | GO:0015926 | 0.00744416873449132 | 60/8060 | 1/235 | NA | NA | NA | NA | NA | NA | glucosidase activity | ENG1\_ACET2 | GO:0008422 | 0.00508684863523573 | 41/8060 | 1/235 | NA | NA | NA | NA | NA | NA | beta-glucosidase activity | ENG1\_ACET2 | GO:0140097 | 0.0122828784119107 | 99/8060 | 1/235 | NA | NA | NA | NA | NA | NA | catalytic activity, acting on DNA | DRNE\_VIBCH | GO:0008320 | 0.000992555831265509 | 8/8060 | 1/235 | NA | NA | NA | NA | NA | NA | protein transmembrane transporter activity | BXX\_CLOBO | GO:0046556 | 0.00173697270471464 | 14/8060 | 1/235 | NA | NA | NA | NA | NA | NA | alpha-L-arabinofuranosidase activity | IABF\_STRAW | GO:0004348 | 0.000496277915632754 | 4/8060 | 1/235 | NA | NA | NA | NA | NA | NA | glucosylceramidase activity | XYNC\_BACSU | GO:0004536 | 0.00521091811414392 | 42/8060 | 1/235 | NA | NA | NA | NA | NA | NA | DNA nuclease activity | DRNE\_VIBCH | GO:0000287 | 0.0796526054590571 | 642/8060 | 1/235 | NA | NA | NA | NA | NA | NA | magnesium ion binding | ENG1\_ACET2 | GO:0009046 | 0.000124069478908189 | 1/8060 | 1/235 | NA | NA | NA | NA | NA | NA | zinc D-Ala-D-Ala carboxypeptidase activity | CBPM\_STRAL | GO:0016899 | 0.00136476426799007 | 11/8060 | 1/235 | NA | NA | NA | NA | NA | NA | oxidoreductase activity, acting on the CH-OH group of donors, oxygen as acceptor | CHOD\_STRS0 | GO:0009044 | 0.00136476426799007 | 11/8060 | 1/235 | NA | NA | NA | NA | NA | NA | xylan 1,4-beta-xylosidase activity | A0A067XG64\_PARTM | GO:0047492 | 0.000248138957816377 | 2/8060 | 1/235 | NA | NA | NA | NA | NA | NA | xanthan lyase activity | XANLY\_BACGL | GO:0140318 | 0.000992555831265509 | 8/8060 | 1/235 | NA | NA | NA | NA | NA | NA | protein transporter activity | BXX\_CLOBO | GO:0052692 | 0.00124069478908189 | 10/8060 | 1/235 | NA | NA | NA | NA | NA | NA | raffinose alpha-galactosidase activity | Q82L26\_STRAW | GO:0016863 | 0.00334987593052109 | 27/8060 | 1/235 | NA | NA | NA | NA | NA | NA | intramolecular oxidoreductase activity, transposing C=C bonds | CHOD\_STRS0 | GO:0016853 | 0.0733250620347395 | 591/8060 | 1/235 | NA | NA | NA | NA | NA | NA | isomerase activity | CHOD\_STRS0 | GO:0042973 | 0.000496277915632754 | 4/8060 | 1/235 | NA | NA | NA | NA | NA | NA | glucan endo-1,3-beta-D-glucosidase activity | ENG1\_ACET2 | GO:0008239 | 0.000992555831265509 | 8/8060 | 1/235 | NA | NA | NA | NA | NA | NA | dipeptidyl-peptidase activity | PEPX\_LACHE | GO:0016161 | 0.000124069478908189 | 1/8060 | 1/235 | NA | NA | NA | NA | NA | NA | beta-amylase activity | AMYB\_BACCE | GO:0052861 | 0.000124069478908189 | 1/8060 | 1/235 | NA | NA | NA | NA | NA | NA | glucan endo-1,3-beta-glucanase activity, C-3 substituted reducing group | ENG1\_ACET2 | GO:0050660 | 0.0255583126550868 | 206/8060 | 1/235 | NA | NA | NA | NA | NA | NA | flavin adenine dinucleotide binding | CHOD\_STRS0 | GO:0033940 | 0.000124069478908189 | 1/8060 | 1/235 | NA | NA | NA | NA | NA | NA | glucuronoarabinoxylan endo-1,4-beta-xylanase activity | XYNC\_BACSU | GO:0003796 | 0.000620347394540943 | 5/8060 | 1/235 | NA | NA | NA | NA | NA | NA | lysozyme activity | O06496\_CLOPF | GO:0045330 | 0.000372208436724566 | 3/8060 | 1/235 | NA | NA | NA | NA | NA | NA | aspartyl esterase activity | PMEA\_DICD3 | GO:0005537 | 0.000124069478908189 | 1/8060 | 1/235 | NA | NA | NA | NA | NA | NA | mannose binding | XANLY\_BACGL | GO:0016830 | 0.0464019851116625 | 374/8060 | 1/235 | NA | NA | NA | NA | NA | NA | carbon-carbon lyase activity | Q5E1U2\_ALIF1 | GO:0050429 | 0.000124069478908189 | 1/8060 | 1/235 | NA | NA | NA | NA | NA | NA | calcium-dependent phospholipase C activity | PHLC1\_CLOPE | GO:0016811 | 0.021712158808933 | 175/8060 | 1/235 | NA | NA | NA | NA | NA | NA | hydrolase activity, acting on carbon-nitrogen (but not peptide) bonds, in linear amides | A0R5R2\_MYCS2 | GO:0102252 | 0.000124069478908189 | 1/8060 | 1/235 | NA | NA | NA | NA | NA | NA | cellulose 1,4-beta-cellobiosidase activity (reducing end) | GUNS\_ACETH | GO:0102229 | 0.000124069478908189 | 1/8060 | 1/235 | NA | NA | NA | NA | NA | NA | amylopectin maltohydrolase activity | AMYB\_BACCE | GO:0046558 | 0.00086848635235732 | 7/8060 | 1/235 | NA | NA | NA | NA | NA | NA | arabinan endo-1,5-alpha-L-arabinosidase activity | Q65GB9\_BACLD | GO:0018738 | 0.00086848635235732 | 7/8060 | 1/235 | NA | NA | NA | NA | NA | NA | S-formylglutathione hydrolase activity | Q9JZ43\_NEIMB | GO:1990404 | 0.000744416873449132 | 6/8060 | 1/235 | NA | NA | NA | NA | NA | NA | NAD+-protein ADP-ribosyltransferase activity | Q9ADS9\_STAAU | GO:0046589 | 0.000124069478908189 | 1/8060 | 1/235 | NA | NA | NA | NA | NA | NA | ribonuclease T1 activity | RNS3\_KITAU | GO:0033927 | 0.000248138957816377 | 2/8060 | 1/235 | NA | NA | NA | NA | NA | NA | glucan 1,4-alpha-maltohexaosidase activity | AMT6\_BACS7 | GO:0004035 | 0.000372208436724566 | 3/8060 | 1/235 | NA | NA | NA | NA | NA | NA | alkaline phosphatase activity | B4SL31\_STRM5 | GO:0004769 | 0.000620347394540943 | 5/8060 | 1/235 | NA | NA | NA | NA | NA | NA | steroid delta-isomerase activity | CHOD\_STRS0 | GO:0030596 | 0.000372208436724566 | 3/8060 | 1/235 | NA | NA | NA | NA | NA | NA | alpha-L-rhamnosidase activity | RHA78\_STRAW | GO:0022857 | 0.00632754342431762 | 51/8060 | 1/235 | NA | NA | NA | NA | NA | NA | transmembrane transporter activity | BXX\_CLOBO |

Total number of genes: 8060   
Total number of Study genes: 235   
Total number of Study gene GMRG terms (pop non-singletons): 206 (193)   
FDR Threshold *P*-values: [10% = 0.0562], [5% = 0.0281], [1% = 0.00462], [0.5% = 0.00208]   
Genes with GMRG information: 235   
Genes with no GMRG information:   

These are:
