# Supplementary material for: Cellular location shapes quaternary structure of enzymes
Source: Nat Commun. 2024 Oct 1;15:8505. doi: 10.1038/s41467-024-52662-2 (PMC11445431; doi:10.1038/s41467-024-52662-2)
Supplement: Supplementary file 5 — Supplementary Data 2 [file 41467_2024_52662_MOESM5_ESM.zip › GO/Euk_P/output.P.txt.html]

 GeneMerge Output - output.P.txt

### GeneMerge v1.4

### Castillo-Davis, C.I. 2015. GeneMerge v1.4 - post-genomic data analysis

Output File Name: output.P.txt   
Gene Association File: terms.txt   
Description File: descriptions.txt   
Population File: prot\_all.txt   
Study File: prot\_test.txt  
Custom FDR: 0.5%  

|  |  |  |  |  |  |  |  |  |  |  |  |
| --- | --- | --- | --- | --- | --- | --- | --- | --- | --- | --- | --- |
| **GMRG Term** | **Pop Frequency** | **Pop Fraction** | **Study Fraction** | ***P*-value** | **Bon. Corr. *P*-value** | **10% FDR** | **5% FDR** | **1% FDR** | **0.5% FDR** | **Description** | **Contributing genes** |
| GO:0009057 | 0.0893854748603352 | 288/3222 | 200/552 | 2.70478978769922e-98 | 4.72797254889824e-95 | T | T | T | T | macromolecule catabolic process | A0A059U759\_9PEZI | A0A068FT77\_9PEZI | A0A086SY89\_ACRC1 | A0A086T6R4\_ACRC1 | A0A088T0J9\_GEOCN | A0A0J5Q413\_ASPFM | A0A0R4I979\_BRABE | A0A0S2GKZ1\_9APHY | A0A1L6CE30\_9EURO | A0A1L9WG58\_ASPA1 | A0A1S9DRB1\_ASPOZ | A0A2H5BN17\_TALPI | A0A2N1LTK3\_TRIHA | A0A3B6UEQ2\_RHIMI | A0A3G2C3I4\_9EURO | A0A5J6BJN2\_MALCI | A0A6M9BP13\_9EURO | A0A7S6G7I6\_9PEZI | A1E266\_9PEZI | A5AB48\_ASPNC | A6PZ97\_SALSA | A6YRT4\_9PEZI | A8NI40\_COPC7 | A9LI60\_BIOOC | A9ZSX9\_9BRYO | ABFB\_ASPKW | ADPG2\_ARATH | AGAL\_ORYSJ | AMY1\_HORVU | AMY1\_ORYSJ | AMYG\_SACFI | ANAG\_HUMAN | ANG4\_MOUSE | AOAH\_MOUSE | AXE1\_ASPAW | AXE2\_TALPU | AXHA2\_EMENI | B7X9Z0\_COPCI | B7X9Z2\_COPCI | B9TU22\_GADMO | BGALA\_ASPNC | BGALA\_ASPOR | BGALA\_PENSQ | BGL1\_ASPAC | BGLA\_ASPFU | BGLA\_ASPOR | BGLR\_HUMAN | C3VEV9\_PENCN | C7YSL3\_FUSV7 | CARP1\_CANAL | CATD\_RAT | CBHB\_ASPFU | CBHRE\_GEOS1 | CBPN\_HUMAN | CDA\_COLLN | CDA\_EMENI | CEL2A\_PIG | CHI1\_COCPS | CHI2\_HORVU | CHI2\_ORYSJ | CHI33\_TRIHA | CHI42\_TRIHA | CHI4\_CRYJA | CHIA\_HUMAN | CHIC\_ARATH | CHIC\_SECCE | CHIL3\_MOUSE | CHIT\_PUNGR | CHLY\_HEVBR | D0QF43\_9HELO | D9MWI4\_9ASPA | DNAS1\_HUMAN | DNSL3\_HUMAN | DPP2\_HUMAN | E13B\_HORVU | E3VTL0\_9ASPA | E9G5J5\_DAPPU | ECP\_HUMAN | EGLB\_ASPNG | ENDO2\_ARATH | ENG1\_RHIMI | EXG1\_CANAL | EXG1\_YEAST | F0ZJZ1\_DICPU | F1CYZ0\_TALFU | F2Z7L1\_9ANNE | FAEA\_ASPNG | FAEB1\_ASPOR | FAEB2\_ASPOR | FUCO\_HUMAN | G0RVK1\_HYPJQ | G2Q665\_MYCTT | G2QVH2\_THETT | G2X3Y1\_VERDV | G3I1H5\_CRIGR | G3JPF7\_CORMM | G3YAL0\_ASPNA | G3YFQ1\_ASPNA | G9NTY1\_HYPAI | GANA\_EMENI | GANA\_HUMIN | GBA1\_HUMAN | GH7B\_LIMQU | GUN2\_HYPJE | GUN6\_HUMIN | GUN7\_HYPJQ | GUNC\_FUSOX | GUN\_ASPAC | GUN\_CRYAT | GUN\_MYTED | GUX1\_HUMGT | GUX1\_HYPJE | GUX1\_TRIHA | GUX2\_HYPJE | H1AE14\_PHACH | HEXC\_OSTFU | HYAL1\_HUMAN | I2FI81\_EISFE | I3RY46\_TRIHA | IDUA\_HUMAN | INU2\_ASPFI | INUE\_ASPAW | L7SVX1\_RHIMI | LAC1\_MELAO | LGMN\_MOUSE | LYG\_STRCA | M2RAI8\_CERS8 | MAN12\_PENCI | MANA\_ASPNC | MANA\_CRYAT | MANA\_HYPJR | MANA\_MYTED | MANA\_PODAN | MANBA\_MOUSE | NUP1\_PENCI | NUS1\_ASPOR | O00095\_HYPJE | O74705\_ASPNG | O77044\_9NEOP | O81934\_CANEN | P79074\_9AGAR | PELA\_ASPNG | PELB\_ASPNG | PGLR1\_ASPAC | PGLR1\_ASPNG | PGLR\_GIBFU | PGPSA\_DROME | PGRP1\_CAMDR | PHAZ\_TALFU | PLY1\_JUNAS | PME\_DAUCA | PME\_SITOR | PPT1\_BOVIN | PPT1\_HUMAN | Q02321\_PHACH | Q0KFV0\_SOLLC | Q12715\_HYPJE | Q2U8V9\_ASPOR | Q43576\_TOBAC | Q4AE59\_OSTFU | Q4WP32\_ASPFU | Q55FE6\_DICDI | Q6VAY1\_9PEZI | Q6WSR8\_PICAB | Q7LHI2\_PHACH | Q7LIJ0\_PHACH | Q7RWP2\_NEUCR | Q86RS6\_MANSE | Q8H0C9\_VIGUN | Q8J0K6\_MELAO | Q8J0K8\_MELAO | Q8NJY6\_9HYPO | Q8TFL9\_TALEM | Q8TG26\_THEAU | Q8TGI8\_TALEM | Q92458\_HYPJE | Q9FUH3\_VIGUS | RGLA\_ASPAC | RHGA\_ASPAC | RNAS6\_HUMAN | RNLE\_SOLLC | RNT2\_HUMAN | S7Q6I2\_GLOTA | S7ZIW0\_PENO1 | TPP1\_HUMAN | W4KMP1\_HETIT | W8P1L2\_TALEM | W8VR85\_TALPI | X0M5X0\_FUSOX | XGHA\_ASPTU | XYN1\_HYPJR | XYN2\_HYPJR | XYN3\_ASPKW | XYN3\_HYPJQ | XYNA\_FUSO4 | XYNA\_PENSI | XYNA\_THEAU | XYNA\_THELA | XYNC\_ASPNC | XYND\_EMENI |
 GO:0000272 | 0.0580384854127871 | 187/3222 | 155/552 | 3.93312678219821e-94 | 6.87510561528247e-91 | T | T | T | T | polysaccharide catabolic process | A0A059U759\_9PEZI | A0A068FT77\_9PEZI | A0A086SY89\_ACRC1 | A0A086T6R4\_ACRC1 | A0A088T0J9\_GEOCN | A0A0J5Q413\_ASPFM | A0A0S2GKZ1\_9APHY | A0A1L6CE30\_9EURO | A0A1L9WG58\_ASPA1 | A0A1S9DRB1\_ASPOZ | A0A2H5BN17\_TALPI | A0A2N1LTK3\_TRIHA | A0A3B6UEQ2\_RHIMI | A0A3G2C3I4\_9EURO | A0A5J6BJN2\_MALCI | A0A6M9BP13\_9EURO | A1E266\_9PEZI | A5AB48\_ASPNC | A6YRT4\_9PEZI | A8NI40\_COPC7 | A9LI60\_BIOOC | ABFB\_ASPKW | ADPG2\_ARATH | AGAL\_ORYSJ | AMY1\_HORVU | AMY1\_ORYSJ | AMYG\_SACFI | AXE1\_ASPAW | AXE2\_TALPU | AXHA2\_EMENI | B7X9Z0\_COPCI | B7X9Z2\_COPCI | BGALA\_ASPNC | BGALA\_ASPOR | BGALA\_PENSQ | BGL1\_ASPAC | BGLA\_ASPFU | BGLA\_ASPOR | C3VEV9\_PENCN | C7YSL3\_FUSV7 | CBHB\_ASPFU | CBHRE\_GEOS1 | CDA\_COLLN | CDA\_EMENI | CHI1\_COCPS | CHI2\_HORVU | CHI2\_ORYSJ | CHI33\_TRIHA | CHI42\_TRIHA | CHI4\_CRYJA | CHIA\_HUMAN | CHIC\_ARATH | CHIC\_SECCE | CHIL3\_MOUSE | CHIT\_PUNGR | CHLY\_HEVBR | D0QF43\_9HELO | D9MWI4\_9ASPA | E13B\_HORVU | E3VTL0\_9ASPA | E9G5J5\_DAPPU | EGLB\_ASPNG | ENG1\_RHIMI | EXG1\_CANAL | EXG1\_YEAST | F0ZJZ1\_DICPU | F1CYZ0\_TALFU | F2Z7L1\_9ANNE | FAEA\_ASPNG | FAEB1\_ASPOR | FAEB2\_ASPOR | G0RVK1\_HYPJQ | G2Q665\_MYCTT | G2QVH2\_THETT | G2X3Y1\_VERDV | G3JPF7\_CORMM | G3YAL0\_ASPNA | G3YFQ1\_ASPNA | G9NTY1\_HYPAI | GANA\_EMENI | GH7B\_LIMQU | GUN2\_HYPJE | GUN6\_HUMIN | GUN7\_HYPJQ | GUNC\_FUSOX | GUN\_ASPAC | GUN\_CRYAT | GUN\_MYTED | GUX1\_HUMGT | GUX1\_HYPJE | GUX1\_TRIHA | GUX2\_HYPJE | H1AE14\_PHACH | HEXC\_OSTFU | I2FI81\_EISFE | I3RY46\_TRIHA | INU2\_ASPFI | INUE\_ASPAW | L7SVX1\_RHIMI | LAC1\_MELAO | M2RAI8\_CERS8 | MANA\_ASPNC | MANA\_CRYAT | MANA\_HYPJR | MANA\_MYTED | MANA\_PODAN | O00095\_HYPJE | O74705\_ASPNG | O77044\_9NEOP | P79074\_9AGAR | PELA\_ASPNG | PELB\_ASPNG | PGLR1\_ASPAC | PGLR1\_ASPNG | PGLR\_GIBFU | PHAZ\_TALFU | PLY1\_JUNAS | PME\_DAUCA | PME\_SITOR | Q02321\_PHACH | Q12715\_HYPJE | Q2U8V9\_ASPOR | Q4WP32\_ASPFU | Q55FE6\_DICDI | Q6VAY1\_9PEZI | Q7LHI2\_PHACH | Q7LIJ0\_PHACH | Q7RWP2\_NEUCR | Q8J0K6\_MELAO | Q8J0K8\_MELAO | Q8NJY6\_9HYPO | Q8TFL9\_TALEM | Q8TG26\_THEAU | Q8TGI8\_TALEM | Q92458\_HYPJE | Q9FUH3\_VIGUS | RGLA\_ASPAC | RHGA\_ASPAC | S7Q6I2\_GLOTA | S7ZIW0\_PENO1 | W4KMP1\_HETIT | W8P1L2\_TALEM | W8VR85\_TALPI | X0M5X0\_FUSOX | XGHA\_ASPTU | XYN1\_HYPJR | XYN2\_HYPJR | XYN3\_ASPKW | XYN3\_HYPJQ | XYNA\_FUSO4 | XYNA\_PENSI | XYNA\_THEAU | XYNA\_THELA | XYNC\_ASPNC | XYND\_EMENI | GO:0005976 | 0.0648665425201738 | 209/3222 | 158/552 | 9.66054700251494e-85 | 1.68866361603961e-81 | T | T | T | T | polysaccharide metabolic process | A0A059U759\_9PEZI | A0A068FT77\_9PEZI | A0A086SY89\_ACRC1 | A0A086T6R4\_ACRC1 | A0A088T0J9\_GEOCN | A0A0J5Q413\_ASPFM | A0A0S2GKZ1\_9APHY | A0A1L6CE30\_9EURO | A0A1L9WG58\_ASPA1 | A0A1S9DRB1\_ASPOZ | A0A2H5BN17\_TALPI | A0A2N1LTK3\_TRIHA | A0A3B6UEQ2\_RHIMI | A0A3G2C3I4\_9EURO | A0A3G4RHU4\_9PEZI | A0A5J6BJN2\_MALCI | A0A6M9BP13\_9EURO | A1E266\_9PEZI | A5AB48\_ASPNC | A6YRT4\_9PEZI | A8NI40\_COPC7 | A9LI60\_BIOOC | ABFB\_ASPKW | ADPG2\_ARATH | AGAL\_ORYSJ | AMY1\_HORVU | AMY1\_ORYSJ | AMYG\_SACFI | AXE1\_ASPAW | AXE2\_TALPU | AXHA2\_EMENI | B7X9Z0\_COPCI | B7X9Z2\_COPCI | BGALA\_ASPNC | BGALA\_ASPOR | BGALA\_PENSQ | BGL1\_ASPAC | BGLA\_ASPFU | BGLA\_ASPOR | C3VEV9\_PENCN | C7YSL3\_FUSV7 | CBHB\_ASPFU | CBHRE\_GEOS1 | CDA\_COLLN | CDA\_EMENI | CHI1\_COCPS | CHI2\_HORVU | CHI2\_ORYSJ | CHI33\_TRIHA | CHI42\_TRIHA | CHI4\_CRYJA | CHIA\_HUMAN | CHIC\_ARATH | CHIC\_SECCE | CHIL3\_MOUSE | CHIT\_PUNGR | CHLY\_HEVBR | D0QF43\_9HELO | D9MWI4\_9ASPA | E13B\_HORVU | E3VTL0\_9ASPA | E9G5J5\_DAPPU | EGLB\_ASPNG | ENG1\_RHIMI | EXG1\_CANAL | EXG1\_YEAST | F0ZJZ1\_DICPU | F1CYZ0\_TALFU | F2Z7L1\_9ANNE | FAEA\_ASPNG | FAEB1\_ASPOR | FAEB2\_ASPOR | G0RVK1\_HYPJQ | G2Q665\_MYCTT | G2QVH2\_THETT | G2X3Y1\_VERDV | G3JPF7\_CORMM | G3YAL0\_ASPNA | G3YFQ1\_ASPNA | G9NTY1\_HYPAI | GANA\_EMENI | GH7B\_LIMQU | GUN2\_HYPJE | GUN6\_HUMIN | GUN7\_HYPJQ | GUNC\_FUSOX | GUN\_ASPAC | GUN\_CRYAT | GUN\_MYTED | GUX1\_HUMGT | GUX1\_HYPJE | GUX1\_TRIHA | GUX2\_HYPJE | H1AE14\_PHACH | HEXC\_OSTFU | I2FI81\_EISFE | I3RY46\_TRIHA | INU2\_ASPFI | INUE\_ASPAW | L7SVX1\_RHIMI | LAC1\_MELAO | M2RAI8\_CERS8 | MANA\_ASPNC | MANA\_CRYAT | MANA\_HYPJR | MANA\_MYTED | MANA\_PODAN | O00095\_HYPJE | O74705\_ASPNG | O77044\_9NEOP | P79074\_9AGAR | PELA\_ASPNG | PELB\_ASPNG | PGLR1\_ASPAC | PGLR1\_ASPNG | PGLR\_GIBFU | PHAZ\_TALFU | PLY1\_JUNAS | PME\_DAUCA | PME\_SITOR | Q02321\_PHACH | Q07524\_TROMA | Q12715\_HYPJE | Q2U8V9\_ASPOR | Q4WP32\_ASPFU | Q55FE6\_DICDI | Q6VAY1\_9PEZI | Q7LHI2\_PHACH | Q7LIJ0\_PHACH | Q7RWP2\_NEUCR | Q8J0K6\_MELAO | Q8J0K8\_MELAO | Q8NJY6\_9HYPO | Q8TFL9\_TALEM | Q8TG26\_THEAU | Q8TGI8\_TALEM | Q92458\_HYPJE | Q9FUH3\_VIGUS | RGLA\_ASPAC | RHGA\_ASPAC | S7Q6I2\_GLOTA | S7ZIW0\_PENO1 | W4KMP1\_HETIT | W8P1L2\_TALEM | W8VR85\_TALPI | X0M5X0\_FUSOX | XGHA\_ASPTU | XTH34\_POPPZ | XYN1\_HYPJR | XYN2\_HYPJR | XYN3\_ASPKW | XYN3\_HYPJQ | XYNA\_FUSO4 | XYNA\_PENSI | XYNA\_THEAU | XYNA\_THELA | XYNC\_ASPNC | XYND\_EMENI | GO:0043170 | 0.252017380509001 | 812/3222 | 281/552 | 2.1595667723968e-47 | 3.77492271814961e-44 | T | T | T | T | macromolecule metabolic process | A0A059U759\_9PEZI | A0A068FT77\_9PEZI | A0A086SY89\_ACRC1 | A0A086T6R4\_ACRC1 | A0A088T0J9\_GEOCN | A0A0J5Q413\_ASPFM | A0A0R3QSA7\_9BILA | A0A0R4I979\_BRABE | A0A0S2GKZ1\_9APHY | A0A1L6CE30\_9EURO | A0A1L9WG58\_ASPA1 | A0A1S9DRB1\_ASPOZ | A0A2H5BN17\_TALPI | A0A2N1LTK3\_TRIHA | A0A3B6UEQ2\_RHIMI | A0A3G2C3I4\_9EURO | A0A3G4RHU4\_9PEZI | A0A3S5H5N2\_LEIDO | A0A5J6BJN2\_MALCI | A0A6M9BP13\_9EURO | A0A6P6YAT6\_DERPT | A0A7S6G7I6\_9PEZI | A0NFU8\_ANOGA | A1E266\_9PEZI | A4GX63\_TOXGO | A5AB48\_ASPNC | A6PZ97\_SALSA | A6YRT4\_9PEZI | A8NI40\_COPC7 | A9LI60\_BIOOC | A9ZSX9\_9BRYO | ABFB\_ASPKW | ADPG2\_ARATH | AGAL\_ORYSJ | AMY1\_HORVU | AMY1\_ORYSJ | AMYG\_SACFI | ANAG\_HUMAN | ANG1\_BOVIN | ANG2\_MOUSE | ANG3\_MOUSE | ANG4\_MOUSE | ANGI\_MOUSE | AOAH\_MOUSE | AXE1\_ASPAW | AXE2\_TALPU | AXHA2\_EMENI | B4F320\_LIMPO | B7X9Z0\_COPCI | B7X9Z2\_COPCI | B9TU22\_GADMO | BGALA\_ASPNC | BGALA\_ASPOR | BGALA\_PENSQ | BGL1\_ASPAC | BGLA\_ASPFU | BGLA\_ASPOR | BGLR\_HUMAN | C3VEV9\_PENCN | C7YSL3\_FUSV7 | CARP1\_CANAL | CARP2\_CANAX | CARP\_RHIPU | CATD\_RAT | CATH\_HUMAN | CATLL\_FASHE | CBHB\_ASPFU | CBHRE\_GEOS1 | CBPA1\_PIG | CBPD\_LOPSP | CBPN\_HUMAN | CDA\_COLLN | CDA\_EMENI | CEL2A\_PIG | CFAD\_MOUSE | CHI1\_COCPS | CHI2\_HORVU | CHI2\_ORYSJ | CHI33\_TRIHA | CHI42\_TRIHA | CHI4\_CRYJA | CHIA\_HUMAN | CHIC\_ARATH | CHIC\_SECCE | CHIL3\_MOUSE | CHIT\_PUNGR | CHLY\_HEVBR | CHYM\_CAMDR | COGS\_HYPLI | CUCM1\_CUCME | CYP5\_CAEEL | CYSP\_BLOTA | D0QF43\_9HELO | D6XHE1\_TRYB2 | D9MWI4\_9ASPA | DDN1\_BOVIN | DNAS1\_HUMAN | DNSL3\_HUMAN | DPP2\_HUMAN | E13B\_HORVU | E3VTL0\_9ASPA | E9G5J5\_DAPPU | ECP\_HUMAN | EGFB2\_MOUSE | EGLB\_ASPNG | ENDO2\_ARATH | ENG1\_RHIMI | ERVB\_TABDI | EXG1\_CANAL | EXG1\_YEAST | F0ZJZ1\_DICPU | F1CYZ0\_TALFU | F2Z7L1\_9ANNE | FAEA\_ASPNG | FAEB1\_ASPOR | FAEB2\_ASPOR | FUCO\_HUMAN | G0RVK1\_HYPJQ | G2Q665\_MYCTT | G2QVH2\_THETT | G2X3Y1\_VERDV | G3I1H5\_CRIGR | G3JPF7\_CORMM | G3YAL0\_ASPNA | G3YFQ1\_ASPNA | G9NTY1\_HYPAI | GANA\_EMENI | GANA\_HUMIN | GBA1\_HUMAN | GH7B\_LIMQU | GRAA\_HUMAN | GRAC\_MOUSE | GRAK\_HUMAN | GRASS\_DROME | GUN2\_HYPJE | GUN6\_HUMIN | GUN7\_HYPJQ | GUNC\_FUSOX | GUN\_ASPAC | GUN\_CRYAT | GUN\_MYTED | GUX1\_HUMGT | GUX1\_HYPJE | GUX1\_TRIHA | GUX2\_HYPJE | H1AE14\_PHACH | HE12\_DANRE | HEXC\_OSTFU | HS3S1\_MOUSE | HYAL1\_HUMAN | I2FI81\_EISFE | I3RY46\_TRIHA | IDUA\_HUMAN | INU2\_ASPFI | INUE\_ASPAW | J7LCB0\_DEIAC | KLK10\_HUMAN | KLK1\_HUMAN | KLK2\_HORSE | KLK2\_HUMAN | KLK7\_HUMAN | KLK7\_MOUSE | KLK8\_MOUSE | L7SVX1\_RHIMI | LAC1\_MELAO | LAPA\_ASPOR | LGMN\_MOUSE | LICH\_HUMAN | LYG\_STRCA | M2RAI8\_CERS8 | MAN12\_PENCI | MANA\_ASPNC | MANA\_CRYAT | MANA\_HYPJR | MANA\_MYTED | MANA\_PODAN | MANBA\_MOUSE | MCPT2\_RAT | MMP1\_PIG | NUP1\_PENCI | NUS1\_ASPOR | O00095\_HYPJE | O74705\_ASPNG | O77044\_9NEOP | O81226\_CARPA | O81934\_CANEN | O97389\_HELAM | OFUT1\_CAEEL | P79074\_9AGAR | PCP\_HUMAN | PELA\_ASPNG | PELB\_ASPNG | PEPA\_ASPPH | PGLR1\_ASPAC | PGLR1\_ASPNG | PGLR\_GIBFU | PGPSA\_DROME | PGRP1\_CAMDR | PHAZ\_TALFU | PLY1\_JUNAS | PME\_DAUCA | PME\_SITOR | PPAF1\_HOLDI | PPT1\_BOVIN | PPT1\_HUMAN | PRS57\_HUMAN | PRTN3\_HUMAN | PTGDS\_HUMAN | PTGDS\_MOUSE | Q02321\_PHACH | Q06AK3\_TOXGO | Q07524\_TROMA | Q0KFV0\_SOLLC | Q12715\_HYPJE | Q2U8V9\_ASPOR | Q43576\_TOBAC | Q4AE59\_OSTFU | Q4WP32\_ASPFU | Q55FE6\_DICDI | Q5B038\_EMENI | Q5WRG2\_RAT | Q69G21\_TENMO | Q6R7Z5\_9TRYP | Q6VAY1\_9PEZI | Q6WSR8\_PICAB | Q7LHI2\_PHACH | Q7LIJ0\_PHACH | Q7RWP2\_NEUCR | Q7YXL2\_TENMO | Q86RS6\_MANSE | Q8H0C9\_VIGUN | Q8J0K6\_MELAO | Q8J0K8\_MELAO | Q8NJY6\_9HYPO | Q8TFL9\_TALEM | Q8TG26\_THEAU | Q8TGI8\_TALEM | Q92458\_HYPJE | Q9FUH3\_VIGUS | QPCT1\_DROME | QPCT2\_DROME | QPCT\_IXOSC | QPCT\_MOUSE | RENI\_RAT | RGLA\_ASPAC | RHGA\_ASPAC | RNAS6\_HUMAN | RNLE\_SOLLC | RNT2\_HUMAN | S7Q6I2\_GLOTA | S7ZIW0\_PENO1 | TPP1\_HUMAN | TRFL\_BUBBU | TRFL\_HORSE | TRY1\_GADMO | TRY3\_SALSA | TRYB2\_HUMAN | VM11\_BOTMO | VM12\_CROAD | VM1A3\_DEIAC | VM1BI\_BOTMO | VM1T1\_PROMU | VM1T2\_PROFL | VSPP\_DEIAC | VSPSX\_GLOSA | W4KMP1\_HETIT | W8P1L2\_TALEM | W8VR85\_TALPI | X0M5X0\_FUSOX | XGHA\_ASPTU | XTH34\_POPPZ | XYN1\_HYPJR | XYN2\_HYPJR | XYN3\_ASPKW | XYN3\_HYPJQ | XYNA\_FUSO4 | XYNA\_PENSI | XYNA\_THEAU | XYNA\_THELA | XYNC\_ASPNC | XYND\_EMENI | GO:1901575 | 0.339851024208566 | 1095/3222 | 335/552 | 1.29492045547697e-45 | 2.26352095617375e-42 | T | T | T | T | organic substance catabolic process | A0A059U759\_9PEZI | A0A068FT77\_9PEZI | A0A086SY89\_ACRC1 | A0A086T6R4\_ACRC1 | A0A087WNH2\_FICBE | A0A088T0J9\_GEOCN | A0A0A0Y4H8\_TRAFO | A0A0J5Q413\_ASPFM | A0A0R4I979\_BRABE | A0A0S2GKZ1\_9APHY | A0A1L6CE30\_9EURO | A0A1L8D5Z7\_BOTAT | A0A1L9WG58\_ASPA1 | A0A1S4NYF8\_PANVG | A0A1S9DRB1\_ASPOZ | A0A2H5BN17\_TALPI | A0A2N1LTK3\_TRIHA | A0A2Z4HIN9\_9EURO | A0A3B6UEQ2\_RHIMI | A0A3G2C3I4\_9EURO | A0A3L6SKP5\_PANMI | A0A5J6BJN2\_MALCI | A0A6M9BP13\_9EURO | A0A7S6G7I6\_9PEZI | A1E266\_9PEZI | A1HA\_LOXIN | A1HB2\_LOXIN | A311\_LOXLA | A5AB48\_ASPNC | A6PZ97\_SALSA | A6YRT4\_9PEZI | A8NI40\_COPC7 | A9LI60\_BIOOC | A9ZSX9\_9BRYO | ABFB\_ASPKW | ADA2\_HUMAN | ADPG2\_ARATH | AGAL\_HUMAN | AGAL\_ORYSJ | AMY1\_HORVU | AMY1\_ORYSJ | AMYA1\_ASPOR | AMYG\_SACFI | ANAG\_HUMAN | ANG4\_MOUSE | AOAH\_MOUSE | APO1\_CYCAE | ASM3A\_HUMAN | ASM3A\_MOUSE | AXE1\_ASPAW | AXE2\_TALPU | AXHA2\_EMENI | B7X9Z0\_COPCI | B7X9Z2\_COPCI | B9TU22\_GADMO | BGALA\_ASPNC | BGALA\_ASPOR | BGALA\_PENSQ | BGL1\_ASPAC | BGLA\_ASPFU | BGLA\_ASPOR | BGLR\_HUMAN | C3VEV9\_PENCN | C7YSL3\_FUSV7 | CARP1\_CANAL | CAT3\_NEUCR | CATD\_RAT | CATH\_HUMAN | CBHB\_ASPFU | CBHRE\_GEOS1 | CBPN\_HUMAN | CDA\_COLLN | CDA\_EMENI | CEL2A\_PIG | CHI1\_COCPS | CHI2\_HORVU | CHI2\_ORYSJ | CHI33\_TRIHA | CHI42\_TRIHA | CHI4\_CRYJA | CHIA\_HUMAN | CHIC\_ARATH | CHIC\_SECCE | CHIL3\_MOUSE | CHIT\_PUNGR | CHLY\_HEVBR | CUTI1\_ASPOR | D0QF43\_9HELO | D1MPT2\_ROYRE | D9MWI4\_9ASPA | DNAS1\_HUMAN | DNSL3\_HUMAN | DOPO\_HUMAN | DPP2\_HUMAN | E0A7J0\_YARLL | E13B\_HORVU | E3VTL0\_9ASPA | E9G5J5\_DAPPU | ECP\_HUMAN | EGLB\_ASPNG | ENDO2\_ARATH | ENG1\_RHIMI | ENPP2\_HUMAN | ENPP2\_RAT | EST6\_DROME | EXG1\_CANAL | EXG1\_YEAST | F0ZJZ1\_DICPU | F1CYZ0\_TALFU | F2Z7L1\_9ANNE | FAEA\_ASPNG | FAEB1\_ASPOR | FAEB2\_ASPOR | FUCO\_HUMAN | G0RVK1\_HYPJQ | G2Q665\_MYCTT | G2QVH2\_THETT | G2X3Y1\_VERDV | G3I1H5\_CRIGR | G3JPF7\_CORMM | G3YAL0\_ASPNA | G3YFQ1\_ASPNA | G9NTY1\_HYPAI | GANA\_EMENI | GANA\_HUMIN | GBA1\_HUMAN | GCE2\_MYCTT | GCE\_CERUI | GCE\_HYPJQ | GH7B\_LIMQU | GPX3\_HUMAN | GUN2\_HYPJE | GUN6\_HUMIN | GUN7\_HYPJQ | GUNC\_FUSOX | GUN\_ASPAC | GUN\_CRYAT | GUN\_MYTED | GUX1\_HUMGT | GUX1\_HYPJE | GUX1\_TRIHA | GUX2\_HYPJE | H1AE14\_PHACH | HEXC\_OSTFU | HYAL1\_HUMAN | I1SB18\_VIPAE | I2FI81\_EISFE | I3RY46\_TRIHA | IDUA\_HUMAN | INU2\_ASPFI | INUE\_ASPAW | K7N5L9\_RAPSA | K9L8F3\_MALCI | KATG2\_MAGO7 | L7SVX1\_RHIMI | LAC1\_MELAO | LAC1\_TRAMX | LAC2\_TRAVE | LGMN\_MOUSE | LICH\_HUMAN | LIG2\_PHACH | LIG4\_PHACH | LIG8\_PHACH | LIP1\_DIURU | LIP2\_DIURU | LIP2\_GEOCN | LIP3\_DIURU | LIPA\_MOEAP | LIPB\_PSEA2 | LIPG\_CANLF | LIPG\_HUMAN | LIPP\_HORSE | LIPR1\_CANLF | LIPR1\_HUMAN | LIPR2\_HUMAN | LIPR2\_RAT | LIP\_THELA | LYG\_STRCA | M2RAI8\_CERS8 | M9TI89\_RHIPU | MAN12\_PENCI | MANA\_ASPNC | MANA\_CRYAT | MANA\_HYPJR | MANA\_MYTED | MANA\_PODAN | MANBA\_MOUSE | MDLA\_PENCA | MDLA\_PENCY | NAGAB\_CHICK | NAGAB\_HUMAN | NUP1\_PENCI | NUS1\_ASPOR | O00095\_HYPJE | O22443\_SOYBN | O74705\_ASPNG | O77044\_9NEOP | O81934\_CANEN | P78583\_ASPOZ | P79074\_9AGAR | PA21B\_BOVIN | PA21B\_PIG | PA2A1\_BUNCE | PA2A1\_ECHCA | PA2A1\_NAJAT | PA2A1\_OPHHA | PA2A2\_NAJNA | PA2A2\_OPHHA | PA2A2\_TROCA | PA2A4\_NAJSG | PA2A5\_TRIST | PA2A7\_GLOHA | PA2A\_BOTJR | PA2A\_CROAT | PA2A\_DEIAC | PA2A\_GLOHA | PA2A\_NAJAT | PA2B1\_AGKPI | PA2B2\_BOTJR | PA2B2\_PROFL | PA2B3\_BOTAS | PA2B3\_BUNCE | PA2B5\_BUNCE | PA2B5\_NOTSC | PA2BA\_VIPAA | PA2BB\_GLOHA | PA2BB\_PSEAU | PA2BC\_VIPAA | PA2BD\_CRODU | PA2B\_BUNCE | PA2B\_NOTSC | PA2GA\_HUMAN | PA2GE\_HUMAN | PA2GX\_HUMAN | PA2H1\_AGKCL | PA2H1\_BOTBZ | PA2H1\_BOTJR | PA2H1\_BOTMO | PA2H1\_BOTPI | PA2H2\_BOTAS | PA2H2\_BOTMO | PA2H2\_BOTPI | PA2H2\_CERGO | PA2H3\_BOTPI | PA2HB\_AGKPI | PA2HB\_OXYSC | PA2HH\_TRIST | PA2HS\_ECHCA | PA2H\_BOTPA | PA2H\_DEIAC | PA2H\_PROMB | PA2N\_GLOHA | PA2\_APIME | PAG15\_HUMAN | PELA\_ASPNG | PELB\_ASPNG | PEM1\_PHACH | PER1A\_ARMRU | PER1\_ARAHY | PER1\_SORBI | PER53\_ARATH | PER59\_ARATH | PERL\_BOVIN | PERL\_BUBBU | PERL\_CAPHI | PER\_ARTRA | PER\_COPCI | PGLR1\_ASPAC | PGLR1\_ASPNG | PGLR\_GIBFU | PGPSA\_DROME | PGRP1\_CAMDR | PHAZ\_TALFU | PLA22\_ORYSJ | PLY1\_JUNAS | PME\_DAUCA | PME\_SITOR | POXA\_DICDI | PPT1\_BOVIN | PPT1\_HUMAN | Q02321\_PHACH | Q0KFV0\_SOLLC | Q12715\_HYPJE | Q2U8V9\_ASPOR | Q40069\_HORVU | Q43576\_TOBAC | Q4AE59\_OSTFU | Q4WP32\_ASPFU | Q55FE6\_DICDI | Q60FD2\_9APHY | Q6VAY1\_9PEZI | Q6WSR8\_PICAB | Q7LHI2\_PHACH | Q7LIJ0\_PHACH | Q7RWP2\_NEUCR | Q86RS6\_MANSE | Q8H0C9\_VIGUN | Q8J0K6\_MELAO | Q8J0K8\_MELAO | Q8NJY6\_9HYPO | Q8TFL9\_TALEM | Q8TG26\_THEAU | Q8TGI8\_TALEM | Q92458\_HYPJE | Q95KP4\_HORSE | Q9FUH3\_VIGUS | Q9P8F7\_YARLL | RGLA\_ASPAC | RHGA\_ASPAC | RNAS6\_HUMAN | RNLE\_SOLLC | RNT2\_HUMAN | S7Q6I2\_GLOTA | S7ZIW0\_PENO1 | SIA\_ASPFU | TPP1\_HUMAN | V5NTD\_NAJAT | VPL1\_PLEER | VPL2\_PLEER | W4KMP1\_HETIT | W6Q990\_PENRF | W8P1L2\_TALEM | W8VR85\_TALPI | X0M5X0\_FUSOX | XGHA\_ASPTU | XYN1\_HYPJR | XYN2\_HYPJR | XYN3\_ASPKW | XYN3\_HYPJQ | XYNA\_FUSO4 | XYNA\_PENSI | XYNA\_THEAU | XYNA\_THELA | XYNC\_ASPNC | XYND\_EMENI | GO:0009056 | 0.35195530726257 | 1134/3222 | 338/552 | 4.37017476828217e-43 | 7.63906549495723e-40 | T | T | T | T | catabolic process | A0A059U759\_9PEZI | A0A068FT77\_9PEZI | A0A086SY89\_ACRC1 | A0A086T6R4\_ACRC1 | A0A087WNH2\_FICBE | A0A088T0J9\_GEOCN | A0A0A0Y4H8\_TRAFO | A0A0J5Q413\_ASPFM | A0A0R4I979\_BRABE | A0A0S2GKZ1\_9APHY | A0A1L6CE30\_9EURO | A0A1L8D5Z7\_BOTAT | A0A1L9WG58\_ASPA1 | A0A1S4NYF8\_PANVG | A0A1S9DRB1\_ASPOZ | A0A2H5BN17\_TALPI | A0A2N1LTK3\_TRIHA | A0A2Z4HIN9\_9EURO | A0A3B6UEQ2\_RHIMI | A0A3G2C3I4\_9EURO | A0A3L6SKP5\_PANMI | A0A5J6BJN2\_MALCI | A0A6M9BP13\_9EURO | A0A7S6G7I6\_9PEZI | A1E266\_9PEZI | A1HA\_LOXIN | A1HB2\_LOXIN | A311\_LOXLA | A5AB48\_ASPNC | A6PZ97\_SALSA | A6YRT4\_9PEZI | A8NI40\_COPC7 | A9LI60\_BIOOC | A9ZSX9\_9BRYO | ABFB\_ASPKW | ADA2\_HUMAN | ADPG2\_ARATH | AGAL\_HUMAN | AGAL\_ORYSJ | AMY1\_HORVU | AMY1\_ORYSJ | AMYA1\_ASPOR | AMYG\_SACFI | ANAG\_HUMAN | ANG4\_MOUSE | AOAH\_MOUSE | APO1\_CYCAE | ASM3A\_HUMAN | ASM3A\_MOUSE | AXE1\_ASPAW | AXE2\_TALPU | AXHA2\_EMENI | B7X9Z0\_COPCI | B7X9Z2\_COPCI | B9TU22\_GADMO | BGALA\_ASPNC | BGALA\_ASPOR | BGALA\_PENSQ | BGL1\_ASPAC | BGLA\_ASPFU | BGLA\_ASPOR | BGLR\_HUMAN | C3VEV9\_PENCN | C7YSL3\_FUSV7 | CARP1\_CANAL | CAT3\_NEUCR | CATD\_RAT | CATH\_HUMAN | CBHB\_ASPFU | CBHRE\_GEOS1 | CBPN\_HUMAN | CDA\_COLLN | CDA\_EMENI | CEL2A\_PIG | CHI1\_COCPS | CHI2\_HORVU | CHI2\_ORYSJ | CHI33\_TRIHA | CHI42\_TRIHA | CHI4\_CRYJA | CHIA\_HUMAN | CHIC\_ARATH | CHIC\_SECCE | CHIL3\_MOUSE | CHIT\_PUNGR | CHLY\_HEVBR | COGS\_HYPLI | CUTI1\_ASPOR | D0QF43\_9HELO | D1MPT2\_ROYRE | D9MWI4\_9ASPA | DNAS1\_HUMAN | DNSL3\_HUMAN | DOPO\_HUMAN | DPP2\_HUMAN | E0A7J0\_YARLL | E13B\_HORVU | E3VTL0\_9ASPA | E9G5J5\_DAPPU | ECP\_HUMAN | EGLB\_ASPNG | ENDO2\_ARATH | ENG1\_RHIMI | ENPP2\_HUMAN | ENPP2\_RAT | EST6\_DROME | EXG1\_CANAL | EXG1\_YEAST | F0ZJZ1\_DICPU | F1CYZ0\_TALFU | F2Z7L1\_9ANNE | FAEA\_ASPNG | FAEB1\_ASPOR | FAEB2\_ASPOR | FUCO\_HUMAN | G0RVK1\_HYPJQ | G2Q665\_MYCTT | G2QVH2\_THETT | G2X3Y1\_VERDV | G3I1H5\_CRIGR | G3JPF7\_CORMM | G3YAL0\_ASPNA | G3YFQ1\_ASPNA | G9NTY1\_HYPAI | GANA\_EMENI | GANA\_HUMIN | GBA1\_HUMAN | GCE2\_MYCTT | GCE\_CERUI | GCE\_HYPJQ | GH7B\_LIMQU | GPX3\_HUMAN | GUN2\_HYPJE | GUN6\_HUMIN | GUN7\_HYPJQ | GUNC\_FUSOX | GUN\_ASPAC | GUN\_CRYAT | GUN\_MYTED | GUX1\_HUMGT | GUX1\_HYPJE | GUX1\_TRIHA | GUX2\_HYPJE | H1AE14\_PHACH | HEXC\_OSTFU | HYAL1\_HUMAN | I1SB18\_VIPAE | I2FI81\_EISFE | I3RY46\_TRIHA | IDUA\_HUMAN | INU2\_ASPFI | INUE\_ASPAW | K7N5L9\_RAPSA | K9L8F3\_MALCI | KATG2\_MAGO7 | L7SVX1\_RHIMI | LAC1\_MELAO | LAC1\_TRAMX | LAC2\_TRAVE | LGMN\_MOUSE | LICH\_HUMAN | LIG2\_PHACH | LIG4\_PHACH | LIG8\_PHACH | LIP1\_DIURU | LIP2\_DIURU | LIP2\_GEOCN | LIP3\_DIURU | LIPA\_MOEAP | LIPB\_PSEA2 | LIPG\_CANLF | LIPG\_HUMAN | LIPP\_HORSE | LIPR1\_CANLF | LIPR1\_HUMAN | LIPR2\_HUMAN | LIPR2\_RAT | LIP\_THELA | LYG\_STRCA | M2RAI8\_CERS8 | M9TI89\_RHIPU | MAN12\_PENCI | MANA\_ASPNC | MANA\_CRYAT | MANA\_HYPJR | MANA\_MYTED | MANA\_PODAN | MANBA\_MOUSE | MDLA\_PENCA | MDLA\_PENCY | MMP1\_PIG | NAGAB\_CHICK | NAGAB\_HUMAN | NUP1\_PENCI | NUS1\_ASPOR | O00095\_HYPJE | O22443\_SOYBN | O74705\_ASPNG | O77044\_9NEOP | O81934\_CANEN | P78583\_ASPOZ | P79074\_9AGAR | PA21B\_BOVIN | PA21B\_PIG | PA2A1\_BUNCE | PA2A1\_ECHCA | PA2A1\_NAJAT | PA2A1\_OPHHA | PA2A2\_NAJNA | PA2A2\_OPHHA | PA2A2\_TROCA | PA2A4\_NAJSG | PA2A5\_TRIST | PA2A7\_GLOHA | PA2A\_BOTJR | PA2A\_CROAT | PA2A\_DEIAC | PA2A\_GLOHA | PA2A\_NAJAT | PA2B1\_AGKPI | PA2B2\_BOTJR | PA2B2\_PROFL | PA2B3\_BOTAS | PA2B3\_BUNCE | PA2B5\_BUNCE | PA2B5\_NOTSC | PA2BA\_VIPAA | PA2BB\_GLOHA | PA2BB\_PSEAU | PA2BC\_VIPAA | PA2BD\_CRODU | PA2B\_BUNCE | PA2B\_NOTSC | PA2GA\_HUMAN | PA2GE\_HUMAN | PA2GX\_HUMAN | PA2H1\_AGKCL | PA2H1\_BOTBZ | PA2H1\_BOTJR | PA2H1\_BOTMO | PA2H1\_BOTPI | PA2H2\_BOTAS | PA2H2\_BOTMO | PA2H2\_BOTPI | PA2H2\_CERGO | PA2H3\_BOTPI | PA2HB\_AGKPI | PA2HB\_OXYSC | PA2HH\_TRIST | PA2HS\_ECHCA | PA2H\_BOTPA | PA2H\_DEIAC | PA2H\_PROMB | PA2N\_GLOHA | PA2\_APIME | PAG15\_HUMAN | PELA\_ASPNG | PELB\_ASPNG | PEM1\_PHACH | PER1A\_ARMRU | PER1\_ARAHY | PER1\_SORBI | PER53\_ARATH | PER59\_ARATH | PERL\_BOVIN | PERL\_BUBBU | PERL\_CAPHI | PER\_ARTRA | PER\_COPCI | PGLR1\_ASPAC | PGLR1\_ASPNG | PGLR\_GIBFU | PGPSA\_DROME | PGRP1\_CAMDR | PHAZ\_TALFU | PLA22\_ORYSJ | PLY1\_JUNAS | PME\_DAUCA | PME\_SITOR | POXA\_DICDI | PPT1\_BOVIN | PPT1\_HUMAN | PRTN3\_HUMAN | Q02321\_PHACH | Q0KFV0\_SOLLC | Q12715\_HYPJE | Q2U8V9\_ASPOR | Q40069\_HORVU | Q43576\_TOBAC | Q4AE59\_OSTFU | Q4WP32\_ASPFU | Q55FE6\_DICDI | Q60FD2\_9APHY | Q6VAY1\_9PEZI | Q6WSR8\_PICAB | Q7LHI2\_PHACH | Q7LIJ0\_PHACH | Q7RWP2\_NEUCR | Q86RS6\_MANSE | Q8H0C9\_VIGUN | Q8J0K6\_MELAO | Q8J0K8\_MELAO | Q8NJY6\_9HYPO | Q8TFL9\_TALEM | Q8TG26\_THEAU | Q8TGI8\_TALEM | Q92458\_HYPJE | Q95KP4\_HORSE | Q9FUH3\_VIGUS | Q9P8F7\_YARLL | RGLA\_ASPAC | RHGA\_ASPAC | RNAS6\_HUMAN | RNLE\_SOLLC | RNT2\_HUMAN | S7Q6I2\_GLOTA | S7ZIW0\_PENO1 | SIA\_ASPFU | TPP1\_HUMAN | V5NTD\_NAJAT | VPL1\_PLEER | VPL2\_PLEER | W4KMP1\_HETIT | W6Q990\_PENRF | W8P1L2\_TALEM | W8VR85\_TALPI | X0M5X0\_FUSOX | XGHA\_ASPTU | XYN1\_HYPJR | XYN2\_HYPJR | XYN3\_ASPKW | XYN3\_HYPJQ | XYNA\_FUSO4 | XYNA\_PENSI | XYNA\_THEAU | XYNA\_THELA | XYNC\_ASPNC | XYND\_EMENI | GO:0071715 | 0.0180012414649286 | 58/3222 | 56/552 | 1.32135267147767e-41 | 2.30972446974296e-38 | T | T | T | T | icosanoid transport | A0A1L8D5Z7\_BOTAT | I1SB18\_VIPAE | PA21B\_BOVIN | PA2A1\_BUNCE | PA2A1\_ECHCA | PA2A1\_NAJAT | PA2A1\_OPHHA | PA2A2\_NAJNA | PA2A2\_OPHHA | PA2A2\_TROCA | PA2A4\_NAJSG | PA2A5\_TRIST | PA2A7\_GLOHA | PA2A\_BOTJR | PA2A\_CROAT | PA2A\_DEIAC | PA2A\_GLOHA | PA2A\_NAJAT | PA2B1\_AGKPI | PA2B2\_BOTJR | PA2B2\_PROFL | PA2B3\_BOTAS | PA2B3\_BUNCE | PA2B5\_BUNCE | PA2B5\_NOTSC | PA2BA\_VIPAA | PA2BB\_GLOHA | PA2BB\_PSEAU | PA2BC\_VIPAA | PA2BD\_CRODU | PA2B\_BUNCE | PA2B\_NOTSC | PA2GA\_HUMAN | PA2GE\_HUMAN | PA2GX\_HUMAN | PA2H1\_AGKCL | PA2H1\_BOTBZ | PA2H1\_BOTJR | PA2H1\_BOTMO | PA2H1\_BOTPI | PA2H2\_BOTAS | PA2H2\_BOTMO | PA2H2\_BOTPI | PA2H2\_CERGO | PA2H3\_BOTPI | PA2HB\_AGKPI | PA2HB\_OXYSC | PA2HH\_TRIST | PA2HS\_ECHCA | PA2H\_BOTPA | PA2H\_DEIAC | PA2H\_PROMB | PA2N\_GLOHA | PA2\_APIME | PGH2\_HUMAN | PLA22\_ORYSJ | GO:0032309 | 0.0180012414649286 | 58/3222 | 56/552 | 1.32135267147767e-41 | 2.30972446974296e-38 | T | T | T | T | icosanoid secretion | A0A1L8D5Z7\_BOTAT | I1SB18\_VIPAE | PA21B\_BOVIN | PA2A1\_BUNCE | PA2A1\_ECHCA | PA2A1\_NAJAT | PA2A1\_OPHHA | PA2A2\_NAJNA | PA2A2\_OPHHA | PA2A2\_TROCA | PA2A4\_NAJSG | PA2A5\_TRIST | PA2A7\_GLOHA | PA2A\_BOTJR | PA2A\_CROAT | PA2A\_DEIAC | PA2A\_GLOHA | PA2A\_NAJAT | PA2B1\_AGKPI | PA2B2\_BOTJR | PA2B2\_PROFL | PA2B3\_BOTAS | PA2B3\_BUNCE | PA2B5\_BUNCE | PA2B5\_NOTSC | PA2BA\_VIPAA | PA2BB\_GLOHA | PA2BB\_PSEAU | PA2BC\_VIPAA | PA2BD\_CRODU | PA2B\_BUNCE | PA2B\_NOTSC | PA2GA\_HUMAN | PA2GE\_HUMAN | PA2GX\_HUMAN | PA2H1\_AGKCL | PA2H1\_BOTBZ | PA2H1\_BOTJR | PA2H1\_BOTMO | PA2H1\_BOTPI | PA2H2\_BOTAS | PA2H2\_BOTMO | PA2H2\_BOTPI | PA2H2\_CERGO | PA2H3\_BOTPI | PA2HB\_AGKPI | PA2HB\_OXYSC | PA2HH\_TRIST | PA2HS\_ECHCA | PA2H\_BOTPA | PA2H\_DEIAC | PA2H\_PROMB | PA2N\_GLOHA | PA2\_APIME | PGH2\_HUMAN | PLA22\_ORYSJ | GO:0050482 | 0.0176908752327747 | 57/3222 | 55/552 | 8.12554454087746e-41 | 1.42034518574538e-37 | T | T | T | T | arachidonic acid secretion | A0A1L8D5Z7\_BOTAT | I1SB18\_VIPAE | PA21B\_BOVIN | PA2A1\_BUNCE | PA2A1\_ECHCA | PA2A1\_NAJAT | PA2A1\_OPHHA | PA2A2\_NAJNA | PA2A2\_OPHHA | PA2A2\_TROCA | PA2A4\_NAJSG | PA2A5\_TRIST | PA2A7\_GLOHA | PA2A\_BOTJR | PA2A\_CROAT | PA2A\_DEIAC | PA2A\_GLOHA | PA2A\_NAJAT | PA2B1\_AGKPI | PA2B2\_BOTJR | PA2B2\_PROFL | PA2B3\_BOTAS | PA2B3\_BUNCE | PA2B5\_BUNCE | PA2B5\_NOTSC | PA2BA\_VIPAA | PA2BB\_GLOHA | PA2BB\_PSEAU | PA2BC\_VIPAA | PA2BD\_CRODU | PA2B\_BUNCE | PA2B\_NOTSC | PA2GA\_HUMAN | PA2GE\_HUMAN | PA2GX\_HUMAN | PA2H1\_AGKCL | PA2H1\_BOTBZ | PA2H1\_BOTJR | PA2H1\_BOTMO | PA2H1\_BOTPI | PA2H2\_BOTAS | PA2H2\_BOTMO | PA2H2\_BOTPI | PA2H2\_CERGO | PA2H3\_BOTPI | PA2HB\_AGKPI | PA2HB\_OXYSC | PA2HH\_TRIST | PA2HS\_ECHCA | PA2H\_BOTPA | PA2H\_DEIAC | PA2H\_PROMB | PA2N\_GLOHA | PA2\_APIME | PLA22\_ORYSJ | GO:1903963 | 0.0176908752327747 | 57/3222 | 55/552 | 8.12554454087746e-41 | 1.42034518574538e-37 | T | T | T | T | arachidonate transport | A0A1L8D5Z7\_BOTAT | I1SB18\_VIPAE | PA21B\_BOVIN | PA2A1\_BUNCE | PA2A1\_ECHCA | PA2A1\_NAJAT | PA2A1\_OPHHA | PA2A2\_NAJNA | PA2A2\_OPHHA | PA2A2\_TROCA | PA2A4\_NAJSG | PA2A5\_TRIST | PA2A7\_GLOHA | PA2A\_BOTJR | PA2A\_CROAT | PA2A\_DEIAC | PA2A\_GLOHA | PA2A\_NAJAT | PA2B1\_AGKPI | PA2B2\_BOTJR | PA2B2\_PROFL | PA2B3\_BOTAS | PA2B3\_BUNCE | PA2B5\_BUNCE | PA2B5\_NOTSC | PA2BA\_VIPAA | PA2BB\_GLOHA | PA2BB\_PSEAU | PA2BC\_VIPAA | PA2BD\_CRODU | PA2B\_BUNCE | PA2B\_NOTSC | PA2GA\_HUMAN | PA2GE\_HUMAN | PA2GX\_HUMAN | PA2H1\_AGKCL | PA2H1\_BOTBZ | PA2H1\_BOTJR | PA2H1\_BOTMO | PA2H1\_BOTPI | PA2H2\_BOTAS | PA2H2\_BOTMO | PA2H2\_BOTPI | PA2H2\_CERGO | PA2H3\_BOTPI | PA2HB\_AGKPI | PA2HB\_OXYSC | PA2HH\_TRIST | PA2HS\_ECHCA | PA2H\_BOTPA | PA2H\_DEIAC | PA2H\_PROMB | PA2N\_GLOHA | PA2\_APIME | PLA22\_ORYSJ | GO:0015909 | 0.0180012414649286 | 58/3222 | 55/552 | 1.32869062146431e-39 | 2.32255120631961e-36 | T | T | T | T | long-chain fatty acid transport | A0A1L8D5Z7\_BOTAT | I1SB18\_VIPAE | PA21B\_BOVIN | PA2A1\_BUNCE | PA2A1\_ECHCA | PA2A1\_NAJAT | PA2A1\_OPHHA | PA2A2\_NAJNA | PA2A2\_OPHHA | PA2A2\_TROCA | PA2A4\_NAJSG | PA2A5\_TRIST | PA2A7\_GLOHA | PA2A\_BOTJR | PA2A\_CROAT | PA2A\_DEIAC | PA2A\_GLOHA | PA2A\_NAJAT | PA2B1\_AGKPI | PA2B2\_BOTJR | PA2B2\_PROFL | PA2B3\_BOTAS | PA2B3\_BUNCE | PA2B5\_BUNCE | PA2B5\_NOTSC | PA2BA\_VIPAA | PA2BB\_GLOHA | PA2BB\_PSEAU | PA2BC\_VIPAA | PA2BD\_CRODU | PA2B\_BUNCE | PA2B\_NOTSC | PA2GA\_HUMAN | PA2GE\_HUMAN | PA2GX\_HUMAN | PA2H1\_AGKCL | PA2H1\_BOTBZ | PA2H1\_BOTJR | PA2H1\_BOTMO | PA2H1\_BOTPI | PA2H2\_BOTAS | PA2H2\_BOTMO | PA2H2\_BOTPI | PA2H2\_CERGO | PA2H3\_BOTPI | PA2HB\_AGKPI | PA2HB\_OXYSC | PA2HH\_TRIST | PA2HS\_ECHCA | PA2H\_BOTPA | PA2H\_DEIAC | PA2H\_PROMB | PA2N\_GLOHA | PA2\_APIME | PLA22\_ORYSJ | GO:0005975 | 0.200496585971446 | 646/3222 | 227/552 | 6.76499589708746e-37 | 1.18252128281089e-33 | T | T | T | T | carbohydrate metabolic process | A0A059U759\_9PEZI | A0A068FT77\_9PEZI | A0A075B5H6\_TRIHA | A0A086SY89\_ACRC1 | A0A086T6R4\_ACRC1 | A0A088T0J9\_GEOCN | A0A0J5Q413\_ASPFM | A0A0M3KKZ6\_RHIMI | A0A0M3KKZ8\_RHIMI | A0A0S2GKZ1\_9APHY | A0A173N065\_EISFE | A0A1L6CE30\_9EURO | A0A1L9WG58\_ASPA1 | A0A1S9DRB1\_ASPOZ | A0A2H5BN17\_TALPI | A0A2N1LTK3\_TRIHA | A0A2Z4HIN9\_9EURO | A0A384E148\_NICBE | A0A3B6UEQ2\_RHIMI | A0A3G2C3I4\_9EURO | A0A3G4RHU4\_9PEZI | A0A482LWB1\_OSTFU | A0A5J6BJN2\_MALCI | A0A6F8Z6Y2\_BOMMO | A0A6M9BP13\_9EURO | A0A856TAI5\_9BASI | A1E266\_9PEZI | A2QZC8\_ASPNC | A2TM14\_HEVBR | A5AB48\_ASPNC | A6YRT4\_9PEZI | A8NI40\_COPC7 | A9LI60\_BIOOC | A9ZSX9\_9BRYO | ABFB\_ASPKW | ADPG2\_ARATH | AGAL\_HUMAN | AGAL\_ORYSJ | AMY1A\_HUMAN | AMY1\_HORVU | AMY1\_ORYSJ | AMYA1\_ASPOR | AMYG\_SACFI | AMY\_ORYLA | AXE1\_ASPAW | AXE2\_TALPU | AXHA2\_EMENI | B2ZGS7\_9ASPA | B7X9Z0\_COPCI | B7X9Z2\_COPCI | BGALA\_ASPNC | BGALA\_ASPOR | BGALA\_PENSQ | BGL1\_ASPAC | BGLA\_ASPFU | BGLA\_ASPOR | BGLR\_HUMAN | C3VEV9\_PENCN | C7YSL3\_FUSV7 | CBHB\_ASPFU | CBHRE\_GEOS1 | CDA\_COLLN | CDA\_EMENI | CHI1\_COCPS | CHI2\_HORVU | CHI2\_ORYSJ | CHI33\_TRIHA | CHI42\_TRIHA | CHI4\_CRYJA | CHIA\_HUMAN | CHIC\_ARATH | CHIC\_SECCE | CHIL3\_MOUSE | CHIT\_PUNGR | CHLY\_HEVBR | CONB\_CANEN | D0QF43\_9HELO | D1M8S7\_HEVBR | D9MWI4\_9ASPA | E0XN39\_9EURO | E13B\_HORVU | E13C\_MUSAC | E3VTL0\_9ASPA | E5D0X5\_SCHOC | E9G5J5\_DAPPU | EGLB\_ASPNG | ENG1\_RHIMI | EXG1\_CANAL | EXG1\_YEAST | F0ZJZ1\_DICPU | F1CYZ0\_TALFU | F2Z7L1\_9ANNE | FAEA\_ASPNG | FAEB1\_ASPOR | FAEB2\_ASPOR | FUCO\_HUMAN | G0RVK1\_HYPJQ | G2Q665\_MYCTT | G2QVH2\_THETT | G2X3Y1\_VERDV | G3JPF7\_CORMM | G3YAL0\_ASPNA | G3YFQ1\_ASPNA | G8GLP2\_LENED | G9NTY1\_HYPAI | GANA\_EMENI | GCE\_CERUI | GCE\_HYPJQ | GH7B\_LIMQU | GUB2\_HORVU | GUN2\_HYPJE | GUN6\_HUMIN | GUN7\_HYPJQ | GUNC\_FUSOX | GUN\_ASPAC | GUN\_CRYAT | GUN\_MYTED | GUX1\_HUMGT | GUX1\_HYPJE | GUX1\_TRIHA | GUX2\_HYPJE | H1AE14\_PHACH | HEXC\_OSTFU | HYAL1\_HUMAN | I2FI81\_EISFE | I3RY46\_TRIHA | IDUA\_HUMAN | INU2\_ASPFI | INUE\_ASPAW | INV\_SCHOC | J9UN47\_GIBZA | K9L8F3\_MALCI | L7SVX1\_RHIMI | LAC1\_MELAO | LALBA\_BOVIN | LALBA\_CAPHI | LALBA\_CAVPO | LALBA\_PAPCY | LICH\_HUMAN | M2RAI8\_CERS8 | M9TI89\_RHIPU | MAN12\_PENCI | MANA\_ASPNC | MANA\_CANEN | MANA\_CRYAT | MANA\_HYPJR | MANA\_MYTED | MANA\_PODAN | MANBA\_MOUSE | MEL1\_YEASX | NAGAB\_CHICK | NAGAB\_HUMAN | NANL\_MACDE | O00095\_HYPJE | O74705\_ASPNG | O77044\_9NEOP | O81100\_SOLLC | O81934\_CANEN | OFUT1\_CAEEL | P79074\_9AGAR | PDH1\_LEUMG | PELA\_ASPNG | PELB\_ASPNG | PGLR1\_ASPAC | PGLR1\_ASPNG | PGLR\_GIBFU | PHAZ\_TALFU | PLY1\_JUNAS | PME\_DAUCA | PME\_SITOR | Q02321\_PHACH | Q07524\_TROMA | Q12715\_HYPJE | Q2U8V9\_ASPOR | Q43576\_TOBAC | Q4AE59\_OSTFU | Q4W6L6\_CYCRE | Q4WP32\_ASPFU | Q50KB2\_PHACH | Q55FE6\_DICDI | Q588B8\_CRYJA | Q6VAY1\_9PEZI | Q6WSR8\_PICAB | Q70C53\_SOLTU | Q70SY0\_HYPJE | Q7LHI2\_PHACH | Q7LIJ0\_PHACH | Q7RWP2\_NEUCR | Q7X9A9\_CAMSI | Q8H0C9\_VIGUN | Q8J0K6\_MELAO | Q8J0K8\_MELAO | Q8NJY6\_9HYPO | Q8T0W7\_9NEOP | Q8TFL9\_TALEM | Q8TG26\_THEAU | Q8TGI8\_TALEM | Q92456\_HYPJE | Q92458\_HYPJE | Q93X60\_CICIN | Q9FUH3\_VIGUS | Q9LYJ5\_ARATH | Q9STC1\_GRALE | Q9XEI3\_HORVV | RGLA\_ASPAC | RHGA\_ASPAC | S7Q6I2\_GLOTA | S7ZIW0\_PENO1 | SIA\_ASPFU | W0T408\_KLUMD | W4KMP1\_HETIT | W8P1L2\_TALEM | W8VR85\_TALPI | X0M5X0\_FUSOX | XGHA\_ASPTU | XTH34\_POPPZ | XYLA\_ASPNC | XYN1\_HYPJR | XYN2\_HYPJR | XYN3\_ASPKW | XYN3\_HYPJQ | XYNA\_FUSO4 | XYNA\_PENSI | XYNA\_THEAU | XYNA\_THELA | XYNC\_ASPNC | XYND\_EMENI | GO:0016052 | 0.119801365611421 | 386/3222 | 164/552 | 6.84529761439734e-37 | 1.19655802299666e-33 | T | T | T | T | carbohydrate catabolic process | A0A059U759\_9PEZI | A0A068FT77\_9PEZI | A0A086SY89\_ACRC1 | A0A086T6R4\_ACRC1 | A0A088T0J9\_GEOCN | A0A0J5Q413\_ASPFM | A0A0S2GKZ1\_9APHY | A0A1L6CE30\_9EURO | A0A1L9WG58\_ASPA1 | A0A1S9DRB1\_ASPOZ | A0A2H5BN17\_TALPI | A0A2N1LTK3\_TRIHA | A0A2Z4HIN9\_9EURO | A0A3B6UEQ2\_RHIMI | A0A3G2C3I4\_9EURO | A0A5J6BJN2\_MALCI | A0A6M9BP13\_9EURO | A1E266\_9PEZI | A5AB48\_ASPNC | A6YRT4\_9PEZI | A8NI40\_COPC7 | A9LI60\_BIOOC | ABFB\_ASPKW | ADPG2\_ARATH | AGAL\_ORYSJ | AMY1\_HORVU | AMY1\_ORYSJ | AMYA1\_ASPOR | AMYG\_SACFI | AXE1\_ASPAW | AXE2\_TALPU | AXHA2\_EMENI | B7X9Z0\_COPCI | B7X9Z2\_COPCI | BGALA\_ASPNC | BGALA\_ASPOR | BGALA\_PENSQ | BGL1\_ASPAC | BGLA\_ASPFU | BGLA\_ASPOR | C3VEV9\_PENCN | C7YSL3\_FUSV7 | CBHB\_ASPFU | CBHRE\_GEOS1 | CDA\_COLLN | CDA\_EMENI | CHI1\_COCPS | CHI2\_HORVU | CHI2\_ORYSJ | CHI33\_TRIHA | CHI42\_TRIHA | CHI4\_CRYJA | CHIA\_HUMAN | CHIC\_ARATH | CHIC\_SECCE | CHIL3\_MOUSE | CHIT\_PUNGR | CHLY\_HEVBR | D0QF43\_9HELO | D9MWI4\_9ASPA | E13B\_HORVU | E3VTL0\_9ASPA | E9G5J5\_DAPPU | EGLB\_ASPNG | ENG1\_RHIMI | EXG1\_CANAL | EXG1\_YEAST | F0ZJZ1\_DICPU | F1CYZ0\_TALFU | F2Z7L1\_9ANNE | FAEA\_ASPNG | FAEB1\_ASPOR | FAEB2\_ASPOR | G0RVK1\_HYPJQ | G2Q665\_MYCTT | G2QVH2\_THETT | G2X3Y1\_VERDV | G3JPF7\_CORMM | G3YAL0\_ASPNA | G3YFQ1\_ASPNA | G9NTY1\_HYPAI | GANA\_EMENI | GH7B\_LIMQU | GUN2\_HYPJE | GUN6\_HUMIN | GUN7\_HYPJQ | GUNC\_FUSOX | GUN\_ASPAC | GUN\_CRYAT | GUN\_MYTED | GUX1\_HUMGT | GUX1\_HYPJE | GUX1\_TRIHA | GUX2\_HYPJE | H1AE14\_PHACH | HEXC\_OSTFU | I2FI81\_EISFE | I3RY46\_TRIHA | IDUA\_HUMAN | INU2\_ASPFI | INUE\_ASPAW | K9L8F3\_MALCI | L7SVX1\_RHIMI | LAC1\_MELAO | LICH\_HUMAN | M2RAI8\_CERS8 | M9TI89\_RHIPU | MANA\_ASPNC | MANA\_CRYAT | MANA\_HYPJR | MANA\_MYTED | MANA\_PODAN | MANBA\_MOUSE | NAGAB\_HUMAN | O00095\_HYPJE | O74705\_ASPNG | O77044\_9NEOP | P79074\_9AGAR | PELA\_ASPNG | PELB\_ASPNG | PGLR1\_ASPAC | PGLR1\_ASPNG | PGLR\_GIBFU | PHAZ\_TALFU | PLY1\_JUNAS | PME\_DAUCA | PME\_SITOR | Q02321\_PHACH | Q12715\_HYPJE | Q2U8V9\_ASPOR | Q4WP32\_ASPFU | Q55FE6\_DICDI | Q6VAY1\_9PEZI | Q7LHI2\_PHACH | Q7LIJ0\_PHACH | Q7RWP2\_NEUCR | Q8J0K6\_MELAO | Q8J0K8\_MELAO | Q8NJY6\_9HYPO | Q8TFL9\_TALEM | Q8TG26\_THEAU | Q8TGI8\_TALEM | Q92458\_HYPJE | Q9FUH3\_VIGUS | RGLA\_ASPAC | RHGA\_ASPAC | S7Q6I2\_GLOTA | S7ZIW0\_PENO1 | SIA\_ASPFU | W4KMP1\_HETIT | W8P1L2\_TALEM | W8VR85\_TALPI | X0M5X0\_FUSOX | XGHA\_ASPTU | XYN1\_HYPJR | XYN2\_HYPJR | XYN3\_ASPKW | XYN3\_HYPJQ | XYNA\_FUSO4 | XYNA\_PENSI | XYNA\_THEAU | XYNA\_THELA | XYNC\_ASPNC | XYND\_EMENI | GO:0015908 | 0.0195530726256983 | 63/3222 | 56/552 | 1.91580346432344e-36 | 3.34882445563738e-33 | T | T | T | T | fatty acid transport | A0A1L8D5Z7\_BOTAT | I1SB18\_VIPAE | PA21B\_BOVIN | PA2A1\_BUNCE | PA2A1\_ECHCA | PA2A1\_NAJAT | PA2A1\_OPHHA | PA2A2\_NAJNA | PA2A2\_OPHHA | PA2A2\_TROCA | PA2A4\_NAJSG | PA2A5\_TRIST | PA2A7\_GLOHA | PA2A\_BOTJR | PA2A\_CROAT | PA2A\_DEIAC | PA2A\_GLOHA | PA2A\_NAJAT | PA2B1\_AGKPI | PA2B2\_BOTJR | PA2B2\_PROFL | PA2B3\_BOTAS | PA2B3\_BUNCE | PA2B5\_BUNCE | PA2B5\_NOTSC | PA2BA\_VIPAA | PA2BB\_GLOHA | PA2BB\_PSEAU | PA2BC\_VIPAA | PA2BD\_CRODU | PA2B\_BUNCE | PA2B\_NOTSC | PA2GA\_HUMAN | PA2GE\_HUMAN | PA2GX\_HUMAN | PA2H1\_AGKCL | PA2H1\_BOTBZ | PA2H1\_BOTJR | PA2H1\_BOTMO | PA2H1\_BOTPI | PA2H2\_BOTAS | PA2H2\_BOTMO | PA2H2\_BOTPI | PA2H2\_CERGO | PA2H3\_BOTPI | PA2HB\_AGKPI | PA2HB\_OXYSC | PA2HH\_TRIST | PA2HS\_ECHCA | PA2H\_BOTPA | PA2H\_DEIAC | PA2H\_PROMB | PA2N\_GLOHA | PA2\_APIME | PGH2\_HUMAN | PLA22\_ORYSJ | GO:0051275 | 0.0183116076970826 | 59/3222 | 53/552 | 4.72736858667319e-35 | 8.26344028950473e-32 | T | T | T | T | beta-glucan catabolic process | A0A068FT77\_9PEZI | A0A088T0J9\_GEOCN | A0A0S2GKZ1\_9APHY | A0A2N1LTK3\_TRIHA | A0A5J6BJN2\_MALCI | AXE1\_ASPAW | AXE2\_TALPU | B7X9Z0\_COPCI | B7X9Z2\_COPCI | BGL1\_ASPAC | BGLA\_ASPFU | BGLA\_ASPOR | CBHB\_ASPFU | CBHRE\_GEOS1 | E13B\_HORVU | E9G5J5\_DAPPU | EGLB\_ASPNG | F0ZJZ1\_DICPU | F1CYZ0\_TALFU | F2Z7L1\_9ANNE | FAEA\_ASPNG | G0RVK1\_HYPJQ | G2Q665\_MYCTT | G9NTY1\_HYPAI | GH7B\_LIMQU | GUN2\_HYPJE | GUN6\_HUMIN | GUN7\_HYPJQ | GUNC\_FUSOX | GUN\_ASPAC | GUN\_CRYAT | GUN\_MYTED | GUX1\_HUMGT | GUX1\_HYPJE | GUX1\_TRIHA | GUX2\_HYPJE | H1AE14\_PHACH | I2FI81\_EISFE | LAC1\_MELAO | M2RAI8\_CERS8 | O77044\_9NEOP | Q02321\_PHACH | Q4WP32\_ASPFU | Q55FE6\_DICDI | Q7LHI2\_PHACH | Q7LIJ0\_PHACH | Q7RWP2\_NEUCR | Q8J0K6\_MELAO | Q8J0K8\_MELAO | Q8TFL9\_TALEM | Q8TGI8\_TALEM | W4KMP1\_HETIT | W8P1L2\_TALEM | GO:0030245 | 0.0180012414649286 | 58/3222 | 52/552 | 2.68844518873566e-34 | 4.69940218990993e-31 | T | T | T | T | cellulose catabolic process | A0A068FT77\_9PEZI | A0A088T0J9\_GEOCN | A0A0S2GKZ1\_9APHY | A0A2N1LTK3\_TRIHA | A0A5J6BJN2\_MALCI | AXE1\_ASPAW | AXE2\_TALPU | B7X9Z0\_COPCI | B7X9Z2\_COPCI | BGL1\_ASPAC | BGLA\_ASPFU | BGLA\_ASPOR | CBHB\_ASPFU | CBHRE\_GEOS1 | E9G5J5\_DAPPU | EGLB\_ASPNG | F0ZJZ1\_DICPU | F1CYZ0\_TALFU | F2Z7L1\_9ANNE | FAEA\_ASPNG | G0RVK1\_HYPJQ | G2Q665\_MYCTT | G9NTY1\_HYPAI | GH7B\_LIMQU | GUN2\_HYPJE | GUN6\_HUMIN | GUN7\_HYPJQ | GUNC\_FUSOX | GUN\_ASPAC | GUN\_CRYAT | GUN\_MYTED | GUX1\_HUMGT | GUX1\_HYPJE | GUX1\_TRIHA | GUX2\_HYPJE | H1AE14\_PHACH | I2FI81\_EISFE | LAC1\_MELAO | M2RAI8\_CERS8 | O77044\_9NEOP | Q02321\_PHACH | Q4WP32\_ASPFU | Q55FE6\_DICDI | Q7LHI2\_PHACH | Q7LIJ0\_PHACH | Q7RWP2\_NEUCR | Q8J0K6\_MELAO | Q8J0K8\_MELAO | Q8TFL9\_TALEM | Q8TGI8\_TALEM | W4KMP1\_HETIT | W8P1L2\_TALEM | GO:0015718 | 0.0204841713221601 | 66/3222 | 56/552 | 4.41994801458307e-34 | 7.72606912949121e-31 | T | T | T | T | monocarboxylic acid transport | A0A1L8D5Z7\_BOTAT | I1SB18\_VIPAE | PA21B\_BOVIN | PA2A1\_BUNCE | PA2A1\_ECHCA | PA2A1\_NAJAT | PA2A1\_OPHHA | PA2A2\_NAJNA | PA2A2\_OPHHA | PA2A2\_TROCA | PA2A4\_NAJSG | PA2A5\_TRIST | PA2A7\_GLOHA | PA2A\_BOTJR | PA2A\_CROAT | PA2A\_DEIAC | PA2A\_GLOHA | PA2A\_NAJAT | PA2B1\_AGKPI | PA2B2\_BOTJR | PA2B2\_PROFL | PA2B3\_BOTAS | PA2B3\_BUNCE | PA2B5\_BUNCE | PA2B5\_NOTSC | PA2BA\_VIPAA | PA2BB\_GLOHA | PA2BB\_PSEAU | PA2BC\_VIPAA | PA2BD\_CRODU | PA2B\_BUNCE | PA2B\_NOTSC | PA2GA\_HUMAN | PA2GE\_HUMAN | PA2GX\_HUMAN | PA2H1\_AGKCL | PA2H1\_BOTBZ | PA2H1\_BOTJR | PA2H1\_BOTMO | PA2H1\_BOTPI | PA2H2\_BOTAS | PA2H2\_BOTMO | PA2H2\_BOTPI | PA2H2\_CERGO | PA2H3\_BOTPI | PA2HB\_AGKPI | PA2HB\_OXYSC | PA2HH\_TRIST | PA2HS\_ECHCA | PA2H\_BOTPA | PA2H\_DEIAC | PA2H\_PROMB | PA2N\_GLOHA | PA2\_APIME | PGH2\_HUMAN | PLA22\_ORYSJ | GO:0030243 | 0.0183116076970826 | 59/3222 | 52/552 | 1.91479927835255e-33 | 3.34706913856026e-30 | T | T | T | T | cellulose metabolic process | A0A068FT77\_9PEZI | A0A088T0J9\_GEOCN | A0A0S2GKZ1\_9APHY | A0A2N1LTK3\_TRIHA | A0A5J6BJN2\_MALCI | AXE1\_ASPAW | AXE2\_TALPU | B7X9Z0\_COPCI | B7X9Z2\_COPCI | BGL1\_ASPAC | BGLA\_ASPFU | BGLA\_ASPOR | CBHB\_ASPFU | CBHRE\_GEOS1 | E9G5J5\_DAPPU | EGLB\_ASPNG | F0ZJZ1\_DICPU | F1CYZ0\_TALFU | F2Z7L1\_9ANNE | FAEA\_ASPNG | G0RVK1\_HYPJQ | G2Q665\_MYCTT | G9NTY1\_HYPAI | GH7B\_LIMQU | GUN2\_HYPJE | GUN6\_HUMIN | GUN7\_HYPJQ | GUNC\_FUSOX | GUN\_ASPAC | GUN\_CRYAT | GUN\_MYTED | GUX1\_HUMGT | GUX1\_HYPJE | GUX1\_TRIHA | GUX2\_HYPJE | H1AE14\_PHACH | I2FI81\_EISFE | LAC1\_MELAO | M2RAI8\_CERS8 | O77044\_9NEOP | Q02321\_PHACH | Q4WP32\_ASPFU | Q55FE6\_DICDI | Q7LHI2\_PHACH | Q7LIJ0\_PHACH | Q7RWP2\_NEUCR | Q8J0K6\_MELAO | Q8J0K8\_MELAO | Q8TFL9\_TALEM | Q8TGI8\_TALEM | W4KMP1\_HETIT | W8P1L2\_TALEM | GO:0046942 | 0.021104903786468 | 68/3222 | 56/552 | 1.09102662867353e-32 | 1.90711454692133e-29 | T | T | T | T | carboxylic acid transport | A0A1L8D5Z7\_BOTAT | I1SB18\_VIPAE | PA21B\_BOVIN | PA2A1\_BUNCE | PA2A1\_ECHCA | PA2A1\_NAJAT | PA2A1\_OPHHA | PA2A2\_NAJNA | PA2A2\_OPHHA | PA2A2\_TROCA | PA2A4\_NAJSG | PA2A5\_TRIST | PA2A7\_GLOHA | PA2A\_BOTJR | PA2A\_CROAT | PA2A\_DEIAC | PA2A\_GLOHA | PA2A\_NAJAT | PA2B1\_AGKPI | PA2B2\_BOTJR | PA2B2\_PROFL | PA2B3\_BOTAS | PA2B3\_BUNCE | PA2B5\_BUNCE | PA2B5\_NOTSC | PA2BA\_VIPAA | PA2BB\_GLOHA | PA2BB\_PSEAU | PA2BC\_VIPAA | PA2BD\_CRODU | PA2B\_BUNCE | PA2B\_NOTSC | PA2GA\_HUMAN | PA2GE\_HUMAN | PA2GX\_HUMAN | PA2H1\_AGKCL | PA2H1\_BOTBZ | PA2H1\_BOTJR | PA2H1\_BOTMO | PA2H1\_BOTPI | PA2H2\_BOTAS | PA2H2\_BOTMO | PA2H2\_BOTPI | PA2H2\_CERGO | PA2H3\_BOTPI | PA2HB\_AGKPI | PA2HB\_OXYSC | PA2HH\_TRIST | PA2HS\_ECHCA | PA2H\_BOTPA | PA2H\_DEIAC | PA2H\_PROMB | PA2N\_GLOHA | PA2\_APIME | PGH2\_HUMAN | PLA22\_ORYSJ | GO:0015849 | 0.021104903786468 | 68/3222 | 56/552 | 1.09102662867353e-32 | 1.90711454692133e-29 | T | T | T | T | organic acid transport | A0A1L8D5Z7\_BOTAT | I1SB18\_VIPAE | PA21B\_BOVIN | PA2A1\_BUNCE | PA2A1\_ECHCA | PA2A1\_NAJAT | PA2A1\_OPHHA | PA2A2\_NAJNA | PA2A2\_OPHHA | PA2A2\_TROCA | PA2A4\_NAJSG | PA2A5\_TRIST | PA2A7\_GLOHA | PA2A\_BOTJR | PA2A\_CROAT | PA2A\_DEIAC | PA2A\_GLOHA | PA2A\_NAJAT | PA2B1\_AGKPI | PA2B2\_BOTJR | PA2B2\_PROFL | PA2B3\_BOTAS | PA2B3\_BUNCE | PA2B5\_BUNCE | PA2B5\_NOTSC | PA2BA\_VIPAA | PA2BB\_GLOHA | PA2BB\_PSEAU | PA2BC\_VIPAA | PA2BD\_CRODU | PA2B\_BUNCE | PA2B\_NOTSC | PA2GA\_HUMAN | PA2GE\_HUMAN | PA2GX\_HUMAN | PA2H1\_AGKCL | PA2H1\_BOTBZ | PA2H1\_BOTJR | PA2H1\_BOTMO | PA2H1\_BOTPI | PA2H2\_BOTAS | PA2H2\_BOTMO | PA2H2\_BOTPI | PA2H2\_CERGO | PA2H3\_BOTPI | PA2HB\_AGKPI | PA2HB\_OXYSC | PA2HH\_TRIST | PA2HS\_ECHCA | PA2H\_BOTPA | PA2H\_DEIAC | PA2H\_PROMB | PA2N\_GLOHA | PA2\_APIME | PGH2\_HUMAN | PLA22\_ORYSJ | GO:0051273 | 0.0192427063935444 | 62/3222 | 53/552 | 1.28505452019288e-32 | 2.24627530129716e-29 | T | T | T | T | beta-glucan metabolic process | A0A068FT77\_9PEZI | A0A088T0J9\_GEOCN | A0A0S2GKZ1\_9APHY | A0A2N1LTK3\_TRIHA | A0A5J6BJN2\_MALCI | AXE1\_ASPAW | AXE2\_TALPU | B7X9Z0\_COPCI | B7X9Z2\_COPCI | BGL1\_ASPAC | BGLA\_ASPFU | BGLA\_ASPOR | CBHB\_ASPFU | CBHRE\_GEOS1 | E13B\_HORVU | E9G5J5\_DAPPU | EGLB\_ASPNG | F0ZJZ1\_DICPU | F1CYZ0\_TALFU | F2Z7L1\_9ANNE | FAEA\_ASPNG | G0RVK1\_HYPJQ | G2Q665\_MYCTT | G9NTY1\_HYPAI | GH7B\_LIMQU | GUN2\_HYPJE | GUN6\_HUMIN | GUN7\_HYPJQ | GUNC\_FUSOX | GUN\_ASPAC | GUN\_CRYAT | GUN\_MYTED | GUX1\_HUMGT | GUX1\_HYPJE | GUX1\_TRIHA | GUX2\_HYPJE | H1AE14\_PHACH | I2FI81\_EISFE | LAC1\_MELAO | M2RAI8\_CERS8 | O77044\_9NEOP | Q02321\_PHACH | Q4WP32\_ASPFU | Q55FE6\_DICDI | Q7LHI2\_PHACH | Q7LIJ0\_PHACH | Q7RWP2\_NEUCR | Q8J0K6\_MELAO | Q8J0K8\_MELAO | Q8TFL9\_TALEM | Q8TGI8\_TALEM | W4KMP1\_HETIT | W8P1L2\_TALEM | GO:0009251 | 0.0223463687150838 | 72/3222 | 57/552 | 1.64591061799947e-31 | 2.87705176026308e-28 | T | T | T | T | glucan catabolic process | A0A068FT77\_9PEZI | A0A088T0J9\_GEOCN | A0A0S2GKZ1\_9APHY | A0A2N1LTK3\_TRIHA | A0A5J6BJN2\_MALCI | AMY1\_HORVU | AMY1\_ORYSJ | AXE1\_ASPAW | AXE2\_TALPU | B7X9Z0\_COPCI | B7X9Z2\_COPCI | BGL1\_ASPAC | BGLA\_ASPFU | BGLA\_ASPOR | CBHB\_ASPFU | CBHRE\_GEOS1 | E13B\_HORVU | E9G5J5\_DAPPU | EGLB\_ASPNG | EXG1\_CANAL | EXG1\_YEAST | F0ZJZ1\_DICPU | F1CYZ0\_TALFU | F2Z7L1\_9ANNE | FAEA\_ASPNG | G0RVK1\_HYPJQ | G2Q665\_MYCTT | G9NTY1\_HYPAI | GH7B\_LIMQU | GUN2\_HYPJE | GUN6\_HUMIN | GUN7\_HYPJQ | GUNC\_FUSOX | GUN\_ASPAC | GUN\_CRYAT | GUN\_MYTED | GUX1\_HUMGT | GUX1\_HYPJE | GUX1\_TRIHA | GUX2\_HYPJE | H1AE14\_PHACH | I2FI81\_EISFE | LAC1\_MELAO | M2RAI8\_CERS8 | O77044\_9NEOP | Q02321\_PHACH | Q4WP32\_ASPFU | Q55FE6\_DICDI | Q7LHI2\_PHACH | Q7LIJ0\_PHACH | Q7RWP2\_NEUCR | Q8J0K6\_MELAO | Q8J0K8\_MELAO | Q8TFL9\_TALEM | Q8TGI8\_TALEM | W4KMP1\_HETIT | W8P1L2\_TALEM | GO:0015711 | 0.0217256362507759 | 70/3222 | 56/552 | 2.07033363919015e-31 | 3.61894320130439e-28 | T | T | T | T | organic anion transport | A0A1L8D5Z7\_BOTAT | I1SB18\_VIPAE | PA21B\_BOVIN | PA2A1\_BUNCE | PA2A1\_ECHCA | PA2A1\_NAJAT | PA2A1\_OPHHA | PA2A2\_NAJNA | PA2A2\_OPHHA | PA2A2\_TROCA | PA2A4\_NAJSG | PA2A5\_TRIST | PA2A7\_GLOHA | PA2A\_BOTJR | PA2A\_CROAT | PA2A\_DEIAC | PA2A\_GLOHA | PA2A\_NAJAT | PA2B1\_AGKPI | PA2B2\_BOTJR | PA2B2\_PROFL | PA2B3\_BOTAS | PA2B3\_BUNCE | PA2B5\_BUNCE | PA2B5\_NOTSC | PA2BA\_VIPAA | PA2BB\_GLOHA | PA2BB\_PSEAU | PA2BC\_VIPAA | PA2BD\_CRODU | PA2B\_BUNCE | PA2B\_NOTSC | PA2GA\_HUMAN | PA2GE\_HUMAN | PA2GX\_HUMAN | PA2H1\_AGKCL | PA2H1\_BOTBZ | PA2H1\_BOTJR | PA2H1\_BOTMO | PA2H1\_BOTPI | PA2H2\_BOTAS | PA2H2\_BOTMO | PA2H2\_BOTPI | PA2H2\_CERGO | PA2H3\_BOTPI | PA2HB\_AGKPI | PA2HB\_OXYSC | PA2HH\_TRIST | PA2HS\_ECHCA | PA2H\_BOTPA | PA2H\_DEIAC | PA2H\_PROMB | PA2N\_GLOHA | PA2\_APIME | PGH2\_HUMAN | PLA22\_ORYSJ | GO:0016998 | 0.0186219739292365 | 60/3222 | 51/552 | 3.74153485205475e-31 | 6.5402029213917e-28 | T | T | T | T | cell wall macromolecule catabolic process | A0A059U759\_9PEZI | A0A086SY89\_ACRC1 | A0A0J5Q413\_ASPFM | A0A1L9WG58\_ASPA1 | A0A2H5BN17\_TALPI | A0A7S6G7I6\_9PEZI | A8NI40\_COPC7 | A9ZSX9\_9BRYO | ABFB\_ASPKW | AXE1\_ASPAW | AXE2\_TALPU | AXHA2\_EMENI | C3VEV9\_PENCN | C7YSL3\_FUSV7 | CHI2\_HORVU | CHI2\_ORYSJ | CHI4\_CRYJA | CHIC\_SECCE | D0QF43\_9HELO | D9MWI4\_9ASPA | E3VTL0\_9ASPA | FAEA\_ASPNG | FAEB1\_ASPOR | FAEB2\_ASPOR | G2QVH2\_THETT | GANA\_HUMIN | L7SVX1\_RHIMI | MANA\_ASPNC | MANA\_CRYAT | MANA\_HYPJR | MANA\_MYTED | MANA\_PODAN | O81934\_CANEN | PHAZ\_TALFU | Q6VAY1\_9PEZI | Q6WSR8\_PICAB | Q8H0C9\_VIGUN | Q92458\_HYPJE | Q9FUH3\_VIGUS | W8VR85\_TALPI | X0M5X0\_FUSOX | XYN1\_HYPJR | XYN2\_HYPJR | XYN3\_ASPKW | XYN3\_HYPJQ | XYNA\_FUSO4 | XYNA\_PENSI | XYNA\_THEAU | XYNA\_THELA | XYNC\_ASPNC | XYND\_EMENI | GO:0006869 | 0.0220360024829299 | 71/3222 | 56/552 | 8.2859522488148e-31 | 1.44838445309283e-27 | T | T | T | T | lipid transport | A0A1L8D5Z7\_BOTAT | I1SB18\_VIPAE | PA21B\_BOVIN | PA2A1\_BUNCE | PA2A1\_ECHCA | PA2A1\_NAJAT | PA2A1\_OPHHA | PA2A2\_NAJNA | PA2A2\_OPHHA | PA2A2\_TROCA | PA2A4\_NAJSG | PA2A5\_TRIST | PA2A7\_GLOHA | PA2A\_BOTJR | PA2A\_CROAT | PA2A\_DEIAC | PA2A\_GLOHA | PA2A\_NAJAT | PA2B1\_AGKPI | PA2B2\_BOTJR | PA2B2\_PROFL | PA2B3\_BOTAS | PA2B3\_BUNCE | PA2B5\_BUNCE | PA2B5\_NOTSC | PA2BA\_VIPAA | PA2BB\_GLOHA | PA2BB\_PSEAU | PA2BC\_VIPAA | PA2BD\_CRODU | PA2B\_BUNCE | PA2B\_NOTSC | PA2GA\_HUMAN | PA2GE\_HUMAN | PA2GX\_HUMAN | PA2H1\_AGKCL | PA2H1\_BOTBZ | PA2H1\_BOTJR | PA2H1\_BOTMO | PA2H1\_BOTPI | PA2H2\_BOTAS | PA2H2\_BOTMO | PA2H2\_BOTPI | PA2H2\_CERGO | PA2H3\_BOTPI | PA2HB\_AGKPI | PA2HB\_OXYSC | PA2HH\_TRIST | PA2HS\_ECHCA | PA2H\_BOTPA | PA2H\_DEIAC | PA2H\_PROMB | PA2N\_GLOHA | PA2\_APIME | PGH2\_HUMAN | PLA22\_ORYSJ | GO:0044036 | 0.021415270018622 | 69/3222 | 53/552 | 3.72798193489943e-28 | 6.5165124222042e-25 | T | T | T | T | cell wall macromolecule metabolic process | A0A059U759\_9PEZI | A0A086SY89\_ACRC1 | A0A0J5Q413\_ASPFM | A0A1L9WG58\_ASPA1 | A0A2H5BN17\_TALPI | A0A7S6G7I6\_9PEZI | A8NI40\_COPC7 | A9ZSX9\_9BRYO | ABFB\_ASPKW | AXE1\_ASPAW | AXE2\_TALPU | AXHA2\_EMENI | C3VEV9\_PENCN | C7YSL3\_FUSV7 | CHI2\_HORVU | CHI2\_ORYSJ | CHI4\_CRYJA | CHIC\_SECCE | D0QF43\_9HELO | D9MWI4\_9ASPA | E3VTL0\_9ASPA | FAEA\_ASPNG | FAEB1\_ASPOR | FAEB2\_ASPOR | G2QVH2\_THETT | GANA\_HUMIN | L7SVX1\_RHIMI | MANA\_ASPNC | MANA\_CRYAT | MANA\_HYPJR | MANA\_MYTED | MANA\_PODAN | O81934\_CANEN | PHAZ\_TALFU | Q07524\_TROMA | Q6VAY1\_9PEZI | Q6WSR8\_PICAB | Q8H0C9\_VIGUN | Q92458\_HYPJE | Q9FUH3\_VIGUS | W8VR85\_TALPI | X0M5X0\_FUSOX | XTH34\_POPPZ | XYN1\_HYPJR | XYN2\_HYPJR | XYN3\_ASPKW | XYN3\_HYPJQ | XYNA\_FUSO4 | XYNA\_PENSI | XYNA\_THEAU | XYNA\_THELA | XYNC\_ASPNC | XYND\_EMENI | GO:0046903 | 0.0270018621973929 | 87/3222 | 60/552 | 1.71821818674476e-27 | 3.00344539042984e-24 | T | T | T | T | secretion | A0A1L8D5Z7\_BOTAT | CERU\_RAT | I1SB18\_VIPAE | PA21B\_BOVIN | PA2A1\_BUNCE | PA2A1\_ECHCA | PA2A1\_NAJAT | PA2A1\_OPHHA | PA2A2\_NAJNA | PA2A2\_OPHHA | PA2A2\_TROCA | PA2A4\_NAJSG | PA2A5\_TRIST | PA2A7\_GLOHA | PA2A\_BOTJR | PA2A\_CROAT | PA2A\_DEIAC | PA2A\_GLOHA | PA2A\_NAJAT | PA2B1\_AGKPI | PA2B2\_BOTJR | PA2B2\_PROFL | PA2B3\_BOTAS | PA2B3\_BUNCE | PA2B5\_BUNCE | PA2B5\_NOTSC | PA2BA\_VIPAA | PA2BB\_GLOHA | PA2BB\_PSEAU | PA2BC\_VIPAA | PA2BD\_CRODU | PA2B\_BUNCE | PA2B\_NOTSC | PA2GA\_HUMAN | PA2GE\_HUMAN | PA2GX\_HUMAN | PA2H1\_AGKCL | PA2H1\_BOTBZ | PA2H1\_BOTJR | PA2H1\_BOTMO | PA2H1\_BOTPI | PA2H2\_BOTAS | PA2H2\_BOTMO | PA2H2\_BOTPI | PA2H2\_CERGO | PA2H3\_BOTPI | PA2HB\_AGKPI | PA2HB\_OXYSC | PA2HH\_TRIST | PA2HS\_ECHCA | PA2H\_BOTPA | PA2H\_DEIAC | PA2H\_PROMB | PA2N\_GLOHA | PA2\_APIME | PGH2\_HUMAN | PLA22\_ORYSJ | PPT1\_HUMAN | PTGDS\_HUMAN | PTGDS\_MOUSE | GO:0044347 | 0.0142768466790813 | 46/3222 | 40/552 | 2.26793952310376e-25 | 3.96435828638537e-22 | T | T | T | T | cell wall polysaccharide catabolic process | A0A059U759\_9PEZI | A0A086SY89\_ACRC1 | A0A0J5Q413\_ASPFM | A0A1L9WG58\_ASPA1 | A0A2H5BN17\_TALPI | A8NI40\_COPC7 | ABFB\_ASPKW | AXE1\_ASPAW | AXE2\_TALPU | AXHA2\_EMENI | C3VEV9\_PENCN | C7YSL3\_FUSV7 | D0QF43\_9HELO | D9MWI4\_9ASPA | E3VTL0\_9ASPA | FAEA\_ASPNG | FAEB1\_ASPOR | FAEB2\_ASPOR | G2QVH2\_THETT | L7SVX1\_RHIMI | MANA\_ASPNC | MANA\_CRYAT | MANA\_HYPJR | MANA\_MYTED | MANA\_PODAN | PHAZ\_TALFU | Q6VAY1\_9PEZI | Q92458\_HYPJE | W8VR85\_TALPI | X0M5X0\_FUSOX | XYN1\_HYPJR | XYN2\_HYPJR | XYN3\_ASPKW | XYN3\_HYPJQ | XYNA\_FUSO4 | XYNA\_PENSI | XYNA\_THEAU | XYNA\_THELA | XYNC\_ASPNC | XYND\_EMENI | GO:0044042 | 0.0279329608938547 | 90/3222 | 59/552 | 3.1350327532134e-25 | 5.48003725261702e-22 | T | T | T | T | glucan metabolic process | A0A068FT77\_9PEZI | A0A088T0J9\_GEOCN | A0A0S2GKZ1\_9APHY | A0A2N1LTK3\_TRIHA | A0A5J6BJN2\_MALCI | AMY1\_HORVU | AMY1\_ORYSJ | AXE1\_ASPAW | AXE2\_TALPU | B7X9Z0\_COPCI | B7X9Z2\_COPCI | BGL1\_ASPAC | BGLA\_ASPFU | BGLA\_ASPOR | CBHB\_ASPFU | CBHRE\_GEOS1 | E13B\_HORVU | E9G5J5\_DAPPU | EGLB\_ASPNG | EXG1\_CANAL | EXG1\_YEAST | F0ZJZ1\_DICPU | F1CYZ0\_TALFU | F2Z7L1\_9ANNE | FAEA\_ASPNG | G0RVK1\_HYPJQ | G2Q665\_MYCTT | G9NTY1\_HYPAI | GH7B\_LIMQU | GUN2\_HYPJE | GUN6\_HUMIN | GUN7\_HYPJQ | GUNC\_FUSOX | GUN\_ASPAC | GUN\_CRYAT | GUN\_MYTED | GUX1\_HUMGT | GUX1\_HYPJE | GUX1\_TRIHA | GUX2\_HYPJE | H1AE14\_PHACH | I2FI81\_EISFE | LAC1\_MELAO | M2RAI8\_CERS8 | O77044\_9NEOP | Q02321\_PHACH | Q07524\_TROMA | Q4WP32\_ASPFU | Q55FE6\_DICDI | Q7LHI2\_PHACH | Q7LIJ0\_PHACH | Q7RWP2\_NEUCR | Q8J0K6\_MELAO | Q8J0K8\_MELAO | Q8TFL9\_TALEM | Q8TGI8\_TALEM | W4KMP1\_HETIT | W8P1L2\_TALEM | XTH34\_POPPZ | GO:0042742 | 0.0226567349472377 | 73/3222 | 52/552 | 5.73666635004672e-25 | 1.00276927798817e-21 | T | T | T | T | defense response to bacterium | A6PZ97\_SALSA | ANG1\_BOVIN | ANG2\_MOUSE | ANG3\_MOUSE | ANG4\_MOUSE | ANGI\_MOUSE | B9TU22\_GADMO | ECP\_HUMAN | GRASS\_DROME | L8ICE9\_9CETA | LYG\_STRCA | LYSC1\_ANAPL | LYSC1\_CANLF | LYSC1\_HORSE | LYSC2\_BOVIN | LYSC2\_ONCMY | LYSC\_COTJA | LYSC\_EQUAS | LYSC\_NUMME | LYSC\_OPIHO | LYSC\_PELSI | LYS\_BOMMO | LYS\_RUDPH | OXLA\_BOTAT | OXLA\_CALRH | OXLA\_GLOHA | PA21B\_BOVIN | PA2A\_BOTJR | PA2B3\_BOTAS | PA2GA\_HUMAN | PA2H1\_BOTBZ | PA2H1\_BOTJR | PA2H2\_BOTAS | PA2H2\_BOTMO | PA2H\_BOTPA | PERL\_BOVIN | PERL\_BUBBU | PERL\_CAPHI | PGPSA\_DROME | PGRP1\_CAMDR | POXA\_DICDI | PPA5\_HUMAN | PPA5\_RAT | Q5WRG2\_RAT | RNAS4\_HUMAN | RNAS4\_PIG | RNAS6\_HUMAN | RNS1B\_RAT | RNSL3\_DANRE | RNS\_BOVIN | TRFL\_BUBBU | TRFL\_HORSE | GO:0010383 | 0.015828677839851 | 51/3222 | 42/552 | 1.14385095409426e-24 | 1.99945146775677e-21 | T | T | T | T | cell wall polysaccharide metabolic process | A0A059U759\_9PEZI | A0A086SY89\_ACRC1 | A0A0J5Q413\_ASPFM | A0A1L9WG58\_ASPA1 | A0A2H5BN17\_TALPI | A8NI40\_COPC7 | ABFB\_ASPKW | AXE1\_ASPAW | AXE2\_TALPU | AXHA2\_EMENI | C3VEV9\_PENCN | C7YSL3\_FUSV7 | D0QF43\_9HELO | D9MWI4\_9ASPA | E3VTL0\_9ASPA | FAEA\_ASPNG | FAEB1\_ASPOR | FAEB2\_ASPOR | G2QVH2\_THETT | L7SVX1\_RHIMI | MANA\_ASPNC | MANA\_CRYAT | MANA\_HYPJR | MANA\_MYTED | MANA\_PODAN | PHAZ\_TALFU | Q07524\_TROMA | Q6VAY1\_9PEZI | Q92458\_HYPJE | W8VR85\_TALPI | X0M5X0\_FUSOX | XTH34\_POPPZ | XYN1\_HYPJR | XYN2\_HYPJR | XYN3\_ASPKW | XYN3\_HYPJQ | XYNA\_FUSO4 | XYNA\_PENSI | XYNA\_THEAU | XYNA\_THELA | XYNC\_ASPNC | XYND\_EMENI | GO:0010410 | 0.0127250155183116 | 41/3222 | 36/552 | 3.14842591264341e-23 | 5.50344849530069e-20 | T | T | T | T | hemicellulose metabolic process | A0A059U759\_9PEZI | A0A086SY89\_ACRC1 | A0A0J5Q413\_ASPFM | A0A1L9WG58\_ASPA1 | A0A2H5BN17\_TALPI | A8NI40\_COPC7 | ABFB\_ASPKW | AXE1\_ASPAW | AXE2\_TALPU | AXHA2\_EMENI | C3VEV9\_PENCN | C7YSL3\_FUSV7 | D0QF43\_9HELO | D9MWI4\_9ASPA | E3VTL0\_9ASPA | FAEA\_ASPNG | FAEB1\_ASPOR | FAEB2\_ASPOR | G2QVH2\_THETT | PHAZ\_TALFU | Q07524\_TROMA | Q6VAY1\_9PEZI | Q92458\_HYPJE | W8VR85\_TALPI | X0M5X0\_FUSOX | XTH34\_POPPZ | XYN1\_HYPJR | XYN2\_HYPJR | XYN3\_ASPKW | XYN3\_HYPJQ | XYNA\_FUSO4 | XYNA\_PENSI | XYNA\_THEAU | XYNA\_THELA | XYNC\_ASPNC | XYND\_EMENI | GO:0045493 | 0.0117939168218498 | 38/3222 | 34/552 | 1.39508992550496e-22 | 2.43861718978267e-19 | T | T | T | T | xylan catabolic process | A0A059U759\_9PEZI | A0A086SY89\_ACRC1 | A0A0J5Q413\_ASPFM | A0A1L9WG58\_ASPA1 | A0A2H5BN17\_TALPI | A8NI40\_COPC7 | ABFB\_ASPKW | AXE1\_ASPAW | AXE2\_TALPU | AXHA2\_EMENI | C3VEV9\_PENCN | C7YSL3\_FUSV7 | D0QF43\_9HELO | D9MWI4\_9ASPA | E3VTL0\_9ASPA | FAEA\_ASPNG | FAEB1\_ASPOR | FAEB2\_ASPOR | G2QVH2\_THETT | PHAZ\_TALFU | Q6VAY1\_9PEZI | Q92458\_HYPJE | W8VR85\_TALPI | X0M5X0\_FUSOX | XYN1\_HYPJR | XYN2\_HYPJR | XYN3\_ASPKW | XYN3\_HYPJQ | XYNA\_FUSO4 | XYNA\_PENSI | XYNA\_THEAU | XYNA\_THELA | XYNC\_ASPNC | XYND\_EMENI | GO:2000895 | 0.0117939168218498 | 38/3222 | 34/552 | 1.39508992550496e-22 | 2.43861718978267e-19 | T | T | T | T | hemicellulose catabolic process | A0A059U759\_9PEZI | A0A086SY89\_ACRC1 | A0A0J5Q413\_ASPFM | A0A1L9WG58\_ASPA1 | A0A2H5BN17\_TALPI | A8NI40\_COPC7 | ABFB\_ASPKW | AXE1\_ASPAW | AXE2\_TALPU | AXHA2\_EMENI | C3VEV9\_PENCN | C7YSL3\_FUSV7 | D0QF43\_9HELO | D9MWI4\_9ASPA | E3VTL0\_9ASPA | FAEA\_ASPNG | FAEB1\_ASPOR | FAEB2\_ASPOR | G2QVH2\_THETT | PHAZ\_TALFU | Q6VAY1\_9PEZI | Q92458\_HYPJE | W8VR85\_TALPI | X0M5X0\_FUSOX | XYN1\_HYPJR | XYN2\_HYPJR | XYN3\_ASPKW | XYN3\_HYPJQ | XYNA\_FUSO4 | XYNA\_PENSI | XYNA\_THEAU | XYNA\_THELA | XYNC\_ASPNC | XYND\_EMENI | GO:0045491 | 0.0117939168218498 | 38/3222 | 34/552 | 1.39508992550496e-22 | 2.43861718978267e-19 | T | T | T | T | xylan metabolic process | A0A059U759\_9PEZI | A0A086SY89\_ACRC1 | A0A0J5Q413\_ASPFM | A0A1L9WG58\_ASPA1 | A0A2H5BN17\_TALPI | A8NI40\_COPC7 | ABFB\_ASPKW | AXE1\_ASPAW | AXE2\_TALPU | AXHA2\_EMENI | C3VEV9\_PENCN | C7YSL3\_FUSV7 | D0QF43\_9HELO | D9MWI4\_9ASPA | E3VTL0\_9ASPA | FAEA\_ASPNG | FAEB1\_ASPOR | FAEB2\_ASPOR | G2QVH2\_THETT | PHAZ\_TALFU | Q6VAY1\_9PEZI | Q92458\_HYPJE | W8VR85\_TALPI | X0M5X0\_FUSOX | XYN1\_HYPJR | XYN2\_HYPJR | XYN3\_ASPKW | XYN3\_HYPJQ | XYNA\_FUSO4 | XYNA\_PENSI | XYNA\_THEAU | XYNA\_THELA | XYNC\_ASPNC | XYND\_EMENI | GO:0006952 | 0.063935443823712 | 206/3222 | 93/552 | 1.80589375111924e-22 | 3.15670227695644e-19 | T | T | T | T | defense response | A6PZ97\_SALSA | ANG1\_BOVIN | ANG2\_MOUSE | ANG3\_MOUSE | ANG4\_MOUSE | ANGI\_MOUSE | B9TU22\_GADMO | CFAD\_MOUSE | CHI2\_HORVU | CHI2\_ORYSJ | CHI4\_CRYJA | CHIC\_SECCE | CHIL3\_MOUSE | DIR\_GLYEC | E0CX04\_MOMBA | E13B\_HORVU | ECP\_HUMAN | GRAC\_MOUSE | GRASS\_DROME | HYAL1\_HUMAN | L8ICE9\_9CETA | LIPR2\_RAT | LYG\_STRCA | LYSC1\_ANAPL | LYSC1\_CANLF | LYSC1\_HORSE | LYSC2\_BOVIN | LYSC2\_ONCMY | LYSC\_COTJA | LYSC\_EQUAS | LYSC\_NUMME | LYSC\_OPIHO | LYSC\_PELSI | LYS\_BOMMO | LYS\_RUDPH | NCS\_THLFG | O04358\_IRIHO | O81934\_CANEN | OXLA\_BOTAT | OXLA\_CALRH | OXLA\_GLOHA | PA21B\_BOVIN | PA2A\_BOTJR | PA2B3\_BOTAS | PA2GA\_HUMAN | PA2GE\_HUMAN | PA2GX\_HUMAN | PA2H1\_BOTBZ | PA2H1\_BOTJR | PA2H2\_BOTAS | PA2H2\_BOTMO | PA2H\_BOTPA | PCP\_HUMAN | PER53\_ARATH | PERL\_BOVIN | PERL\_BUBBU | PERL\_CAPHI | PGH2\_HUMAN | PGPSA\_DROME | PGRP1\_CAMDR | POXA\_DICDI | PPA5\_HUMAN | PPA5\_RAT | PPAF1\_HOLDI | PRTN3\_HUMAN | Q2QEH4\_SAPOF | Q5WRG2\_RAT | Q70C53\_SOLTU | Q86RS6\_MANSE | Q8H0C9\_VIGUN | Q94BW3\_CINCA | Q9FUH3\_VIGUS | RIP0\_DIACA | RIP1\_BRYDI | RIP1\_HORVU | RIP1\_MOMCH | RIP1\_PHYAM | RIP2\_PHYAM | RIP3\_MOMCH | RIPA\_PHYAM | RIPG\_SURMU | RIPL1\_PHYDI | RIPL2\_PHYDI | RIPT\_TRIKI | RNAS4\_HUMAN | RNAS4\_PIG | RNAS6\_HUMAN | RNS1B\_RAT | RNSL3\_DANRE | RNS\_BOVIN | RNT2\_HUMAN | TRFL\_BUBBU | TRFL\_HORSE | GO:0016042 | 0.0664183736809435 | 214/3222 | 94/552 | 1.2010640516307e-21 | 2.09945996225046e-18 | T | T | T | T | lipid catabolic process | A0A1L8D5Z7\_BOTAT | A1HA\_LOXIN | A1HB2\_LOXIN | A311\_LOXLA | AGAL\_HUMAN | ANAG\_HUMAN | AOAH\_MOUSE | CUTI1\_ASPOR | E0A7J0\_YARLL | ENPP2\_HUMAN | ENPP2\_RAT | FUCO\_HUMAN | GBA1\_HUMAN | I1SB18\_VIPAE | LICH\_HUMAN | LIP1\_DIURU | LIP2\_DIURU | LIP2\_GEOCN | LIP3\_DIURU | LIPA\_MOEAP | LIPB\_PSEA2 | LIPG\_CANLF | LIPG\_HUMAN | LIPP\_HORSE | LIPR1\_CANLF | LIPR1\_HUMAN | LIPR2\_HUMAN | LIPR2\_RAT | LIP\_THELA | MDLA\_PENCA | MDLA\_PENCY | NAGAB\_HUMAN | P78583\_ASPOZ | PA21B\_BOVIN | PA21B\_PIG | PA2A1\_BUNCE | PA2A1\_ECHCA | PA2A1\_NAJAT | PA2A1\_OPHHA | PA2A2\_NAJNA | PA2A2\_OPHHA | PA2A2\_TROCA | PA2A4\_NAJSG | PA2A5\_TRIST | PA2A7\_GLOHA | PA2A\_BOTJR | PA2A\_CROAT | PA2A\_DEIAC | PA2A\_GLOHA | PA2A\_NAJAT | PA2B1\_AGKPI | PA2B2\_BOTJR | PA2B2\_PROFL | PA2B3\_BOTAS | PA2B3\_BUNCE | PA2B5\_BUNCE | PA2B5\_NOTSC | PA2BA\_VIPAA | PA2BB\_GLOHA | PA2BB\_PSEAU | PA2BC\_VIPAA | PA2BD\_CRODU | PA2B\_BUNCE | PA2B\_NOTSC | PA2GA\_HUMAN | PA2GE\_HUMAN | PA2GX\_HUMAN | PA2H1\_AGKCL | PA2H1\_BOTBZ | PA2H1\_BOTJR | PA2H1\_BOTMO | PA2H1\_BOTPI | PA2H2\_BOTAS | PA2H2\_BOTMO | PA2H2\_BOTPI | PA2H2\_CERGO | PA2H3\_BOTPI | PA2HB\_AGKPI | PA2HB\_OXYSC | PA2HH\_TRIST | PA2HS\_ECHCA | PA2H\_BOTPA | PA2H\_DEIAC | PA2H\_PROMB | PA2N\_GLOHA | PA2\_APIME | PAG15\_HUMAN | PLA22\_ORYSJ | PPT1\_BOVIN | PPT1\_HUMAN | Q95KP4\_HORSE | Q9P8F7\_YARLL | SIA\_ASPFU | W6Q990\_PENRF | GO:0006026 | 0.0148975791433892 | 48/3222 | 38/552 | 3.05996855955504e-21 | 5.34882504210221e-18 | T | T | T | T | aminoglycan catabolic process | A0A0R4I979\_BRABE | A0A3B6UEQ2\_RHIMI | A0A7S6G7I6\_9PEZI | A5AB48\_ASPNC | A6PZ97\_SALSA | A9LI60\_BIOOC | A9ZSX9\_9BRYO | B9TU22\_GADMO | BGLR\_HUMAN | CDA\_COLLN | CDA\_EMENI | CHI1\_COCPS | CHI2\_HORVU | CHI2\_ORYSJ | CHI33\_TRIHA | CHI42\_TRIHA | CHI4\_CRYJA | CHIA\_HUMAN | CHIC\_ARATH | CHIC\_SECCE | CHIL3\_MOUSE | CHIT\_PUNGR | CHLY\_HEVBR | FUCO\_HUMAN | G3JPF7\_CORMM | HEXC\_OSTFU | HYAL1\_HUMAN | IDUA\_HUMAN | LYG\_STRCA | O81934\_CANEN | PGPSA\_DROME | PGRP1\_CAMDR | Q43576\_TOBAC | Q4AE59\_OSTFU | Q6WSR8\_PICAB | Q86RS6\_MANSE | Q8H0C9\_VIGUN | Q9FUH3\_VIGUS | GO:0009617 | 0.0291744258224705 | 94/3222 | 55/552 | 4.22392202147068e-20 | 7.38341569353076e-17 | T | T | T | T | response to bacterium | A6PZ97\_SALSA | ANG1\_BOVIN | ANG2\_MOUSE | ANG3\_MOUSE | ANG4\_MOUSE | ANGI\_MOUSE | B9TU22\_GADMO | CFAD\_MOUSE | ECP\_HUMAN | GRAA\_HUMAN | GRASS\_DROME | L8ICE9\_9CETA | LIPR2\_RAT | LYG\_STRCA | LYSC1\_ANAPL | LYSC1\_CANLF | LYSC1\_HORSE | LYSC2\_BOVIN | LYSC2\_ONCMY | LYSC\_COTJA | LYSC\_EQUAS | LYSC\_NUMME | LYSC\_OPIHO | LYSC\_PELSI | LYS\_BOMMO | LYS\_RUDPH | OXLA\_BOTAT | OXLA\_CALRH | OXLA\_GLOHA | PA21B\_BOVIN | PA2A\_BOTJR | PA2B3\_BOTAS | PA2GA\_HUMAN | PA2H1\_BOTBZ | PA2H1\_BOTJR | PA2H2\_BOTAS | PA2H2\_BOTMO | PA2H\_BOTPA | PERL\_BOVIN | PERL\_BUBBU | PERL\_CAPHI | PGPSA\_DROME | PGRP1\_CAMDR | POXA\_DICDI | PPA5\_HUMAN | PPA5\_RAT | Q5WRG2\_RAT | RNAS4\_HUMAN | RNAS4\_PIG | RNAS6\_HUMAN | RNS1B\_RAT | RNSL3\_DANRE | RNS\_BOVIN | TRFL\_BUBBU | TRFL\_HORSE | GO:0098542 | 0.0431409062693979 | 139/3222 | 68/552 | 7.74386633982923e-19 | 1.35362783620215e-15 | T | T | T | T | defense response to other organism | A6PZ97\_SALSA | ANG1\_BOVIN | ANG2\_MOUSE | ANG3\_MOUSE | ANG4\_MOUSE | ANGI\_MOUSE | B9TU22\_GADMO | CFAD\_MOUSE | CHI2\_ORYSJ | CHI4\_CRYJA | CHIC\_SECCE | E13B\_HORVU | ECP\_HUMAN | GRAC\_MOUSE | GRASS\_DROME | L8ICE9\_9CETA | LYG\_STRCA | LYSC1\_ANAPL | LYSC1\_CANLF | LYSC1\_HORSE | LYSC2\_BOVIN | LYSC2\_ONCMY | LYSC\_COTJA | LYSC\_EQUAS | LYSC\_NUMME | LYSC\_OPIHO | LYSC\_PELSI | LYS\_BOMMO | LYS\_RUDPH | OXLA\_BOTAT | OXLA\_CALRH | OXLA\_GLOHA | PA21B\_BOVIN | PA2A\_BOTJR | PA2B3\_BOTAS | PA2GA\_HUMAN | PA2GX\_HUMAN | PA2H1\_BOTBZ | PA2H1\_BOTJR | PA2H2\_BOTAS | PA2H2\_BOTMO | PA2H\_BOTPA | PER53\_ARATH | PERL\_BOVIN | PERL\_BUBBU | PERL\_CAPHI | PGPSA\_DROME | PGRP1\_CAMDR | POXA\_DICDI | PPA5\_HUMAN | PPA5\_RAT | PPAF1\_HOLDI | PRTN3\_HUMAN | Q5WRG2\_RAT | Q86RS6\_MANSE | RIP1\_HORVU | RIP1\_PHYAM | RIP2\_PHYAM | RIPA\_PHYAM | RNAS4\_HUMAN | RNAS4\_PIG | RNAS6\_HUMAN | RNS1B\_RAT | RNSL3\_DANRE | RNS\_BOVIN | RNT2\_HUMAN | TRFL\_BUBBU | TRFL\_HORSE | GO:0006022 | 0.0180012414649286 | 58/3222 | 40/552 | 1.39691422671188e-18 | 2.44180606829236e-15 | T | T | T | T | aminoglycan metabolic process | A0A0R4I979\_BRABE | A0A3B6UEQ2\_RHIMI | A0A7S6G7I6\_9PEZI | A5AB48\_ASPNC | A6PZ97\_SALSA | A9LI60\_BIOOC | A9ZSX9\_9BRYO | ANAG\_HUMAN | B9TU22\_GADMO | BGLR\_HUMAN | CDA\_COLLN | CDA\_EMENI | CHI1\_COCPS | CHI2\_HORVU | CHI2\_ORYSJ | CHI33\_TRIHA | CHI42\_TRIHA | CHI4\_CRYJA | CHIA\_HUMAN | CHIC\_ARATH | CHIC\_SECCE | CHIL3\_MOUSE | CHIT\_PUNGR | CHLY\_HEVBR | FUCO\_HUMAN | G3JPF7\_CORMM | HEXC\_OSTFU | HS3S1\_MOUSE | HYAL1\_HUMAN | IDUA\_HUMAN | LYG\_STRCA | O81934\_CANEN | PGPSA\_DROME | PGRP1\_CAMDR | Q43576\_TOBAC | Q4AE59\_OSTFU | Q6WSR8\_PICAB | Q86RS6\_MANSE | Q8H0C9\_VIGUN | Q9FUH3\_VIGUS | GO:0006032 | 0.00869025450031037 | 28/3222 | 26/552 | 1.9541475642281e-18 | 3.41584994227072e-15 | T | T | T | T | chitin catabolic process | A0A3B6UEQ2\_RHIMI | A5AB48\_ASPNC | A9LI60\_BIOOC | A9ZSX9\_9BRYO | CDA\_COLLN | CDA\_EMENI | CHI1\_COCPS | CHI2\_HORVU | CHI2\_ORYSJ | CHI33\_TRIHA | CHI42\_TRIHA | CHI4\_CRYJA | CHIA\_HUMAN | CHIC\_ARATH | CHIC\_SECCE | CHIL3\_MOUSE | CHIT\_PUNGR | CHLY\_HEVBR | G3JPF7\_CORMM | HEXC\_OSTFU | O81934\_CANEN | Q43576\_TOBAC | Q4AE59\_OSTFU | Q6WSR8\_PICAB | Q8H0C9\_VIGUN | Q9FUH3\_VIGUS | GO:0006030 | 0.00900062073246431 | 29/3222 | 26/552 | 1.58960595411276e-17 | 2.7786312077891e-14 | T | T | T | T | chitin metabolic process | A0A3B6UEQ2\_RHIMI | A5AB48\_ASPNC | A9LI60\_BIOOC | A9ZSX9\_9BRYO | CDA\_COLLN | CDA\_EMENI | CHI1\_COCPS | CHI2\_HORVU | CHI2\_ORYSJ | CHI33\_TRIHA | CHI42\_TRIHA | CHI4\_CRYJA | CHIA\_HUMAN | CHIC\_ARATH | CHIC\_SECCE | CHIL3\_MOUSE | CHIT\_PUNGR | CHLY\_HEVBR | G3JPF7\_CORMM | HEXC\_OSTFU | O81934\_CANEN | Q43576\_TOBAC | Q4AE59\_OSTFU | Q6WSR8\_PICAB | Q8H0C9\_VIGUN | Q9FUH3\_VIGUS | GO:0006644 | 0.0381750465549348 | 123/3222 | 61/552 | 2.46782273938373e-17 | 4.31375414844275e-14 | T | T | T | T | phospholipid metabolic process | A0A1L8D5Z7\_BOTAT | ENPP2\_HUMAN | ENPP2\_RAT | I1SB18\_VIPAE | LIPR2\_HUMAN | LIPR2\_RAT | PA21B\_BOVIN | PA21B\_PIG | PA2A1\_BUNCE | PA2A1\_ECHCA | PA2A1\_NAJAT | PA2A1\_OPHHA | PA2A2\_NAJNA | PA2A2\_OPHHA | PA2A2\_TROCA | PA2A4\_NAJSG | PA2A5\_TRIST | PA2A7\_GLOHA | PA2A\_BOTJR | PA2A\_CROAT | PA2A\_DEIAC | PA2A\_GLOHA | PA2A\_NAJAT | PA2B1\_AGKPI | PA2B2\_BOTJR | PA2B2\_PROFL | PA2B3\_BOTAS | PA2B3\_BUNCE | PA2B5\_BUNCE | PA2B5\_NOTSC | PA2BA\_VIPAA | PA2BB\_GLOHA | PA2BB\_PSEAU | PA2BC\_VIPAA | PA2BD\_CRODU | PA2B\_BUNCE | PA2B\_NOTSC | PA2GA\_HUMAN | PA2GE\_HUMAN | PA2GX\_HUMAN | PA2H1\_AGKCL | PA2H1\_BOTBZ | PA2H1\_BOTJR | PA2H1\_BOTMO | PA2H1\_BOTPI | PA2H2\_BOTAS | PA2H2\_BOTMO | PA2H2\_BOTPI | PA2H2\_CERGO | PA2H3\_BOTPI | PA2HB\_AGKPI | PA2HB\_OXYSC | PA2HH\_TRIST | PA2HS\_ECHCA | PA2H\_BOTPA | PA2H\_DEIAC | PA2H\_PROMB | PA2N\_GLOHA | PA2\_APIME | PAG15\_HUMAN | PLA22\_ORYSJ | GO:1901072 | 0.00931098696461825 | 30/3222 | 26/552 | 1.00323658823027e-16 | 1.75365755622652e-13 | T | T | T | T | glucosamine-containing compound catabolic process | A0A3B6UEQ2\_RHIMI | A5AB48\_ASPNC | A9LI60\_BIOOC | A9ZSX9\_9BRYO | CDA\_COLLN | CDA\_EMENI | CHI1\_COCPS | CHI2\_HORVU | CHI2\_ORYSJ | CHI33\_TRIHA | CHI42\_TRIHA | CHI4\_CRYJA | CHIA\_HUMAN | CHIC\_ARATH | CHIC\_SECCE | CHIL3\_MOUSE | CHIT\_PUNGR | CHLY\_HEVBR | G3JPF7\_CORMM | HEXC\_OSTFU | O81934\_CANEN | Q43576\_TOBAC | Q4AE59\_OSTFU | Q6WSR8\_PICAB | Q8H0C9\_VIGUN | Q9FUH3\_VIGUS | GO:0044419 | 0.0599006828057107 | 193/3222 | 80/552 | 1.3580554919665e-16 | 2.37388099995744e-13 | T | T | T | T | biological process involved in interspecies interaction between organisms | A1HA\_LOXIN | A1HB2\_LOXIN | A311\_LOXLA | A6PZ97\_SALSA | AGAL\_ORYSJ | ANG1\_BOVIN | ANG2\_MOUSE | ANG3\_MOUSE | ANG4\_MOUSE | ANGI\_MOUSE | B9TU22\_GADMO | CARP1\_CANAL | CFAD\_MOUSE | CHI2\_ORYSJ | CHI4\_CRYJA | CHIC\_SECCE | E13B\_HORVU | ECP\_HUMAN | EXG1\_CANAL | GRAA\_HUMAN | GRAC\_MOUSE | GRASS\_DROME | HYAL1\_HUMAN | L8ICE9\_9CETA | LIPR2\_RAT | LYG\_STRCA | LYSC1\_ANAPL | LYSC1\_CANLF | LYSC1\_HORSE | LYSC2\_BOVIN | LYSC2\_ONCMY | LYSC\_COTJA | LYSC\_EQUAS | LYSC\_NUMME | LYSC\_OPIHO | LYSC\_PELSI | LYS\_BOMMO | LYS\_RUDPH | OXLA\_BOTAT | OXLA\_CALRH | OXLA\_GLOHA | PA1\_VESBA | PA21B\_BOVIN | PA2A\_BOTJR | PA2B3\_BOTAS | PA2GA\_HUMAN | PA2GX\_HUMAN | PA2H1\_BOTBZ | PA2H1\_BOTJR | PA2H2\_BOTAS | PA2H2\_BOTMO | PA2H\_BOTPA | PER53\_ARATH | PERL\_BOVIN | PERL\_BUBBU | PERL\_CAPHI | PGH2\_HUMAN | PGPSA\_DROME | PGRP1\_CAMDR | POXA\_DICDI | PPA5\_HUMAN | PPA5\_RAT | PPAF1\_HOLDI | PRTN3\_HUMAN | Q5WRG2\_RAT | Q86RS6\_MANSE | RIP1\_HORVU | RIP1\_PHYAM | RIP2\_PHYAM | RIPA\_PHYAM | RNAS4\_HUMAN | RNAS4\_PIG | RNAS6\_HUMAN | RNS1B\_RAT | RNSL3\_DANRE | RNS\_BOVIN | RNT2\_HUMAN | TRFL\_BUBBU | TRFL\_HORSE | TTHY\_CHICK | GO:0051707 | 0.0567970204841713 | 183/3222 | 75/552 | 2.93137790102514e-15 | 5.12404857099194e-12 | T | T | T | T | response to other organism | A6PZ97\_SALSA | AGAL\_ORYSJ | ANG1\_BOVIN | ANG2\_MOUSE | ANG3\_MOUSE | ANG4\_MOUSE | ANGI\_MOUSE | B9TU22\_GADMO | CARP1\_CANAL | CFAD\_MOUSE | CHI2\_ORYSJ | CHI4\_CRYJA | CHIC\_SECCE | E13B\_HORVU | ECP\_HUMAN | GRAA\_HUMAN | GRAC\_MOUSE | GRASS\_DROME | HYAL1\_HUMAN | L8ICE9\_9CETA | LIPR2\_RAT | LYG\_STRCA | LYSC1\_ANAPL | LYSC1\_CANLF | LYSC1\_HORSE | LYSC2\_BOVIN | LYSC2\_ONCMY | LYSC\_COTJA | LYSC\_EQUAS | LYSC\_NUMME | LYSC\_OPIHO | LYSC\_PELSI | LYS\_BOMMO | LYS\_RUDPH | OXLA\_BOTAT | OXLA\_CALRH | OXLA\_GLOHA | PA21B\_BOVIN | PA2A\_BOTJR | PA2B3\_BOTAS | PA2GA\_HUMAN | PA2GX\_HUMAN | PA2H1\_BOTBZ | PA2H1\_BOTJR | PA2H2\_BOTAS | PA2H2\_BOTMO | PA2H\_BOTPA | PER53\_ARATH | PERL\_BOVIN | PERL\_BUBBU | PERL\_CAPHI | PGH2\_HUMAN | PGPSA\_DROME | PGRP1\_CAMDR | POXA\_DICDI | PPA5\_HUMAN | PPA5\_RAT | PPAF1\_HOLDI | PRTN3\_HUMAN | Q5WRG2\_RAT | Q86RS6\_MANSE | RIP1\_HORVU | RIP1\_PHYAM | RIP2\_PHYAM | RIPA\_PHYAM | RNAS4\_HUMAN | RNAS4\_PIG | RNAS6\_HUMAN | RNS1B\_RAT | RNSL3\_DANRE | RNS\_BOVIN | RNT2\_HUMAN | TRFL\_BUBBU | TRFL\_HORSE | TTHY\_CHICK | GO:0050830 | 0.00713842333954066 | 23/3222 | 21/552 | 1.05813640769175e-14 | 1.84962244064518e-11 | T | T | T | T | defense response to Gram-positive bacterium | ANG1\_BOVIN | ANG2\_MOUSE | ANG3\_MOUSE | ANG4\_MOUSE | ANGI\_MOUSE | ECP\_HUMAN | GRASS\_DROME | LYSC2\_BOVIN | OXLA\_BOTAT | PA21B\_BOVIN | PA2GA\_HUMAN | PGPSA\_DROME | PPA5\_HUMAN | PPA5\_RAT | Q5WRG2\_RAT | RNAS4\_HUMAN | RNAS4\_PIG | RNAS6\_HUMAN | RNS1B\_RAT | RNSL3\_DANRE | RNS\_BOVIN | GO:0071702 | 0.0397268777157045 | 128/3222 | 58/552 | 2.88631265716168e-14 | 5.04527452471861e-11 | T | T | T | T | organic substance transport | A0A1L8D5Z7\_BOTAT | I1SB18\_VIPAE | PA21B\_BOVIN | PA2A1\_BUNCE | PA2A1\_ECHCA | PA2A1\_NAJAT | PA2A1\_OPHHA | PA2A2\_NAJNA | PA2A2\_OPHHA | PA2A2\_TROCA | PA2A4\_NAJSG | PA2A5\_TRIST | PA2A7\_GLOHA | PA2A\_BOTJR | PA2A\_CROAT | PA2A\_DEIAC | PA2A\_GLOHA | PA2A\_NAJAT | PA2B1\_AGKPI | PA2B2\_BOTJR | PA2B2\_PROFL | PA2B3\_BOTAS | PA2B3\_BUNCE | PA2B5\_BUNCE | PA2B5\_NOTSC | PA2BA\_VIPAA | PA2BB\_GLOHA | PA2BB\_PSEAU | PA2BC\_VIPAA | PA2BD\_CRODU | PA2B\_BUNCE | PA2B\_NOTSC | PA2GA\_HUMAN | PA2GE\_HUMAN | PA2GX\_HUMAN | PA2H1\_AGKCL | PA2H1\_BOTBZ | PA2H1\_BOTJR | PA2H1\_BOTMO | PA2H1\_BOTPI | PA2H2\_BOTAS | PA2H2\_BOTMO | PA2H2\_BOTPI | PA2H2\_CERGO | PA2H3\_BOTPI | PA2HB\_AGKPI | PA2HB\_OXYSC | PA2HH\_TRIST | PA2HS\_ECHCA | PA2H\_BOTPA | PA2H\_DEIAC | PA2H\_PROMB | PA2N\_GLOHA | PA2\_APIME | PGH2\_HUMAN | PLA22\_ORYSJ | PPT1\_BOVIN | PPT1\_HUMAN | GO:0141061 | 0.0130353817504655 | 42/3222 | 29/552 | 8.78111662394986e-14 | 1.53493918586643e-10 | T | T | T | T | disruption of cell in another organism | A1HA\_LOXIN | A1HB2\_LOXIN | A311\_LOXLA | A6PZ97\_SALSA | B9TU22\_GADMO | CHI4\_CRYJA | CHIC\_SECCE | GRAA\_HUMAN | GRAC\_MOUSE | LYG\_STRCA | LYSC1\_ANAPL | LYSC1\_CANLF | LYSC1\_HORSE | LYSC2\_BOVIN | LYSC2\_ONCMY | LYSC\_COTJA | LYSC\_EQUAS | LYSC\_NUMME | LYSC\_OPIHO | LYSC\_PELSI | LYS\_BOMMO | LYS\_RUDPH | OXLA\_BOTAT | OXLA\_CALRH | OXLA\_GLOHA | PA1\_VESBA | PA2GA\_HUMAN | PA2H2\_BOTMO | RIP1\_HORVU | GO:0141060 | 0.0130353817504655 | 42/3222 | 29/552 | 8.78111662394986e-14 | 1.53493918586643e-10 | T | T | T | T | disruption of anatomical structure in another organism | A1HA\_LOXIN | A1HB2\_LOXIN | A311\_LOXLA | A6PZ97\_SALSA | B9TU22\_GADMO | CHI4\_CRYJA | CHIC\_SECCE | GRAA\_HUMAN | GRAC\_MOUSE | LYG\_STRCA | LYSC1\_ANAPL | LYSC1\_CANLF | LYSC1\_HORSE | LYSC2\_BOVIN | LYSC2\_ONCMY | LYSC\_COTJA | LYSC\_EQUAS | LYSC\_NUMME | LYSC\_OPIHO | LYSC\_PELSI | LYS\_BOMMO | LYS\_RUDPH | OXLA\_BOTAT | OXLA\_CALRH | OXLA\_GLOHA | PA1\_VESBA | PA2GA\_HUMAN | PA2H2\_BOTMO | RIP1\_HORVU | GO:0031640 | 0.0130353817504655 | 42/3222 | 29/552 | 8.78111662394986e-14 | 1.53493918586643e-10 | T | T | T | T | killing of cells of another organism | A1HA\_LOXIN | A1HB2\_LOXIN | A311\_LOXLA | A6PZ97\_SALSA | B9TU22\_GADMO | CHI4\_CRYJA | CHIC\_SECCE | GRAA\_HUMAN | GRAC\_MOUSE | LYG\_STRCA | LYSC1\_ANAPL | LYSC1\_CANLF | LYSC1\_HORSE | LYSC2\_BOVIN | LYSC2\_ONCMY | LYSC\_COTJA | LYSC\_EQUAS | LYSC\_NUMME | LYSC\_OPIHO | LYSC\_PELSI | LYS\_BOMMO | LYS\_RUDPH | OXLA\_BOTAT | OXLA\_CALRH | OXLA\_GLOHA | PA1\_VESBA | PA2GA\_HUMAN | PA2H2\_BOTMO | RIP1\_HORVU | GO:0001906 | 0.0142768466790813 | 46/3222 | 30/552 | 3.33378938079395e-13 | 5.82746383762783e-10 | T | T | T | T | cell killing | A1HA\_LOXIN | A1HB2\_LOXIN | A311\_LOXLA | A6PZ97\_SALSA | B9TU22\_GADMO | CATH\_HUMAN | CHI4\_CRYJA | CHIC\_SECCE | GRAA\_HUMAN | GRAC\_MOUSE | LYG\_STRCA | LYSC1\_ANAPL | LYSC1\_CANLF | LYSC1\_HORSE | LYSC2\_BOVIN | LYSC2\_ONCMY | LYSC\_COTJA | LYSC\_EQUAS | LYSC\_NUMME | LYSC\_OPIHO | LYSC\_PELSI | LYS\_BOMMO | LYS\_RUDPH | OXLA\_BOTAT | OXLA\_CALRH | OXLA\_GLOHA | PA1\_VESBA | PA2GA\_HUMAN | PA2H2\_BOTMO | RIP1\_HORVU | GO:0043207 | 0.0645561762880199 | 208/3222 | 77/552 | 8.08684165451112e-13 | 1.41357992120854e-09 | T | T | T | T | response to external biotic stimulus | A6PZ97\_SALSA | AGAL\_ORYSJ | ANAG\_HUMAN | ANG1\_BOVIN | ANG2\_MOUSE | ANG3\_MOUSE | ANG4\_MOUSE | ANGI\_MOUSE | B9TU22\_GADMO | CARP1\_CANAL | CFAD\_MOUSE | CHI2\_ORYSJ | CHI4\_CRYJA | CHIC\_SECCE | E13B\_HORVU | ECP\_HUMAN | GRAA\_HUMAN | GRAC\_MOUSE | GRASS\_DROME | HYAL1\_HUMAN | L8ICE9\_9CETA | LIPR2\_RAT | LYG\_STRCA | LYSC1\_ANAPL | LYSC1\_CANLF | LYSC1\_HORSE | LYSC2\_BOVIN | LYSC2\_ONCMY | LYSC\_COTJA | LYSC\_EQUAS | LYSC\_NUMME | LYSC\_OPIHO | LYSC\_PELSI | LYS\_BOMMO | LYS\_RUDPH | OXLA\_BOTAT | OXLA\_CALRH | OXLA\_GLOHA | PA21B\_BOVIN | PA2A\_BOTJR | PA2B3\_BOTAS | PA2GA\_HUMAN | PA2GX\_HUMAN | PA2H1\_BOTBZ | PA2H1\_BOTJR | PA2H2\_BOTAS | PA2H2\_BOTMO | PA2H\_BOTPA | PER53\_ARATH | PERL\_BOVIN | PERL\_BUBBU | PERL\_CAPHI | PGH2\_HUMAN | PGPSA\_DROME | PGRP1\_CAMDR | POXA\_DICDI | PPA5\_HUMAN | PPA5\_RAT | PPAF1\_HOLDI | PRTN3\_HUMAN | Q5WRG2\_RAT | Q86RS6\_MANSE | RENI\_RAT | RIP1\_HORVU | RIP1\_PHYAM | RIP2\_PHYAM | RIPA\_PHYAM | RNAS4\_HUMAN | RNAS4\_PIG | RNAS6\_HUMAN | RNS1B\_RAT | RNSL3\_DANRE | RNS\_BOVIN | RNT2\_HUMAN | TRFL\_BUBBU | TRFL\_HORSE | TTHY\_CHICK | GO:0006508 | 0.0549348230912477 | 177/3222 | 69/552 | 8.20748226834369e-13 | 1.43466790050648e-09 | T | T | T | T | proteolysis | A0A6P6YAT6\_DERPT | A0NFU8\_ANOGA | ANAG\_HUMAN | B4F320\_LIMPO | CARP1\_CANAL | CARP2\_CANAX | CARP\_RHIPU | CATD\_RAT | CATH\_HUMAN | CATLL\_FASHE | CBPA1\_PIG | CBPD\_LOPSP | CBPN\_HUMAN | CEL2A\_PIG | CFAD\_MOUSE | CHYM\_CAMDR | COGS\_HYPLI | CUCM1\_CUCME | CYSP\_BLOTA | D6XHE1\_TRYB2 | DDN1\_BOVIN | DPP2\_HUMAN | EGFB2\_MOUSE | ERVB\_TABDI | G3I1H5\_CRIGR | GBA1\_HUMAN | GRAA\_HUMAN | GRAC\_MOUSE | GRAK\_HUMAN | GRASS\_DROME | HE12\_DANRE | J7LCB0\_DEIAC | KLK10\_HUMAN | KLK1\_HUMAN | KLK2\_HORSE | KLK2\_HUMAN | KLK7\_HUMAN | KLK7\_MOUSE | KLK8\_MOUSE | LAPA\_ASPOR | LGMN\_MOUSE | MAN12\_PENCI | MCPT2\_RAT | MMP1\_PIG | O97389\_HELAM | PCP\_HUMAN | PEPA\_ASPPH | PGPSA\_DROME | PPAF1\_HOLDI | PRS57\_HUMAN | PRTN3\_HUMAN | Q69G21\_TENMO | Q6R7Z5\_9TRYP | Q7YXL2\_TENMO | RENI\_RAT | TPP1\_HUMAN | TRFL\_BUBBU | TRFL\_HORSE | TRY1\_GADMO | TRY3\_SALSA | TRYB2\_HUMAN | VM11\_BOTMO | VM12\_CROAD | VM1A3\_DEIAC | VM1BI\_BOTMO | VM1T1\_PROMU | VM1T2\_PROFL | VSPP\_DEIAC | VSPSX\_GLOSA | GO:0046348 | 0.0114835505896958 | 37/3222 | 26/552 | 9.35088256820485e-13 | 1.63453427292221e-09 | T | T | T | T | amino sugar catabolic process | A0A3B6UEQ2\_RHIMI | A5AB48\_ASPNC | A9LI60\_BIOOC | A9ZSX9\_9BRYO | CDA\_COLLN | CDA\_EMENI | CHI1\_COCPS | CHI2\_HORVU | CHI2\_ORYSJ | CHI33\_TRIHA | CHI42\_TRIHA | CHI4\_CRYJA | CHIA\_HUMAN | CHIC\_ARATH | CHIC\_SECCE | CHIL3\_MOUSE | CHIT\_PUNGR | CHLY\_HEVBR | G3JPF7\_CORMM | HEXC\_OSTFU | O81934\_CANEN | Q43576\_TOBAC | Q4AE59\_OSTFU | Q6WSR8\_PICAB | Q8H0C9\_VIGUN | Q9FUH3\_VIGUS | GO:1901071 | 0.0114835505896958 | 37/3222 | 26/552 | 9.35088256820485e-13 | 1.63453427292221e-09 | T | T | T | T | glucosamine-containing compound metabolic process | A0A3B6UEQ2\_RHIMI | A5AB48\_ASPNC | A9LI60\_BIOOC | A9ZSX9\_9BRYO | CDA\_COLLN | CDA\_EMENI | CHI1\_COCPS | CHI2\_HORVU | CHI2\_ORYSJ | CHI33\_TRIHA | CHI42\_TRIHA | CHI4\_CRYJA | CHIA\_HUMAN | CHIC\_ARATH | CHIC\_SECCE | CHIL3\_MOUSE | CHIT\_PUNGR | CHLY\_HEVBR | G3JPF7\_CORMM | HEXC\_OSTFU | O81934\_CANEN | Q43576\_TOBAC | Q4AE59\_OSTFU | Q6WSR8\_PICAB | Q8H0C9\_VIGUN | Q9FUH3\_VIGUS | GO:0045488 | 0.00465549348230913 | 15/3222 | 15/552 | 2.74178909890702e-12 | 4.79264734488947e-09 | T | T | T | T | pectin metabolic process | A0A6M9BP13\_9EURO | A1E266\_9PEZI | ADPG2\_ARATH | AXHA2\_EMENI | FAEA\_ASPNG | G3YAL0\_ASPNA | GANA\_EMENI | P79074\_9AGAR | PGLR1\_ASPAC | PGLR1\_ASPNG | PGLR\_GIBFU | PLY1\_JUNAS | PME\_DAUCA | PME\_SITOR | XGHA\_ASPTU | GO:0010393 | 0.00465549348230913 | 15/3222 | 15/552 | 2.74178909890702e-12 | 4.79264734488947e-09 | T | T | T | T | galacturonan metabolic process | A0A6M9BP13\_9EURO | A1E266\_9PEZI | ADPG2\_ARATH | AXHA2\_EMENI | FAEA\_ASPNG | G3YAL0\_ASPNA | GANA\_EMENI | P79074\_9AGAR | PGLR1\_ASPAC | PGLR1\_ASPNG | PGLR\_GIBFU | PLY1\_JUNAS | PME\_DAUCA | PME\_SITOR | XGHA\_ASPTU | GO:0045490 | 0.00465549348230913 | 15/3222 | 15/552 | 2.74178909890702e-12 | 4.79264734488947e-09 | T | T | T | T | pectin catabolic process | A0A6M9BP13\_9EURO | A1E266\_9PEZI | ADPG2\_ARATH | AXHA2\_EMENI | FAEA\_ASPNG | G3YAL0\_ASPNA | GANA\_EMENI | P79074\_9AGAR | PGLR1\_ASPAC | PGLR1\_ASPNG | PGLR\_GIBFU | PLY1\_JUNAS | PME\_DAUCA | PME\_SITOR | XGHA\_ASPTU | GO:0071555 | 0.0111731843575419 | 36/3222 | 25/552 | 3.98621526160384e-12 | 6.96790427728352e-09 | T | T | T | T | cell wall organization | A0A6M9BP13\_9EURO | A1E266\_9PEZI | ADPG2\_ARATH | CARP1\_CANAL | CDA\_COLLN | CDA\_EMENI | ENG1\_RHIMI | EXG1\_CANAL | EXG1\_YEAST | G3YAL0\_ASPNA | GANA\_EMENI | P79074\_9AGAR | PELA\_ASPNG | PELB\_ASPNG | PGLR1\_ASPAC | PGLR1\_ASPNG | PGLR\_GIBFU | PME\_DAUCA | PME\_SITOR | Q07524\_TROMA | Q9LYJ5\_ARATH | RGLA\_ASPAC | RHGA\_ASPAC | XGHA\_ASPTU | XTH34\_POPPZ | GO:0009607 | 0.0664183736809435 | 214/3222 | 77/552 | 4.46270256849151e-12 | 7.80080408972316e-09 | T | T | T | T | response to biotic stimulus | A6PZ97\_SALSA | AGAL\_ORYSJ | ANAG\_HUMAN | ANG1\_BOVIN | ANG2\_MOUSE | ANG3\_MOUSE | ANG4\_MOUSE | ANGI\_MOUSE | B9TU22\_GADMO | CARP1\_CANAL | CFAD\_MOUSE | CHI2\_ORYSJ | CHI4\_CRYJA | CHIC\_SECCE | E13B\_HORVU | ECP\_HUMAN | GRAA\_HUMAN | GRAC\_MOUSE | GRASS\_DROME | HYAL1\_HUMAN | L8ICE9\_9CETA | LIPR2\_RAT | LYG\_STRCA | LYSC1\_ANAPL | LYSC1\_CANLF | LYSC1\_HORSE | LYSC2\_BOVIN | LYSC2\_ONCMY | LYSC\_COTJA | LYSC\_EQUAS | LYSC\_NUMME | LYSC\_OPIHO | LYSC\_PELSI | LYS\_BOMMO | LYS\_RUDPH | OXLA\_BOTAT | OXLA\_CALRH | OXLA\_GLOHA | PA21B\_BOVIN | PA2A\_BOTJR | PA2B3\_BOTAS | PA2GA\_HUMAN | PA2GX\_HUMAN | PA2H1\_BOTBZ | PA2H1\_BOTJR | PA2H2\_BOTAS | PA2H2\_BOTMO | PA2H\_BOTPA | PER53\_ARATH | PERL\_BOVIN | PERL\_BUBBU | PERL\_CAPHI | PGH2\_HUMAN | PGPSA\_DROME | PGRP1\_CAMDR | POXA\_DICDI | PPA5\_HUMAN | PPA5\_RAT | PPAF1\_HOLDI | PRTN3\_HUMAN | Q5WRG2\_RAT | Q86RS6\_MANSE | RENI\_RAT | RIP1\_HORVU | RIP1\_PHYAM | RIP2\_PHYAM | RIPA\_PHYAM | RNAS4\_HUMAN | RNAS4\_PIG | RNAS6\_HUMAN | RNS1B\_RAT | RNSL3\_DANRE | RNS\_BOVIN | RNT2\_HUMAN | TRFL\_BUBBU | TRFL\_HORSE | TTHY\_CHICK | GO:0045229 | 0.0130353817504655 | 42/3222 | 27/552 | 8.90688890338754e-12 | 1.55692418031214e-08 | T | T | T | T | external encapsulating structure organization | A0A6M9BP13\_9EURO | A1E266\_9PEZI | ADPG2\_ARATH | CARP1\_CANAL | CDA\_COLLN | CDA\_EMENI | ENG1\_RHIMI | EXG1\_CANAL | EXG1\_YEAST | G3YAL0\_ASPNA | GANA\_EMENI | KLK7\_HUMAN | MMP1\_PIG | P79074\_9AGAR | PELA\_ASPNG | PELB\_ASPNG | PGLR1\_ASPAC | PGLR1\_ASPNG | PGLR\_GIBFU | PME\_DAUCA | PME\_SITOR | Q07524\_TROMA | Q9LYJ5\_ARATH | RGLA\_ASPAC | RHGA\_ASPAC | XGHA\_ASPTU | XTH34\_POPPZ | GO:0071554 | 0.0121042830540037 | 39/3222 | 25/552 | 5.96385621976181e-11 | 1.04248206721436e-07 | T | T | T | T | cell wall organization or biogenesis | A0A6M9BP13\_9EURO | A1E266\_9PEZI | ADPG2\_ARATH | CARP1\_CANAL | CDA\_COLLN | CDA\_EMENI | ENG1\_RHIMI | EXG1\_CANAL | EXG1\_YEAST | G3YAL0\_ASPNA | GANA\_EMENI | P79074\_9AGAR | PELA\_ASPNG | PELB\_ASPNG | PGLR1\_ASPAC | PGLR1\_ASPNG | PGLR\_GIBFU | PME\_DAUCA | PME\_SITOR | Q07524\_TROMA | Q9LYJ5\_ARATH | RGLA\_ASPAC | RHGA\_ASPAC | XGHA\_ASPTU | XTH34\_POPPZ | GO:0019731 | 0.00465549348230913 | 15/3222 | 14/552 | 2.06847092837228e-10 | 3.61568718279475e-07 | T | T | T | T | antibacterial humoral response | ANG1\_BOVIN | ANG2\_MOUSE | ANG3\_MOUSE | ANG4\_MOUSE | ANGI\_MOUSE | ECP\_HUMAN | PA21B\_BOVIN | PERL\_BOVIN | PERL\_BUBBU | PERL\_CAPHI | Q5WRG2\_RAT | RNAS6\_HUMAN | TRFL\_BUBBU | TRFL\_HORSE | GO:0046274 | 0.00434512725015518 | 14/3222 | 13/552 | 1.15014935375124e-09 | 2.01046107035716e-06 | T | T | T | T | lignin catabolic process | GCE2\_MYCTT | GCE\_CERUI | GCE\_HYPJQ | LAC1\_MELAO | LAC1\_TRAMX | LAC2\_TRAVE | LIG2\_PHACH | LIG4\_PHACH | LIG8\_PHACH | PEM1\_PHACH | Q60FD2\_9APHY | VPL1\_PLEER | VPL2\_PLEER | GO:0019730 | 0.00589695841092489 | 19/3222 | 15/552 | 5.37014705361045e-09 | 9.38701704971106e-06 | T | T | T | T | antimicrobial humoral response | ANG1\_BOVIN | ANG2\_MOUSE | ANG3\_MOUSE | ANG4\_MOUSE | ANGI\_MOUSE | ECP\_HUMAN | PA21B\_BOVIN | PERL\_BOVIN | PERL\_BUBBU | PERL\_CAPHI | PRTN3\_HUMAN | Q5WRG2\_RAT | RNAS6\_HUMAN | TRFL\_BUBBU | TRFL\_HORSE | GO:0006810 | 0.0620732464307883 | 200/3222 | 65/552 | 3.0328608056064e-08 | 5.30144068819999e-05 | T | T | T | T | transport | A0A1L8D5Z7\_BOTAT | CERU\_RAT | I1SB18\_VIPAE | PA21B\_BOVIN | PA2A1\_BUNCE | PA2A1\_ECHCA | PA2A1\_NAJAT | PA2A1\_OPHHA | PA2A2\_NAJNA | PA2A2\_OPHHA | PA2A2\_TROCA | PA2A4\_NAJSG | PA2A5\_TRIST | PA2A7\_GLOHA | PA2A\_BOTJR | PA2A\_CROAT | PA2A\_DEIAC | PA2A\_GLOHA | PA2A\_NAJAT | PA2B1\_AGKPI | PA2B2\_BOTJR | PA2B2\_PROFL | PA2B3\_BOTAS | PA2B3\_BUNCE | PA2B5\_BUNCE | PA2B5\_NOTSC | PA2BA\_VIPAA | PA2BB\_GLOHA | PA2BB\_PSEAU | PA2BC\_VIPAA | PA2BD\_CRODU | PA2B\_BUNCE | PA2B\_NOTSC | PA2GA\_HUMAN | PA2GE\_HUMAN | PA2GX\_HUMAN | PA2H1\_AGKCL | PA2H1\_BOTBZ | PA2H1\_BOTJR | PA2H1\_BOTMO | PA2H1\_BOTPI | PA2H2\_BOTAS | PA2H2\_BOTMO | PA2H2\_BOTPI | PA2H2\_CERGO | PA2H3\_BOTPI | PA2HB\_AGKPI | PA2HB\_OXYSC | PA2HH\_TRIST | PA2HS\_ECHCA | PA2H\_BOTPA | PA2H\_DEIAC | PA2H\_PROMB | PA2N\_GLOHA | PA2\_APIME | PGH2\_HUMAN | PLA22\_ORYSJ | PPA5\_PIG | PPT1\_BOVIN | PPT1\_HUMAN | PTGDS\_HUMAN | PTGDS\_MOUSE | TRFL\_BUBBU | TRFL\_HORSE | TTHY\_CHICK | GO:0006959 | 0.00713842333954066 | 23/3222 | 16/552 | 3.40034082487552e-08 | 5.94379576188241e-05 | T | T | T | T | humoral immune response | ANG1\_BOVIN | ANG2\_MOUSE | ANG3\_MOUSE | ANG4\_MOUSE | ANGI\_MOUSE | CFAD\_MOUSE | ECP\_HUMAN | PA21B\_BOVIN | PERL\_BOVIN | PERL\_BUBBU | PERL\_CAPHI | PRTN3\_HUMAN | Q5WRG2\_RAT | RNAS6\_HUMAN | TRFL\_BUBBU | TRFL\_HORSE | GO:0051179 | 0.0679702048417132 | 219/3222 | 69/552 | 4.51996534208049e-08 | 7.9008994179567e-05 | T | T | T | T | localization | A0A1L8D5Z7\_BOTAT | ANAG\_HUMAN | CERU\_RAT | GBA1\_HUMAN | I1SB18\_VIPAE | LICH\_HUMAN | PA21B\_BOVIN | PA2A1\_BUNCE | PA2A1\_ECHCA | PA2A1\_NAJAT | PA2A1\_OPHHA | PA2A2\_NAJNA | PA2A2\_OPHHA | PA2A2\_TROCA | PA2A4\_NAJSG | PA2A5\_TRIST | PA2A7\_GLOHA | PA2A\_BOTJR | PA2A\_CROAT | PA2A\_DEIAC | PA2A\_GLOHA | PA2A\_NAJAT | PA2B1\_AGKPI | PA2B2\_BOTJR | PA2B2\_PROFL | PA2B3\_BOTAS | PA2B3\_BUNCE | PA2B5\_BUNCE | PA2B5\_NOTSC | PA2BA\_VIPAA | PA2BB\_GLOHA | PA2BB\_PSEAU | PA2BC\_VIPAA | PA2BD\_CRODU | PA2B\_BUNCE | PA2B\_NOTSC | PA2GA\_HUMAN | PA2GE\_HUMAN | PA2GX\_HUMAN | PA2H1\_AGKCL | PA2H1\_BOTBZ | PA2H1\_BOTJR | PA2H1\_BOTMO | PA2H1\_BOTPI | PA2H2\_BOTAS | PA2H2\_BOTMO | PA2H2\_BOTPI | PA2H2\_CERGO | PA2H3\_BOTPI | PA2HB\_AGKPI | PA2HB\_OXYSC | PA2HH\_TRIST | PA2HS\_ECHCA | PA2H\_BOTPA | PA2H\_DEIAC | PA2H\_PROMB | PA2N\_GLOHA | PA2\_APIME | PGH2\_HUMAN | PLA22\_ORYSJ | PPA5\_PIG | PPT1\_BOVIN | PPT1\_HUMAN | PTGDS\_HUMAN | PTGDS\_MOUSE | TPP1\_HUMAN | TRFL\_BUBBU | TRFL\_HORSE | TTHY\_CHICK | GO:0051234 | 0.0633147113594041 | 204/3222 | 65/552 | 7.2014611701029e-08 | 0.000125881541253399 | T | T | T | T | establishment of localization | A0A1L8D5Z7\_BOTAT | CERU\_RAT | I1SB18\_VIPAE | PA21B\_BOVIN | PA2A1\_BUNCE | PA2A1\_ECHCA | PA2A1\_NAJAT | PA2A1\_OPHHA | PA2A2\_NAJNA | PA2A2\_OPHHA | PA2A2\_TROCA | PA2A4\_NAJSG | PA2A5\_TRIST | PA2A7\_GLOHA | PA2A\_BOTJR | PA2A\_CROAT | PA2A\_DEIAC | PA2A\_GLOHA | PA2A\_NAJAT | PA2B1\_AGKPI | PA2B2\_BOTJR | PA2B2\_PROFL | PA2B3\_BOTAS | PA2B3\_BUNCE | PA2B5\_BUNCE | PA2B5\_NOTSC | PA2BA\_VIPAA | PA2BB\_GLOHA | PA2BB\_PSEAU | PA2BC\_VIPAA | PA2BD\_CRODU | PA2B\_BUNCE | PA2B\_NOTSC | PA2GA\_HUMAN | PA2GE\_HUMAN | PA2GX\_HUMAN | PA2H1\_AGKCL | PA2H1\_BOTBZ | PA2H1\_BOTJR | PA2H1\_BOTMO | PA2H1\_BOTPI | PA2H2\_BOTAS | PA2H2\_BOTMO | PA2H2\_BOTPI | PA2H2\_CERGO | PA2H3\_BOTPI | PA2HB\_AGKPI | PA2HB\_OXYSC | PA2HH\_TRIST | PA2HS\_ECHCA | PA2H\_BOTPA | PA2H\_DEIAC | PA2H\_PROMB | PA2N\_GLOHA | PA2\_APIME | PGH2\_HUMAN | PLA22\_ORYSJ | PPA5\_PIG | PPT1\_BOVIN | PPT1\_HUMAN | PTGDS\_HUMAN | PTGDS\_MOUSE | TRFL\_BUBBU | TRFL\_HORSE | TTHY\_CHICK | GO:0002376 | 0.037243947858473 | 120/3222 | 43/552 | 3.93717956729669e-07 | 0.000688218988363462 | T | T | T | T | immune system process | A0A0R4I979\_BRABE | ANAG\_HUMAN | ANG1\_BOVIN | ANG2\_MOUSE | ANG3\_MOUSE | ANG4\_MOUSE | ANGI\_MOUSE | CATH\_HUMAN | CFAD\_MOUSE | CHIA\_HUMAN | DNAS1\_HUMAN | DNSL3\_HUMAN | DOPO\_HUMAN | ECP\_HUMAN | ENPP2\_HUMAN | ENPP2\_RAT | GBA1\_HUMAN | GILT\_MOUSE | GRAA\_HUMAN | GRAC\_MOUSE | GRASS\_DROME | K7CID1\_PANTR | LICH\_HUMAN | PA21B\_BOVIN | PA21B\_PIG | PA2GX\_HUMAN | PERL\_BOVIN | PERL\_BUBBU | PERL\_CAPHI | PGPSA\_DROME | PGRP1\_CAMDR | POXA\_DICDI | PPAF1\_HOLDI | PRTN3\_HUMAN | PTGDS\_HUMAN | PTGDS\_MOUSE | Q5WRG2\_RAT | Q86RS6\_MANSE | RNAS6\_HUMAN | RNSL3\_DANRE | RNT2\_HUMAN | TRFL\_BUBBU | TRFL\_HORSE | GO:0042744 | 0.0201738050900062 | 65/3222 | 28/552 | 6.5425368831648e-07 | 0.00114363544717721 | T | T | T | T | hydrogen peroxide catabolic process | A0A087WNH2\_FICBE | A0A0A0Y4H8\_TRAFO | A0A1S4NYF8\_PANVG | A0A3L6SKP5\_PANMI | APO1\_CYCAE | CAT3\_NEUCR | D1MPT2\_ROYRE | GPX3\_HUMAN | K7N5L9\_RAPSA | KATG2\_MAGO7 | LIG4\_PHACH | LIG8\_PHACH | O22443\_SOYBN | PEM1\_PHACH | PER1A\_ARMRU | PER1\_ARAHY | PER1\_SORBI | PER53\_ARATH | PER59\_ARATH | PERL\_BOVIN | PERL\_BUBBU | PERL\_CAPHI | PER\_ARTRA | PER\_COPCI | POXA\_DICDI | Q40069\_HORVU | VPL1\_PLEER | VPL2\_PLEER | GO:0019732 | 0.00248292985723153 | 8/3222 | 8/552 | 7.11468232016806e-07 | 0.00124364646956538 | T | T | T | T | antifungal humoral response | ANG2\_MOUSE | ANG3\_MOUSE | ANG4\_MOUSE | ANGI\_MOUSE | PERL\_CAPHI | Q5WRG2\_RAT | TRFL\_BUBBU | TRFL\_HORSE | GO:1901136 | 0.0459342023587834 | 148/3222 | 49/552 | 9.43666552900265e-07 | 0.00164952913446966 | T | T | T | T | carbohydrate derivative catabolic process | A0A0R4I979\_BRABE | A0A3B6UEQ2\_RHIMI | A0A7S6G7I6\_9PEZI | A5AB48\_ASPNC | A6PZ97\_SALSA | A9LI60\_BIOOC | A9ZSX9\_9BRYO | ADA2\_HUMAN | AGAL\_HUMAN | ANAG\_HUMAN | AOAH\_MOUSE | B9TU22\_GADMO | BGLR\_HUMAN | CDA\_COLLN | CDA\_EMENI | CHI1\_COCPS | CHI2\_HORVU | CHI2\_ORYSJ | CHI33\_TRIHA | CHI42\_TRIHA | CHI4\_CRYJA | CHIA\_HUMAN | CHIC\_ARATH | CHIC\_SECCE | CHIL3\_MOUSE | CHIT\_PUNGR | CHLY\_HEVBR | FUCO\_HUMAN | G3JPF7\_CORMM | GBA1\_HUMAN | HEXC\_OSTFU | HYAL1\_HUMAN | IDUA\_HUMAN | LIPR2\_HUMAN | LIPR2\_RAT | LYG\_STRCA | MANBA\_MOUSE | NAGAB\_CHICK | NAGAB\_HUMAN | O81934\_CANEN | PGPSA\_DROME | PGRP1\_CAMDR | Q43576\_TOBAC | Q4AE59\_OSTFU | Q6WSR8\_PICAB | Q86RS6\_MANSE | Q8H0C9\_VIGUN | Q9FUH3\_VIGUS | SIA\_ASPFU | GO:0006950 | 0.161080074487896 | 519/3222 | 128/552 | 1.13208284129699e-06 | 0.00197888080658713 | T | T | T | T | response to stress | A0A087WNH2\_FICBE | A0A0A0Y4H8\_TRAFO | A0A1S4NYF8\_PANVG | A0A3L6SKP5\_PANMI | A6PZ97\_SALSA | ANAG\_HUMAN | ANG1\_BOVIN | ANG2\_MOUSE | ANG3\_MOUSE | ANG4\_MOUSE | ANGI\_MOUSE | AOC1\_HUMAN | B9TU22\_GADMO | CAT3\_NEUCR | CFAD\_MOUSE | CHI2\_HORVU | CHI2\_ORYSJ | CHI4\_CRYJA | CHIC\_ARATH | CHIC\_SECCE | CHIL3\_MOUSE | D1MPT2\_ROYRE | DABA\_PSEMU | DIR\_GLYEC | DOPO\_HUMAN | E0CX04\_MOMBA | E13B\_HORVU | ECP\_HUMAN | GBA1\_HUMAN | GPX3\_HUMAN | GPX5\_HUMAN | GPX6\_MOUSE | GRAC\_MOUSE | GRASS\_DROME | HYAL1\_HUMAN | K7N5L9\_RAPSA | KATG2\_MAGO7 | KLK8\_MOUSE | L8ICE9\_9CETA | LIG2\_PHACH | LIG4\_PHACH | LIG8\_PHACH | LIPR2\_RAT | LYG\_STRCA | LYSC1\_ANAPL | LYSC1\_CANLF | LYSC1\_HORSE | LYSC2\_BOVIN | LYSC2\_ONCMY | LYSC\_COTJA | LYSC\_EQUAS | LYSC\_NUMME | LYSC\_OPIHO | LYSC\_PELSI | LYS\_BOMMO | LYS\_RUDPH | MAN12\_PENCI | NCS\_THLFG | O04358\_IRIHO | O22443\_SOYBN | O81934\_CANEN | OXLA\_BOTAT | OXLA\_CALRH | OXLA\_GLOHA | PA21B\_BOVIN | PA2A\_BOTJR | PA2B3\_BOTAS | PA2GA\_HUMAN | PA2GE\_HUMAN | PA2GX\_HUMAN | PA2H1\_BOTBZ | PA2H1\_BOTJR | PA2H2\_BOTAS | PA2H2\_BOTMO | PA2H\_BOTPA | PCP\_HUMAN | PEM1\_PHACH | PER1A\_ARMRU | PER1\_ARAHY | PER1\_SORBI | PER53\_ARATH | PER59\_ARATH | PERL\_BOVIN | PERL\_BUBBU | PERL\_CAPHI | PER\_ARTRA | PER\_COPCI | PGH2\_HUMAN | PGPSA\_DROME | PGRP1\_CAMDR | POXA\_DICDI | PPA5\_HUMAN | PPA5\_RAT | PPAF1\_HOLDI | PRTN3\_HUMAN | Q2QEH4\_SAPOF | Q40069\_HORVU | Q5WRG2\_RAT | Q60FD2\_9APHY | Q70C53\_SOLTU | Q86RS6\_MANSE | Q8H0C9\_VIGUN | Q94BW3\_CINCA | Q9FUH3\_VIGUS | RENI\_RAT | RIP0\_DIACA | RIP1\_BRYDI | RIP1\_HORVU | RIP1\_MOMCH | RIP1\_PHYAM | RIP2\_PHYAM | RIP3\_MOMCH | RIPA\_PHYAM | RIPG\_SURMU | RIPL1\_PHYDI | RIPL2\_PHYDI | RIPT\_TRIKI | RNAS4\_HUMAN | RNAS4\_PIG | RNAS6\_HUMAN | RNS1B\_RAT | RNSL3\_DANRE | RNS\_BOVIN | RNT2\_HUMAN | TRFL\_BUBBU | TRFL\_HORSE | VPL1\_PLEER | VPL2\_PLEER | GO:0006040 | 0.0189323401613904 | 61/3222 | 26/552 | 2.12029544678521e-06 | 0.00370627644098054 | T | T | T | T | amino sugar metabolic process | A0A3B6UEQ2\_RHIMI | A5AB48\_ASPNC | A9LI60\_BIOOC | A9ZSX9\_9BRYO | CDA\_COLLN | CDA\_EMENI | CHI1\_COCPS | CHI2\_HORVU | CHI2\_ORYSJ | CHI33\_TRIHA | CHI42\_TRIHA | CHI4\_CRYJA | CHIA\_HUMAN | CHIC\_ARATH | CHIC\_SECCE | CHIL3\_MOUSE | CHIT\_PUNGR | CHLY\_HEVBR | G3JPF7\_CORMM | HEXC\_OSTFU | O81934\_CANEN | Q43576\_TOBAC | Q4AE59\_OSTFU | Q6WSR8\_PICAB | Q8H0C9\_VIGUN | Q9FUH3\_VIGUS | GO:0046271 | 0.00620732464307883 | 20/3222 | 13/552 | 2.30871311716276e-06 | 0.00403563052880051 | T | T | T | T | phenylpropanoid catabolic process | GCE2\_MYCTT | GCE\_CERUI | GCE\_HYPJQ | LAC1\_MELAO | LAC1\_TRAMX | LAC2\_TRAVE | LIG2\_PHACH | LIG4\_PHACH | LIG8\_PHACH | PEM1\_PHACH | Q60FD2\_9APHY | VPL1\_PLEER | VPL2\_PLEER | GO:0009605 | 0.0934202358783364 | 301/3222 | 82/552 | 2.55716464642996e-06 | 0.00446992380195957 | T | T | T | T | response to external stimulus | A6PZ97\_SALSA | AGAL\_ORYSJ | ANAG\_HUMAN | ANG1\_BOVIN | ANG2\_MOUSE | ANG3\_MOUSE | ANG4\_MOUSE | ANGI\_MOUSE | AOC1\_HUMAN | B9TU22\_GADMO | CARP1\_CANAL | CATD\_RAT | CERU\_RAT | CFAD\_MOUSE | CHI2\_ORYSJ | CHI4\_CRYJA | CHIC\_SECCE | DABA\_PSEMU | E13B\_HORVU | ECP\_HUMAN | GBA1\_HUMAN | GRAA\_HUMAN | GRAC\_MOUSE | GRASS\_DROME | HYAL1\_HUMAN | L8ICE9\_9CETA | LIPR2\_RAT | LYG\_STRCA | LYSC1\_ANAPL | LYSC1\_CANLF | LYSC1\_HORSE | LYSC2\_BOVIN | LYSC2\_ONCMY | LYSC\_COTJA | LYSC\_EQUAS | LYSC\_NUMME | LYSC\_OPIHO | LYSC\_PELSI | LYS\_BOMMO | LYS\_RUDPH | OXLA\_BOTAT | OXLA\_CALRH | OXLA\_GLOHA | PA21B\_BOVIN | PA2A\_BOTJR | PA2B3\_BOTAS | PA2GA\_HUMAN | PA2GX\_HUMAN | PA2H1\_BOTBZ | PA2H1\_BOTJR | PA2H2\_BOTAS | PA2H2\_BOTMO | PA2H\_BOTPA | PER53\_ARATH | PERL\_BOVIN | PERL\_BUBBU | PERL\_CAPHI | PGH2\_HUMAN | PGPSA\_DROME | PGRP1\_CAMDR | POXA\_DICDI | PPA5\_HUMAN | PPA5\_RAT | PPAF1\_HOLDI | PRTN3\_HUMAN | Q5WRG2\_RAT | Q86RS6\_MANSE | RENI\_RAT | RIP1\_HORVU | RIP1\_PHYAM | RIP2\_PHYAM | RIPA\_PHYAM | RNAS4\_HUMAN | RNAS4\_PIG | RNAS6\_HUMAN | RNS1B\_RAT | RNSL3\_DANRE | RNS\_BOVIN | RNT2\_HUMAN | TRFL\_BUBBU | TRFL\_HORSE | TTHY\_CHICK | GO:0032429 | 0.00217256362507759 | 7/3222 | 7/552 | 4.19700984574882e-06 | 0.00733637321036894 | T | T | T | T | regulation of phospholipase A2 activity | ANG1\_BOVIN | ANG2\_MOUSE | ANG3\_MOUSE | ANG4\_MOUSE | ANGI\_MOUSE | PPT1\_HUMAN | Q5WRG2\_RAT | GO:0006651 | 0.00217256362507759 | 7/3222 | 7/552 | 4.19700984574882e-06 | 0.00733637321036894 | T | T | T | T | diacylglycerol biosynthetic process | ANG1\_BOVIN | ANG2\_MOUSE | ANG3\_MOUSE | ANG4\_MOUSE | ANGI\_MOUSE | PAG15\_HUMAN | Q5WRG2\_RAT | GO:0005988 | 0.00217256362507759 | 7/3222 | 7/552 | 4.19700984574882e-06 | 0.00733637321036894 | T | T | T | T | lactose metabolic process | BGALA\_ASPNC | BGALA\_ASPOR | BGALA\_PENSQ | LALBA\_BOVIN | LALBA\_CAPHI | LALBA\_CAVPO | LALBA\_PAPCY | GO:0044238 | 0.709807572936065 | 2287/3222 | 434/552 | 5.58526320416611e-06 | 0.00976304008088236 | T | T | T | T | primary metabolic process | A0A059U759\_9PEZI | A0A068FT77\_9PEZI | A0A075B5H6\_TRIHA | A0A086SY89\_ACRC1 | A0A086T6R4\_ACRC1 | A0A088T0J9\_GEOCN | A0A0J5Q413\_ASPFM | A0A0M3KKZ6\_RHIMI | A0A0M3KKZ8\_RHIMI | A0A0R3QSA7\_9BILA | A0A0S2GKZ1\_9APHY | A0A173N065\_EISFE | A0A1L6CE30\_9EURO | A0A1L8D5Z7\_BOTAT | A0A1L9WG58\_ASPA1 | A0A1S9DRB1\_ASPOZ | A0A2H5BN17\_TALPI | A0A2N1LTK3\_TRIHA | A0A2Z4HIN9\_9EURO | A0A384E148\_NICBE | A0A3B6UEQ2\_RHIMI | A0A3G2C3I4\_9EURO | A0A3G4RHU4\_9PEZI | A0A3S5H5N2\_LEIDO | A0A482LWB1\_OSTFU | A0A5J6BJN2\_MALCI | A0A6F8Z6Y2\_BOMMO | A0A6M9BP13\_9EURO | A0A6P6YAT6\_DERPT | A0A856TAI5\_9BASI | A0NFU8\_ANOGA | A1E266\_9PEZI | A1HA\_LOXIN | A1HB2\_LOXIN | A2QZC8\_ASPNC | A2TM14\_HEVBR | A311\_LOXLA | A4GX63\_TOXGO | A5AB48\_ASPNC | A6YRT4\_9PEZI | A8NI40\_COPC7 | A8PUY1\_MALGO | A8PUY5\_MALGO | A9LI60\_BIOOC | A9ZSX9\_9BRYO | ABFB\_ASPKW | ADA2\_HUMAN | ADPG2\_ARATH | AGAL\_HUMAN | AGAL\_ORYSJ | AMY1A\_HUMAN | AMY1\_HORVU | AMY1\_ORYSJ | AMYA1\_ASPOR | AMYG\_SACFI | AMY\_ORYLA | ANAG\_HUMAN | ANG1\_BOVIN | ANG2\_MOUSE | ANG3\_MOUSE | ANG4\_MOUSE | ANGI\_MOUSE | AOAH\_MOUSE | ASAH1\_BALAS | ASM3A\_HUMAN | ASM3A\_MOUSE | AXE1\_ASPAW | AXE2\_TALPU | AXHA2\_EMENI | B2ZGS7\_9ASPA | B4F320\_LIMPO | B7X9Z0\_COPCI | B7X9Z2\_COPCI | BGALA\_ASPNC | BGALA\_ASPOR | BGALA\_PENSQ | BGL1\_ASPAC | BGLA\_ASPFU | BGLA\_ASPOR | BGLR\_HUMAN | C3VEV9\_PENCN | C7YSL3\_FUSV7 | CARP1\_CANAL | CARP2\_CANAX | CARP\_RHIPU | CATD\_RAT | CATH\_HUMAN | CATLL\_FASHE | CBHB\_ASPFU | CBHRE\_GEOS1 | CBPA1\_PIG | CBPD\_LOPSP | CBPN\_HUMAN | CDA\_COLLN | CDA\_EMENI | CEL2A\_PIG | CFAD\_MOUSE | CHI1\_COCPS | CHI2\_HORVU | CHI2\_ORYSJ | CHI33\_TRIHA | CHI42\_TRIHA | CHI4\_CRYJA | CHIA\_HUMAN | CHIC\_ARATH | CHIC\_SECCE | CHIL3\_MOUSE | CHIT\_PUNGR | CHLY\_HEVBR | CHYM\_CAMDR | COGS\_HYPLI | CONB\_CANEN | CUCM1\_CUCME | CUTI1\_ASPOR | CYP5\_CAEEL | CYSP\_BLOTA | D0QF43\_9HELO | D1M8S7\_HEVBR | D6XHE1\_TRYB2 | D9MWI4\_9ASPA | DDN1\_BOVIN | DNAS1\_HUMAN | DNSL3\_HUMAN | DPP2\_HUMAN | E0A7J0\_YARLL | E0XN39\_9EURO | E13B\_HORVU | E13C\_MUSAC | E3VTL0\_9ASPA | E5D0X5\_SCHOC | E9G5J5\_DAPPU | ECP\_HUMAN | EGFB2\_MOUSE | EGLB\_ASPNG | ENDO2\_ARATH | ENG1\_RHIMI | ENPP2\_HUMAN | ENPP2\_RAT | ERVB\_TABDI | EXG1\_CANAL | EXG1\_YEAST | F0ZJZ1\_DICPU | F1CYZ0\_TALFU | F2Z7L1\_9ANNE | FAEA\_ASPNG | FAEB1\_ASPOR | FAEB2\_ASPOR | FUCO\_HUMAN | G0RVK1\_HYPJQ | G2Q665\_MYCTT | G2QVH2\_THETT | G2X3Y1\_VERDV | G3I1H5\_CRIGR | G3JPF7\_CORMM | G3YAL0\_ASPNA | G3YFQ1\_ASPNA | G8GLP2\_LENED | G9NTY1\_HYPAI | GANA\_EMENI | GBA1\_HUMAN | GCE\_CERUI | GCE\_HYPJQ | GH7B\_LIMQU | GPX5\_HUMAN | GRAA\_HUMAN | GRAC\_MOUSE | GRAK\_HUMAN | GRASS\_DROME | GUB2\_HORVU | GUN2\_HYPJE | GUN6\_HUMIN | GUN7\_HYPJQ | GUNC\_FUSOX | GUN\_ASPAC | GUN\_CRYAT | GUN\_MYTED | GUX1\_HUMGT | GUX1\_HYPJE | GUX1\_TRIHA | GUX2\_HYPJE | H1AE14\_PHACH | HE12\_DANRE | HEXC\_OSTFU | HS3S1\_MOUSE | HYAL1\_HUMAN | I1SB18\_VIPAE | I2FI81\_EISFE | I3RY46\_TRIHA | IDH\_OSTTA | IDUA\_HUMAN | INU2\_ASPFI | INUE\_ASPAW | INV\_SCHOC | J7LCB0\_DEIAC | J9UN47\_GIBZA | K7CID1\_PANTR | K9L8F3\_MALCI | KLK10\_HUMAN | KLK1\_HUMAN | KLK2\_HORSE | KLK2\_HUMAN | KLK7\_HUMAN | KLK7\_MOUSE | KLK8\_MOUSE | L7SVX1\_RHIMI | LAC1\_MELAO | LALBA\_BOVIN | LALBA\_CAPHI | LALBA\_CAVPO | LALBA\_PAPCY | LAPA\_ASPOR | LGMN\_MOUSE | LICH\_HUMAN | LIP1\_DIURU | LIP2\_DIURU | LIP2\_GEOCN | LIP3\_DIURU | LIPA\_MOEAP | LIPB\_PSEA2 | LIPG\_CANLF | LIPG\_HUMAN | LIPP\_HORSE | LIPR1\_CANLF | LIPR1\_HUMAN | LIPR2\_HUMAN | LIPR2\_RAT | LIP\_THELA | M2RAI8\_CERS8 | M9TI89\_RHIPU | MAN12\_PENCI | MANA\_ASPNC | MANA\_CANEN | MANA\_CRYAT | MANA\_HYPJR | MANA\_MYTED | MANA\_PODAN | MANBA\_MOUSE | MCPT2\_RAT | MDLA\_PENCA | MDLA\_PENCY | MEL1\_YEASX | MMP1\_PIG | MNLOX\_MAGO7 | NAGAB\_CHICK | NAGAB\_HUMAN | NANL\_MACDE | NUP1\_PENCI | NUS1\_ASPOR | O00095\_HYPJE | O74705\_ASPNG | O77044\_9NEOP | O81100\_SOLLC | O81226\_CARPA | O81934\_CANEN | O97389\_HELAM | OFUT1\_CAEEL | P78583\_ASPOZ | P79074\_9AGAR | PA1\_VESBA | PA21B\_BOVIN | PA21B\_PIG | PA2A1\_BUNCE | PA2A1\_ECHCA | PA2A1\_NAJAT | PA2A1\_OPHHA | PA2A2\_NAJNA | PA2A2\_OPHHA | PA2A2\_TROCA | PA2A4\_NAJSG | PA2A5\_TRIST | PA2A7\_GLOHA | PA2A\_BOTJR | PA2A\_CROAT | PA2A\_DEIAC | PA2A\_GLOHA | PA2A\_NAJAT | PA2B1\_AGKPI | PA2B2\_BOTJR | PA2B2\_PROFL | PA2B3\_BOTAS | PA2B3\_BUNCE | PA2B5\_BUNCE | PA2B5\_NOTSC | PA2BA\_VIPAA | PA2BB\_GLOHA | PA2BB\_PSEAU | PA2BC\_VIPAA | PA2BD\_CRODU | PA2B\_BUNCE | PA2B\_NOTSC | PA2GA\_HUMAN | PA2GE\_HUMAN | PA2GX\_HUMAN | PA2H1\_AGKCL | PA2H1\_BOTBZ | PA2H1\_BOTJR | PA2H1\_BOTMO | PA2H1\_BOTPI | PA2H2\_BOTAS | PA2H2\_BOTMO | PA2H2\_BOTPI | PA2H2\_CERGO | PA2H3\_BOTPI | PA2HB\_AGKPI | PA2HB\_OXYSC | PA2HH\_TRIST | PA2HS\_ECHCA | PA2H\_BOTPA | PA2H\_DEIAC | PA2H\_PROMB | PA2N\_GLOHA | PA2\_APIME | PAG15\_HUMAN | PCP\_HUMAN | PDH1\_LEUMG | PELA\_ASPNG | PELB\_ASPNG | PEPA\_ASPPH | PGH2\_HUMAN | PGLR1\_ASPAC | PGLR1\_ASPNG | PGLR\_GIBFU | PGPSA\_DROME | PHAZ\_TALFU | PLA22\_ORYSJ | PLY1\_JUNAS | PME\_DAUCA | PME\_SITOR | PPAF1\_HOLDI | PPAP\_RAT | PPT1\_BOVIN | PPT1\_HUMAN | PPT2\_HUMAN | PRS57\_HUMAN | PRTN3\_HUMAN | PTGDS\_HUMAN | PTGDS\_MOUSE | Q02321\_PHACH | Q06AK3\_TOXGO | Q07524\_TROMA | Q0KFV0\_SOLLC | Q12715\_HYPJE | Q2U8V9\_ASPOR | Q43576\_TOBAC | Q4AE59\_OSTFU | Q4W6L6\_CYCRE | Q4WP32\_ASPFU | Q50KB2\_PHACH | Q55FE6\_DICDI | Q588B8\_CRYJA | Q5B038\_EMENI | Q5WRG2\_RAT | Q69G21\_TENMO | Q6NY42\_DANRE | Q6R7Z5\_9TRYP | Q6VAY1\_9PEZI | Q6WER3\_GIBZA | Q6WSR8\_PICAB | Q70C53\_SOLTU | Q70SY0\_HYPJE | Q7LHI2\_PHACH | Q7LIJ0\_PHACH | Q7LST4\_PENEN | Q7RWP2\_NEUCR | Q7X9A9\_CAMSI | Q7YXL2\_TENMO | Q8H0C9\_VIGUN | Q8J0K6\_MELAO | Q8J0K8\_MELAO | Q8NJY6\_9HYPO | Q8T0W7\_9NEOP | Q8TFL9\_TALEM | Q8TG26\_THEAU | Q8TGI8\_TALEM | Q92456\_HYPJE | Q92458\_HYPJE | Q93X60\_CICIN | Q95KP4\_HORSE | Q9FUH3\_VIGUS | Q9LYJ5\_ARATH | Q9P8F7\_YARLL | Q9STC1\_GRALE | Q9XEI3\_HORVV | QPCT1\_DROME | QPCT2\_DROME | QPCT\_IXOSC | QPCT\_MOUSE | RENI\_RAT | RGLA\_ASPAC | RHGA\_ASPAC | RNAS6\_HUMAN | RNLE\_SOLLC | RNT2\_HUMAN | S7Q6I2\_GLOTA | S7ZIW0\_PENO1 | SIA\_ASPFU | THCAS\_CANSA | TPP1\_HUMAN | TRFL\_BUBBU | TRFL\_HORSE | TRY1\_GADMO | TRY3\_SALSA | TRYB2\_HUMAN | TTHY\_CHICK | TTHY\_MOUSE | TTHY\_RAT | V5NTD\_NAJAT | VM11\_BOTMO | VM12\_CROAD | VM1A3\_DEIAC | VM1BI\_BOTMO | VM1T1\_PROMU | VM1T2\_PROFL | VSPP\_DEIAC | VSPSX\_GLOSA | W0T408\_KLUMD | W4KMP1\_HETIT | W6Q990\_PENRF | W8P1L2\_TALEM | W8VR85\_TALPI | X0M5X0\_FUSOX | XGHA\_ASPTU | XTH34\_POPPZ | XYLA\_ASPNC | XYN1\_HYPJR | XYN2\_HYPJR | XYN3\_ASPKW | XYN3\_HYPJQ | XYNA\_FUSO4 | XYNA\_PENSI | XYNA\_THEAU | XYNA\_THELA | XYNC\_ASPNC | XYND\_EMENI | GO:0042743 | 0.0229671011793917 | 74/3222 | 28/552 | 1.3900434147587e-05 | 0.0242979588899822 | T | T | T | T | hydrogen peroxide metabolic process | A0A087WNH2\_FICBE | A0A0A0Y4H8\_TRAFO | A0A1S4NYF8\_PANVG | A0A3L6SKP5\_PANMI | APO1\_CYCAE | CAT3\_NEUCR | D1MPT2\_ROYRE | GPX3\_HUMAN | K7N5L9\_RAPSA | KATG2\_MAGO7 | LIG4\_PHACH | LIG8\_PHACH | O22443\_SOYBN | PEM1\_PHACH | PER1A\_ARMRU | PER1\_ARAHY | PER1\_SORBI | PER53\_ARATH | PER59\_ARATH | PERL\_BOVIN | PERL\_BUBBU | PERL\_CAPHI | PER\_ARTRA | PER\_COPCI | POXA\_DICDI | Q40069\_HORVU | VPL1\_PLEER | VPL2\_PLEER | GO:0061844 | 0.0037243947858473 | 12/3222 | 9/552 | 1.61631611456595e-05 | 0.0282532056826128 | T | T | T | T | antimicrobial humoral immune response mediated by antimicrobial peptide | ANG1\_BOVIN | ANG2\_MOUSE | ANG3\_MOUSE | ANG4\_MOUSE | ANGI\_MOUSE | ECP\_HUMAN | PA21B\_BOVIN | Q5WRG2\_RAT | RNAS6\_HUMAN | GO:0006027 | 0.00620732464307883 | 20/3222 | 12/552 | 1.89784151756965e-05 | 0.0331742697271176 | T | T | T | T | glycosaminoglycan catabolic process | A0A0R4I979\_BRABE | A0A7S6G7I6\_9PEZI | A6PZ97\_SALSA | B9TU22\_GADMO | BGLR\_HUMAN | FUCO\_HUMAN | HYAL1\_HUMAN | IDUA\_HUMAN | LYG\_STRCA | PGPSA\_DROME | PGRP1\_CAMDR | Q86RS6\_MANSE | GO:0050832 | 0.00900062073246431 | 29/3222 | 15/552 | 1.9638383392169e-05 | 0.0343278941695114 | T | T | T | T | defense response to fungus | ANG2\_MOUSE | ANG3\_MOUSE | ANG4\_MOUSE | ANGI\_MOUSE | CHI2\_ORYSJ | CHI4\_CRYJA | CHIC\_SECCE | E13B\_HORVU | GRASS\_DROME | PA2H2\_BOTMO | PERL\_CAPHI | Q5WRG2\_RAT | RIP1\_HORVU | TRFL\_BUBBU | TRFL\_HORSE | GO:0017148 | 0.0142768466790813 | 46/3222 | 20/552 | 2.28927382502978e-05 | 0.0400165064615205 | T | T | T | T | negative regulation of translation | ANG1\_BOVIN | ANG3\_MOUSE | ANGI\_MOUSE | E0CX04\_MOMBA | GRAC\_MOUSE | O04358\_IRIHO | Q2QEH4\_SAPOF | Q94BW3\_CINCA | RIP0\_DIACA | RIP1\_BRYDI | RIP1\_HORVU | RIP1\_MOMCH | RIP1\_PHYAM | RIP2\_PHYAM | RIP3\_MOMCH | RIPA\_PHYAM | RIPG\_SURMU | RIPL1\_PHYDI | RIPL2\_PHYDI | RIPT\_TRIKI | GO:0032431 | 0.00186219739292365 | 6/3222 | 6/552 | 2.47208492013441e-05 | 0.0432120444039494 | T | T | T | T | activation of phospholipase A2 activity | ANG1\_BOVIN | ANG2\_MOUSE | ANG3\_MOUSE | ANG4\_MOUSE | ANGI\_MOUSE | Q5WRG2\_RAT | GO:0007202 | 0.00186219739292365 | 6/3222 | 6/552 | 2.47208492013441e-05 | 0.0432120444039494 | T | T | T | T | activation of phospholipase C activity | ANG1\_BOVIN | ANG2\_MOUSE | ANG3\_MOUSE | ANG4\_MOUSE | ANGI\_MOUSE | Q5WRG2\_RAT | GO:0032430 | 0.00186219739292365 | 6/3222 | 6/552 | 2.47208492013441e-05 | 0.0432120444039494 | T | T | T | T | positive regulation of phospholipase A2 activity | ANG1\_BOVIN | ANG2\_MOUSE | ANG3\_MOUSE | ANG4\_MOUSE | ANGI\_MOUSE | Q5WRG2\_RAT | GO:0010517 | 0.00248292985723153 | 8/3222 | 7/552 | 2.85958011418886e-05 | 0.0499854603960213 | T | T | T | T | regulation of phospholipase activity | ANG1\_BOVIN | ANG2\_MOUSE | ANG3\_MOUSE | ANG4\_MOUSE | ANGI\_MOUSE | PPT1\_HUMAN | Q5WRG2\_RAT | GO:0006955 | 0.0251396648044693 | 81/3222 | 29/552 | 3.3392726509503e-05 | 0.0583704859386112 | T | T | T | T | immune response | ANG1\_BOVIN | ANG2\_MOUSE | ANG3\_MOUSE | ANG4\_MOUSE | ANGI\_MOUSE | CATH\_HUMAN | CFAD\_MOUSE | ECP\_HUMAN | ENPP2\_HUMAN | ENPP2\_RAT | GRAA\_HUMAN | GRAC\_MOUSE | GRASS\_DROME | K7CID1\_PANTR | PA21B\_BOVIN | PERL\_BOVIN | PERL\_BUBBU | PERL\_CAPHI | PGPSA\_DROME | PGRP1\_CAMDR | POXA\_DICDI | PPAF1\_HOLDI | PRTN3\_HUMAN | Q5WRG2\_RAT | Q86RS6\_MANSE | RNAS6\_HUMAN | RNT2\_HUMAN | TRFL\_BUBBU | TRFL\_HORSE | GO:0034249 | 0.0145872129112353 | 47/3222 | 20/552 | 3.35840805137242e-05 | 0.0587049727379899 | T | T | T | T | negative regulation of amide metabolic process | ANG1\_BOVIN | ANG3\_MOUSE | ANGI\_MOUSE | E0CX04\_MOMBA | GRAC\_MOUSE | O04358\_IRIHO | Q2QEH4\_SAPOF | Q94BW3\_CINCA | RIP0\_DIACA | RIP1\_BRYDI | RIP1\_HORVU | RIP1\_MOMCH | RIP1\_PHYAM | RIP2\_PHYAM | RIP3\_MOMCH | RIPA\_PHYAM | RIPG\_SURMU | RIPL1\_PHYDI | RIPL2\_PHYDI | RIPT\_TRIKI | GO:0009808 | 0.00775915580384854 | 25/3222 | 13/552 | 6.67584155285838e-05 | 0.116693710343964 | T | T | T | T | lignin metabolic process | GCE2\_MYCTT | GCE\_CERUI | GCE\_HYPJQ | LAC1\_MELAO | LAC1\_TRAMX | LAC2\_TRAVE | LIG2\_PHACH | LIG4\_PHACH | LIG8\_PHACH | PEM1\_PHACH | Q60FD2\_9APHY | VPL1\_PLEER | VPL2\_PLEER | GO:0009620 | 0.010862818125388 | 35/3222 | 16/552 | 7.30757765146288e-05 | 0.127736457347571 | T | T | T | T | response to fungus | AGAL\_ORYSJ | ANG2\_MOUSE | ANG3\_MOUSE | ANG4\_MOUSE | ANGI\_MOUSE | CHI2\_ORYSJ | CHI4\_CRYJA | CHIC\_SECCE | E13B\_HORVU | GRASS\_DROME | PA2H2\_BOTMO | PERL\_CAPHI | Q5WRG2\_RAT | RIP1\_HORVU | TRFL\_BUBBU | TRFL\_HORSE | GO:0030203 | 0.00900062073246431 | 29/3222 | 14/552 | 9.98794570212809e-05 | 0.174589290873199 | T | T | T | T | glycosaminoglycan metabolic process | A0A0R4I979\_BRABE | A0A7S6G7I6\_9PEZI | A6PZ97\_SALSA | ANAG\_HUMAN | B9TU22\_GADMO | BGLR\_HUMAN | FUCO\_HUMAN | HS3S1\_MOUSE | HYAL1\_HUMAN | IDUA\_HUMAN | LYG\_STRCA | PGPSA\_DROME | PGRP1\_CAMDR | Q86RS6\_MANSE | GO:0046460 | 0.00279329608938547 | 9/3222 | 7/552 | 0.000109641692827468 | 0.191653679062415 | T | T | T | T | neutral lipid biosynthetic process | ANG1\_BOVIN | ANG2\_MOUSE | ANG3\_MOUSE | ANG4\_MOUSE | ANGI\_MOUSE | PAG15\_HUMAN | Q5WRG2\_RAT | GO:0046463 | 0.00279329608938547 | 9/3222 | 7/552 | 0.000109641692827468 | 0.191653679062415 | T | T | T | T | acylglycerol biosynthetic process | ANG1\_BOVIN | ANG2\_MOUSE | ANG3\_MOUSE | ANG4\_MOUSE | ANGI\_MOUSE | PAG15\_HUMAN | Q5WRG2\_RAT | GO:0046339 | 0.00279329608938547 | 9/3222 | 7/552 | 0.000109641692827468 | 0.191653679062415 | T | T | T | T | diacylglycerol metabolic process | ANG1\_BOVIN | ANG2\_MOUSE | ANG3\_MOUSE | ANG4\_MOUSE | ANGI\_MOUSE | PAG15\_HUMAN | Q5WRG2\_RAT | GO:0072593 | 0.0307262569832402 | 99/3222 | 32/552 | 0.000131672184425179 | 0.230162978375213 | T | T | T | T | reactive oxygen species metabolic process | A0A087WNH2\_FICBE | A0A0A0Y4H8\_TRAFO | A0A1S4NYF8\_PANVG | A0A3L6SKP5\_PANMI | ANAG\_HUMAN | APO1\_CYCAE | CAT3\_NEUCR | D1MPT2\_ROYRE | GPX3\_HUMAN | K7N5L9\_RAPSA | KATG2\_MAGO7 | LICH\_HUMAN | LIG4\_PHACH | LIG8\_PHACH | O22443\_SOYBN | PEM1\_PHACH | PER1A\_ARMRU | PER1\_ARAHY | PER1\_SORBI | PER53\_ARATH | PER59\_ARATH | PERL\_BOVIN | PERL\_BUBBU | PERL\_CAPHI | PER\_ARTRA | PER\_COPCI | POXA\_DICDI | PPA5\_HUMAN | PPA5\_RAT | Q40069\_HORVU | VPL1\_PLEER | VPL2\_PLEER | GO:0050829 | 0.00155183116076971 | 5/3222 | 5/552 | 0.000145387517149465 | 0.254137379977265 | T | T | T | T | defense response to Gram-negative bacterium | ECP\_HUMAN | LYSC2\_BOVIN | OXLA\_BOTAT | RNAS6\_HUMAN | RNSL3\_DANRE | GO:0001878 | 0.00155183116076971 | 5/3222 | 5/552 | 0.000145387517149465 | 0.254137379977265 | T | T | T | T | response to yeast | ANG2\_MOUSE | ANG3\_MOUSE | ANG4\_MOUSE | ANGI\_MOUSE | Q5WRG2\_RAT | GO:0010863 | 0.00217256362507759 | 7/3222 | 6/552 | 0.000147863885334872 | 0.258466071565357 | T | T | T | T | positive regulation of phospholipase C activity | ANG1\_BOVIN | ANG2\_MOUSE | ANG3\_MOUSE | ANG4\_MOUSE | ANGI\_MOUSE | Q5WRG2\_RAT | GO:1900274 | 0.00217256362507759 | 7/3222 | 6/552 | 0.000147863885334872 | 0.258466071565357 | T | T | T | T | regulation of phospholipase C activity | ANG1\_BOVIN | ANG2\_MOUSE | ANG3\_MOUSE | ANG4\_MOUSE | ANGI\_MOUSE | Q5WRG2\_RAT | GO:0009303 | 0.00217256362507759 | 7/3222 | 6/552 | 0.000147863885334872 | 0.258466071565357 | T | T | T | T | rRNA transcription | ANG1\_BOVIN | ANG2\_MOUSE | ANG3\_MOUSE | ANG4\_MOUSE | ANGI\_MOUSE | Q5WRG2\_RAT | GO:0010518 | 0.00217256362507759 | 7/3222 | 6/552 | 0.000147863885334872 | 0.258466071565357 | T | T | T | T | positive regulation of phospholipase activity | ANG1\_BOVIN | ANG2\_MOUSE | ANG3\_MOUSE | ANG4\_MOUSE | ANGI\_MOUSE | Q5WRG2\_RAT | GO:0098781 | 0.00217256362507759 | 7/3222 | 6/552 | 0.000147863885334872 | 0.258466071565357 | T | T | T | T | ncRNA transcription | ANG1\_BOVIN | ANG2\_MOUSE | ANG3\_MOUSE | ANG4\_MOUSE | ANGI\_MOUSE | Q5WRG2\_RAT | GO:0010629 | 0.0270018621973929 | 87/3222 | 29/552 | 0.00014816817584402 | 0.258997971375347 | T | T | T | T | negative regulation of gene expression | ANG1\_BOVIN | ANG3\_MOUSE | ANGI\_MOUSE | E0CX04\_MOMBA | G3I1H5\_CRIGR | GBA1\_HUMAN | GRAC\_MOUSE | LGMN\_MOUSE | O04358\_IRIHO | PA2GX\_HUMAN | PGRP1\_CAMDR | PPA5\_HUMAN | PPA5\_RAT | Q2QEH4\_SAPOF | Q94BW3\_CINCA | RIP0\_DIACA | RIP1\_BRYDI | RIP1\_HORVU | RIP1\_MOMCH | RIP1\_PHYAM | RIP2\_PHYAM | RIP3\_MOMCH | RIPA\_PHYAM | RIPG\_SURMU | RIPL1\_PHYDI | RIPL2\_PHYDI | RIPT\_TRIKI | TRFL\_BUBBU | TRFL\_HORSE | GO:0009253 | 0.0037243947858473 | 12/3222 | 8/552 | 0.000183833772523299 | 0.321341434370726 | T | T | T | T | peptidoglycan catabolic process | A0A0R4I979\_BRABE | A0A7S6G7I6\_9PEZI | A6PZ97\_SALSA | B9TU22\_GADMO | LYG\_STRCA | PGPSA\_DROME | PGRP1\_CAMDR | Q86RS6\_MANSE | GO:0060191 | 0.00310366232153942 | 10/3222 | 7/552 | 0.000311537527577398 | 0.544567598205292 | T | T | T | T | regulation of lipase activity | ANG1\_BOVIN | ANG2\_MOUSE | ANG3\_MOUSE | ANG4\_MOUSE | ANGI\_MOUSE | PPT1\_HUMAN | Q5WRG2\_RAT | GO:0019377 | 0.00310366232153942 | 10/3222 | 7/552 | 0.000311537527577398 | 0.544567598205292 | T | T | T | T | glycolipid catabolic process | AGAL\_HUMAN | FUCO\_HUMAN | GBA1\_HUMAN | LIPR2\_HUMAN | LIPR2\_RAT | NAGAB\_HUMAN | SIA\_ASPFU | GO:0000270 | 0.00403476101800124 | 13/3222 | 8/552 | 0.000406642420464679 | 0.710810950972258 | T | T | T | T | peptidoglycan metabolic process | A0A0R4I979\_BRABE | A0A7S6G7I6\_9PEZI | A6PZ97\_SALSA | B9TU22\_GADMO | LYG\_STRCA | PGPSA\_DROME | PGRP1\_CAMDR | Q86RS6\_MANSE | GO:0046470 | 0.00589695841092489 | 19/3222 | 10/552 | 0.000423658253227094 | 0.74055462664096 | T | T | T | T | phosphatidylcholine metabolic process | ENPP2\_HUMAN | ENPP2\_RAT | LIPR2\_HUMAN | LIPR2\_RAT | PA21B\_BOVIN | PA21B\_PIG | PA2GA\_HUMAN | PA2GE\_HUMAN | PA2GX\_HUMAN | PAG15\_HUMAN | GO:0046355 | 0.00248292985723153 | 8/3222 | 6/552 | 0.000505668137913914 | 0.883907905073522 | T | T | T | T | mannan catabolic process | L7SVX1\_RHIMI | MANA\_ASPNC | MANA\_CRYAT | MANA\_HYPJR | MANA\_MYTED | MANA\_PODAN | GO:0034638 | 0.00248292985723153 | 8/3222 | 6/552 | 0.000505668137913914 | 0.883907905073522 | T | T | T | T | phosphatidylcholine catabolic process | ENPP2\_HUMAN | ENPP2\_RAT | LIPR2\_HUMAN | LIPR2\_RAT | PA2GX\_HUMAN | PAG15\_HUMAN | GO:0060193 | 0.00248292985723153 | 8/3222 | 6/552 | 0.000505668137913914 | 0.883907905073522 | T | T | T | T | positive regulation of lipase activity | ANG1\_BOVIN | ANG2\_MOUSE | ANG3\_MOUSE | ANG4\_MOUSE | ANGI\_MOUSE | Q5WRG2\_RAT | GO:0010412 | 0.00248292985723153 | 8/3222 | 6/552 | 0.000505668137913914 | 0.883907905073522 | T | T | T | T | mannan metabolic process | L7SVX1\_RHIMI | MANA\_ASPNC | MANA\_CRYAT | MANA\_HYPJR | MANA\_MYTED | MANA\_PODAN | GO:0046373 | 0.00248292985723153 | 8/3222 | 6/552 | 0.000505668137913914 | 0.883907905073522 | T | T | T | T | L-arabinose metabolic process | A0A059U759\_9PEZI | A0A2H5BN17\_TALPI | A8NI40\_COPC7 | ABFB\_ASPKW | AXHA2\_EMENI | G2QVH2\_THETT | GO:0051345 | 0.00713842333954066 | 23/3222 | 11/552 | 0.000628539555767834 | 1 | T | T | T | F | positive regulation of hydrolase activity | ANG1\_BOVIN | ANG2\_MOUSE | ANG3\_MOUSE | ANG4\_MOUSE | ANGI\_MOUSE | CATD\_RAT | CATH\_HUMAN | G3I1H5\_CRIGR | LGMN\_MOUSE | PRTN3\_HUMAN | Q5WRG2\_RAT | GO:0010608 | 0.021415270018622 | 69/3222 | 23/552 | 0.000714141340756159 | 1 | T | T | T | F | post-transcriptional regulation of gene expression | ANG1\_BOVIN | ANG3\_MOUSE | ANGI\_MOUSE | D6XHE1\_TRYB2 | E0CX04\_MOMBA | GRAC\_MOUSE | O04358\_IRIHO | Q2QEH4\_SAPOF | Q5WRG2\_RAT | Q6R7Z5\_9TRYP | Q94BW3\_CINCA | RIP0\_DIACA | RIP1\_BRYDI | RIP1\_HORVU | RIP1\_MOMCH | RIP1\_PHYAM | RIP2\_PHYAM | RIP3\_MOMCH | RIPA\_PHYAM | RIPG\_SURMU | RIPL1\_PHYDI | RIPL2\_PHYDI | RIPT\_TRIKI | GO:0042545 | 0.00186219739292365 | 6/3222 | 5/552 | 0.000748720856889623 | 1 | T | T | T | F | cell wall modification | ADPG2\_ARATH | G3YAL0\_ASPNA | PME\_DAUCA | PME\_SITOR | Q9LYJ5\_ARATH | GO:0018199 | 0.00186219739292365 | 6/3222 | 5/552 | 0.000748720856889623 | 1 | T | T | T | F | peptidyl-glutamine modification | O81226\_CARPA | QPCT1\_DROME | QPCT2\_DROME | QPCT\_IXOSC | QPCT\_MOUSE | GO:0017186 | 0.00186219739292365 | 6/3222 | 5/552 | 0.000748720856889623 | 1 | T | T | T | F | peptidyl-pyroglutamic acid biosynthetic process, using glutaminyl-peptide cyclotransferase | O81226\_CARPA | QPCT1\_DROME | QPCT2\_DROME | QPCT\_IXOSC | QPCT\_MOUSE | GO:0002385 | 0.00124146492861577 | 4/3222 | 4/552 | 0.000853753704719909 | 1 | T | T | T | F | mucosal immune response | ECP\_HUMAN | PA21B\_BOVIN | TRFL\_BUBBU | TRFL\_HORSE | GO:0002251 | 0.00124146492861577 | 4/3222 | 4/552 | 0.000853753704719909 | 1 | T | T | T | F | organ or tissue specific immune response | ECP\_HUMAN | PA21B\_BOVIN | TRFL\_BUBBU | TRFL\_HORSE | GO:0002784 | 0.00124146492861577 | 4/3222 | 4/552 | 0.000853753704719909 | 1 | T | T | T | F | regulation of antimicrobial peptide production | GRASS\_DROME | KLK7\_HUMAN | KLK7\_MOUSE | PGPSA\_DROME | GO:1900426 | 0.00124146492861577 | 4/3222 | 4/552 | 0.000853753704719909 | 1 | T | T | T | F | positive regulation of defense response to bacterium | GRASS\_DROME | KLK7\_HUMAN | KLK7\_MOUSE | PGPSA\_DROME | GO:0005989 | 0.00124146492861577 | 4/3222 | 4/552 | 0.000853753704719909 | 1 | T | T | T | F | lactose biosynthetic process | LALBA\_BOVIN | LALBA\_CAPHI | LALBA\_CAVPO | LALBA\_PAPCY | GO:0002803 | 0.00124146492861577 | 4/3222 | 4/552 | 0.000853753704719909 | 1 | T | T | T | F | positive regulation of antibacterial peptide production | GRASS\_DROME | KLK7\_HUMAN | KLK7\_MOUSE | PGPSA\_DROME | GO:0002225 | 0.00124146492861577 | 4/3222 | 4/552 | 0.000853753704719909 | 1 | T | T | T | F | positive regulation of antimicrobial peptide production | GRASS\_DROME | KLK7\_HUMAN | KLK7\_MOUSE | PGPSA\_DROME | GO:0002786 | 0.00124146492861577 | 4/3222 | 4/552 | 0.000853753704719909 | 1 | T | T | T | F | regulation of antibacterial peptide production | GRASS\_DROME | KLK7\_HUMAN | KLK7\_MOUSE | PGPSA\_DROME | GO:0008306 | 0.00124146492861577 | 4/3222 | 4/552 | 0.000853753704719909 | 1 | T | T | T | F | associative learning | DOPO\_HUMAN | G3I1H5\_CRIGR | LGMN\_MOUSE | PPT1\_HUMAN | GO:0002227 | 0.00124146492861577 | 4/3222 | 4/552 | 0.000853753704719909 | 1 | T | T | T | F | innate immune response in mucosa | ECP\_HUMAN | PA21B\_BOVIN | TRFL\_BUBBU | TRFL\_HORSE | GO:0006643 | 0.00962135319677219 | 31/3222 | 13/552 | 0.000970057612017984 | 1 | T | T | T | F | membrane lipid metabolic process | AGAL\_HUMAN | ANAG\_HUMAN | ASAH1\_BALAS | ENPP2\_HUMAN | ENPP2\_RAT | FUCO\_HUMAN | GBA1\_HUMAN | LIPR2\_HUMAN | LIPR2\_RAT | NAGAB\_HUMAN | PAG15\_HUMAN | PPT1\_HUMAN | SIA\_ASPFU | GO:0045087 | 0.0167597765363128 | 54/3222 | 19/552 | 0.000981154200975846 | 1 | T | T | T | F | innate immune response | ANG1\_BOVIN | ANG2\_MOUSE | ANG3\_MOUSE | ANG4\_MOUSE | ANGI\_MOUSE | CFAD\_MOUSE | ECP\_HUMAN | GRAC\_MOUSE | GRASS\_DROME | PA21B\_BOVIN | PGPSA\_DROME | PGRP1\_CAMDR | PPAF1\_HOLDI | Q5WRG2\_RAT | Q86RS6\_MANSE | RNAS6\_HUMAN | RNT2\_HUMAN | TRFL\_BUBBU | TRFL\_HORSE | GO:0016139 | 0.00279329608938547 | 9/3222 | 6/552 | 0.00129772102808665 | 1 | T | T | F | F | glycoside catabolic process | AGAL\_HUMAN | BGLR\_HUMAN | FUCO\_HUMAN | GBA1\_HUMAN | NAGAB\_CHICK | NAGAB\_HUMAN | GO:0019566 | 0.00279329608938547 | 9/3222 | 6/552 | 0.00129772102808665 | 1 | T | T | F | F | arabinose metabolic process | A0A059U759\_9PEZI | A0A2H5BN17\_TALPI | A8NI40\_COPC7 | ABFB\_ASPKW | AXHA2\_EMENI | G2QVH2\_THETT | GO:0001938 | 0.00279329608938547 | 9/3222 | 6/552 | 0.00129772102808665 | 1 | T | T | F | F | positive regulation of endothelial cell proliferation | ANG1\_BOVIN | ANG2\_MOUSE | ANG3\_MOUSE | ANG4\_MOUSE | ANGI\_MOUSE | Q5WRG2\_RAT | GO:0006308 | 0.00279329608938547 | 9/3222 | 6/552 | 0.00129772102808665 | 1 | T | T | F | F | DNA catabolic process | DNAS1\_HUMAN | DNSL3\_HUMAN | ENDO2\_ARATH | NUP1\_PENCI | NUS1\_ASPOR | Q0KFV0\_SOLLC | GO:1903509 | 0.00558659217877095 | 18/3222 | 9/552 | 0.00134048895056627 | 1 | T | T | F | F | liposaccharide metabolic process | AGAL\_HUMAN | ANAG\_HUMAN | AOAH\_MOUSE | FUCO\_HUMAN | GBA1\_HUMAN | LIPR2\_HUMAN | LIPR2\_RAT | NAGAB\_HUMAN | SIA\_ASPFU | GO:0046466 | 0.00682805710738672 | 22/3222 | 10/552 | 0.0018134394162352 | 1 | T | T | F | F | membrane lipid catabolic process | AGAL\_HUMAN | ENPP2\_HUMAN | ENPP2\_RAT | FUCO\_HUMAN | GBA1\_HUMAN | LIPR2\_HUMAN | LIPR2\_RAT | NAGAB\_HUMAN | PPT1\_HUMAN | SIA\_ASPFU | GO:0002252 | 0.00682805710738672 | 22/3222 | 10/552 | 0.0018134394162352 | 1 | T | T | F | F | immune effector process | CATH\_HUMAN | CFAD\_MOUSE | DNAS1\_HUMAN | DNSL3\_HUMAN | DOPO\_HUMAN | GRAC\_MOUSE | LICH\_HUMAN | PA21B\_PIG | PTGDS\_HUMAN | PTGDS\_MOUSE | GO:0006417 | 0.0189323401613904 | 61/3222 | 20/552 | 0.00197310417421698 | 1 | T | T | F | F | regulation of translation | ANG1\_BOVIN | ANG3\_MOUSE | ANGI\_MOUSE | E0CX04\_MOMBA | GRAC\_MOUSE | O04358\_IRIHO | Q2QEH4\_SAPOF | Q94BW3\_CINCA | RIP0\_DIACA | RIP1\_BRYDI | RIP1\_HORVU | RIP1\_MOMCH | RIP1\_PHYAM | RIP2\_PHYAM | RIP3\_MOMCH | RIPA\_PHYAM | RIPG\_SURMU | RIPL1\_PHYDI | RIPL2\_PHYDI | RIPT\_TRIKI | GO:0009311 | 0.0127250155183116 | 41/3222 | 15/552 | 0.00212511829833071 | 1 | T | T | F | F | oligosaccharide metabolic process | AGAL\_HUMAN | AMY1A\_HUMAN | AMY1\_ORYSJ | BGALA\_ASPNC | BGALA\_ASPOR | BGALA\_PENSQ | IDUA\_HUMAN | LALBA\_BOVIN | LALBA\_CAPHI | LALBA\_CAVPO | LALBA\_PAPCY | MANBA\_MOUSE | NAGAB\_CHICK | NAGAB\_HUMAN | SIA\_ASPFU | GO:0007612 | 0.00217256362507759 | 7/3222 | 5/552 | 0.00225086328577618 | 1 | T | T | F | F | learning | DOPO\_HUMAN | G3I1H5\_CRIGR | LGMN\_MOUSE | PGH2\_HUMAN | PPT1\_HUMAN | GO:0080171 | 0.00217256362507759 | 7/3222 | 5/552 | 0.00225086328577618 | 1 | T | T | F | F | lytic vacuole organization | ANAG\_HUMAN | GBA1\_HUMAN | LICH\_HUMAN | PPAP\_RAT | TPP1\_HUMAN | GO:0031638 | 0.00217256362507759 | 7/3222 | 5/552 | 0.00225086328577618 | 1 | T | T | F | F | zymogen activation | CATH\_HUMAN | EGFB2\_MOUSE | G3I1H5\_CRIGR | KLK1\_HUMAN | LGMN\_MOUSE | GO:0007040 | 0.00217256362507759 | 7/3222 | 5/552 | 0.00225086328577618 | 1 | T | T | F | F | lysosome organization | ANAG\_HUMAN | GBA1\_HUMAN | LICH\_HUMAN | PPAP\_RAT | TPP1\_HUMAN | GO:0046475 | 0.00310366232153942 | 10/3222 | 6/552 | 0.0027769962788505 | 1 | T | T | F | F | glycerophospholipid catabolic process | ENPP2\_HUMAN | ENPP2\_RAT | LIPR2\_HUMAN | LIPR2\_RAT | PA2GX\_HUMAN | PAG15\_HUMAN | GO:0006629 | 0.17163252638113 | 553/3222 | 118/552 | 0.00282930135550646 | 1 | T | T | F | F | lipid metabolic process | A0A1L8D5Z7\_BOTAT | A1HA\_LOXIN | A1HB2\_LOXIN | A311\_LOXLA | A8PUY1\_MALGO | A8PUY5\_MALGO | AGAL\_HUMAN | ANAG\_HUMAN | ANG1\_BOVIN | ANG2\_MOUSE | ANG3\_MOUSE | ANG4\_MOUSE | ANGI\_MOUSE | AOAH\_MOUSE | ASAH1\_BALAS | CUTI1\_ASPOR | E0A7J0\_YARLL | ENPP2\_HUMAN | ENPP2\_RAT | FAEA\_ASPNG | FUCO\_HUMAN | GBA1\_HUMAN | GPX5\_HUMAN | I1SB18\_VIPAE | LICH\_HUMAN | LIP1\_DIURU | LIP2\_DIURU | LIP2\_GEOCN | LIP3\_DIURU | LIPA\_MOEAP | LIPB\_PSEA2 | LIPG\_CANLF | LIPG\_HUMAN | LIPP\_HORSE | LIPR1\_CANLF | LIPR1\_HUMAN | LIPR2\_HUMAN | LIPR2\_RAT | LIP\_THELA | MDLA\_PENCA | MDLA\_PENCY | MNLOX\_MAGO7 | NAGAB\_CHICK | NAGAB\_HUMAN | P78583\_ASPOZ | PA1\_VESBA | PA21B\_BOVIN | PA21B\_PIG | PA2A1\_BUNCE | PA2A1\_ECHCA | PA2A1\_NAJAT | PA2A1\_OPHHA | PA2A2\_NAJNA | PA2A2\_OPHHA | PA2A2\_TROCA | PA2A4\_NAJSG | PA2A5\_TRIST | PA2A7\_GLOHA | PA2A\_BOTJR | PA2A\_CROAT | PA2A\_DEIAC | PA2A\_GLOHA | PA2A\_NAJAT | PA2B1\_AGKPI | PA2B2\_BOTJR | PA2B2\_PROFL | PA2B3\_BOTAS | PA2B3\_BUNCE | PA2B5\_BUNCE | PA2B5\_NOTSC | PA2BA\_VIPAA | PA2BB\_GLOHA | PA2BB\_PSEAU | PA2BC\_VIPAA | PA2BD\_CRODU | PA2B\_BUNCE | PA2B\_NOTSC | PA2GA\_HUMAN | PA2GE\_HUMAN | PA2GX\_HUMAN | PA2H1\_AGKCL | PA2H1\_BOTBZ | PA2H1\_BOTJR | PA2H1\_BOTMO | PA2H1\_BOTPI | PA2H2\_BOTAS | PA2H2\_BOTMO | PA2H2\_BOTPI | PA2H2\_CERGO | PA2H3\_BOTPI | PA2HB\_AGKPI | PA2HB\_OXYSC | PA2HH\_TRIST | PA2HS\_ECHCA | PA2H\_BOTPA | PA2H\_DEIAC | PA2H\_PROMB | PA2N\_GLOHA | PA2\_APIME | PAG15\_HUMAN | PGH2\_HUMAN | PLA22\_ORYSJ | PPAP\_RAT | PPT1\_BOVIN | PPT1\_HUMAN | PPT2\_HUMAN | PTGDS\_HUMAN | PTGDS\_MOUSE | Q5WRG2\_RAT | Q6WER3\_GIBZA | Q7LST4\_PENEN | Q95KP4\_HORSE | Q9P8F7\_YARLL | SIA\_ASPFU | THCAS\_CANSA | TPP1\_HUMAN | TTHY\_CHICK | W6Q990\_PENRF | GO:0006638 | 0.00837988826815642 | 27/3222 | 11/552 | 0.00313562704277083 | 1 | T | T | F | F | neutral lipid metabolic process | ANG1\_BOVIN | ANG2\_MOUSE | ANG3\_MOUSE | ANG4\_MOUSE | ANGI\_MOUSE | LICH\_HUMAN | LIPG\_HUMAN | LIPR2\_HUMAN | LIPR2\_RAT | PAG15\_HUMAN | Q5WRG2\_RAT | GO:0034248 | 0.0223463687150838 | 72/3222 | 22/552 | 0.0032848074921632 | 1 | T | T | F | F | regulation of amide metabolic process | ANG1\_BOVIN | ANG3\_MOUSE | ANGI\_MOUSE | E0CX04\_MOMBA | GRAC\_MOUSE | GRASS\_DROME | O04358\_IRIHO | PGPSA\_DROME | Q2QEH4\_SAPOF | Q94BW3\_CINCA | RIP0\_DIACA | RIP1\_BRYDI | RIP1\_HORVU | RIP1\_MOMCH | RIP1\_PHYAM | RIP2\_PHYAM | RIP3\_MOMCH | RIPA\_PHYAM | RIPG\_SURMU | RIPL1\_PHYDI | RIPL2\_PHYDI | RIPT\_TRIKI | GO:0001525 | 0.00620732464307883 | 20/3222 | 9/552 | 0.00334927013475474 | 1 | T | T | F | F | angiogenesis | ANG1\_BOVIN | ANG2\_MOUSE | ANG3\_MOUSE | ANG4\_MOUSE | ANGI\_MOUSE | PCP\_HUMAN | PGH2\_HUMAN | Q5WRG2\_RAT | RNSL3\_DANRE | GO:0001934 | 0.00620732464307883 | 20/3222 | 9/552 | 0.00334927013475474 | 1 | T | T | F | F | positive regulation of protein phosphorylation | ANG2\_MOUSE | ANG3\_MOUSE | ANG4\_MOUSE | ANGI\_MOUSE | ENPP2\_HUMAN | ENPP2\_RAT | PA21B\_PIG | PGH2\_HUMAN | Q5WRG2\_RAT | GO:0051604 | 0.010862818125388 | 35/3222 | 13/552 | 0.00356959079346528 | 1 | T | T | F | F | protein maturation | ANAG\_HUMAN | CARP1\_CANAL | CATH\_HUMAN | CBPN\_HUMAN | EGFB2\_MOUSE | G3I1H5\_CRIGR | GRAA\_HUMAN | GRASS\_DROME | KLK1\_HUMAN | LGMN\_MOUSE | PCP\_HUMAN | PGPSA\_DROME | RENI\_RAT | GO:0051247 | 0.0133457479826195 | 43/3222 | 15/552 | 0.00363217773455106 | 1 | T | T | F | F | positive regulation of protein metabolic process | ANG2\_MOUSE | ANG3\_MOUSE | ANG4\_MOUSE | ANGI\_MOUSE | CATD\_RAT | CATH\_HUMAN | ENPP2\_HUMAN | ENPP2\_RAT | G3I1H5\_CRIGR | GBA1\_HUMAN | LGMN\_MOUSE | PA21B\_PIG | PA2GX\_HUMAN | PGH2\_HUMAN | Q5WRG2\_RAT | GO:0060349 | 0.00155183116076971 | 5/3222 | 4/552 | 0.00368721845500558 | 1 | T | T | F | F | bone morphogenesis | PPA5\_HUMAN | PPA5\_RAT | TRFL\_BUBBU | TRFL\_HORSE | GO:0048660 | 0.00527622594661701 | 17/3222 | 8/552 | 0.00404042052618264 | 1 | T | T | F | F | regulation of smooth muscle cell proliferation | ANG1\_BOVIN | ANG2\_MOUSE | ANG3\_MOUSE | ANG4\_MOUSE | ANGI\_MOUSE | DOPO\_HUMAN | PGH2\_HUMAN | Q5WRG2\_RAT | GO:0006664 | 0.00527622594661701 | 17/3222 | 8/552 | 0.00404042052618264 | 1 | T | T | F | F | glycolipid metabolic process | AGAL\_HUMAN | ANAG\_HUMAN | FUCO\_HUMAN | GBA1\_HUMAN | LIPR2\_HUMAN | LIPR2\_RAT | NAGAB\_HUMAN | SIA\_ASPFU | GO:0010634 | 0.00434512725015518 | 14/3222 | 7/552 | 0.00472695470495533 | 1 | T | T | F | F | positive regulation of epithelial cell migration | CATH\_HUMAN | ENPP2\_HUMAN | ENPP2\_RAT | G3I1H5\_CRIGR | HYAL1\_HUMAN | LGMN\_MOUSE | PGH2\_HUMAN | GO:0001818 | 0.00434512725015518 | 14/3222 | 7/552 | 0.00472695470495533 | 1 | T | T | F | F | negative regulation of cytokine production | GBA1\_HUMAN | PA2GX\_HUMAN | PGRP1\_CAMDR | PPA5\_HUMAN | PPA5\_RAT | TRFL\_BUBBU | TRFL\_HORSE | GO:0030163 | 0.0124146492861577 | 40/3222 | 14/552 | 0.00473696065660328 | 1 | T | T | F | F | protein catabolic process | ANAG\_HUMAN | BGLR\_HUMAN | CARP1\_CANAL | CATD\_RAT | CBPN\_HUMAN | CEL2A\_PIG | DPP2\_HUMAN | GBA1\_HUMAN | IDUA\_HUMAN | MAN12\_PENCI | MANBA\_MOUSE | PPT1\_BOVIN | PPT1\_HUMAN | TPP1\_HUMAN | GO:0050906 | 0.000931098696461825 | 3/3222 | 3/552 | 0.00500588920854884 | 1 | T | T | F | F | detection of stimulus involved in sensory perception | CAH6\_HUMAN | L8ICE9\_9CETA | PERL\_CAPHI | GO:0072376 | 0.000931098696461825 | 3/3222 | 3/552 | 0.00500588920854884 | 1 | T | T | F | F | protein activation cascade | GRASS\_DROME | PCP\_HUMAN | PGPSA\_DROME | GO:0001580 | 0.000931098696461825 | 3/3222 | 3/552 | 0.00500588920854884 | 1 | T | T | F | F | detection of chemical stimulus involved in sensory perception of bitter taste | CAH6\_HUMAN | L8ICE9\_9CETA | PERL\_CAPHI | GO:0005990 | 0.000931098696461825 | 3/3222 | 3/552 | 0.00500588920854884 | 1 | T | T | F | F | lactose catabolic process | BGALA\_ASPNC | BGALA\_ASPOR | BGALA\_PENSQ | GO:0050907 | 0.000931098696461825 | 3/3222 | 3/552 | 0.00500588920854884 | 1 | T | T | F | F | detection of chemical stimulus involved in sensory perception | CAH6\_HUMAN | L8ICE9\_9CETA | PERL\_CAPHI | GO:0050912 | 0.000931098696461825 | 3/3222 | 3/552 | 0.00500588920854884 | 1 | T | T | F | F | detection of chemical stimulus involved in sensory perception of taste | CAH6\_HUMAN | L8ICE9\_9CETA | PERL\_CAPHI | GO:0046471 | 0.00248292985723153 | 8/3222 | 5/552 | 0.00515952186554746 | 1 | T | T | F | F | phosphatidylglycerol metabolic process | PA21B\_BOVIN | PA21B\_PIG | PA2GE\_HUMAN | PA2GX\_HUMAN | PAG15\_HUMAN | GO:0032148 | 0.00248292985723153 | 8/3222 | 5/552 | 0.00515952186554746 | 1 | T | T | F | F | activation of protein kinase B activity | ANG2\_MOUSE | ANG3\_MOUSE | ANG4\_MOUSE | ANGI\_MOUSE | Q5WRG2\_RAT | GO:0032147 | 0.00248292985723153 | 8/3222 | 5/552 | 0.00515952186554746 | 1 | T | T | F | F | activation of protein kinase activity | ANG2\_MOUSE | ANG3\_MOUSE | ANG4\_MOUSE | ANGI\_MOUSE | Q5WRG2\_RAT | GO:0006351 | 0.00341402855369336 | 11/3222 | 6/552 | 0.00523263003883918 | 1 | T | T | F | F | DNA-templated transcription | ANG1\_BOVIN | ANG2\_MOUSE | ANG3\_MOUSE | ANG4\_MOUSE | ANGI\_MOUSE | Q5WRG2\_RAT | GO:0016072 | 0.00341402855369336 | 11/3222 | 6/552 | 0.00523263003883918 | 1 | T | T | F | F | rRNA metabolic process | ANG1\_BOVIN | ANG2\_MOUSE | ANG3\_MOUSE | ANG4\_MOUSE | ANGI\_MOUSE | Q5WRG2\_RAT | GO:0048662 | 0.00341402855369336 | 11/3222 | 6/552 | 0.00523263003883918 | 1 | T | T | F | F | negative regulation of smooth muscle cell proliferation | ANG1\_BOVIN | ANG2\_MOUSE | ANG3\_MOUSE | ANG4\_MOUSE | ANGI\_MOUSE | Q5WRG2\_RAT | GO:0051336 | 0.0127250155183116 | 41/3222 | 14/552 | 0.00609124928681862 | 1 | T | T | F | F | regulation of hydrolase activity | ANG1\_BOVIN | ANG2\_MOUSE | ANG3\_MOUSE | ANG4\_MOUSE | ANGI\_MOUSE | CATD\_RAT | CATH\_HUMAN | G3I1H5\_CRIGR | GRAA\_HUMAN | LGMN\_MOUSE | PGH2\_HUMAN | PPT1\_HUMAN | PRTN3\_HUMAN | Q5WRG2\_RAT | GO:0048871 | 0.0114835505896958 | 37/3222 | 13/552 | 0.00618434481234162 | 1 | T | T | F | F | multicellular organismal-level homeostasis | ANAG\_HUMAN | CATH\_HUMAN | DOPO\_HUMAN | GBA1\_HUMAN | LICH\_HUMAN | PA2GA\_HUMAN | PA2GX\_HUMAN | PCP\_HUMAN | PGH2\_HUMAN | PPA5\_HUMAN | PPA5\_PIG | PPA5\_RAT | TPP1\_HUMAN | GO:0010632 | 0.00558659217877095 | 18/3222 | 8/552 | 0.00620036578668115 | 1 | T | T | F | F | regulation of epithelial cell migration | CATH\_HUMAN | ENPP2\_HUMAN | ENPP2\_RAT | G3I1H5\_CRIGR | HYAL1\_HUMAN | LGMN\_MOUSE | PCP\_HUMAN | PGH2\_HUMAN | GO:0016485 | 0.00682805710738672 | 22/3222 | 9/552 | 0.00718485116506019 | 1 | T | T | F | F | protein processing | ANAG\_HUMAN | CARP1\_CANAL | CATH\_HUMAN | CBPN\_HUMAN | EGFB2\_MOUSE | G3I1H5\_CRIGR | KLK1\_HUMAN | LGMN\_MOUSE | RENI\_RAT | GO:0001936 | 0.00465549348230913 | 15/3222 | 7/552 | 0.00757380228157741 | 1 | T | T | F | F | regulation of endothelial cell proliferation | ANG1\_BOVIN | ANG2\_MOUSE | ANG3\_MOUSE | ANG4\_MOUSE | ANGI\_MOUSE | DOPO\_HUMAN | Q5WRG2\_RAT | GO:0050679 | 0.00465549348230913 | 15/3222 | 7/552 | 0.00757380228157741 | 1 | T | T | F | F | positive regulation of epithelial cell proliferation | ANG1\_BOVIN | ANG2\_MOUSE | ANG3\_MOUSE | ANG4\_MOUSE | ANGI\_MOUSE | HYAL1\_HUMAN | Q5WRG2\_RAT | GO:0010558 | 0.0338299193047796 | 109/3222 | 29/552 | 0.0076222429809895 | 1 | T | T | F | F | negative regulation of macromolecule biosynthetic process | ANG1\_BOVIN | ANG3\_MOUSE | ANGI\_MOUSE | E0CX04\_MOMBA | G3I1H5\_CRIGR | GBA1\_HUMAN | GRAC\_MOUSE | LGMN\_MOUSE | O04358\_IRIHO | PA2GX\_HUMAN | PGRP1\_CAMDR | PPA5\_HUMAN | PPA5\_RAT | Q2QEH4\_SAPOF | Q94BW3\_CINCA | RIP0\_DIACA | RIP1\_BRYDI | RIP1\_HORVU | RIP1\_MOMCH | RIP1\_PHYAM | RIP2\_PHYAM | RIP3\_MOMCH | RIPA\_PHYAM | RIPG\_SURMU | RIPL1\_PHYDI | RIPL2\_PHYDI | RIPT\_TRIKI | TRFL\_BUBBU | TRFL\_HORSE | GO:0006639 | 0.00806952203600248 | 26/3222 | 10/552 | 0.00776577016461461 | 1 | T | T | F | F | acylglycerol metabolic process | ANG1\_BOVIN | ANG2\_MOUSE | ANG3\_MOUSE | ANG4\_MOUSE | ANGI\_MOUSE | LIPG\_HUMAN | LIPR2\_HUMAN | LIPR2\_RAT | PAG15\_HUMAN | Q5WRG2\_RAT | GO:0002920 | 0.00186219739292365 | 6/3222 | 4/552 | 0.00956421365123264 | 1 | T | F | F | F | regulation of humoral immune response | GRASS\_DROME | KLK7\_HUMAN | KLK7\_MOUSE | PGPSA\_DROME | GO:0002760 | 0.00186219739292365 | 6/3222 | 4/552 | 0.00956421365123264 | 1 | T | F | F | F | positive regulation of antimicrobial humoral response | GRASS\_DROME | KLK7\_HUMAN | KLK7\_MOUSE | PGPSA\_DROME | GO:0002922 | 0.00186219739292365 | 6/3222 | 4/552 | 0.00956421365123264 | 1 | T | F | F | F | positive regulation of humoral immune response | GRASS\_DROME | KLK7\_HUMAN | KLK7\_MOUSE | PGPSA\_DROME | GO:0002759 | 0.00186219739292365 | 6/3222 | 4/552 | 0.00956421365123264 | 1 | T | F | F | F | regulation of antimicrobial humoral response | GRASS\_DROME | KLK7\_HUMAN | KLK7\_MOUSE | PGPSA\_DROME | GO:0051246 | 0.0403476101800124 | 130/3222 | 33/552 | 0.0097798789413118 | 1 | T | F | F | F | regulation of protein metabolic process | ANG1\_BOVIN | ANG2\_MOUSE | ANG3\_MOUSE | ANG4\_MOUSE | ANGI\_MOUSE | CATD\_RAT | CATH\_HUMAN | E0CX04\_MOMBA | ENPP2\_HUMAN | ENPP2\_RAT | G3I1H5\_CRIGR | GBA1\_HUMAN | GRAC\_MOUSE | LGMN\_MOUSE | O04358\_IRIHO | PA21B\_PIG | PA2GX\_HUMAN | PGH2\_HUMAN | Q2QEH4\_SAPOF | Q5WRG2\_RAT | Q94BW3\_CINCA | RIP0\_DIACA | RIP1\_BRYDI | RIP1\_HORVU | RIP1\_MOMCH | RIP1\_PHYAM | RIP2\_PHYAM | RIP3\_MOMCH | RIPA\_PHYAM | RIPG\_SURMU | RIPL1\_PHYDI | RIPL2\_PHYDI | RIPT\_TRIKI | GO:0090501 | 0.00279329608938547 | 9/3222 | 5/552 | 0.00998677291237379 | 1 | T | F | F | F | obsolete RNA phosphodiester bond hydrolysis | Q5WRG2\_RAT | RNAS4\_HUMAN | RNAS6\_HUMAN | RNS1B\_RAT | RNS\_BOVIN | GO:0007613 | 0.00279329608938547 | 9/3222 | 5/552 | 0.00998677291237379 | 1 | T | F | F | F | memory | DOPO\_HUMAN | G3I1H5\_CRIGR | KLK8\_MOUSE | LGMN\_MOUSE | PGH2\_HUMAN | GO:0042327 | 0.00713842333954066 | 23/3222 | 9/552 | 0.0100519620835813 | 1 | T | F | F | F | positive regulation of phosphorylation | ANG2\_MOUSE | ANG3\_MOUSE | ANG4\_MOUSE | ANGI\_MOUSE | ENPP2\_HUMAN | ENPP2\_RAT | PA21B\_PIG | PGH2\_HUMAN | Q5WRG2\_RAT | GO:0006665 | 0.00713842333954066 | 23/3222 | 9/552 | 0.0100519620835813 | 1 | T | F | F | F | sphingolipid metabolic process | AGAL\_HUMAN | ANAG\_HUMAN | ASAH1\_BALAS | ENPP2\_HUMAN | ENPP2\_RAT | GBA1\_HUMAN | PAG15\_HUMAN | PPT1\_HUMAN | SIA\_ASPFU | GO:0031401 | 0.00837988826815642 | 27/3222 | 10/552 | 0.0104893837656848 | 1 | T | F | F | F | positive regulation of protein modification process | ANG2\_MOUSE | ANG3\_MOUSE | ANG4\_MOUSE | ANGI\_MOUSE | ENPP2\_HUMAN | ENPP2\_RAT | GBA1\_HUMAN | PA21B\_PIG | PGH2\_HUMAN | Q5WRG2\_RAT | GO:0050896 | 0.23618870266915 | 761/3222 | 152/552 | 0.0107311102722333 | 1 | T | F | F | F | response to stimulus | A0A087WNH2\_FICBE | A0A0A0Y4H8\_TRAFO | A0A1S4NYF8\_PANVG | A0A3L6SKP5\_PANMI | A6PZ97\_SALSA | AGAL\_ORYSJ | ANAG\_HUMAN | ANG1\_BOVIN | ANG2\_MOUSE | ANG3\_MOUSE | ANG4\_MOUSE | ANGI\_MOUSE | AOC1\_HUMAN | AOCX\_BOVIN | B9TU22\_GADMO | CAH6\_HUMAN | CARP1\_CANAL | CAT3\_NEUCR | CATD\_RAT | CATH\_HUMAN | CBPN\_HUMAN | CEL2A\_PIG | CERU\_RAT | CFAD\_MOUSE | CHI2\_HORVU | CHI2\_ORYSJ | CHI4\_CRYJA | CHIC\_ARATH | CHIC\_SECCE | CHIL3\_MOUSE | D1MPT2\_ROYRE | DABA\_PSEMU | DIR\_GLYEC | DOPO\_HUMAN | E0CX04\_MOMBA | E13B\_HORVU | ECP\_HUMAN | ENPP2\_HUMAN | ENPP2\_RAT | G3I1H5\_CRIGR | GBA1\_HUMAN | GPX3\_HUMAN | GPX5\_HUMAN | GPX6\_MOUSE | GRAA\_HUMAN | GRAC\_MOUSE | GRASS\_DROME | HYAL1\_HUMAN | K7CID1\_PANTR | K7N5L9\_RAPSA | KATG2\_MAGO7 | KLK8\_MOUSE | L8ICE9\_9CETA | LALBA\_BOVIN | LGMN\_MOUSE | LICH\_HUMAN | LIG2\_PHACH | LIG4\_PHACH | LIG8\_PHACH | LIPR2\_RAT | LYG\_STRCA | LYSC1\_ANAPL | LYSC1\_CANLF | LYSC1\_HORSE | LYSC2\_BOVIN | LYSC2\_ONCMY | LYSC\_COTJA | LYSC\_EQUAS | LYSC\_NUMME | LYSC\_OPIHO | LYSC\_PELSI | LYS\_BOMMO | LYS\_RUDPH | MAN12\_PENCI | MMP1\_PIG | NCS\_THLFG | O04358\_IRIHO | O22443\_SOYBN | O81934\_CANEN | OXLA\_BOTAT | OXLA\_CALRH | OXLA\_GLOHA | PA21B\_BOVIN | PA21B\_PIG | PA2A\_BOTJR | PA2B3\_BOTAS | PA2GA\_HUMAN | PA2GE\_HUMAN | PA2GX\_HUMAN | PA2H1\_BOTBZ | PA2H1\_BOTJR | PA2H2\_BOTAS | PA2H2\_BOTMO | PA2H\_BOTPA | PCP\_HUMAN | PEM1\_PHACH | PER1A\_ARMRU | PER1\_ARAHY | PER1\_SORBI | PER53\_ARATH | PER59\_ARATH | PERL\_BOVIN | PERL\_BUBBU | PERL\_CAPHI | PER\_ARTRA | PER\_COPCI | PGH2\_HUMAN | PGPSA\_DROME | PGRP1\_CAMDR | POXA\_DICDI | PPA5\_HUMAN | PPA5\_RAT | PPAF1\_HOLDI | PPT1\_HUMAN | PRTN3\_HUMAN | PTGDS\_HUMAN | PTGDS\_MOUSE | Q2QEH4\_SAPOF | Q40069\_HORVU | Q5WRG2\_RAT | Q60FD2\_9APHY | Q70C53\_SOLTU | Q86RS6\_MANSE | Q8H0C9\_VIGUN | Q94BW3\_CINCA | Q96X16\_PICPA | Q9FUH3\_VIGUS | RENI\_RAT | RIP0\_DIACA | RIP1\_BRYDI | RIP1\_HORVU | RIP1\_MOMCH | RIP1\_PHYAM | RIP2\_PHYAM | RIP3\_MOMCH | RIPA\_PHYAM | RIPG\_SURMU | RIPL1\_PHYDI | RIPL2\_PHYDI | RIPT\_TRIKI | RNAS4\_HUMAN | RNAS4\_PIG | RNAS6\_HUMAN | RNS1B\_RAT | RNSL3\_DANRE | RNS\_BOVIN | RNT2\_HUMAN | TRFL\_BUBBU | TRFL\_HORSE | TTHY\_CHICK | VPL1\_PLEER | VPL2\_PLEER | GO:0008284 | 0.0189323401613904 | 61/3222 | 18/552 | 0.0111027085851957 | 1 | T | F | F | F | positive regulation of cell population proliferation | ANG1\_BOVIN | ANG2\_MOUSE | ANG3\_MOUSE | ANG4\_MOUSE | ANGI\_MOUSE | CATH\_HUMAN | ENPP2\_HUMAN | ENPP2\_RAT | G3I1H5\_CRIGR | HYAL1\_HUMAN | LGMN\_MOUSE | PA21B\_BOVIN | PA21B\_PIG | PGH2\_HUMAN | PRTN3\_HUMAN | Q5WRG2\_RAT | TRFL\_BUBBU | TRFL\_HORSE | GO:0031327 | 0.0363128491620112 | 117/3222 | 30/552 | 0.0116339858605996 | 1 | T | F | F | F | negative regulation of cellular biosynthetic process | AGAL\_HUMAN | ANG1\_BOVIN | ANG3\_MOUSE | ANGI\_MOUSE | E0CX04\_MOMBA | G3I1H5\_CRIGR | GBA1\_HUMAN | GRAC\_MOUSE | LGMN\_MOUSE | O04358\_IRIHO | PA2GX\_HUMAN | PGRP1\_CAMDR | PPA5\_HUMAN | PPA5\_RAT | Q2QEH4\_SAPOF | Q94BW3\_CINCA | RIP0\_DIACA | RIP1\_BRYDI | RIP1\_HORVU | RIP1\_MOMCH | RIP1\_PHYAM | RIP2\_PHYAM | RIP3\_MOMCH | RIPA\_PHYAM | RIPG\_SURMU | RIPL1\_PHYDI | RIPL2\_PHYDI | RIPT\_TRIKI | TRFL\_BUBBU | TRFL\_HORSE | GO:0007586 | 0.00403476101800124 | 13/3222 | 6/552 | 0.0142849735702559 | 1 | T | F | F | F | digestion | CHIA\_HUMAN | CHYM\_CAMDR | LIPR2\_HUMAN | LYSC2\_BOVIN | TRY1\_GADMO | TRY3\_SALSA | GO:0060249 | 0.00527622594661701 | 17/3222 | 7/552 | 0.0167424839388119 | 1 | T | F | F | F | anatomical structure homeostasis | ANAG\_HUMAN | LICH\_HUMAN | PGH2\_HUMAN | PPA5\_HUMAN | PPA5\_PIG | PPA5\_RAT | TPP1\_HUMAN | GO:0001894 | 0.00527622594661701 | 17/3222 | 7/552 | 0.0167424839388119 | 1 | T | F | F | F | tissue homeostasis | ANAG\_HUMAN | LICH\_HUMAN | PGH2\_HUMAN | PPA5\_HUMAN | PPA5\_PIG | PPA5\_RAT | TPP1\_HUMAN | GO:0046486 | 0.0183116076970826 | 59/3222 | 17/552 | 0.0170737513778417 | 1 | T | F | F | F | glycerolipid metabolic process | ANG1\_BOVIN | ANG2\_MOUSE | ANG3\_MOUSE | ANG4\_MOUSE | ANGI\_MOUSE | ENPP2\_HUMAN | ENPP2\_RAT | LIPG\_HUMAN | LIPR2\_HUMAN | LIPR2\_RAT | PA21B\_BOVIN | PA21B\_PIG | PA2GA\_HUMAN | PA2GE\_HUMAN | PA2GX\_HUMAN | PAG15\_HUMAN | Q5WRG2\_RAT | GO:0008154 | 0.00124146492861577 | 4/3222 | 3/552 | 0.0174622957200355 | 1 | T | F | F | F | actin polymerization or depolymerization | ANG1\_BOVIN | ANGI\_MOUSE | Q5WRG2\_RAT | GO:0034367 | 0.00124146492861577 | 4/3222 | 3/552 | 0.0174622957200355 | 1 | T | F | F | F | protein-containing complex remodeling | PA2GA\_HUMAN | PA2GE\_HUMAN | PA2GX\_HUMAN | GO:0034374 | 0.00124146492861577 | 4/3222 | 3/552 | 0.0174622957200355 | 1 | T | F | F | F | low-density lipoprotein particle remodeling | PA2GA\_HUMAN | PA2GE\_HUMAN | PA2GX\_HUMAN | GO:0030574 | 0.00124146492861577 | 4/3222 | 3/552 | 0.0174622957200355 | 1 | T | F | F | F | collagen catabolic process | COGS\_HYPLI | MMP1\_PIG | PRTN3\_HUMAN | GO:0034368 | 0.00124146492861577 | 4/3222 | 3/552 | 0.0174622957200355 | 1 | T | F | F | F | protein-lipid complex remodeling | PA2GA\_HUMAN | PA2GE\_HUMAN | PA2GX\_HUMAN | GO:0030041 | 0.00124146492861577 | 4/3222 | 3/552 | 0.0174622957200355 | 1 | T | F | F | F | actin filament polymerization | ANG1\_BOVIN | ANGI\_MOUSE | Q5WRG2\_RAT | GO:0019882 | 0.00124146492861577 | 4/3222 | 3/552 | 0.0174622957200355 | 1 | T | F | F | F | antigen processing and presentation | CATH\_HUMAN | GBA1\_HUMAN | GILT\_MOUSE | GO:0031341 | 0.00124146492861577 | 4/3222 | 3/552 | 0.0174622957200355 | 1 | T | F | F | F | regulation of cell killing | DNAS1\_HUMAN | DNSL3\_HUMAN | OXLA\_BOTAT | GO:0050688 | 0.00124146492861577 | 4/3222 | 3/552 | 0.0174622957200355 | 1 | T | F | F | F | regulation of defense response to virus | RIP0\_DIACA | RIP3\_MOMCH | RIPT\_TRIKI | GO:0034369 | 0.00124146492861577 | 4/3222 | 3/552 | 0.0174622957200355 | 1 | T | F | F | F | plasma lipoprotein particle remodeling | PA2GA\_HUMAN | PA2GE\_HUMAN | PA2GX\_HUMAN | GO:0043112 | 0.00124146492861577 | 4/3222 | 3/552 | 0.0174622957200355 | 1 | T | F | F | F | receptor metabolic process | CATD\_RAT | G3I1H5\_CRIGR | LGMN\_MOUSE | GO:0010562 | 0.00900062073246431 | 29/3222 | 10/552 | 0.0180326905494802 | 1 | T | F | F | F | positive regulation of phosphorus metabolic process | ANG2\_MOUSE | ANG3\_MOUSE | ANG4\_MOUSE | ANGI\_MOUSE | ENPP2\_HUMAN | ENPP2\_RAT | GBA1\_HUMAN | PA21B\_PIG | PGH2\_HUMAN | Q5WRG2\_RAT | GO:0045937 | 0.00900062073246431 | 29/3222 | 10/552 | 0.0180326905494802 | 1 | T | F | F | F | positive regulation of phosphate metabolic process | ANG2\_MOUSE | ANG3\_MOUSE | ANG4\_MOUSE | ANGI\_MOUSE | ENPP2\_HUMAN | ENPP2\_RAT | GBA1\_HUMAN | PA21B\_PIG | PGH2\_HUMAN | Q5WRG2\_RAT | GO:0009890 | 0.0375543140906269 | 121/3222 | 30/552 | 0.0186693580385087 | 1 | T | F | F | F | negative regulation of biosynthetic process | AGAL\_HUMAN | ANG1\_BOVIN | ANG3\_MOUSE | ANGI\_MOUSE | E0CX04\_MOMBA | G3I1H5\_CRIGR | GBA1\_HUMAN | GRAC\_MOUSE | LGMN\_MOUSE | O04358\_IRIHO | PA2GX\_HUMAN | PGRP1\_CAMDR | PPA5\_HUMAN | PPA5\_RAT | Q2QEH4\_SAPOF | Q94BW3\_CINCA | RIP0\_DIACA | RIP1\_BRYDI | RIP1\_HORVU | RIP1\_MOMCH | RIP1\_PHYAM | RIP2\_PHYAM | RIP3\_MOMCH | RIPA\_PHYAM | RIPG\_SURMU | RIPL1\_PHYDI | RIPL2\_PHYDI | RIPT\_TRIKI | TRFL\_BUBBU | TRFL\_HORSE | GO:0002532 | 0.00217256362507759 | 7/3222 | 4/552 | 0.019315347471839 | 1 | T | F | F | F | production of molecular mediator involved in inflammatory response | CHIA\_HUMAN | CHIL3\_MOUSE | LICH\_HUMAN | PA2GX\_HUMAN | GO:1903556 | 0.00217256362507759 | 7/3222 | 4/552 | 0.019315347471839 | 1 | T | F | F | F | negative regulation of tumor necrosis factor superfamily cytokine production | PPA5\_HUMAN | PPA5\_RAT | TRFL\_BUBBU | TRFL\_HORSE | GO:0045453 | 0.00217256362507759 | 7/3222 | 4/552 | 0.019315347471839 | 1 | T | F | F | F | bone resorption | PPA5\_HUMAN | PPA5\_PIG | PPA5\_RAT | TPP1\_HUMAN | GO:1900424 | 0.00217256362507759 | 7/3222 | 4/552 | 0.019315347471839 | 1 | T | F | F | F | regulation of defense response to bacterium | GRASS\_DROME | KLK7\_HUMAN | KLK7\_MOUSE | PGPSA\_DROME | GO:0002443 | 0.00217256362507759 | 7/3222 | 4/552 | 0.019315347471839 | 1 | T | F | F | F | leukocyte mediated immunity | CATH\_HUMAN | DOPO\_HUMAN | GRAC\_MOUSE | PA21B\_PIG | GO:0032963 | 0.00217256362507759 | 7/3222 | 4/552 | 0.019315347471839 | 1 | T | F | F | F | collagen metabolic process | ANAG\_HUMAN | COGS\_HYPLI | MMP1\_PIG | PRTN3\_HUMAN | GO:0032101 | 0.0257603972687772 | 83/3222 | 22/552 | 0.0196616065196605 | 1 | F | F | F | F | regulation of response to external stimulus | ANAG\_HUMAN | AOAH\_MOUSE | DNAS1\_HUMAN | DNSL3\_HUMAN | G3I1H5\_CRIGR | GBA1\_HUMAN | GRASS\_DROME | KLK7\_HUMAN | KLK7\_MOUSE | KLK8\_MOUSE | LGMN\_MOUSE | PA2GA\_HUMAN | PA2GX\_HUMAN | PGH2\_HUMAN | PGPSA\_DROME | PPA5\_HUMAN | PPA5\_RAT | RIP0\_DIACA | RIP3\_MOMCH | RIPT\_TRIKI | TRFL\_BUBBU | TRFL\_HORSE | GO:0007033 | 0.00434512725015518 | 14/3222 | 6/552 | 0.0214534877192637 | 1 | F | F | F | F | vacuole organization | ANAG\_HUMAN | CATD\_RAT | GBA1\_HUMAN | LICH\_HUMAN | PPAP\_RAT | TPP1\_HUMAN | GO:0032774 | 0.00434512725015518 | 14/3222 | 6/552 | 0.0214534877192637 | 1 | F | F | F | F | RNA biosynthetic process | ANG1\_BOVIN | ANG2\_MOUSE | ANG3\_MOUSE | ANG4\_MOUSE | ANGI\_MOUSE | Q5WRG2\_RAT | GO:0045860 | 0.00434512725015518 | 14/3222 | 6/552 | 0.0214534877192637 | 1 | F | F | F | F | positive regulation of protein kinase activity | ANG2\_MOUSE | ANG3\_MOUSE | ANG4\_MOUSE | ANGI\_MOUSE | PA21B\_PIG | Q5WRG2\_RAT | GO:0005984 | 0.00806952203600248 | 26/3222 | 9/552 | 0.023796468266013 | 1 | F | F | F | F | disaccharide metabolic process | AMY1\_ORYSJ | BGALA\_ASPNC | BGALA\_ASPOR | BGALA\_PENSQ | IDUA\_HUMAN | LALBA\_BOVIN | LALBA\_CAPHI | LALBA\_CAVPO | LALBA\_PAPCY | GO:0051248 | 0.026381129733085 | 85/3222 | 22/552 | 0.0256518505091033 | 1 | F | F | F | F | negative regulation of protein metabolic process | ANG1\_BOVIN | ANG3\_MOUSE | ANGI\_MOUSE | E0CX04\_MOMBA | GBA1\_HUMAN | GRAC\_MOUSE | O04358\_IRIHO | PGH2\_HUMAN | Q2QEH4\_SAPOF | Q94BW3\_CINCA | RIP0\_DIACA | RIP1\_BRYDI | RIP1\_HORVU | RIP1\_MOMCH | RIP1\_PHYAM | RIP2\_PHYAM | RIP3\_MOMCH | RIPA\_PHYAM | RIPG\_SURMU | RIPL1\_PHYDI | RIPL2\_PHYDI | RIPT\_TRIKI | GO:0045862 | 0.00341402855369336 | 11/3222 | 5/552 | 0.0271664824684607 | 1 | F | F | F | F | positive regulation of proteolysis | CATD\_RAT | CATH\_HUMAN | G3I1H5\_CRIGR | GBA1\_HUMAN | LGMN\_MOUSE | GO:0030099 | 0.00341402855369336 | 11/3222 | 5/552 | 0.0271664824684607 | 1 | F | F | F | F | myeloid cell differentiation | ANAG\_HUMAN | GBA1\_HUMAN | LICH\_HUMAN | PPA5\_RAT | PRTN3\_HUMAN | GO:0035729 | 0.000620732464307883 | 2/3222 | 2/552 | 0.0293072059118488 | 1 | F | F | F | F | cellular response to hepatocyte growth factor stimulus | G3I1H5\_CRIGR | LGMN\_MOUSE | GO:0032929 | 0.000620732464307883 | 2/3222 | 2/552 | 0.0293072059118488 | 1 | F | F | F | F | negative regulation of superoxide anion generation | PPA5\_HUMAN | PPA5\_RAT | GO:0044406 | 0.000620732464307883 | 2/3222 | 2/552 | 0.0293072059118488 | 1 | F | F | F | F | adhesion of symbiont to host | CARP1\_CANAL | EXG1\_CANAL | GO:1900229 | 0.000620732464307883 | 2/3222 | 2/552 | 0.0293072059118488 | 1 | F | F | F | F | negative regulation of single-species biofilm formation in or on host organism | TRFL\_BUBBU | TRFL\_HORSE | GO:0097202 | 0.000620732464307883 | 2/3222 | 2/552 | 0.0293072059118488 | 1 | F | F | F | F | activation of cysteine-type endopeptidase activity | G3I1H5\_CRIGR | LGMN\_MOUSE | GO:1902732 | 0.000620732464307883 | 2/3222 | 2/552 | 0.0293072059118488 | 1 | F | F | F | F | positive regulation of chondrocyte proliferation | TRFL\_BUBBU | TRFL\_HORSE | GO:2001204 | 0.000620732464307883 | 2/3222 | 2/552 | 0.0293072059118488 | 1 | F | F | F | F | regulation of osteoclast development | TRFL\_BUBBU | TRFL\_HORSE | GO:1905146 | 0.000620732464307883 | 2/3222 | 2/552 | 0.0293072059118488 | 1 | F | F | F | F | lysosomal protein catabolic process | DPP2\_HUMAN | TPP1\_HUMAN | GO:1901184 | 0.000620732464307883 | 2/3222 | 2/552 | 0.0293072059118488 | 1 | F | F | F | F | regulation of ERBB signaling pathway | G3I1H5\_CRIGR | LGMN\_MOUSE | GO:0007042 | 0.000620732464307883 | 2/3222 | 2/552 | 0.0293072059118488 | 1 | F | F | F | F | lysosomal lumen acidification | PPT1\_BOVIN | PPT1\_HUMAN | GO:0006963 | 0.000620732464307883 | 2/3222 | 2/552 | 0.0293072059118488 | 1 | F | F | F | F | positive regulation of antibacterial peptide biosynthetic process | GRASS\_DROME | PGPSA\_DROME | GO:2000308 | 0.000620732464307883 | 2/3222 | 2/552 | 0.0293072059118488 | 1 | F | F | F | F | negative regulation of tumor necrosis factor (ligand) superfamily member 11 production | TRFL\_BUBBU | TRFL\_HORSE | GO:0035633 | 0.000620732464307883 | 2/3222 | 2/552 | 0.0293072059118488 | 1 | F | F | F | F | maintenance of blood-brain barrier | ANAG\_HUMAN | PGH2\_HUMAN | GO:0035751 | 0.000620732464307883 | 2/3222 | 2/552 | 0.0293072059118488 | 1 | F | F | F | F | regulation of lysosomal lumen pH | PPT1\_BOVIN | PPT1\_HUMAN | GO:0002761 | 0.000620732464307883 | 2/3222 | 2/552 | 0.0293072059118488 | 1 | F | F | F | F | regulation of myeloid leukocyte differentiation | TRFL\_BUBBU | TRFL\_HORSE | GO:0007039 | 0.000620732464307883 | 2/3222 | 2/552 | 0.0293072059118488 | 1 | F | F | F | F | protein catabolic process in the vacuole | DPP2\_HUMAN | TPP1\_HUMAN | GO:0002762 | 0.000620732464307883 | 2/3222 | 2/552 | 0.0293072059118488 | 1 | F | F | F | F | negative regulation of myeloid leukocyte differentiation | TRFL\_BUBBU | TRFL\_HORSE | GO:2001026 | 0.000620732464307883 | 2/3222 | 2/552 | 0.0293072059118488 | 1 | F | F | F | F | regulation of endothelial cell chemotaxis | G3I1H5\_CRIGR | LGMN\_MOUSE | GO:0045671 | 0.000620732464307883 | 2/3222 | 2/552 | 0.0293072059118488 | 1 | F | F | F | F | negative regulation of osteoclast differentiation | TRFL\_BUBBU | TRFL\_HORSE | GO:0140507 | 0.000620732464307883 | 2/3222 | 2/552 | 0.0293072059118488 | 1 | F | F | F | F | granzyme-mediated programmed cell death signaling pathway | GRAA\_HUMAN | GRAC\_MOUSE | GO:0006658 | 0.000620732464307883 | 2/3222 | 2/552 | 0.0293072059118488 | 1 | F | F | F | F | phosphatidylserine metabolic process | PA2GX\_HUMAN | PAG15\_HUMAN | GO:1904635 | 0.000620732464307883 | 2/3222 | 2/552 | 0.0293072059118488 | 1 | F | F | F | F | positive regulation of podocyte apoptotic process | PA21B\_BOVIN | PA21B\_PIG | GO:0009664 | 0.000620732464307883 | 2/3222 | 2/552 | 0.0293072059118488 | 1 | F | F | F | F | plant-type cell wall organization | ADPG2\_ARATH | Q9LYJ5\_ARATH | GO:2001028 | 0.000620732464307883 | 2/3222 | 2/552 | 0.0293072059118488 | 1 | F | F | F | F | positive regulation of endothelial cell chemotaxis | G3I1H5\_CRIGR | LGMN\_MOUSE | GO:0160032 | 0.000620732464307883 | 2/3222 | 2/552 | 0.0293072059118488 | 1 | F | F | F | F | Toll receptor ligand protein activation cascade | GRASS\_DROME | PGPSA\_DROME | GO:1900159 | 0.000620732464307883 | 2/3222 | 2/552 | 0.0293072059118488 | 1 | F | F | F | F | positive regulation of bone mineralization involved in bone maturation | TRFL\_BUBBU | TRFL\_HORSE | GO:0033619 | 0.000620732464307883 | 2/3222 | 2/552 | 0.0293072059118488 | 1 | F | F | F | F | membrane protein proteolysis | CATH\_HUMAN | PRTN3\_HUMAN | GO:1900191 | 0.000620732464307883 | 2/3222 | 2/552 | 0.0293072059118488 | 1 | F | F | F | F | negative regulation of single-species biofilm formation | TRFL\_BUBBU | TRFL\_HORSE | GO:0036335 | 0.000620732464307883 | 2/3222 | 2/552 | 0.0293072059118488 | 1 | F | F | F | F | intestinal stem cell homeostasis | PA2GA\_HUMAN | PA2GX\_HUMAN | GO:1903328 | 0.000620732464307883 | 2/3222 | 2/552 | 0.0293072059118488 | 1 | F | F | F | F | positive regulation of tRNA metabolic process | ANGI\_MOUSE | Q5WRG2\_RAT | GO:0040015 | 0.000620732464307883 | 2/3222 | 2/552 | 0.0293072059118488 | 1 | F | F | F | F | negative regulation of multicellular organism growth | G3I1H5\_CRIGR | LGMN\_MOUSE | GO:2000307 | 0.000620732464307883 | 2/3222 | 2/552 | 0.0293072059118488 | 1 | F | F | F | F | regulation of tumor necrosis factor (ligand) superfamily member 11 production | TRFL\_BUBBU | TRFL\_HORSE | GO:0051656 | 0.000620732464307883 | 2/3222 | 2/552 | 0.0293072059118488 | 1 | F | F | F | F | establishment of organelle localization | PTGDS\_HUMAN | PTGDS\_MOUSE | GO:1900228 | 0.000620732464307883 | 2/3222 | 2/552 | 0.0293072059118488 | 1 | F | F | F | F | regulation of single-species biofilm formation in or on host organism | TRFL\_BUBBU | TRFL\_HORSE | GO:1902372 | 0.000620732464307883 | 2/3222 | 2/552 | 0.0293072059118488 | 1 | F | F | F | F | positive regulation of tRNA catabolic process | ANGI\_MOUSE | Q5WRG2\_RAT | GO:0002807 | 0.000620732464307883 | 2/3222 | 2/552 | 0.0293072059118488 | 1 | F | F | F | F | positive regulation of antimicrobial peptide biosynthetic process | GRASS\_DROME | PGPSA\_DROME | GO:0035728 | 0.000620732464307883 | 2/3222 | 2/552 | 0.0293072059118488 | 1 | F | F | F | F | response to hepatocyte growth factor | G3I1H5\_CRIGR | LGMN\_MOUSE | GO:0043299 | 0.000620732464307883 | 2/3222 | 2/552 | 0.0293072059118488 | 1 | F | F | F | F | leukocyte degranulation | PTGDS\_HUMAN | PTGDS\_MOUSE | GO:0042119 | 0.000620732464307883 | 2/3222 | 2/552 | 0.0293072059118488 | 1 | F | F | F | F | neutrophil activation | DNAS1\_HUMAN | DNSL3\_HUMAN | GO:0097066 | 0.000620732464307883 | 2/3222 | 2/552 | 0.0293072059118488 | 1 | F | F | F | F | response to thyroid hormone | CATH\_HUMAN | GBA1\_HUMAN | GO:0009827 | 0.000620732464307883 | 2/3222 | 2/552 | 0.0293072059118488 | 1 | F | F | F | F | plant-type cell wall modification | ADPG2\_ARATH | Q9LYJ5\_ARATH | GO:0031221 | 0.000620732464307883 | 2/3222 | 2/552 | 0.0293072059118488 | 1 | F | F | F | F | arabinan metabolic process | ABFB\_ASPKW | XYND\_EMENI | GO:0030207 | 0.000620732464307883 | 2/3222 | 2/552 | 0.0293072059118488 | 1 | F | F | F | F | chondroitin sulfate catabolic process | BGLR\_HUMAN | HYAL1\_HUMAN | GO:0071669 | 0.000620732464307883 | 2/3222 | 2/552 | 0.0293072059118488 | 1 | F | F | F | F | plant-type cell wall organization or biogenesis | ADPG2\_ARATH | Q9LYJ5\_ARATH | GO:0043303 | 0.000620732464307883 | 2/3222 | 2/552 | 0.0293072059118488 | 1 | F | F | F | F | mast cell degranulation | PTGDS\_HUMAN | PTGDS\_MOUSE | GO:0002275 | 0.000620732464307883 | 2/3222 | 2/552 | 0.0293072059118488 | 1 | F | F | F | F | myeloid cell activation involved in immune response | DNAS1\_HUMAN | DNSL3\_HUMAN | GO:0002283 | 0.000620732464307883 | 2/3222 | 2/552 | 0.0293072059118488 | 1 | F | F | F | F | neutrophil activation involved in immune response | DNAS1\_HUMAN | DNSL3\_HUMAN | GO:2001205 | 0.000620732464307883 | 2/3222 | 2/552 | 0.0293072059118488 | 1 | F | F | F | F | negative regulation of osteoclast development | TRFL\_BUBBU | TRFL\_HORSE | GO:0048260 | 0.000620732464307883 | 2/3222 | 2/552 | 0.0293072059118488 | 1 | F | F | F | F | positive regulation of receptor-mediated endocytosis | PPT1\_BOVIN | PPT1\_HUMAN | GO:1900157 | 0.000620732464307883 | 2/3222 | 2/552 | 0.0293072059118488 | 1 | F | F | F | F | regulation of bone mineralization involved in bone maturation | TRFL\_BUBBU | TRFL\_HORSE | GO:1901185 | 0.000620732464307883 | 2/3222 | 2/552 | 0.0293072059118488 | 1 | F | F | F | F | negative regulation of ERBB signaling pathway | G3I1H5\_CRIGR | LGMN\_MOUSE | GO:0010936 | 0.000620732464307883 | 2/3222 | 2/552 | 0.0293072059118488 | 1 | F | F | F | F | negative regulation of macrophage cytokine production | PPA5\_HUMAN | PPA5\_RAT | GO:0048549 | 0.000620732464307883 | 2/3222 | 2/552 | 0.0293072059118488 | 1 | F | F | F | F | positive regulation of pinocytosis | PPT1\_BOVIN | PPT1\_HUMAN | GO:1904633 | 0.000620732464307883 | 2/3222 | 2/552 | 0.0293072059118488 | 1 | F | F | F | F | regulation of podocyte apoptotic process | PA21B\_BOVIN | PA21B\_PIG | GO:0002805 | 0.000620732464307883 | 2/3222 | 2/552 | 0.0293072059118488 | 1 | F | F | F | F | regulation of antimicrobial peptide biosynthetic process | GRASS\_DROME | PGPSA\_DROME | GO:0033690 | 0.000620732464307883 | 2/3222 | 2/552 | 0.0293072059118488 | 1 | F | F | F | F | positive regulation of osteoblast proliferation | TRFL\_BUBBU | TRFL\_HORSE | GO:0032695 | 0.000620732464307883 | 2/3222 | 2/552 | 0.0293072059118488 | 1 | F | F | F | F | negative regulation of interleukin-12 production | PPA5\_HUMAN | PPA5\_RAT | GO:0097264 | 0.000620732464307883 | 2/3222 | 2/552 | 0.0293072059118488 | 1 | F | F | F | F | self proteolysis | G3I1H5\_CRIGR | LGMN\_MOUSE | GO:1901142 | 0.000620732464307883 | 2/3222 | 2/552 | 0.0293072059118488 | 1 | F | F | F | F | insulin metabolic process | CATD\_RAT | CEL2A\_PIG | GO:0031222 | 0.000620732464307883 | 2/3222 | 2/552 | 0.0293072059118488 | 1 | F | F | F | F | arabinan catabolic process | ABFB\_ASPKW | XYND\_EMENI | GO:0007035 | 0.000620732464307883 | 2/3222 | 2/552 | 0.0293072059118488 | 1 | F | F | F | F | vacuolar acidification | PPT1\_BOVIN | PPT1\_HUMAN | GO:0002808 | 0.000620732464307883 | 2/3222 | 2/552 | 0.0293072059118488 | 1 | F | F | F | F | regulation of antibacterial peptide biosynthetic process | GRASS\_DROME | PGPSA\_DROME | GO:1900190 | 0.000620732464307883 | 2/3222 | 2/552 | 0.0293072059118488 | 1 | F | F | F | F | regulation of single-species biofilm formation | TRFL\_BUBBU | TRFL\_HORSE | GO:0032801 | 0.000620732464307883 | 2/3222 | 2/552 | 0.0293072059118488 | 1 | F | F | F | F | receptor catabolic process | G3I1H5\_CRIGR | LGMN\_MOUSE | GO:0046900 | 0.000620732464307883 | 2/3222 | 2/552 | 0.0293072059118488 | 1 | F | F | F | F | tetrahydrofolylpolyglutamate metabolic process | GGH\_HUMAN | Q6NY42\_DANRE | GO:0010935 | 0.000620732464307883 | 2/3222 | 2/552 | 0.0293072059118488 | 1 | F | F | F | F | regulation of macrophage cytokine production | PPA5\_HUMAN | PPA5\_RAT | GO:0000737 | 0.000620732464307883 | 2/3222 | 2/552 | 0.0293072059118488 | 1 | F | F | F | F | obsolete DNA catabolic process, endonucleolytic | DNAS1\_HUMAN | DNSL3\_HUMAN | GO:0045670 | 0.000620732464307883 | 2/3222 | 2/552 | 0.0293072059118488 | 1 | F | F | F | F | regulation of osteoclast differentiation | TRFL\_BUBBU | TRFL\_HORSE | GO:0036417 | 0.000620732464307883 | 2/3222 | 2/552 | 0.0293072059118488 | 1 | F | F | F | F | tRNA destabilization | ANGI\_MOUSE | Q5WRG2\_RAT | GO:1901143 | 0.000620732464307883 | 2/3222 | 2/552 | 0.0293072059118488 | 1 | F | F | F | F | insulin catabolic process | CATD\_RAT | CEL2A\_PIG | GO:0009313 | 0.00465549348230913 | 15/3222 | 6/552 | 0.0307066113443708 | 1 | F | F | F | F | oligosaccharide catabolic process | AMY1\_ORYSJ | BGALA\_ASPNC | BGALA\_ASPOR | BGALA\_PENSQ | MANBA\_MOUSE | SIA\_ASPFU | GO:0010952 | 0.00248292985723153 | 8/3222 | 4/552 | 0.03347117307814 | 1 | F | F | F | F | positive regulation of peptidase activity | CATD\_RAT | CATH\_HUMAN | G3I1H5\_CRIGR | LGMN\_MOUSE | GO:0002573 | 0.00248292985723153 | 8/3222 | 4/552 | 0.03347117307814 | 1 | F | F | F | F | myeloid leukocyte differentiation | ANAG\_HUMAN | GBA1\_HUMAN | PPA5\_RAT | PRTN3\_HUMAN | GO:0002274 | 0.00248292985723153 | 8/3222 | 4/552 | 0.03347117307814 | 1 | F | F | F | F | myeloid leukocyte activation | ANAG\_HUMAN | DNAS1\_HUMAN | DNSL3\_HUMAN | PA2GX\_HUMAN | GO:0009268 | 0.00248292985723153 | 8/3222 | 4/552 | 0.03347117307814 | 1 | F | F | F | F | response to pH | G3I1H5\_CRIGR | GBA1\_HUMAN | HYAL1\_HUMAN | LGMN\_MOUSE | GO:0046352 | 0.00248292985723153 | 8/3222 | 4/552 | 0.03347117307814 | 1 | F | F | F | F | disaccharide catabolic process | AMY1\_ORYSJ | BGALA\_ASPNC | BGALA\_ASPOR | BGALA\_PENSQ | GO:0017000 | 0.00248292985723153 | 8/3222 | 4/552 | 0.03347117307814 | 1 | F | F | F | F | antibiotic biosynthetic process | MDLA\_PENCA | MDLA\_PENCY | Q7LST4\_PENEN | W6Q990\_PENRF | GO:0006516 | 0.00248292985723153 | 8/3222 | 4/552 | 0.03347117307814 | 1 | F | F | F | F | glycoprotein catabolic process | ANAG\_HUMAN | BGLR\_HUMAN | IDUA\_HUMAN | MANBA\_MOUSE | GO:0045019 | 0.00155183116076971 | 5/3222 | 3/552 | 0.038124911617623 | 1 | F | F | F | F | negative regulation of nitric oxide biosynthetic process | AGAL\_HUMAN | PPA5\_HUMAN | PPA5\_RAT | GO:1904406 | 0.00155183116076971 | 5/3222 | 3/552 | 0.038124911617623 | 1 | F | F | F | F | negative regulation of nitric oxide metabolic process | AGAL\_HUMAN | PPA5\_HUMAN | PPA5\_RAT | GO:0034755 | 0.00155183116076971 | 5/3222 | 3/552 | 0.038124911617623 | 1 | F | F | F | F | iron ion transmembrane transport | PPA5\_PIG | TRFL\_BUBBU | TRFL\_HORSE | GO:0031505 | 0.00155183116076971 | 5/3222 | 3/552 | 0.038124911617623 | 1 | F | F | F | F | fungal-type cell wall organization | CARP1\_CANAL | EXG1\_CANAL | EXG1\_YEAST | GO:0050795 | 0.00155183116076971 | 5/3222 | 3/552 | 0.038124911617623 | 1 | F | F | F | F | regulation of behavior | EST6\_DROME | PTGDS\_HUMAN | PTGDS\_MOUSE | GO:0006004 | 0.00155183116076971 | 5/3222 | 3/552 | 0.038124911617623 | 1 | F | F | F | F | fucose metabolic process | FUCO\_HUMAN | J9UN47\_GIBZA | OFUT1\_CAEEL | GO:0002521 | 0.0037243947858473 | 12/3222 | 5/552 | 0.0401646100816836 | 1 | F | F | F | F | leukocyte differentiation | ANAG\_HUMAN | GBA1\_HUMAN | LICH\_HUMAN | PPA5\_RAT | PRTN3\_HUMAN | GO:0002697 | 0.0074487895716946 | 24/3222 | 8/552 | 0.040422922599705 | 1 | F | F | F | F | regulation of immune effector process | DNAS1\_HUMAN | DNSL3\_HUMAN | GRASS\_DROME | KLK7\_HUMAN | KLK7\_MOUSE | PGPSA\_DROME | PPA5\_HUMAN | PPA5\_RAT | GO:0030149 | 0.00496585971446307 | 16/3222 | 6/552 | 0.0422232446308463 | 1 | F | F | F | F | sphingolipid catabolic process | AGAL\_HUMAN | ENPP2\_HUMAN | ENPP2\_RAT | GBA1\_HUMAN | PPT1\_HUMAN | SIA\_ASPFU | GO:0050714 | 0.00496585971446307 | 16/3222 | 6/552 | 0.0422232446308463 | 1 | F | F | F | F | positive regulation of protein secretion | ANG1\_BOVIN | ANG2\_MOUSE | ANG3\_MOUSE | ANG4\_MOUSE | ANGI\_MOUSE | Q5WRG2\_RAT | GO:0051603 | 0.0102420856610801 | 33/3222 | 10/552 | 0.0438959565687743 | 1 | F | F | F | F | proteolysis involved in protein catabolic process | ANAG\_HUMAN | CATH\_HUMAN | CHYM\_CAMDR | D6XHE1\_TRYB2 | G3I1H5\_CRIGR | GBA1\_HUMAN | GRAA\_HUMAN | GRAC\_MOUSE | LGMN\_MOUSE | MAN12\_PENCI | GO:0051241 | 0.0173805090006207 | 56/3222 | 15/552 | 0.0451197716383984 | 1 | F | F | F | F | negative regulation of multicellular organismal process | G3I1H5\_CRIGR | GBA1\_HUMAN | KLK8\_MOUSE | LGMN\_MOUSE | PA2GA\_HUMAN | PA2GE\_HUMAN | PA2GX\_HUMAN | PGH2\_HUMAN | PGRP1\_CAMDR | PPA5\_HUMAN | PPA5\_RAT | PTGDS\_HUMAN | PTGDS\_MOUSE | TRFL\_BUBBU | TRFL\_HORSE | GO:0031347 | 0.0220360024829299 | 71/3222 | 18/552 | 0.0495473414218998 | 1 | F | F | F | F | regulation of defense response | ANAG\_HUMAN | AOAH\_MOUSE | CARP1\_CANAL | DNAS1\_HUMAN | DNSL3\_HUMAN | GBA1\_HUMAN | GRASS\_DROME | KLK7\_HUMAN | KLK7\_MOUSE | PA2GA\_HUMAN | PA2GX\_HUMAN | PGH2\_HUMAN | PGPSA\_DROME | PPA5\_HUMAN | PPA5\_RAT | RIP0\_DIACA | RIP3\_MOMCH | RIPT\_TRIKI | GO:0002682 | 0.0251396648044693 | 81/3222 | 20/552 | 0.0511752418628746 | 1 | F | F | F | F | regulation of immune system process | ANAG\_HUMAN | CATH\_HUMAN | CFAD\_MOUSE | DNAS1\_HUMAN | DNSL3\_HUMAN | G3I1H5\_CRIGR | GRASS\_DROME | KLK7\_HUMAN | KLK7\_MOUSE | LGMN\_MOUSE | LICH\_HUMAN | PA21B\_PIG | PA2GA\_HUMAN | PA2GE\_HUMAN | PA2GX\_HUMAN | PGPSA\_DROME | PPA5\_HUMAN | PPA5\_RAT | TRFL\_BUBBU | TRFL\_HORSE | GO:0098660 | 0.00279329608938547 | 9/3222 | 4/552 | 0.0522586932107502 | 1 | F | F | F | F | inorganic ion transmembrane transport | CERU\_RAT | PPA5\_PIG | TRFL\_BUBBU | TRFL\_HORSE | GO:0098655 | 0.00279329608938547 | 9/3222 | 4/552 | 0.0522586932107502 | 1 | F | F | F | F | monoatomic cation transmembrane transport | CERU\_RAT | PPA5\_PIG | TRFL\_BUBBU | TRFL\_HORSE | GO:0098662 | 0.00279329608938547 | 9/3222 | 4/552 | 0.0522586932107502 | 1 | F | F | F | F | inorganic cation transmembrane transport | CERU\_RAT | PPA5\_PIG | TRFL\_BUBBU | TRFL\_HORSE | GO:0016999 | 0.00279329608938547 | 9/3222 | 4/552 | 0.0522586932107502 | 1 | F | F | F | F | antibiotic metabolic process | MDLA\_PENCA | MDLA\_PENCY | Q7LST4\_PENEN | W6Q990\_PENRF | GO:0003014 | 0.00279329608938547 | 9/3222 | 4/552 | 0.0522586932107502 | 1 | F | F | F | F | renal system process | ANG2\_MOUSE | G3I1H5\_CRIGR | LGMN\_MOUSE | RENI\_RAT | GO:0009887 | 0.00651769087523277 | 21/3222 | 7/552 | 0.0539380438794858 | 1 | F | F | F | F | animal organ morphogenesis | GBA1\_HUMAN | HYAL1\_HUMAN | LICH\_HUMAN | PPA5\_HUMAN | PPA5\_RAT | TRFL\_BUBBU | TRFL\_HORSE | GO:1904951 | 0.00651769087523277 | 21/3222 | 7/552 | 0.0539380438794858 | 1 | F | F | F | F | positive regulation of establishment of protein localization | ANG1\_BOVIN | ANG2\_MOUSE | ANG3\_MOUSE | ANG4\_MOUSE | ANGI\_MOUSE | PGH2\_HUMAN | Q5WRG2\_RAT | GO:0051222 | 0.00651769087523277 | 21/3222 | 7/552 | 0.0539380438794858 | 1 | F | F | F | F | positive regulation of protein transport | ANG1\_BOVIN | ANG2\_MOUSE | ANG3\_MOUSE | ANG4\_MOUSE | ANGI\_MOUSE | PGH2\_HUMAN | Q5WRG2\_RAT | GO:0010605 | 0.04252017380509 | 137/3222 | 31/552 | 0.055228406083298 | 1 | F | F | F | F | negative regulation of macromolecule metabolic process | ANG1\_BOVIN | ANG3\_MOUSE | ANGI\_MOUSE | E0CX04\_MOMBA | G3I1H5\_CRIGR | GBA1\_HUMAN | GRAA\_HUMAN | GRAC\_MOUSE | LGMN\_MOUSE | O04358\_IRIHO | PA2GX\_HUMAN | PGH2\_HUMAN | PGRP1\_CAMDR | PPA5\_HUMAN | PPA5\_RAT | Q2QEH4\_SAPOF | Q94BW3\_CINCA | RIP0\_DIACA | RIP1\_BRYDI | RIP1\_HORVU | RIP1\_MOMCH | RIP1\_PHYAM | RIP2\_PHYAM | RIP3\_MOMCH | RIPA\_PHYAM | RIPG\_SURMU | RIPL1\_PHYDI | RIPL2\_PHYDI | RIPT\_TRIKI | TRFL\_BUBBU | TRFL\_HORSE | GO:0007611 | 0.00527622594661701 | 17/3222 | 6/552 | 0.0561218413719036 | 1 | F | F | F | F | learning or memory | DOPO\_HUMAN | G3I1H5\_CRIGR | KLK8\_MOUSE | LGMN\_MOUSE | PGH2\_HUMAN | PPT1\_HUMAN | GO:0033674 | 0.00527622594661701 | 17/3222 | 6/552 | 0.0561218413719036 | 1 | F | F | F | F | positive regulation of kinase activity | ANG2\_MOUSE | ANG3\_MOUSE | ANG4\_MOUSE | ANGI\_MOUSE | PA21B\_PIG | Q5WRG2\_RAT | GO:0002700 | 0.00527622594661701 | 17/3222 | 6/552 | 0.0561218413719036 | 1 | F | F | F | F | regulation of production of molecular mediator of immune response | GRASS\_DROME | KLK7\_HUMAN | KLK7\_MOUSE | PGPSA\_DROME | PPA5\_HUMAN | PPA5\_RAT | GO:0009395 | 0.00527622594661701 | 17/3222 | 6/552 | 0.0561218413719036 | 1 | F | F | F | F | phospholipid catabolic process | ENPP2\_HUMAN | ENPP2\_RAT | LIPR2\_HUMAN | LIPR2\_RAT | PA2GX\_HUMAN | PAG15\_HUMAN | GO:0046503 | 0.00527622594661701 | 17/3222 | 6/552 | 0.0561218413719036 | 1 | F | F | F | F | glycerolipid catabolic process | ENPP2\_HUMAN | ENPP2\_RAT | LIPR2\_HUMAN | LIPR2\_RAT | PA2GX\_HUMAN | PAG15\_HUMAN | GO:0035296 | 0.00403476101800124 | 13/3222 | 5/552 | 0.0563393829012906 | 1 | F | F | F | F | regulation of tube diameter | DOPO\_HUMAN | NP1\_RHOPR | NP2\_RHOPR | NP4\_RHOPR | PGH2\_HUMAN | GO:0097746 | 0.00403476101800124 | 13/3222 | 5/552 | 0.0563393829012906 | 1 | F | F | F | F | blood vessel diameter maintenance | DOPO\_HUMAN | NP1\_RHOPR | NP2\_RHOPR | NP4\_RHOPR | PGH2\_HUMAN | GO:0019538 | 0.137181874612042 | 442/3222 | 88/552 | 0.0564923500624374 | 1 | F | F | F | F | protein metabolic process | A0A0R3QSA7\_9BILA | A0A3S5H5N2\_LEIDO | A0A6P6YAT6\_DERPT | A0NFU8\_ANOGA | A4GX63\_TOXGO | ANAG\_HUMAN | ANG2\_MOUSE | B4F320\_LIMPO | BGLR\_HUMAN | CARP1\_CANAL | CARP2\_CANAX | CARP\_RHIPU | CATD\_RAT | CATH\_HUMAN | CATLL\_FASHE | CBPA1\_PIG | CBPD\_LOPSP | CBPN\_HUMAN | CEL2A\_PIG | CFAD\_MOUSE | CHYM\_CAMDR | COGS\_HYPLI | CUCM1\_CUCME | CYP5\_CAEEL | CYSP\_BLOTA | D6XHE1\_TRYB2 | DDN1\_BOVIN | DPP2\_HUMAN | EGFB2\_MOUSE | ERVB\_TABDI | G3I1H5\_CRIGR | GBA1\_HUMAN | GRAA\_HUMAN | GRAC\_MOUSE | GRAK\_HUMAN | GRASS\_DROME | HE12\_DANRE | HS3S1\_MOUSE | IDUA\_HUMAN | J7LCB0\_DEIAC | KLK10\_HUMAN | KLK1\_HUMAN | KLK2\_HORSE | KLK2\_HUMAN | KLK7\_HUMAN | KLK7\_MOUSE | KLK8\_MOUSE | LAPA\_ASPOR | LGMN\_MOUSE | MAN12\_PENCI | MANBA\_MOUSE | MCPT2\_RAT | MMP1\_PIG | O81226\_CARPA | O97389\_HELAM | OFUT1\_CAEEL | PCP\_HUMAN | PEPA\_ASPPH | PGPSA\_DROME | PPAF1\_HOLDI | PPT1\_BOVIN | PPT1\_HUMAN | PRS57\_HUMAN | PRTN3\_HUMAN | Q06AK3\_TOXGO | Q5B038\_EMENI | Q69G21\_TENMO | Q6R7Z5\_9TRYP | Q7YXL2\_TENMO | QPCT1\_DROME | QPCT2\_DROME | QPCT\_IXOSC | QPCT\_MOUSE | RENI\_RAT | TPP1\_HUMAN | TRFL\_BUBBU | TRFL\_HORSE | TRY1\_GADMO | TRY3\_SALSA | TRYB2\_HUMAN | VM11\_BOTMO | VM12\_CROAD | VM1A3\_DEIAC | VM1BI\_BOTMO | VM1T1\_PROMU | VM1T2\_PROFL | VSPP\_DEIAC | VSPSX\_GLOSA | GO:0050678 | 0.00806952203600248 | 26/3222 | 8/552 | 0.0628860289856925 | 1 | F | F | F | F | regulation of epithelial cell proliferation | ANG1\_BOVIN | ANG2\_MOUSE | ANG3\_MOUSE | ANG4\_MOUSE | ANGI\_MOUSE | DOPO\_HUMAN | HYAL1\_HUMAN | Q5WRG2\_RAT | GO:0030335 | 0.00806952203600248 | 26/3222 | 8/552 | 0.0628860289856925 | 1 | F | F | F | F | positive regulation of cell migration | CATH\_HUMAN | ENPP2\_HUMAN | ENPP2\_RAT | G3I1H5\_CRIGR | HYAL1\_HUMAN | LGMN\_MOUSE | PGH2\_HUMAN | PPA5\_RAT | GO:0002831 | 0.010862818125388 | 35/3222 | 10/552 | 0.0633489772938727 | 1 | F | F | F | F | regulation of response to biotic stimulus | ANAG\_HUMAN | GRASS\_DROME | KLK7\_HUMAN | KLK7\_MOUSE | PGPSA\_DROME | RIP0\_DIACA | RIP3\_MOMCH | RIPT\_TRIKI | TRFL\_BUBBU | TRFL\_HORSE | GO:0016477 | 0.010862818125388 | 35/3222 | 10/552 | 0.0633489772938727 | 1 | F | F | F | F | cell migration | ANG2\_MOUSE | ANG3\_MOUSE | ANG4\_MOUSE | ANGI\_MOUSE | DOPO\_HUMAN | ENPP2\_RAT | GBA1\_HUMAN | PA21B\_PIG | PRTN3\_HUMAN | Q5WRG2\_RAT | GO:0030167 | 0.00186219739292365 | 6/3222 | 3/552 | 0.0666856095839806 | 1 | F | F | F | F | proteoglycan catabolic process | ANAG\_HUMAN | BGLR\_HUMAN | IDUA\_HUMAN | GO:0046479 | 0.00186219739292365 | 6/3222 | 3/552 | 0.0666856095839806 | 1 | F | F | F | F | glycosphingolipid catabolic process | AGAL\_HUMAN | GBA1\_HUMAN | SIA\_ASPFU | GO:0048771 | 0.00186219739292365 | 6/3222 | 3/552 | 0.0666856095839806 | 1 | F | F | F | F | tissue remodeling | CERU\_RAT | DOPO\_HUMAN | LICH\_HUMAN | GO:0030200 | 0.00186219739292365 | 6/3222 | 3/552 | 0.0666856095839806 | 1 | F | F | F | F | heparan sulfate proteoglycan catabolic process | ANAG\_HUMAN | BGLR\_HUMAN | IDUA\_HUMAN | GO:0051354 | 0.00186219739292365 | 6/3222 | 3/552 | 0.0666856095839806 | 1 | F | F | F | F | negative regulation of oxidoreductase activity | AGAL\_HUMAN | AOCX\_BOVIN | GRAA\_HUMAN | GO:0043085 | 0.0139664804469274 | 45/3222 | 12/552 | 0.0709129109495553 | 1 | F | F | F | F | positive regulation of catalytic activity | ANG1\_BOVIN | ANG2\_MOUSE | ANG3\_MOUSE | ANG4\_MOUSE | ANGI\_MOUSE | CATD\_RAT | CATH\_HUMAN | G3I1H5\_CRIGR | LGMN\_MOUSE | PA21B\_PIG | PRTN3\_HUMAN | Q5WRG2\_RAT | GO:0050890 | 0.00558659217877095 | 18/3222 | 6/552 | 0.0724572097673762 | 1 | F | F | F | F | cognition | DOPO\_HUMAN | G3I1H5\_CRIGR | KLK8\_MOUSE | LGMN\_MOUSE | PGH2\_HUMAN | PPT1\_HUMAN | GO:0034220 | 0.00310366232153942 | 10/3222 | 4/552 | 0.0756334556539759 | 1 | F | F | F | F | monoatomic ion transmembrane transport | CERU\_RAT | PPA5\_PIG | TRFL\_BUBBU | TRFL\_HORSE | GO:0035150 | 0.00434512725015518 | 14/3222 | 5/552 | 0.0757204357802997 | 1 | F | F | F | F | regulation of tube size | DOPO\_HUMAN | NP1\_RHOPR | NP2\_RHOPR | NP4\_RHOPR | PGH2\_HUMAN | GO:0006672 | 0.00434512725015518 | 14/3222 | 5/552 | 0.0757204357802997 | 1 | F | F | F | F | ceramide metabolic process | AGAL\_HUMAN | ANAG\_HUMAN | GBA1\_HUMAN | PAG15\_HUMAN | SIA\_ASPFU | GO:2000147 | 0.00837988826815642 | 27/3222 | 8/552 | 0.0765432999201377 | 1 | F | F | F | F | positive regulation of cell motility | CATH\_HUMAN | ENPP2\_HUMAN | ENPP2\_RAT | G3I1H5\_CRIGR | HYAL1\_HUMAN | LGMN\_MOUSE | PGH2\_HUMAN | PPA5\_RAT | GO:0040017 | 0.00837988826815642 | 27/3222 | 8/552 | 0.0765432999201377 | 1 | F | F | F | F | positive regulation of locomotion | CATH\_HUMAN | ENPP2\_HUMAN | ENPP2\_RAT | G3I1H5\_CRIGR | HYAL1\_HUMAN | LGMN\_MOUSE | PGH2\_HUMAN | PPA5\_RAT | GO:0030214 | 0.000931098696461825 | 3/3222 | 2/552 | 0.0779098393185133 | 1 | F | F | F | F | hyaluronan catabolic process | BGLR\_HUMAN | HYAL1\_HUMAN | GO:1904035 | 0.000931098696461825 | 3/3222 | 2/552 | 0.0779098393185133 | 1 | F | F | F | F | regulation of epithelial cell apoptotic process | PA21B\_BOVIN | PA21B\_PIG | GO:0006907 | 0.000931098696461825 | 3/3222 | 2/552 | 0.0779098393185133 | 1 | F | F | F | F | pinocytosis | PPT1\_BOVIN | PPT1\_HUMAN | GO:0014004 | 0.000931098696461825 | 3/3222 | 2/552 | 0.0779098393185133 | 1 | F | F | F | F | microglia differentiation | ANAG\_HUMAN | GBA1\_HUMAN | GO:1904645 | 0.000931098696461825 | 3/3222 | 2/552 | 0.0779098393185133 | 1 | F | F | F | F | response to amyloid-beta | G3I1H5\_CRIGR | LGMN\_MOUSE | GO:0010411 | 0.000931098696461825 | 3/3222 | 2/552 | 0.0779098393185133 | 1 | F | F | F | F | xyloglucan metabolic process | Q07524\_TROMA | XTH34\_POPPZ | GO:0043535 | 0.000931098696461825 | 3/3222 | 2/552 | 0.0779098393185133 | 1 | F | F | F | F | regulation of blood vessel endothelial cell migration | PCP\_HUMAN | PGH2\_HUMAN | GO:1900273 | 0.000931098696461825 | 3/3222 | 2/552 | 0.0779098393185133 | 1 | F | F | F | F | positive regulation of long-term synaptic potentiation | G3I1H5\_CRIGR | LGMN\_MOUSE | GO:1904646 | 0.000931098696461825 | 3/3222 | 2/552 | 0.0779098393185133 | 1 | F | F | F | F | cellular response to amyloid-beta | G3I1H5\_CRIGR | LGMN\_MOUSE | GO:1902106 | 0.000931098696461825 | 3/3222 | 2/552 | 0.0779098393185133 | 1 | F | F | F | F | negative regulation of leukocyte differentiation | TRFL\_BUBBU | TRFL\_HORSE | GO:0030212 | 0.000931098696461825 | 3/3222 | 2/552 | 0.0779098393185133 | 1 | F | F | F | F | hyaluronan metabolic process | BGLR\_HUMAN | HYAL1\_HUMAN | GO:0042749 | 0.000931098696461825 | 3/3222 | 2/552 | 0.0779098393185133 | 1 | F | F | F | F | regulation of circadian sleep/wake cycle | PTGDS\_HUMAN | PTGDS\_MOUSE | GO:0033688 | 0.000931098696461825 | 3/3222 | 2/552 | 0.0779098393185133 | 1 | F | F | F | F | regulation of osteoblast proliferation | TRFL\_BUBBU | TRFL\_HORSE | GO:0030501 | 0.000931098696461825 | 3/3222 | 2/552 | 0.0779098393185133 | 1 | F | F | F | F | positive regulation of bone mineralization | TRFL\_BUBBU | TRFL\_HORSE | GO:1902370 | 0.000931098696461825 | 3/3222 | 2/552 | 0.0779098393185133 | 1 | F | F | F | F | regulation of tRNA catabolic process | ANGI\_MOUSE | Q5WRG2\_RAT | GO:1904037 | 0.000931098696461825 | 3/3222 | 2/552 | 0.0779098393185133 | 1 | F | F | F | F | positive regulation of epithelial cell apoptotic process | PA21B\_BOVIN | PA21B\_PIG | GO:0070948 | 0.000931098696461825 | 3/3222 | 2/552 | 0.0779098393185133 | 1 | F | F | F | F | regulation of neutrophil mediated cytotoxicity | DNAS1\_HUMAN | DNSL3\_HUMAN | GO:0048548 | 0.000931098696461825 | 3/3222 | 2/552 | 0.0779098393185133 | 1 | F | F | F | F | regulation of pinocytosis | PPT1\_BOVIN | PPT1\_HUMAN | GO:0090025 | 0.000931098696461825 | 3/3222 | 2/552 | 0.0779098393185133 | 1 | F | F | F | F | regulation of monocyte chemotaxis | G3I1H5\_CRIGR | LGMN\_MOUSE | GO:1903326 | 0.000931098696461825 | 3/3222 | 2/552 | 0.0779098393185133 | 1 | F | F | F | F | regulation of tRNA metabolic process | ANGI\_MOUSE | Q5WRG2\_RAT | GO:0010743 | 0.000931098696461825 | 3/3222 | 2/552 | 0.0779098393185133 | 1 | F | F | F | F | regulation of macrophage derived foam cell differentiation | PA2GA\_HUMAN | PA2GX\_HUMAN | GO:0010744 | 0.000931098696461825 | 3/3222 | 2/552 | 0.0779098393185133 | 1 | F | F | F | F | positive regulation of macrophage derived foam cell differentiation | PA2GA\_HUMAN | PA2GX\_HUMAN | GO:0030204 | 0.000931098696461825 | 3/3222 | 2/552 | 0.0779098393185133 | 1 | F | F | F | F | chondroitin sulfate metabolic process | BGLR\_HUMAN | HYAL1\_HUMAN | GO:0030728 | 0.000931098696461825 | 3/3222 | 2/552 | 0.0779098393185133 | 1 | F | F | F | F | ovulation | EST6\_DROME | PGH2\_HUMAN | GO:1902743 | 0.000931098696461825 | 3/3222 | 2/552 | 0.0779098393185133 | 1 | F | F | F | F | regulation of lamellipodium organization | ENPP2\_HUMAN | ENPP2\_RAT | GO:0090026 | 0.000931098696461825 | 3/3222 | 2/552 | 0.0779098393185133 | 1 | F | F | F | F | positive regulation of monocyte chemotaxis | G3I1H5\_CRIGR | LGMN\_MOUSE | GO:0001910 | 0.000931098696461825 | 3/3222 | 2/552 | 0.0779098393185133 | 1 | F | F | F | F | regulation of leukocyte mediated cytotoxicity | DNAS1\_HUMAN | DNSL3\_HUMAN | GO:0034110 | 0.000931098696461825 | 3/3222 | 2/552 | 0.0779098393185133 | 1 | F | F | F | F | regulation of homotypic cell-cell adhesion | CEL2A\_PIG | OXLA\_BOTAT | GO:0045638 | 0.000931098696461825 | 3/3222 | 2/552 | 0.0779098393185133 | 1 | F | F | F | F | negative regulation of myeloid cell differentiation | TRFL\_BUBBU | TRFL\_HORSE | GO:0045778 | 0.000931098696461825 | 3/3222 | 2/552 | 0.0779098393185133 | 1 | F | F | F | F | positive regulation of ossification | TRFL\_BUBBU | TRFL\_HORSE | GO:2000394 | 0.000931098696461825 | 3/3222 | 2/552 | 0.0779098393185133 | 1 | F | F | F | F | positive regulation of lamellipodium morphogenesis | ENPP2\_HUMAN | ENPP2\_RAT | GO:0032418 | 0.000931098696461825 | 3/3222 | 2/552 | 0.0779098393185133 | 1 | F | F | F | F | lysosome localization | PTGDS\_HUMAN | PTGDS\_MOUSE | GO:1903707 | 0.000931098696461825 | 3/3222 | 2/552 | 0.0779098393185133 | 1 | F | F | F | F | negative regulation of hemopoiesis | TRFL\_BUBBU | TRFL\_HORSE | GO:1990849 | 0.000931098696461825 | 3/3222 | 2/552 | 0.0779098393185133 | 1 | F | F | F | F | vacuolar localization | PTGDS\_HUMAN | PTGDS\_MOUSE | GO:0070169 | 0.000931098696461825 | 3/3222 | 2/552 | 0.0779098393185133 | 1 | F | F | F | F | positive regulation of biomineral tissue development | TRFL\_BUBBU | TRFL\_HORSE | GO:0036415 | 0.000931098696461825 | 3/3222 | 2/552 | 0.0779098393185133 | 1 | F | F | F | F | regulation of tRNA stability | ANGI\_MOUSE | Q5WRG2\_RAT | GO:0070269 | 0.000931098696461825 | 3/3222 | 2/552 | 0.0779098393185133 | 1 | F | F | F | F | pyroptosis | GRAA\_HUMAN | GRAC\_MOUSE | GO:2000392 | 0.000931098696461825 | 3/3222 | 2/552 | 0.0779098393185133 | 1 | F | F | F | F | regulation of lamellipodium morphogenesis | ENPP2\_HUMAN | ENPP2\_RAT | GO:0031069 | 0.000931098696461825 | 3/3222 | 2/552 | 0.0779098393185133 | 1 | F | F | F | F | hair follicle morphogenesis | ANAG\_HUMAN | PA2GX\_HUMAN | GO:0090330 | 0.000931098696461825 | 3/3222 | 2/552 | 0.0779098393185133 | 1 | F | F | F | F | regulation of platelet aggregation | CEL2A\_PIG | OXLA\_BOTAT | GO:0045669 | 0.000931098696461825 | 3/3222 | 2/552 | 0.0779098393185133 | 1 | F | F | F | F | positive regulation of osteoblast differentiation | TRFL\_BUBBU | TRFL\_HORSE | GO:0002701 | 0.000931098696461825 | 3/3222 | 2/552 | 0.0779098393185133 | 1 | F | F | F | F | negative regulation of production of molecular mediator of immune response | PPA5\_HUMAN | PPA5\_RAT | GO:0023061 | 0.000931098696461825 | 3/3222 | 2/552 | 0.0779098393185133 | 1 | F | F | F | F | signal release | PGH2\_HUMAN | PPT1\_HUMAN | GO:0045637 | 0.000931098696461825 | 3/3222 | 2/552 | 0.0779098393185133 | 1 | F | F | F | F | regulation of myeloid cell differentiation | TRFL\_BUBBU | TRFL\_HORSE | GO:0045187 | 0.000931098696461825 | 3/3222 | 2/552 | 0.0779098393185133 | 1 | F | F | F | F | regulation of circadian sleep/wake cycle, sleep | PTGDS\_HUMAN | PTGDS\_MOUSE | GO:0002719 | 0.000931098696461825 | 3/3222 | 2/552 | 0.0779098393185133 | 1 | F | F | F | F | negative regulation of cytokine production involved in immune response | PPA5\_HUMAN | PPA5\_RAT | GO:0030225 | 0.000931098696461825 | 3/3222 | 2/552 | 0.0779098393185133 | 1 | F | F | F | F | macrophage differentiation | ANAG\_HUMAN | GBA1\_HUMAN | GO:1902745 | 0.000931098696461825 | 3/3222 | 2/552 | 0.0779098393185133 | 1 | F | F | F | F | positive regulation of lamellipodium organization | ENPP2\_HUMAN | ENPP2\_RAT | GO:0044255 | 0.132836747361887 | 428/3222 | 84/552 | 0.0819866094416525 | 1 | F | F | F | F | cellular lipid metabolic process | A0A1L8D5Z7\_BOTAT | AGAL\_HUMAN | ANAG\_HUMAN | ANG1\_BOVIN | ANG2\_MOUSE | ANG3\_MOUSE | ANG4\_MOUSE | ANGI\_MOUSE | AOAH\_MOUSE | ASAH1\_BALAS | ENPP2\_HUMAN | ENPP2\_RAT | FUCO\_HUMAN | GBA1\_HUMAN | I1SB18\_VIPAE | LICH\_HUMAN | LIPG\_HUMAN | LIPR2\_HUMAN | LIPR2\_RAT | MNLOX\_MAGO7 | NAGAB\_HUMAN | PA21B\_BOVIN | PA21B\_PIG | PA2A1\_BUNCE | PA2A1\_ECHCA | PA2A1\_NAJAT | PA2A1\_OPHHA | PA2A2\_NAJNA | PA2A2\_OPHHA | PA2A2\_TROCA | PA2A4\_NAJSG | PA2A5\_TRIST | PA2A7\_GLOHA | PA2A\_BOTJR | PA2A\_CROAT | PA2A\_DEIAC | PA2A\_GLOHA | PA2A\_NAJAT | PA2B1\_AGKPI | PA2B2\_BOTJR | PA2B2\_PROFL | PA2B3\_BOTAS | PA2B3\_BUNCE | PA2B5\_BUNCE | PA2B5\_NOTSC | PA2BA\_VIPAA | PA2BB\_GLOHA | PA2BB\_PSEAU | PA2BC\_VIPAA | PA2BD\_CRODU | PA2B\_BUNCE | PA2B\_NOTSC | PA2GA\_HUMAN | PA2GE\_HUMAN | PA2GX\_HUMAN | PA2H1\_AGKCL | PA2H1\_BOTBZ | PA2H1\_BOTJR | PA2H1\_BOTMO | PA2H1\_BOTPI | PA2H2\_BOTAS | PA2H2\_BOTMO | PA2H2\_BOTPI | PA2H2\_CERGO | PA2H3\_BOTPI | PA2HB\_AGKPI | PA2HB\_OXYSC | PA2HH\_TRIST | PA2HS\_ECHCA | PA2H\_BOTPA | PA2H\_DEIAC | PA2H\_PROMB | PA2N\_GLOHA | PA2\_APIME | PAG15\_HUMAN | PGH2\_HUMAN | PLA22\_ORYSJ | PPT1\_HUMAN | PTGDS\_HUMAN | PTGDS\_MOUSE | Q5WRG2\_RAT | SIA\_ASPFU | THCAS\_CANSA | TTHY\_CHICK | GO:0032103 | 0.00993171942892613 | 32/3222 | 9/552 | 0.0829267752355521 | 1 | F | F | F | F | positive regulation of response to external stimulus | ANAG\_HUMAN | G3I1H5\_CRIGR | GRASS\_DROME | KLK7\_HUMAN | KLK7\_MOUSE | LGMN\_MOUSE | PA2GA\_HUMAN | PGH2\_HUMAN | PGPSA\_DROME | GO:0042127 | 0.0344506517690875 | 111/3222 | 25/552 | 0.0831136708610738 | 1 | F | F | F | F | regulation of cell population proliferation | ANG1\_BOVIN | ANG2\_MOUSE | ANG3\_MOUSE | ANG4\_MOUSE | ANGI\_MOUSE | CATH\_HUMAN | DOPO\_HUMAN | ENPP2\_HUMAN | ENPP2\_RAT | G3I1H5\_CRIGR | GILT\_MOUSE | HYAL1\_HUMAN | KLK8\_MOUSE | LGMN\_MOUSE | PA21B\_BOVIN | PA21B\_PIG | PA2GA\_HUMAN | PA2GE\_HUMAN | PGH2\_HUMAN | PRTN3\_HUMAN | PTGDS\_HUMAN | PTGDS\_MOUSE | Q5WRG2\_RAT | TRFL\_BUBBU | TRFL\_HORSE | GO:0009698 | 0.0173805090006207 | 56/3222 | 14/552 | 0.0854087245036162 | 1 | F | F | F | F | phenylpropanoid metabolic process | DIR\_GLYEC | GCE2\_MYCTT | GCE\_CERUI | GCE\_HYPJQ | LAC1\_MELAO | LAC1\_TRAMX | LAC2\_TRAVE | LIG2\_PHACH | LIG4\_PHACH | LIG8\_PHACH | PEM1\_PHACH | Q60FD2\_9APHY | VPL1\_PLEER | VPL2\_PLEER | GO:0006650 | 0.0114835505896958 | 37/3222 | 10/552 | 0.0876244363910374 | 1 | F | F | F | F | glycerophospholipid metabolic process | ENPP2\_HUMAN | ENPP2\_RAT | LIPR2\_HUMAN | LIPR2\_RAT | PA21B\_BOVIN | PA21B\_PIG | PA2GA\_HUMAN | PA2GE\_HUMAN | PA2GX\_HUMAN | PAG15\_HUMAN | GO:0048870 | 0.0130353817504655 | 42/3222 | 11/552 | 0.091051088195356 | 1 | F | F | F | F | cell motility | ANG2\_MOUSE | ANG3\_MOUSE | ANG4\_MOUSE | ANGI\_MOUSE | DOPO\_HUMAN | ENPP2\_HUMAN | ENPP2\_RAT | GBA1\_HUMAN | PA21B\_PIG | PRTN3\_HUMAN | Q5WRG2\_RAT | GO:0050778 | 0.00869025450031037 | 28/3222 | 8/552 | 0.0918413669260255 | 1 | F | F | F | F | positive regulation of immune response | ANAG\_HUMAN | CFAD\_MOUSE | GRASS\_DROME | KLK7\_HUMAN | KLK7\_MOUSE | LICH\_HUMAN | PA21B\_PIG | PGPSA\_DROME | GO:0008285 | 0.016139044072005 | 52/3222 | 13/552 | 0.0951986128776178 | 1 | F | F | F | F | negative regulation of cell population proliferation | ANG1\_BOVIN | ANG2\_MOUSE | ANG3\_MOUSE | ANG4\_MOUSE | ANGI\_MOUSE | GILT\_MOUSE | KLK8\_MOUSE | PA2GA\_HUMAN | PA2GE\_HUMAN | PGH2\_HUMAN | PTGDS\_HUMAN | PTGDS\_MOUSE | Q5WRG2\_RAT | GO:0031324 | 0.0446927374301676 | 144/3222 | 31/552 | 0.0959475629063536 | 1 | F | F | F | F | negative regulation of cellular metabolic process | AGAL\_HUMAN | ANG1\_BOVIN | ANG3\_MOUSE | ANGI\_MOUSE | E0CX04\_MOMBA | G3I1H5\_CRIGR | GBA1\_HUMAN | GRAA\_HUMAN | GRAC\_MOUSE | LGMN\_MOUSE | O04358\_IRIHO | PA2GX\_HUMAN | PGRP1\_CAMDR | PPA5\_HUMAN | PPA5\_RAT | Q2QEH4\_SAPOF | Q94BW3\_CINCA | RIP0\_DIACA | RIP1\_BRYDI | RIP1\_HORVU | RIP1\_MOMCH | RIP1\_PHYAM | RIP2\_PHYAM | RIP3\_MOMCH | RIPA\_PHYAM | RIPG\_SURMU | RIPL1\_PHYDI | RIPL2\_PHYDI | RIPT\_TRIKI | TRFL\_BUBBU | TRFL\_HORSE | GO:0050790 | 0.0238981998758535 | 77/3222 | 18/552 | 0.0967799413903943 | 1 | F | F | F | F | regulation of catalytic activity | AGAL\_HUMAN | ANG1\_BOVIN | ANG2\_MOUSE | ANG3\_MOUSE | ANG4\_MOUSE | ANGI\_MOUSE | AOCX\_BOVIN | CATD\_RAT | CATH\_HUMAN | G3I1H5\_CRIGR | GBA1\_HUMAN | GRAA\_HUMAN | LGMN\_MOUSE | PA21B\_PIG | PGH2\_HUMAN | PPT1\_HUMAN | PRTN3\_HUMAN | Q5WRG2\_RAT | GO:0003018 | 0.00465549348230913 | 15/3222 | 5/552 | 0.0982273479982074 | 1 | F | F | F | F | vascular process in circulatory system | DOPO\_HUMAN | NP1\_RHOPR | NP2\_RHOPR | NP4\_RHOPR | PGH2\_HUMAN | GO:0043524 | 0.00465549348230913 | 15/3222 | 5/552 | 0.0982273479982074 | 1 | F | F | F | F | negative regulation of neuron apoptotic process | G3I1H5\_CRIGR | GBA1\_HUMAN | LGMN\_MOUSE | PPT1\_BOVIN | PPT1\_HUMAN | GO:1903829 | 0.0074487895716946 | 24/3222 | 7/552 | 0.101657576680607 | 1 | F | F | F | F | positive regulation of protein localization | ANG1\_BOVIN | ANG2\_MOUSE | ANG3\_MOUSE | ANG4\_MOUSE | ANGI\_MOUSE | PGH2\_HUMAN | Q5WRG2\_RAT | GO:0031644 | 0.00217256362507759 | 7/3222 | 3/552 | 0.102213306168067 | 1 | F | F | F | F | regulation of nervous system process | GBA1\_HUMAN | KLK8\_MOUSE | PPAP\_RAT | GO:2001056 | 0.00217256362507759 | 7/3222 | 3/552 | 0.102213306168067 | 1 | F | F | F | F | positive regulation of cysteine-type endopeptidase activity | CATD\_RAT | G3I1H5\_CRIGR | LGMN\_MOUSE | GO:0051258 | 0.00217256362507759 | 7/3222 | 3/552 | 0.102213306168067 | 1 | F | F | F | F | protein polymerization | ANG1\_BOVIN | ANGI\_MOUSE | Q5WRG2\_RAT | GO:0048469 | 0.00217256362507759 | 7/3222 | 3/552 | 0.102213306168067 | 1 | F | F | F | F | cell maturation | GBA1\_HUMAN | PA2GX\_HUMAN | RENI\_RAT | GO:0010950 | 0.00217256362507759 | 7/3222 | 3/552 | 0.102213306168067 | 1 | F | F | F | F | positive regulation of endopeptidase activity | CATD\_RAT | G3I1H5\_CRIGR | LGMN\_MOUSE | GO:0042311 | 0.00217256362507759 | 7/3222 | 3/552 | 0.102213306168067 | 1 | F | F | F | F | vasodilation | NP1\_RHOPR | NP2\_RHOPR | NP4\_RHOPR | GO:0010467 | 0.00341402855369336 | 11/3222 | 4/552 | 0.103328868902917 | 1 | F | F | F | F | gene expression | ANG2\_MOUSE | LICH\_HUMAN | PTGDS\_HUMAN | PTGDS\_MOUSE | GO:0080164 | 0.00341402855369336 | 11/3222 | 4/552 | 0.103328868902917 | 1 | F | F | F | F | regulation of nitric oxide metabolic process | AGAL\_HUMAN | PGH2\_HUMAN | PPA5\_HUMAN | PPA5\_RAT | GO:1903555 | 0.00341402855369336 | 11/3222 | 4/552 | 0.103328868902917 | 1 | F | F | F | F | regulation of tumor necrosis factor superfamily cytokine production | PPA5\_HUMAN | PPA5\_RAT | TRFL\_BUBBU | TRFL\_HORSE | GO:0032680 | 0.00341402855369336 | 11/3222 | 4/552 | 0.103328868902917 | 1 | F | F | F | F | regulation of tumor necrosis factor production | PPA5\_HUMAN | PPA5\_RAT | TRFL\_BUBBU | TRFL\_HORSE | GO:0050900 | 0.00341402855369336 | 11/3222 | 4/552 | 0.103328868902917 | 1 | F | F | F | F | leukocyte migration | DOPO\_HUMAN | GBA1\_HUMAN | PA21B\_PIG | PRTN3\_HUMAN | GO:1903510 | 0.00341402855369336 | 11/3222 | 4/552 | 0.103328868902917 | 1 | F | F | F | F | mucopolysaccharide metabolic process | ANAG\_HUMAN | BGLR\_HUMAN | HYAL1\_HUMAN | IDUA\_HUMAN | GO:0010594 | 0.00341402855369336 | 11/3222 | 4/552 | 0.103328868902917 | 1 | F | F | F | F | regulation of endothelial cell migration | G3I1H5\_CRIGR | LGMN\_MOUSE | PCP\_HUMAN | PGH2\_HUMAN | GO:0045428 | 0.00341402855369336 | 11/3222 | 4/552 | 0.103328868902917 | 1 | F | F | F | F | regulation of nitric oxide biosynthetic process | AGAL\_HUMAN | PGH2\_HUMAN | PPA5\_HUMAN | PPA5\_RAT | GO:0006687 | 0.00341402855369336 | 11/3222 | 4/552 | 0.103328868902917 | 1 | F | F | F | F | glycosphingolipid metabolic process | AGAL\_HUMAN | ANAG\_HUMAN | GBA1\_HUMAN | SIA\_ASPFU | GO:0006029 | 0.00341402855369336 | 11/3222 | 4/552 | 0.103328868902917 | 1 | F | F | F | F | proteoglycan metabolic process | ANAG\_HUMAN | BGLR\_HUMAN | HS3S1\_MOUSE | IDUA\_HUMAN | GO:0051172 | 0.037243947858473 | 120/3222 | 26/552 | 0.11310766546505 | 1 | F | F | F | F | negative regulation of nitrogen compound metabolic process | AGAL\_HUMAN | ANG1\_BOVIN | ANG3\_MOUSE | ANGI\_MOUSE | E0CX04\_MOMBA | GBA1\_HUMAN | GRAA\_HUMAN | GRAC\_MOUSE | O04358\_IRIHO | PGH2\_HUMAN | PPA5\_HUMAN | PPA5\_RAT | Q2QEH4\_SAPOF | Q94BW3\_CINCA | RIP0\_DIACA | RIP1\_BRYDI | RIP1\_HORVU | RIP1\_MOMCH | RIP1\_PHYAM | RIP2\_PHYAM | RIP3\_MOMCH | RIPA\_PHYAM | RIPG\_SURMU | RIPL1\_PHYDI | RIPL2\_PHYDI | RIPT\_TRIKI | GO:0070201 | 0.010552451893234 | 34/3222 | 9/552 | 0.113593873272085 | 1 | F | F | F | F | regulation of establishment of protein localization | ANG1\_BOVIN | ANG2\_MOUSE | ANG3\_MOUSE | ANG4\_MOUSE | ANGI\_MOUSE | CATD\_RAT | CEL2A\_PIG | PGH2\_HUMAN | Q5WRG2\_RAT | GO:0008152 | 0.921477343265053 | 2969/3222 | 516/552 | 0.115580583254501 | 1 | F | F | F | F | metabolic process | A0A059U759\_9PEZI | A0A068FT77\_9PEZI | A0A075B5H6\_TRIHA | A0A075C6T6\_RHIMI | A0A086SY89\_ACRC1 | A0A086T6R4\_ACRC1 | A0A087WNH2\_FICBE | A0A088T0J9\_GEOCN | A0A098DND1\_GIBZE | A0A0A0Y4H8\_TRAFO | A0A0J5Q413\_ASPFM | A0A0M3KKZ6\_RHIMI | A0A0M3KKZ8\_RHIMI | A0A0R3QSA7\_9BILA | A0A0R4I979\_BRABE | A0A0S2GKZ1\_9APHY | A0A173N065\_EISFE | A0A1L6CE30\_9EURO | A0A1L8D5Z7\_BOTAT | A0A1L9WG58\_ASPA1 | A0A1S4NYF8\_PANVG | A0A1S9DRB1\_ASPOZ | A0A2H5BN17\_TALPI | A0A2N1LTK3\_TRIHA | A0A2U8ZTY7\_RHIZD | A0A2Z4HIN9\_9EURO | A0A384E148\_NICBE | A0A3B6UEQ2\_RHIMI | A0A3B6UEQ6\_EISFE | A0A3G2C3I4\_9EURO | A0A3G4RHU4\_9PEZI | A0A3L6SKP5\_PANMI | A0A3S5H5N2\_LEIDO | A0A482LWB1\_OSTFU | A0A5J6BJN2\_MALCI | A0A6F8Z6Y2\_BOMMO | A0A6M9BP13\_9EURO | A0A6P6YAT6\_DERPT | A0A7S6G7I6\_9PEZI | A0A856TAI5\_9BASI | A0NFU8\_ANOGA | A1E266\_9PEZI | A1HA\_LOXIN | A1HB2\_LOXIN | A2QZC8\_ASPNC | A2TM14\_HEVBR | A311\_LOXLA | A4GX63\_TOXGO | A5AB48\_ASPNC | A6PZ97\_SALSA | A6YRT4\_9PEZI | A7KMF0\_9CAEN | A8NI40\_COPC7 | A8PUY1\_MALGO | A8PUY5\_MALGO | A9LI60\_BIOOC | A9ZSX9\_9BRYO | ABFB\_ASPKW | ADA2\_HUMAN | ADPG2\_ARATH | AGAL\_HUMAN | AGAL\_ORYSJ | AMY1A\_HUMAN | AMY1\_HORVU | AMY1\_ORYSJ | AMYA1\_ASPOR | AMYG\_SACFI | AMY\_ORYLA | ANAG\_HUMAN | ANG1\_BOVIN | ANG2\_MOUSE | ANG3\_MOUSE | ANG4\_MOUSE | ANGI\_MOUSE | AOAH\_MOUSE | AOC1\_HUMAN | AOCX\_BOVIN | APO1\_CYCAE | ASAH1\_BALAS | ASM3A\_HUMAN | ASM3A\_MOUSE | AXE1\_ASPAW | AXE2\_TALPU | AXHA2\_EMENI | B2ZGS7\_9ASPA | B4F320\_LIMPO | B7X9Z0\_COPCI | B7X9Z2\_COPCI | B9TU22\_GADMO | BGALA\_ASPNC | BGALA\_ASPOR | BGALA\_PENSQ | BGL1\_ASPAC | BGLA\_ASPFU | BGLA\_ASPOR | BGLR\_HUMAN | C3VEV9\_PENCN | C7YSL3\_FUSV7 | CARP1\_CANAL | CARP2\_CANAX | CARP\_RHIPU | CAT3\_NEUCR | CATD\_RAT | CATH\_HUMAN | CATLL\_FASHE | CBHB\_ASPFU | CBHRE\_GEOS1 | CBPA1\_PIG | CBPD\_LOPSP | CBPN\_HUMAN | CDA\_COLLN | CDA\_EMENI | CEL2A\_PIG | CFAD\_MOUSE | CHI1\_COCPS | CHI2\_HORVU | CHI2\_ORYSJ | CHI33\_TRIHA | CHI42\_TRIHA | CHI4\_CRYJA | CHIA\_HUMAN | CHIC\_ARATH | CHIC\_SECCE | CHIL3\_MOUSE | CHIT\_PUNGR | CHLY\_HEVBR | CHYM\_CAMDR | CKX1\_MAIZE | COGS\_HYPLI | CONB\_CANEN | CUCM1\_CUCME | CUTI1\_ASPOR | CYP5\_CAEEL | CYSP\_BLOTA | D0QF43\_9HELO | D1M8S7\_HEVBR | D1MPT2\_ROYRE | D6XHE1\_TRYB2 | D9MWI4\_9ASPA | DDN1\_BOVIN | DEXT\_TALMI | DIR\_GLYEC | DNAS1\_HUMAN | DNSL3\_HUMAN | DOPO\_HUMAN | DPP2\_HUMAN | E0A7J0\_YARLL | E0XN39\_9EURO | E13B\_HORVU | E13C\_MUSAC | E3VTL0\_9ASPA | E5D0X5\_SCHOC | E9G5J5\_DAPPU | ECP\_HUMAN | EGFB2\_MOUSE | EGLB\_ASPNG | ENDO2\_ARATH | ENG1\_RHIMI | ENPP2\_HUMAN | ENPP2\_RAT | ERVB\_TABDI | EST6\_DROME | EXG1\_CANAL | EXG1\_YEAST | F0ZJZ1\_DICPU | F1CYZ0\_TALFU | F2Z7L1\_9ANNE | FAEA\_ASPNG | FAEB1\_ASPOR | FAEB2\_ASPOR | FUCO\_HUMAN | G0RVK1\_HYPJQ | G2Q665\_MYCTT | G2QVH2\_THETT | G2X3Y1\_VERDV | G3I1H5\_CRIGR | G3JPF7\_CORMM | G3YAL0\_ASPNA | G3YFQ1\_ASPNA | G8GLP2\_LENED | G9NTY1\_HYPAI | GANA\_ASPAC | GANA\_EMENI | GANA\_HUMIN | GBA1\_HUMAN | GCE2\_MYCTT | GCE\_CERUI | GCE\_HYPJQ | GGH\_HUMAN | GH7B\_LIMQU | GOOX\_SARSR | GPX3\_HUMAN | GPX5\_HUMAN | GRAA\_HUMAN | GRAC\_MOUSE | GRAK\_HUMAN | GRASS\_DROME | GUB2\_HORVU | GUN2\_HYPJE | GUN6\_HUMIN | GUN7\_HYPJQ | GUNC\_FUSOX | GUN\_ASPAC | GUN\_CRYAT | GUN\_MYTED | GUX1\_HUMGT | GUX1\_HYPJE | GUX1\_TRIHA | GUX2\_HYPJE | H1AE14\_PHACH | HE12\_DANRE | HEXC\_OSTFU | HS3S1\_MOUSE | HYAL1\_HUMAN | I1SB18\_VIPAE | I2FI81\_EISFE | I3RY46\_TRIHA | IDH\_OSTTA | IDUA\_HUMAN | INU2\_ASPFI | INUE\_ASPAW | INV\_SCHOC | IPUA\_ASPNG | J7LCB0\_DEIAC | J9UN47\_GIBZA | K7CID1\_PANTR | K7N5L9\_RAPSA | K9L8F3\_MALCI | KATG2\_MAGO7 | KLK10\_HUMAN | KLK1\_HUMAN | KLK2\_HORSE | KLK2\_HUMAN | KLK7\_HUMAN | KLK7\_MOUSE | KLK8\_MOUSE | L7SVX1\_RHIMI | LAC1\_MELAO | LAC1\_TRAMX | LAC2\_TRAVE | LALBA\_BOVIN | LALBA\_CAPHI | LALBA\_CAVPO | LALBA\_PAPCY | LAPA\_ASPOR | LGMN\_MOUSE | LICH\_HUMAN | LIG2\_PHACH | LIG4\_PHACH | LIG8\_PHACH | LIP1\_DIURU | LIP2\_DIURU | LIP2\_GEOCN | LIP3\_DIURU | LIPA\_MOEAP | LIPB\_PSEA2 | LIPG\_CANLF | LIPG\_HUMAN | LIPP\_HORSE | LIPR1\_CANLF | LIPR1\_HUMAN | LIPR2\_HUMAN | LIPR2\_RAT | LIP\_THELA | LUCI\_OPLGR | LYG\_STRCA | LYS1\_MUSDO | LYSC1\_ANAPL | LYSC1\_CANLF | LYSC1\_HORSE | LYSC2\_BOVIN | LYSC2\_ONCMY | LYSC\_COTJA | LYSC\_EQUAS | LYSC\_NUMME | LYSC\_OPIHO | LYSC\_PELSI | LYS\_BOMMO | LYS\_RUDPH | M2RAI8\_CERS8 | M9TI89\_RHIPU | MAN12\_PENCI | MAN4\_SOLLC | MANA\_ASPNC | MANA\_CANEN | MANA\_CRYAT | MANA\_HYPJR | MANA\_MYTED | MANA\_PODAN | MANBA\_MOUSE | MCPT2\_RAT | MDLA\_PENCA | MDLA\_PENCY | MEL1\_YEASX | MMP1\_PIG | MNCO\_MICNN | MNLOX\_MAGO7 | NAGAB\_CHICK | NAGAB\_HUMAN | NANL\_MACDE | NCS\_THLFG | NUP1\_PENCI | NUS1\_ASPOR | O00095\_HYPJE | O22443\_SOYBN | O44049\_TRYRA | O74705\_ASPNG | O77044\_9NEOP | O81100\_SOLLC | O81226\_CARPA | O81934\_CANEN | O97389\_HELAM | OFUT1\_CAEEL | P78583\_ASPOZ | P79074\_9AGAR | PA1\_VESBA | PA21B\_BOVIN | PA21B\_PIG | PA2A1\_BUNCE | PA2A1\_ECHCA | PA2A1\_NAJAT | PA2A1\_OPHHA | PA2A2\_NAJNA | PA2A2\_OPHHA | PA2A2\_TROCA | PA2A4\_NAJSG | PA2A5\_TRIST | PA2A7\_GLOHA | PA2A\_BOTJR | PA2A\_CROAT | PA2A\_DEIAC | PA2A\_GLOHA | PA2A\_NAJAT | PA2B1\_AGKPI | PA2B2\_BOTJR | PA2B2\_PROFL | PA2B3\_BOTAS | PA2B3\_BUNCE | PA2B5\_BUNCE | PA2B5\_NOTSC | PA2BA\_VIPAA | PA2BB\_GLOHA | PA2BB\_PSEAU | PA2BC\_VIPAA | PA2BD\_CRODU | PA2B\_BUNCE | PA2B\_NOTSC | PA2GA\_HUMAN | PA2GE\_HUMAN | PA2GX\_HUMAN | PA2H1\_AGKCL | PA2H1\_BOTBZ | PA2H1\_BOTJR | PA2H1\_BOTMO | PA2H1\_BOTPI | PA2H2\_BOTAS | PA2H2\_BOTMO | PA2H2\_BOTPI | PA2H2\_CERGO | PA2H3\_BOTPI | PA2HB\_AGKPI | PA2HB\_OXYSC | PA2HH\_TRIST | PA2HS\_ECHCA | PA2H\_BOTPA | PA2H\_DEIAC | PA2H\_PROMB | PA2N\_GLOHA | PA2\_APIME | PAG15\_HUMAN | PCP\_HUMAN | PDH1\_LEUMG | PELA\_ASPNG | PELB\_ASPNG | PEM1\_PHACH | PEPA\_ASPPH | PER1A\_ARMRU | PER1\_ARAHY | PER1\_SORBI | PER53\_ARATH | PER59\_ARATH | PERL\_BOVIN | PERL\_BUBBU | PERL\_CAPHI | PER\_ARTRA | PER\_COPCI | PGH2\_HUMAN | PGLR1\_ASPAC | PGLR1\_ASPNG | PGLR\_GIBFU | PGPSA\_DROME | PGRP1\_CAMDR | PHAZ\_TALFU | PLA22\_ORYSJ | PLY1\_JUNAS | PME\_DAUCA | PME\_SITOR | POXA\_DICDI | PPA5\_HUMAN | PPA5\_RAT | PPAF1\_HOLDI | PPAP\_RAT | PPT1\_BOVIN | PPT1\_HUMAN | PPT2\_HUMAN | PRS57\_HUMAN | PRTN3\_HUMAN | PTGDS\_HUMAN | PTGDS\_MOUSE | Q02321\_PHACH | Q06AK3\_TOXGO | Q07524\_TROMA | Q0KFV0\_SOLLC | Q12715\_HYPJE | Q2U8V9\_ASPOR | Q2Z1W1\_PHACH | Q40069\_HORVU | Q43576\_TOBAC | Q4AE59\_OSTFU | Q4W6L6\_CYCRE | Q4WP32\_ASPFU | Q50KB2\_PHACH | Q55FE6\_DICDI | Q588B8\_CRYJA | Q5B038\_EMENI | Q5WRG2\_RAT | Q60FD2\_9APHY | Q69G21\_TENMO | Q6NY42\_DANRE | Q6R7Z5\_9TRYP | Q6VAY1\_9PEZI | Q6WER3\_GIBZA | Q6WSR8\_PICAB | Q70C53\_SOLTU | Q70SY0\_HYPJE | Q7LHI2\_PHACH | Q7LIJ0\_PHACH | Q7LST4\_PENEN | Q7RWP2\_NEUCR | Q7X9A9\_CAMSI | Q7YXL2\_TENMO | Q86RS6\_MANSE | Q8H0C9\_VIGUN | Q8J0K6\_MELAO | Q8J0K8\_MELAO | Q8NJY6\_9HYPO | Q8T0W7\_9NEOP | Q8TFL9\_TALEM | Q8TG26\_THEAU | Q8TGI8\_TALEM | Q92456\_HYPJE | Q92458\_HYPJE | Q93X60\_CICIN | Q95KP4\_HORSE | Q95V66\_PENVA | Q96X16\_PICPA | Q9FUH3\_VIGUS | Q9LYJ5\_ARATH | Q9P8F7\_YARLL | Q9STC1\_GRALE | Q9XEI3\_HORVV | QPCT1\_DROME | QPCT2\_DROME | QPCT\_IXOSC | QPCT\_MOUSE | RENI\_RAT | RGLA\_ASPAC | RHGA\_ASPAC | RNAS6\_HUMAN | RNLE\_SOLLC | RNT2\_HUMAN | S7Q6I2\_GLOTA | S7ZIW0\_PENO1 | SIA\_ASPFU | THCAS\_CANSA | TLP\_PRUAV | TPP1\_HUMAN | TRFL\_BUBBU | TRFL\_HORSE | TRY1\_GADMO | TRY3\_SALSA | TRYB2\_HUMAN | TTHY\_CHICK | TTHY\_MOUSE | TTHY\_RAT | V5NTD\_NAJAT | VM11\_BOTMO | VM12\_CROAD | VM1A3\_DEIAC | VM1BI\_BOTMO | VM1T1\_PROMU | VM1T2\_PROFL | VPL1\_PLEER | VPL2\_PLEER | VSPP\_DEIAC | VSPSX\_GLOSA | W0T408\_KLUMD | W4KMP1\_HETIT | W6Q990\_PENRF | W8P1L2\_TALEM | W8VR85\_TALPI | X0M5X0\_FUSOX | XGHA\_ASPTU | XTH34\_POPPZ | XYLA\_ASPNC | XYLO\_MYCTT | XYN1\_HYPJR | XYN2\_HYPJR | XYN3\_ASPKW | XYN3\_HYPJQ | XYNA\_FUSO4 | XYNA\_PENSI | XYNA\_THEAU | XYNA\_THELA | XYNC\_ASPNC | XYND\_EMENI | GO:0009653 | 0.0152079453755431 | 49/3222 | 12/552 | 0.120022137362975 | 1 | F | F | F | F | anatomical structure morphogenesis | AGAL\_ORYSJ | ANAG\_HUMAN | CATH\_HUMAN | GBA1\_HUMAN | HYAL1\_HUMAN | KLK8\_MOUSE | LICH\_HUMAN | PA2GX\_HUMAN | PPA5\_HUMAN | PPA5\_RAT | TRFL\_BUBBU | TRFL\_HORSE | GO:0071704 | 0.864059590316574 | 2784/3222 | 486/552 | 0.12114985907759 | 1 | F | F | F | F | organic substance metabolic process | A0A059U759\_9PEZI | A0A068FT77\_9PEZI | A0A075B5H6\_TRIHA | A0A086SY89\_ACRC1 | A0A086T6R4\_ACRC1 | A0A087WNH2\_FICBE | A0A088T0J9\_GEOCN | A0A0A0Y4H8\_TRAFO | A0A0J5Q413\_ASPFM | A0A0M3KKZ6\_RHIMI | A0A0M3KKZ8\_RHIMI | A0A0R3QSA7\_9BILA | A0A0R4I979\_BRABE | A0A0S2GKZ1\_9APHY | A0A173N065\_EISFE | A0A1L6CE30\_9EURO | A0A1L8D5Z7\_BOTAT | A0A1L9WG58\_ASPA1 | A0A1S4NYF8\_PANVG | A0A1S9DRB1\_ASPOZ | A0A2H5BN17\_TALPI | A0A2N1LTK3\_TRIHA | A0A2Z4HIN9\_9EURO | A0A384E148\_NICBE | A0A3B6UEQ2\_RHIMI | A0A3B6UEQ6\_EISFE | A0A3G2C3I4\_9EURO | A0A3G4RHU4\_9PEZI | A0A3L6SKP5\_PANMI | A0A3S5H5N2\_LEIDO | A0A482LWB1\_OSTFU | A0A5J6BJN2\_MALCI | A0A6F8Z6Y2\_BOMMO | A0A6M9BP13\_9EURO | A0A6P6YAT6\_DERPT | A0A7S6G7I6\_9PEZI | A0A856TAI5\_9BASI | A0NFU8\_ANOGA | A1E266\_9PEZI | A1HA\_LOXIN | A1HB2\_LOXIN | A2QZC8\_ASPNC | A2TM14\_HEVBR | A311\_LOXLA | A4GX63\_TOXGO | A5AB48\_ASPNC | A6PZ97\_SALSA | A6YRT4\_9PEZI | A8NI40\_COPC7 | A8PUY1\_MALGO | A8PUY5\_MALGO | A9LI60\_BIOOC | A9ZSX9\_9BRYO | ABFB\_ASPKW | ADA2\_HUMAN | ADPG2\_ARATH | AGAL\_HUMAN | AGAL\_ORYSJ | AMY1A\_HUMAN | AMY1\_HORVU | AMY1\_ORYSJ | AMYA1\_ASPOR | AMYG\_SACFI | AMY\_ORYLA | ANAG\_HUMAN | ANG1\_BOVIN | ANG2\_MOUSE | ANG3\_MOUSE | ANG4\_MOUSE | ANGI\_MOUSE | AOAH\_MOUSE | AOC1\_HUMAN | AOCX\_BOVIN | APO1\_CYCAE | ASAH1\_BALAS | ASM3A\_HUMAN | ASM3A\_MOUSE | AXE1\_ASPAW | AXE2\_TALPU | AXHA2\_EMENI | B2ZGS7\_9ASPA | B4F320\_LIMPO | B7X9Z0\_COPCI | B7X9Z2\_COPCI | B9TU22\_GADMO | BGALA\_ASPNC | BGALA\_ASPOR | BGALA\_PENSQ | BGL1\_ASPAC | BGLA\_ASPFU | BGLA\_ASPOR | BGLR\_HUMAN | C3VEV9\_PENCN | C7YSL3\_FUSV7 | CARP1\_CANAL | CARP2\_CANAX | CARP\_RHIPU | CAT3\_NEUCR | CATD\_RAT | CATH\_HUMAN | CATLL\_FASHE | CBHB\_ASPFU | CBHRE\_GEOS1 | CBPA1\_PIG | CBPD\_LOPSP | CBPN\_HUMAN | CDA\_COLLN | CDA\_EMENI | CEL2A\_PIG | CFAD\_MOUSE | CHI1\_COCPS | CHI2\_HORVU | CHI2\_ORYSJ | CHI33\_TRIHA | CHI42\_TRIHA | CHI4\_CRYJA | CHIA\_HUMAN | CHIC\_ARATH | CHIC\_SECCE | CHIL3\_MOUSE | CHIT\_PUNGR | CHLY\_HEVBR | CHYM\_CAMDR | CKX1\_MAIZE | COGS\_HYPLI | CONB\_CANEN | CUCM1\_CUCME | CUTI1\_ASPOR | CYP5\_CAEEL | CYSP\_BLOTA | D0QF43\_9HELO | D1M8S7\_HEVBR | D1MPT2\_ROYRE | D6XHE1\_TRYB2 | D9MWI4\_9ASPA | DDN1\_BOVIN | DIR\_GLYEC | DNAS1\_HUMAN | DNSL3\_HUMAN | DOPO\_HUMAN | DPP2\_HUMAN | E0A7J0\_YARLL | E0XN39\_9EURO | E13B\_HORVU | E13C\_MUSAC | E3VTL0\_9ASPA | E5D0X5\_SCHOC | E9G5J5\_DAPPU | ECP\_HUMAN | EGFB2\_MOUSE | EGLB\_ASPNG | ENDO2\_ARATH | ENG1\_RHIMI | ENPP2\_HUMAN | ENPP2\_RAT | ERVB\_TABDI | EST6\_DROME | EXG1\_CANAL | EXG1\_YEAST | F0ZJZ1\_DICPU | F1CYZ0\_TALFU | F2Z7L1\_9ANNE | FAEA\_ASPNG | FAEB1\_ASPOR | FAEB2\_ASPOR | FUCO\_HUMAN | G0RVK1\_HYPJQ | G2Q665\_MYCTT | G2QVH2\_THETT | G2X3Y1\_VERDV | G3I1H5\_CRIGR | G3JPF7\_CORMM | G3YAL0\_ASPNA | G3YFQ1\_ASPNA | G8GLP2\_LENED | G9NTY1\_HYPAI | GANA\_EMENI | GANA\_HUMIN | GBA1\_HUMAN | GCE2\_MYCTT | GCE\_CERUI | GCE\_HYPJQ | GGH\_HUMAN | GH7B\_LIMQU | GPX3\_HUMAN | GPX5\_HUMAN | GRAA\_HUMAN | GRAC\_MOUSE | GRAK\_HUMAN | GRASS\_DROME | GUB2\_HORVU | GUN2\_HYPJE | GUN6\_HUMIN | GUN7\_HYPJQ | GUNC\_FUSOX | GUN\_ASPAC | GUN\_CRYAT | GUN\_MYTED | GUX1\_HUMGT | GUX1\_HYPJE | GUX1\_TRIHA | GUX2\_HYPJE | H1AE14\_PHACH | HE12\_DANRE | HEXC\_OSTFU | HS3S1\_MOUSE | HYAL1\_HUMAN | I1SB18\_VIPAE | I2FI81\_EISFE | I3RY46\_TRIHA | IDH\_OSTTA | IDUA\_HUMAN | INU2\_ASPFI | INUE\_ASPAW | INV\_SCHOC | J7LCB0\_DEIAC | J9UN47\_GIBZA | K7CID1\_PANTR | K7N5L9\_RAPSA | K9L8F3\_MALCI | KATG2\_MAGO7 | KLK10\_HUMAN | KLK1\_HUMAN | KLK2\_HORSE | KLK2\_HUMAN | KLK7\_HUMAN | KLK7\_MOUSE | KLK8\_MOUSE | L7SVX1\_RHIMI | LAC1\_MELAO | LAC1\_TRAMX | LAC2\_TRAVE | LALBA\_BOVIN | LALBA\_CAPHI | LALBA\_CAVPO | LALBA\_PAPCY | LAPA\_ASPOR | LGMN\_MOUSE | LICH\_HUMAN | LIG2\_PHACH | LIG4\_PHACH | LIG8\_PHACH | LIP1\_DIURU | LIP2\_DIURU | LIP2\_GEOCN | LIP3\_DIURU | LIPA\_MOEAP | LIPB\_PSEA2 | LIPG\_CANLF | LIPG\_HUMAN | LIPP\_HORSE | LIPR1\_CANLF | LIPR1\_HUMAN | LIPR2\_HUMAN | LIPR2\_RAT | LIP\_THELA | LYG\_STRCA | M2RAI8\_CERS8 | M9TI89\_RHIPU | MAN12\_PENCI | MAN4\_SOLLC | MANA\_ASPNC | MANA\_CANEN | MANA\_CRYAT | MANA\_HYPJR | MANA\_MYTED | MANA\_PODAN | MANBA\_MOUSE | MCPT2\_RAT | MDLA\_PENCA | MDLA\_PENCY | MEL1\_YEASX | MMP1\_PIG | MNLOX\_MAGO7 | NAGAB\_CHICK | NAGAB\_HUMAN | NANL\_MACDE | NCS\_THLFG | NUP1\_PENCI | NUS1\_ASPOR | O00095\_HYPJE | O22443\_SOYBN | O74705\_ASPNG | O77044\_9NEOP | O81100\_SOLLC | O81226\_CARPA | O81934\_CANEN | O97389\_HELAM | OFUT1\_CAEEL | P78583\_ASPOZ | P79074\_9AGAR | PA1\_VESBA | PA21B\_BOVIN | PA21B\_PIG | PA2A1\_BUNCE | PA2A1\_ECHCA | PA2A1\_NAJAT | PA2A1\_OPHHA | PA2A2\_NAJNA | PA2A2\_OPHHA | PA2A2\_TROCA | PA2A4\_NAJSG | PA2A5\_TRIST | PA2A7\_GLOHA | PA2A\_BOTJR | PA2A\_CROAT | PA2A\_DEIAC | PA2A\_GLOHA | PA2A\_NAJAT | PA2B1\_AGKPI | PA2B2\_BOTJR | PA2B2\_PROFL | PA2B3\_BOTAS | PA2B3\_BUNCE | PA2B5\_BUNCE | PA2B5\_NOTSC | PA2BA\_VIPAA | PA2BB\_GLOHA | PA2BB\_PSEAU | PA2BC\_VIPAA | PA2BD\_CRODU | PA2B\_BUNCE | PA2B\_NOTSC | PA2GA\_HUMAN | PA2GE\_HUMAN | PA2GX\_HUMAN | PA2H1\_AGKCL | PA2H1\_BOTBZ | PA2H1\_BOTJR | PA2H1\_BOTMO | PA2H1\_BOTPI | PA2H2\_BOTAS | PA2H2\_BOTMO | PA2H2\_BOTPI | PA2H2\_CERGO | PA2H3\_BOTPI | PA2HB\_AGKPI | PA2HB\_OXYSC | PA2HH\_TRIST | PA2HS\_ECHCA | PA2H\_BOTPA | PA2H\_DEIAC | PA2H\_PROMB | PA2N\_GLOHA | PA2\_APIME | PAG15\_HUMAN | PCP\_HUMAN | PDH1\_LEUMG | PELA\_ASPNG | PELB\_ASPNG | PEM1\_PHACH | PEPA\_ASPPH | PER1A\_ARMRU | PER1\_ARAHY | PER1\_SORBI | PER53\_ARATH | PER59\_ARATH | PERL\_BOVIN | PERL\_BUBBU | PERL\_CAPHI | PER\_ARTRA | PER\_COPCI | PGH2\_HUMAN | PGLR1\_ASPAC | PGLR1\_ASPNG | PGLR\_GIBFU | PGPSA\_DROME | PGRP1\_CAMDR | PHAZ\_TALFU | PLA22\_ORYSJ | PLY1\_JUNAS | PME\_DAUCA | PME\_SITOR | POXA\_DICDI | PPAF1\_HOLDI | PPAP\_RAT | PPT1\_BOVIN | PPT1\_HUMAN | PPT2\_HUMAN | PRS57\_HUMAN | PRTN3\_HUMAN | PTGDS\_HUMAN | PTGDS\_MOUSE | Q02321\_PHACH | Q06AK3\_TOXGO | Q07524\_TROMA | Q0KFV0\_SOLLC | Q12715\_HYPJE | Q2U8V9\_ASPOR | Q40069\_HORVU | Q43576\_TOBAC | Q4AE59\_OSTFU | Q4W6L6\_CYCRE | Q4WP32\_ASPFU | Q50KB2\_PHACH | Q55FE6\_DICDI | Q588B8\_CRYJA | Q5B038\_EMENI | Q5WRG2\_RAT | Q60FD2\_9APHY | Q69G21\_TENMO | Q6NY42\_DANRE | Q6R7Z5\_9TRYP | Q6VAY1\_9PEZI | Q6WER3\_GIBZA | Q6WSR8\_PICAB | Q70C53\_SOLTU | Q70SY0\_HYPJE | Q7LHI2\_PHACH | Q7LIJ0\_PHACH | Q7LST4\_PENEN | Q7RWP2\_NEUCR | Q7X9A9\_CAMSI | Q7YXL2\_TENMO | Q86RS6\_MANSE | Q8H0C9\_VIGUN | Q8J0K6\_MELAO | Q8J0K8\_MELAO | Q8NJY6\_9HYPO | Q8T0W7\_9NEOP | Q8TFL9\_TALEM | Q8TG26\_THEAU | Q8TGI8\_TALEM | Q92456\_HYPJE | Q92458\_HYPJE | Q93X60\_CICIN | Q95KP4\_HORSE | Q96X16\_PICPA | Q9FUH3\_VIGUS | Q9LYJ5\_ARATH | Q9P8F7\_YARLL | Q9STC1\_GRALE | Q9XEI3\_HORVV | QPCT1\_DROME | QPCT2\_DROME | QPCT\_IXOSC | QPCT\_MOUSE | RENI\_RAT | RGLA\_ASPAC | RHGA\_ASPAC | RNAS6\_HUMAN | RNLE\_SOLLC | RNT2\_HUMAN | S7Q6I2\_GLOTA | S7ZIW0\_PENO1 | SIA\_ASPFU | THCAS\_CANSA | TPP1\_HUMAN | TRFL\_BUBBU | TRFL\_HORSE | TRY1\_GADMO | TRY3\_SALSA | TRYB2\_HUMAN | TTHY\_CHICK | TTHY\_MOUSE | TTHY\_RAT | V5NTD\_NAJAT | VM11\_BOTMO | VM12\_CROAD | VM1A3\_DEIAC | VM1BI\_BOTMO | VM1T1\_PROMU | VM1T2\_PROFL | VPL1\_PLEER | VPL2\_PLEER | VSPP\_DEIAC | VSPSX\_GLOSA | W0T408\_KLUMD | W4KMP1\_HETIT | W6Q990\_PENRF | W8P1L2\_TALEM | W8VR85\_TALPI | X0M5X0\_FUSOX | XGHA\_ASPTU | XTH34\_POPPZ | XYLA\_ASPNC | XYN1\_HYPJR | XYN2\_HYPJR | XYN3\_ASPKW | XYN3\_HYPJQ | XYNA\_FUSO4 | XYNA\_PENSI | XYNA\_THEAU | XYNA\_THELA | XYNC\_ASPNC | XYND\_EMENI | GO:0050708 | 0.00775915580384854 | 25/3222 | 7/552 | 0.121412784118218 | 1 | F | F | F | F | regulation of protein secretion | ANG1\_BOVIN | ANG2\_MOUSE | ANG3\_MOUSE | ANG4\_MOUSE | ANGI\_MOUSE | CEL2A\_PIG | Q5WRG2\_RAT | GO:0045859 | 0.00775915580384854 | 25/3222 | 7/552 | 0.121412784118218 | 1 | F | F | F | F | regulation of protein kinase activity | ANG2\_MOUSE | ANG3\_MOUSE | ANG4\_MOUSE | ANGI\_MOUSE | GBA1\_HUMAN | PA21B\_PIG | Q5WRG2\_RAT | GO:2000241 | 0.00496585971446307 | 16/3222 | 5/552 | 0.123683758619819 | 1 | F | F | F | F | regulation of reproductive process | AGAL\_ORYSJ | EST6\_DROME | PA2GX\_HUMAN | PTGDS\_HUMAN | PTGDS\_MOUSE | GO:0031349 | 0.00931098696461825 | 30/3222 | 8/552 | 0.127272079886824 | 1 | F | F | F | F | positive regulation of defense response | ANAG\_HUMAN | CARP1\_CANAL | GRASS\_DROME | KLK7\_HUMAN | KLK7\_MOUSE | PA2GA\_HUMAN | PGH2\_HUMAN | PGPSA\_DROME | GO:0001932 | 0.0124146492861577 | 40/3222 | 10/552 | 0.133209080633641 | 1 | F | F | F | F | regulation of protein phosphorylation | ANG2\_MOUSE | ANG3\_MOUSE | ANG4\_MOUSE | ANGI\_MOUSE | ENPP2\_HUMAN | ENPP2\_RAT | GBA1\_HUMAN | PA21B\_PIG | PGH2\_HUMAN | Q5WRG2\_RAT | GO:0001817 | 0.0139664804469274 | 45/3222 | 11/552 | 0.134363027824738 | 1 | F | F | F | F | regulation of cytokine production | CHIA\_HUMAN | CHIL3\_MOUSE | GBA1\_HUMAN | PA21B\_PIG | PA2GX\_HUMAN | PGH2\_HUMAN | PGRP1\_CAMDR | PPA5\_HUMAN | PPA5\_RAT | TRFL\_BUBBU | TRFL\_HORSE | GO:0006935 | 0.0037243947858473 | 12/3222 | 4/552 | 0.1349109983135 | 1 | F | F | F | F | chemotaxis | ECP\_HUMAN | ENPP2\_HUMAN | ENPP2\_RAT | PA21B\_PIG | GO:0051347 | 0.00651769087523277 | 21/3222 | 6/552 | 0.135708093297396 | 1 | F | F | F | F | positive regulation of transferase activity | ANG2\_MOUSE | ANG3\_MOUSE | ANG4\_MOUSE | ANGI\_MOUSE | PA21B\_PIG | Q5WRG2\_RAT | GO:0042116 | 0.00124146492861577 | 4/3222 | 2/552 | 0.138357382916998 | 1 | F | F | F | F | macrophage activation | ANAG\_HUMAN | PA2GX\_HUMAN | GO:0008544 | 0.00124146492861577 | 4/3222 | 2/552 | 0.138357382916998 | 1 | F | F | F | F | epidermis development | GBA1\_HUMAN | KLK7\_HUMAN | GO:0061744 | 0.00124146492861577 | 4/3222 | 2/552 | 0.138357382916998 | 1 | F | F | F | F | motor behavior | ANAG\_HUMAN | GBA1\_HUMAN | GO:0032691 | 0.00124146492861577 | 4/3222 | 2/552 | 0.138357382916998 | 1 | F | F | F | F | negative regulation of interleukin-1 beta production | PPA5\_HUMAN | PPA5\_RAT | GO:0045667 | 0.00124146492861577 | 4/3222 | 2/552 | 0.138357382916998 | 1 | F | F | F | F | regulation of osteoblast differentiation | TRFL\_BUBBU | TRFL\_HORSE | GO:0036230 | 0.00124146492861577 | 4/3222 | 2/552 | 0.138357382916998 | 1 | F | F | F | F | granulocyte activation | DNAS1\_HUMAN | DNSL3\_HUMAN | GO:0019374 | 0.00124146492861577 | 4/3222 | 2/552 | 0.138357382916998 | 1 | F | F | F | F | galactolipid metabolic process | LIPR2\_HUMAN | LIPR2\_RAT | GO:0001573 | 0.00124146492861577 | 4/3222 | 2/552 | 0.138357382916998 | 1 | F | F | F | F | ganglioside metabolic process | ANAG\_HUMAN | SIA\_ASPFU | GO:0022404 | 0.00124146492861577 | 4/3222 | 2/552 | 0.138357382916998 | 1 | F | F | F | F | molting cycle process | ANAG\_HUMAN | PA2GX\_HUMAN | GO:0006677 | 0.00124146492861577 | 4/3222 | 2/552 | 0.138357382916998 | 1 | F | F | F | F | glycosylceramide metabolic process | AGAL\_HUMAN | GBA1\_HUMAN | GO:2000255 | 0.00124146492861577 | 4/3222 | 2/552 | 0.138357382916998 | 1 | F | F | F | F | negative regulation of male germ cell proliferation | PTGDS\_HUMAN | PTGDS\_MOUSE | GO:0097061 | 0.00124146492861577 | 4/3222 | 2/552 | 0.138357382916998 | 1 | F | F | F | F | dendritic spine organization | G3I1H5\_CRIGR | LGMN\_MOUSE | GO:0046473 | 0.00124146492861577 | 4/3222 | 2/552 | 0.138357382916998 | 1 | F | F | F | F | phosphatidic acid metabolic process | PA2GA\_HUMAN | PA2GX\_HUMAN | GO:0002886 | 0.00124146492861577 | 4/3222 | 2/552 | 0.138357382916998 | 1 | F | F | F | F | regulation of myeloid leukocyte mediated immunity | DNAS1\_HUMAN | DNSL3\_HUMAN | GO:0030202 | 0.00124146492861577 | 4/3222 | 2/552 | 0.138357382916998 | 1 | F | F | F | F | heparin metabolic process | ANAG\_HUMAN | IDUA\_HUMAN | GO:0070167 | 0.00124146492861577 | 4/3222 | 2/552 | 0.138357382916998 | 1 | F | F | F | F | regulation of biomineral tissue development | TRFL\_BUBBU | TRFL\_HORSE | GO:0032692 | 0.00124146492861577 | 4/3222 | 2/552 | 0.138357382916998 | 1 | F | F | F | F | negative regulation of interleukin-1 production | PPA5\_HUMAN | PPA5\_RAT | GO:1900271 | 0.00124146492861577 | 4/3222 | 2/552 | 0.138357382916998 | 1 | F | F | F | F | regulation of long-term synaptic potentiation | G3I1H5\_CRIGR | LGMN\_MOUSE | GO:0010721 | 0.00124146492861577 | 4/3222 | 2/552 | 0.138357382916998 | 1 | F | F | F | F | negative regulation of cell development | TRFL\_BUBBU | TRFL\_HORSE | GO:0045907 | 0.00124146492861577 | 4/3222 | 2/552 | 0.138357382916998 | 1 | F | F | F | F | positive regulation of vasoconstriction | DOPO\_HUMAN | PGH2\_HUMAN | GO:0046477 | 0.00124146492861577 | 4/3222 | 2/552 | 0.138357382916998 | 1 | F | F | F | F | glycosylceramide catabolic process | AGAL\_HUMAN | GBA1\_HUMAN | GO:0019376 | 0.00124146492861577 | 4/3222 | 2/552 | 0.138357382916998 | 1 | F | F | F | F | galactolipid catabolic process | LIPR2\_HUMAN | LIPR2\_RAT | GO:0030500 | 0.00124146492861577 | 4/3222 | 2/552 | 0.138357382916998 | 1 | F | F | F | F | regulation of bone mineralization | TRFL\_BUBBU | TRFL\_HORSE | GO:0099173 | 0.00124146492861577 | 4/3222 | 2/552 | 0.138357382916998 | 1 | F | F | F | F | postsynapse organization | G3I1H5\_CRIGR | LGMN\_MOUSE | GO:0032655 | 0.00124146492861577 | 4/3222 | 2/552 | 0.138357382916998 | 1 | F | F | F | F | regulation of interleukin-12 production | PPA5\_HUMAN | PPA5\_RAT | GO:0042554 | 0.00124146492861577 | 4/3222 | 2/552 | 0.138357382916998 | 1 | F | F | F | F | superoxide anion generation | PPA5\_HUMAN | PPA5\_RAT | GO:0006080 | 0.00124146492861577 | 4/3222 | 2/552 | 0.138357382916998 | 1 | F | F | F | F | substituted mannan metabolic process | A0A3G4RHU4\_9PEZI | AGAL\_ORYSJ | GO:0071677 | 0.00124146492861577 | 4/3222 | 2/552 | 0.138357382916998 | 1 | F | F | F | F | positive regulation of mononuclear cell migration | G3I1H5\_CRIGR | LGMN\_MOUSE | GO:0043062 | 0.00124146492861577 | 4/3222 | 2/552 | 0.138357382916998 | 1 | F | F | F | F | extracellular structure organization | KLK7\_HUMAN | MMP1\_PIG | GO:0048259 | 0.00124146492861577 | 4/3222 | 2/552 | 0.138357382916998 | 1 | F | F | F | F | regulation of receptor-mediated endocytosis | PPT1\_BOVIN | PPT1\_HUMAN | GO:0030198 | 0.00124146492861577 | 4/3222 | 2/552 | 0.138357382916998 | 1 | F | F | F | F | extracellular matrix organization | KLK7\_HUMAN | MMP1\_PIG | GO:0010543 | 0.00124146492861577 | 4/3222 | 2/552 | 0.138357382916998 | 1 | F | F | F | F | regulation of platelet activation | CEL2A\_PIG | OXLA\_BOTAT | GO:0031579 | 0.00124146492861577 | 4/3222 | 2/552 | 0.138357382916998 | 1 | F | F | F | F | membrane raft organization | PPT1\_BOVIN | PPT1\_HUMAN | GO:0022405 | 0.00124146492861577 | 4/3222 | 2/552 | 0.138357382916998 | 1 | F | F | F | F | hair cycle process | ANAG\_HUMAN | PA2GX\_HUMAN | GO:0006898 | 0.00124146492861577 | 4/3222 | 2/552 | 0.138357382916998 | 1 | F | F | F | F | receptor-mediated endocytosis | PPT1\_BOVIN | PPT1\_HUMAN | GO:2000243 | 0.00124146492861577 | 4/3222 | 2/552 | 0.138357382916998 | 1 | F | F | F | F | positive regulation of reproductive process | AGAL\_ORYSJ | PA2GX\_HUMAN | GO:1905937 | 0.00124146492861577 | 4/3222 | 2/552 | 0.138357382916998 | 1 | F | F | F | F | negative regulation of germ cell proliferation | PTGDS\_HUMAN | PTGDS\_MOUSE | GO:0050673 | 0.00124146492861577 | 4/3222 | 2/552 | 0.138357382916998 | 1 | F | F | F | F | epithelial cell proliferation | KLK8\_MOUSE | LICH\_HUMAN | GO:0106027 | 0.00124146492861577 | 4/3222 | 2/552 | 0.138357382916998 | 1 | F | F | F | F | neuron projection organization | G3I1H5\_CRIGR | LGMN\_MOUSE | GO:0046337 | 0.00248292985723153 | 8/3222 | 3/552 | 0.143458586022055 | 1 | F | F | F | F | phosphatidylethanolamine metabolic process | PA2GA\_HUMAN | PA2GX\_HUMAN | PAG15\_HUMAN | GO:0009593 | 0.00248292985723153 | 8/3222 | 3/552 | 0.143458586022055 | 1 | F | F | F | F | detection of chemical stimulus | CAH6\_HUMAN | L8ICE9\_9CETA | PERL\_CAPHI | GO:0071825 | 0.00248292985723153 | 8/3222 | 3/552 | 0.143458586022055 | 1 | F | F | F | F | protein-lipid complex organization | PA2GA\_HUMAN | PA2GE\_HUMAN | PA2GX\_HUMAN | GO:0010595 | 0.00248292985723153 | 8/3222 | 3/552 | 0.143458586022055 | 1 | F | F | F | F | positive regulation of endothelial cell migration | G3I1H5\_CRIGR | LGMN\_MOUSE | PGH2\_HUMAN | GO:0045931 | 0.00248292985723153 | 8/3222 | 3/552 | 0.143458586022055 | 1 | F | F | F | F | positive regulation of mitotic cell cycle | G3I1H5\_CRIGR | HYAL1\_HUMAN | LGMN\_MOUSE | GO:0071827 | 0.00248292985723153 | 8/3222 | 3/552 | 0.143458586022055 | 1 | F | F | F | F | plasma lipoprotein particle organization | PA2GA\_HUMAN | PA2GE\_HUMAN | PA2GX\_HUMAN | GO:0042157 | 0.00248292985723153 | 8/3222 | 3/552 | 0.143458586022055 | 1 | F | F | F | F | lipoprotein metabolic process | CATD\_RAT | PPT1\_BOVIN | PPT1\_HUMAN | GO:1903131 | 0.00248292985723153 | 8/3222 | 3/552 | 0.143458586022055 | 1 | F | F | F | F | mononuclear cell differentiation | GBA1\_HUMAN | LICH\_HUMAN | PRTN3\_HUMAN | GO:0002673 | 0.00248292985723153 | 8/3222 | 3/552 | 0.143458586022055 | 1 | F | F | F | F | regulation of acute inflammatory response | DNAS1\_HUMAN | DNSL3\_HUMAN | PGH2\_HUMAN | GO:0042159 | 0.00248292985723153 | 8/3222 | 3/552 | 0.143458586022055 | 1 | F | F | F | F | lipoprotein catabolic process | CATD\_RAT | PPT1\_BOVIN | PPT1\_HUMAN | GO:0071852 | 0.00248292985723153 | 8/3222 | 3/552 | 0.143458586022055 | 1 | F | F | F | F | fungal-type cell wall organization or biogenesis | CARP1\_CANAL | EXG1\_CANAL | EXG1\_YEAST | GO:0008340 | 0.00248292985723153 | 8/3222 | 3/552 | 0.143458586022055 | 1 | F | F | F | F | determination of adult lifespan | ANAG\_HUMAN | GBA1\_HUMAN | LICH\_HUMAN | GO:0060341 | 0.0127250155183116 | 41/3222 | 10/552 | 0.150764562960464 | 1 | F | F | F | F | regulation of cellular localization | ANG1\_BOVIN | ANG2\_MOUSE | ANG3\_MOUSE | ANG4\_MOUSE | ANGI\_MOUSE | CATD\_RAT | CEL2A\_PIG | PA21B\_PIG | PGH2\_HUMAN | Q5WRG2\_RAT | GO:0008217 | 0.00682805710738672 | 22/3222 | 6/552 | 0.161141490814221 | 1 | F | F | F | F | regulation of blood pressure | EGFB2\_MOUSE | KLK1\_HUMAN | PA2A\_BOTJR | PCP\_HUMAN | PGH2\_HUMAN | RENI\_RAT | GO:0051047 | 0.00837988826815642 | 27/3222 | 7/552 | 0.166226463377029 | 1 | F | F | F | F | positive regulation of secretion | ANG1\_BOVIN | ANG2\_MOUSE | ANG3\_MOUSE | ANG4\_MOUSE | ANGI\_MOUSE | PA2GX\_HUMAN | Q5WRG2\_RAT | GO:1903532 | 0.00837988826815642 | 27/3222 | 7/552 | 0.166226463377029 | 1 | F | F | F | F | positive regulation of secretion by cell | ANG1\_BOVIN | ANG2\_MOUSE | ANG3\_MOUSE | ANG4\_MOUSE | ANGI\_MOUSE | PA2GX\_HUMAN | Q5WRG2\_RAT | GO:0010468 | 0.0642458100558659 | 207/3222 | 41/552 | 0.167973297134619 | 1 | F | F | F | F | regulation of gene expression | ANG1\_BOVIN | ANG3\_MOUSE | ANGI\_MOUSE | CATH\_HUMAN | CHIA\_HUMAN | CHIL3\_MOUSE | D6XHE1\_TRYB2 | E0CX04\_MOMBA | G3I1H5\_CRIGR | GBA1\_HUMAN | GRAC\_MOUSE | GRASS\_DROME | KLK7\_HUMAN | KLK7\_MOUSE | LGMN\_MOUSE | O04358\_IRIHO | PA21B\_PIG | PA2GX\_HUMAN | PGH2\_HUMAN | PGPSA\_DROME | PGRP1\_CAMDR | PPA5\_HUMAN | PPA5\_RAT | Q2QEH4\_SAPOF | Q5WRG2\_RAT | Q6R7Z5\_9TRYP | Q94BW3\_CINCA | RIP0\_DIACA | RIP1\_BRYDI | RIP1\_HORVU | RIP1\_MOMCH | RIP1\_PHYAM | RIP2\_PHYAM | RIP3\_MOMCH | RIPA\_PHYAM | RIPG\_SURMU | RIPL1\_PHYDI | RIPL2\_PHYDI | RIPT\_TRIKI | TRFL\_BUBBU | TRFL\_HORSE | GO:0032880 | 0.0114835505896958 | 37/3222 | 9/552 | 0.169640676542738 | 1 | F | F | F | F | regulation of protein localization | ANG1\_BOVIN | ANG2\_MOUSE | ANG3\_MOUSE | ANG4\_MOUSE | ANGI\_MOUSE | CATD\_RAT | CEL2A\_PIG | PGH2\_HUMAN | Q5WRG2\_RAT | GO:0032940 | 0.00403476101800124 | 13/3222 | 4/552 | 0.169831716274382 | 1 | F | F | F | F | secretion by cell | PGH2\_HUMAN | PPT1\_HUMAN | PTGDS\_HUMAN | PTGDS\_MOUSE | GO:0140352 | 0.00403476101800124 | 13/3222 | 4/552 | 0.169831716274382 | 1 | F | F | F | F | export from cell | PGH2\_HUMAN | PPT1\_HUMAN | PTGDS\_HUMAN | PTGDS\_MOUSE | GO:0002833 | 0.00558659217877095 | 18/3222 | 5/552 | 0.182364306590718 | 1 | F | F | F | F | positive regulation of response to biotic stimulus | ANAG\_HUMAN | GRASS\_DROME | KLK7\_HUMAN | KLK7\_MOUSE | PGPSA\_DROME | GO:0045926 | 0.00558659217877095 | 18/3222 | 5/552 | 0.182364306590718 | 1 | F | F | F | F | negative regulation of growth | G3I1H5\_CRIGR | HYAL1\_HUMAN | LGMN\_MOUSE | PPT1\_BOVIN | PPT1\_HUMAN | GO:0001503 | 0.00279329608938547 | 9/3222 | 3/552 | 0.189058532427702 | 1 | F | F | F | F | ossification | PPA5\_RAT | TRFL\_BUBBU | TRFL\_HORSE | GO:0030534 | 0.00279329608938547 | 9/3222 | 3/552 | 0.189058532427702 | 1 | F | F | F | F | adult behavior | ANAG\_HUMAN | DOPO\_HUMAN | PPT1\_HUMAN | GO:0003073 | 0.00279329608938547 | 9/3222 | 3/552 | 0.189058532427702 | 1 | F | F | F | F | regulation of systemic arterial blood pressure | EGFB2\_MOUSE | KLK1\_HUMAN | RENI\_RAT | GO:0046514 | 0.00279329608938547 | 9/3222 | 3/552 | 0.189058532427702 | 1 | F | F | F | F | ceramide catabolic process | AGAL\_HUMAN | GBA1\_HUMAN | SIA\_ASPFU | GO:0050806 | 0.00279329608938547 | 9/3222 | 3/552 | 0.189058532427702 | 1 | F | F | F | F | positive regulation of synaptic transmission | G3I1H5\_CRIGR | LGMN\_MOUSE | PGH2\_HUMAN | GO:0048646 | 0.0133457479826195 | 43/3222 | 10/552 | 0.189126800286508 | 1 | F | F | F | F | anatomical structure formation involved in morphogenesis | ANG1\_BOVIN | ANG2\_MOUSE | ANG3\_MOUSE | ANG4\_MOUSE | ANGI\_MOUSE | GBA1\_HUMAN | PCP\_HUMAN | PGH2\_HUMAN | Q5WRG2\_RAT | RNSL3\_DANRE | GO:0051223 | 0.0102420856610801 | 33/3222 | 8/552 | 0.191536485480543 | 1 | F | F | F | F | regulation of protein transport | ANG1\_BOVIN | ANG2\_MOUSE | ANG3\_MOUSE | ANG4\_MOUSE | ANGI\_MOUSE | CEL2A\_PIG | PGH2\_HUMAN | Q5WRG2\_RAT | GO:0016043 | 0.0738671632526381 | 238/3222 | 46/552 | 0.197768417786585 | 1 | F | F | F | F | cellular component organization | A0A6M9BP13\_9EURO | A1E266\_9PEZI | ADPG2\_ARATH | ANAG\_HUMAN | ANG1\_BOVIN | ANGI\_MOUSE | CARP1\_CANAL | CATD\_RAT | CDA\_COLLN | CDA\_EMENI | ENG1\_RHIMI | EXG1\_CANAL | EXG1\_YEAST | G3I1H5\_CRIGR | G3YAL0\_ASPNA | GANA\_EMENI | GBA1\_HUMAN | KLK7\_HUMAN | KLK8\_MOUSE | LGMN\_MOUSE | LICH\_HUMAN | MMP1\_PIG | P79074\_9AGAR | PA2GA\_HUMAN | PA2GE\_HUMAN | PA2GX\_HUMAN | PELA\_ASPNG | PELB\_ASPNG | PGLR1\_ASPAC | PGLR1\_ASPNG | PGLR\_GIBFU | PME\_DAUCA | PME\_SITOR | PPAP\_RAT | PPT1\_BOVIN | PPT1\_HUMAN | PRTN3\_HUMAN | Q07524\_TROMA | Q5WRG2\_RAT | Q9LYJ5\_ARATH | RGLA\_ASPAC | RHGA\_ASPAC | TPP1\_HUMAN | TTHY\_CHICK | XGHA\_ASPTU | XTH34\_POPPZ | GO:0010556 | 0.0654872749844817 | 211/3222 | 41/552 | 0.203751660838522 | 1 | F | F | F | F | regulation of macromolecule biosynthetic process | ANG1\_BOVIN | ANG3\_MOUSE | ANGI\_MOUSE | CATH\_HUMAN | CHIA\_HUMAN | CHIL3\_MOUSE | D6XHE1\_TRYB2 | E0CX04\_MOMBA | G3I1H5\_CRIGR | GBA1\_HUMAN | GRAC\_MOUSE | GRASS\_DROME | KLK7\_HUMAN | KLK7\_MOUSE | LGMN\_MOUSE | O04358\_IRIHO | PA21B\_PIG | PA2GX\_HUMAN | PGH2\_HUMAN | PGPSA\_DROME | PGRP1\_CAMDR | PPA5\_HUMAN | PPA5\_RAT | Q2QEH4\_SAPOF | Q5WRG2\_RAT | Q6R7Z5\_9TRYP | Q94BW3\_CINCA | RIP0\_DIACA | RIP1\_BRYDI | RIP1\_HORVU | RIP1\_MOMCH | RIP1\_PHYAM | RIP2\_PHYAM | RIP3\_MOMCH | RIPA\_PHYAM | RIPG\_SURMU | RIPL1\_PHYDI | RIPL2\_PHYDI | RIPT\_TRIKI | TRFL\_BUBBU | TRFL\_HORSE | GO:0006979 | 0.0552451893234016 | 178/3222 | 35/552 | 0.20432188691579 | 1 | F | F | F | F | response to oxidative stress | A0A087WNH2\_FICBE | A0A0A0Y4H8\_TRAFO | A0A1S4NYF8\_PANVG | A0A3L6SKP5\_PANMI | ANAG\_HUMAN | CAT3\_NEUCR | D1MPT2\_ROYRE | GPX3\_HUMAN | GPX5\_HUMAN | GPX6\_MOUSE | HYAL1\_HUMAN | K7N5L9\_RAPSA | KATG2\_MAGO7 | L8ICE9\_9CETA | LIG2\_PHACH | LIG4\_PHACH | LIG8\_PHACH | O22443\_SOYBN | PEM1\_PHACH | PER1A\_ARMRU | PER1\_ARAHY | PER1\_SORBI | PER53\_ARATH | PER59\_ARATH | PERL\_BOVIN | PERL\_BUBBU | PERL\_CAPHI | PER\_ARTRA | PER\_COPCI | PGH2\_HUMAN | POXA\_DICDI | Q40069\_HORVU | Q60FD2\_9APHY | VPL1\_PLEER | VPL2\_PLEER | GO:0042130 | 0.00155183116076971 | 5/3222 | 2/552 | 0.205179030450101 | 1 | F | F | F | F | negative regulation of T cell proliferation | PA2GA\_HUMAN | PA2GE\_HUMAN | GO:0031665 | 0.00155183116076971 | 5/3222 | 2/552 | 0.205179030450101 | 1 | F | F | F | F | negative regulation of lipopolysaccharide-mediated signaling pathway | TRFL\_BUBBU | TRFL\_HORSE | GO:0051452 | 0.00155183116076971 | 5/3222 | 2/552 | 0.205179030450101 | 1 | F | F | F | F | intracellular pH reduction | PPT1\_BOVIN | PPT1\_HUMAN | GO:0019229 | 0.00155183116076971 | 5/3222 | 2/552 | 0.205179030450101 | 1 | F | F | F | F | regulation of vasoconstriction | DOPO\_HUMAN | PGH2\_HUMAN | GO:0051453 | 0.00155183116076971 | 5/3222 | 2/552 | 0.205179030450101 | 1 | F | F | F | F | regulation of intracellular pH | PPT1\_BOVIN | PPT1\_HUMAN | GO:0050905 | 0.00155183116076971 | 5/3222 | 2/552 | 0.205179030450101 | 1 | F | F | F | F | neuromuscular process | GBA1\_HUMAN | TPP1\_HUMAN | GO:0002690 | 0.00155183116076971 | 5/3222 | 2/552 | 0.205179030450101 | 1 | F | F | F | F | positive regulation of leukocyte chemotaxis | G3I1H5\_CRIGR | LGMN\_MOUSE | GO:0051703 | 0.00155183116076971 | 5/3222 | 2/552 | 0.205179030450101 | 1 | F | F | F | F | biological process involved in intraspecies interaction between organisms | EXG1\_CANAL | Q55FE6\_DICDI | GO:0022411 | 0.00155183116076971 | 5/3222 | 2/552 | 0.205179030450101 | 1 | F | F | F | F | cellular component disassembly | ADPG2\_ARATH | KLK7\_HUMAN | GO:0033555 | 0.00155183116076971 | 5/3222 | 2/552 | 0.205179030450101 | 1 | F | F | F | F | multicellular organismal response to stress | DOPO\_HUMAN | PPA5\_RAT | GO:0009566 | 0.00155183116076971 | 5/3222 | 2/552 | 0.205179030450101 | 1 | F | F | F | F | fertilization | NADA\_APLCA | PA2GX\_HUMAN | GO:0019098 | 0.00155183116076971 | 5/3222 | 2/552 | 0.205179030450101 | 1 | F | F | F | F | reproductive behavior | DOPO\_HUMAN | EST6\_DROME | GO:0032928 | 0.00155183116076971 | 5/3222 | 2/552 | 0.205179030450101 | 1 | F | F | F | F | regulation of superoxide anion generation | PPA5\_HUMAN | PPA5\_RAT | GO:1905936 | 0.00155183116076971 | 5/3222 | 2/552 | 0.205179030450101 | 1 | F | F | F | F | regulation of germ cell proliferation | PTGDS\_HUMAN | PTGDS\_MOUSE | GO:0010447 | 0.00155183116076971 | 5/3222 | 2/552 | 0.205179030450101 | 1 | F | F | F | F | response to acidic pH | G3I1H5\_CRIGR | LGMN\_MOUSE | GO:0045055 | 0.00155183116076971 | 5/3222 | 2/552 | 0.205179030450101 | 1 | F | F | F | F | regulated exocytosis | PTGDS\_HUMAN | PTGDS\_MOUSE | GO:0032720 | 0.00155183116076971 | 5/3222 | 2/552 | 0.205179030450101 | 1 | F | F | F | F | negative regulation of tumor necrosis factor production | PPA5\_HUMAN | PPA5\_RAT | GO:0048640 | 0.00155183116076971 | 5/3222 | 2/552 | 0.205179030450101 | 1 | F | F | F | F | negative regulation of developmental growth | G3I1H5\_CRIGR | LGMN\_MOUSE | GO:0019433 | 0.00155183116076971 | 5/3222 | 2/552 | 0.205179030450101 | 1 | F | F | F | F | triglyceride catabolic process | LIPR2\_HUMAN | LIPR2\_RAT | GO:2000254 | 0.00155183116076971 | 5/3222 | 2/552 | 0.205179030450101 | 1 | F | F | F | F | regulation of male germ cell proliferation | PTGDS\_HUMAN | PTGDS\_MOUSE | GO:0050672 | 0.00155183116076971 | 5/3222 | 2/552 | 0.205179030450101 | 1 | F | F | F | F | negative regulation of lymphocyte proliferation | PA2GA\_HUMAN | PA2GE\_HUMAN | GO:1990266 | 0.00155183116076971 | 5/3222 | 2/552 | 0.205179030450101 | 1 | F | F | F | F | neutrophil migration | PA21B\_PIG | PRTN3\_HUMAN | GO:1905037 | 0.00155183116076971 | 5/3222 | 2/552 | 0.205179030450101 | 1 | F | F | F | F | autophagosome organization | CATD\_RAT | GBA1\_HUMAN | GO:0060326 | 0.00155183116076971 | 5/3222 | 2/552 | 0.205179030450101 | 1 | F | F | F | F | cell chemotaxis | ENPP2\_RAT | PA21B\_PIG | GO:0001909 | 0.00155183116076971 | 5/3222 | 2/552 | 0.205179030450101 | 1 | F | F | F | F | leukocyte mediated cytotoxicity | CATH\_HUMAN | GRAC\_MOUSE | GO:0032945 | 0.00155183116076971 | 5/3222 | 2/552 | 0.205179030450101 | 1 | F | F | F | F | negative regulation of mononuclear cell proliferation | PA2GA\_HUMAN | PA2GE\_HUMAN | GO:0097530 | 0.00155183116076971 | 5/3222 | 2/552 | 0.205179030450101 | 1 | F | F | F | F | granulocyte migration | PA21B\_PIG | PRTN3\_HUMAN | GO:0002449 | 0.00155183116076971 | 5/3222 | 2/552 | 0.205179030450101 | 1 | F | F | F | F | lymphocyte mediated immunity | CATH\_HUMAN | GRAC\_MOUSE | GO:0050921 | 0.00155183116076971 | 5/3222 | 2/552 | 0.205179030450101 | 1 | F | F | F | F | positive regulation of chemotaxis | G3I1H5\_CRIGR | LGMN\_MOUSE | GO:0009312 | 0.00434512725015518 | 14/3222 | 4/552 | 0.20747622847229 | 1 | F | F | F | F | oligosaccharide biosynthetic process | LALBA\_BOVIN | LALBA\_CAPHI | LALBA\_CAVPO | LALBA\_PAPCY | GO:0007399 | 0.00434512725015518 | 14/3222 | 4/552 | 0.20747622847229 | 1 | F | F | F | F | nervous system development | ANAG\_HUMAN | PPT1\_BOVIN | PPT1\_HUMAN | TPP1\_HUMAN | GO:0002702 | 0.00434512725015518 | 14/3222 | 4/552 | 0.20747622847229 | 1 | F | F | F | F | positive regulation of production of molecular mediator of immune response | GRASS\_DROME | KLK7\_HUMAN | KLK7\_MOUSE | PGPSA\_DROME | GO:0046351 | 0.00434512725015518 | 14/3222 | 4/552 | 0.20747622847229 | 1 | F | F | F | F | disaccharide biosynthetic process | LALBA\_BOVIN | LALBA\_CAPHI | LALBA\_CAVPO | LALBA\_PAPCY | GO:0042325 | 0.0136561142147734 | 44/3222 | 10/552 | 0.209795565295532 | 1 | F | F | F | F | regulation of phosphorylation | ANG2\_MOUSE | ANG3\_MOUSE | ANG4\_MOUSE | ANGI\_MOUSE | ENPP2\_HUMAN | ENPP2\_RAT | GBA1\_HUMAN | PA21B\_PIG | PGH2\_HUMAN | Q5WRG2\_RAT | GO:0090066 | 0.00589695841092489 | 19/3222 | 5/552 | 0.214915487732377 | 1 | F | F | F | F | regulation of anatomical structure size | DOPO\_HUMAN | NP1\_RHOPR | NP2\_RHOPR | NP4\_RHOPR | PGH2\_HUMAN | GO:0043549 | 0.00900062073246431 | 29/3222 | 7/552 | 0.217194107877964 | 1 | F | F | F | F | regulation of kinase activity | ANG2\_MOUSE | ANG3\_MOUSE | ANG4\_MOUSE | ANGI\_MOUSE | GBA1\_HUMAN | PA21B\_PIG | Q5WRG2\_RAT | GO:0045017 | 0.00900062073246431 | 29/3222 | 7/552 | 0.217194107877964 | 1 | F | F | F | F | glycerolipid biosynthetic process | ANG1\_BOVIN | ANG2\_MOUSE | ANG3\_MOUSE | ANG4\_MOUSE | ANGI\_MOUSE | PAG15\_HUMAN | Q5WRG2\_RAT | GO:0016137 | 0.0074487895716946 | 24/3222 | 6/552 | 0.217366648814959 | 1 | F | F | F | F | glycoside metabolic process | AGAL\_HUMAN | BGLR\_HUMAN | FUCO\_HUMAN | GBA1\_HUMAN | NAGAB\_CHICK | NAGAB\_HUMAN | GO:0045321 | 0.0074487895716946 | 24/3222 | 6/552 | 0.217366648814959 | 1 | F | F | F | F | leukocyte activation | ANAG\_HUMAN | DNAS1\_HUMAN | DNSL3\_HUMAN | GBA1\_HUMAN | LICH\_HUMAN | PA2GX\_HUMAN | GO:0007610 | 0.0139664804469274 | 45/3222 | 10/552 | 0.231347718858636 | 1 | F | F | F | F | behavior | ANAG\_HUMAN | DOPO\_HUMAN | EST6\_DROME | G3I1H5\_CRIGR | GBA1\_HUMAN | KLK8\_MOUSE | LGMN\_MOUSE | PGH2\_HUMAN | PPT1\_HUMAN | RENI\_RAT | GO:0071840 | 0.0751086281812539 | 242/3222 | 46/552 | 0.234318622817251 | 1 | F | F | F | F | cellular component organization or biogenesis | A0A6M9BP13\_9EURO | A1E266\_9PEZI | ADPG2\_ARATH | ANAG\_HUMAN | ANG1\_BOVIN | ANGI\_MOUSE | CARP1\_CANAL | CATD\_RAT | CDA\_COLLN | CDA\_EMENI | ENG1\_RHIMI | EXG1\_CANAL | EXG1\_YEAST | G3I1H5\_CRIGR | G3YAL0\_ASPNA | GANA\_EMENI | GBA1\_HUMAN | KLK7\_HUMAN | KLK8\_MOUSE | LGMN\_MOUSE | LICH\_HUMAN | MMP1\_PIG | P79074\_9AGAR | PA2GA\_HUMAN | PA2GE\_HUMAN | PA2GX\_HUMAN | PELA\_ASPNG | PELB\_ASPNG | PGLR1\_ASPAC | PGLR1\_ASPNG | PGLR\_GIBFU | PME\_DAUCA | PME\_SITOR | PPAP\_RAT | PPT1\_BOVIN | PPT1\_HUMAN | PRTN3\_HUMAN | Q07524\_TROMA | Q5WRG2\_RAT | Q9LYJ5\_ARATH | RGLA\_ASPAC | RHGA\_ASPAC | TPP1\_HUMAN | TTHY\_CHICK | XGHA\_ASPTU | XTH34\_POPPZ | GO:0042330 | 0.00465549348230913 | 15/3222 | 4/552 | 0.247203094099091 | 1 | F | F | F | F | taxis | ECP\_HUMAN | ENPP2\_HUMAN | ENPP2\_RAT | PA21B\_PIG | GO:0007162 | 0.00465549348230913 | 15/3222 | 4/552 | 0.247203094099091 | 1 | F | F | F | F | negative regulation of cell adhesion | ENPP2\_RAT | PA2GA\_HUMAN | PA2GE\_HUMAN | PPA5\_RAT | GO:0048872 | 0.00465549348230913 | 15/3222 | 4/552 | 0.247203094099091 | 1 | F | F | F | F | homeostasis of number of cells | GBA1\_HUMAN | LICH\_HUMAN | PA2GA\_HUMAN | PA2GX\_HUMAN | GO:0019321 | 0.00775915580384854 | 25/3222 | 6/552 | 0.247667869245592 | 1 | F | F | F | F | pentose metabolic process | A0A059U759\_9PEZI | A0A2H5BN17\_TALPI | A8NI40\_COPC7 | ABFB\_ASPKW | AXHA2\_EMENI | G2QVH2\_THETT | GO:0003013 | 0.00620732464307883 | 20/3222 | 5/552 | 0.249105576298127 | 1 | F | F | F | F | circulatory system process | DOPO\_HUMAN | NP1\_RHOPR | NP2\_RHOPR | NP4\_RHOPR | PGH2\_HUMAN | GO:0050727 | 0.0127250155183116 | 41/3222 | 9/552 | 0.260081185444366 | 1 | F | F | F | F | regulation of inflammatory response | AOAH\_MOUSE | DNAS1\_HUMAN | DNSL3\_HUMAN | GBA1\_HUMAN | PA2GA\_HUMAN | PA2GX\_HUMAN | PGH2\_HUMAN | PPA5\_HUMAN | PPA5\_RAT | GO:0009892 | 0.0518311607697083 | 167/3222 | 32/552 | 0.26688639591306 | 1 | F | F | F | F | negative regulation of metabolic process | AGAL\_HUMAN | ANG1\_BOVIN | ANG3\_MOUSE | ANGI\_MOUSE | E0CX04\_MOMBA | G3I1H5\_CRIGR | GBA1\_HUMAN | GRAA\_HUMAN | GRAC\_MOUSE | LGMN\_MOUSE | O04358\_IRIHO | PA2GX\_HUMAN | PGH2\_HUMAN | PGRP1\_CAMDR | PPA5\_HUMAN | PPA5\_RAT | Q2QEH4\_SAPOF | Q94BW3\_CINCA | RIP0\_DIACA | RIP1\_BRYDI | RIP1\_HORVU | RIP1\_MOMCH | RIP1\_PHYAM | RIP2\_PHYAM | RIP3\_MOMCH | RIPA\_PHYAM | RIPG\_SURMU | RIPL1\_PHYDI | RIPL2\_PHYDI | RIPT\_TRIKI | TRFL\_BUBBU | TRFL\_HORSE | GO:0051050 | 0.016139044072005 | 52/3222 | 11/552 | 0.269010977848673 | 1 | F | F | F | F | positive regulation of transport | ANG1\_BOVIN | ANG2\_MOUSE | ANG3\_MOUSE | ANG4\_MOUSE | ANGI\_MOUSE | PA21B\_PIG | PA2GX\_HUMAN | PGH2\_HUMAN | PPT1\_BOVIN | PPT1\_HUMAN | Q5WRG2\_RAT | GO:0010815 | 0.00186219739292365 | 6/3222 | 2/552 | 0.274425740883015 | 1 | F | F | F | F | bradykinin catabolic process | CATH\_HUMAN | CBPN\_HUMAN | GO:0030098 | 0.00186219739292365 | 6/3222 | 2/552 | 0.274425740883015 | 1 | F | F | F | F | lymphocyte differentiation | GBA1\_HUMAN | LICH\_HUMAN | GO:0019915 | 0.00186219739292365 | 6/3222 | 2/552 | 0.274425740883015 | 1 | F | F | F | F | lipid storage | GBA1\_HUMAN | LICH\_HUMAN | GO:0042546 | 0.00186219739292365 | 6/3222 | 2/552 | 0.274425740883015 | 1 | F | F | F | F | cell wall biogenesis | Q07524\_TROMA | XTH34\_POPPZ | GO:0097529 | 0.00186219739292365 | 6/3222 | 2/552 | 0.274425740883015 | 1 | F | F | F | F | myeloid leukocyte migration | PA21B\_PIG | PRTN3\_HUMAN | GO:0005983 | 0.00186219739292365 | 6/3222 | 2/552 | 0.274425740883015 | 1 | F | F | F | F | starch catabolic process | AMY1\_HORVU | AMY1\_ORYSJ | GO:0010001 | 0.00186219739292365 | 6/3222 | 2/552 | 0.274425740883015 | 1 | F | F | F | F | glial cell differentiation | ANAG\_HUMAN | GBA1\_HUMAN | GO:0002698 | 0.00186219739292365 | 6/3222 | 2/552 | 0.274425740883015 | 1 | F | F | F | F | negative regulation of immune effector process | PPA5\_HUMAN | PPA5\_RAT | GO:0032722 | 0.00186219739292365 | 6/3222 | 2/552 | 0.274425740883015 | 1 | F | F | F | F | positive regulation of chemokine production | CHIA\_HUMAN | CHIL3\_MOUSE | GO:0046949 | 0.00186219739292365 | 6/3222 | 2/552 | 0.274425740883015 | 1 | F | F | F | F | fatty-acyl-CoA biosynthetic process | PPT1\_HUMAN | PPT2\_HUMAN | GO:0002764 | 0.00186219739292365 | 6/3222 | 2/552 | 0.274425740883015 | 1 | F | F | F | F | immune response-regulating signaling pathway | ANAG\_HUMAN | CATH\_HUMAN | GO:0019371 | 0.00186219739292365 | 6/3222 | 2/552 | 0.274425740883015 | 1 | F | F | F | F | cyclooxygenase pathway | PGH2\_HUMAN | PTGDS\_HUMAN | GO:0002703 | 0.00186219739292365 | 6/3222 | 2/552 | 0.274425740883015 | 1 | F | F | F | F | regulation of leukocyte mediated immunity | DNAS1\_HUMAN | DNSL3\_HUMAN | GO:0048588 | 0.00186219739292365 | 6/3222 | 2/552 | 0.274425740883015 | 1 | F | F | F | F | developmental cell growth | ANG2\_MOUSE | TTHY\_CHICK | GO:0140962 | 0.00186219739292365 | 6/3222 | 2/552 | 0.274425740883015 | 1 | F | F | F | F | multicellular organismal-level chemical homeostasis | ANAG\_HUMAN | CATH\_HUMAN | GO:0050731 | 0.00186219739292365 | 6/3222 | 2/552 | 0.274425740883015 | 1 | F | F | F | F | positive regulation of peptidyl-tyrosine phosphorylation | ENPP2\_HUMAN | ENPP2\_RAT | GO:0030641 | 0.00186219739292365 | 6/3222 | 2/552 | 0.274425740883015 | 1 | F | F | F | F | regulation of cellular pH | PPT1\_BOVIN | PPT1\_HUMAN | GO:0030217 | 0.00186219739292365 | 6/3222 | 2/552 | 0.274425740883015 | 1 | F | F | F | F | T cell differentiation | GBA1\_HUMAN | LICH\_HUMAN | GO:0030278 | 0.00186219739292365 | 6/3222 | 2/552 | 0.274425740883015 | 1 | F | F | F | F | regulation of ossification | TRFL\_BUBBU | TRFL\_HORSE | GO:0071675 | 0.00186219739292365 | 6/3222 | 2/552 | 0.274425740883015 | 1 | F | F | F | F | regulation of mononuclear cell migration | G3I1H5\_CRIGR | LGMN\_MOUSE | GO:0002687 | 0.00186219739292365 | 6/3222 | 2/552 | 0.274425740883015 | 1 | F | F | F | F | positive regulation of leukocyte migration | G3I1H5\_CRIGR | LGMN\_MOUSE | GO:0031399 | 0.0145872129112353 | 47/3222 | 10/552 | 0.276730449509462 | 1 | F | F | F | F | regulation of protein modification process | ANG2\_MOUSE | ANG3\_MOUSE | ANG4\_MOUSE | ANGI\_MOUSE | ENPP2\_HUMAN | ENPP2\_RAT | GBA1\_HUMAN | PA21B\_PIG | PGH2\_HUMAN | Q5WRG2\_RAT | GO:0001775 | 0.00806952203600248 | 26/3222 | 6/552 | 0.279075648164033 | 1 | F | F | F | F | cell activation | ANAG\_HUMAN | DNAS1\_HUMAN | DNSL3\_HUMAN | GBA1\_HUMAN | LICH\_HUMAN | PA2GX\_HUMAN | GO:0052547 | 0.00651769087523277 | 21/3222 | 5/552 | 0.284542289735633 | 1 | F | F | F | F | regulation of peptidase activity | CATD\_RAT | CATH\_HUMAN | G3I1H5\_CRIGR | LGMN\_MOUSE | PGH2\_HUMAN | GO:0043523 | 0.00651769087523277 | 21/3222 | 5/552 | 0.284542289735633 | 1 | F | F | F | F | regulation of neuron apoptotic process | G3I1H5\_CRIGR | GBA1\_HUMAN | LGMN\_MOUSE | PPT1\_BOVIN | PPT1\_HUMAN | GO:0009100 | 0.00651769087523277 | 21/3222 | 5/552 | 0.284542289735633 | 1 | F | F | F | F | glycoprotein metabolic process | ANAG\_HUMAN | BGLR\_HUMAN | HS3S1\_MOUSE | IDUA\_HUMAN | MANBA\_MOUSE | GO:0050776 | 0.0130353817504655 | 42/3222 | 9/552 | 0.284805593159119 | 1 | F | F | F | F | regulation of immune response | ANAG\_HUMAN | CATH\_HUMAN | CFAD\_MOUSE | GRASS\_DROME | KLK7\_HUMAN | KLK7\_MOUSE | LICH\_HUMAN | PA21B\_PIG | PGPSA\_DROME | GO:0045787 | 0.00341402855369336 | 11/3222 | 3/552 | 0.288046933634223 | 1 | F | F | F | F | positive regulation of cell cycle | G3I1H5\_CRIGR | HYAL1\_HUMAN | LGMN\_MOUSE | GO:0048167 | 0.00341402855369336 | 11/3222 | 3/552 | 0.288046933634223 | 1 | F | F | F | F | regulation of synaptic plasticity | G3I1H5\_CRIGR | LGMN\_MOUSE | PGH2\_HUMAN | GO:0043171 | 0.00341402855369336 | 11/3222 | 3/552 | 0.288046933634223 | 1 | F | F | F | F | peptide catabolic process | CATH\_HUMAN | CBPN\_HUMAN | TPP1\_HUMAN | GO:0030308 | 0.00341402855369336 | 11/3222 | 3/552 | 0.288046933634223 | 1 | F | F | F | F | negative regulation of cell growth | HYAL1\_HUMAN | PPT1\_BOVIN | PPT1\_HUMAN | GO:2000116 | 0.00496585971446307 | 16/3222 | 4/552 | 0.288376205925675 | 1 | F | F | F | F | regulation of cysteine-type endopeptidase activity | CATD\_RAT | G3I1H5\_CRIGR | LGMN\_MOUSE | PGH2\_HUMAN | GO:0040011 | 0.00496585971446307 | 16/3222 | 4/552 | 0.288376205925675 | 1 | F | F | F | F | locomotion | ECP\_HUMAN | ENPP2\_HUMAN | ENPP2\_RAT | PA21B\_PIG | GO:0009615 | 0.00993171942892613 | 32/3222 | 7/552 | 0.302099283515566 | 1 | F | F | F | F | response to virus | HYAL1\_HUMAN | PA2GX\_HUMAN | RIP1\_PHYAM | RIP2\_PHYAM | RIPA\_PHYAM | RNAS6\_HUMAN | TTHY\_CHICK | GO:0030155 | 0.00993171942892613 | 32/3222 | 7/552 | 0.302099283515566 | 1 | F | F | F | F | regulation of cell adhesion | CEL2A\_PIG | ENPP2\_RAT | HYAL1\_HUMAN | OXLA\_BOTAT | PA2GA\_HUMAN | PA2GE\_HUMAN | PPA5\_RAT | GO:0008219 | 0.0183116076970826 | 59/3222 | 12/552 | 0.303905232066715 | 1 | F | F | F | F | cell death | ATLE\_CYCAE | CHIA\_HUMAN | DNAS1\_HUMAN | DNSL3\_HUMAN | GBA1\_HUMAN | GRAA\_HUMAN | GRAC\_MOUSE | KLK8\_MOUSE | LICH\_HUMAN | OXLA\_BOTAT | OXLA\_CALRH | OXLA\_GLOHA | GO:0044093 | 0.0183116076970826 | 59/3222 | 12/552 | 0.303905232066715 | 1 | F | F | F | F | positive regulation of molecular function | ANG1\_BOVIN | ANG2\_MOUSE | ANG3\_MOUSE | ANG4\_MOUSE | ANGI\_MOUSE | CATD\_RAT | CATH\_HUMAN | G3I1H5\_CRIGR | LGMN\_MOUSE | PA21B\_PIG | PRTN3\_HUMAN | Q5WRG2\_RAT | GO:1903530 | 0.0133457479826195 | 43/3222 | 9/552 | 0.310132332448862 | 1 | F | F | F | F | regulation of secretion by cell | ANG1\_BOVIN | ANG2\_MOUSE | ANG3\_MOUSE | ANG4\_MOUSE | ANGI\_MOUSE | CEL2A\_PIG | PA2GX\_HUMAN | Q5WRG2\_RAT | RENI\_RAT | GO:0048731 | 0.00837988826815642 | 27/3222 | 6/552 | 0.311318272388462 | 1 | F | F | F | F | system development | ANAG\_HUMAN | ANGI\_MOUSE | PER53\_ARATH | PPT1\_BOVIN | PPT1\_HUMAN | TPP1\_HUMAN | GO:0051239 | 0.0496585971446307 | 160/3222 | 30/552 | 0.320517620979593 | 1 | F | F | F | F | regulation of multicellular organismal process | AGAL\_ORYSJ | CATH\_HUMAN | CEL2A\_PIG | CHIA\_HUMAN | CHIL3\_MOUSE | DOPO\_HUMAN | ENPP2\_HUMAN | ENPP2\_RAT | EST6\_DROME | G3I1H5\_CRIGR | GBA1\_HUMAN | HYAL1\_HUMAN | KLK8\_MOUSE | LGMN\_MOUSE | OXLA\_BOTAT | PA21B\_PIG | PA2GA\_HUMAN | PA2GE\_HUMAN | PA2GX\_HUMAN | PCP\_HUMAN | PGH2\_HUMAN | PGRP1\_CAMDR | PPA5\_HUMAN | PPA5\_RAT | PPAP\_RAT | PTGDS\_HUMAN | PTGDS\_MOUSE | RENI\_RAT | TRFL\_BUBBU | TRFL\_HORSE | GO:0140546 | 0.00682805710738672 | 22/3222 | 5/552 | 0.320836642359289 | 1 | F | F | F | F | defense response to symbiont | PA2GX\_HUMAN | RIP1\_PHYAM | RIP2\_PHYAM | RIPA\_PHYAM | RNAS6\_HUMAN | GO:0050728 | 0.00682805710738672 | 22/3222 | 5/552 | 0.320836642359289 | 1 | F | F | F | F | negative regulation of inflammatory response | AOAH\_MOUSE | GBA1\_HUMAN | PA2GX\_HUMAN | PPA5\_HUMAN | PPA5\_RAT | GO:0051607 | 0.00682805710738672 | 22/3222 | 5/552 | 0.320836642359289 | 1 | F | F | F | F | defense response to virus | PA2GX\_HUMAN | RIP1\_PHYAM | RIP2\_PHYAM | RIPA\_PHYAM | RNAS6\_HUMAN | GO:0032501 | 0.0673494723774053 | 217/3222 | 40/552 | 0.327219989742338 | 1 | F | F | F | F | multicellular organismal process | ANAG\_HUMAN | ANG2\_MOUSE | CATH\_HUMAN | CERU\_RAT | CHIA\_HUMAN | CHIL3\_MOUSE | CHIT\_PUNGR | CHYM\_CAMDR | DNAS1\_HUMAN | DNSL3\_HUMAN | DOPO\_HUMAN | EST6\_DROME | G3I1H5\_CRIGR | GBA1\_HUMAN | HE12\_DANRE | KLK8\_MOUSE | LGMN\_MOUSE | LICH\_HUMAN | LIPR2\_HUMAN | LIPR2\_RAT | LYSC2\_BOVIN | NP1\_RHOPR | NP2\_RHOPR | NP4\_RHOPR | PA2GA\_HUMAN | PA2GE\_HUMAN | PA2GX\_HUMAN | PCP\_HUMAN | PER53\_ARATH | PGH2\_HUMAN | PPA5\_HUMAN | PPA5\_PIG | PPA5\_RAT | PPT1\_HUMAN | RENI\_RAT | TPP1\_HUMAN | TRFL\_BUBBU | TRFL\_HORSE | TRY1\_GADMO | TRY3\_SALSA | GO:0071363 | 0.0037243947858473 | 12/3222 | 3/552 | 0.339092245407707 | 1 | F | F | F | F | cellular response to growth factor stimulus | G3I1H5\_CRIGR | HYAL1\_HUMAN | LGMN\_MOUSE | GO:0048145 | 0.0037243947858473 | 12/3222 | 3/552 | 0.339092245407707 | 1 | F | F | F | F | regulation of fibroblast proliferation | GILT\_MOUSE | PA21B\_BOVIN | PA21B\_PIG | GO:0051606 | 0.0037243947858473 | 12/3222 | 3/552 | 0.339092245407707 | 1 | F | F | F | F | detection of stimulus | CAH6\_HUMAN | L8ICE9\_9CETA | PERL\_CAPHI | GO:0071695 | 0.0037243947858473 | 12/3222 | 3/552 | 0.339092245407707 | 1 | F | F | F | F | anatomical structure maturation | GBA1\_HUMAN | PA2GX\_HUMAN | RENI\_RAT | GO:2000378 | 0.00217256362507759 | 7/3222 | 2/552 | 0.343310714768901 | 1 | F | F | F | F | negative regulation of reactive oxygen species metabolic process | PPA5\_HUMAN | PPA5\_RAT | GO:0042752 | 0.00217256362507759 | 7/3222 | 2/552 | 0.343310714768901 | 1 | F | F | F | F | regulation of circadian rhythm | PTGDS\_HUMAN | PTGDS\_MOUSE | GO:0006887 | 0.00217256362507759 | 7/3222 | 2/552 | 0.343310714768901 | 1 | F | F | F | F | exocytosis | PTGDS\_HUMAN | PTGDS\_MOUSE | GO:0031346 | 0.00217256362507759 | 7/3222 | 2/552 | 0.343310714768901 | 1 | F | F | F | F | positive regulation of cell projection organization | ENPP2\_HUMAN | ENPP2\_RAT | GO:0002366 | 0.00217256362507759 | 7/3222 | 2/552 | 0.343310714768901 | 1 | F | F | F | F | leukocyte activation involved in immune response | DNAS1\_HUMAN | DNSL3\_HUMAN | GO:0098734 | 0.00217256362507759 | 7/3222 | 2/552 | 0.343310714768901 | 1 | F | F | F | F | macromolecule depalmitoylation | PPT1\_BOVIN | PPT1\_HUMAN | GO:0051250 | 0.00217256362507759 | 7/3222 | 2/552 | 0.343310714768901 | 1 | F | F | F | F | negative regulation of lymphocyte activation | PA2GA\_HUMAN | PA2GE\_HUMAN | GO:0046688 | 0.00217256362507759 | 7/3222 | 2/552 | 0.343310714768901 | 1 | F | F | F | F | response to copper ion | AOC1\_HUMAN | CERU\_RAT | GO:0050868 | 0.00217256362507759 | 7/3222 | 2/552 | 0.343310714768901 | 1 | F | F | F | F | negative regulation of T cell activation | PA2GA\_HUMAN | PA2GE\_HUMAN | GO:0048666 | 0.00217256362507759 | 7/3222 | 2/552 | 0.343310714768901 | 1 | F | F | F | F | neuron development | ANAG\_HUMAN | PPT1\_HUMAN | GO:0002084 | 0.00217256362507759 | 7/3222 | 2/552 | 0.343310714768901 | 1 | F | F | F | F | protein depalmitoylation | PPT1\_BOVIN | PPT1\_HUMAN | GO:0048146 | 0.00217256362507759 | 7/3222 | 2/552 | 0.343310714768901 | 1 | F | F | F | F | positive regulation of fibroblast proliferation | PA21B\_BOVIN | PA21B\_PIG | GO:0007417 | 0.00217256362507759 | 7/3222 | 2/552 | 0.343310714768901 | 1 | F | F | F | F | central nervous system development | ANGI\_MOUSE | TPP1\_HUMAN | GO:0002263 | 0.00217256362507759 | 7/3222 | 2/552 | 0.343310714768901 | 1 | F | F | F | F | cell activation involved in immune response | DNAS1\_HUMAN | DNSL3\_HUMAN | GO:0032102 | 0.0121042830540037 | 39/3222 | 8/552 | 0.348373570849356 | 1 | F | F | F | F | negative regulation of response to external stimulus | AOAH\_MOUSE | GBA1\_HUMAN | KLK8\_MOUSE | PA2GX\_HUMAN | PPA5\_HUMAN | PPA5\_RAT | TRFL\_BUBBU | TRFL\_HORSE | GO:0051338 | 0.010552451893234 | 34/3222 | 7/552 | 0.362030321822053 | 1 | F | F | F | F | regulation of transferase activity | ANG2\_MOUSE | ANG3\_MOUSE | ANG4\_MOUSE | ANGI\_MOUSE | GBA1\_HUMAN | PA21B\_PIG | Q5WRG2\_RAT | GO:0046457 | 0.00558659217877095 | 18/3222 | 4/552 | 0.372681796332364 | 1 | F | F | F | F | prostanoid biosynthetic process | PA2GX\_HUMAN | PGH2\_HUMAN | PTGDS\_HUMAN | PTGDS\_MOUSE | GO:0001516 | 0.00558659217877095 | 18/3222 | 4/552 | 0.372681796332364 | 1 | F | F | F | F | prostaglandin biosynthetic process | PA2GX\_HUMAN | PGH2\_HUMAN | PTGDS\_HUMAN | PTGDS\_MOUSE | GO:1902075 | 0.00558659217877095 | 18/3222 | 4/552 | 0.372681796332364 | 1 | F | F | F | F | cellular response to salt | AOC1\_HUMAN | G3I1H5\_CRIGR | LGMN\_MOUSE | PGH2\_HUMAN | GO:0002684 | 0.015828677839851 | 51/3222 | 10/552 | 0.373746152445237 | 1 | F | F | F | F | positive regulation of immune system process | ANAG\_HUMAN | CFAD\_MOUSE | G3I1H5\_CRIGR | GRASS\_DROME | KLK7\_HUMAN | KLK7\_MOUSE | LGMN\_MOUSE | LICH\_HUMAN | PA21B\_PIG | PGPSA\_DROME | GO:0060255 | 0.0810055865921788 | 261/3222 | 47/552 | 0.374472525881073 | 1 | F | F | F | F | regulation of macromolecule metabolic process | ANG1\_BOVIN | ANG2\_MOUSE | ANG3\_MOUSE | ANG4\_MOUSE | ANGI\_MOUSE | CATD\_RAT | CATH\_HUMAN | CHIA\_HUMAN | CHIL3\_MOUSE | D6XHE1\_TRYB2 | E0CX04\_MOMBA | ENPP2\_HUMAN | ENPP2\_RAT | G3I1H5\_CRIGR | GBA1\_HUMAN | GRAA\_HUMAN | GRAC\_MOUSE | GRASS\_DROME | KLK7\_HUMAN | KLK7\_MOUSE | LGMN\_MOUSE | O04358\_IRIHO | PA21B\_PIG | PA2GX\_HUMAN | PGH2\_HUMAN | PGPSA\_DROME | PGRP1\_CAMDR | PPA5\_HUMAN | PPA5\_RAT | Q2QEH4\_SAPOF | Q5WRG2\_RAT | Q6R7Z5\_9TRYP | Q94BW3\_CINCA | RIP0\_DIACA | RIP1\_BRYDI | RIP1\_HORVU | RIP1\_MOMCH | RIP1\_PHYAM | RIP2\_PHYAM | RIP3\_MOMCH | RIPA\_PHYAM | RIPG\_SURMU | RIPL1\_PHYDI | RIPL2\_PHYDI | RIPT\_TRIKI | TRFL\_BUBBU | TRFL\_HORSE | GO:0065009 | 0.031657355679702 | 102/3222 | 19/552 | 0.382492915188876 | 1 | F | F | F | F | regulation of molecular function | AGAL\_HUMAN | ANG1\_BOVIN | ANG2\_MOUSE | ANG3\_MOUSE | ANG4\_MOUSE | ANGI\_MOUSE | AOCX\_BOVIN | CATD\_RAT | CATH\_HUMAN | G3I1H5\_CRIGR | GBA1\_HUMAN | GRAA\_HUMAN | LGMN\_MOUSE | PA21B\_PIG | PA2GX\_HUMAN | PGH2\_HUMAN | PPT1\_HUMAN | PRTN3\_HUMAN | Q5WRG2\_RAT | GO:0012501 | 0.0176908752327747 | 57/3222 | 11/552 | 0.383871275580383 | 1 | F | F | F | F | programmed cell death | ATLE\_CYCAE | CHIA\_HUMAN | DNAS1\_HUMAN | DNSL3\_HUMAN | GBA1\_HUMAN | GRAA\_HUMAN | GRAC\_MOUSE | LICH\_HUMAN | OXLA\_BOTAT | OXLA\_CALRH | OXLA\_GLOHA | GO:0043161 | 0.00403476101800124 | 13/3222 | 3/552 | 0.38987040414748 | 1 | F | F | F | F | proteasome-mediated ubiquitin-dependent protein catabolic process | ANAG\_HUMAN | GBA1\_HUMAN | MAN12\_PENCI | GO:0007346 | 0.00403476101800124 | 13/3222 | 3/552 | 0.38987040414748 | 1 | F | F | F | F | regulation of mitotic cell cycle | G3I1H5\_CRIGR | HYAL1\_HUMAN | LGMN\_MOUSE | GO:0046677 | 0.00403476101800124 | 13/3222 | 3/552 | 0.38987040414748 | 1 | F | F | F | F | response to antibiotic | AOC1\_HUMAN | AOCX\_BOVIN | HYAL1\_HUMAN | GO:0010498 | 0.00403476101800124 | 13/3222 | 3/552 | 0.38987040414748 | 1 | F | F | F | F | proteasomal protein catabolic process | ANAG\_HUMAN | GBA1\_HUMAN | MAN12\_PENCI | GO:0031344 | 0.00403476101800124 | 13/3222 | 3/552 | 0.38987040414748 | 1 | F | F | F | F | regulation of cell projection organization | ENPP2\_HUMAN | ENPP2\_RAT | KLK8\_MOUSE | GO:0007015 | 0.00403476101800124 | 13/3222 | 3/552 | 0.38987040414748 | 1 | F | F | F | F | actin filament organization | ANG1\_BOVIN | ANGI\_MOUSE | Q5WRG2\_RAT | GO:0120035 | 0.00403476101800124 | 13/3222 | 3/552 | 0.38987040414748 | 1 | F | F | F | F | regulation of plasma membrane bounded cell projection organization | ENPP2\_HUMAN | ENPP2\_RAT | KLK8\_MOUSE | GO:0006401 | 0.0074487895716946 | 24/3222 | 5/552 | 0.39452076752962 | 1 | F | F | F | F | RNA catabolic process | ANG4\_MOUSE | ECP\_HUMAN | RNAS6\_HUMAN | RNLE\_SOLLC | RNT2\_HUMAN | GO:0050730 | 0.00248292985723153 | 8/3222 | 2/552 | 0.409928091018004 | 1 | F | F | F | F | regulation of peptidyl-tyrosine phosphorylation | ENPP2\_HUMAN | ENPP2\_RAT | GO:0005982 | 0.00248292985723153 | 8/3222 | 2/552 | 0.409928091018004 | 1 | F | F | F | F | starch metabolic process | AMY1\_HORVU | AMY1\_ORYSJ | GO:0090322 | 0.00248292985723153 | 8/3222 | 2/552 | 0.409928091018004 | 1 | F | F | F | F | regulation of superoxide metabolic process | PPA5\_HUMAN | PPA5\_RAT | GO:0045807 | 0.00248292985723153 | 8/3222 | 2/552 | 0.409928091018004 | 1 | F | F | F | F | positive regulation of endocytosis | PPT1\_BOVIN | PPT1\_HUMAN | GO:0071277 | 0.00248292985723153 | 8/3222 | 2/552 | 0.409928091018004 | 1 | F | F | F | F | cellular response to calcium ion | G3I1H5\_CRIGR | LGMN\_MOUSE | GO:0022604 | 0.00248292985723153 | 8/3222 | 2/552 | 0.409928091018004 | 1 | F | F | F | F | regulation of cell morphogenesis | ENPP2\_HUMAN | ENPP2\_RAT | GO:0035821 | 0.00248292985723153 | 8/3222 | 2/552 | 0.409928091018004 | 1 | F | F | F | F | modulation of process of another organism | CARP1\_CANAL | OXLA\_BOTAT | GO:0031664 | 0.00248292985723153 | 8/3222 | 2/552 | 0.409928091018004 | 1 | F | F | F | F | regulation of lipopolysaccharide-mediated signaling pathway | TRFL\_BUBBU | TRFL\_HORSE | GO:0016049 | 0.00248292985723153 | 8/3222 | 2/552 | 0.409928091018004 | 1 | F | F | F | F | cell growth | ANG2\_MOUSE | TTHY\_CHICK | GO:0018158 | 0.00248292985723153 | 8/3222 | 2/552 | 0.409928091018004 | 1 | F | F | F | F | protein oxidation | ANAG\_HUMAN | Q5B038\_EMENI | GO:0051592 | 0.00248292985723153 | 8/3222 | 2/552 | 0.409928091018004 | 1 | F | F | F | F | response to calcium ion | G3I1H5\_CRIGR | LGMN\_MOUSE | GO:0042129 | 0.00248292985723153 | 8/3222 | 2/552 | 0.409928091018004 | 1 | F | F | F | F | regulation of T cell proliferation | PA2GA\_HUMAN | PA2GE\_HUMAN | GO:0051640 | 0.00248292985723153 | 8/3222 | 2/552 | 0.409928091018004 | 1 | F | F | F | F | organelle localization | PTGDS\_HUMAN | PTGDS\_MOUSE | GO:0006024 | 0.00248292985723153 | 8/3222 | 2/552 | 0.409928091018004 | 1 | F | F | F | F | glycosaminoglycan biosynthetic process | HS3S1\_MOUSE | HYAL1\_HUMAN | GO:0040014 | 0.00248292985723153 | 8/3222 | 2/552 | 0.409928091018004 | 1 | F | F | F | F | regulation of multicellular organism growth | G3I1H5\_CRIGR | LGMN\_MOUSE | GO:0002683 | 0.00931098696461825 | 30/3222 | 6/552 | 0.410408677219253 | 1 | F | F | F | F | negative regulation of immune system process | PA2GA\_HUMAN | PA2GE\_HUMAN | PPA5\_HUMAN | PPA5\_RAT | TRFL\_BUBBU | TRFL\_HORSE | GO:0044057 | 0.00931098696461825 | 30/3222 | 6/552 | 0.410408677219253 | 1 | F | F | F | F | regulation of system process | DOPO\_HUMAN | GBA1\_HUMAN | KLK8\_MOUSE | PGH2\_HUMAN | PPAP\_RAT | RENI\_RAT | GO:0002699 | 0.00589695841092489 | 19/3222 | 4/552 | 0.414752811958663 | 1 | F | F | F | F | positive regulation of immune effector process | GRASS\_DROME | KLK7\_HUMAN | KLK7\_MOUSE | PGPSA\_DROME | GO:0006915 | 0.0145872129112353 | 47/3222 | 9/552 | 0.41490267919851 | 1 | F | F | F | F | apoptotic process | ATLE\_CYCAE | CHIA\_HUMAN | DNAS1\_HUMAN | GBA1\_HUMAN | GRAA\_HUMAN | LICH\_HUMAN | OXLA\_BOTAT | OXLA\_CALRH | OXLA\_GLOHA | GO:0030334 | 0.0145872129112353 | 47/3222 | 9/552 | 0.41490267919851 | 1 | F | F | F | F | regulation of cell migration | CATH\_HUMAN | ENPP2\_HUMAN | ENPP2\_RAT | G3I1H5\_CRIGR | HYAL1\_HUMAN | LGMN\_MOUSE | PCP\_HUMAN | PGH2\_HUMAN | PPA5\_RAT | GO:0051046 | 0.0145872129112353 | 47/3222 | 9/552 | 0.41490267919851 | 1 | F | F | F | F | regulation of secretion | ANG1\_BOVIN | ANG2\_MOUSE | ANG3\_MOUSE | ANG4\_MOUSE | ANGI\_MOUSE | CEL2A\_PIG | PA2GX\_HUMAN | Q5WRG2\_RAT | RENI\_RAT | GO:0051173 | 0.0288640595903166 | 93/3222 | 17/552 | 0.426049643993958 | 1 | F | F | F | F | positive regulation of nitrogen compound metabolic process | ANG2\_MOUSE | ANG3\_MOUSE | ANG4\_MOUSE | ANGI\_MOUSE | CATD\_RAT | CATH\_HUMAN | ENPP2\_HUMAN | ENPP2\_RAT | G3I1H5\_CRIGR | GBA1\_HUMAN | GRASS\_DROME | LGMN\_MOUSE | PA21B\_PIG | PA2GX\_HUMAN | PGH2\_HUMAN | PGPSA\_DROME | Q5WRG2\_RAT | GO:0031326 | 0.0735567970204842 | 237/3222 | 42/552 | 0.429809313384605 | 1 | F | F | F | F | regulation of cellular biosynthetic process | AGAL\_HUMAN | ANG1\_BOVIN | ANG3\_MOUSE | ANGI\_MOUSE | CATH\_HUMAN | CHIA\_HUMAN | CHIL3\_MOUSE | D6XHE1\_TRYB2 | E0CX04\_MOMBA | G3I1H5\_CRIGR | GBA1\_HUMAN | GRAC\_MOUSE | GRASS\_DROME | KLK7\_HUMAN | KLK7\_MOUSE | LGMN\_MOUSE | O04358\_IRIHO | PA21B\_PIG | PA2GX\_HUMAN | PGH2\_HUMAN | PGPSA\_DROME | PGRP1\_CAMDR | PPA5\_HUMAN | PPA5\_RAT | Q2QEH4\_SAPOF | Q5WRG2\_RAT | Q6R7Z5\_9TRYP | Q94BW3\_CINCA | RIP0\_DIACA | RIP1\_BRYDI | RIP1\_HORVU | RIP1\_MOMCH | RIP1\_PHYAM | RIP2\_PHYAM | RIP3\_MOMCH | RIPA\_PHYAM | RIPG\_SURMU | RIPL1\_PHYDI | RIPL2\_PHYDI | RIPT\_TRIKI | TRFL\_BUBBU | TRFL\_HORSE | GO:0048523 | 0.0825574177529485 | 266/3222 | 47/552 | 0.431394482978619 | 1 | F | F | F | F | negative regulation of cellular process | AGAL\_HUMAN | ANG1\_BOVIN | ANG2\_MOUSE | ANG3\_MOUSE | ANG4\_MOUSE | ANGI\_MOUSE | CATH\_HUMAN | E0CX04\_MOMBA | ENPP2\_RAT | G3I1H5\_CRIGR | GBA1\_HUMAN | GILT\_MOUSE | GRAA\_HUMAN | GRAC\_MOUSE | HYAL1\_HUMAN | KLK8\_MOUSE | LGMN\_MOUSE | O04358\_IRIHO | PA2GA\_HUMAN | PA2GE\_HUMAN | PA2GX\_HUMAN | PGH2\_HUMAN | PGRP1\_CAMDR | PPA5\_HUMAN | PPA5\_RAT | PPT1\_BOVIN | PPT1\_HUMAN | PRTN3\_HUMAN | PTGDS\_HUMAN | PTGDS\_MOUSE | Q2QEH4\_SAPOF | Q5WRG2\_RAT | Q94BW3\_CINCA | RIP0\_DIACA | RIP1\_BRYDI | RIP1\_HORVU | RIP1\_MOMCH | RIP1\_PHYAM | RIP2\_PHYAM | RIP3\_MOMCH | RIPA\_PHYAM | RIPG\_SURMU | RIPL1\_PHYDI | RIPL2\_PHYDI | RIPT\_TRIKI | TRFL\_BUBBU | TRFL\_HORSE | GO:0050877 | 0.0130353817504655 | 42/3222 | 8/552 | 0.432855282389959 | 1 | F | F | F | F | nervous system process | DOPO\_HUMAN | G3I1H5\_CRIGR | GBA1\_HUMAN | KLK8\_MOUSE | LGMN\_MOUSE | PGH2\_HUMAN | PPT1\_HUMAN | TPP1\_HUMAN | GO:0006641 | 0.00434512725015518 | 14/3222 | 3/552 | 0.439614270241243 | 1 | F | F | F | F | triglyceride metabolic process | LIPG\_HUMAN | LIPR2\_HUMAN | LIPR2\_RAT | GO:0030100 | 0.00434512725015518 | 14/3222 | 3/552 | 0.439614270241243 | 1 | F | F | F | F | regulation of endocytosis | PPT1\_BOVIN | PPT1\_HUMAN | PRTN3\_HUMAN | GO:0021700 | 0.00434512725015518 | 14/3222 | 3/552 | 0.439614270241243 | 1 | F | F | F | F | developmental maturation | GBA1\_HUMAN | PA2GX\_HUMAN | RENI\_RAT | GO:0070848 | 0.00434512725015518 | 14/3222 | 3/552 | 0.439614270241243 | 1 | F | F | F | F | response to growth factor | G3I1H5\_CRIGR | HYAL1\_HUMAN | LGMN\_MOUSE | GO:0031960 | 0.0114835505896958 | 37/3222 | 7/552 | 0.45299962432614 | 1 | F | F | F | F | response to corticosteroid | CBPN\_HUMAN | GBA1\_HUMAN | LALBA\_BOVIN | LIPR2\_RAT | PGH2\_HUMAN | PTGDS\_HUMAN | PTGDS\_MOUSE | GO:0080134 | 0.0328988206083178 | 106/3222 | 19/552 | 0.453917968559861 | 1 | F | F | F | F | regulation of response to stress | ANAG\_HUMAN | AOAH\_MOUSE | CARP1\_CANAL | DNAS1\_HUMAN | DNSL3\_HUMAN | GBA1\_HUMAN | GRASS\_DROME | KLK7\_HUMAN | KLK7\_MOUSE | KLK8\_MOUSE | PA2GA\_HUMAN | PA2GX\_HUMAN | PGH2\_HUMAN | PGPSA\_DROME | PPA5\_HUMAN | PPA5\_RAT | RIP0\_DIACA | RIP3\_MOMCH | RIPT\_TRIKI | GO:0052548 | 0.00620732464307883 | 20/3222 | 4/552 | 0.456164620873842 | 1 | F | F | F | F | regulation of endopeptidase activity | CATD\_RAT | G3I1H5\_CRIGR | LGMN\_MOUSE | PGH2\_HUMAN | GO:0055085 | 0.00620732464307883 | 20/3222 | 4/552 | 0.456164620873842 | 1 | F | F | F | F | transmembrane transport | CERU\_RAT | PPA5\_PIG | TRFL\_BUBBU | TRFL\_HORSE | GO:0046456 | 0.00806952203600248 | 26/3222 | 5/552 | 0.467462169228879 | 1 | F | F | F | F | icosanoid biosynthetic process | PA21B\_PIG | PA2GX\_HUMAN | PGH2\_HUMAN | PTGDS\_HUMAN | PTGDS\_MOUSE | GO:2000145 | 0.0152079453755431 | 49/3222 | 9/552 | 0.467628085379331 | 1 | F | F | F | F | regulation of cell motility | CATH\_HUMAN | ENPP2\_HUMAN | ENPP2\_RAT | G3I1H5\_CRIGR | HYAL1\_HUMAN | LGMN\_MOUSE | PCP\_HUMAN | PGH2\_HUMAN | PPA5\_RAT | GO:0040012 | 0.0152079453755431 | 49/3222 | 9/552 | 0.467628085379331 | 1 | F | F | F | F | regulation of locomotion | CATH\_HUMAN | ENPP2\_HUMAN | ENPP2\_RAT | G3I1H5\_CRIGR | HYAL1\_HUMAN | LGMN\_MOUSE | PCP\_HUMAN | PGH2\_HUMAN | PPA5\_RAT | GO:0003008 | 0.0242085661080074 | 78/3222 | 14/552 | 0.470689376350232 | 1 | F | F | F | F | system process | ANG2\_MOUSE | DOPO\_HUMAN | G3I1H5\_CRIGR | GBA1\_HUMAN | KLK8\_MOUSE | LGMN\_MOUSE | LICH\_HUMAN | NP1\_RHOPR | NP2\_RHOPR | NP4\_RHOPR | PGH2\_HUMAN | PPT1\_HUMAN | RENI\_RAT | TPP1\_HUMAN | GO:0070664 | 0.00279329608938547 | 9/3222 | 2/552 | 0.473033679186644 | 1 | F | F | F | F | negative regulation of leukocyte proliferation | PA2GA\_HUMAN | PA2GE\_HUMAN | GO:0032652 | 0.00279329608938547 | 9/3222 | 2/552 | 0.473033679186644 | 1 | F | F | F | F | regulation of interleukin-1 production | PPA5\_HUMAN | PPA5\_RAT | GO:0002695 | 0.00279329608938547 | 9/3222 | 2/552 | 0.473033679186644 | 1 | F | F | F | F | negative regulation of leukocyte activation | PA2GA\_HUMAN | PA2GE\_HUMAN | GO:0051960 | 0.00279329608938547 | 9/3222 | 2/552 | 0.473033679186644 | 1 | F | F | F | F | regulation of nervous system development | ENPP2\_RAT | KLK8\_MOUSE | GO:0032642 | 0.00279329608938547 | 9/3222 | 2/552 | 0.473033679186644 | 1 | F | F | F | F | regulation of chemokine production | CHIA\_HUMAN | CHIL3\_MOUSE | GO:0043903 | 0.00279329608938547 | 9/3222 | 2/552 | 0.473033679186644 | 1 | F | F | F | F | regulation of biological process involved in symbiotic interaction | TRFL\_BUBBU | TRFL\_HORSE | GO:0032651 | 0.00279329608938547 | 9/3222 | 2/552 | 0.473033679186644 | 1 | F | F | F | F | regulation of interleukin-1 beta production | PPA5\_HUMAN | PPA5\_RAT | GO:0035337 | 0.00279329608938547 | 9/3222 | 2/552 | 0.473033679186644 | 1 | F | F | F | F | fatty-acyl-CoA metabolic process | PPT1\_HUMAN | PPT2\_HUMAN | GO:0046464 | 0.00279329608938547 | 9/3222 | 2/552 | 0.473033679186644 | 1 | F | F | F | F | acylglycerol catabolic process | LIPR2\_HUMAN | LIPR2\_RAT | GO:1903038 | 0.00279329608938547 | 9/3222 | 2/552 | 0.473033679186644 | 1 | F | F | F | F | negative regulation of leukocyte cell-cell adhesion | PA2GA\_HUMAN | PA2GE\_HUMAN | GO:0044703 | 0.00279329608938547 | 9/3222 | 2/552 | 0.473033679186644 | 1 | F | F | F | F | multi-organism reproductive process | CERU\_RAT | EST6\_DROME | GO:0007219 | 0.00279329608938547 | 9/3222 | 2/552 | 0.473033679186644 | 1 | F | F | F | F | Notch signaling pathway | CFAD\_MOUSE | OFUT1\_CAEEL | GO:0046461 | 0.00279329608938547 | 9/3222 | 2/552 | 0.473033679186644 | 1 | F | F | F | F | neutral lipid catabolic process | LIPR2\_HUMAN | LIPR2\_RAT | GO:0006023 | 0.00279329608938547 | 9/3222 | 2/552 | 0.473033679186644 | 1 | F | F | F | F | aminoglycan biosynthetic process | HS3S1\_MOUSE | HYAL1\_HUMAN | GO:2000242 | 0.00279329608938547 | 9/3222 | 2/552 | 0.473033679186644 | 1 | F | F | F | F | negative regulation of reproductive process | PTGDS\_HUMAN | PTGDS\_MOUSE | GO:0030182 | 0.00279329608938547 | 9/3222 | 2/552 | 0.473033679186644 | 1 | F | F | F | F | neuron differentiation | ANAG\_HUMAN | GBA1\_HUMAN | GO:0010604 | 0.0369335816263191 | 119/3222 | 21/552 | 0.478785893230971 | 1 | F | F | F | F | positive regulation of macromolecule metabolic process | ANG2\_MOUSE | ANG3\_MOUSE | ANG4\_MOUSE | ANGI\_MOUSE | CATD\_RAT | CATH\_HUMAN | CHIA\_HUMAN | CHIL3\_MOUSE | ENPP2\_HUMAN | ENPP2\_RAT | G3I1H5\_CRIGR | GBA1\_HUMAN | GRASS\_DROME | KLK7\_HUMAN | KLK7\_MOUSE | LGMN\_MOUSE | PA21B\_PIG | PA2GX\_HUMAN | PGH2\_HUMAN | PGPSA\_DROME | Q5WRG2\_RAT | GO:0043065 | 0.0117939168218498 | 38/3222 | 7/552 | 0.482898148314519 | 1 | F | F | F | F | positive regulation of apoptotic process | ATLE\_CYCAE | CATD\_RAT | CATH\_HUMAN | GRAA\_HUMAN | PA21B\_BOVIN | PA21B\_PIG | PGH2\_HUMAN | GO:0071241 | 0.0117939168218498 | 38/3222 | 7/552 | 0.482898148314519 | 1 | F | F | F | F | cellular response to inorganic substance | AOC1\_HUMAN | DABA\_PSEMU | ENPP2\_RAT | G3I1H5\_CRIGR | LGMN\_MOUSE | PGH2\_HUMAN | Q96X16\_PICPA | GO:0002237 | 0.0117939168218498 | 38/3222 | 7/552 | 0.482898148314519 | 1 | F | F | F | F | response to molecule of bacterial origin | ANAG\_HUMAN | ANG4\_MOUSE | PGH2\_HUMAN | PGPSA\_DROME | PPA5\_HUMAN | PPA5\_RAT | RENI\_RAT | GO:0097435 | 0.00465549348230913 | 15/3222 | 3/552 | 0.487717064276533 | 1 | F | F | F | F | supramolecular fiber organization | ANG1\_BOVIN | ANGI\_MOUSE | Q5WRG2\_RAT | GO:0034644 | 0.00465549348230913 | 15/3222 | 3/552 | 0.487717064276533 | 1 | F | F | F | F | cellular response to UV | HYAL1\_HUMAN | MMP1\_PIG | PGH2\_HUMAN | GO:0045597 | 0.0102420856610801 | 33/3222 | 6/552 | 0.508044604992734 | 1 | F | F | F | F | positive regulation of cell differentiation | ENPP2\_RAT | PA2GA\_HUMAN | PA2GX\_HUMAN | PGH2\_HUMAN | TRFL\_BUBBU | TRFL\_HORSE | GO:0030162 | 0.0102420856610801 | 33/3222 | 6/552 | 0.508044604992734 | 1 | F | F | F | F | regulation of proteolysis | CATD\_RAT | CATH\_HUMAN | G3I1H5\_CRIGR | GBA1\_HUMAN | LGMN\_MOUSE | PGH2\_HUMAN | GO:0006954 | 0.0102420856610801 | 33/3222 | 6/552 | 0.508044604992734 | 1 | F | F | F | F | inflammatory response | CHIL3\_MOUSE | HYAL1\_HUMAN | PA2GA\_HUMAN | PA2GE\_HUMAN | PCP\_HUMAN | PGH2\_HUMAN | GO:0051130 | 0.0121042830540037 | 39/3222 | 7/552 | 0.512325399634428 | 1 | F | F | F | F | positive regulation of cellular component organization | ENPP2\_HUMAN | ENPP2\_RAT | GBA1\_HUMAN | HYAL1\_HUMAN | MMP1\_PIG | PPT1\_BOVIN | PPT1\_HUMAN | GO:1902105 | 0.00310366232153942 | 10/3222 | 2/552 | 0.531874779079226 | 1 | F | F | F | F | regulation of leukocyte differentiation | TRFL\_BUBBU | TRFL\_HORSE | GO:0044706 | 0.00310366232153942 | 10/3222 | 2/552 | 0.531874779079226 | 1 | F | F | F | F | multi-multicellular organism process | CERU\_RAT | EST6\_DROME | GO:0050866 | 0.00310366232153942 | 10/3222 | 2/552 | 0.531874779079226 | 1 | F | F | F | F | negative regulation of cell activation | PA2GA\_HUMAN | PA2GE\_HUMAN | GO:0035601 | 0.00310366232153942 | 10/3222 | 2/552 | 0.531874779079226 | 1 | F | F | F | F | protein deacylation | PPT1\_BOVIN | PPT1\_HUMAN | GO:0006885 | 0.00310366232153942 | 10/3222 | 2/552 | 0.531874779079226 | 1 | F | F | F | F | regulation of pH | PPT1\_BOVIN | PPT1\_HUMAN | GO:0002718 | 0.00310366232153942 | 10/3222 | 2/552 | 0.531874779079226 | 1 | F | F | F | F | regulation of cytokine production involved in immune response | PPA5\_HUMAN | PPA5\_RAT | GO:0006809 | 0.00310366232153942 | 10/3222 | 2/552 | 0.531874779079226 | 1 | F | F | F | F | nitric oxide biosynthetic process | PPA5\_HUMAN | PPA5\_RAT | GO:0070661 | 0.00310366232153942 | 10/3222 | 2/552 | 0.531874779079226 | 1 | F | F | F | F | leukocyte proliferation | GBA1\_HUMAN | LICH\_HUMAN | GO:0098732 | 0.00310366232153942 | 10/3222 | 2/552 | 0.531874779079226 | 1 | F | F | F | F | macromolecule deacylation | PPT1\_BOVIN | PPT1\_HUMAN | GO:0002253 | 0.00310366232153942 | 10/3222 | 2/552 | 0.531874779079226 | 1 | F | F | F | F | activation of immune response | ANAG\_HUMAN | CFAD\_MOUSE | GO:0034330 | 0.00310366232153942 | 10/3222 | 2/552 | 0.531874779079226 | 1 | F | F | F | F | cell junction organization | KLK8\_MOUSE | PRTN3\_HUMAN | GO:0022408 | 0.00310366232153942 | 10/3222 | 2/552 | 0.531874779079226 | 1 | F | F | F | F | negative regulation of cell-cell adhesion | PA2GA\_HUMAN | PA2GE\_HUMAN | GO:0044085 | 0.00310366232153942 | 10/3222 | 2/552 | 0.531874779079226 | 1 | F | F | F | F | cellular component biogenesis | Q07524\_TROMA | XTH34\_POPPZ | GO:0050729 | 0.00310366232153942 | 10/3222 | 2/552 | 0.531874779079226 | 1 | F | F | F | F | positive regulation of inflammatory response | PA2GA\_HUMAN | PGH2\_HUMAN | GO:0060429 | 0.00310366232153942 | 10/3222 | 2/552 | 0.531874779079226 | 1 | F | F | F | F | epithelium development | ANAG\_HUMAN | GBA1\_HUMAN | GO:0006511 | 0.00496585971446307 | 16/3222 | 3/552 | 0.533718800819278 | 1 | F | F | F | F | ubiquitin-dependent protein catabolic process | ANAG\_HUMAN | GBA1\_HUMAN | MAN12\_PENCI | GO:0043632 | 0.00496585971446307 | 16/3222 | 3/552 | 0.533718800819278 | 1 | F | F | F | F | modification-dependent macromolecule catabolic process | ANAG\_HUMAN | GBA1\_HUMAN | MAN12\_PENCI | GO:0071482 | 0.00496585971446307 | 16/3222 | 3/552 | 0.533718800819278 | 1 | F | F | F | F | cellular response to light stimulus | HYAL1\_HUMAN | MMP1\_PIG | PGH2\_HUMAN | GO:0019941 | 0.00496585971446307 | 16/3222 | 3/552 | 0.533718800819278 | 1 | F | F | F | F | modification-dependent protein catabolic process | ANAG\_HUMAN | GBA1\_HUMAN | MAN12\_PENCI | GO:0051341 | 0.00496585971446307 | 16/3222 | 3/552 | 0.533718800819278 | 1 | F | F | F | F | regulation of oxidoreductase activity | AGAL\_HUMAN | AOCX\_BOVIN | GRAA\_HUMAN | GO:0031348 | 0.00869025450031037 | 28/3222 | 5/552 | 0.537476143346797 | 1 | F | F | F | F | negative regulation of defense response | AOAH\_MOUSE | GBA1\_HUMAN | PA2GX\_HUMAN | PPA5\_HUMAN | PPA5\_RAT | GO:0044273 | 0.00869025450031037 | 28/3222 | 5/552 | 0.537476143346797 | 1 | F | F | F | F | sulfur compound catabolic process | ANAG\_HUMAN | BGLR\_HUMAN | HYAL1\_HUMAN | IDUA\_HUMAN | PERL\_CAPHI | GO:0051384 | 0.010552451893234 | 34/3222 | 6/552 | 0.539333379957837 | 1 | F | F | F | F | response to glucocorticoid | CBPN\_HUMAN | GBA1\_HUMAN | LIPR2\_RAT | PGH2\_HUMAN | PTGDS\_HUMAN | PTGDS\_MOUSE | GO:0051094 | 0.0180012414649286 | 58/3222 | 10/552 | 0.546303054637117 | 1 | F | F | F | F | positive regulation of developmental process | AGAL\_ORYSJ | CATH\_HUMAN | ENPP2\_HUMAN | ENPP2\_RAT | HYAL1\_HUMAN | PA2GA\_HUMAN | PA2GX\_HUMAN | PGH2\_HUMAN | TRFL\_BUBBU | TRFL\_HORSE | GO:0008150 | 0.999068901303538 | 3219/3222 | 552/552 | 0.568949248080686 | 1 | F | F | F | F | biological\_process | A0A059U759\_9PEZI | A0A068FT77\_9PEZI | A0A075B5H6\_TRIHA | A0A075C6T6\_RHIMI | A0A086SY89\_ACRC1 | A0A086T6R4\_ACRC1 | A0A087WNH2\_FICBE | A0A088T0J9\_GEOCN | A0A098DND1\_GIBZE | A0A0A0Y4H8\_TRAFO | A0A0J5Q413\_ASPFM | A0A0M3KKZ6\_RHIMI | A0A0M3KKZ8\_RHIMI | A0A0R3QSA7\_9BILA | A0A0R4I979\_BRABE | A0A0S2GKZ1\_9APHY | A0A173N065\_EISFE | A0A1L6CE30\_9EURO | A0A1L8D5Z7\_BOTAT | A0A1L9WG58\_ASPA1 | A0A1S4NYF8\_PANVG | A0A1S9DRB1\_ASPOZ | A0A2H5BN17\_TALPI | A0A2N1LTK3\_TRIHA | A0A2U8ZTY7\_RHIZD | A0A2Z4HIN9\_9EURO | A0A384E148\_NICBE | A0A3B6UEQ2\_RHIMI | A0A3B6UEQ6\_EISFE | A0A3G2C3I4\_9EURO | A0A3G4RHU4\_9PEZI | A0A3L6SKP5\_PANMI | A0A3S5H5N2\_LEIDO | A0A482LWB1\_OSTFU | A0A5J6BJN2\_MALCI | A0A6F8Z6Y2\_BOMMO | A0A6M9BP13\_9EURO | A0A6P6YAT6\_DERPT | A0A7S6G7I6\_9PEZI | A0A856TAI5\_9BASI | A0NFU8\_ANOGA | A1E266\_9PEZI | A1HA\_LOXIN | A1HB2\_LOXIN | A2QZC8\_ASPNC | A2TM14\_HEVBR | A311\_LOXLA | A4GX63\_TOXGO | A5AB48\_ASPNC | A6PZ97\_SALSA | A6YRT4\_9PEZI | A7KMF0\_9CAEN | A8NI40\_COPC7 | A8PUY1\_MALGO | A8PUY5\_MALGO | A9LI60\_BIOOC | A9ZSX9\_9BRYO | ABFB\_ASPKW | ADA2\_HUMAN | ADPG2\_ARATH | AGAL\_HUMAN | AGAL\_ORYSJ | AMY1A\_HUMAN | AMY1\_HORVU | AMY1\_ORYSJ | AMYA1\_ASPOR | AMYG\_SACFI | AMY\_ORYLA | ANAG\_HUMAN | ANG1\_BOVIN | ANG2\_MOUSE | ANG3\_MOUSE | ANG4\_MOUSE | ANGI\_MOUSE | AOAH\_MOUSE | AOC1\_HUMAN | AOCX\_BOVIN | APO1\_CYCAE | ASAH1\_BALAS | ASM3A\_HUMAN | ASM3A\_MOUSE | ATLE\_CYCAE | AXE1\_ASPAW | AXE2\_TALPU | AXHA2\_EMENI | B2ZGS7\_9ASPA | B4F320\_LIMPO | B7X9Z0\_COPCI | B7X9Z2\_COPCI | B9TU22\_GADMO | BGALA\_ASPNC | BGALA\_ASPOR | BGALA\_PENSQ | BGL1\_ASPAC | BGLA\_ASPFU | BGLA\_ASPOR | BGLR\_HUMAN | C3VEV9\_PENCN | C7YSL3\_FUSV7 | CAH6\_HUMAN | CARP1\_CANAL | CARP2\_CANAX | CARP\_RHIPU | CAT3\_NEUCR | CATD\_RAT | CATH\_HUMAN | CATLL\_FASHE | CBHB\_ASPFU | CBHRE\_GEOS1 | CBPA1\_PIG | CBPD\_LOPSP | CBPN\_HUMAN | CDA\_COLLN | CDA\_EMENI | CEL2A\_PIG | CERU\_RAT | CFAD\_MOUSE | CHI1\_COCPS | CHI2\_HORVU | CHI2\_ORYSJ | CHI33\_TRIHA | CHI42\_TRIHA | CHI4\_CRYJA | CHIA\_HUMAN | CHIC\_ARATH | CHIC\_SECCE | CHIL3\_MOUSE | CHIT\_PUNGR | CHLY\_HEVBR | CHYM\_CAMDR | CKX1\_MAIZE | COGS\_HYPLI | CONB\_CANEN | CUCM1\_CUCME | CUTI1\_ASPOR | CYP5\_CAEEL | CYSP\_BLOTA | D0QF43\_9HELO | D1M8S7\_HEVBR | D1MPT2\_ROYRE | D6XHE1\_TRYB2 | D9MWI4\_9ASPA | DABA\_PSEMU | DDN1\_BOVIN | DEXT\_TALMI | DIR\_GLYEC | DNAS1\_HUMAN | DNSL3\_HUMAN | DOPO\_HUMAN | DPP2\_HUMAN | E0A7J0\_YARLL | E0CX04\_MOMBA | E0XN39\_9EURO | E13B\_HORVU | E13C\_MUSAC | E3VTL0\_9ASPA | E5D0X5\_SCHOC | E9G5J5\_DAPPU | ECP\_HUMAN | EGFB2\_MOUSE | EGLB\_ASPNG | ENDO2\_ARATH | ENG1\_RHIMI | ENPP2\_HUMAN | ENPP2\_RAT | ERVB\_TABDI | EST6\_DROME | EXG1\_CANAL | EXG1\_YEAST | F0ZJZ1\_DICPU | F1CYZ0\_TALFU | F2Z7L1\_9ANNE | FAEA\_ASPNG | FAEB1\_ASPOR | FAEB2\_ASPOR | FUCO\_HUMAN | G0RVK1\_HYPJQ | G2Q665\_MYCTT | G2QVH2\_THETT | G2X3Y1\_VERDV | G3I1H5\_CRIGR | G3JPF7\_CORMM | G3YAL0\_ASPNA | G3YFQ1\_ASPNA | G8GLP2\_LENED | G9NTY1\_HYPAI | GANA\_ASPAC | GANA\_EMENI | GANA\_HUMIN | GBA1\_HUMAN | GCE2\_MYCTT | GCE\_CERUI | GCE\_HYPJQ | GGH\_HUMAN | GH7B\_LIMQU | GILT\_MOUSE | GOOX\_SARSR | GPX3\_HUMAN | GPX5\_HUMAN | GPX6\_MOUSE | GRAA\_HUMAN | GRAC\_MOUSE | GRAK\_HUMAN | GRASS\_DROME | GUB2\_HORVU | GUN2\_HYPJE | GUN6\_HUMIN | GUN7\_HYPJQ | GUNC\_FUSOX | GUN\_ASPAC | GUN\_CRYAT | GUN\_MYTED | GUX1\_HUMGT | GUX1\_HYPJE | GUX1\_TRIHA | GUX2\_HYPJE | H1AE14\_PHACH | HE12\_DANRE | HEXC\_OSTFU | HS3S1\_MOUSE | HYAL1\_HUMAN | I1SB18\_VIPAE | I2FI81\_EISFE | I3RY46\_TRIHA | IDH\_OSTTA | IDUA\_HUMAN | INU2\_ASPFI | INUE\_ASPAW | INV\_SCHOC | IPUA\_ASPNG | J7LCB0\_DEIAC | J9UN47\_GIBZA | K7CID1\_PANTR | K7N5L9\_RAPSA | K9L8F3\_MALCI | KATG2\_MAGO7 | KLK10\_HUMAN | KLK1\_HUMAN | KLK2\_HORSE | KLK2\_HUMAN | KLK7\_HUMAN | KLK7\_MOUSE | KLK8\_MOUSE | L7SVX1\_RHIMI | L8ICE9\_9CETA | LAC1\_MELAO | LAC1\_TRAMX | LAC2\_TRAVE | LALBA\_BOVIN | LALBA\_CAPHI | LALBA\_CAVPO | LALBA\_PAPCY | LAPA\_ASPOR | LGMN\_MOUSE | LICH\_HUMAN | LIG2\_PHACH | LIG4\_PHACH | LIG8\_PHACH | LIP1\_DIURU | LIP2\_DIURU | LIP2\_GEOCN | LIP3\_DIURU | LIPA\_MOEAP | LIPB\_PSEA2 | LIPG\_CANLF | LIPG\_HUMAN | LIPP\_HORSE | LIPR1\_CANLF | LIPR1\_HUMAN | LIPR2\_HUMAN | LIPR2\_RAT | LIP\_THELA | LUCI\_OPLGR | LYG\_STRCA | LYS1\_MUSDO | LYSC1\_ANAPL | LYSC1\_CANLF | LYSC1\_HORSE | LYSC2\_BOVIN | LYSC2\_ONCMY | LYSC\_COTJA | LYSC\_EQUAS | LYSC\_NUMME | LYSC\_OPIHO | LYSC\_PELSI | LYS\_BOMMO | LYS\_RUDPH | M2RAI8\_CERS8 | M9TI89\_RHIPU | MAN12\_PENCI | MAN4\_SOLLC | MANA\_ASPNC | MANA\_CANEN | MANA\_CRYAT | MANA\_HYPJR | MANA\_MYTED | MANA\_PODAN | MANBA\_MOUSE | MCPT2\_RAT | MDLA\_PENCA | MDLA\_PENCY | MEL1\_YEASX | MMP1\_PIG | MNCO\_MICNN | MNLOX\_MAGO7 | NADA\_APLCA | NAGAB\_CHICK | NAGAB\_HUMAN | NANL\_MACDE | NCS\_THLFG | NP1\_RHOPR | NP2\_RHOPR | NP4\_RHOPR | NUP1\_PENCI | NUS1\_ASPOR | O00095\_HYPJE | O04358\_IRIHO | O22443\_SOYBN | O44049\_TRYRA | O74705\_ASPNG | O77044\_9NEOP | O81100\_SOLLC | O81226\_CARPA | O81934\_CANEN | O97389\_HELAM | OFUT1\_CAEEL | OXLA\_BOTAT | OXLA\_CALRH | OXLA\_GLOHA | P78583\_ASPOZ | P79074\_9AGAR | PA1\_VESBA | PA21B\_BOVIN | PA21B\_PIG | PA2A1\_BUNCE | PA2A1\_ECHCA | PA2A1\_NAJAT | PA2A1\_OPHHA | PA2A2\_NAJNA | PA2A2\_OPHHA | PA2A2\_TROCA | PA2A4\_NAJSG | PA2A5\_TRIST | PA2A7\_GLOHA | PA2A\_BOTJR | PA2A\_CROAT | PA2A\_DEIAC | PA2A\_GLOHA | PA2A\_NAJAT | PA2B1\_AGKPI | PA2B2\_BOTJR | PA2B2\_PROFL | PA2B3\_BOTAS | PA2B3\_BUNCE | PA2B5\_BUNCE | PA2B5\_NOTSC | PA2BA\_VIPAA | PA2BB\_GLOHA | PA2BB\_PSEAU | PA2BC\_VIPAA | PA2BD\_CRODU | PA2B\_BUNCE | PA2B\_NOTSC | PA2GA\_HUMAN | PA2GE\_HUMAN | PA2GX\_HUMAN | PA2H1\_AGKCL | PA2H1\_BOTBZ | PA2H1\_BOTJR | PA2H1\_BOTMO | PA2H1\_BOTPI | PA2H2\_BOTAS | PA2H2\_BOTMO | PA2H2\_BOTPI | PA2H2\_CERGO | PA2H3\_BOTPI | PA2HB\_AGKPI | PA2HB\_OXYSC | PA2HH\_TRIST | PA2HS\_ECHCA | PA2H\_BOTPA | PA2H\_DEIAC | PA2H\_PROMB | PA2N\_GLOHA | PA2\_APIME | PAG15\_HUMAN | PCP\_HUMAN | PDH1\_LEUMG | PELA\_ASPNG | PELB\_ASPNG | PEM1\_PHACH | PEPA\_ASPPH | PER1A\_ARMRU | PER1\_ARAHY | PER1\_SORBI | PER53\_ARATH | PER59\_ARATH | PERL\_BOVIN | PERL\_BUBBU | PERL\_CAPHI | PER\_ARTRA | PER\_COPCI | PGH2\_HUMAN | PGLR1\_ASPAC | PGLR1\_ASPNG | PGLR\_GIBFU | PGPSA\_DROME | PGRP1\_CAMDR | PHAZ\_TALFU | PLA22\_ORYSJ | PLY1\_JUNAS | PME\_DAUCA | PME\_SITOR | POXA\_DICDI | PPA5\_HUMAN | PPA5\_PIG | PPA5\_RAT | PPAF1\_HOLDI | PPAP\_RAT | PPT1\_BOVIN | PPT1\_HUMAN | PPT2\_HUMAN | PRS57\_HUMAN | PRTN3\_HUMAN | PTGDS\_HUMAN | PTGDS\_MOUSE | Q02321\_PHACH | Q06AK3\_TOXGO | Q07524\_TROMA | Q0KFV0\_SOLLC | Q12715\_HYPJE | Q2QEH4\_SAPOF | Q2U8V9\_ASPOR | Q2Z1W1\_PHACH | Q40069\_HORVU | Q43576\_TOBAC | Q4AE59\_OSTFU | Q4W6L6\_CYCRE | Q4WP32\_ASPFU | Q50KB2\_PHACH | Q55FE6\_DICDI | Q588B8\_CRYJA | Q5B038\_EMENI | Q5WRG2\_RAT | Q60FD2\_9APHY | Q69G21\_TENMO | Q6NY42\_DANRE | Q6R7Z5\_9TRYP | Q6VAY1\_9PEZI | Q6WER3\_GIBZA | Q6WSR8\_PICAB | Q70C53\_SOLTU | Q70SY0\_HYPJE | Q7LHI2\_PHACH | Q7LIJ0\_PHACH | Q7LST4\_PENEN | Q7RWP2\_NEUCR | Q7X9A9\_CAMSI | Q7YXL2\_TENMO | Q86RS6\_MANSE | Q8H0C9\_VIGUN | Q8J0K6\_MELAO | Q8J0K8\_MELAO | Q8NJY6\_9HYPO | Q8T0W7\_9NEOP | Q8TFL9\_TALEM | Q8TG26\_THEAU | Q8TGI8\_TALEM | Q92456\_HYPJE | Q92458\_HYPJE | Q93X60\_CICIN | Q94BW3\_CINCA | Q95KP4\_HORSE | Q95V66\_PENVA | Q96X16\_PICPA | Q9FUH3\_VIGUS | Q9LYJ5\_ARATH | Q9P8F7\_YARLL | Q9STC1\_GRALE | Q9XEI3\_HORVV | QPCT1\_DROME | QPCT2\_DROME | QPCT\_IXOSC | QPCT\_MOUSE | RENI\_RAT | RGLA\_ASPAC | RHGA\_ASPAC | RIP0\_DIACA | RIP1\_BRYDI | RIP1\_HORVU | RIP1\_MOMCH | RIP1\_PHYAM | RIP2\_PHYAM | RIP3\_MOMCH | RIPA\_PHYAM | RIPG\_SURMU | RIPL1\_PHYDI | RIPL2\_PHYDI | RIPT\_TRIKI | RNAS4\_HUMAN | RNAS4\_PIG | RNAS6\_HUMAN | RNLE\_SOLLC | RNS1B\_RAT | RNSL3\_DANRE | RNS\_BOVIN | RNT2\_HUMAN | S7Q6I2\_GLOTA | S7ZIW0\_PENO1 | SIA\_ASPFU | THCAS\_CANSA | TLP\_PRUAV | TPP1\_HUMAN | TRFL\_BUBBU | TRFL\_HORSE | TRY1\_GADMO | TRY3\_SALSA | TRYB2\_HUMAN | TTHY\_CHICK | TTHY\_MOUSE | TTHY\_RAT | V5NTD\_NAJAT | VM11\_BOTMO | VM12\_CROAD | VM1A3\_DEIAC | VM1BI\_BOTMO | VM1T1\_PROMU | VM1T2\_PROFL | VPL1\_PLEER | VPL2\_PLEER | VSPP\_DEIAC | VSPSX\_GLOSA | W0T408\_KLUMD | W4KMP1\_HETIT | W6Q990\_PENRF | W8P1L2\_TALEM | W8VR85\_TALPI | X0M5X0\_FUSOX | XGHA\_ASPTU | XTH34\_POPPZ | XYLA\_ASPNC | XYLO\_MYCTT | XYN1\_HYPJR | XYN2\_HYPJR | XYN3\_ASPKW | XYN3\_HYPJQ | XYNA\_FUSO4 | XYNA\_PENSI | XYNA\_THEAU | XYNA\_THELA | XYNC\_ASPNC | XYND\_EMENI | GO:0009059 | 0.0201738050900062 | 65/3222 | 11/552 | 0.570288548194993 | 1 | F | F | F | F | macromolecule biosynthetic process | ANG1\_BOVIN | ANG2\_MOUSE | ANG3\_MOUSE | ANG4\_MOUSE | ANGI\_MOUSE | HS3S1\_MOUSE | HYAL1\_HUMAN | LICH\_HUMAN | PTGDS\_HUMAN | PTGDS\_MOUSE | Q5WRG2\_RAT | GO:0051093 | 0.00900062073246431 | 29/3222 | 5/552 | 0.570858823607393 | 1 | F | F | F | F | negative regulation of developmental process | G3I1H5\_CRIGR | KLK8\_MOUSE | LGMN\_MOUSE | TRFL\_BUBBU | TRFL\_HORSE | GO:0071248 | 0.00900062073246431 | 29/3222 | 5/552 | 0.570858823607393 | 1 | F | F | F | F | cellular response to metal ion | AOC1\_HUMAN | ENPP2\_RAT | G3I1H5\_CRIGR | LGMN\_MOUSE | PGH2\_HUMAN | GO:0050865 | 0.00900062073246431 | 29/3222 | 5/552 | 0.570858823607393 | 1 | F | F | F | F | regulation of cell activation | CEL2A\_PIG | OXLA\_BOTAT | PA2GA\_HUMAN | PA2GE\_HUMAN | PA2GX\_HUMAN | GO:0000413 | 0.00527622594661701 | 17/3222 | 3/552 | 0.577289467958762 | 1 | F | F | F | F | protein peptidyl-prolyl isomerization | A0A0R3QSA7\_9BILA | A0A3S5H5N2\_LEIDO | CYP5\_CAEEL | GO:0009791 | 0.00527622594661701 | 17/3222 | 3/552 | 0.577289467958762 | 1 | F | F | F | F | post-embryonic development | CHIT\_PUNGR | LIPR2\_RAT | PER53\_ARATH | GO:0009889 | 0.0775915580384854 | 250/3222 | 42/552 | 0.585913645672946 | 1 | F | F | F | F | regulation of biosynthetic process | AGAL\_HUMAN | ANG1\_BOVIN | ANG3\_MOUSE | ANGI\_MOUSE | CATH\_HUMAN | CHIA\_HUMAN | CHIL3\_MOUSE | D6XHE1\_TRYB2 | E0CX04\_MOMBA | G3I1H5\_CRIGR | GBA1\_HUMAN | GRAC\_MOUSE | GRASS\_DROME | KLK7\_HUMAN | KLK7\_MOUSE | LGMN\_MOUSE | O04358\_IRIHO | PA21B\_PIG | PA2GX\_HUMAN | PGH2\_HUMAN | PGPSA\_DROME | PGRP1\_CAMDR | PPA5\_HUMAN | PPA5\_RAT | Q2QEH4\_SAPOF | Q5WRG2\_RAT | Q6R7Z5\_9TRYP | Q94BW3\_CINCA | RIP0\_DIACA | RIP1\_BRYDI | RIP1\_HORVU | RIP1\_MOMCH | RIP1\_PHYAM | RIP2\_PHYAM | RIP3\_MOMCH | RIPA\_PHYAM | RIPG\_SURMU | RIPL1\_PHYDI | RIPL2\_PHYDI | RIPT\_TRIKI | TRFL\_BUBBU | TRFL\_HORSE | GO:0046085 | 0.00341402855369336 | 11/3222 | 2/552 | 0.586058744734067 | 1 | F | F | F | F | adenosine metabolic process | ADA2\_HUMAN | PPAP\_RAT | GO:0002832 | 0.00341402855369336 | 11/3222 | 2/552 | 0.586058744734067 | 1 | F | F | F | F | negative regulation of response to biotic stimulus | TRFL\_BUBBU | TRFL\_HORSE | GO:1901570 | 0.00341402855369336 | 11/3222 | 2/552 | 0.586058744734067 | 1 | F | F | F | F | fatty acid derivative biosynthetic process | PPT1\_HUMAN | PPT2\_HUMAN | GO:0034250 | 0.00341402855369336 | 11/3222 | 2/552 | 0.586058744734067 | 1 | F | F | F | F | positive regulation of amide metabolic process | GRASS\_DROME | PGPSA\_DROME | GO:1903706 | 0.00341402855369336 | 11/3222 | 2/552 | 0.586058744734067 | 1 | F | F | F | F | regulation of hemopoiesis | TRFL\_BUBBU | TRFL\_HORSE | GO:0006691 | 0.00341402855369336 | 11/3222 | 2/552 | 0.586058744734067 | 1 | F | F | F | F | leukotriene metabolic process | CBPA1\_PIG | PA21B\_PIG | GO:0046209 | 0.00341402855369336 | 11/3222 | 2/552 | 0.586058744734067 | 1 | F | F | F | F | nitric oxide metabolic process | PPA5\_HUMAN | PPA5\_RAT | GO:0050779 | 0.00341402855369336 | 11/3222 | 2/552 | 0.586058744734067 | 1 | F | F | F | F | RNA destabilization | ANGI\_MOUSE | Q5WRG2\_RAT | GO:0009612 | 0.00341402855369336 | 11/3222 | 2/552 | 0.586058744734067 | 1 | F | F | F | F | response to mechanical stimulus | PGH2\_HUMAN | PPA5\_RAT | GO:0051171 | 0.0726256983240224 | 234/3222 | 39/552 | 0.606660656122066 | 1 | F | F | F | F | regulation of nitrogen compound metabolic process | AGAL\_HUMAN | ANG1\_BOVIN | ANG2\_MOUSE | ANG3\_MOUSE | ANG4\_MOUSE | ANGI\_MOUSE | CATD\_RAT | CATH\_HUMAN | E0CX04\_MOMBA | ENPP2\_HUMAN | ENPP2\_RAT | G3I1H5\_CRIGR | GBA1\_HUMAN | GRAA\_HUMAN | GRAC\_MOUSE | GRASS\_DROME | LGMN\_MOUSE | O04358\_IRIHO | PA21B\_PIG | PA2GX\_HUMAN | PGH2\_HUMAN | PGPSA\_DROME | PPA5\_HUMAN | PPA5\_RAT | Q2QEH4\_SAPOF | Q5WRG2\_RAT | Q94BW3\_CINCA | RIP0\_DIACA | RIP1\_BRYDI | RIP1\_HORVU | RIP1\_MOMCH | RIP1\_PHYAM | RIP2\_PHYAM | RIP3\_MOMCH | RIPA\_PHYAM | RIPG\_SURMU | RIPL1\_PHYDI | RIPL2\_PHYDI | RIPT\_TRIKI | GO:0044089 | 0.00558659217877095 | 18/3222 | 3/552 | 0.618211002284397 | 1 | F | F | F | F | positive regulation of cellular component biogenesis | ENPP2\_RAT | HYAL1\_HUMAN | MMP1\_PIG | GO:0007626 | 0.00558659217877095 | 18/3222 | 3/552 | 0.618211002284397 | 1 | F | F | F | F | locomotory behavior | ANAG\_HUMAN | DOPO\_HUMAN | PPT1\_HUMAN | GO:0034612 | 0.00558659217877095 | 18/3222 | 3/552 | 0.618211002284397 | 1 | F | F | F | F | response to tumor necrosis factor | GBA1\_HUMAN | HYAL1\_HUMAN | PGH2\_HUMAN | GO:0001558 | 0.00558659217877095 | 18/3222 | 3/552 | 0.618211002284397 | 1 | F | F | F | F | regulation of cell growth | HYAL1\_HUMAN | PPT1\_BOVIN | PPT1\_HUMAN | GO:0071478 | 0.00558659217877095 | 18/3222 | 3/552 | 0.618211002284397 | 1 | F | F | F | F | cellular response to radiation | HYAL1\_HUMAN | MMP1\_PIG | PGH2\_HUMAN | GO:0048545 | 0.0133457479826195 | 43/3222 | 7/552 | 0.622899821442233 | 1 | F | F | F | F | response to steroid hormone | CBPN\_HUMAN | GBA1\_HUMAN | LALBA\_BOVIN | LIPR2\_RAT | PGH2\_HUMAN | PTGDS\_HUMAN | PTGDS\_MOUSE | GO:0048583 | 0.058348851644941 | 188/3222 | 31/552 | 0.626809468984204 | 1 | F | F | F | F | regulation of response to stimulus | ANAG\_HUMAN | AOAH\_MOUSE | CARP1\_CANAL | CATH\_HUMAN | CFAD\_MOUSE | DNAS1\_HUMAN | DNSL3\_HUMAN | DOPO\_HUMAN | G3I1H5\_CRIGR | GBA1\_HUMAN | GRASS\_DROME | KLK7\_HUMAN | KLK7\_MOUSE | KLK8\_MOUSE | LGMN\_MOUSE | LICH\_HUMAN | PA21B\_PIG | PA2GA\_HUMAN | PA2GX\_HUMAN | PCP\_HUMAN | PGH2\_HUMAN | PGPSA\_DROME | PPA5\_HUMAN | PPA5\_RAT | PPAP\_RAT | RENI\_RAT | RIP0\_DIACA | RIP3\_MOMCH | RIPT\_TRIKI | TRFL\_BUBBU | TRFL\_HORSE | GO:0032496 | 0.0114835505896958 | 37/3222 | 6/552 | 0.627407680920844 | 1 | F | F | F | F | response to lipopolysaccharide | ANAG\_HUMAN | ANG4\_MOUSE | PGH2\_HUMAN | PPA5\_HUMAN | PPA5\_RAT | RENI\_RAT | GO:0042981 | 0.0304158907510863 | 98/3222 | 16/552 | 0.627715804062949 | 1 | F | F | F | F | regulation of apoptotic process | ATLE\_CYCAE | CATD\_RAT | CATH\_HUMAN | DOPO\_HUMAN | G3I1H5\_CRIGR | GBA1\_HUMAN | GRAA\_HUMAN | LGMN\_MOUSE | OXLA\_BOTAT | PA21B\_BOVIN | PA21B\_PIG | PGH2\_HUMAN | PPT1\_BOVIN | PPT1\_HUMAN | TRFL\_BUBBU | TRFL\_HORSE | GO:0009888 | 0.00962135319677219 | 31/3222 | 5/552 | 0.633630651409799 | 1 | F | F | F | F | tissue development | ANAG\_HUMAN | GBA1\_HUMAN | KLK7\_HUMAN | LICH\_HUMAN | PGH2\_HUMAN | GO:0051701 | 0.0037243947858473 | 12/3222 | 2/552 | 0.635452044599176 | 1 | F | F | F | F | biological process involved in interaction with host | CARP1\_CANAL | EXG1\_CANAL | GO:0071356 | 0.0037243947858473 | 12/3222 | 2/552 | 0.635452044599176 | 1 | F | F | F | F | cellular response to tumor necrosis factor | GBA1\_HUMAN | HYAL1\_HUMAN | GO:0042110 | 0.0037243947858473 | 12/3222 | 2/552 | 0.635452044599176 | 1 | F | F | F | F | T cell activation | GBA1\_HUMAN | LICH\_HUMAN | GO:1905952 | 0.0037243947858473 | 12/3222 | 2/552 | 0.635452044599176 | 1 | F | F | F | F | regulation of lipid localization | PA2GX\_HUMAN | RENI\_RAT | GO:1904018 | 0.0037243947858473 | 12/3222 | 2/552 | 0.635452044599176 | 1 | F | F | F | F | positive regulation of vasculature development | CATH\_HUMAN | HYAL1\_HUMAN | GO:0045766 | 0.0037243947858473 | 12/3222 | 2/552 | 0.635452044599176 | 1 | F | F | F | F | positive regulation of angiogenesis | CATH\_HUMAN | HYAL1\_HUMAN | GO:0032368 | 0.0037243947858473 | 12/3222 | 2/552 | 0.635452044599176 | 1 | F | F | F | F | regulation of lipid transport | PA2GX\_HUMAN | RENI\_RAT | GO:0043405 | 0.0037243947858473 | 12/3222 | 2/552 | 0.635452044599176 | 1 | F | F | F | F | regulation of MAP kinase activity | GBA1\_HUMAN | PA21B\_PIG | GO:0042391 | 0.0037243947858473 | 12/3222 | 2/552 | 0.635452044599176 | 1 | F | F | F | F | regulation of membrane potential | GBA1\_HUMAN | LICH\_HUMAN | GO:0019220 | 0.0192427063935444 | 62/3222 | 10/552 | 0.636793986859784 | 1 | F | F | F | F | regulation of phosphate metabolic process | ANG2\_MOUSE | ANG3\_MOUSE | ANG4\_MOUSE | ANGI\_MOUSE | ENPP2\_HUMAN | ENPP2\_RAT | GBA1\_HUMAN | PA21B\_PIG | PGH2\_HUMAN | Q5WRG2\_RAT | GO:0051174 | 0.0192427063935444 | 62/3222 | 10/552 | 0.636793986859784 | 1 | F | F | F | F | regulation of phosphorus metabolic process | ANG2\_MOUSE | ANG3\_MOUSE | ANG4\_MOUSE | ANGI\_MOUSE | ENPP2\_HUMAN | ENPP2\_RAT | GBA1\_HUMAN | PA21B\_PIG | PGH2\_HUMAN | Q5WRG2\_RAT | GO:0009267 | 0.00775915580384854 | 25/3222 | 4/552 | 0.642587061950556 | 1 | F | F | F | F | cellular response to starvation | AOC1\_HUMAN | DABA\_PSEMU | GBA1\_HUMAN | PPA5\_RAT | GO:0006636 | 0.00775915580384854 | 25/3222 | 4/552 | 0.642587061950556 | 1 | F | F | F | F | unsaturated fatty acid biosynthetic process | PA2GX\_HUMAN | PGH2\_HUMAN | PTGDS\_HUMAN | PTGDS\_MOUSE | GO:0030030 | 0.00775915580384854 | 25/3222 | 4/552 | 0.642587061950556 | 1 | F | F | F | F | cell projection organization | G3I1H5\_CRIGR | KLK8\_MOUSE | LGMN\_MOUSE | PA2GX\_HUMAN | GO:0044087 | 0.00775915580384854 | 25/3222 | 4/552 | 0.642587061950556 | 1 | F | F | F | F | regulation of cellular component biogenesis | ENPP2\_RAT | GBA1\_HUMAN | HYAL1\_HUMAN | MMP1\_PIG | GO:0022407 | 0.00775915580384854 | 25/3222 | 4/552 | 0.642587061950556 | 1 | F | F | F | F | regulation of cell-cell adhesion | CEL2A\_PIG | OXLA\_BOTAT | PA2GA\_HUMAN | PA2GE\_HUMAN | GO:0048519 | 0.090626939788951 | 292/3222 | 48/552 | 0.655080453739001 | 1 | F | F | F | F | negative regulation of biological process | AGAL\_HUMAN | ANG1\_BOVIN | ANG2\_MOUSE | ANG3\_MOUSE | ANG4\_MOUSE | ANGI\_MOUSE | AOAH\_MOUSE | CATH\_HUMAN | E0CX04\_MOMBA | ENPP2\_RAT | G3I1H5\_CRIGR | GBA1\_HUMAN | GILT\_MOUSE | GRAA\_HUMAN | GRAC\_MOUSE | HYAL1\_HUMAN | KLK8\_MOUSE | LGMN\_MOUSE | O04358\_IRIHO | PA2GA\_HUMAN | PA2GE\_HUMAN | PA2GX\_HUMAN | PGH2\_HUMAN | PGRP1\_CAMDR | PPA5\_HUMAN | PPA5\_RAT | PPT1\_BOVIN | PPT1\_HUMAN | PRTN3\_HUMAN | PTGDS\_HUMAN | PTGDS\_MOUSE | Q2QEH4\_SAPOF | Q5WRG2\_RAT | Q94BW3\_CINCA | RIP0\_DIACA | RIP1\_BRYDI | RIP1\_HORVU | RIP1\_MOMCH | RIP1\_PHYAM | RIP2\_PHYAM | RIP3\_MOMCH | RIPA\_PHYAM | RIPG\_SURMU | RIPL1\_PHYDI | RIPL2\_PHYDI | RIPT\_TRIKI | TRFL\_BUBBU | TRFL\_HORSE | GO:0060284 | 0.00589695841092489 | 19/3222 | 3/552 | 0.656359412969993 | 1 | F | F | F | F | regulation of cell development | ENPP2\_RAT | TRFL\_BUBBU | TRFL\_HORSE | GO:0051129 | 0.00589695841092489 | 19/3222 | 3/552 | 0.656359412969993 | 1 | F | F | F | F | negative regulation of cellular component organization | GBA1\_HUMAN | KLK8\_MOUSE | PRTN3\_HUMAN | GO:0018208 | 0.00589695841092489 | 19/3222 | 3/552 | 0.656359412969993 | 1 | F | F | F | F | peptidyl-proline modification | A0A0R3QSA7\_9BILA | A0A3S5H5N2\_LEIDO | CYP5\_CAEEL | GO:0043086 | 0.00993171942892613 | 32/3222 | 5/552 | 0.662819259814152 | 1 | F | F | F | F | negative regulation of catalytic activity | AGAL\_HUMAN | AOCX\_BOVIN | GBA1\_HUMAN | GRAA\_HUMAN | PGH2\_HUMAN | GO:1901658 | 0.0139664804469274 | 45/3222 | 7/552 | 0.672653589617249 | 1 | F | F | F | F | glycosyl compound catabolic process | ADA2\_HUMAN | AGAL\_HUMAN | BGLR\_HUMAN | FUCO\_HUMAN | GBA1\_HUMAN | NAGAB\_CHICK | NAGAB\_HUMAN | GO:0046883 | 0.00403476101800124 | 13/3222 | 2/552 | 0.68010325195361 | 1 | F | F | F | F | regulation of hormone secretion | CEL2A\_PIG | RENI\_RAT | GO:0051924 | 0.00403476101800124 | 13/3222 | 2/552 | 0.68010325195361 | 1 | F | F | F | F | regulation of calcium ion transport | PA21B\_PIG | PGH2\_HUMAN | GO:2001057 | 0.00403476101800124 | 13/3222 | 2/552 | 0.68010325195361 | 1 | F | F | F | F | reactive nitrogen species metabolic process | PPA5\_HUMAN | PPA5\_RAT | GO:0061919 | 0.00403476101800124 | 13/3222 | 2/552 | 0.68010325195361 | 1 | F | F | F | F | process utilizing autophagic mechanism | ANAG\_HUMAN | GBA1\_HUMAN | GO:0030324 | 0.00403476101800124 | 13/3222 | 2/552 | 0.68010325195361 | 1 | F | F | F | F | lung development | CERU\_RAT | LICH\_HUMAN | GO:0048638 | 0.00403476101800124 | 13/3222 | 2/552 | 0.68010325195361 | 1 | F | F | F | F | regulation of developmental growth | G3I1H5\_CRIGR | LGMN\_MOUSE | GO:0006914 | 0.00403476101800124 | 13/3222 | 2/552 | 0.68010325195361 | 1 | F | F | F | F | autophagy | ANAG\_HUMAN | GBA1\_HUMAN | GO:0002688 | 0.00403476101800124 | 13/3222 | 2/552 | 0.68010325195361 | 1 | F | F | F | F | regulation of leukocyte chemotaxis | G3I1H5\_CRIGR | LGMN\_MOUSE | GO:1903522 | 0.00403476101800124 | 13/3222 | 2/552 | 0.68010325195361 | 1 | F | F | F | F | regulation of blood circulation | DOPO\_HUMAN | PGH2\_HUMAN | GO:0031647 | 0.00403476101800124 | 13/3222 | 2/552 | 0.68010325195361 | 1 | F | F | F | F | regulation of protein stability | CATH\_HUMAN | GILT\_MOUSE | GO:0071900 | 0.00403476101800124 | 13/3222 | 2/552 | 0.68010325195361 | 1 | F | F | F | F | regulation of protein serine/threonine kinase activity | GBA1\_HUMAN | PA21B\_PIG | GO:1901565 | 0.11576660459342 | 373/3222 | 61/552 | 0.687194225288489 | 1 | F | F | F | F | organonitrogen compound catabolic process | A0A0R4I979\_BRABE | A0A3B6UEQ2\_RHIMI | A0A7S6G7I6\_9PEZI | A5AB48\_ASPNC | A6PZ97\_SALSA | A9LI60\_BIOOC | A9ZSX9\_9BRYO | ADA2\_HUMAN | AGAL\_HUMAN | ANAG\_HUMAN | B9TU22\_GADMO | BGLR\_HUMAN | CARP1\_CANAL | CATD\_RAT | CATH\_HUMAN | CBPN\_HUMAN | CDA\_COLLN | CDA\_EMENI | CEL2A\_PIG | CHI1\_COCPS | CHI2\_HORVU | CHI2\_ORYSJ | CHI33\_TRIHA | CHI42\_TRIHA | CHI4\_CRYJA | CHIA\_HUMAN | CHIC\_ARATH | CHIC\_SECCE | CHIL3\_MOUSE | CHIT\_PUNGR | CHLY\_HEVBR | DOPO\_HUMAN | DPP2\_HUMAN | ENPP2\_HUMAN | ENPP2\_RAT | FUCO\_HUMAN | G3JPF7\_CORMM | GBA1\_HUMAN | HEXC\_OSTFU | HYAL1\_HUMAN | IDUA\_HUMAN | LIPR2\_HUMAN | LIPR2\_RAT | LYG\_STRCA | MAN12\_PENCI | MANBA\_MOUSE | O81934\_CANEN | PA2GX\_HUMAN | PAG15\_HUMAN | PGPSA\_DROME | PGRP1\_CAMDR | PPT1\_BOVIN | PPT1\_HUMAN | Q43576\_TOBAC | Q4AE59\_OSTFU | Q6WSR8\_PICAB | Q86RS6\_MANSE | Q8H0C9\_VIGUN | Q9FUH3\_VIGUS | SIA\_ASPFU | TPP1\_HUMAN | GO:0050804 | 0.00620732464307883 | 20/3222 | 3/552 | 0.691687905692883 | 1 | F | F | F | F | modulation of chemical synaptic transmission | G3I1H5\_CRIGR | LGMN\_MOUSE | PGH2\_HUMAN | GO:0099177 | 0.00620732464307883 | 20/3222 | 3/552 | 0.691687905692883 | 1 | F | F | F | F | regulation of trans-synaptic signaling | G3I1H5\_CRIGR | LGMN\_MOUSE | PGH2\_HUMAN | GO:0043068 | 0.0142768466790813 | 46/3222 | 7/552 | 0.695939969732543 | 1 | F | F | F | F | positive regulation of programmed cell death | ATLE\_CYCAE | CATD\_RAT | CATH\_HUMAN | GRAA\_HUMAN | PA21B\_BOVIN | PA21B\_PIG | PGH2\_HUMAN | GO:0001666 | 0.0142768466790813 | 46/3222 | 7/552 | 0.695939969732543 | 1 | F | F | F | F | response to hypoxia | ANG1\_BOVIN | ANG2\_MOUSE | ANG3\_MOUSE | ANG4\_MOUSE | ANGI\_MOUSE | PGH2\_HUMAN | Q5WRG2\_RAT | GO:0010628 | 0.0183116076970826 | 59/3222 | 9/552 | 0.703378882633063 | 1 | F | F | F | F | positive regulation of gene expression | CATH\_HUMAN | CHIA\_HUMAN | CHIL3\_MOUSE | GRASS\_DROME | KLK7\_HUMAN | KLK7\_MOUSE | PA21B\_PIG | PGH2\_HUMAN | PGPSA\_DROME | GO:0031323 | 0.0924891371818746 | 298/3222 | 48/552 | 0.713523525581461 | 1 | F | F | F | F | regulation of cellular metabolic process | AGAL\_HUMAN | ANG1\_BOVIN | ANG2\_MOUSE | ANG3\_MOUSE | ANG4\_MOUSE | ANGI\_MOUSE | CATH\_HUMAN | CHIA\_HUMAN | CHIL3\_MOUSE | D6XHE1\_TRYB2 | E0CX04\_MOMBA | ENPP2\_HUMAN | ENPP2\_RAT | G3I1H5\_CRIGR | GBA1\_HUMAN | GRAA\_HUMAN | GRAC\_MOUSE | GRASS\_DROME | KLK7\_HUMAN | KLK7\_MOUSE | LGMN\_MOUSE | O04358\_IRIHO | PA21B\_PIG | PA2GX\_HUMAN | PCP\_HUMAN | PGH2\_HUMAN | PGPSA\_DROME | PGRP1\_CAMDR | PPA5\_HUMAN | PPA5\_RAT | Q2QEH4\_SAPOF | Q5WRG2\_RAT | Q6R7Z5\_9TRYP | Q94BW3\_CINCA | RIP0\_DIACA | RIP1\_BRYDI | RIP1\_HORVU | RIP1\_MOMCH | RIP1\_PHYAM | RIP2\_PHYAM | RIP3\_MOMCH | RIPA\_PHYAM | RIPG\_SURMU | RIPL1\_PHYDI | RIPL2\_PHYDI | RIPT\_TRIKI | TRFL\_BUBBU | TRFL\_HORSE | GO:1901652 | 0.0145872129112353 | 47/3222 | 7/552 | 0.718123986104527 | 1 | F | F | F | F | response to peptide | CEL2A\_PIG | G3I1H5\_CRIGR | LGMN\_MOUSE | LIPR2\_RAT | PA21B\_PIG | PGH2\_HUMAN | PPA5\_RAT | GO:0036293 | 0.0145872129112353 | 47/3222 | 7/552 | 0.718123986104527 | 1 | F | F | F | F | response to decreased oxygen levels | ANG1\_BOVIN | ANG2\_MOUSE | ANG3\_MOUSE | ANG4\_MOUSE | ANGI\_MOUSE | PGH2\_HUMAN | Q5WRG2\_RAT | GO:0048598 | 0.00434512725015518 | 14/3222 | 2/552 | 0.720184748906575 | 1 | F | F | F | F | embryonic morphogenesis | ANAG\_HUMAN | HYAL1\_HUMAN | GO:0050670 | 0.00434512725015518 | 14/3222 | 2/552 | 0.720184748906575 | 1 | F | F | F | F | regulation of lymphocyte proliferation | PA2GA\_HUMAN | PA2GE\_HUMAN | GO:0050920 | 0.00434512725015518 | 14/3222 | 2/552 | 0.720184748906575 | 1 | F | F | F | F | regulation of chemotaxis | G3I1H5\_CRIGR | LGMN\_MOUSE | GO:0043487 | 0.00434512725015518 | 14/3222 | 2/552 | 0.720184748906575 | 1 | F | F | F | F | regulation of RNA stability | ANGI\_MOUSE | Q5WRG2\_RAT | GO:0043281 | 0.00434512725015518 | 14/3222 | 2/552 | 0.720184748906575 | 1 | F | F | F | F | regulation of cysteine-type endopeptidase activity involved in apoptotic process | CATD\_RAT | PGH2\_HUMAN | GO:0032944 | 0.00434512725015518 | 14/3222 | 2/552 | 0.720184748906575 | 1 | F | F | F | F | regulation of mononuclear cell proliferation | PA2GA\_HUMAN | PA2GE\_HUMAN | GO:0006897 | 0.00434512725015518 | 14/3222 | 2/552 | 0.720184748906575 | 1 | F | F | F | F | endocytosis | PPT1\_BOVIN | PPT1\_HUMAN | GO:0007420 | 0.00434512725015518 | 14/3222 | 2/552 | 0.720184748906575 | 1 | F | F | F | F | brain development | PPT1\_BOVIN | PPT1\_HUMAN | GO:0043254 | 0.00434512725015518 | 14/3222 | 2/552 | 0.720184748906575 | 1 | F | F | F | F | regulation of protein-containing complex assembly | GBA1\_HUMAN | MMP1\_PIG | GO:0120036 | 0.00651769087523277 | 21/3222 | 3/552 | 0.724211499000035 | 1 | F | F | F | F | plasma membrane bounded cell projection organization | G3I1H5\_CRIGR | LGMN\_MOUSE | PA2GX\_HUMAN | GO:0009896 | 0.00651769087523277 | 21/3222 | 3/552 | 0.724211499000035 | 1 | F | F | F | F | positive regulation of catabolic process | ANGI\_MOUSE | GBA1\_HUMAN | Q5WRG2\_RAT | GO:0048584 | 0.0304158907510863 | 98/3222 | 15/552 | 0.727611442447201 | 1 | F | F | F | F | positive regulation of response to stimulus | ANAG\_HUMAN | CARP1\_CANAL | CATH\_HUMAN | CFAD\_MOUSE | G3I1H5\_CRIGR | GRASS\_DROME | KLK7\_HUMAN | KLK7\_MOUSE | LGMN\_MOUSE | LICH\_HUMAN | PA21B\_PIG | PA2GA\_HUMAN | PGH2\_HUMAN | PGPSA\_DROME | PPAP\_RAT | GO:0070482 | 0.0148975791433892 | 48/3222 | 7/552 | 0.739189215708233 | 1 | F | F | F | F | response to oxygen levels | ANG1\_BOVIN | ANG2\_MOUSE | ANG3\_MOUSE | ANG4\_MOUSE | ANGI\_MOUSE | PGH2\_HUMAN | Q5WRG2\_RAT | GO:0040008 | 0.010862818125388 | 35/3222 | 5/552 | 0.740976719799524 | 1 | F | F | F | F | regulation of growth | G3I1H5\_CRIGR | HYAL1\_HUMAN | LGMN\_MOUSE | PPT1\_BOVIN | PPT1\_HUMAN | GO:0032879 | 0.0307262569832402 | 99/3222 | 15/552 | 0.742383599051927 | 1 | F | F | F | F | regulation of localization | ANG1\_BOVIN | ANG2\_MOUSE | ANG3\_MOUSE | ANG4\_MOUSE | ANGI\_MOUSE | CATD\_RAT | CEL2A\_PIG | PA21B\_PIG | PA2GX\_HUMAN | PGH2\_HUMAN | PPT1\_BOVIN | PPT1\_HUMAN | PRTN3\_HUMAN | Q5WRG2\_RAT | RENI\_RAT | GO:0032355 | 0.00682805710738672 | 22/3222 | 3/552 | 0.753993375690669 | 1 | F | F | F | F | response to estradiol | ENPP2\_RAT | LALBA\_BOVIN | PGH2\_HUMAN | GO:0051235 | 0.00682805710738672 | 22/3222 | 3/552 | 0.753993375690669 | 1 | F | F | F | F | maintenance of location | ANAG\_HUMAN | GBA1\_HUMAN | LICH\_HUMAN | GO:0007049 | 0.00465549348230913 | 15/3222 | 2/552 | 0.755949008080022 | 1 | F | F | F | F | cell cycle | KLK10\_HUMAN | LICH\_HUMAN | GO:0045596 | 0.00465549348230913 | 15/3222 | 2/552 | 0.755949008080022 | 1 | F | F | F | F | negative regulation of cell differentiation | TRFL\_BUBBU | TRFL\_HORSE | GO:0048729 | 0.00465549348230913 | 15/3222 | 2/552 | 0.755949008080022 | 1 | F | F | F | F | tissue morphogenesis | ANAG\_HUMAN | CATH\_HUMAN | GO:0048609 | 0.00900062073246431 | 29/3222 | 4/552 | 0.75783594675221 | 1 | F | F | F | F | multicellular organismal reproductive process | ADPG2\_ARATH | ENPP2\_RAT | EST6\_DROME | PGH2\_HUMAN | GO:0000041 | 0.00900062073246431 | 29/3222 | 4/552 | 0.75783594675221 | 1 | F | F | F | F | transition metal ion transport | CERU\_RAT | PPA5\_PIG | TRFL\_BUBBU | TRFL\_HORSE | GO:0006826 | 0.00900062073246431 | 29/3222 | 4/552 | 0.75783594675221 | 1 | F | F | F | F | iron ion transport | CERU\_RAT | PPA5\_PIG | TRFL\_BUBBU | TRFL\_HORSE | GO:0051049 | 0.0291744258224705 | 94/3222 | 14/552 | 0.760974167355318 | 1 | F | F | F | F | regulation of transport | ANG1\_BOVIN | ANG2\_MOUSE | ANG3\_MOUSE | ANG4\_MOUSE | ANGI\_MOUSE | CEL2A\_PIG | PA21B\_PIG | PA2GX\_HUMAN | PGH2\_HUMAN | PPT1\_BOVIN | PPT1\_HUMAN | PRTN3\_HUMAN | Q5WRG2\_RAT | RENI\_RAT | GO:0009719 | 0.042830540037244 | 138/3222 | 21/552 | 0.762528751691161 | 1 | F | F | F | F | response to endogenous stimulus | ANG2\_MOUSE | ANG3\_MOUSE | ANG4\_MOUSE | ANGI\_MOUSE | AOC1\_HUMAN | CATH\_HUMAN | CBPN\_HUMAN | CEL2A\_PIG | CHIC\_ARATH | G3I1H5\_CRIGR | GBA1\_HUMAN | HYAL1\_HUMAN | LALBA\_BOVIN | LGMN\_MOUSE | LIPR2\_RAT | PA21B\_PIG | PGH2\_HUMAN | PPA5\_RAT | PTGDS\_HUMAN | PTGDS\_MOUSE | Q5WRG2\_RAT | GO:0009725 | 0.0353817504655493 | 114/3222 | 17/552 | 0.775242883109399 | 1 | F | F | F | F | response to hormone | ANG2\_MOUSE | ANG3\_MOUSE | ANG4\_MOUSE | ANGI\_MOUSE | CATH\_HUMAN | CBPN\_HUMAN | CEL2A\_PIG | CHIC\_ARATH | GBA1\_HUMAN | LALBA\_BOVIN | LIPR2\_RAT | PA21B\_PIG | PGH2\_HUMAN | PPA5\_RAT | PTGDS\_HUMAN | PTGDS\_MOUSE | Q5WRG2\_RAT | GO:0051051 | 0.00713842333954066 | 23/3222 | 3/552 | 0.781133041509175 | 1 | F | F | F | F | negative regulation of transport | PA2GX\_HUMAN | PGH2\_HUMAN | PRTN3\_HUMAN | GO:0006801 | 0.00713842333954066 | 23/3222 | 3/552 | 0.781133041509175 | 1 | F | F | F | F | superoxide metabolic process | ANAG\_HUMAN | PPA5\_HUMAN | PPA5\_RAT | GO:0051726 | 0.00931098696461825 | 30/3222 | 4/552 | 0.781664730423627 | 1 | F | F | F | F | regulation of cell cycle | G3I1H5\_CRIGR | HYAL1\_HUMAN | LGMN\_MOUSE | PGH2\_HUMAN | GO:0051240 | 0.0257603972687772 | 83/3222 | 12/552 | 0.78571279991487 | 1 | F | F | F | F | positive regulation of multicellular organismal process | AGAL\_ORYSJ | CATH\_HUMAN | CHIA\_HUMAN | CHIL3\_MOUSE | DOPO\_HUMAN | ENPP2\_RAT | GBA1\_HUMAN | HYAL1\_HUMAN | PA21B\_PIG | PGH2\_HUMAN | TRFL\_BUBBU | TRFL\_HORSE | GO:0051346 | 0.00496585971446307 | 16/3222 | 2/552 | 0.787696180546172 | 1 | F | F | F | F | negative regulation of hydrolase activity | GRAA\_HUMAN | PGH2\_HUMAN | GO:0046649 | 0.00496585971446307 | 16/3222 | 2/552 | 0.787696180546172 | 1 | F | F | F | F | lymphocyte activation | GBA1\_HUMAN | LICH\_HUMAN | GO:0043066 | 0.0198634388578523 | 64/3222 | 9/552 | 0.792337283818028 | 1 | F | F | F | F | negative regulation of apoptotic process | CATH\_HUMAN | G3I1H5\_CRIGR | GBA1\_HUMAN | LGMN\_MOUSE | PGH2\_HUMAN | PPT1\_BOVIN | PPT1\_HUMAN | TRFL\_BUBBU | TRFL\_HORSE | GO:0001819 | 0.00962135319677219 | 31/3222 | 4/552 | 0.803595705092727 | 1 | F | F | F | F | positive regulation of cytokine production | CHIA\_HUMAN | CHIL3\_MOUSE | PA21B\_PIG | PGH2\_HUMAN | GO:0043408 | 0.00962135319677219 | 31/3222 | 4/552 | 0.803595705092727 | 1 | F | F | F | F | regulation of MAPK cascade | GBA1\_HUMAN | PA21B\_PIG | PA2GA\_HUMAN | RENI\_RAT | GO:0071214 | 0.0074487895716946 | 24/3222 | 3/552 | 0.805756251613621 | 1 | F | F | F | F | cellular response to abiotic stimulus | HYAL1\_HUMAN | MMP1\_PIG | PGH2\_HUMAN | GO:0001889 | 0.0074487895716946 | 24/3222 | 3/552 | 0.805756251613621 | 1 | F | F | F | F | liver development | ANAG\_HUMAN | CERU\_RAT | Q5WRG2\_RAT | GO:0104004 | 0.0074487895716946 | 24/3222 | 3/552 | 0.805756251613621 | 1 | F | F | F | F | cellular response to environmental stimulus | HYAL1\_HUMAN | MMP1\_PIG | PGH2\_HUMAN | GO:0045595 | 0.0139664804469274 | 45/3222 | 6/552 | 0.808149614575318 | 1 | F | F | F | F | regulation of cell differentiation | ENPP2\_RAT | PA2GA\_HUMAN | PA2GX\_HUMAN | PGH2\_HUMAN | TRFL\_BUBBU | TRFL\_HORSE | GO:0048589 | 0.00527622594661701 | 17/3222 | 2/552 | 0.815750408890902 | 1 | F | F | F | F | developmental growth | ANG2\_MOUSE | TTHY\_CHICK | GO:0045785 | 0.00527622594661701 | 17/3222 | 2/552 | 0.815750408890902 | 1 | F | F | F | F | positive regulation of cell adhesion | ENPP2\_RAT | HYAL1\_HUMAN | GO:0010959 | 0.00527622594661701 | 17/3222 | 2/552 | 0.815750408890902 | 1 | F | F | F | F | regulation of metal ion transport | PA21B\_PIG | PGH2\_HUMAN | GO:0009143 | 0.00527622594661701 | 17/3222 | 2/552 | 0.815750408890902 | 1 | F | F | F | F | nucleoside triphosphate catabolic process | ASM3A\_HUMAN | ASM3A\_MOUSE | GO:0001822 | 0.00527622594661701 | 17/3222 | 2/552 | 0.815750408890902 | 1 | F | F | F | F | kidney development | CATH\_HUMAN | RENI\_RAT | GO:0002685 | 0.00527622594661701 | 17/3222 | 2/552 | 0.815750408890902 | 1 | F | F | F | F | regulation of leukocyte migration | G3I1H5\_CRIGR | LGMN\_MOUSE | GO:0048608 | 0.00527622594661701 | 17/3222 | 2/552 | 0.815750408890902 | 1 | F | F | F | F | reproductive structure development | PER53\_ARATH | RENI\_RAT | GO:0051090 | 0.00527622594661701 | 17/3222 | 2/552 | 0.815750408890902 | 1 | F | F | F | F | regulation of DNA-binding transcription factor activity | PA21B\_PIG | PA2GX\_HUMAN | GO:0050863 | 0.00527622594661701 | 17/3222 | 2/552 | 0.815750408890902 | 1 | F | F | F | F | regulation of T cell activation | PA2GA\_HUMAN | PA2GE\_HUMAN | GO:0034599 | 0.0245189323401614 | 79/3222 | 11/552 | 0.819378042211629 | 1 | F | F | F | F | cellular response to oxidative stress | ANAG\_HUMAN | GPX5\_HUMAN | LIG2\_PHACH | LIG4\_PHACH | LIG8\_PHACH | PEM1\_PHACH | PER\_ARTRA | PER\_COPCI | Q60FD2\_9APHY | VPL1\_PLEER | VPL2\_PLEER | GO:0048585 | 0.0245189323401614 | 79/3222 | 11/552 | 0.819378042211629 | 1 | F | F | F | F | negative regulation of response to stimulus | AOAH\_MOUSE | G3I1H5\_CRIGR | GBA1\_HUMAN | KLK8\_MOUSE | LGMN\_MOUSE | PA2GX\_HUMAN | PGH2\_HUMAN | PPA5\_HUMAN | PPA5\_RAT | TRFL\_BUBBU | TRFL\_HORSE | GO:0031669 | 0.00993171942892613 | 32/3222 | 4/552 | 0.823706625846815 | 1 | F | F | F | F | cellular response to nutrient levels | AOC1\_HUMAN | DABA\_PSEMU | GBA1\_HUMAN | PPA5\_RAT | GO:0030001 | 0.00993171942892613 | 32/3222 | 4/552 | 0.823706625846815 | 1 | F | F | F | F | metal ion transport | CERU\_RAT | PPA5\_PIG | TRFL\_BUBBU | TRFL\_HORSE | GO:0062197 | 0.026691495965239 | 86/3222 | 12/552 | 0.825142766282054 | 1 | F | F | F | F | cellular response to chemical stress | ANAG\_HUMAN | GPX5\_HUMAN | LIG2\_PHACH | LIG4\_PHACH | LIG8\_PHACH | PEM1\_PHACH | PER\_ARTRA | PER\_COPCI | PGH2\_HUMAN | Q60FD2\_9APHY | VPL1\_PLEER | VPL2\_PLEER | GO:2001233 | 0.00775915580384854 | 25/3222 | 3/552 | 0.828006595658809 | 1 | F | F | F | F | regulation of apoptotic signaling pathway | CATH\_HUMAN | DOPO\_HUMAN | PGH2\_HUMAN | GO:0034284 | 0.00775915580384854 | 25/3222 | 3/552 | 0.828006595658809 | 1 | F | F | F | F | response to monosaccharide | ANG2\_MOUSE | PGH2\_HUMAN | PPA5\_RAT | GO:0006518 | 0.0124146492861577 | 40/3222 | 5/552 | 0.840213331646425 | 1 | F | F | F | F | peptide metabolic process | CARP1\_CANAL | CATH\_HUMAN | CBPN\_HUMAN | RENI\_RAT | TPP1\_HUMAN | GO:0035384 | 0.00558659217877095 | 18/3222 | 2/552 | 0.840442834717138 | 1 | F | F | F | F | thioester biosynthetic process | PPT1\_HUMAN | PPT2\_HUMAN | GO:0071616 | 0.00558659217877095 | 18/3222 | 2/552 | 0.840442834717138 | 1 | F | F | F | F | acyl-CoA biosynthetic process | PPT1\_HUMAN | PPT2\_HUMAN | GO:0098657 | 0.00558659217877095 | 18/3222 | 2/552 | 0.840442834717138 | 1 | F | F | F | F | import into cell | PPT1\_BOVIN | PPT1\_HUMAN | GO:0044092 | 0.0145872129112353 | 47/3222 | 6/552 | 0.84061868206223 | 1 | F | F | F | F | negative regulation of molecular function | AGAL\_HUMAN | AOCX\_BOVIN | GBA1\_HUMAN | GRAA\_HUMAN | PA2GX\_HUMAN | PGH2\_HUMAN | GO:0016192 | 0.0102420856610801 | 33/3222 | 4/552 | 0.842085803063042 | 1 | F | F | F | F | vesicle-mediated transport | PPT1\_BOVIN | PPT1\_HUMAN | PTGDS\_HUMAN | PTGDS\_MOUSE | GO:0006812 | 0.0102420856610801 | 33/3222 | 4/552 | 0.842085803063042 | 1 | F | F | F | F | monoatomic cation transport | CERU\_RAT | PPA5\_PIG | TRFL\_BUBBU | TRFL\_HORSE | GO:0060627 | 0.00806952203600248 | 26/3222 | 3/552 | 0.848038592718785 | 1 | F | F | F | F | regulation of vesicle-mediated transport | PPT1\_BOVIN | PPT1\_HUMAN | PRTN3\_HUMAN | GO:0002694 | 0.00806952203600248 | 26/3222 | 3/552 | 0.848038592718785 | 1 | F | F | F | F | regulation of leukocyte activation | PA2GA\_HUMAN | PA2GE\_HUMAN | PA2GX\_HUMAN | GO:0070663 | 0.00589695841092489 | 19/3222 | 2/552 | 0.862099707863292 | 1 | F | F | F | F | regulation of leukocyte proliferation | PA2GA\_HUMAN | PA2GE\_HUMAN | GO:0045765 | 0.00589695841092489 | 19/3222 | 2/552 | 0.862099707863292 | 1 | F | F | F | F | regulation of angiogenesis | CATH\_HUMAN | HYAL1\_HUMAN | GO:1901342 | 0.00589695841092489 | 19/3222 | 2/552 | 0.862099707863292 | 1 | F | F | F | F | regulation of vasculature development | CATH\_HUMAN | HYAL1\_HUMAN | GO:0032868 | 0.00837988826815642 | 27/3222 | 3/552 | 0.866012128669161 | 1 | F | F | F | F | response to insulin | CEL2A\_PIG | PA21B\_PIG | PPA5\_RAT | GO:0019369 | 0.00837988826815642 | 27/3222 | 3/552 | 0.866012128669161 | 1 | F | F | F | F | arachidonic acid metabolic process | PA2GX\_HUMAN | PGH2\_HUMAN | PTGDS\_HUMAN | GO:2000026 | 0.0173805090006207 | 56/3222 | 7/552 | 0.868487781928769 | 1 | F | F | F | F | regulation of multicellular organismal development | AGAL\_ORYSJ | CATH\_HUMAN | ENPP2\_RAT | HYAL1\_HUMAN | KLK8\_MOUSE | TRFL\_BUBBU | TRFL\_HORSE | GO:0048869 | 0.0341402855369336 | 110/3222 | 15/552 | 0.870218882595039 | 1 | F | F | F | F | cellular developmental process | ANAG\_HUMAN | ANG1\_BOVIN | ANG3\_MOUSE | ANGI\_MOUSE | DNSL3\_HUMAN | GBA1\_HUMAN | LICH\_HUMAN | PA2GX\_HUMAN | PGH2\_HUMAN | PPA5\_RAT | PPT1\_HUMAN | PRTN3\_HUMAN | RENI\_RAT | RNSL3\_DANRE | TPP1\_HUMAN | GO:0043067 | 0.0363128491620112 | 117/3222 | 16/552 | 0.873945252411306 | 1 | F | F | F | F | regulation of programmed cell death | ATLE\_CYCAE | CATD\_RAT | CATH\_HUMAN | DOPO\_HUMAN | G3I1H5\_CRIGR | GBA1\_HUMAN | GRAA\_HUMAN | LGMN\_MOUSE | OXLA\_BOTAT | PA21B\_BOVIN | PA21B\_PIG | PGH2\_HUMAN | PPT1\_BOVIN | PPT1\_HUMAN | TRFL\_BUBBU | TRFL\_HORSE | GO:0033993 | 0.0363128491620112 | 117/3222 | 16/552 | 0.873945252411306 | 1 | F | F | F | F | response to lipid | ANAG\_HUMAN | ANG4\_MOUSE | CATH\_HUMAN | CBPN\_HUMAN | CHIC\_ARATH | ENPP2\_RAT | GBA1\_HUMAN | GPX3\_HUMAN | LALBA\_BOVIN | LIPR2\_RAT | PGH2\_HUMAN | PPA5\_HUMAN | PPA5\_RAT | PTGDS\_HUMAN | PTGDS\_MOUSE | RENI\_RAT | GO:0071407 | 0.010862818125388 | 35/3222 | 4/552 | 0.874035495419566 | 1 | F | F | F | F | cellular response to organic cyclic compound | ANAG\_HUMAN | AOC1\_HUMAN | ENPP2\_RAT | PGH2\_HUMAN | GO:0034655 | 0.0344506517690875 | 111/3222 | 15/552 | 0.878824335988278 | 1 | F | F | F | F | nucleobase-containing compound catabolic process | ADA2\_HUMAN | ANG4\_MOUSE | ASM3A\_HUMAN | ASM3A\_MOUSE | DNAS1\_HUMAN | DNSL3\_HUMAN | ECP\_HUMAN | ENDO2\_ARATH | NUP1\_PENCI | NUS1\_ASPOR | Q0KFV0\_SOLLC | RNAS6\_HUMAN | RNLE\_SOLLC | RNT2\_HUMAN | V5NTD\_NAJAT | GO:0009746 | 0.00620732464307883 | 20/3222 | 2/552 | 0.881034352548939 | 1 | F | F | F | F | response to hexose | ANG2\_MOUSE | PGH2\_HUMAN | GO:0031331 | 0.00620732464307883 | 20/3222 | 2/552 | 0.881034352548939 | 1 | F | F | F | F | positive regulation of cellular catabolic process | ANGI\_MOUSE | Q5WRG2\_RAT | GO:0043410 | 0.00620732464307883 | 20/3222 | 2/552 | 0.881034352548939 | 1 | F | F | F | F | positive regulation of MAPK cascade | PA21B\_PIG | PA2GA\_HUMAN | GO:1901653 | 0.00869025450031037 | 28/3222 | 3/552 | 0.882088064261155 | 1 | F | F | F | F | cellular response to peptide | G3I1H5\_CRIGR | LGMN\_MOUSE | PA21B\_PIG | GO:0022603 | 0.0111731843575419 | 36/3222 | 4/552 | 0.887807934581121 | 1 | F | F | F | F | regulation of anatomical structure morphogenesis | CATH\_HUMAN | ENPP2\_HUMAN | ENPP2\_RAT | HYAL1\_HUMAN | GO:0006144 | 0.0111731843575419 | 36/3222 | 4/552 | 0.887807934581121 | 1 | F | F | F | F | purine nucleobase metabolic process | PPAP\_RAT | TTHY\_CHICK | TTHY\_MOUSE | TTHY\_RAT | GO:0009743 | 0.0111731843575419 | 36/3222 | 4/552 | 0.887807934581121 | 1 | F | F | F | F | response to carbohydrate | ANAG\_HUMAN | ANG2\_MOUSE | PGH2\_HUMAN | PPA5\_RAT | GO:0042594 | 0.0111731843575419 | 36/3222 | 4/552 | 0.887807934581121 | 1 | F | F | F | F | response to starvation | AOC1\_HUMAN | DABA\_PSEMU | GBA1\_HUMAN | PPA5\_RAT | GO:0043434 | 0.0136561142147734 | 44/3222 | 5/552 | 0.895045419977887 | 1 | F | F | F | F | response to peptide hormone | CEL2A\_PIG | LIPR2\_RAT | PA21B\_PIG | PGH2\_HUMAN | PPA5\_RAT | GO:0071417 | 0.0136561142147734 | 44/3222 | 5/552 | 0.895045419977887 | 1 | F | F | F | F | cellular response to organonitrogen compound | AOC1\_HUMAN | G3I1H5\_CRIGR | LGMN\_MOUSE | PA21B\_PIG | PGH2\_HUMAN | GO:2000377 | 0.00900062073246431 | 29/3222 | 3/552 | 0.896424847051515 | 1 | F | F | F | F | regulation of reactive oxygen species metabolic process | PCP\_HUMAN | PPA5\_HUMAN | PPA5\_RAT | GO:0051249 | 0.00651769087523277 | 21/3222 | 2/552 | 0.897542021342988 | 1 | F | F | F | F | regulation of lymphocyte activation | PA2GA\_HUMAN | PA2GE\_HUMAN | GO:1903037 | 0.00651769087523277 | 21/3222 | 2/552 | 0.897542021342988 | 1 | F | F | F | F | regulation of leukocyte cell-cell adhesion | PA2GA\_HUMAN | PA2GE\_HUMAN | GO:0050793 | 0.0310366232153942 | 100/3222 | 13/552 | 0.897633099688417 | 1 | F | F | F | F | regulation of developmental process | AGAL\_ORYSJ | CATH\_HUMAN | ENPP2\_HUMAN | ENPP2\_RAT | G3I1H5\_CRIGR | HYAL1\_HUMAN | KLK8\_MOUSE | LGMN\_MOUSE | PA2GA\_HUMAN | PA2GX\_HUMAN | PGH2\_HUMAN | TRFL\_BUBBU | TRFL\_HORSE | GO:0071495 | 0.0183116076970826 | 59/3222 | 7/552 | 0.900899351800551 | 1 | F | F | F | F | cellular response to endogenous stimulus | AOC1\_HUMAN | CATH\_HUMAN | G3I1H5\_CRIGR | HYAL1\_HUMAN | LGMN\_MOUSE | PA21B\_PIG | PGH2\_HUMAN | GO:0030154 | 0.0270018621973929 | 87/3222 | 11/552 | 0.902132921721304 | 1 | F | F | F | F | cell differentiation | ANAG\_HUMAN | ANG1\_BOVIN | ANG3\_MOUSE | ANGI\_MOUSE | GBA1\_HUMAN | LICH\_HUMAN | PGH2\_HUMAN | PPA5\_RAT | PRTN3\_HUMAN | RNSL3\_DANRE | TPP1\_HUMAN | GO:0009968 | 0.016139044072005 | 52/3222 | 6/552 | 0.902631509445638 | 1 | F | F | F | F | negative regulation of signal transduction | G3I1H5\_CRIGR | GBA1\_HUMAN | LGMN\_MOUSE | PGH2\_HUMAN | TRFL\_BUBBU | TRFL\_HORSE | GO:0071496 | 0.0139664804469274 | 45/3222 | 5/552 | 0.905907395653377 | 1 | F | F | F | F | cellular response to external stimulus | AOC1\_HUMAN | DABA\_PSEMU | GBA1\_HUMAN | PGH2\_HUMAN | PPA5\_RAT | GO:0019222 | 0.105214152700186 | 339/3222 | 50/552 | 0.906179445656867 | 1 | F | F | F | F | regulation of metabolic process | AGAL\_HUMAN | ANG1\_BOVIN | ANG2\_MOUSE | ANG3\_MOUSE | ANG4\_MOUSE | ANGI\_MOUSE | CATD\_RAT | CATH\_HUMAN | CHIA\_HUMAN | CHIL3\_MOUSE | D6XHE1\_TRYB2 | DOPO\_HUMAN | E0CX04\_MOMBA | ENPP2\_HUMAN | ENPP2\_RAT | G3I1H5\_CRIGR | GBA1\_HUMAN | GRAA\_HUMAN | GRAC\_MOUSE | GRASS\_DROME | KLK7\_HUMAN | KLK7\_MOUSE | LGMN\_MOUSE | O04358\_IRIHO | PA21B\_PIG | PA2GX\_HUMAN | PCP\_HUMAN | PGH2\_HUMAN | PGPSA\_DROME | PGRP1\_CAMDR | PPA5\_HUMAN | PPA5\_RAT | Q2QEH4\_SAPOF | Q5WRG2\_RAT | Q6R7Z5\_9TRYP | Q94BW3\_CINCA | RIP0\_DIACA | RIP1\_BRYDI | RIP1\_HORVU | RIP1\_MOMCH | RIP1\_PHYAM | RIP2\_PHYAM | RIP3\_MOMCH | RIPA\_PHYAM | RIPG\_SURMU | RIPL1\_PHYDI | RIPL2\_PHYDI | RIPT\_TRIKI | TRFL\_BUBBU | TRFL\_HORSE | GO:0043069 | 0.0229671011793917 | 74/3222 | 9/552 | 0.90878276851297 | 1 | F | F | F | F | negative regulation of programmed cell death | CATH\_HUMAN | G3I1H5\_CRIGR | GBA1\_HUMAN | LGMN\_MOUSE | PGH2\_HUMAN | PPT1\_BOVIN | PPT1\_HUMAN | TRFL\_BUBBU | TRFL\_HORSE | GO:0009966 | 0.0356921166977033 | 115/3222 | 15/552 | 0.908810228350894 | 1 | F | F | F | F | regulation of signal transduction | CARP1\_CANAL | CATH\_HUMAN | DOPO\_HUMAN | G3I1H5\_CRIGR | GBA1\_HUMAN | LGMN\_MOUSE | LICH\_HUMAN | PA21B\_PIG | PA2GA\_HUMAN | PCP\_HUMAN | PGH2\_HUMAN | PPAP\_RAT | RENI\_RAT | TRFL\_BUBBU | TRFL\_HORSE | GO:0006457 | 0.00931098696461825 | 30/3222 | 3/552 | 0.909175971120653 | 1 | F | F | F | F | protein folding | A0A0R3QSA7\_9BILA | A0A3S5H5N2\_LEIDO | CYP5\_CAEEL | GO:0070085 | 0.00931098696461825 | 30/3222 | 3/552 | 0.909175971120653 | 1 | F | F | F | F | glycosylation | GBA1\_HUMAN | MAN12\_PENCI | OFUT1\_CAEEL | GO:0006693 | 0.0117939168218498 | 38/3222 | 4/552 | 0.911456780315223 | 1 | F | F | F | F | prostaglandin metabolic process | PA2GX\_HUMAN | PGH2\_HUMAN | PTGDS\_HUMAN | PTGDS\_MOUSE | GO:0006692 | 0.0117939168218498 | 38/3222 | 4/552 | 0.911456780315223 | 1 | F | F | F | F | prostanoid metabolic process | PA2GX\_HUMAN | PGH2\_HUMAN | PTGDS\_HUMAN | PTGDS\_MOUSE | GO:0048468 | 0.00682805710738672 | 22/3222 | 2/552 | 0.911896885907462 | 1 | F | F | F | F | cell development | ANAG\_HUMAN | PPT1\_HUMAN | GO:0040007 | 0.00682805710738672 | 22/3222 | 2/552 | 0.911896885907462 | 1 | F | F | F | F | growth | ANG2\_MOUSE | TTHY\_CHICK | GO:0048511 | 0.00682805710738672 | 22/3222 | 2/552 | 0.911896885907462 | 1 | F | F | F | F | rhythmic process | ANAG\_HUMAN | ENPP2\_RAT | GO:0009893 | 0.0502793296089385 | 162/3222 | 22/552 | 0.91273360193896 | 1 | F | F | F | F | positive regulation of metabolic process | ANG2\_MOUSE | ANG3\_MOUSE | ANG4\_MOUSE | ANGI\_MOUSE | CATD\_RAT | CATH\_HUMAN | CHIA\_HUMAN | CHIL3\_MOUSE | DOPO\_HUMAN | ENPP2\_HUMAN | ENPP2\_RAT | G3I1H5\_CRIGR | GBA1\_HUMAN | GRASS\_DROME | KLK7\_HUMAN | KLK7\_MOUSE | LGMN\_MOUSE | PA21B\_PIG | PA2GX\_HUMAN | PGH2\_HUMAN | PGPSA\_DROME | Q5WRG2\_RAT | GO:0010647 | 0.0232774674115456 | 75/3222 | 9/552 | 0.916592521085425 | 1 | F | F | F | F | positive regulation of cell communication | CATH\_HUMAN | G3I1H5\_CRIGR | GBA1\_HUMAN | LGMN\_MOUSE | LICH\_HUMAN | PA21B\_PIG | PA2GA\_HUMAN | PGH2\_HUMAN | PPAP\_RAT | GO:0044242 | 0.031967721911856 | 103/3222 | 13/552 | 0.918790599156726 | 1 | F | F | F | F | cellular lipid catabolic process | AGAL\_HUMAN | ANAG\_HUMAN | ENPP2\_HUMAN | ENPP2\_RAT | FUCO\_HUMAN | GBA1\_HUMAN | LIPR2\_HUMAN | LIPR2\_RAT | NAGAB\_HUMAN | PA2GX\_HUMAN | PAG15\_HUMAN | PPT1\_HUMAN | SIA\_ASPFU | GO:0048732 | 0.00962135319677219 | 31/3222 | 3/552 | 0.920488142481209 | 1 | F | F | F | F | gland development | ANAG\_HUMAN | CERU\_RAT | Q5WRG2\_RAT | GO:0033500 | 0.00962135319677219 | 31/3222 | 3/552 | 0.920488142481209 | 1 | F | F | F | F | carbohydrate homeostasis | ANG2\_MOUSE | DOPO\_HUMAN | PCP\_HUMAN | GO:0009411 | 0.00962135319677219 | 31/3222 | 3/552 | 0.920488142481209 | 1 | F | F | F | F | response to UV | HYAL1\_HUMAN | MMP1\_PIG | PGH2\_HUMAN | GO:0042593 | 0.00962135319677219 | 31/3222 | 3/552 | 0.920488142481209 | 1 | F | F | F | F | glucose homeostasis | ANG2\_MOUSE | DOPO\_HUMAN | PCP\_HUMAN | GO:0006811 | 0.0121042830540037 | 39/3222 | 4/552 | 0.921531659396947 | 1 | F | F | F | F | monoatomic ion transport | CERU\_RAT | PPA5\_PIG | TRFL\_BUBBU | TRFL\_HORSE | GO:0006970 | 0.00713842333954066 | 23/3222 | 2/552 | 0.924350585373943 | 1 | F | F | F | F | response to osmotic stress | CHIC\_ARATH | PGH2\_HUMAN | GO:0044403 | 0.00713842333954066 | 23/3222 | 2/552 | 0.924350585373943 | 1 | F | F | F | F | biological process involved in symbiotic interaction | CARP1\_CANAL | EXG1\_CANAL | GO:0007600 | 0.00713842333954066 | 23/3222 | 2/552 | 0.924350585373943 | 1 | F | F | F | F | sensory perception | PGH2\_HUMAN | PPT1\_HUMAN | GO:0034030 | 0.0074487895716946 | 24/3222 | 2/552 | 0.935131888442464 | 1 | F | F | F | F | ribonucleoside bisphosphate biosynthetic process | PPT1\_HUMAN | PPT2\_HUMAN | GO:0033866 | 0.0074487895716946 | 24/3222 | 2/552 | 0.935131888442464 | 1 | F | F | F | F | nucleoside bisphosphate biosynthetic process | PPT1\_HUMAN | PPT2\_HUMAN | GO:0033273 | 0.0074487895716946 | 24/3222 | 2/552 | 0.935131888442464 | 1 | F | F | F | F | response to vitamin | PGH2\_HUMAN | PPA5\_RAT | GO:0034033 | 0.0074487895716946 | 24/3222 | 2/552 | 0.935131888442464 | 1 | F | F | F | F | purine nucleoside bisphosphate biosynthetic process | PPT1\_HUMAN | PPT2\_HUMAN | GO:0006259 | 0.0173805090006207 | 56/3222 | 6/552 | 0.936106902816939 | 1 | F | F | F | F | DNA metabolic process | DNAS1\_HUMAN | DNSL3\_HUMAN | ENDO2\_ARATH | NUP1\_PENCI | NUS1\_ASPOR | Q0KFV0\_SOLLC | GO:0010557 | 0.0242085661080074 | 78/3222 | 9/552 | 0.93669188936508 | 1 | F | F | F | F | positive regulation of macromolecule biosynthetic process | CATH\_HUMAN | CHIA\_HUMAN | CHIL3\_MOUSE | GRASS\_DROME | KLK7\_HUMAN | KLK7\_MOUSE | PA21B\_PIG | PGH2\_HUMAN | PGPSA\_DROME | GO:0009416 | 0.0127250155183116 | 41/3222 | 4/552 | 0.938652769518355 | 1 | F | F | F | F | response to light stimulus | DOPO\_HUMAN | HYAL1\_HUMAN | MMP1\_PIG | PGH2\_HUMAN | GO:0071310 | 0.0332091868404718 | 107/3222 | 13/552 | 0.941195532057221 | 1 | F | F | F | F | cellular response to organic substance | ANAG\_HUMAN | ANG2\_MOUSE | ANG4\_MOUSE | AOC1\_HUMAN | CATH\_HUMAN | ENPP2\_RAT | G3I1H5\_CRIGR | GBA1\_HUMAN | HYAL1\_HUMAN | LGMN\_MOUSE | PA21B\_PIG | PA2GX\_HUMAN | PGH2\_HUMAN | GO:0008283 | 0.0176908752327747 | 57/3222 | 6/552 | 0.942690162329857 | 1 | F | F | F | F | cell population proliferation | G3I1H5\_CRIGR | GBA1\_HUMAN | KLK8\_MOUSE | LGMN\_MOUSE | LICH\_HUMAN | PA2GX\_HUMAN | GO:0019439 | 0.0667287399130975 | 215/3222 | 29/552 | 0.944194723835974 | 1 | F | F | F | F | aromatic compound catabolic process | ADA2\_HUMAN | ANG4\_MOUSE | ASM3A\_HUMAN | ASM3A\_MOUSE | DNAS1\_HUMAN | DNSL3\_HUMAN | DOPO\_HUMAN | ECP\_HUMAN | ENDO2\_ARATH | GCE2\_MYCTT | GCE\_CERUI | GCE\_HYPJQ | LAC1\_MELAO | LAC1\_TRAMX | LAC2\_TRAVE | LIG2\_PHACH | LIG4\_PHACH | LIG8\_PHACH | NUP1\_PENCI | NUS1\_ASPOR | PEM1\_PHACH | Q0KFV0\_SOLLC | Q60FD2\_9APHY | RNAS6\_HUMAN | RNLE\_SOLLC | RNT2\_HUMAN | V5NTD\_NAJAT | VPL1\_PLEER | VPL2\_PLEER | GO:0061024 | 0.00775915580384854 | 25/3222 | 2/552 | 0.944447131293476 | 1 | F | F | F | F | membrane organization | PPT1\_BOVIN | PPT1\_HUMAN | GO:0046683 | 0.00775915580384854 | 25/3222 | 2/552 | 0.944447131293476 | 1 | F | F | F | F | response to organophosphorus | PGH2\_HUMAN | RENI\_RAT | GO:1901568 | 0.00775915580384854 | 25/3222 | 2/552 | 0.944447131293476 | 1 | F | F | F | F | fatty acid derivative metabolic process | PPT1\_HUMAN | PPT2\_HUMAN | GO:0031668 | 0.0130353817504655 | 42/3222 | 4/552 | 0.945873934084573 | 1 | F | F | F | F | cellular response to extracellular stimulus | AOC1\_HUMAN | DABA\_PSEMU | GBA1\_HUMAN | PPA5\_RAT | GO:1901699 | 0.0155183116076971 | 50/3222 | 5/552 | 0.946713943288285 | 1 | F | F | F | F | cellular response to nitrogen compound | AOC1\_HUMAN | G3I1H5\_CRIGR | LGMN\_MOUSE | PA21B\_PIG | PGH2\_HUMAN | GO:0051128 | 0.0248292985723153 | 80/3222 | 9/552 | 0.947625725635388 | 1 | F | F | F | F | regulation of cellular component organization | ENPP2\_HUMAN | ENPP2\_RAT | GBA1\_HUMAN | HYAL1\_HUMAN | KLK8\_MOUSE | MMP1\_PIG | PPT1\_BOVIN | PPT1\_HUMAN | PRTN3\_HUMAN | GO:0010648 | 0.0180012414649286 | 58/3222 | 6/552 | 0.948661536308978 | 1 | F | F | F | F | negative regulation of cell communication | G3I1H5\_CRIGR | GBA1\_HUMAN | LGMN\_MOUSE | PGH2\_HUMAN | TRFL\_BUBBU | TRFL\_HORSE | GO:0023057 | 0.0180012414649286 | 58/3222 | 6/552 | 0.948661536308978 | 1 | F | F | F | F | negative regulation of signaling | G3I1H5\_CRIGR | GBA1\_HUMAN | LGMN\_MOUSE | PGH2\_HUMAN | TRFL\_BUBBU | TRFL\_HORSE | GO:0022414 | 0.031657355679702 | 102/3222 | 12/552 | 0.950353150286949 | 1 | F | F | F | F | reproductive process | ADPG2\_ARATH | ANG1\_BOVIN | CERU\_RAT | DOPO\_HUMAN | ENPP2\_RAT | EST6\_DROME | NADA\_APLCA | PA2GX\_HUMAN | PER53\_ARATH | PGH2\_HUMAN | RENI\_RAT | TTHY\_CHICK | GO:0043269 | 0.00806952203600248 | 26/3222 | 2/552 | 0.952481176175322 | 1 | F | F | F | F | regulation of monoatomic ion transport | PA21B\_PIG | PGH2\_HUMAN | GO:0023056 | 0.0229671011793917 | 74/3222 | 8/552 | 0.953975969060622 | 1 | F | F | F | F | positive regulation of signaling | CATH\_HUMAN | G3I1H5\_CRIGR | LGMN\_MOUSE | LICH\_HUMAN | PA21B\_PIG | PA2GA\_HUMAN | PGH2\_HUMAN | PPAP\_RAT | GO:0031329 | 0.010862818125388 | 35/3222 | 3/552 | 0.95398588450455 | 1 | F | F | F | F | regulation of cellular catabolic process | ANGI\_MOUSE | GBA1\_HUMAN | Q5WRG2\_RAT | GO:0034097 | 0.0183116076970826 | 59/3222 | 6/552 | 0.954068576064758 | 1 | F | F | F | F | response to cytokine | GBA1\_HUMAN | HYAL1\_HUMAN | PA2GX\_HUMAN | PGH2\_HUMAN | PPA5\_HUMAN | PPA5\_RAT | GO:0080090 | 0.0782122905027933 | 252/3222 | 34/552 | 0.957127539631892 | 1 | F | F | F | F | regulation of primary metabolic process | ANG1\_BOVIN | ANG2\_MOUSE | ANG3\_MOUSE | ANG4\_MOUSE | ANGI\_MOUSE | CATD\_RAT | CATH\_HUMAN | E0CX04\_MOMBA | ENPP2\_HUMAN | ENPP2\_RAT | G3I1H5\_CRIGR | GBA1\_HUMAN | GRAA\_HUMAN | GRAC\_MOUSE | LGMN\_MOUSE | O04358\_IRIHO | PA21B\_PIG | PA2GX\_HUMAN | PGH2\_HUMAN | Q2QEH4\_SAPOF | Q5WRG2\_RAT | Q94BW3\_CINCA | RIP0\_DIACA | RIP1\_BRYDI | RIP1\_HORVU | RIP1\_MOMCH | RIP1\_PHYAM | RIP2\_PHYAM | RIP3\_MOMCH | RIPA\_PHYAM | RIPG\_SURMU | RIPL1\_PHYDI | RIPL2\_PHYDI | RIPT\_TRIKI | GO:0007154 | 0.016139044072005 | 52/3222 | 5/552 | 0.957958514138056 | 1 | F | F | F | F | cell communication | AOC1\_HUMAN | DABA\_PSEMU | DOPO\_HUMAN | GBA1\_HUMAN | PPA5\_RAT | GO:0065008 | 0.0661080074487896 | 213/3222 | 28/552 | 0.958246288195129 | 1 | F | F | F | F | regulation of biological quality | ANAG\_HUMAN | ANGI\_MOUSE | CATH\_HUMAN | CEL2A\_PIG | CERU\_RAT | CKX1\_MAIZE | DOPO\_HUMAN | EGFB2\_MOUSE | EST6\_DROME | G3I1H5\_CRIGR | GBA1\_HUMAN | GILT\_MOUSE | KLK1\_HUMAN | LGMN\_MOUSE | LICH\_HUMAN | NP1\_RHOPR | NP2\_RHOPR | NP4\_RHOPR | PA21B\_PIG | PA2A\_BOTJR | PCP\_HUMAN | PGH2\_HUMAN | PPT1\_BOVIN | PPT1\_HUMAN | Q5WRG2\_RAT | RENI\_RAT | TTHY\_CHICK | TTHY\_RAT | GO:0070542 | 0.00837988826815642 | 27/3222 | 2/552 | 0.959398699974423 | 1 | F | F | F | F | response to fatty acid | CHIC\_ARATH | PGH2\_HUMAN | GO:0030003 | 0.0111731843575419 | 36/3222 | 3/552 | 0.960002061770381 | 1 | F | F | F | F | intracellular monoatomic cation homeostasis | CERU\_RAT | PPT1\_BOVIN | PPT1\_HUMAN | GO:0055080 | 0.0139664804469274 | 45/3222 | 4/552 | 0.963122760669242 | 1 | F | F | F | F | monoatomic cation homeostasis | ANAG\_HUMAN | CERU\_RAT | PPT1\_BOVIN | PPT1\_HUMAN | GO:0006873 | 0.0114835505896958 | 37/3222 | 3/552 | 0.965274464378825 | 1 | F | F | F | F | intracellular monoatomic ion homeostasis | CERU\_RAT | PPT1\_BOVIN | PPT1\_HUMAN | GO:0051254 | 0.0114835505896958 | 37/3222 | 3/552 | 0.965274464378825 | 1 | F | F | F | F | positive regulation of RNA metabolic process | ANGI\_MOUSE | PA21B\_PIG | Q5WRG2\_RAT | GO:0007166 | 0.0114835505896958 | 37/3222 | 3/552 | 0.965274464378825 | 1 | F | F | F | F | cell surface receptor signaling pathway | ANAG\_HUMAN | CFAD\_MOUSE | OFUT1\_CAEEL | GO:0014074 | 0.00869025450031037 | 28/3222 | 2/552 | 0.965345671951845 | 1 | F | F | F | F | response to purine-containing compound | PGH2\_HUMAN | RENI\_RAT | GO:0043413 | 0.00869025450031037 | 28/3222 | 2/552 | 0.965345671951845 | 1 | F | F | F | F | macromolecule glycosylation | MAN12\_PENCI | OFUT1\_CAEEL | GO:0006486 | 0.00869025450031037 | 28/3222 | 2/552 | 0.965345671951845 | 1 | F | F | F | F | protein glycosylation | MAN12\_PENCI | OFUT1\_CAEEL | GO:0003006 | 0.0192427063935444 | 62/3222 | 6/552 | 0.967343335277134 | 1 | F | F | F | F | developmental process involved in reproduction | ADPG2\_ARATH | ANG1\_BOVIN | PER53\_ARATH | PGH2\_HUMAN | RENI\_RAT | TTHY\_CHICK | GO:0031325 | 0.0440720049658597 | 142/3222 | 17/552 | 0.967364827623173 | 1 | F | F | F | F | positive regulation of cellular metabolic process | ANG2\_MOUSE | ANG3\_MOUSE | ANG4\_MOUSE | ANGI\_MOUSE | CATH\_HUMAN | CHIA\_HUMAN | CHIL3\_MOUSE | ENPP2\_HUMAN | ENPP2\_RAT | GBA1\_HUMAN | GRASS\_DROME | KLK7\_HUMAN | KLK7\_MOUSE | PA21B\_PIG | PGH2\_HUMAN | PGPSA\_DROME | Q5WRG2\_RAT | GO:0009314 | 0.0142768466790813 | 46/3222 | 4/552 | 0.967631932083242 | 1 | F | F | F | F | response to radiation | DOPO\_HUMAN | HYAL1\_HUMAN | MMP1\_PIG | PGH2\_HUMAN | GO:0008203 | 0.0117939168218498 | 38/3222 | 3/552 | 0.969887408728611 | 1 | F | F | F | F | cholesterol metabolic process | GBA1\_HUMAN | LICH\_HUMAN | LIP3\_DIURU | GO:0019748 | 0.0400372439478585 | 129/3222 | 15/552 | 0.969972949357912 | 1 | F | F | F | F | secondary metabolic process | DIR\_GLYEC | EST6\_DROME | GCE2\_MYCTT | GCE\_CERUI | GCE\_HYPJQ | LAC1\_MELAO | LAC1\_TRAMX | LAC2\_TRAVE | LIG2\_PHACH | LIG4\_PHACH | LIG8\_PHACH | PEM1\_PHACH | Q60FD2\_9APHY | VPL1\_PLEER | VPL2\_PLEER | GO:1902532 | 0.00900062073246431 | 29/3222 | 2/552 | 0.970450918241683 | 1 | F | F | F | F | negative regulation of intracellular signal transduction | GBA1\_HUMAN | PGH2\_HUMAN | GO:1902074 | 0.0170701427684668 | 55/3222 | 5/552 | 0.970808279820246 | 1 | F | F | F | F | response to salt | AOC1\_HUMAN | G3I1H5\_CRIGR | LGMN\_MOUSE | PGH2\_HUMAN | PPA5\_RAT | GO:0046434 | 0.026691495965239 | 86/3222 | 9/552 | 0.971103951196262 | 1 | F | F | F | F | organophosphate catabolic process | ASM3A\_HUMAN | ASM3A\_MOUSE | ENPP2\_HUMAN | ENPP2\_RAT | LIPR2\_HUMAN | LIPR2\_RAT | PA2GX\_HUMAN | PAG15\_HUMAN | V5NTD\_NAJAT | GO:0010038 | 0.0220360024829299 | 71/3222 | 7/552 | 0.97161251119112 | 1 | F | F | F | F | response to metal ion | AOC1\_HUMAN | CERU\_RAT | ENPP2\_RAT | G3I1H5\_CRIGR | LGMN\_MOUSE | PGH2\_HUMAN | PPA5\_RAT | GO:0050801 | 0.0145872129112353 | 47/3222 | 4/552 | 0.971623384795338 | 1 | F | F | F | F | monoatomic ion homeostasis | ANAG\_HUMAN | CERU\_RAT | PPT1\_BOVIN | PPT1\_HUMAN | GO:0023051 | 0.04252017380509 | 137/3222 | 16/552 | 0.972309188160721 | 1 | F | F | F | F | regulation of signaling | CARP1\_CANAL | CATH\_HUMAN | CEL2A\_PIG | DOPO\_HUMAN | G3I1H5\_CRIGR | GBA1\_HUMAN | LGMN\_MOUSE | LICH\_HUMAN | PA21B\_PIG | PA2GA\_HUMAN | PCP\_HUMAN | PGH2\_HUMAN | PPAP\_RAT | RENI\_RAT | TRFL\_BUBBU | TRFL\_HORSE | GO:0010243 | 0.0360024829298572 | 116/3222 | 13/552 | 0.973078092951814 | 1 | F | F | F | F | response to organonitrogen compound | AOC1\_HUMAN | CEL2A\_PIG | DOPO\_HUMAN | G3I1H5\_CRIGR | LGMN\_MOUSE | LICH\_HUMAN | LIPR2\_RAT | MAN12\_PENCI | PA21B\_PIG | PGH2\_HUMAN | PGPSA\_DROME | PPA5\_RAT | RENI\_RAT | GO:1901654 | 0.0121042830540037 | 39/3222 | 3/552 | 0.973917054505269 | 1 | F | F | F | F | response to ketone | GBA1\_HUMAN | LALBA\_BOVIN | LICH\_HUMAN | GO:0010646 | 0.042830540037244 | 138/3222 | 16/552 | 0.974554634432613 | 1 | F | F | F | F | regulation of cell communication | CARP1\_CANAL | CATH\_HUMAN | CEL2A\_PIG | DOPO\_HUMAN | G3I1H5\_CRIGR | GBA1\_HUMAN | LGMN\_MOUSE | LICH\_HUMAN | PA21B\_PIG | PA2GA\_HUMAN | PCP\_HUMAN | PGH2\_HUMAN | PPAP\_RAT | RENI\_RAT | TRFL\_BUBBU | TRFL\_HORSE | GO:0071396 | 0.00931098696461825 | 30/3222 | 2/552 | 0.974827700178236 | 1 | F | F | F | F | cellular response to lipid | ANG4\_MOUSE | ENPP2\_RAT | GO:0031328 | 0.0294847920546245 | 95/3222 | 10/552 | 0.975301172295659 | 1 | F | F | F | F | positive regulation of cellular biosynthetic process | CATH\_HUMAN | CHIA\_HUMAN | CHIL3\_MOUSE | GBA1\_HUMAN | GRASS\_DROME | KLK7\_HUMAN | KLK7\_MOUSE | PA21B\_PIG | PGH2\_HUMAN | PGPSA\_DROME | GO:0034660 | 0.0204841713221601 | 66/3222 | 6/552 | 0.979600845900094 | 1 | F | F | F | F | ncRNA metabolic process | ANG1\_BOVIN | ANG2\_MOUSE | ANG3\_MOUSE | ANG4\_MOUSE | ANGI\_MOUSE | Q5WRG2\_RAT | GO:0048513 | 0.0279329608938547 | 90/3222 | 9/552 | 0.980948363177338 | 1 | F | F | F | F | animal organ development | ANAG\_HUMAN | CATH\_HUMAN | CERU\_RAT | HYAL1\_HUMAN | LICH\_HUMAN | PPT1\_BOVIN | PPT1\_HUMAN | Q5WRG2\_RAT | RENI\_RAT | GO:0009611 | 0.00993171942892613 | 32/3222 | 2/552 | 0.981780271373198 | 1 | F | F | F | F | response to wounding | ANAG\_HUMAN | KLK8\_MOUSE | GO:0009967 | 0.0183116076970826 | 59/3222 | 5/552 | 0.98233261423242 | 1 | F | F | F | F | positive regulation of signal transduction | CATH\_HUMAN | LICH\_HUMAN | PA21B\_PIG | PA2GA\_HUMAN | PPAP\_RAT | GO:0006690 | 0.021104903786468 | 68/3222 | 6/552 | 0.983978179499085 | 1 | F | F | F | F | icosanoid metabolic process | CBPA1\_PIG | PA21B\_PIG | PA2GX\_HUMAN | PGH2\_HUMAN | PTGDS\_HUMAN | PTGDS\_MOUSE | GO:0009891 | 0.0310366232153942 | 100/3222 | 10/552 | 0.985197785443194 | 1 | F | F | F | F | positive regulation of biosynthetic process | CATH\_HUMAN | CHIA\_HUMAN | CHIL3\_MOUSE | GBA1\_HUMAN | GRASS\_DROME | KLK7\_HUMAN | KLK7\_MOUSE | PA21B\_PIG | PGH2\_HUMAN | PGPSA\_DROME | GO:0032870 | 0.010552451893234 | 34/3222 | 2/552 | 0.98685483926887 | 1 | F | F | F | F | cellular response to hormone stimulus | CATH\_HUMAN | PA21B\_PIG | GO:0007568 | 0.010552451893234 | 34/3222 | 2/552 | 0.98685483926887 | 1 | F | F | F | F | obsolete aging | CERU\_RAT | PGH2\_HUMAN | GO:0048522 | 0.0881440099317194 | 284/3222 | 36/552 | 0.987257436328763 | 1 | F | F | F | F | positive regulation of cellular process | ANG1\_BOVIN | ANG2\_MOUSE | ANG3\_MOUSE | ANG4\_MOUSE | ANGI\_MOUSE | ATLE\_CYCAE | CATD\_RAT | CATH\_HUMAN | CHIA\_HUMAN | CHIL3\_MOUSE | ENPP2\_HUMAN | ENPP2\_RAT | G3I1H5\_CRIGR | GBA1\_HUMAN | GRAA\_HUMAN | GRASS\_DROME | HYAL1\_HUMAN | KLK7\_HUMAN | KLK7\_MOUSE | LGMN\_MOUSE | LICH\_HUMAN | MMP1\_PIG | PA21B\_BOVIN | PA21B\_PIG | PA2GA\_HUMAN | PA2GX\_HUMAN | PGH2\_HUMAN | PGPSA\_DROME | PPA5\_RAT | PPAP\_RAT | PPT1\_BOVIN | PPT1\_HUMAN | PRTN3\_HUMAN | Q5WRG2\_RAT | TRFL\_BUBBU | TRFL\_HORSE | GO:0014070 | 0.0384854127870888 | 124/3222 | 13/552 | 0.987318492210418 | 1 | F | F | F | F | response to organic cyclic compound | ANAG\_HUMAN | AOC1\_HUMAN | CBPN\_HUMAN | ENPP2\_RAT | GBA1\_HUMAN | LALBA\_BOVIN | LICH\_HUMAN | LIPR2\_RAT | PGH2\_HUMAN | PPA5\_RAT | PTGDS\_HUMAN | PTGDS\_MOUSE | RENI\_RAT | GO:0097305 | 0.0164494103041589 | 53/3222 | 4/552 | 0.987406797835962 | 1 | F | F | F | F | response to alcohol | CHIC\_ARATH | LALBA\_BOVIN | LICH\_HUMAN | PPA5\_RAT | GO:0071345 | 0.0136561142147734 | 44/3222 | 3/552 | 0.987477214362931 | 1 | F | F | F | F | cellular response to cytokine stimulus | GBA1\_HUMAN | HYAL1\_HUMAN | PA2GX\_HUMAN | GO:1901361 | 0.0735567970204842 | 237/3222 | 29/552 | 0.987494824261991 | 1 | F | F | F | F | organic cyclic compound catabolic process | ADA2\_HUMAN | ANG4\_MOUSE | ASM3A\_HUMAN | ASM3A\_MOUSE | DNAS1\_HUMAN | DNSL3\_HUMAN | DOPO\_HUMAN | ECP\_HUMAN | ENDO2\_ARATH | GCE2\_MYCTT | GCE\_CERUI | GCE\_HYPJQ | LAC1\_MELAO | LAC1\_TRAMX | LAC2\_TRAVE | LIG2\_PHACH | LIG4\_PHACH | LIG8\_PHACH | NUP1\_PENCI | NUS1\_ASPOR | PEM1\_PHACH | Q0KFV0\_SOLLC | Q60FD2\_9APHY | RNAS6\_HUMAN | RNLE\_SOLLC | RNT2\_HUMAN | V5NTD\_NAJAT | VPL1\_PLEER | VPL2\_PLEER | GO:0016070 | 0.031657355679702 | 102/3222 | 10/552 | 0.988012442395676 | 1 | F | F | F | F | RNA metabolic process | ANG1\_BOVIN | ANG2\_MOUSE | ANG3\_MOUSE | ANG4\_MOUSE | ANGI\_MOUSE | ECP\_HUMAN | Q5WRG2\_RAT | RNAS6\_HUMAN | RNLE\_SOLLC | RNT2\_HUMAN | GO:0048518 | 0.0990068280571074 | 319/3222 | 41/552 | 0.988626194934696 | 1 | F | F | F | F | positive regulation of biological process | AGAL\_ORYSJ | ANAG\_HUMAN | ANG1\_BOVIN | ANG2\_MOUSE | ANG3\_MOUSE | ANG4\_MOUSE | ANGI\_MOUSE | ATLE\_CYCAE | CARP1\_CANAL | CATD\_RAT | CATH\_HUMAN | CFAD\_MOUSE | CHIA\_HUMAN | CHIL3\_MOUSE | DOPO\_HUMAN | ENPP2\_HUMAN | ENPP2\_RAT | G3I1H5\_CRIGR | GBA1\_HUMAN | GRAA\_HUMAN | GRASS\_DROME | HYAL1\_HUMAN | KLK7\_HUMAN | KLK7\_MOUSE | LGMN\_MOUSE | LICH\_HUMAN | MMP1\_PIG | PA21B\_BOVIN | PA21B\_PIG | PA2GA\_HUMAN | PA2GX\_HUMAN | PGH2\_HUMAN | PGPSA\_DROME | PPA5\_RAT | PPAP\_RAT | PPT1\_BOVIN | PPT1\_HUMAN | PRTN3\_HUMAN | Q5WRG2\_RAT | TRFL\_BUBBU | TRFL\_HORSE | GO:0070887 | 0.0676598386095593 | 218/3222 | 26/552 | 0.988914359936416 | 1 | F | F | F | F | cellular response to chemical stimulus | ANAG\_HUMAN | ANG2\_MOUSE | ANG4\_MOUSE | AOC1\_HUMAN | CATH\_HUMAN | DABA\_PSEMU | ENPP2\_RAT | G3I1H5\_CRIGR | GBA1\_HUMAN | GPX5\_HUMAN | HYAL1\_HUMAN | LGMN\_MOUSE | LIG2\_PHACH | LIG4\_PHACH | LIG8\_PHACH | PA21B\_PIG | PA2GX\_HUMAN | PEM1\_PHACH | PER\_ARTRA | PER\_COPCI | PGH2\_HUMAN | Q60FD2\_9APHY | Q96X16\_PICPA | RENI\_RAT | VPL1\_PLEER | VPL2\_PLEER | GO:0007584 | 0.0139664804469274 | 45/3222 | 3/552 | 0.989216818198381 | 1 | F | F | F | F | response to nutrient | CERU\_RAT | PGH2\_HUMAN | PPA5\_RAT | GO:0045935 | 0.0139664804469274 | 45/3222 | 3/552 | 0.989216818198381 | 1 | F | F | F | F | positive regulation of nucleobase-containing compound metabolic process | ANGI\_MOUSE | PA21B\_PIG | Q5WRG2\_RAT | GO:1902531 | 0.0226567349472377 | 73/3222 | 6/552 | 0.991386274499256 | 1 | F | F | F | F | regulation of intracellular signal transduction | CARP1\_CANAL | GBA1\_HUMAN | PA21B\_PIG | PA2GA\_HUMAN | PGH2\_HUMAN | RENI\_RAT | GO:1901698 | 0.0397268777157045 | 128/3222 | 13/552 | 0.991458596668155 | 1 | F | F | F | F | response to nitrogen compound | AOC1\_HUMAN | CEL2A\_PIG | DOPO\_HUMAN | G3I1H5\_CRIGR | LGMN\_MOUSE | LICH\_HUMAN | LIPR2\_RAT | MAN12\_PENCI | PA21B\_PIG | PGH2\_HUMAN | PGPSA\_DROME | PPA5\_RAT | RENI\_RAT | GO:0042592 | 0.0490378646803228 | 158/3222 | 17/552 | 0.99185192079714 | 1 | F | F | F | F | homeostatic process | ANAG\_HUMAN | ANG2\_MOUSE | CATH\_HUMAN | CERU\_RAT | DOPO\_HUMAN | GBA1\_HUMAN | LICH\_HUMAN | PA2GA\_HUMAN | PA2GX\_HUMAN | PCP\_HUMAN | PGH2\_HUMAN | PPA5\_HUMAN | PPA5\_PIG | PPA5\_RAT | PPT1\_BOVIN | PPT1\_HUMAN | TPP1\_HUMAN | GO:1901701 | 0.0304158907510863 | 98/3222 | 9/552 | 0.992069715617651 | 1 | F | F | F | F | cellular response to oxygen-containing compound | ANG2\_MOUSE | ANG4\_MOUSE | AOC1\_HUMAN | DABA\_PSEMU | ENPP2\_RAT | G3I1H5\_CRIGR | LGMN\_MOUSE | PA21B\_PIG | PGH2\_HUMAN | GO:0018193 | 0.0304158907510863 | 98/3222 | 9/552 | 0.992069715617651 | 1 | F | F | F | F | peptidyl-amino acid modification | A0A0R3QSA7\_9BILA | A0A3S5H5N2\_LEIDO | CYP5\_CAEEL | O81226\_CARPA | Q5B038\_EMENI | QPCT1\_DROME | QPCT2\_DROME | QPCT\_IXOSC | QPCT\_MOUSE | GO:0050794 | 0.167597765363128 | 540/3222 | 74/552 | 0.992408741741023 | 1 | F | F | F | F | regulation of cellular process | AGAL\_HUMAN | ANAG\_HUMAN | ANG1\_BOVIN | ANG2\_MOUSE | ANG3\_MOUSE | ANG4\_MOUSE | ANGI\_MOUSE | ATLE\_CYCAE | CARP1\_CANAL | CATD\_RAT | CATH\_HUMAN | CEL2A\_PIG | CFAD\_MOUSE | CHIA\_HUMAN | CHIL3\_MOUSE | D6XHE1\_TRYB2 | DNAS1\_HUMAN | DNSL3\_HUMAN | DOPO\_HUMAN | E0CX04\_MOMBA | ENPP2\_HUMAN | ENPP2\_RAT | G3I1H5\_CRIGR | GBA1\_HUMAN | GILT\_MOUSE | GRAA\_HUMAN | GRAC\_MOUSE | GRASS\_DROME | HYAL1\_HUMAN | KLK7\_HUMAN | KLK7\_MOUSE | KLK8\_MOUSE | LGMN\_MOUSE | LICH\_HUMAN | MMP1\_PIG | O04358\_IRIHO | OFUT1\_CAEEL | OXLA\_BOTAT | PA21B\_BOVIN | PA21B\_PIG | PA2GA\_HUMAN | PA2GE\_HUMAN | PA2GX\_HUMAN | PCP\_HUMAN | PGH2\_HUMAN | PGPSA\_DROME | PGRP1\_CAMDR | PPA5\_HUMAN | PPA5\_RAT | PPAP\_RAT | PPT1\_BOVIN | PPT1\_HUMAN | PRTN3\_HUMAN | PTGDS\_HUMAN | PTGDS\_MOUSE | Q2QEH4\_SAPOF | Q5WRG2\_RAT | Q6R7Z5\_9TRYP | Q94BW3\_CINCA | RENI\_RAT | RIP0\_DIACA | RIP1\_BRYDI | RIP1\_HORVU | RIP1\_MOMCH | RIP1\_PHYAM | RIP2\_PHYAM | RIP3\_MOMCH | RIPA\_PHYAM | RIPG\_SURMU | RIPL1\_PHYDI | RIPL2\_PHYDI | RIPT\_TRIKI | TRFL\_BUBBU | TRFL\_HORSE | GO:0006996 | 0.0229671011793917 | 74/3222 | 6/552 | 0.992412216816226 | 1 | F | F | F | F | organelle organization | ANAG\_HUMAN | CATD\_RAT | GBA1\_HUMAN | LICH\_HUMAN | PPAP\_RAT | TPP1\_HUMAN | GO:0090304 | 0.0471756672873991 | 152/3222 | 16/552 | 0.992793491643757 | 1 | F | F | F | F | nucleic acid metabolic process | ANG1\_BOVIN | ANG2\_MOUSE | ANG3\_MOUSE | ANG4\_MOUSE | ANGI\_MOUSE | DNAS1\_HUMAN | DNSL3\_HUMAN | ECP\_HUMAN | ENDO2\_ARATH | NUP1\_PENCI | NUS1\_ASPOR | Q0KFV0\_SOLLC | Q5WRG2\_RAT | RNAS6\_HUMAN | RNLE\_SOLLC | RNT2\_HUMAN | GO:0015031 | 0.0117939168218498 | 38/3222 | 2/552 | 0.993215394466381 | 1 | F | F | F | F | protein transport | PPT1\_BOVIN | PPT1\_HUMAN | GO:0048878 | 0.0310366232153942 | 100/3222 | 9/552 | 0.993683100507176 | 1 | F | F | F | F | chemical homeostasis | ANAG\_HUMAN | ANG2\_MOUSE | CATH\_HUMAN | CERU\_RAT | DOPO\_HUMAN | PA2GX\_HUMAN | PCP\_HUMAN | PPT1\_BOVIN | PPT1\_HUMAN | GO:0009628 | 0.0453134698944755 | 146/3222 | 15/552 | 0.993699122979255 | 1 | F | F | F | F | response to abiotic stimulus | ANG1\_BOVIN | ANG2\_MOUSE | ANG3\_MOUSE | ANG4\_MOUSE | ANGI\_MOUSE | CHIC\_ARATH | DOPO\_HUMAN | G3I1H5\_CRIGR | GBA1\_HUMAN | HYAL1\_HUMAN | LGMN\_MOUSE | MMP1\_PIG | PGH2\_HUMAN | PPA5\_RAT | Q5WRG2\_RAT | GO:0016311 | 0.0180012414649286 | 58/3222 | 4/552 | 0.993766909024898 | 1 | F | F | F | F | dephosphorylation | A4GX63\_TOXGO | PPA5\_HUMAN | PPA5\_RAT | PPAP\_RAT | GO:0055082 | 0.0180012414649286 | 58/3222 | 4/552 | 0.993766909024898 | 1 | F | F | F | F | intracellular chemical homeostasis | ANG2\_MOUSE | CERU\_RAT | PPT1\_BOVIN | PPT1\_HUMAN | GO:0051649 | 0.0152079453755431 | 49/3222 | 3/552 | 0.99412069367276 | 1 | F | F | F | F | establishment of localization in cell | PPT1\_HUMAN | PTGDS\_HUMAN | PTGDS\_MOUSE | GO:0008104 | 0.0152079453755431 | 49/3222 | 3/552 | 0.99412069367276 | 1 | F | F | F | F | protein localization | PPT1\_BOVIN | PPT1\_HUMAN | TPP1\_HUMAN | GO:0006633 | 0.0235878336436996 | 76/3222 | 6/552 | 0.994127015958232 | 1 | F | F | F | F | fatty acid biosynthetic process | PA21B\_BOVIN | PA21B\_PIG | PA2GX\_HUMAN | PGH2\_HUMAN | PTGDS\_HUMAN | PTGDS\_MOUSE | GO:0070727 | 0.0155183116076971 | 50/3222 | 3/552 | 0.994957685857153 | 1 | F | F | F | F | cellular macromolecule localization | PPT1\_BOVIN | PPT1\_HUMAN | TPP1\_HUMAN | GO:0033036 | 0.0155183116076971 | 50/3222 | 3/552 | 0.994957685857153 | 1 | F | F | F | F | macromolecule localization | PPT1\_BOVIN | PPT1\_HUMAN | TPP1\_HUMAN | GO:0098771 | 0.0124146492861577 | 40/3222 | 2/552 | 0.99514415990067 | 1 | F | F | F | F | inorganic ion homeostasis | ANAG\_HUMAN | CERU\_RAT | GO:0035383 | 0.0127250155183116 | 41/3222 | 2/552 | 0.995895479207392 | 1 | F | F | F | F | thioester metabolic process | PPT1\_HUMAN | PPT2\_HUMAN | GO:0006637 | 0.0127250155183116 | 41/3222 | 2/552 | 0.995895479207392 | 1 | F | F | F | F | acyl-CoA metabolic process | PPT1\_HUMAN | PPT2\_HUMAN | GO:0033559 | 0.0217256362507759 | 70/3222 | 5/552 | 0.995897645536667 | 1 | F | F | F | F | unsaturated fatty acid metabolic process | MNLOX\_MAGO7 | PA2GX\_HUMAN | PGH2\_HUMAN | PTGDS\_HUMAN | PTGDS\_MOUSE | GO:0006760 | 0.0130353817504655 | 42/3222 | 2/552 | 0.996532429577509 | 1 | F | F | F | F | folic acid-containing compound metabolic process | GGH\_HUMAN | Q6NY42\_DANRE | GO:0031667 | 0.0304158907510863 | 98/3222 | 8/552 | 0.996949280346975 | 1 | F | F | F | F | response to nutrient levels | AOC1\_HUMAN | CATD\_RAT | CERU\_RAT | DABA\_PSEMU | GBA1\_HUMAN | LIPR2\_RAT | PGH2\_HUMAN | PPA5\_RAT | GO:1902652 | 0.0195530726256983 | 63/3222 | 4/552 | 0.99697715655537 | 1 | F | F | F | F | secondary alcohol metabolic process | GBA1\_HUMAN | IDH\_OSTTA | LICH\_HUMAN | LIP3\_DIURU | GO:0001676 | 0.0198634388578523 | 64/3222 | 4/552 | 0.997390253852203 | 1 | F | F | F | F | long-chain fatty acid metabolic process | MNLOX\_MAGO7 | PA2GX\_HUMAN | PGH2\_HUMAN | PTGDS\_HUMAN | GO:0043933 | 0.0282433271260087 | 91/3222 | 7/552 | 0.997448554977146 | 1 | F | F | F | F | protein-containing complex organization | ANG1\_BOVIN | ANGI\_MOUSE | PA2GA\_HUMAN | PA2GE\_HUMAN | PA2GX\_HUMAN | Q5WRG2\_RAT | TTHY\_CHICK | GO:0045184 | 0.0136561142147734 | 44/3222 | 2/552 | 0.997528961614757 | 1 | F | F | F | F | establishment of protein localization | PPT1\_BOVIN | PPT1\_HUMAN | GO:0080135 | 0.0136561142147734 | 44/3222 | 2/552 | 0.997528961614757 | 1 | F | F | F | F | regulation of cellular response to stress | KLK8\_MOUSE | PGH2\_HUMAN | GO:1902533 | 0.0136561142147734 | 44/3222 | 2/552 | 0.997528961614757 | 1 | F | F | F | F | positive regulation of intracellular signal transduction | PA21B\_PIG | PA2GA\_HUMAN | GO:0044270 | 0.0484171322160149 | 156/3222 | 15/552 | 0.997709911961207 | 1 | F | F | F | F | cellular nitrogen compound catabolic process | ADA2\_HUMAN | ANG4\_MOUSE | ASM3A\_HUMAN | ASM3A\_MOUSE | DNAS1\_HUMAN | DNSL3\_HUMAN | ECP\_HUMAN | ENDO2\_ARATH | NUP1\_PENCI | NUS1\_ASPOR | Q0KFV0\_SOLLC | RNAS6\_HUMAN | RNLE\_SOLLC | RNT2\_HUMAN | V5NTD\_NAJAT | GO:0009410 | 0.0173805090006207 | 56/3222 | 3/552 | 0.998022219177465 | 1 | F | F | F | F | response to xenobiotic stimulus | LICH\_HUMAN | PGH2\_HUMAN | RENI\_RAT | GO:0046700 | 0.0490378646803228 | 158/3222 | 15/552 | 0.998143554240377 | 1 | F | F | F | F | heterocycle catabolic process | ADA2\_HUMAN | ANG4\_MOUSE | ASM3A\_HUMAN | ASM3A\_MOUSE | DNAS1\_HUMAN | DNSL3\_HUMAN | ECP\_HUMAN | ENDO2\_ARATH | NUP1\_PENCI | NUS1\_ASPOR | Q0KFV0\_SOLLC | RNAS6\_HUMAN | RNLE\_SOLLC | RNT2\_HUMAN | V5NTD\_NAJAT | GO:0046128 | 0.0142768466790813 | 46/3222 | 2/552 | 0.998242519865831 | 1 | F | F | F | F | purine ribonucleoside metabolic process | ADA2\_HUMAN | PPAP\_RAT | GO:0009112 | 0.0207945375543141 | 67/3222 | 4/552 | 0.998327482655787 | 1 | F | F | F | F | nucleobase metabolic process | PPAP\_RAT | TTHY\_CHICK | TTHY\_MOUSE | TTHY\_RAT | GO:0042445 | 0.026691495965239 | 86/3222 | 6/552 | 0.998442585813356 | 1 | F | F | F | F | hormone metabolic process | ANAG\_HUMAN | CKX1\_MAIZE | EST6\_DROME | RENI\_RAT | TTHY\_CHICK | TTHY\_RAT | GO:0010033 | 0.0887647423960273 | 286/3222 | 32/552 | 0.998687542265761 | 1 | F | F | F | F | response to organic substance | ANAG\_HUMAN | ANG2\_MOUSE | ANG3\_MOUSE | ANG4\_MOUSE | ANGI\_MOUSE | AOC1\_HUMAN | CATH\_HUMAN | CBPN\_HUMAN | CEL2A\_PIG | CHIC\_ARATH | DABA\_PSEMU | DOPO\_HUMAN | ENPP2\_RAT | G3I1H5\_CRIGR | GBA1\_HUMAN | GPX3\_HUMAN | HYAL1\_HUMAN | LALBA\_BOVIN | LGMN\_MOUSE | LICH\_HUMAN | LIPR2\_RAT | MAN12\_PENCI | PA21B\_PIG | PA2GX\_HUMAN | PGH2\_HUMAN | PGPSA\_DROME | PPA5\_HUMAN | PPA5\_RAT | PTGDS\_HUMAN | PTGDS\_MOUSE | Q5WRG2\_RAT | RENI\_RAT | GO:0071705 | 0.0148975791433892 | 48/3222 | 2/552 | 0.998752288534322 | 1 | F | F | F | F | nitrogen compound transport | PPT1\_BOVIN | PPT1\_HUMAN | GO:0065003 | 0.021415270018622 | 69/3222 | 4/552 | 0.998760792545668 | 1 | F | F | F | F | protein-containing complex assembly | ANG1\_BOVIN | ANGI\_MOUSE | Q5WRG2\_RAT | TTHY\_CHICK | GO:0009894 | 0.0183116076970826 | 59/3222 | 3/552 | 0.998771701992527 | 1 | F | F | F | F | regulation of catabolic process | ANGI\_MOUSE | GBA1\_HUMAN | Q5WRG2\_RAT | GO:0009991 | 0.0328988206083178 | 106/3222 | 8/552 | 0.998885283784028 | 1 | F | F | F | F | response to extracellular stimulus | AOC1\_HUMAN | CATD\_RAT | CERU\_RAT | DABA\_PSEMU | GBA1\_HUMAN | LIPR2\_RAT | PGH2\_HUMAN | PPA5\_RAT | GO:0032502 | 0.0915580384854128 | 295/3222 | 33/552 | 0.998888648286842 | 1 | F | F | F | F | developmental process | ADPG2\_ARATH | AGAL\_ORYSJ | ANAG\_HUMAN | ANG1\_BOVIN | ANG2\_MOUSE | ANG3\_MOUSE | ANG4\_MOUSE | ANGI\_MOUSE | CATH\_HUMAN | CERU\_RAT | DNSL3\_HUMAN | GBA1\_HUMAN | HYAL1\_HUMAN | KLK7\_HUMAN | KLK8\_MOUSE | LICH\_HUMAN | PA2GX\_HUMAN | PCP\_HUMAN | PER53\_ARATH | PGH2\_HUMAN | PPA5\_HUMAN | PPA5\_RAT | PPT1\_BOVIN | PPT1\_HUMAN | PRTN3\_HUMAN | Q55FE6\_DICDI | Q5WRG2\_RAT | RENI\_RAT | RNSL3\_DANRE | TPP1\_HUMAN | TRFL\_BUBBU | TRFL\_HORSE | TTHY\_CHICK | GO:0035556 | 0.0217256362507759 | 70/3222 | 4/552 | 0.998934316607786 | 1 | F | F | F | F | intracellular signal transduction | ANG2\_MOUSE | CATH\_HUMAN | LICH\_HUMAN | PA21B\_PIG | GO:0016125 | 0.0189323401613904 | 61/3222 | 3/552 | 0.999108396956831 | 1 | F | F | F | F | sterol metabolic process | GBA1\_HUMAN | LICH\_HUMAN | LIP3\_DIURU | GO:1901292 | 0.0189323401613904 | 61/3222 | 3/552 | 0.999108396956831 | 1 | F | F | F | F | nucleoside phosphate catabolic process | ASM3A\_HUMAN | ASM3A\_MOUSE | V5NTD\_NAJAT | GO:0044248 | 0.176288019863439 | 568/3222 | 73/552 | 0.999118373042923 | 1 | F | F | F | F | cellular catabolic process | A0A087WNH2\_FICBE | A0A0A0Y4H8\_TRAFO | A0A1S4NYF8\_PANVG | A0A3L6SKP5\_PANMI | ADA2\_HUMAN | AGAL\_HUMAN | ANAG\_HUMAN | ANG4\_MOUSE | APO1\_CYCAE | ASM3A\_HUMAN | ASM3A\_MOUSE | BGLR\_HUMAN | CAT3\_NEUCR | CATH\_HUMAN | CBPN\_HUMAN | D1MPT2\_ROYRE | DNAS1\_HUMAN | DNSL3\_HUMAN | DOPO\_HUMAN | ECP\_HUMAN | ENDO2\_ARATH | ENPP2\_HUMAN | ENPP2\_RAT | FUCO\_HUMAN | G3I1H5\_CRIGR | GBA1\_HUMAN | GCE2\_MYCTT | GCE\_CERUI | GCE\_HYPJQ | GPX3\_HUMAN | HYAL1\_HUMAN | IDUA\_HUMAN | K7N5L9\_RAPSA | KATG2\_MAGO7 | LAC1\_MELAO | LAC1\_TRAMX | LAC2\_TRAVE | LGMN\_MOUSE | LIG2\_PHACH | LIG4\_PHACH | LIG8\_PHACH | LIPR2\_HUMAN | LIPR2\_RAT | NAGAB\_HUMAN | NUP1\_PENCI | NUS1\_ASPOR | O22443\_SOYBN | PA2GX\_HUMAN | PAG15\_HUMAN | PEM1\_PHACH | PER1A\_ARMRU | PER1\_ARAHY | PER1\_SORBI | PER53\_ARATH | PER59\_ARATH | PERL\_BOVIN | PERL\_BUBBU | PERL\_CAPHI | PER\_ARTRA | PER\_COPCI | POXA\_DICDI | PPT1\_HUMAN | Q0KFV0\_SOLLC | Q40069\_HORVU | Q60FD2\_9APHY | RNAS6\_HUMAN | RNLE\_SOLLC | RNT2\_HUMAN | SIA\_ASPFU | TPP1\_HUMAN | V5NTD\_NAJAT | VPL1\_PLEER | VPL2\_PLEER | GO:0051641 | 0.0192427063935444 | 62/3222 | 3/552 | 0.999240968439158 | 1 | F | F | F | F | cellular localization | PPT1\_BOVIN | PPT1\_HUMAN | TPP1\_HUMAN | GO:0072330 | 0.0391061452513966 | 126/3222 | 10/552 | 0.999245103683774 | 1 | F | F | F | F | monocarboxylic acid biosynthetic process | MDLA\_PENCA | MDLA\_PENCY | PA21B\_BOVIN | PA21B\_PIG | PA2GX\_HUMAN | PGH2\_HUMAN | PTGDS\_HUMAN | PTGDS\_MOUSE | Q7LST4\_PENEN | W6Q990\_PENRF | GO:0042278 | 0.016139044072005 | 52/3222 | 2/552 | 0.999374259974737 | 1 | F | F | F | F | purine nucleoside metabolic process | ADA2\_HUMAN | PPAP\_RAT | GO:0010817 | 0.031657355679702 | 102/3222 | 7/552 | 0.999404460066425 | 1 | F | F | F | F | regulation of hormone levels | ANAG\_HUMAN | CEL2A\_PIG | CKX1\_MAIZE | EST6\_DROME | RENI\_RAT | TTHY\_CHICK | TTHY\_RAT | GO:0050789 | 0.186840471756673 | 602/3222 | 77/552 | 0.999486438433699 | 1 | F | F | F | F | regulation of biological process | AGAL\_HUMAN | AGAL\_ORYSJ | ANAG\_HUMAN | ANG1\_BOVIN | ANG2\_MOUSE | ANG3\_MOUSE | ANG4\_MOUSE | ANGI\_MOUSE | AOAH\_MOUSE | ATLE\_CYCAE | CARP1\_CANAL | CATD\_RAT | CATH\_HUMAN | CEL2A\_PIG | CFAD\_MOUSE | CHIA\_HUMAN | CHIL3\_MOUSE | D6XHE1\_TRYB2 | DNAS1\_HUMAN | DNSL3\_HUMAN | DOPO\_HUMAN | E0CX04\_MOMBA | ENPP2\_HUMAN | ENPP2\_RAT | EST6\_DROME | G3I1H5\_CRIGR | GBA1\_HUMAN | GILT\_MOUSE | GRAA\_HUMAN | GRAC\_MOUSE | GRASS\_DROME | HYAL1\_HUMAN | KLK7\_HUMAN | KLK7\_MOUSE | KLK8\_MOUSE | LGMN\_MOUSE | LICH\_HUMAN | MMP1\_PIG | O04358\_IRIHO | OFUT1\_CAEEL | OXLA\_BOTAT | PA21B\_BOVIN | PA21B\_PIG | PA2GA\_HUMAN | PA2GE\_HUMAN | PA2GX\_HUMAN | PCP\_HUMAN | PGH2\_HUMAN | PGPSA\_DROME | PGRP1\_CAMDR | PPA5\_HUMAN | PPA5\_RAT | PPAP\_RAT | PPT1\_BOVIN | PPT1\_HUMAN | PRTN3\_HUMAN | PTGDS\_HUMAN | PTGDS\_MOUSE | Q2QEH4\_SAPOF | Q5WRG2\_RAT | Q6R7Z5\_9TRYP | Q94BW3\_CINCA | RENI\_RAT | RIP0\_DIACA | RIP1\_BRYDI | RIP1\_HORVU | RIP1\_MOMCH | RIP1\_PHYAM | RIP2\_PHYAM | RIP3\_MOMCH | RIPA\_PHYAM | RIPG\_SURMU | RIPL1\_PHYDI | RIPL2\_PHYDI | RIPT\_TRIKI | TRFL\_BUBBU | TRFL\_HORSE | GO:1901657 | 0.0350713842333954 | 113/3222 | 8/552 | 0.999553307196474 | 1 | F | F | F | F | glycosyl compound metabolic process | ADA2\_HUMAN | AGAL\_HUMAN | BGLR\_HUMAN | FUCO\_HUMAN | GBA1\_HUMAN | NAGAB\_CHICK | NAGAB\_HUMAN | PPAP\_RAT | GO:0018958 | 0.0167597765363128 | 54/3222 | 2/552 | 0.999557886059759 | 1 | F | F | F | F | phenol-containing compound metabolic process | DOPO\_HUMAN | TTHY\_RAT | GO:0006468 | 0.0167597765363128 | 54/3222 | 2/552 | 0.999557886059759 | 1 | F | F | F | F | protein phosphorylation | ANG2\_MOUSE | Q06AK3\_TOXGO | GO:0010035 | 0.0384854127870888 | 124/3222 | 9/552 | 0.999668792475968 | 1 | F | F | F | F | response to inorganic substance | AOC1\_HUMAN | CERU\_RAT | DABA\_PSEMU | ENPP2\_RAT | G3I1H5\_CRIGR | LGMN\_MOUSE | PGH2\_HUMAN | PPA5\_RAT | Q96X16\_PICPA | GO:0048856 | 0.0589695841092489 | 190/3222 | 17/552 | 0.999687685231321 | 1 | F | F | F | F | anatomical structure development | ANAG\_HUMAN | ANG1\_BOVIN | ANGI\_MOUSE | CATH\_HUMAN | CERU\_RAT | GBA1\_HUMAN | HYAL1\_HUMAN | KLK7\_HUMAN | LICH\_HUMAN | PER53\_ARATH | PGH2\_HUMAN | PPT1\_BOVIN | PPT1\_HUMAN | Q55FE6\_DICDI | Q5WRG2\_RAT | RENI\_RAT | TPP1\_HUMAN | GO:1901700 | 0.0732464307883302 | 236/3222 | 23/552 | 0.999687966148876 | 1 | F | F | F | F | response to oxygen-containing compound | ANAG\_HUMAN | ANG2\_MOUSE | ANG4\_MOUSE | AOC1\_HUMAN | CATH\_HUMAN | CEL2A\_PIG | CHIC\_ARATH | DABA\_PSEMU | ENPP2\_RAT | G3I1H5\_CRIGR | GBA1\_HUMAN | GPX3\_HUMAN | HYAL1\_HUMAN | LALBA\_BOVIN | LGMN\_MOUSE | LICH\_HUMAN | LIPR2\_RAT | PA21B\_PIG | PGH2\_HUMAN | PGPSA\_DROME | PPA5\_HUMAN | PPA5\_RAT | RENI\_RAT | GO:0042558 | 0.0176908752327747 | 57/3222 | 2/552 | 0.999738123016271 | 1 | F | F | F | F | pteridine-containing compound metabolic process | GGH\_HUMAN | Q6NY42\_DANRE | GO:0033554 | 0.0599006828057107 | 193/3222 | 17/552 | 0.999776413222714 | 1 | F | F | F | F | cellular response to stress | ANAG\_HUMAN | AOC1\_HUMAN | DABA\_PSEMU | GBA1\_HUMAN | GPX5\_HUMAN | LIG2\_PHACH | LIG4\_PHACH | LIG8\_PHACH | MAN12\_PENCI | PEM1\_PHACH | PER\_ARTRA | PER\_COPCI | PGH2\_HUMAN | PPA5\_RAT | Q60FD2\_9APHY | VPL1\_PLEER | VPL2\_PLEER | GO:0009308 | 0.0313469894475481 | 101/3222 | 6/552 | 0.999811797854293 | 1 | F | F | F | F | amine metabolic process | AOC1\_HUMAN | AOCX\_BOVIN | CKX1\_MAIZE | DOPO\_HUMAN | Q5B038\_EMENI | Q96X16\_PICPA | GO:0009119 | 0.0183116076970826 | 59/3222 | 2/552 | 0.999815602990515 | 1 | F | F | F | F | ribonucleoside metabolic process | ADA2\_HUMAN | PPAP\_RAT | GO:0065007 | 0.214463066418374 | 691/3222 | 88/552 | 0.999848472177248 | 1 | F | F | F | F | biological regulation | AGAL\_HUMAN | AGAL\_ORYSJ | ANAG\_HUMAN | ANG1\_BOVIN | ANG2\_MOUSE | ANG3\_MOUSE | ANG4\_MOUSE | ANGI\_MOUSE | AOAH\_MOUSE | AOCX\_BOVIN | ATLE\_CYCAE | CARP1\_CANAL | CATD\_RAT | CATH\_HUMAN | CEL2A\_PIG | CERU\_RAT | CFAD\_MOUSE | CHIA\_HUMAN | CHIL3\_MOUSE | CKX1\_MAIZE | D6XHE1\_TRYB2 | DNAS1\_HUMAN | DNSL3\_HUMAN | DOPO\_HUMAN | E0CX04\_MOMBA | EGFB2\_MOUSE | ENPP2\_HUMAN | ENPP2\_RAT | EST6\_DROME | G3I1H5\_CRIGR | GBA1\_HUMAN | GILT\_MOUSE | GRAA\_HUMAN | GRAC\_MOUSE | GRASS\_DROME | HYAL1\_HUMAN | KLK1\_HUMAN | KLK7\_HUMAN | KLK7\_MOUSE | KLK8\_MOUSE | LGMN\_MOUSE | LICH\_HUMAN | MMP1\_PIG | NP1\_RHOPR | NP2\_RHOPR | NP4\_RHOPR | O04358\_IRIHO | OFUT1\_CAEEL | OXLA\_BOTAT | PA21B\_BOVIN | PA21B\_PIG | PA2A\_BOTJR | PA2GA\_HUMAN | PA2GE\_HUMAN | PA2GX\_HUMAN | PCP\_HUMAN | PGH2\_HUMAN | PGPSA\_DROME | PGRP1\_CAMDR | PPA5\_HUMAN | PPA5\_RAT | PPAP\_RAT | PPT1\_BOVIN | PPT1\_HUMAN | PRTN3\_HUMAN | PTGDS\_HUMAN | PTGDS\_MOUSE | Q2QEH4\_SAPOF | Q5WRG2\_RAT | Q6R7Z5\_9TRYP | Q94BW3\_CINCA | RENI\_RAT | RIP0\_DIACA | RIP1\_BRYDI | RIP1\_HORVU | RIP1\_MOMCH | RIP1\_PHYAM | RIP2\_PHYAM | RIP3\_MOMCH | RIPA\_PHYAM | RIPG\_SURMU | RIPL1\_PHYDI | RIPL2\_PHYDI | RIPT\_TRIKI | TRFL\_BUBBU | TRFL\_HORSE | TTHY\_CHICK | TTHY\_RAT | GO:0043604 | 0.0223463687150838 | 72/3222 | 3/552 | 0.999852173910369 | 1 | F | F | F | F | amide biosynthetic process | GBA1\_HUMAN | PPT1\_HUMAN | PPT2\_HUMAN | GO:0022607 | 0.0288640595903166 | 93/3222 | 5/552 | 0.99985365595905 | 1 | F | F | F | F | cellular component assembly | ANG1\_BOVIN | ANGI\_MOUSE | CATD\_RAT | Q5WRG2\_RAT | TTHY\_CHICK | GO:0120254 | 0.0288640595903166 | 93/3222 | 5/552 | 0.99985365595905 | 1 | F | F | F | F | olefinic compound metabolic process | MNLOX\_MAGO7 | PA2GX\_HUMAN | PGH2\_HUMAN | PTGDS\_HUMAN | TTHY\_CHICK | GO:0033875 | 0.0189323401613904 | 61/3222 | 2/552 | 0.99987032142011 | 1 | F | F | F | F | ribonucleoside bisphosphate metabolic process | PPT1\_HUMAN | PPT2\_HUMAN | GO:0033865 | 0.0189323401613904 | 61/3222 | 2/552 | 0.99987032142011 | 1 | F | F | F | F | nucleoside bisphosphate metabolic process | PPT1\_HUMAN | PPT2\_HUMAN | GO:0034032 | 0.0189323401613904 | 61/3222 | 2/552 | 0.99987032142011 | 1 | F | F | F | F | purine nucleoside bisphosphate metabolic process | PPT1\_HUMAN | PPT2\_HUMAN | GO:0051252 | 0.0260707635009311 | 84/3222 | 4/552 | 0.999878238059183 | 1 | F | F | F | F | regulation of RNA metabolic process | ANGI\_MOUSE | PA21B\_PIG | PA2GX\_HUMAN | Q5WRG2\_RAT | GO:0006355 | 0.0201738050900062 | 65/3222 | 2/552 | 0.999936089873651 | 1 | F | F | F | F | regulation of DNA-templated transcription | PA21B\_PIG | PA2GX\_HUMAN | GO:2001141 | 0.0207945375543141 | 67/3222 | 2/552 | 0.999955207941136 | 1 | F | F | F | F | regulation of RNA biosynthetic process | PA21B\_PIG | PA2GX\_HUMAN | GO:0019637 | 0.180633147113594 | 582/3222 | 69/552 | 0.9999574224477 | 1 | F | F | F | F | organophosphate metabolic process | A0A1L8D5Z7\_BOTAT | ASM3A\_HUMAN | ASM3A\_MOUSE | ENPP2\_HUMAN | ENPP2\_RAT | I1SB18\_VIPAE | IDH\_OSTTA | LICH\_HUMAN | LIPR2\_HUMAN | LIPR2\_RAT | PA21B\_BOVIN | PA21B\_PIG | PA2A1\_BUNCE | PA2A1\_ECHCA | PA2A1\_NAJAT | PA2A1\_OPHHA | PA2A2\_NAJNA | PA2A2\_OPHHA | PA2A2\_TROCA | PA2A4\_NAJSG | PA2A5\_TRIST | PA2A7\_GLOHA | PA2A\_BOTJR | PA2A\_CROAT | PA2A\_DEIAC | PA2A\_GLOHA | PA2A\_NAJAT | PA2B1\_AGKPI | PA2B2\_BOTJR | PA2B2\_PROFL | PA2B3\_BOTAS | PA2B3\_BUNCE | PA2B5\_BUNCE | PA2B5\_NOTSC | PA2BA\_VIPAA | PA2BB\_GLOHA | PA2BB\_PSEAU | PA2BC\_VIPAA | PA2BD\_CRODU | PA2B\_BUNCE | PA2B\_NOTSC | PA2GA\_HUMAN | PA2GE\_HUMAN | PA2GX\_HUMAN | PA2H1\_AGKCL | PA2H1\_BOTBZ | PA2H1\_BOTJR | PA2H1\_BOTMO | PA2H1\_BOTPI | PA2H2\_BOTAS | PA2H2\_BOTMO | PA2H2\_BOTPI | PA2H2\_CERGO | PA2H3\_BOTPI | PA2HB\_AGKPI | PA2HB\_OXYSC | PA2HH\_TRIST | PA2HS\_ECHCA | PA2H\_BOTPA | PA2H\_DEIAC | PA2H\_PROMB | PA2N\_GLOHA | PA2\_APIME | PAG15\_HUMAN | PLA22\_ORYSJ | PPAP\_RAT | PPT1\_HUMAN | PPT2\_HUMAN | V5NTD\_NAJAT | GO:0051716 | 0.0934202358783364 | 301/3222 | 29/552 | 0.99995963411677 | 1 | F | F | F | F | cellular response to stimulus | ANAG\_HUMAN | ANG2\_MOUSE | ANG4\_MOUSE | AOC1\_HUMAN | CATH\_HUMAN | DABA\_PSEMU | ENPP2\_RAT | G3I1H5\_CRIGR | GBA1\_HUMAN | GPX5\_HUMAN | HYAL1\_HUMAN | LGMN\_MOUSE | LIG2\_PHACH | LIG4\_PHACH | LIG8\_PHACH | MAN12\_PENCI | MMP1\_PIG | PA21B\_PIG | PA2GX\_HUMAN | PEM1\_PHACH | PER\_ARTRA | PER\_COPCI | PGH2\_HUMAN | PPA5\_RAT | Q60FD2\_9APHY | Q96X16\_PICPA | RENI\_RAT | VPL1\_PLEER | VPL2\_PLEER | GO:0007165 | 0.0487274984481688 | 157/3222 | 10/552 | 0.999987124578867 | 1 | F | F | F | F | signal transduction | ANAG\_HUMAN | ANG2\_MOUSE | CATH\_HUMAN | CFAD\_MOUSE | GRAA\_HUMAN | GRAC\_MOUSE | LICH\_HUMAN | OFUT1\_CAEEL | PA21B\_PIG | RENI\_RAT | GO:0019725 | 0.0304158907510863 | 98/3222 | 4/552 | 0.99998726702809 | 1 | F | F | F | F | cellular homeostasis | ANG2\_MOUSE | CERU\_RAT | PPT1\_BOVIN | PPT1\_HUMAN | GO:0006576 | 0.0232774674115456 | 75/3222 | 2/552 | 0.999989300422321 | 1 | F | F | F | F | biogenic amine metabolic process | AOC1\_HUMAN | DOPO\_HUMAN | GO:0006575 | 0.0341402855369336 | 110/3222 | 5/552 | 0.99998953478157 | 1 | F | F | F | F | cellular modified amino acid metabolic process | GGH\_HUMAN | PA2GX\_HUMAN | PAG15\_HUMAN | Q6NY42\_DANRE | TTHY\_RAT | GO:0042221 | 0.134078212290503 | 432/3222 | 45/552 | 0.999989895071374 | 1 | F | F | F | F | response to chemical | ANAG\_HUMAN | ANG2\_MOUSE | ANG3\_MOUSE | ANG4\_MOUSE | ANGI\_MOUSE | AOC1\_HUMAN | AOCX\_BOVIN | CATH\_HUMAN | CBPN\_HUMAN | CEL2A\_PIG | CERU\_RAT | CHIC\_ARATH | DABA\_PSEMU | DOPO\_HUMAN | ENPP2\_RAT | G3I1H5\_CRIGR | GBA1\_HUMAN | GPX3\_HUMAN | GPX5\_HUMAN | HYAL1\_HUMAN | LALBA\_BOVIN | LGMN\_MOUSE | LICH\_HUMAN | LIG2\_PHACH | LIG4\_PHACH | LIG8\_PHACH | LIPR2\_RAT | MAN12\_PENCI | PA21B\_PIG | PA2GX\_HUMAN | PEM1\_PHACH | PER\_ARTRA | PER\_COPCI | PGH2\_HUMAN | PGPSA\_DROME | PPA5\_HUMAN | PPA5\_RAT | PTGDS\_HUMAN | PTGDS\_MOUSE | Q5WRG2\_RAT | Q60FD2\_9APHY | Q96X16\_PICPA | RENI\_RAT | VPL1\_PLEER | VPL2\_PLEER | GO:0019219 | 0.0344506517690875 | 111/3222 | 5/552 | 0.99999107151165 | 1 | F | F | F | F | regulation of nucleobase-containing compound metabolic process | ANGI\_MOUSE | GRAA\_HUMAN | PA21B\_PIG | PA2GX\_HUMAN | Q5WRG2\_RAT | GO:0030258 | 0.0238981998758535 | 77/3222 | 2/552 | 0.999992537276919 | 1 | F | F | F | F | lipid modification | GBA1\_HUMAN | MNLOX\_MAGO7 | GO:0034308 | 0.0245189323401614 | 79/3222 | 2/552 | 0.99999479942452 | 1 | F | F | F | F | primary alcohol metabolic process | PPAP\_RAT | TTHY\_CHICK | GO:0009141 | 0.0285536933581626 | 92/3222 | 3/552 | 0.999995002500445 | 1 | F | F | F | F | nucleoside triphosphate metabolic process | ASM3A\_HUMAN | ASM3A\_MOUSE | LICH\_HUMAN | GO:0044550 | 0.0248292985723153 | 80/3222 | 2/552 | 0.999995659988882 | 1 | F | F | F | F | secondary metabolite biosynthetic process | DIR\_GLYEC | EST6\_DROME | GO:0046165 | 0.0288640595903166 | 93/3222 | 3/552 | 0.999995794962508 | 1 | F | F | F | F | alcohol biosynthetic process | DOPO\_HUMAN | GBA1\_HUMAN | LICH\_HUMAN | GO:0009116 | 0.026381129733085 | 85/3222 | 2/552 | 0.999998248663311 | 1 | F | F | F | F | nucleoside metabolic process | ADA2\_HUMAN | PPAP\_RAT | GO:0043603 | 0.0617628801986344 | 199/3222 | 12/552 | 0.999999541137038 | 1 | F | F | F | F | amide metabolic process | AGAL\_HUMAN | ANAG\_HUMAN | CARP1\_CANAL | CATH\_HUMAN | CBPN\_HUMAN | GBA1\_HUMAN | PAG15\_HUMAN | PPT1\_HUMAN | PPT2\_HUMAN | RENI\_RAT | SIA\_ASPFU | TPP1\_HUMAN | GO:0008202 | 0.0328988206083178 | 106/3222 | 3/552 | 0.999999564850506 | 1 | F | F | F | F | steroid metabolic process | GBA1\_HUMAN | LICH\_HUMAN | LIP3\_DIURU | GO:0008610 | 0.0788330229671012 | 254/3222 | 18/552 | 0.999999651997584 | 1 | F | F | F | F | lipid biosynthetic process | ANG1\_BOVIN | ANG2\_MOUSE | ANG3\_MOUSE | ANG4\_MOUSE | ANGI\_MOUSE | GBA1\_HUMAN | LICH\_HUMAN | PA21B\_BOVIN | PA21B\_PIG | PA2GX\_HUMAN | PAG15\_HUMAN | PGH2\_HUMAN | PPT1\_HUMAN | PPT2\_HUMAN | PTGDS\_HUMAN | PTGDS\_MOUSE | Q5WRG2\_RAT | THCAS\_CANSA | GO:0016051 | 0.0378646803227809 | 122/3222 | 4/552 | 0.999999772645828 | 1 | F | F | F | F | carbohydrate biosynthetic process | LALBA\_BOVIN | LALBA\_CAPHI | LALBA\_CAVPO | LALBA\_PAPCY | GO:0072329 | 0.0301055245189323 | 97/3222 | 2/552 | 0.999999805359919 | 1 | F | F | F | F | monocarboxylic acid catabolic process | IDUA\_HUMAN | PAG15\_HUMAN | GO:0006631 | 0.0580384854127871 | 187/3222 | 10/552 | 0.999999830348612 | 1 | F | F | F | F | fatty acid metabolic process | AOAH\_MOUSE | ASAH1\_BALAS | MNLOX\_MAGO7 | PA21B\_BOVIN | PA21B\_PIG | PA2GX\_HUMAN | PAG15\_HUMAN | PGH2\_HUMAN | PTGDS\_HUMAN | PTGDS\_MOUSE | GO:0044272 | 0.0350713842333954 | 113/3222 | 3/552 | 0.999999873838397 | 1 | F | F | F | F | sulfur compound biosynthetic process | HS3S1\_MOUSE | PPT1\_HUMAN | PPT2\_HUMAN | GO:0006721 | 0.031967721911856 | 103/3222 | 2/552 | 0.999999935703557 | 1 | F | F | F | F | terpenoid metabolic process | THCAS\_CANSA | TTHY\_CHICK | GO:1901135 | 0.169770328988206 | 547/3222 | 54/552 | 0.999999938198237 | 1 | F | F | F | F | carbohydrate derivative metabolic process | A0A0R4I979\_BRABE | A0A3B6UEQ2\_RHIMI | A0A7S6G7I6\_9PEZI | A5AB48\_ASPNC | A6PZ97\_SALSA | A9LI60\_BIOOC | A9ZSX9\_9BRYO | ADA2\_HUMAN | AGAL\_HUMAN | ANAG\_HUMAN | AOAH\_MOUSE | B9TU22\_GADMO | BGLR\_HUMAN | CDA\_COLLN | CDA\_EMENI | CHI1\_COCPS | CHI2\_HORVU | CHI2\_ORYSJ | CHI33\_TRIHA | CHI42\_TRIHA | CHI4\_CRYJA | CHIA\_HUMAN | CHIC\_ARATH | CHIC\_SECCE | CHIL3\_MOUSE | CHIT\_PUNGR | CHLY\_HEVBR | FUCO\_HUMAN | G3JPF7\_CORMM | GBA1\_HUMAN | HEXC\_OSTFU | HS3S1\_MOUSE | HYAL1\_HUMAN | IDUA\_HUMAN | LICH\_HUMAN | LIPR2\_HUMAN | LIPR2\_RAT | LYG\_STRCA | MANBA\_MOUSE | NAGAB\_CHICK | NAGAB\_HUMAN | O81934\_CANEN | PGPSA\_DROME | PGRP1\_CAMDR | PPAP\_RAT | PPT1\_HUMAN | PPT2\_HUMAN | Q43576\_TOBAC | Q4AE59\_OSTFU | Q6WSR8\_PICAB | Q86RS6\_MANSE | Q8H0C9\_VIGUN | Q9FUH3\_VIGUS | SIA\_ASPFU | GO:0009152 | 0.0366232153941651 | 118/3222 | 3/552 | 0.999999948233686 | 1 | F | F | F | F | purine ribonucleotide biosynthetic process | LICH\_HUMAN | PPT1\_HUMAN | PPT2\_HUMAN | GO:0005996 | 0.0645561762880199 | 208/3222 | 11/552 | 0.999999969095461 | 1 | F | F | F | F | monosaccharide metabolic process | A0A059U759\_9PEZI | A0A2H5BN17\_TALPI | A8NI40\_COPC7 | ABFB\_ASPKW | AXHA2\_EMENI | FUCO\_HUMAN | G2QVH2\_THETT | IDUA\_HUMAN | J9UN47\_GIBZA | MANA\_CANEN | OFUT1\_CAEEL | GO:0036211 | 0.0819366852886406 | 264/3222 | 17/552 | 0.999999972321867 | 1 | F | F | F | F | protein modification process | A0A0R3QSA7\_9BILA | A0A3S5H5N2\_LEIDO | A4GX63\_TOXGO | ANAG\_HUMAN | ANG2\_MOUSE | CYP5\_CAEEL | MAN12\_PENCI | O81226\_CARPA | OFUT1\_CAEEL | PPT1\_BOVIN | PPT1\_HUMAN | Q06AK3\_TOXGO | Q5B038\_EMENI | QPCT1\_DROME | QPCT2\_DROME | QPCT\_IXOSC | QPCT\_MOUSE | GO:0006790 | 0.0623836126629423 | 201/3222 | 9/552 | 0.999999995819792 | 1 | F | F | F | F | sulfur compound metabolic process | ANAG\_HUMAN | BGLR\_HUMAN | HS3S1\_MOUSE | HYAL1\_HUMAN | IDUA\_HUMAN | PERL\_CAPHI | PPAP\_RAT | PPT1\_HUMAN | PPT2\_HUMAN | GO:0006720 | 0.0378646803227809 | 122/3222 | 2/552 | 0.999999998141578 | 1 | F | F | F | F | isoprenoid metabolic process | THCAS\_CANSA | TTHY\_CHICK | GO:0043412 | 0.0890751086281813 | 287/3222 | 17/552 | 0.999999998955137 | 1 | F | F | F | F | macromolecule modification | A0A0R3QSA7\_9BILA | A0A3S5H5N2\_LEIDO | A4GX63\_TOXGO | ANAG\_HUMAN | ANG2\_MOUSE | CYP5\_CAEEL | MAN12\_PENCI | O81226\_CARPA | OFUT1\_CAEEL | PPT1\_BOVIN | PPT1\_HUMAN | Q06AK3\_TOXGO | Q5B038\_EMENI | QPCT1\_DROME | QPCT2\_DROME | QPCT\_IXOSC | QPCT\_MOUSE | GO:1901617 | 0.0453134698944755 | 146/3222 | 3/552 | 0.999999999676864 | 1 | F | F | F | F | organic hydroxy compound biosynthetic process | DOPO\_HUMAN | GBA1\_HUMAN | LICH\_HUMAN | GO:0006796 | 0.229671011793917 | 740/3222 | 74/552 | 0.999999999742365 | 1 | F | F | F | F | phosphate-containing compound metabolic process | A0A1L8D5Z7\_BOTAT | A4GX63\_TOXGO | ANG2\_MOUSE | ASM3A\_HUMAN | ASM3A\_MOUSE | ENPP2\_HUMAN | ENPP2\_RAT | I1SB18\_VIPAE | IDH\_OSTTA | LICH\_HUMAN | LIPR2\_HUMAN | LIPR2\_RAT | PA21B\_BOVIN | PA21B\_PIG | PA2A1\_BUNCE | PA2A1\_ECHCA | PA2A1\_NAJAT | PA2A1\_OPHHA | PA2A2\_NAJNA | PA2A2\_OPHHA | PA2A2\_TROCA | PA2A4\_NAJSG | PA2A5\_TRIST | PA2A7\_GLOHA | PA2A\_BOTJR | PA2A\_CROAT | PA2A\_DEIAC | PA2A\_GLOHA | PA2A\_NAJAT | PA2B1\_AGKPI | PA2B2\_BOTJR | PA2B2\_PROFL | PA2B3\_BOTAS | PA2B3\_BUNCE | PA2B5\_BUNCE | PA2B5\_NOTSC | PA2BA\_VIPAA | PA2BB\_GLOHA | PA2BB\_PSEAU | PA2BC\_VIPAA | PA2BD\_CRODU | PA2B\_BUNCE | PA2B\_NOTSC | PA2GA\_HUMAN | PA2GE\_HUMAN | PA2GX\_HUMAN | PA2H1\_AGKCL | PA2H1\_BOTBZ | PA2H1\_BOTJR | PA2H1\_BOTMO | PA2H1\_BOTPI | PA2H2\_BOTAS | PA2H2\_BOTMO | PA2H2\_BOTPI | PA2H2\_CERGO | PA2H3\_BOTPI | PA2HB\_AGKPI | PA2HB\_OXYSC | PA2HH\_TRIST | PA2HS\_ECHCA | PA2H\_BOTPA | PA2H\_DEIAC | PA2H\_PROMB | PA2N\_GLOHA | PA2\_APIME | PAG15\_HUMAN | PLA22\_ORYSJ | PPA5\_HUMAN | PPA5\_RAT | PPAP\_RAT | PPT1\_HUMAN | PPT2\_HUMAN | Q06AK3\_TOXGO | V5NTD\_NAJAT | GO:0006164 | 0.0459342023587834 | 148/3222 | 3/552 | 0.999999999776289 | 1 | F | F | F | F | purine nucleotide biosynthetic process | LICH\_HUMAN | PPT1\_HUMAN | PPT2\_HUMAN | GO:0072522 | 0.0521415270018622 | 168/3222 | 4/552 | 0.999999999932257 | 1 | F | F | F | F | purine-containing compound biosynthetic process | ADA2\_HUMAN | LICH\_HUMAN | PPT1\_HUMAN | PPT2\_HUMAN | GO:0009260 | 0.048106765983861 | 155/3222 | 3/552 | 0.999999999938699 | 1 | F | F | F | F | ribonucleotide biosynthetic process | LICH\_HUMAN | PPT1\_HUMAN | PPT2\_HUMAN | GO:0019318 | 0.0524518932340161 | 169/3222 | 4/552 | 0.999999999944745 | 1 | F | F | F | F | hexose metabolic process | FUCO\_HUMAN | J9UN47\_GIBZA | MANA\_CANEN | OFUT1\_CAEEL | GO:0046390 | 0.0493482309124767 | 159/3222 | 3/552 | 0.99999999997225 | 1 | F | F | F | F | ribose phosphate biosynthetic process | LICH\_HUMAN | PPT1\_HUMAN | PPT2\_HUMAN | GO:0006066 | 0.0657976412166356 | 212/3222 | 7/552 | 0.999999999982286 | 1 | F | F | F | F | alcohol metabolic process | DOPO\_HUMAN | GBA1\_HUMAN | IDH\_OSTTA | LICH\_HUMAN | LIP3\_DIURU | PPAP\_RAT | TTHY\_CHICK | GO:1901564 | 0.458721291123526 | 1478/3222 | 152/552 | 0.999999999994023 | 1 | F | F | F | F | organonitrogen compound metabolic process | A0A0R3QSA7\_9BILA | A0A0R4I979\_BRABE | A0A3B6UEQ2\_RHIMI | A0A3S5H5N2\_LEIDO | A0A6P6YAT6\_DERPT | A0A7S6G7I6\_9PEZI | A0NFU8\_ANOGA | A4GX63\_TOXGO | A5AB48\_ASPNC | A6PZ97\_SALSA | A9LI60\_BIOOC | A9ZSX9\_9BRYO | ADA2\_HUMAN | AGAL\_HUMAN | ANAG\_HUMAN | ANG2\_MOUSE | AOC1\_HUMAN | AOCX\_BOVIN | ASAH1\_BALAS | B4F320\_LIMPO | B9TU22\_GADMO | BGLR\_HUMAN | CARP1\_CANAL | CARP2\_CANAX | CARP\_RHIPU | CATD\_RAT | CATH\_HUMAN | CATLL\_FASHE | CBPA1\_PIG | CBPD\_LOPSP | CBPN\_HUMAN | CDA\_COLLN | CDA\_EMENI | CEL2A\_PIG | CFAD\_MOUSE | CHI1\_COCPS | CHI2\_HORVU | CHI2\_ORYSJ | CHI33\_TRIHA | CHI42\_TRIHA | CHI4\_CRYJA | CHIA\_HUMAN | CHIC\_ARATH | CHIC\_SECCE | CHIL3\_MOUSE | CHIT\_PUNGR | CHLY\_HEVBR | CHYM\_CAMDR | CKX1\_MAIZE | COGS\_HYPLI | CUCM1\_CUCME | CYP5\_CAEEL | CYSP\_BLOTA | D6XHE1\_TRYB2 | DDN1\_BOVIN | DOPO\_HUMAN | DPP2\_HUMAN | EGFB2\_MOUSE | ENPP2\_HUMAN | ENPP2\_RAT | ERVB\_TABDI | FUCO\_HUMAN | G3I1H5\_CRIGR | G3JPF7\_CORMM | GBA1\_HUMAN | GGH\_HUMAN | GRAA\_HUMAN | GRAC\_MOUSE | GRAK\_HUMAN | GRASS\_DROME | HE12\_DANRE | HEXC\_OSTFU | HS3S1\_MOUSE | HYAL1\_HUMAN | IDH\_OSTTA | IDUA\_HUMAN | J7LCB0\_DEIAC | KLK10\_HUMAN | KLK1\_HUMAN | KLK2\_HORSE | KLK2\_HUMAN | KLK7\_HUMAN | KLK7\_MOUSE | KLK8\_MOUSE | LAPA\_ASPOR | LGMN\_MOUSE | LICH\_HUMAN | LIPR2\_HUMAN | LIPR2\_RAT | LYG\_STRCA | MAN12\_PENCI | MANBA\_MOUSE | MCPT2\_RAT | MMP1\_PIG | NCS\_THLFG | O81226\_CARPA | O81934\_CANEN | O97389\_HELAM | OFUT1\_CAEEL | PA21B\_BOVIN | PA21B\_PIG | PA2GA\_HUMAN | PA2GE\_HUMAN | PA2GX\_HUMAN | PAG15\_HUMAN | PCP\_HUMAN | PEPA\_ASPPH | PGPSA\_DROME | PGRP1\_CAMDR | PPAF1\_HOLDI | PPAP\_RAT | PPT1\_BOVIN | PPT1\_HUMAN | PPT2\_HUMAN | PRS57\_HUMAN | PRTN3\_HUMAN | Q06AK3\_TOXGO | Q43576\_TOBAC | Q4AE59\_OSTFU | Q5B038\_EMENI | Q69G21\_TENMO | Q6NY42\_DANRE | Q6R7Z5\_9TRYP | Q6WSR8\_PICAB | Q7YXL2\_TENMO | Q86RS6\_MANSE | Q8H0C9\_VIGUN | Q96X16\_PICPA | Q9FUH3\_VIGUS | QPCT1\_DROME | QPCT2\_DROME | QPCT\_IXOSC | QPCT\_MOUSE | RENI\_RAT | SIA\_ASPFU | TPP1\_HUMAN | TRFL\_BUBBU | TRFL\_HORSE | TRY1\_GADMO | TRY3\_SALSA | TRYB2\_HUMAN | TTHY\_CHICK | TTHY\_MOUSE | TTHY\_RAT | VM11\_BOTMO | VM12\_CROAD | VM1A3\_DEIAC | VM1BI\_BOTMO | VM1T1\_PROMU | VM1T2\_PROFL | VSPP\_DEIAC | VSPSX\_GLOSA | GO:1901576 | 0.352265673494724 | 1135/3222 | 31/552 | 0.999999999994515 | 1 | F | F | F | F | organic substance biosynthetic process | ADA2\_HUMAN | ANG1\_BOVIN | ANG2\_MOUSE | ANG3\_MOUSE | ANG4\_MOUSE | ANGI\_MOUSE | DIR\_GLYEC | DOPO\_HUMAN | GBA1\_HUMAN | HS3S1\_MOUSE | HYAL1\_HUMAN | LALBA\_BOVIN | LALBA\_CAPHI | LALBA\_CAVPO | LALBA\_PAPCY | LICH\_HUMAN | MDLA\_PENCA | MDLA\_PENCY | PA21B\_BOVIN | PA21B\_PIG | PA2GX\_HUMAN | PAG15\_HUMAN | PGH2\_HUMAN | PPT1\_HUMAN | PPT2\_HUMAN | PTGDS\_HUMAN | PTGDS\_MOUSE | Q5WRG2\_RAT | Q7LST4\_PENEN | THCAS\_CANSA | W6Q990\_PENRF | GO:0009987 | 0.750775915580385 | 2419/3222 | 241/552 | 0.999999999995127 | 1 | F | F | F | F | cellular process | A0A087WNH2\_FICBE | A0A0A0Y4H8\_TRAFO | A0A0R3QSA7\_9BILA | A0A1L8D5Z7\_BOTAT | A0A1S4NYF8\_PANVG | A0A3L6SKP5\_PANMI | A0A3S5H5N2\_LEIDO | A0A6M9BP13\_9EURO | A1E266\_9PEZI | A1HA\_LOXIN | A1HB2\_LOXIN | A311\_LOXLA | A4GX63\_TOXGO | A6PZ97\_SALSA | ADA2\_HUMAN | ADPG2\_ARATH | AGAL\_HUMAN | ANAG\_HUMAN | ANG1\_BOVIN | ANG2\_MOUSE | ANG3\_MOUSE | ANG4\_MOUSE | ANGI\_MOUSE | AOAH\_MOUSE | AOC1\_HUMAN | APO1\_CYCAE | ASAH1\_BALAS | ASM3A\_HUMAN | ASM3A\_MOUSE | ATLE\_CYCAE | B9TU22\_GADMO | BGLR\_HUMAN | CARP1\_CANAL | CAT3\_NEUCR | CATD\_RAT | CATH\_HUMAN | CBPA1\_PIG | CBPN\_HUMAN | CDA\_COLLN | CDA\_EMENI | CERU\_RAT | CFAD\_MOUSE | CHI4\_CRYJA | CHIA\_HUMAN | CHIC\_SECCE | CKX1\_MAIZE | CYP5\_CAEEL | D1MPT2\_ROYRE | DABA\_PSEMU | DIR\_GLYEC | DNAS1\_HUMAN | DNSL3\_HUMAN | DOPO\_HUMAN | ECP\_HUMAN | ENDO2\_ARATH | ENG1\_RHIMI | ENPP2\_HUMAN | ENPP2\_RAT | EST6\_DROME | EXG1\_CANAL | EXG1\_YEAST | FUCO\_HUMAN | G3I1H5\_CRIGR | G3YAL0\_ASPNA | GANA\_EMENI | GBA1\_HUMAN | GCE2\_MYCTT | GCE\_CERUI | GCE\_HYPJQ | GGH\_HUMAN | GPX3\_HUMAN | GPX5\_HUMAN | GRAA\_HUMAN | GRAC\_MOUSE | HS3S1\_MOUSE | HYAL1\_HUMAN | I1SB18\_VIPAE | IDH\_OSTTA | IDUA\_HUMAN | K7N5L9\_RAPSA | KATG2\_MAGO7 | KLK10\_HUMAN | KLK7\_HUMAN | KLK8\_MOUSE | LAC1\_MELAO | LAC1\_TRAMX | LAC2\_TRAVE | LGMN\_MOUSE | LICH\_HUMAN | LIG2\_PHACH | LIG4\_PHACH | LIG8\_PHACH | LIPG\_HUMAN | LIPR2\_HUMAN | LIPR2\_RAT | LUCI\_OPLGR | LYG\_STRCA | LYSC1\_ANAPL | LYSC1\_CANLF | LYSC1\_HORSE | LYSC2\_BOVIN | LYSC2\_ONCMY | LYSC\_COTJA | LYSC\_EQUAS | LYSC\_NUMME | LYSC\_OPIHO | LYSC\_PELSI | LYS\_BOMMO | LYS\_RUDPH | MAN12\_PENCI | MDLA\_PENCA | MDLA\_PENCY | MMP1\_PIG | MNLOX\_MAGO7 | NAGAB\_HUMAN | NUP1\_PENCI | NUS1\_ASPOR | O22443\_SOYBN | OFUT1\_CAEEL | OXLA\_BOTAT | OXLA\_CALRH | OXLA\_GLOHA | P79074\_9AGAR | PA1\_VESBA | PA21B\_BOVIN | PA21B\_PIG | PA2A1\_BUNCE | PA2A1\_ECHCA | PA2A1\_NAJAT | PA2A1\_OPHHA | PA2A2\_NAJNA | PA2A2\_OPHHA | PA2A2\_TROCA | PA2A4\_NAJSG | PA2A5\_TRIST | PA2A7\_GLOHA | PA2A\_BOTJR | PA2A\_CROAT | PA2A\_DEIAC | PA2A\_GLOHA | PA2A\_NAJAT | PA2B1\_AGKPI | PA2B2\_BOTJR | PA2B2\_PROFL | PA2B3\_BOTAS | PA2B3\_BUNCE | PA2B5\_BUNCE | PA2B5\_NOTSC | PA2BA\_VIPAA | PA2BB\_GLOHA | PA2BB\_PSEAU | PA2BC\_VIPAA | PA2BD\_CRODU | PA2B\_BUNCE | PA2B\_NOTSC | PA2GA\_HUMAN | PA2GE\_HUMAN | PA2GX\_HUMAN | PA2H1\_AGKCL | PA2H1\_BOTBZ | PA2H1\_BOTJR | PA2H1\_BOTMO | PA2H1\_BOTPI | PA2H2\_BOTAS | PA2H2\_BOTMO | PA2H2\_BOTPI | PA2H2\_CERGO | PA2H3\_BOTPI | PA2HB\_AGKPI | PA2HB\_OXYSC | PA2HH\_TRIST | PA2HS\_ECHCA | PA2H\_BOTPA | PA2H\_DEIAC | PA2H\_PROMB | PA2N\_GLOHA | PA2\_APIME | PAG15\_HUMAN | PELA\_ASPNG | PELB\_ASPNG | PEM1\_PHACH | PER1A\_ARMRU | PER1\_ARAHY | PER1\_SORBI | PER53\_ARATH | PER59\_ARATH | PERL\_BOVIN | PERL\_BUBBU | PERL\_CAPHI | PER\_ARTRA | PER\_COPCI | PGH2\_HUMAN | PGLR1\_ASPAC | PGLR1\_ASPNG | PGLR\_GIBFU | PLA22\_ORYSJ | PME\_DAUCA | PME\_SITOR | POXA\_DICDI | PPA5\_HUMAN | PPA5\_PIG | PPA5\_RAT | PPAP\_RAT | PPT1\_BOVIN | PPT1\_HUMAN | PPT2\_HUMAN | PRTN3\_HUMAN | PTGDS\_HUMAN | PTGDS\_MOUSE | Q06AK3\_TOXGO | Q07524\_TROMA | Q0KFV0\_SOLLC | Q40069\_HORVU | Q5WRG2\_RAT | Q60FD2\_9APHY | Q6NY42\_DANRE | Q7LST4\_PENEN | Q96X16\_PICPA | Q9LYJ5\_ARATH | RENI\_RAT | RGLA\_ASPAC | RHGA\_ASPAC | RIP1\_HORVU | RNAS6\_HUMAN | RNLE\_SOLLC | RNSL3\_DANRE | RNT2\_HUMAN | SIA\_ASPFU | THCAS\_CANSA | TPP1\_HUMAN | TRFL\_BUBBU | TRFL\_HORSE | TTHY\_CHICK | TTHY\_MOUSE | TTHY\_RAT | V5NTD\_NAJAT | VPL1\_PLEER | VPL2\_PLEER | W6Q990\_PENRF | XGHA\_ASPTU | XTH34\_POPPZ | GO:0044281 | 0.465238981998759 | 1499/3222 | 44/552 | 0.999999999995806 | 1 | F | F | F | F | small molecule metabolic process | A0A059U759\_9PEZI | A0A2H5BN17\_TALPI | A8NI40\_COPC7 | ABFB\_ASPKW | ADA2\_HUMAN | AOAH\_MOUSE | ASAH1\_BALAS | ASM3A\_HUMAN | ASM3A\_MOUSE | AXHA2\_EMENI | CBPA1\_PIG | DOPO\_HUMAN | FUCO\_HUMAN | G2QVH2\_THETT | GBA1\_HUMAN | IDH\_OSTTA | IDUA\_HUMAN | J9UN47\_GIBZA | LICH\_HUMAN | LIP3\_DIURU | LIPG\_HUMAN | MANA\_CANEN | MDLA\_PENCA | MDLA\_PENCY | MNLOX\_MAGO7 | OFUT1\_CAEEL | PA21B\_BOVIN | PA21B\_PIG | PA2GX\_HUMAN | PAG15\_HUMAN | PERL\_CAPHI | PGH2\_HUMAN | PPAP\_RAT | PPT1\_HUMAN | PPT2\_HUMAN | PTGDS\_HUMAN | PTGDS\_MOUSE | Q6NY42\_DANRE | Q7LST4\_PENEN | TTHY\_CHICK | TTHY\_MOUSE | TTHY\_RAT | V5NTD\_NAJAT | W6Q990\_PENRF | GO:1901360 | 0.311607697082557 | 1004/3222 | 47/552 | 0.999999999996707 | 1 | F | F | F | F | organic cyclic compound metabolic process | ADA2\_HUMAN | ANG1\_BOVIN | ANG2\_MOUSE | ANG3\_MOUSE | ANG4\_MOUSE | ANGI\_MOUSE | ASM3A\_HUMAN | ASM3A\_MOUSE | DIR\_GLYEC | DNAS1\_HUMAN | DNSL3\_HUMAN | DOPO\_HUMAN | ECP\_HUMAN | ENDO2\_ARATH | GBA1\_HUMAN | GCE2\_MYCTT | GCE\_CERUI | GCE\_HYPJQ | GGH\_HUMAN | IDH\_OSTTA | LAC1\_MELAO | LAC1\_TRAMX | LAC2\_TRAVE | LICH\_HUMAN | LIG2\_PHACH | LIG4\_PHACH | LIG8\_PHACH | LIP3\_DIURU | NUP1\_PENCI | NUS1\_ASPOR | PEM1\_PHACH | PPAP\_RAT | PPT1\_HUMAN | PPT2\_HUMAN | Q0KFV0\_SOLLC | Q5WRG2\_RAT | Q60FD2\_9APHY | Q6NY42\_DANRE | RNAS6\_HUMAN | RNLE\_SOLLC | RNT2\_HUMAN | TTHY\_CHICK | TTHY\_MOUSE | TTHY\_RAT | V5NTD\_NAJAT | VPL1\_PLEER | VPL2\_PLEER | GO:0009058 | 0.372749844816884 | 1201/3222 | 34/552 | 0.999999999997352 | 1 | F | F | F | F | biosynthetic process | ADA2\_HUMAN | ANG1\_BOVIN | ANG2\_MOUSE | ANG3\_MOUSE | ANG4\_MOUSE | ANGI\_MOUSE | DIR\_GLYEC | DOPO\_HUMAN | EST6\_DROME | GBA1\_HUMAN | HS3S1\_MOUSE | HYAL1\_HUMAN | LALBA\_BOVIN | LALBA\_CAPHI | LALBA\_CAVPO | LALBA\_PAPCY | LICH\_HUMAN | MDLA\_PENCA | MDLA\_PENCY | PA21B\_BOVIN | PA21B\_PIG | PA2GX\_HUMAN | PAG15\_HUMAN | PGH2\_HUMAN | PPA5\_HUMAN | PPA5\_RAT | PPT1\_HUMAN | PPT2\_HUMAN | PTGDS\_HUMAN | PTGDS\_MOUSE | Q5WRG2\_RAT | Q7LST4\_PENEN | THCAS\_CANSA | W6Q990\_PENRF | GO:0009150 | 0.0543140906269398 | 175/3222 | 3/552 | 0.999999999997656 | 1 | F | F | F | F | purine ribonucleotide metabolic process | LICH\_HUMAN | PPT1\_HUMAN | PPT2\_HUMAN | GO:0006139 | 0.188081936685289 | 606/3222 | 28/552 | 0.999999999997669 | 1 | F | F | F | F | nucleobase-containing compound metabolic process | ADA2\_HUMAN | ANG1\_BOVIN | ANG2\_MOUSE | ANG3\_MOUSE | ANG4\_MOUSE | ANGI\_MOUSE | ASM3A\_HUMAN | ASM3A\_MOUSE | DNAS1\_HUMAN | DNSL3\_HUMAN | ECP\_HUMAN | ENDO2\_ARATH | IDH\_OSTTA | LICH\_HUMAN | NUP1\_PENCI | NUS1\_ASPOR | PPAP\_RAT | PPT1\_HUMAN | PPT2\_HUMAN | Q0KFV0\_SOLLC | Q5WRG2\_RAT | RNAS6\_HUMAN | RNLE\_SOLLC | RNT2\_HUMAN | TTHY\_CHICK | TTHY\_MOUSE | TTHY\_RAT | V5NTD\_NAJAT | GO:0006163 | 0.0822470515207945 | 265/3222 | 4/552 | 0.999999999998385 | 1 | F | F | F | F | purine nucleotide metabolic process | IDH\_OSTTA | LICH\_HUMAN | PPT1\_HUMAN | PPT2\_HUMAN | GO:0044237 | 0.662942271880819 | 2136/3222 | 164/552 | 0.999999999999075 | 1 | F | F | F | F | cellular metabolic process | A0A087WNH2\_FICBE | A0A0A0Y4H8\_TRAFO | A0A1L8D5Z7\_BOTAT | A0A1S4NYF8\_PANVG | A0A3L6SKP5\_PANMI | A4GX63\_TOXGO | ADA2\_HUMAN | AGAL\_HUMAN | ANAG\_HUMAN | ANG1\_BOVIN | ANG2\_MOUSE | ANG3\_MOUSE | ANG4\_MOUSE | ANGI\_MOUSE | AOAH\_MOUSE | APO1\_CYCAE | ASAH1\_BALAS | ASM3A\_HUMAN | ASM3A\_MOUSE | BGLR\_HUMAN | CAT3\_NEUCR | CATH\_HUMAN | CBPA1\_PIG | CBPN\_HUMAN | CKX1\_MAIZE | D1MPT2\_ROYRE | DIR\_GLYEC | DNAS1\_HUMAN | DNSL3\_HUMAN | DOPO\_HUMAN | ECP\_HUMAN | ENDO2\_ARATH | ENPP2\_HUMAN | ENPP2\_RAT | EST6\_DROME | FUCO\_HUMAN | G3I1H5\_CRIGR | GBA1\_HUMAN | GCE2\_MYCTT | GCE\_CERUI | GCE\_HYPJQ | GGH\_HUMAN | GPX3\_HUMAN | HS3S1\_MOUSE | HYAL1\_HUMAN | I1SB18\_VIPAE | IDH\_OSTTA | IDUA\_HUMAN | K7N5L9\_RAPSA | KATG2\_MAGO7 | LAC1\_MELAO | LAC1\_TRAMX | LAC2\_TRAVE | LGMN\_MOUSE | LICH\_HUMAN | LIG2\_PHACH | LIG4\_PHACH | LIG8\_PHACH | LIPG\_HUMAN | LIPR2\_HUMAN | LIPR2\_RAT | LUCI\_OPLGR | MDLA\_PENCA | MDLA\_PENCY | MNLOX\_MAGO7 | NAGAB\_HUMAN | NUP1\_PENCI | NUS1\_ASPOR | O22443\_SOYBN | PA21B\_BOVIN | PA21B\_PIG | PA2A1\_BUNCE | PA2A1\_ECHCA | PA2A1\_NAJAT | PA2A1\_OPHHA | PA2A2\_NAJNA | PA2A2\_OPHHA | PA2A2\_TROCA | PA2A4\_NAJSG | PA2A5\_TRIST | PA2A7\_GLOHA | PA2A\_BOTJR | PA2A\_CROAT | PA2A\_DEIAC | PA2A\_GLOHA | PA2A\_NAJAT | PA2B1\_AGKPI | PA2B2\_BOTJR | PA2B2\_PROFL | PA2B3\_BOTAS | PA2B3\_BUNCE | PA2B5\_BUNCE | PA2B5\_NOTSC | PA2BA\_VIPAA | PA2BB\_GLOHA | PA2BB\_PSEAU | PA2BC\_VIPAA | PA2BD\_CRODU | PA2B\_BUNCE | PA2B\_NOTSC | PA2GA\_HUMAN | PA2GE\_HUMAN | PA2GX\_HUMAN | PA2H1\_AGKCL | PA2H1\_BOTBZ | PA2H1\_BOTJR | PA2H1\_BOTMO | PA2H1\_BOTPI | PA2H2\_BOTAS | PA2H2\_BOTMO | PA2H2\_BOTPI | PA2H2\_CERGO | PA2H3\_BOTPI | PA2HB\_AGKPI | PA2HB\_OXYSC | PA2HH\_TRIST | PA2HS\_ECHCA | PA2H\_BOTPA | PA2H\_DEIAC | PA2H\_PROMB | PA2N\_GLOHA | PA2\_APIME | PAG15\_HUMAN | PEM1\_PHACH | PER1A\_ARMRU | PER1\_ARAHY | PER1\_SORBI | PER53\_ARATH | PER59\_ARATH | PERL\_BOVIN | PERL\_BUBBU | PERL\_CAPHI | PER\_ARTRA | PER\_COPCI | PGH2\_HUMAN | PLA22\_ORYSJ | POXA\_DICDI | PPA5\_HUMAN | PPA5\_RAT | PPAP\_RAT | PPT1\_HUMAN | PPT2\_HUMAN | PTGDS\_HUMAN | PTGDS\_MOUSE | Q06AK3\_TOXGO | Q0KFV0\_SOLLC | Q40069\_HORVU | Q5WRG2\_RAT | Q60FD2\_9APHY | Q6NY42\_DANRE | Q7LST4\_PENEN | RNAS6\_HUMAN | RNLE\_SOLLC | RNT2\_HUMAN | SIA\_ASPFU | THCAS\_CANSA | TPP1\_HUMAN | TTHY\_CHICK | TTHY\_MOUSE | TTHY\_RAT | V5NTD\_NAJAT | VPL1\_PLEER | VPL2\_PLEER | W6Q990\_PENRF | GO:0006793 | 0.240533829919305 | 775/3222 | 74/552 | 0.999999999999246 | 1 | F | F | F | F | phosphorus metabolic process | A0A1L8D5Z7\_BOTAT | A4GX63\_TOXGO | ANG2\_MOUSE | ASM3A\_HUMAN | ASM3A\_MOUSE | ENPP2\_HUMAN | ENPP2\_RAT | I1SB18\_VIPAE | IDH\_OSTTA | LICH\_HUMAN | LIPR2\_HUMAN | LIPR2\_RAT | PA21B\_BOVIN | PA21B\_PIG | PA2A1\_BUNCE | PA2A1\_ECHCA | PA2A1\_NAJAT | PA2A1\_OPHHA | PA2A2\_NAJNA | PA2A2\_OPHHA | PA2A2\_TROCA | PA2A4\_NAJSG | PA2A5\_TRIST | PA2A7\_GLOHA | PA2A\_BOTJR | PA2A\_CROAT | PA2A\_DEIAC | PA2A\_GLOHA | PA2A\_NAJAT | PA2B1\_AGKPI | PA2B2\_BOTJR | PA2B2\_PROFL | PA2B3\_BOTAS | PA2B3\_BUNCE | PA2B5\_BUNCE | PA2B5\_NOTSC | PA2BA\_VIPAA | PA2BB\_GLOHA | PA2BB\_PSEAU | PA2BC\_VIPAA | PA2BD\_CRODU | PA2B\_BUNCE | PA2B\_NOTSC | PA2GA\_HUMAN | PA2GE\_HUMAN | PA2GX\_HUMAN | PA2H1\_AGKCL | PA2H1\_BOTBZ | PA2H1\_BOTJR | PA2H1\_BOTMO | PA2H1\_BOTPI | PA2H2\_BOTAS | PA2H2\_BOTMO | PA2H2\_BOTPI | PA2H2\_CERGO | PA2H3\_BOTPI | PA2HB\_AGKPI | PA2HB\_OXYSC | PA2HH\_TRIST | PA2HS\_ECHCA | PA2H\_BOTPA | PA2H\_DEIAC | PA2H\_PROMB | PA2N\_GLOHA | PA2\_APIME | PAG15\_HUMAN | PLA22\_ORYSJ | PPA5\_HUMAN | PPA5\_RAT | PPAP\_RAT | PPT1\_HUMAN | PPT2\_HUMAN | Q06AK3\_TOXGO | V5NTD\_NAJAT | GO:0034654 | 0.090316573556797 | 291/3222 | 10/552 | 0.999999999999418 | 1 | F | F | F | F | nucleobase-containing compound biosynthetic process | ADA2\_HUMAN | ANG1\_BOVIN | ANG2\_MOUSE | ANG3\_MOUSE | ANG4\_MOUSE | ANGI\_MOUSE | LICH\_HUMAN | PPT1\_HUMAN | PPT2\_HUMAN | Q5WRG2\_RAT | GO:0046394 | 0.102731222842955 | 331/3222 | 10/552 | 0.999999999999431 | 1 | F | F | F | F | carboxylic acid biosynthetic process | MDLA\_PENCA | MDLA\_PENCY | PA21B\_BOVIN | PA21B\_PIG | PA2GX\_HUMAN | PGH2\_HUMAN | PTGDS\_HUMAN | PTGDS\_MOUSE | Q7LST4\_PENEN | W6Q990\_PENRF | GO:0044271 | 0.148355058969584 | 478/3222 | 14/552 | 0.999999999999482 | 1 | F | F | F | F | cellular nitrogen compound biosynthetic process | ADA2\_HUMAN | ANG1\_BOVIN | ANG2\_MOUSE | ANG3\_MOUSE | ANG4\_MOUSE | ANGI\_MOUSE | DOPO\_HUMAN | GBA1\_HUMAN | LICH\_HUMAN | PPA5\_HUMAN | PPA5\_RAT | PPT1\_HUMAN | PPT2\_HUMAN | Q5WRG2\_RAT | GO:0009117 | 0.111731843575419 | 360/3222 | 6/552 | 0.999999999999503 | 1 | F | F | F | F | nucleotide metabolic process | IDH\_OSTTA | LICH\_HUMAN | PPAP\_RAT | PPT1\_HUMAN | PPT2\_HUMAN | V5NTD\_NAJAT | GO:0006091 | 0.0757293606455618 | 244/3222 | 2/552 | 0.999999999999869 | 1 | F | F | F | F | generation of precursor metabolites and energy | GBA1\_HUMAN | LICH\_HUMAN | GO:0032787 | 0.15114835505897 | 487/3222 | 16/552 | 1 | 1 | F | F | F | F | monocarboxylic acid metabolic process | AOAH\_MOUSE | ASAH1\_BALAS | IDUA\_HUMAN | LICH\_HUMAN | MDLA\_PENCA | MDLA\_PENCY | MNLOX\_MAGO7 | PA21B\_BOVIN | PA21B\_PIG | PA2GX\_HUMAN | PAG15\_HUMAN | PGH2\_HUMAN | PTGDS\_HUMAN | PTGDS\_MOUSE | Q7LST4\_PENEN | W6Q990\_PENRF | GO:0019438 | 0.139975170701428 | 451/3222 | 12/552 | 1 | 1 | F | F | F | F | aromatic compound biosynthetic process | ADA2\_HUMAN | ANG1\_BOVIN | ANG2\_MOUSE | ANG3\_MOUSE | ANG4\_MOUSE | ANGI\_MOUSE | DIR\_GLYEC | DOPO\_HUMAN | LICH\_HUMAN | PPT1\_HUMAN | PPT2\_HUMAN | Q5WRG2\_RAT | GO:0006725 | 0.276225946617008 | 890/3222 | 46/552 | 1 | 1 | F | F | F | F | cellular aromatic compound metabolic process | ADA2\_HUMAN | ANG1\_BOVIN | ANG2\_MOUSE | ANG3\_MOUSE | ANG4\_MOUSE | ANGI\_MOUSE | ASM3A\_HUMAN | ASM3A\_MOUSE | CKX1\_MAIZE | DIR\_GLYEC | DNAS1\_HUMAN | DNSL3\_HUMAN | DOPO\_HUMAN | ECP\_HUMAN | ENDO2\_ARATH | GCE2\_MYCTT | GCE\_CERUI | GCE\_HYPJQ | GGH\_HUMAN | IDH\_OSTTA | LAC1\_MELAO | LAC1\_TRAMX | LAC2\_TRAVE | LICH\_HUMAN | LIG2\_PHACH | LIG4\_PHACH | LIG8\_PHACH | NUP1\_PENCI | NUS1\_ASPOR | PEM1\_PHACH | PPAP\_RAT | PPT1\_HUMAN | PPT2\_HUMAN | Q0KFV0\_SOLLC | Q5WRG2\_RAT | Q60FD2\_9APHY | Q6NY42\_DANRE | RNAS6\_HUMAN | RNLE\_SOLLC | RNT2\_HUMAN | TTHY\_CHICK | TTHY\_MOUSE | TTHY\_RAT | V5NTD\_NAJAT | VPL1\_PLEER | VPL2\_PLEER | GO:0044249 | 0.300744878957169 | 969/3222 | 30/552 | 1 | 1 | F | F | F | F | cellular biosynthetic process | ADA2\_HUMAN | ANG1\_BOVIN | ANG2\_MOUSE | ANG3\_MOUSE | ANG4\_MOUSE | ANGI\_MOUSE | DIR\_GLYEC | DOPO\_HUMAN | EST6\_DROME | GBA1\_HUMAN | HS3S1\_MOUSE | HYAL1\_HUMAN | LICH\_HUMAN | MDLA\_PENCA | MDLA\_PENCY | PA21B\_BOVIN | PA21B\_PIG | PA2GX\_HUMAN | PAG15\_HUMAN | PGH2\_HUMAN | PPA5\_HUMAN | PPA5\_RAT | PPT1\_HUMAN | PPT2\_HUMAN | PTGDS\_HUMAN | PTGDS\_MOUSE | Q5WRG2\_RAT | Q7LST4\_PENEN | THCAS\_CANSA | W6Q990\_PENRF | GO:0046395 | 0.0732464307883302 | 236/3222 | 2/552 | 1 | 1 | F | F | F | F | carboxylic acid catabolic process | IDUA\_HUMAN | PAG15\_HUMAN | GO:1901362 | 0.171011793916822 | 551/3222 | 12/552 | 1 | 1 | F | F | F | F | organic cyclic compound biosynthetic process | ADA2\_HUMAN | ANG1\_BOVIN | ANG2\_MOUSE | ANG3\_MOUSE | ANG4\_MOUSE | ANGI\_MOUSE | DIR\_GLYEC | DOPO\_HUMAN | LICH\_HUMAN | PPT1\_HUMAN | PPT2\_HUMAN | Q5WRG2\_RAT | GO:0009259 | 0.0667287399130975 | 215/3222 | 3/552 | 1 | 1 | F | F | F | F | ribonucleotide metabolic process | LICH\_HUMAN | PPT1\_HUMAN | PPT2\_HUMAN | GO:0090407 | 0.102420856610801 | 330/3222 | 3/552 | 1 | 1 | F | F | F | F | organophosphate biosynthetic process | LICH\_HUMAN | PPT1\_HUMAN | PPT2\_HUMAN | GO:0009165 | 0.0679702048417132 | 219/3222 | 3/552 | 1 | 1 | F | F | F | F | nucleotide biosynthetic process | LICH\_HUMAN | PPT1\_HUMAN | PPT2\_HUMAN | GO:1901137 | 0.0859714463066418 | 277/3222 | 6/552 | 1 | 1 | F | F | F | F | carbohydrate derivative biosynthetic process | ADA2\_HUMAN | HS3S1\_MOUSE | HYAL1\_HUMAN | LICH\_HUMAN | PPT1\_HUMAN | PPT2\_HUMAN | GO:0006082 | 0.291433891992551 | 939/3222 | 21/552 | 1 | 1 | F | F | F | F | organic acid metabolic process | AOAH\_MOUSE | ASAH1\_BALAS | CBPA1\_PIG | IDH\_OSTTA | IDUA\_HUMAN | LICH\_HUMAN | LIPG\_HUMAN | MDLA\_PENCA | MDLA\_PENCY | MNLOX\_MAGO7 | PA21B\_BOVIN | PA21B\_PIG | PA2GX\_HUMAN | PAG15\_HUMAN | PERL\_CAPHI | PGH2\_HUMAN | PTGDS\_HUMAN | PTGDS\_MOUSE | Q6NY42\_DANRE | Q7LST4\_PENEN | W6Q990\_PENRF | GO:0055086 | 0.144009931719429 | 464/3222 | 12/552 | 1 | 1 | F | F | F | F | nucleobase-containing small molecule metabolic process | ADA2\_HUMAN | ASM3A\_HUMAN | ASM3A\_MOUSE | IDH\_OSTTA | LICH\_HUMAN | PPAP\_RAT | PPT1\_HUMAN | PPT2\_HUMAN | TTHY\_CHICK | TTHY\_MOUSE | TTHY\_RAT | V5NTD\_NAJAT | GO:0018130 | 0.134388578522657 | 433/3222 | 10/552 | 1 | 1 | F | F | F | F | heterocycle biosynthetic process | ADA2\_HUMAN | ANG1\_BOVIN | ANG2\_MOUSE | ANG3\_MOUSE | ANG4\_MOUSE | ANGI\_MOUSE | LICH\_HUMAN | PPT1\_HUMAN | PPT2\_HUMAN | Q5WRG2\_RAT | GO:0016310 | 0.0679702048417132 | 219/3222 | 2/552 | 1 | 1 | F | F | F | F | phosphorylation | ANG2\_MOUSE | Q06AK3\_TOXGO | GO:0072521 | 0.0965238981998758 | 311/3222 | 9/552 | 1 | 1 | F | F | F | F | purine-containing compound metabolic process | ADA2\_HUMAN | IDH\_OSTTA | LICH\_HUMAN | PPAP\_RAT | PPT1\_HUMAN | PPT2\_HUMAN | TTHY\_CHICK | TTHY\_MOUSE | TTHY\_RAT | GO:0044283 | 0.175667287399131 | 566/3222 | 14/552 | 1 | 1 | F | F | F | F | small molecule biosynthetic process | ADA2\_HUMAN | DOPO\_HUMAN | GBA1\_HUMAN | LICH\_HUMAN | MDLA\_PENCA | MDLA\_PENCY | PA21B\_BOVIN | PA21B\_PIG | PA2GX\_HUMAN | PGH2\_HUMAN | PTGDS\_HUMAN | PTGDS\_MOUSE | Q7LST4\_PENEN | W6Q990\_PENRF | GO:0043436 | 0.290192427063935 | 935/3222 | 20/552 | 1 | 1 | F | F | F | F | oxoacid metabolic process | AOAH\_MOUSE | ASAH1\_BALAS | CBPA1\_PIG | IDH\_OSTTA | IDUA\_HUMAN | LICH\_HUMAN | LIPG\_HUMAN | MDLA\_PENCA | MDLA\_PENCY | MNLOX\_MAGO7 | PA21B\_BOVIN | PA21B\_PIG | PA2GX\_HUMAN | PAG15\_HUMAN | PGH2\_HUMAN | PTGDS\_HUMAN | PTGDS\_MOUSE | Q6NY42\_DANRE | Q7LST4\_PENEN | W6Q990\_PENRF | GO:0019693 | 0.0679702048417132 | 219/3222 | 3/552 | 1 | 1 | F | F | F | F | ribose phosphate metabolic process | LICH\_HUMAN | PPT1\_HUMAN | PPT2\_HUMAN | GO:0034641 | 0.251396648044693 | 810/3222 | 32/552 | 1 | 1 | F | F | F | F | cellular nitrogen compound metabolic process | ADA2\_HUMAN | ANG1\_BOVIN | ANG2\_MOUSE | ANG3\_MOUSE | ANG4\_MOUSE | ANGI\_MOUSE | ASM3A\_HUMAN | ASM3A\_MOUSE | DNAS1\_HUMAN | DNSL3\_HUMAN | DOPO\_HUMAN | ECP\_HUMAN | ENDO2\_ARATH | GBA1\_HUMAN | IDH\_OSTTA | LICH\_HUMAN | NUP1\_PENCI | NUS1\_ASPOR | PPA5\_HUMAN | PPA5\_RAT | PPAP\_RAT | PPT1\_HUMAN | PPT2\_HUMAN | Q0KFV0\_SOLLC | Q5WRG2\_RAT | RNAS6\_HUMAN | RNLE\_SOLLC | RNT2\_HUMAN | TTHY\_CHICK | TTHY\_MOUSE | TTHY\_RAT | V5NTD\_NAJAT | GO:0044282 | 0.11887026691496 | 383/3222 | 4/552 | 1 | 1 | F | F | F | F | small molecule catabolic process | ADA2\_HUMAN | IDUA\_HUMAN | PAG15\_HUMAN | PERL\_CAPHI | GO:0006807 | 0.511483550589696 | 1648/3222 | 173/552 | 1 | 1 | F | F | F | F | nitrogen compound metabolic process | A0A0R3QSA7\_9BILA | A0A0R4I979\_BRABE | A0A3B6UEQ2\_RHIMI | A0A3S5H5N2\_LEIDO | A0A6P6YAT6\_DERPT | A0A7S6G7I6\_9PEZI | A0NFU8\_ANOGA | A4GX63\_TOXGO | A5AB48\_ASPNC | A6PZ97\_SALSA | A9LI60\_BIOOC | A9ZSX9\_9BRYO | ADA2\_HUMAN | AGAL\_HUMAN | ANAG\_HUMAN | ANG1\_BOVIN | ANG2\_MOUSE | ANG3\_MOUSE | ANG4\_MOUSE | ANGI\_MOUSE | AOC1\_HUMAN | AOCX\_BOVIN | ASAH1\_BALAS | ASM3A\_HUMAN | ASM3A\_MOUSE | B4F320\_LIMPO | B9TU22\_GADMO | BGLR\_HUMAN | CARP1\_CANAL | CARP2\_CANAX | CARP\_RHIPU | CATD\_RAT | CATH\_HUMAN | CATLL\_FASHE | CBPA1\_PIG | CBPD\_LOPSP | CBPN\_HUMAN | CDA\_COLLN | CDA\_EMENI | CEL2A\_PIG | CFAD\_MOUSE | CHI1\_COCPS | CHI2\_HORVU | CHI2\_ORYSJ | CHI33\_TRIHA | CHI42\_TRIHA | CHI4\_CRYJA | CHIA\_HUMAN | CHIC\_ARATH | CHIC\_SECCE | CHIL3\_MOUSE | CHIT\_PUNGR | CHLY\_HEVBR | CHYM\_CAMDR | CKX1\_MAIZE | COGS\_HYPLI | CUCM1\_CUCME | CYP5\_CAEEL | CYSP\_BLOTA | D6XHE1\_TRYB2 | DDN1\_BOVIN | DNAS1\_HUMAN | DNSL3\_HUMAN | DOPO\_HUMAN | DPP2\_HUMAN | ECP\_HUMAN | EGFB2\_MOUSE | ENDO2\_ARATH | ENPP2\_HUMAN | ENPP2\_RAT | ERVB\_TABDI | FUCO\_HUMAN | G3I1H5\_CRIGR | G3JPF7\_CORMM | GBA1\_HUMAN | GGH\_HUMAN | GRAA\_HUMAN | GRAC\_MOUSE | GRAK\_HUMAN | GRASS\_DROME | HE12\_DANRE | HEXC\_OSTFU | HS3S1\_MOUSE | HYAL1\_HUMAN | IDH\_OSTTA | IDUA\_HUMAN | J7LCB0\_DEIAC | K7CID1\_PANTR | KLK10\_HUMAN | KLK1\_HUMAN | KLK2\_HORSE | KLK2\_HUMAN | KLK7\_HUMAN | KLK7\_MOUSE | KLK8\_MOUSE | LAPA\_ASPOR | LGMN\_MOUSE | LICH\_HUMAN | LIPR2\_HUMAN | LIPR2\_RAT | LYG\_STRCA | MAN12\_PENCI | MANBA\_MOUSE | MCPT2\_RAT | MMP1\_PIG | NCS\_THLFG | NUP1\_PENCI | NUS1\_ASPOR | O81226\_CARPA | O81934\_CANEN | O97389\_HELAM | OFUT1\_CAEEL | PA21B\_BOVIN | PA21B\_PIG | PA2GA\_HUMAN | PA2GE\_HUMAN | PA2GX\_HUMAN | PAG15\_HUMAN | PCP\_HUMAN | PEPA\_ASPPH | PGPSA\_DROME | PGRP1\_CAMDR | PPA5\_HUMAN | PPA5\_RAT | PPAF1\_HOLDI | PPAP\_RAT | PPT1\_BOVIN | PPT1\_HUMAN | PPT2\_HUMAN | PRS57\_HUMAN | PRTN3\_HUMAN | Q06AK3\_TOXGO | Q0KFV0\_SOLLC | Q43576\_TOBAC | Q4AE59\_OSTFU | Q5B038\_EMENI | Q5WRG2\_RAT | Q69G21\_TENMO | Q6NY42\_DANRE | Q6R7Z5\_9TRYP | Q6WSR8\_PICAB | Q7YXL2\_TENMO | Q86RS6\_MANSE | Q8H0C9\_VIGUN | Q96X16\_PICPA | Q9FUH3\_VIGUS | QPCT1\_DROME | QPCT2\_DROME | QPCT\_IXOSC | QPCT\_MOUSE | RENI\_RAT | RNAS6\_HUMAN | RNLE\_SOLLC | RNT2\_HUMAN | SIA\_ASPFU | TPP1\_HUMAN | TRFL\_BUBBU | TRFL\_HORSE | TRY1\_GADMO | TRY3\_SALSA | TRYB2\_HUMAN | TTHY\_CHICK | TTHY\_MOUSE | TTHY\_RAT | V5NTD\_NAJAT | VM11\_BOTMO | VM12\_CROAD | VM1A3\_DEIAC | VM1BI\_BOTMO | VM1T1\_PROMU | VM1T2\_PROFL | VSPP\_DEIAC | VSPSX\_GLOSA | GO:1901293 | 0.068901303538175 | 222/3222 | 3/552 | 1 | 1 | F | F | F | F | nucleoside phosphate biosynthetic process | LICH\_HUMAN | PPT1\_HUMAN | PPT2\_HUMAN | GO:0016054 | 0.0732464307883302 | 236/3222 | 2/552 | 1 | 1 | F | F | F | F | organic acid catabolic process | IDUA\_HUMAN | PAG15\_HUMAN | GO:0016053 | 0.10397268777157 | 335/3222 | 10/552 | 1 | 1 | F | F | F | F | organic acid biosynthetic process | MDLA\_PENCA | MDLA\_PENCY | PA21B\_BOVIN | PA21B\_PIG | PA2GX\_HUMAN | PGH2\_HUMAN | PTGDS\_HUMAN | PTGDS\_MOUSE | Q7LST4\_PENEN | W6Q990\_PENRF | GO:1901566 | 0.197703289882061 | 637/3222 | 8/552 | 1 | 1 | F | F | F | F | organonitrogen compound biosynthetic process | ADA2\_HUMAN | DOPO\_HUMAN | GBA1\_HUMAN | HS3S1\_MOUSE | HYAL1\_HUMAN | LICH\_HUMAN | PPT1\_HUMAN | PPT2\_HUMAN | GO:0019752 | 0.284916201117318 | 918/3222 | 20/552 | 1 | 1 | F | F | F | F | carboxylic acid metabolic process | AOAH\_MOUSE | ASAH1\_BALAS | CBPA1\_PIG | IDH\_OSTTA | IDUA\_HUMAN | LICH\_HUMAN | LIPG\_HUMAN | MDLA\_PENCA | MDLA\_PENCY | MNLOX\_MAGO7 | PA21B\_BOVIN | PA21B\_PIG | PA2GX\_HUMAN | PAG15\_HUMAN | PGH2\_HUMAN | PTGDS\_HUMAN | PTGDS\_MOUSE | Q6NY42\_DANRE | Q7LST4\_PENEN | W6Q990\_PENRF | GO:0006753 | 0.114214773432651 | 368/3222 | 8/552 | 1 | 1 | F | F | F | F | nucleoside phosphate metabolic process | ASM3A\_HUMAN | ASM3A\_MOUSE | IDH\_OSTTA | LICH\_HUMAN | PPAP\_RAT | PPT1\_HUMAN | PPT2\_HUMAN | V5NTD\_NAJAT | GO:0046483 | 0.253879577901924 | 818/3222 | 31/552 | 1 | 1 | F | F | F | F | heterocycle metabolic process | ADA2\_HUMAN | ANG1\_BOVIN | ANG2\_MOUSE | ANG3\_MOUSE | ANG4\_MOUSE | ANGI\_MOUSE | ASM3A\_HUMAN | ASM3A\_MOUSE | CKX1\_MAIZE | DNAS1\_HUMAN | DNSL3\_HUMAN | ECP\_HUMAN | ENDO2\_ARATH | GGH\_HUMAN | IDH\_OSTTA | LICH\_HUMAN | NUP1\_PENCI | NUS1\_ASPOR | PPAP\_RAT | PPT1\_HUMAN | PPT2\_HUMAN | Q0KFV0\_SOLLC | Q5WRG2\_RAT | Q6NY42\_DANRE | RNAS6\_HUMAN | RNLE\_SOLLC | RNT2\_HUMAN | TTHY\_CHICK | TTHY\_MOUSE | TTHY\_RAT | V5NTD\_NAJAT | GO:1901615 | 0.0983860955927995 | 317/3222 | 8/552 | 1 | 1 | F | F | F | F | organic hydroxy compound metabolic process | DOPO\_HUMAN | GBA1\_HUMAN | IDH\_OSTTA | LICH\_HUMAN | LIP3\_DIURU | PPAP\_RAT | TTHY\_CHICK | TTHY\_RAT | GO:0055010 | 0.000310366232153942 | 1/3222 | 1/552 | NA | NA | NA | NA | NA | NA | ventricular cardiac muscle tissue morphogenesis | ANAG\_HUMAN | GO:0002444 | 0.000310366232153942 | 1/3222 | 1/552 | NA | NA | NA | NA | NA | NA | myeloid leukocyte mediated immunity | PA21B\_PIG | GO:0021675 | 0.000310366232153942 | 1/3222 | 1/552 | NA | NA | NA | NA | NA | NA | nerve development | ANAG\_HUMAN | GO:0021859 | 0.000310366232153942 | 1/3222 | 1/552 | NA | NA | NA | NA | NA | NA | pyramidal neuron differentiation | GBA1\_HUMAN | GO:0007028 | 0.000620732464307883 | 2/3222 | 1/552 | NA | NA | NA | NA | NA | NA | cytoplasm organization | ANAG\_HUMAN | GO:0070198 | 0.000310366232153942 | 1/3222 | 1/552 | NA | NA | NA | NA | NA | NA | protein localization to chromosome, telomeric region | TPP1\_HUMAN | GO:0071333 | 0.00217256362507759 | 7/3222 | 1/552 | NA | NA | NA | NA | NA | NA | cellular response to glucose stimulus | ANG2\_MOUSE | GO:0001555 | 0.000310366232153942 | 1/3222 | 1/552 | NA | NA | NA | NA | NA | NA | oocyte growth | TTHY\_CHICK | GO:0002220 | 0.000310366232153942 | 1/3222 | 1/552 | NA | NA | NA | NA | NA | NA | innate immune response activating cell surface receptor signaling pathway | ANAG\_HUMAN | GO:0036294 | 0.00682805710738672 | 22/3222 | 1/552 | NA | NA | NA | NA | NA | NA | cellular response to decreased oxygen levels | PGH2\_HUMAN | GO:0061436 | 0.000310366232153942 | 1/3222 | 1/552 | NA | NA | NA | NA | NA | NA | establishment of skin barrier | GBA1\_HUMAN | GO:0050767 | 0.00217256362507759 | 7/3222 | 1/552 | NA | NA | NA | NA | NA | NA | regulation of neurogenesis | ENPP2\_RAT | GO:0071887 | 0.000931098696461825 | 3/3222 | 1/552 | NA | NA | NA | NA | NA | NA | leukocyte apoptotic process | LICH\_HUMAN | GO:0051962 | 0.00186219739292365 | 6/3222 | 1/552 | NA | NA | NA | NA | NA | NA | positive regulation of nervous system development | ENPP2\_RAT | GO:0042698 | 0.000620732464307883 | 2/3222 | 1/552 | NA | NA | NA | NA | NA | NA | ovulation cycle | ENPP2\_RAT | GO:1903496 | 0.000310366232153942 | 1/3222 | 1/552 | NA | NA | NA | NA | NA | NA | response to 11-deoxycorticosterone | LALBA\_BOVIN | GO:0097029 | 0.000310366232153942 | 1/3222 | 1/552 | NA | NA | NA | NA | NA | NA | mature conventional dendritic cell differentiation | PRTN3\_HUMAN | GO:0006805 | 0.0189323401613904 | 61/3222 | 1/552 | NA | NA | NA | NA | NA | NA | xenobiotic metabolic process | PERL\_CAPHI | GO:0006766 | 0.0288640595903166 | 93/3222 | 1/552 | NA | NA | NA | NA | NA | NA | vitamin metabolic process | PPAP\_RAT | GO:0007566 | 0.00155183116076971 | 5/3222 | 1/552 | NA | NA | NA | NA | NA | NA | embryo implantation | PGH2\_HUMAN | GO:0042267 | 0.000931098696461825 | 3/3222 | 1/552 | NA | NA | NA | NA | NA | NA | natural killer cell mediated cytotoxicity | GRAC\_MOUSE | GO:0003012 | 0.00434512725015518 | 14/3222 | 1/552 | NA | NA | NA | NA | NA | NA | muscle system process | ANG2\_MOUSE | GO:0000165 | 0.00279329608938547 | 9/3222 | 1/552 | NA | NA | NA | NA | NA | NA | MAPK cascade | CATH\_HUMAN | GO:0097009 | 0.000620732464307883 | 2/3222 | 1/552 | NA | NA | NA | NA | NA | NA | energy homeostasis | PCP\_HUMAN | GO:0002215 | 0.000310366232153942 | 1/3222 | 1/552 | NA | NA | NA | NA | NA | NA | defense response to nematode | PER53\_ARATH | GO:0032386 | 0.0037243947858473 | 12/3222 | 1/552 | NA | NA | NA | NA | NA | NA | regulation of intracellular transport | PGH2\_HUMAN | GO:0006825 | 0.000310366232153942 | 1/3222 | 1/552 | NA | NA | NA | NA | NA | NA | copper ion transport | CERU\_RAT | GO:0032303 | 0.00186219739292365 | 6/3222 | 1/552 | NA | NA | NA | NA | NA | NA | regulation of icosanoid secretion | PA2GX\_HUMAN | GO:0140448 | 0.00124146492861577 | 4/3222 | 1/552 | NA | NA | NA | NA | NA | NA | signaling receptor ligand precursor processing | RENI\_RAT | GO:0034765 | 0.00620732464307883 | 20/3222 | 1/552 | NA | NA | NA | NA | NA | NA | regulation of monoatomic ion transmembrane transport | PA21B\_PIG | GO:0006680 | 0.000620732464307883 | 2/3222 | 1/552 | NA | NA | NA | NA | NA | NA | glucosylceramide catabolic process | GBA1\_HUMAN | GO:0140353 | 0.000310366232153942 | 1/3222 | 1/552 | NA | NA | NA | NA | NA | NA | lipid export from cell | PGH2\_HUMAN | GO:0071684 | 0.000310366232153942 | 1/3222 | 1/552 | NA | NA | NA | NA | NA | NA | organism emergence from protective structure | HE12\_DANRE | GO:1902219 | 0.000310366232153942 | 1/3222 | 1/552 | NA | NA | NA | NA | NA | NA | negative regulation of intrinsic apoptotic signaling pathway in response to osmotic stress | PGH2\_HUMAN | GO:0044003 | 0.00186219739292365 | 6/3222 | 1/552 | NA | NA | NA | NA | NA | NA | modulation by symbiont of host process | CARP1\_CANAL | GO:0032372 | 0.000310366232153942 | 1/3222 | 1/552 | NA | NA | NA | NA | NA | NA | negative regulation of sterol transport | PA2GX\_HUMAN | GO:0052173 | 0.00186219739292365 | 6/3222 | 1/552 | NA | NA | NA | NA | NA | NA | response to defenses of other organism | CARP1\_CANAL | GO:0032070 | 0.000620732464307883 | 2/3222 | 1/552 | NA | NA | NA | NA | NA | NA | regulation of deoxyribonuclease activity | GRAA\_HUMAN | GO:0033591 | 0.00155183116076971 | 5/3222 | 1/552 | NA | NA | NA | NA | NA | NA | response to L-ascorbic acid | PPA5\_RAT | GO:0090237 | 0.00124146492861577 | 4/3222 | 1/552 | NA | NA | NA | NA | NA | NA | regulation of arachidonic acid secretion | PA2GX\_HUMAN | GO:0048580 | 0.00186219739292365 | 6/3222 | 1/552 | NA | NA | NA | NA | NA | NA | regulation of post-embryonic development | AGAL\_ORYSJ | GO:0030282 | 0.000931098696461825 | 3/3222 | 1/552 | NA | NA | NA | NA | NA | NA | bone mineralization | PGH2\_HUMAN | GO:0051930 | 0.000931098696461825 | 3/3222 | 1/552 | NA | NA | NA | NA | NA | NA | regulation of sensory perception of pain | PPAP\_RAT | GO:0032436 | 0.000310366232153942 | 1/3222 | 1/552 | NA | NA | NA | NA | NA | NA | positive regulation of proteasomal ubiquitin-dependent protein catabolic process | GBA1\_HUMAN | GO:0002791 | 0.00341402855369336 | 11/3222 | 1/552 | NA | NA | NA | NA | NA | NA | regulation of peptide secretion | CEL2A\_PIG | GO:0098900 | 0.000620732464307883 | 2/3222 | 1/552 | NA | NA | NA | NA | NA | NA | regulation of action potential | GBA1\_HUMAN | GO:0045730 | 0.000931098696461825 | 3/3222 | 1/552 | NA | NA | NA | NA | NA | NA | respiratory burst | LICH\_HUMAN | GO:0031650 | 0.000620732464307883 | 2/3222 | 1/552 | NA | NA | NA | NA | NA | NA | regulation of heat generation | PGH2\_HUMAN | GO:0071498 | 0.000931098696461825 | 3/3222 | 1/552 | NA | NA | NA | NA | NA | NA | cellular response to fluid shear stress | PGH2\_HUMAN | GO:0046496 | 0.0248292985723153 | 80/3222 | 1/552 | NA | NA | NA | NA | NA | NA | nicotinamide nucleotide metabolic process | IDH\_OSTTA | GO:0043616 | 0.000310366232153942 | 1/3222 | 1/552 | NA | NA | NA | NA | NA | NA | keratinocyte proliferation | KLK8\_MOUSE | GO:0030205 | 0.000620732464307883 | 2/3222 | 1/552 | NA | NA | NA | NA | NA | NA | dermatan sulfate metabolic process | IDUA\_HUMAN | GO:0002155 | 0.000310366232153942 | 1/3222 | 1/552 | NA | NA | NA | NA | NA | NA | regulation of thyroid hormone mediated signaling pathway | PCP\_HUMAN | GO:0044245 | 0.000310366232153942 | 1/3222 | 1/552 | NA | NA | NA | NA | NA | NA | polysaccharide digestion | CHIA\_HUMAN | GO:0015914 | 0.000620732464307883 | 2/3222 | 1/552 | NA | NA | NA | NA | NA | NA | phospholipid transport | PA2GX\_HUMAN | GO:0052782 | 0.000620732464307883 | 2/3222 | 1/552 | NA | NA | NA | NA | NA | NA | amino disaccharide catabolic process | IDUA\_HUMAN | GO:0051969 | 0.000620732464307883 | 2/3222 | 1/552 | NA | NA | NA | NA | NA | NA | regulation of transmission of nerve impulse | GBA1\_HUMAN | GO:0002003 | 0.000310366232153942 | 1/3222 | 1/552 | NA | NA | NA | NA | NA | NA | angiotensin maturation | RENI\_RAT | GO:0032675 | 0.000620732464307883 | 2/3222 | 1/552 | NA | NA | NA | NA | NA | NA | regulation of interleukin-6 production | GBA1\_HUMAN | GO:1905962 | 0.000310366232153942 | 1/3222 | 1/552 | NA | NA | NA | NA | NA | NA | glutamatergic neuron differentiation | ANAG\_HUMAN | GO:0002218 | 0.00186219739292365 | 6/3222 | 1/552 | NA | NA | NA | NA | NA | NA | activation of innate immune response | ANAG\_HUMAN | GO:0010975 | 0.00279329608938547 | 9/3222 | 1/552 | NA | NA | NA | NA | NA | NA | regulation of neuron projection development | KLK8\_MOUSE | GO:0071322 | 0.00279329608938547 | 9/3222 | 1/552 | NA | NA | NA | NA | NA | NA | cellular response to carbohydrate stimulus | ANG2\_MOUSE | GO:0051069 | 0.000310366232153942 | 1/3222 | 1/552 | NA | NA | NA | NA | NA | NA | galactomannan metabolic process | AGAL\_ORYSJ | GO:0043280 | 0.00155183116076971 | 5/3222 | 1/552 | NA | NA | NA | NA | NA | NA | positive regulation of cysteine-type endopeptidase activity involved in apoptotic process | CATD\_RAT | GO:0035874 | 0.000310366232153942 | 1/3222 | 1/552 | NA | NA | NA | NA | NA | NA | cellular response to copper ion starvation | AOC1\_HUMAN | GO:0002269 | 0.00124146492861577 | 4/3222 | 1/552 | NA | NA | NA | NA | NA | NA | leukocyte activation involved in inflammatory response | ANAG\_HUMAN | GO:0048512 | 0.000931098696461825 | 3/3222 | 1/552 | NA | NA | NA | NA | NA | NA | circadian behavior | ANAG\_HUMAN | GO:0019884 | 0.000310366232153942 | 1/3222 | 1/552 | NA | NA | NA | NA | NA | NA | antigen processing and presentation of exogenous antigen | GILT\_MOUSE | GO:0050848 | 0.00248292985723153 | 8/3222 | 1/552 | NA | NA | NA | NA | NA | NA | regulation of calcium-mediated signaling | CARP1\_CANAL | GO:0005987 | 0.000310366232153942 | 1/3222 | 1/552 | NA | NA | NA | NA | NA | NA | sucrose catabolic process | AMY1\_ORYSJ | GO:0042461 | 0.000310366232153942 | 1/3222 | 1/552 | NA | NA | NA | NA | NA | NA | photoreceptor cell development | ANAG\_HUMAN | GO:0071222 | 0.00403476101800124 | 13/3222 | 1/552 | NA | NA | NA | NA | NA | NA | cellular response to lipopolysaccharide | ANG4\_MOUSE | GO:0007005 | 0.00713842333954066 | 23/3222 | 1/552 | NA | NA | NA | NA | NA | NA | mitochondrion organization | LICH\_HUMAN | GO:0009911 | 0.000310366232153942 | 1/3222 | 1/552 | NA | NA | NA | NA | NA | NA | positive regulation of flower development | AGAL\_ORYSJ | GO:0006836 | 0.000931098696461825 | 3/3222 | 1/552 | NA | NA | NA | NA | NA | NA | neurotransmitter transport | PPT1\_HUMAN | GO:0044060 | 0.000620732464307883 | 2/3222 | 1/552 | NA | NA | NA | NA | NA | NA | regulation of endocrine process | RENI\_RAT | GO:1901888 | 0.00155183116076971 | 5/3222 | 1/552 | NA | NA | NA | NA | NA | NA | regulation of cell junction assembly | ENPP2\_RAT | GO:0015679 | 0.000310366232153942 | 1/3222 | 1/552 | NA | NA | NA | NA | NA | NA | plasma membrane copper ion transport | CERU\_RAT | GO:0046103 | 0.00186219739292365 | 6/3222 | 1/552 | NA | NA | NA | NA | NA | NA | inosine biosynthetic process | ADA2\_HUMAN | GO:0002768 | 0.00124146492861577 | 4/3222 | 1/552 | NA | NA | NA | NA | NA | NA | immune response-regulating cell surface receptor signaling pathway | ANAG\_HUMAN | GO:1905392 | 0.000310366232153942 | 1/3222 | 1/552 | NA | NA | NA | NA | NA | NA | plant organ morphogenesis | AGAL\_ORYSJ | GO:0046129 | 0.00993171942892613 | 32/3222 | 1/552 | NA | NA | NA | NA | NA | NA | purine ribonucleoside biosynthetic process | ADA2\_HUMAN | GO:0051385 | 0.000931098696461825 | 3/3222 | 1/552 | NA | NA | NA | NA | NA | NA | response to mineralocorticoid | LALBA\_BOVIN | GO:0033365 | 0.00496585971446307 | 16/3222 | 1/552 | NA | NA | NA | NA | NA | NA | protein localization to organelle | TPP1\_HUMAN | GO:0014015 | 0.00124146492861577 | 4/3222 | 1/552 | NA | NA | NA | NA | NA | NA | positive regulation of gliogenesis | ENPP2\_RAT | GO:0031915 | 0.000310366232153942 | 1/3222 | 1/552 | NA | NA | NA | NA | NA | NA | positive regulation of synaptic plasticity | PGH2\_HUMAN | GO:0055008 | 0.000310366232153942 | 1/3222 | 1/552 | NA | NA | NA | NA | NA | NA | cardiac muscle tissue morphogenesis | ANAG\_HUMAN | GO:0019614 | 0.00186219739292365 | 6/3222 | 1/552 | NA | NA | NA | NA | NA | NA | catechol-containing compound catabolic process | DOPO\_HUMAN | GO:1904350 | 0.000620732464307883 | 2/3222 | 1/552 | NA | NA | NA | NA | NA | NA | regulation of protein catabolic process in the vacuole | GBA1\_HUMAN | GO:0043152 | 0.000310366232153942 | 1/3222 | 1/552 | NA | NA | NA | NA | NA | NA | induction of bacterial agglutination | ECP\_HUMAN | GO:0034381 | 0.000310366232153942 | 1/3222 | 1/552 | NA | NA | NA | NA | NA | NA | plasma lipoprotein particle clearance | LICH\_HUMAN | GO:0031392 | 0.000310366232153942 | 1/3222 | 1/552 | NA | NA | NA | NA | NA | NA | regulation of prostaglandin biosynthetic process | PGH2\_HUMAN | GO:0042417 | 0.0037243947858473 | 12/3222 | 1/552 | NA | NA | NA | NA | NA | NA | dopamine metabolic process | DOPO\_HUMAN | GO:0043407 | 0.00155183116076971 | 5/3222 | 1/552 | NA | NA | NA | NA | NA | NA | negative regulation of MAP kinase activity | GBA1\_HUMAN | GO:0022617 | 0.000310366232153942 | 1/3222 | 1/552 | NA | NA | NA | NA | NA | NA | extracellular matrix disassembly | KLK7\_HUMAN | GO:0043627 | 0.00155183116076971 | 5/3222 | 1/552 | NA | NA | NA | NA | NA | NA | response to estrogen | GBA1\_HUMAN | GO:0001659 | 0.00155183116076971 | 5/3222 | 1/552 | NA | NA | NA | NA | NA | NA | temperature homeostasis | DOPO\_HUMAN | GO:0009712 | 0.00651769087523277 | 21/3222 | 1/552 | NA | NA | NA | NA | NA | NA | catechol-containing compound metabolic process | DOPO\_HUMAN | GO:0046324 | 0.00124146492861577 | 4/3222 | 1/552 | NA | NA | NA | NA | NA | NA | regulation of glucose import | PA21B\_PIG | GO:0061900 | 0.000931098696461825 | 3/3222 | 1/552 | NA | NA | NA | NA | NA | NA | glial cell activation | ANAG\_HUMAN | GO:0032069 | 0.000931098696461825 | 3/3222 | 1/552 | NA | NA | NA | NA | NA | NA | regulation of nuclease activity | GRAA\_HUMAN | GO:0048514 | 0.000931098696461825 | 3/3222 | 1/552 | NA | NA | NA | NA | NA | NA | blood vessel morphogenesis | ANAG\_HUMAN | GO:0071636 | 0.000310366232153942 | 1/3222 | 1/552 | NA | NA | NA | NA | NA | NA | positive regulation of transforming growth factor beta production | PGH2\_HUMAN | GO:0007565 | 0.00248292985723153 | 8/3222 | 1/552 | NA | NA | NA | NA | NA | NA | female pregnancy | CERU\_RAT | GO:0090362 | 0.000310366232153942 | 1/3222 | 1/552 | NA | NA | NA | NA | NA | NA | positive regulation of platelet-derived growth factor production | PGH2\_HUMAN | GO:0098916 | 0.00186219739292365 | 6/3222 | 1/552 | NA | NA | NA | NA | NA | NA | anterograde trans-synaptic signaling | DOPO\_HUMAN | GO:0052572 | 0.000620732464307883 | 2/3222 | 1/552 | NA | NA | NA | NA | NA | NA | response to host immune response | CARP1\_CANAL | GO:0010874 | 0.000931098696461825 | 3/3222 | 1/552 | NA | NA | NA | NA | NA | NA | regulation of cholesterol efflux | PA2GX\_HUMAN | GO:0006754 | 0.00310366232153942 | 10/3222 | 1/552 | NA | NA | NA | NA | NA | NA | ATP biosynthetic process | LICH\_HUMAN | GO:0009909 | 0.000310366232153942 | 1/3222 | 1/552 | NA | NA | NA | NA | NA | NA | regulation of flower development | AGAL\_ORYSJ | GO:0032757 | 0.000620732464307883 | 2/3222 | 1/552 | NA | NA | NA | NA | NA | NA | positive regulation of interleukin-8 production | PA21B\_PIG | GO:1900024 | 0.000310366232153942 | 1/3222 | 1/552 | NA | NA | NA | NA | NA | NA | regulation of substrate adhesion-dependent cell spreading | ENPP2\_RAT | GO:0018205 | 0.00837988826815642 | 27/3222 | 1/552 | NA | NA | NA | NA | NA | NA | peptidyl-lysine modification | Q5B038\_EMENI | GO:0009749 | 0.00558659217877095 | 18/3222 | 1/552 | NA | NA | NA | NA | NA | NA | response to glucose | ANG2\_MOUSE | GO:1904389 | 0.000310366232153942 | 1/3222 | 1/552 | NA | NA | NA | NA | NA | NA | rod bipolar cell differentiation | ANAG\_HUMAN | GO:0055006 | 0.000310366232153942 | 1/3222 | 1/552 | NA | NA | NA | NA | NA | NA | cardiac cell development | ANAG\_HUMAN | GO:0044269 | 0.000620732464307883 | 2/3222 | 1/552 | NA | NA | NA | NA | NA | NA | glycerol ether catabolic process | PA2GX\_HUMAN | GO:1901804 | 0.00155183116076971 | 5/3222 | 1/552 | NA | NA | NA | NA | NA | NA | beta-glucoside metabolic process | GBA1\_HUMAN | GO:2000191 | 0.00186219739292365 | 6/3222 | 1/552 | NA | NA | NA | NA | NA | NA | regulation of fatty acid transport | PA2GX\_HUMAN | GO:0015012 | 0.00124146492861577 | 4/3222 | 1/552 | NA | NA | NA | NA | NA | NA | heparan sulfate proteoglycan biosynthetic process | HS3S1\_MOUSE | GO:0050953 | 0.00155183116076971 | 5/3222 | 1/552 | NA | NA | NA | NA | NA | NA | sensory perception of light stimulus | PPT1\_HUMAN | GO:0003183 | 0.000310366232153942 | 1/3222 | 1/552 | NA | NA | NA | NA | NA | NA | mitral valve morphogenesis | ANAG\_HUMAN | GO:0050807 | 0.00186219739292365 | 6/3222 | 1/552 | NA | NA | NA | NA | NA | NA | regulation of synapse organization | KLK8\_MOUSE | GO:0090316 | 0.00124146492861577 | 4/3222 | 1/552 | NA | NA | NA | NA | NA | NA | positive regulation of intracellular protein transport | PGH2\_HUMAN | GO:0048265 | 0.000620732464307883 | 2/3222 | 1/552 | NA | NA | NA | NA | NA | NA | response to pain | DOPO\_HUMAN | GO:0045936 | 0.00651769087523277 | 21/3222 | 1/552 | NA | NA | NA | NA | NA | NA | negative regulation of phosphate metabolic process | GBA1\_HUMAN | GO:0022900 | 0.00248292985723153 | 8/3222 | 1/552 | NA | NA | NA | NA | NA | NA | electron transport chain | GBA1\_HUMAN | GO:0002262 | 0.00217256362507759 | 7/3222 | 1/552 | NA | NA | NA | NA | NA | NA | myeloid cell homeostasis | LICH\_HUMAN | GO:0044277 | 0.000310366232153942 | 1/3222 | 1/552 | NA | NA | NA | NA | NA | NA | cell wall disassembly | ADPG2\_ARATH | GO:0051682 | 0.000310366232153942 | 1/3222 | 1/552 | NA | NA | NA | NA | NA | NA | galactomannan catabolic process | AGAL\_ORYSJ | GO:0010720 | 0.00434512725015518 | 14/3222 | 1/552 | NA | NA | NA | NA | NA | NA | positive regulation of cell development | ENPP2\_RAT | GO:0030595 | 0.00124146492861577 | 4/3222 | 1/552 | NA | NA | NA | NA | NA | NA | leukocyte chemotaxis | PA21B\_PIG | GO:0006074 | 0.000620732464307883 | 2/3222 | 1/552 | NA | NA | NA | NA | NA | NA | (1->3)-beta-D-glucan metabolic process | E13B\_HORVU | GO:0071425 | 0.000310366232153942 | 1/3222 | 1/552 | NA | NA | NA | NA | NA | NA | hematopoietic stem cell proliferation | GBA1\_HUMAN | GO:0019370 | 0.00217256362507759 | 7/3222 | 1/552 | NA | NA | NA | NA | NA | NA | leukotriene biosynthetic process | PA21B\_PIG | GO:0048681 | 0.000310366232153942 | 1/3222 | 1/552 | NA | NA | NA | NA | NA | NA | negative regulation of axon regeneration | KLK8\_MOUSE | GO:0045089 | 0.00248292985723153 | 8/3222 | 1/552 | NA | NA | NA | NA | NA | NA | positive regulation of innate immune response | ANAG\_HUMAN | GO:0046102 | 0.00341402855369336 | 11/3222 | 1/552 | NA | NA | NA | NA | NA | NA | inosine metabolic process | ADA2\_HUMAN | GO:0070723 | 0.00155183116076971 | 5/3222 | 1/552 | NA | NA | NA | NA | NA | NA | response to cholesterol | PPA5\_RAT | GO:0033028 | 0.000310366232153942 | 1/3222 | 1/552 | NA | NA | NA | NA | NA | NA | myeloid cell apoptotic process | LICH\_HUMAN | GO:0030855 | 0.00837988826815642 | 27/3222 | 1/552 | NA | NA | NA | NA | NA | NA | epithelial cell differentiation | TPP1\_HUMAN | GO:0043154 | 0.00279329608938547 | 9/3222 | 1/552 | NA | NA | NA | NA | NA | NA | negative regulation of cysteine-type endopeptidase activity involved in apoptotic process | PGH2\_HUMAN | GO:0007268 | 0.00186219739292365 | 6/3222 | 1/552 | NA | NA | NA | NA | NA | NA | chemical synaptic transmission | DOPO\_HUMAN | GO:1990823 | 0.0037243947858473 | 12/3222 | 1/552 | NA | NA | NA | NA | NA | NA | response to leukemia inhibitory factor | PA2GX\_HUMAN | GO:0009163 | 0.010552451893234 | 34/3222 | 1/552 | NA | NA | NA | NA | NA | NA | nucleoside biosynthetic process | ADA2\_HUMAN | GO:0042710 | 0.000310366232153942 | 1/3222 | 1/552 | NA | NA | NA | NA | NA | NA | biofilm formation | EXG1\_CANAL | GO:0043648 | 0.0406579764121664 | 131/3222 | 1/552 | NA | NA | NA | NA | NA | NA | dicarboxylic acid metabolic process | LIPG\_HUMAN | GO:0033280 | 0.000310366232153942 | 1/3222 | 1/552 | NA | NA | NA | NA | NA | NA | response to vitamin D | PGH2\_HUMAN | GO:0045745 | 0.000310366232153942 | 1/3222 | 1/552 | NA | NA | NA | NA | NA | NA | positive regulation of G protein-coupled receptor signaling pathway | PPAP\_RAT | GO:0009838 | 0.000310366232153942 | 1/3222 | 1/552 | NA | NA | NA | NA | NA | NA | abscission | ADPG2\_ARATH | GO:0009164 | 0.010862818125388 | 35/3222 | 1/552 | NA | NA | NA | NA | NA | NA | nucleoside catabolic process | ADA2\_HUMAN | GO:0048873 | 0.000620732464307883 | 2/3222 | 1/552 | NA | NA | NA | NA | NA | NA | homeostasis of number of cells within a tissue | LICH\_HUMAN | GO:0031394 | 0.000310366232153942 | 1/3222 | 1/552 | NA | NA | NA | NA | NA | NA | positive regulation of prostaglandin biosynthetic process | PGH2\_HUMAN | GO:0010811 | 0.000931098696461825 | 3/3222 | 1/552 | NA | NA | NA | NA | NA | NA | positive regulation of cell-substrate adhesion | ENPP2\_RAT | GO:0031333 | 0.000931098696461825 | 3/3222 | 1/552 | NA | NA | NA | NA | NA | NA | negative regulation of protein-containing complex assembly | GBA1\_HUMAN | GO:0031334 | 0.0037243947858473 | 12/3222 | 1/552 | NA | NA | NA | NA | NA | NA | positive regulation of protein-containing complex assembly | MMP1\_PIG | GO:0046173 | 0.0130353817504655 | 42/3222 | 1/552 | NA | NA | NA | NA | NA | NA | polyol biosynthetic process | GBA1\_HUMAN | GO:0009713 | 0.00217256362507759 | 7/3222 | 1/552 | NA | NA | NA | NA | NA | NA | catechol-containing compound biosynthetic process | DOPO\_HUMAN | GO:0046333 | 0.000310366232153942 | 1/3222 | 1/552 | NA | NA | NA | NA | NA | NA | octopamine metabolic process | DOPO\_HUMAN | GO:0006099 | 0.0133457479826195 | 43/3222 | 1/552 | NA | NA | NA | NA | NA | NA | tricarboxylic acid cycle | IDH\_OSTTA | GO:0043249 | 0.000620732464307883 | 2/3222 | 1/552 | NA | NA | NA | NA | NA | NA | erythrocyte maturation | PA2GX\_HUMAN | GO:0019362 | 0.0251396648044693 | 81/3222 | 1/552 | NA | NA | NA | NA | NA | NA | pyridine nucleotide metabolic process | IDH\_OSTTA | GO:0071260 | 0.000620732464307883 | 2/3222 | 1/552 | NA | NA | NA | NA | NA | NA | cellular response to mechanical stimulus | PGH2\_HUMAN | GO:0003044 | 0.00155183116076971 | 5/3222 | 1/552 | NA | NA | NA | NA | NA | NA | regulation of systemic arterial blood pressure mediated by a chemical signal | RENI\_RAT | GO:0071504 | 0.000310366232153942 |
[truncated: 99,297 more chars]
